# Helical ambivalency induced by point mutations

**DOI:** 10.1186/1472-6807-13-9

**Published:** 2013-05-15

**Authors:** Nicholus Bhattacharjee, Parbati Biswas

**Affiliations:** 1 Department of Chemistry, University of Delhi, Delhi-110007, India

## Abstract

**Background:**

Mutation of amino acid sequences in a protein may have diverse effects on its structure and function. Point mutations of even a single amino acid residue in the helices of the non-redundant database may lead to sequentially identical peptides which adopt different secondary structures in different proteins. However, various physico-chemical factors which govern the formation of these ambivalent helices generated by point mutations of a sequence are not clearly known.

**Results:**

Sequences generated by point mutations of helices are mapped on to their non-helical counterparts in the SCOP database. The results show that short helices are prone to transform into non-helical conformations upon point mutations. Mutation of amino acid residues by helix breakers preferentially yield non-helical conformations, while mutation with residues of intermediate helix propensity display least preferences for non-helical conformations. Differences in the solvent accessibility of the mutating/mutated residues are found to be a major criteria for these sequences to conform to non-helical conformations. Even with minimal differences in the amino acid distributions of the sequences flanking the helical and non-helical conformations, helix-flanking sequences are found be more solvent accessible.

**Conclusions:**

All types of mutations from helical to non-helical conformations are investigated. The primary factors attributing such changes in conformation can be: i) type/propensity of the mutating and mutant residues ii) solvent accessibility of the residue at the mutation site iii) context/environment dependence of the flanking sequences. The results from the present study may be used to design *de novo* proteins via point mutations.

## Background

Under physiological conditions, folding of proteins to well-defined three dimensional structures is crucial for executing their specific biological functions. To the extent that the folding pattern of proteins dictates function, modifying the structure may entail in either altering or disrupting its function. Point mutations in a protein sequence, which are introduced by single amino acid residue replacements by site-directed mutagenesis, may yield different phenotypes by changing the native structure. The effect of point mutations are important in exploring various functional and structural features viz. protein sequenceŰstructure relationships [1], engineering protein stability [2,3], predicting the evolutionary dynamics of proteins [4-7], validating and refining various protein models and simulations [8-10], and designing *de novo* proteins [11]. Mutations may also influence the local structure of a protein by changing the secondary structures of peptide sequences from one conformation to another [12,13]. It is established that the inherited prion diseases of humans are all linked to specific point and insertion mutations, which are responsible for the *α*→*β* transition leading to aggregation due to the formation of amyloid fibrils [14]. Similar conclusions are drawn from the theoretical studies of prion protein [15].

Experimental studies of mutating different positions in a protein structure with various amino acid residues are time-consuming and expensive process. Such an investigation is facilitated by the three-dimensional modeling of polypeptide chain mutations. Experimental determinations of secondary structure propensity and stability [16-19] are usually studied through point mutations. The results obtained from the mutation of helices are used for rationalizing helix-coil transitions and compared to that of Lifson-Roig [20] and Zimm-Bragg [21] models. However, the knowledge of the type and position of mutations required for the transformation of helical → non-helical conformation or vice versa is largely unclear. The computational demands of such problems limit the number of mutations and size of sequences that may be considered. Alternatively, crystallographic structures from the Protein Data Bank [22] may be used as templates to explore point mutations leading to helical ambivalency.

The present work aims at investigating the physico-chemical properties of ambivalent helices generated by point mutations. Ambivalent sequences conform to different secondary structures in two different proteins. Kabsch and Sander [23] first reported the occurrence and physico-chemical properties of these sequences which were studied in great detail in the subsequent works [24-30]. Importance of these sequences lie in their implication in the pathogenesis of amyloid diseases including Alzheimer’s disease, transmissible bovine spongiform encephalopathy etc [31,32]. These sequences also challenge the existing protein structure prediction methods based on sequence homology [33]. Recently we have shown that variable (ambivalent) helices, sequences which are in helical conformations in one protein and non-helical in other, have different physico-chemical, context and dynamical properties in comparison to conserved helices i.e. sequences which remain in helical conformation throughout [34-36]. Although Argos [24] reported ambivalent sequences which differ by a single amino acid, no subsequent studies on the occurrence and properties of these sequences were performed. However, these type of sequences are important as point mutations may cause secondary structure transformation, which may have significant implications in various misfolding diseases. Recent studies demonstrate that a conformational switch between two proteins with completely different structures and functions occurs via a single amino acid mutation [37].

This switching between the folds can occur in multiple ways via successive single amino acid mutation at different positions [38]. Both theoretical [39] and computational [40] studies have highlighted the bifunctionality of proteins via single point mutation.

This work presents a detailed study of helical ambivalency induced by point mutations. Helices from the non-redundant database are point mutated at all residue positions and the resulting sequences are mapped onto the SCOP database to obtain completely non-helical conformations. Previous analysis of physico-chemical properties of ambivalent sequences were restricted to the local structure neglecting the global influence of the other parts of the protein [25,27-30,34]. Along similar lines, the present study also analyzes the physico-chemical properties of ambivalent sequences induced by point mutations disregarding the effect on the remaining parts of the protein. The results show that smaller helices readily transform into non-helical conformations after point mutations. Sequences obtained by point mutations with helix-breaking residues usually correspond to non-helical conformations in the SCOP database. However, point mutations of helix indifferent residues yield mutated sequences which has a low probability of conforming to non-helical conformations. Differences in the solvent accessibility of the mutating/mutated residues in helical and non-helical conformations is found to be a major factor for the mutated sequence conforming to non-helical structures. Most of the mutated residues display different degree of solvent accessibility in helical and non-helical conformations. The non-helical conformations are found to be less solvent-accessible as compared to the original helices. Despite marginal differences in the amino acid distributions of the sequences flanking helical and non-helical conformations, the solvent accessibility of the sequences flanking helices are greater than that of the corresponding non-helical conformations.

## Methods

### Database and mutation

The database used in the present study comprises the crystal structures from May-2008 release of PDB-select [41], which were compiled to create a database of non-redundant proteins from PDB [22] (Protein Data Bank). The database comprises protein chains with a sequence identity of 25% or less. All protein chains considered in this study have resolution ≤ 3Å and crystallographic *R*-factor, *R*≤0.3. The selected database consists of 2586 non-redundant protein chains from 2466 protein structures. Secondary structures are annotated residue-wise with the help of DSSP software [42]. According to the widely used definition, H and G denote helical conformations while all other classes (B, E, I, S, T, -) are considered to be non-helical [43-45]. Neglecting helices with less than 5 residues long, there are 11592 helices in the non-redundant database. These helices were point mutated at each position by all 20 amino acids (excluding the amino acid present at the given position in wild type helix). So for a helix of length 5 amino acids this method will generate (5*X*19) 95 mutated sequences. The total number of mutated helices generated from 11592 helices is 2662375 which constitute the database of mutated sequences.

Proteins from nine SCOP (Structural Classification of Proteins) [46] classes viz. (I)All alpha proteins, (II)All beta proteins, (III)Alpha and beta proteins(a+b), (IV)Alpha and beta proteins(a/b), (V)Coiled coiled proteins, (VI)Membrane and cell surface proteins and peptides, (VII)Multi-domain proteins(alpha and beta), (VIII)Peptides and (IX)Small proteins were compiled to obtain sequences identical to the mutated sequences. A structural cut-off resolution ≤ 3Å and *R*≤0.3 were applied on these proteins with the PISCES server [47]. The resultant SCOP database consists of 48244 protein chains from 22309 protein structures.

Both the non-redundant protein and SCOP database used in the present study are similar to our recent work on ambivalent helices [34] which helps in comparison between both the works.

### Identical sequence search

The method for identifying the sequences identical to the mutated sequences in SCOP database in non-helical conformations is similar to previous method of searching variable and conserved helices [34]. However, a slight modification of the method was performed due to the large number of mutation generated sequences as compared to the original wild type helices. The method is outlined as follows: for a mutated sequence of *N* residues to be mapped onto a protein in the SCOP database of *M* residues, an *NXM* matrix is created where an element of the matrix, *A*(*i*,*j*)[*i*=1→*N*,*j*=1→*M*], is equal to 1 if *i*^*th*^ position of the helix and *j*^*th*^ position of the protein chain have identical residues and the conformation of the residue in *j*^*th*^ position of the protein in SCOP database is non-helical. Now if an element *A*(*k*,*l*)[*kεi*,*lεj*] = 1 and $\overset{N-1}{\underset{m=0}{\sum }}A(k+m,l+m)=N$, where *m* is a running index, then the helix from non-redundant database is mapped from *l* to *l*+*N*−1 position of the SCOP database protein with non-helical conformations. Sequences identical to the mutated sequences with non-helical conformations are only identified in the present study. This method yields sequences identical to the mutated sequences with completely non-helical conformations only. Among 2662375 mutated sequences, 28957 were mapped in 253728 sequences in the SCOP database with completely non-helical conformations. The list of 28957 mutation generated helical sequences are provided in Additional file 1: Table S1 of supplementary material. These 28957 mutated sequences are generated from 1775 non-redundant helices through point mutations. These 1775 helices are denoted as wild type helices, 28957 mutated sequences as ambivalent mutated sequences and 253728 mapped sequences in the SCOP database as non-helical sequences respectively.

## Results and discussion

### Number distribution of mutation generated ambivalent helices

**Figure 1:**
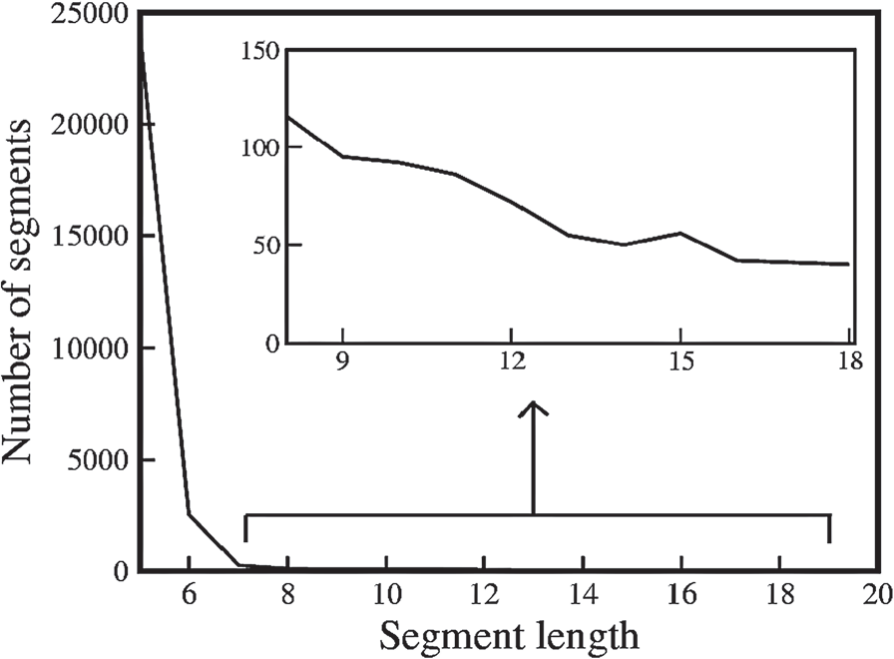
**Occurrence frequency vs. mutated sequence length.** Frequency of occurrence of mutation generated sequences conforming into non-helical structure in the SCOP database (28957 in number) as a function of sequence length. Inset depicts a magnified view of sequence length between 8−20.

The number of ambivalent mutated sequences (28957) which conform to non-helical structures in the SCOP database is plotted as a function of sequence length in Figure 1. From Figure 1 it is observed that the shorter mutated helical sequences (≤6) are more probable to conform to non-helical conformations in the SCOP database. However, a small number of sequences (∼6*%*) of length ≥7 are found to conform to non-helical conformations in the SCOP database. This further dips to ∼2*%* when mapped 253728 non-helical sequences in SCOP are considered. For long helices, hydrogen bonds render stability to the overall structure which is not disrupted by the point mutations. This explains why few long helices generated by point mutations switch to non-helical conformations.

The inset of Figure 1 highlights the region of sequence length ≥7. An interesting phenomenon may be observed here. The number of mutated sequences with length in multiples of 3 (i.e. 9, 12,...) either shows a peak or a trough, which is similar to the trend observed in helices [48]. A larger number of these sequences imply a greater probability of switching into non-helical conformations subject to point mutations.

### Propensity of conformation change with different mutations

Table 1 depicts the number of times an ambivalent mutated sequence is mapped into non-helical conformations in the SCOP database when a particular amino acid (first column) is mutated by 19 other amino acids (first row). A glance at the table shows that the number of non-helical conformations increases either by mutating an amino acid with helix breaking residues (e.g. columns starting with G, P) or substituting for a helix forming amino acid (e.g. row starting with L).

**Table 1:** **Number of times a helical sequence gets mapped into non-helical conformation in SCOP database when a particular amino acid (first column) is mutated by 19** other amino acids (first row)

**Non-helix→**	**A**	**C**	**D**	**E**	**F**	**G**	**H**	**I**	**K**	**L**	**M**	**N**	**P**	**Q**	**R**	**S**	**T**	**V**	**W**	**Y**
**Helix↓**																				
A	0	67	210	192	139	260	95	197	186	252	69	171	208	115	169	194	195	245	55	129
C	24	0	25	17	16	31	5	21	25	32	12	24	22	14	19	25	22	23	9	15
D	125	33	0	107	92	148	62	107	114	139	39	91	121	62	95	124	130	143	34	65
E	182	70	167	0	151	230	71	167	158	218	54	130	187	99	145	180	175	225	49	106
F	87	21	83	79	0	123	40	77	90	100	29	69	77	41	80	77	73	107	23	58
G	53	19	66	43	42	0	34	55	53	71	17	46	64	47	63	63	53	80	13	43
H	39	15	43	48	28	56	0	35	37	48	18	33	40	20	29	33	29	50	10	29
I	80	17	87	80	66	112	37	0	91	92	28	75	102	51	77	88	79	100	24	60
K	111	42	122	94	87	129	53	113	0	123	35	92	101	67	80	113	102	136	23	70
L	222	63	214	220	164	283	119	211	196	0	70	197	250	135	199	237	231	267	58	135
M	31	6	29	27	24	45	18	34	30	25	0	26	35	23	26	34	30	33	5	15
N	66	29	61	59	43	79	29	69	48	62	21	0	66	35	46	66	62	66	19	36
P	71	16	61	60	45	81	27	57	59	69	21	41	0	37	66	56	61	83	21	40
Q	72	29	67	62	49	97	30	73	60	91	21	55	73	0	58	65	73	87	19	52
R	94	25	99	93	66	121	47	88	89	105	33	72	107	46	0	92	97	107	30	64
S	101	22	93	86	81	128	43	84	87	112	38	83	98	64	73	0	90	124	17	53
T	83	22	72	74	48	98	27	80	66	88	23	61	81	32	65	72	0	101	22	51
V	124	32	120	109	83	143	62	96	122	135	39	90	127	82	91	109	115	0	32	73
W	49	11	38	36	37	54	23	44	43	41	12	31	49	23	36	47	40	52	0	30
Y	76	26	89	69	52	109	38	74	67	85	32	60	81	46	63	87	87	102	20	0

Diagonal elements are zero as these mutations will lead to wild type helices.

It is computationally infeasible to generate sequences identical to the mutated helical sequences. However, it is possible to obtain the number of mutated sequences which retain their helical conformations in the SCOP database. To estimate the number of different type of mutations, which retains the helical conformations, the probability of occurence of 20 amino acids in conserved helices is used from our earlier study [34]. The number of X → Y mutations leading to the retention of helical sequences is obtained by multiplying the total number of mutations to generate mutated sequences in the database with the fraction of occurence of amino acids, *Y*, in the conserved helices. Table 2 depicts the estimated number of each type of amino acid mutations which preserve helical conformations. The results show that a large number of sequences mutated by helix forming residues like Ala, Val etc. retain their helical conformation when mapped into SCOP database.

**Table 2:** Estimated number of times a helical sequence gets mapped into another helical conformation in SCOP database when a particular amino acid (first column) is mutated by 19 other amino acids (first row)

**Non-helix→**	**A**	**C**	**D**	**E**	**F**	**G**	**H**	**I**	**K**	**L**	**M**	**N**	**P**	**Q**	**R**	**S**	**T**	**V**	**W**	**Y**
**Helix↓**																				
A	0	208	750	1293	641	487	339	929	1028	1759	388	506	280	784	899	703	661	951	238	507
C	191	0	86	149	74	56	39	107	118	203	44	58	32	90	103	81	76	109	27	58
D	773	97	0	602	298	226	158	433	479	820	181	235	130	365	419	327	308	443	111	236
E	1413	177	638	0	545	414	288	791	875	1497	330	430	238	667	765	598	562	809	203	431
F	651	81	294	507	0	190	133	364	403	690	152	198	110	307	352	275	259	373	93	198
G	482	60	217	375	186	0	98	270	298	511	112	147	81	228	261	204	192	276	69	147
H	333	41	150	259	128	97	0	186	206	353	78	101	56	157	180	141	132	191	47	101
I	996	125	450	776	384	292	203	0	617	1056	233	303	168	470	539	422	396	570	143	304
K	1086	136	490	846	419	318	222	608	0	1151	254	331	183	513	588	460	432	622	156	331
L	1863	234	841	1451	719	546	380	1043	1153	0	436	568	314	880	1009	789	742	1067	267	569
M	348	43	157	271	134	102	71	195	215	369	0	106	58	164	188	147	138	199	50	106
N	528	66	238	411	204	154	107	295	327	560	123	0	89	249	286	223	210	302	75	161
P	280	35	126	218	108	82	57	157	173	297	65	85	0	132	151	118	111	160	40	85
Q	791	99	357	616	305	232	161	443	490	839	185	241	133	0	428	335	315	453	113	241
R	937	117	423	730	362	274	191	524	580	993	219	285	158	443	0	397	373	537	134	286
S	719	90	325	560	277	211	147	402	445	762	168	219	121	340	389	0	286	412	103	219
T	658	82	297	512	254	193	134	368	407	697	154	200	111	311	356	278	0	377	94	201
V	981	123	443	764	379	287	200	549	607	1040	229	299	165	463	531	415	390	0	141	299
W	234	29	105	182	90	68	47	131	144	248	54	71	39	110	126	99	93	134	0	71
Y	552	69	249	430	213	161	112	309	341	584	129	168	93	260	299	233	219	316	79	0

Diagonal elements are zero as these mutations will lead to wild type helices.

For simplification we divide the 20 amino acids into three groups based on their helix forming propensity: F for helix forming amino acids which include A, E, F, H, L, M, Q, V and W, I for helix indifferent amino acids viz. C, D, I, K, R, S and T and B for helix breaking amino acids like G, N, P and Y [49,50]. This division is primarily based upon the statistical analysis values rather than experimental scales like thermodynamic stability [51]. The number of different types of mutation is reduced from 380(20*X*19) to 3*X*3=9 viz. F →F, F →I, F →B,...etc. The propensity of a particular mutation X →Y, which transforms the sequence from helical to non-helical conformation, is defined as

(1) $$\text{Propensity}=\frac{p}{q}$$

where *p* is the probability of occurrence of X →Y mutation leading to 28957 ambivalent mutated sequences, while *q* denotes the probability of the same mutation in the database of mutated sequences. A propensity value >1 denotes a preference for the specific mutation to conform to the non-helical structures while <1 implies that the mutation may not preferably yield the same.

**Figure 2:**
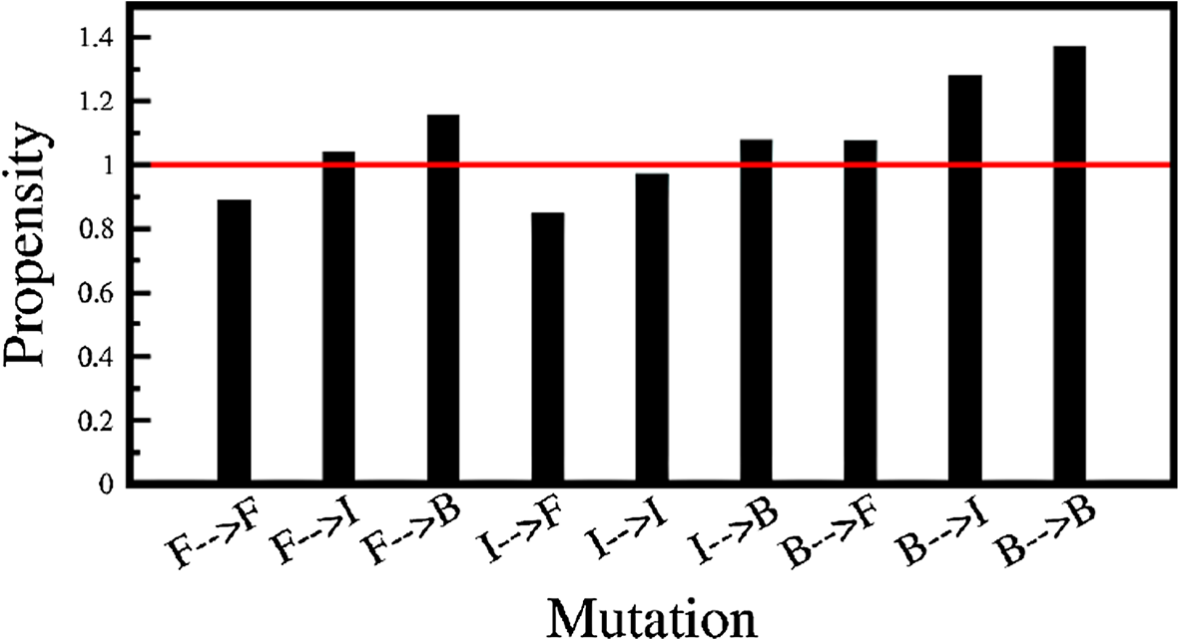
**Propensity of different mutations.** Propensity of 9 different types of mutations generating a helical conformation from the non-redundant database which map into non-helical conformation in the SCOP database. As depicted in Equation 1, propensity here is the ratio of probability of a given type of mutation leading to ambivalent mutated sequences to the probability of the same type of mutation in the total database of mutated sequences.

Figure 2 shows the propensity of a particular mutation leading to non-helical conformations with 9 different types of mutations. Propensity of mutations with helix-breaking residues have a higher probability to form non-helical structures in the SCOP database.

However, propensity of mutation of helix indifferent residues shows that it is not favored for conforming into non-helical structures.

Earlier results depict that amino acids have different preferences for the two termini of helices [48,52-57]. However, the present work did not find any perceptible difference in the propensities of different types of mutations at *N*- and *C*-terminus of the helical sequences.

Interestingly propensity for B →F, B →I and B →B are found to be greater than 1 in Figure 2. While higher propensities for B →B are expected, a similar trend for B →I and B →F may be explained by a marked increase in the solvent accessibility, which outweighs the decrease in propensity yielding predominantly non-helical conformations. Previous studies have shown that amino acid propensities are position specific with respect to both termini displaying an oscillatory behavior [48].

Moreover, helices are found to be ampiphilic in nature with respect to solvent accessibility and hydrophobicity [48,58-60].

Hence, mutation of helix breaking residues with helix-forming and helix-indifferent residues are position-specific, which may account for the behavior observed in Figure 2.

### Mutation generated sequences leading to non-helical conformations show change in solvent accessibility of the mutated site

The solvent accessibility of each residue is calculated using DSSP [42]. The normalized solvent accessibility is calculated as the ratio of this absolute value to the maximum solvent accessibility of amino acid residues found in Gly-X-Gly [61]. In this study, the residues are classified as buried or exposed according to their different degree of solvent accessibility. Buried residues are ≤7*%* solvent accessible, while the exposed surface residues have ≥37*%* relative solvent accessibility. Residues in the intermediate zone have relative solvent accessibility in the range between 7% and 37% [62].

**Figure 3:**
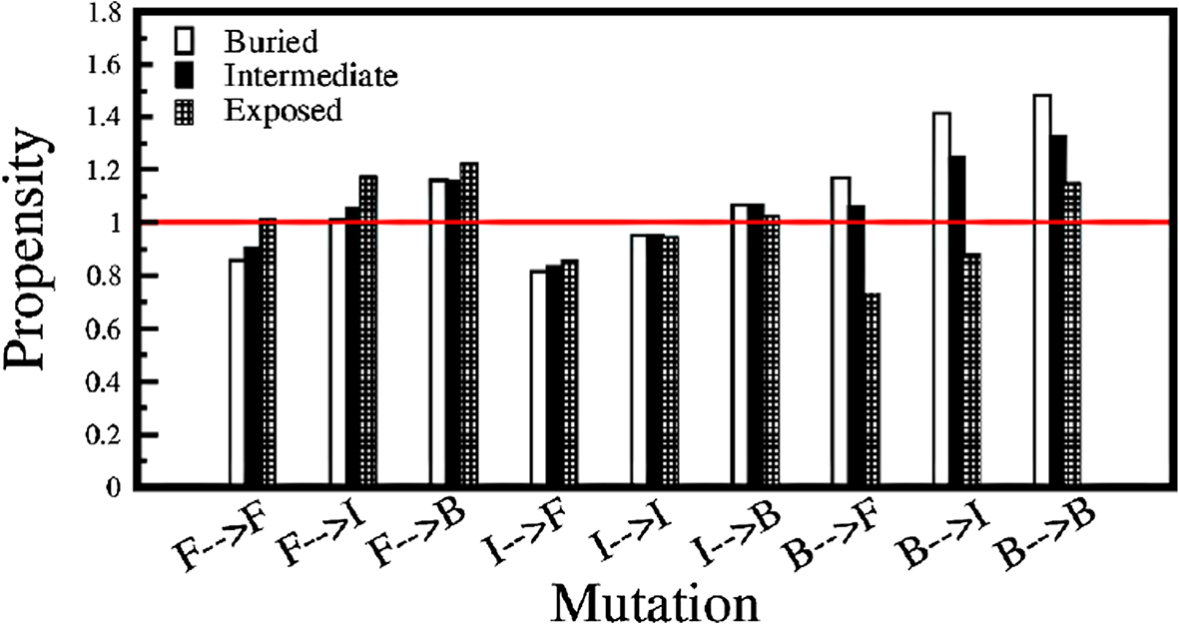
**Propensity of mutated Residue with different solvent accessibility.** Propensity of 9 different types of mutations at different solvent accessible positions (buried, intermediate and exposed) generating a helical conformation from the non-redundant database which map into non-helical conformation in the SCOP database. The propensity here depicts the ratio of probability of a given type of mutation at a given solvent accessibility leading to ambivalent mutated sequence to the probability of the same type of mutation at the similar solvent accessibility in the total database of mutated sequences.

Propensity of a X →Y mutation for different solvent accessible (buried/ intermediate/ exposed) residues of type X in the wild type helices is defined by equation 1. For a given solvent accessible residue of type X, *p* is the probability of occurrence of X →Y mutation in the ambivalent mutated sequences, while *q* is the probability of the same mutation for a residue of the same solvent accessibility in database of mutated sequences. Figure 3 depicts the propensity of a given type of mutation for residues of different solvent accessibility in the helices of the non-redundant database, corresponding to non-helical conformations in the SCOP database. The results show that mutations of helix-forming and helix-indifferent residues at solvent accessible sites mostly yields non-helical conformations followed by mutation of residues having intermediate solvent accessibility, while an opposite trend is observed for mutations of helix-breaking residues.

**Figure 4:**
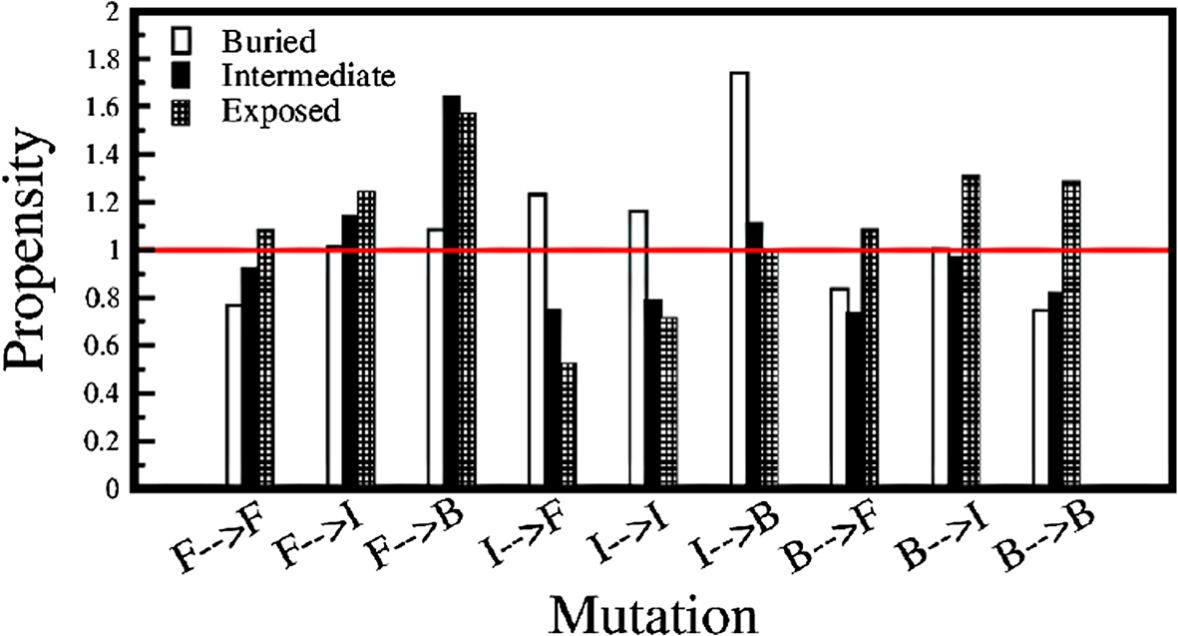
**Propensity of resultant residue with different solvent accessibility.** Propensity of 9 different types of mutations resulting in different solvent accessible (buried, intermediate and exposed) residues generating a helical conformation from the non-redundant database which map into non-helical conformation in the SCOP database. The propensity depicted here is similar to that shown in Figure 3. However, while calculating this propensity solvent accessibility of the resultant mutant residue is considered rather than solvent accessibility of the mutated residue.

Mapping the entire dataset of all mutation generated sequences (2662375) in the SCOP database demands enormous computational time and resources and hence is out of the scope of the present work. Hence the propensity of mutation X →Y for residues of different solvent accessibility (buried/ intermediate/ exposed) of Y in 253728 non-helical sequences of the SCOP database may be approximately calculated as the ratio of probabilities given by

(2) $$\text{Propensity}=\frac{p}{q_{1}\times q_{2}}$$

where *p* is the probability of X →Y mutation in the non-helical sequences (253728) with a particular type of solvent accessibility of Y and *q*_1_ and *q*_2_ are the probabilities of residue X and Y of same/different solvent accessibility in the non-redundant database and the SCOP database respectively. The denominator is normalized to unity for the different types of mutations. Figure 4 show the propensity of various types of mutations leading to different solvent accessibility of the mutated position. Excluding mutation of the helix forming residues (F) a completely opposite trend is observed in Figure 4 compared to that of Figure 3. This shows that mutating the helix-breaking and helix-indifferent residues the solvent accessibility changes, which makes the sequence non-helical.

**Figure 5:**
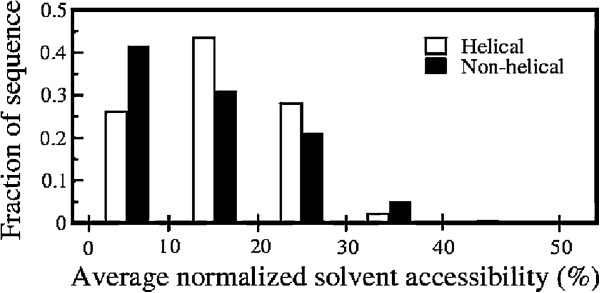
**Fraction of sequences vs. average solvent accessibility.** Fraction of helices and their corresponding mutation generated sequences in non-helical conformations with different average normalized solvent accessibility. The solvent accessibility of each residue was calculated using DSSP and normalized as depicted in the text.

The above results motivated us to investigate the overall solvent accessibility of the wild type helices, yielding the ambivalent mutated sequences which corresponds to the non-helical sequences in the SCOP database. Figure 5 shows the fraction of wild type helices and their corresponding non-helical sequences in the SCOP database at different values of the average normalized solvent accessibility. The results show that mutated sequences in the non-helical conformations have lower solvent accessibility i.e. they are buried in the interior of the protein compared to the parent helices. The data displayed in Figure 5 are validated through the t-test and the difference is found to be highly significant with P-value *P*<0.001 and T-value *T*=25.3569. For more than 65% cases, the non-helical conformations are found to be less solvent accessible as compared to the original helical conformations.

### Flanking sequences have vast differences in solvent accessibility though amino acid distributions are similar in them

**Figure 6:**
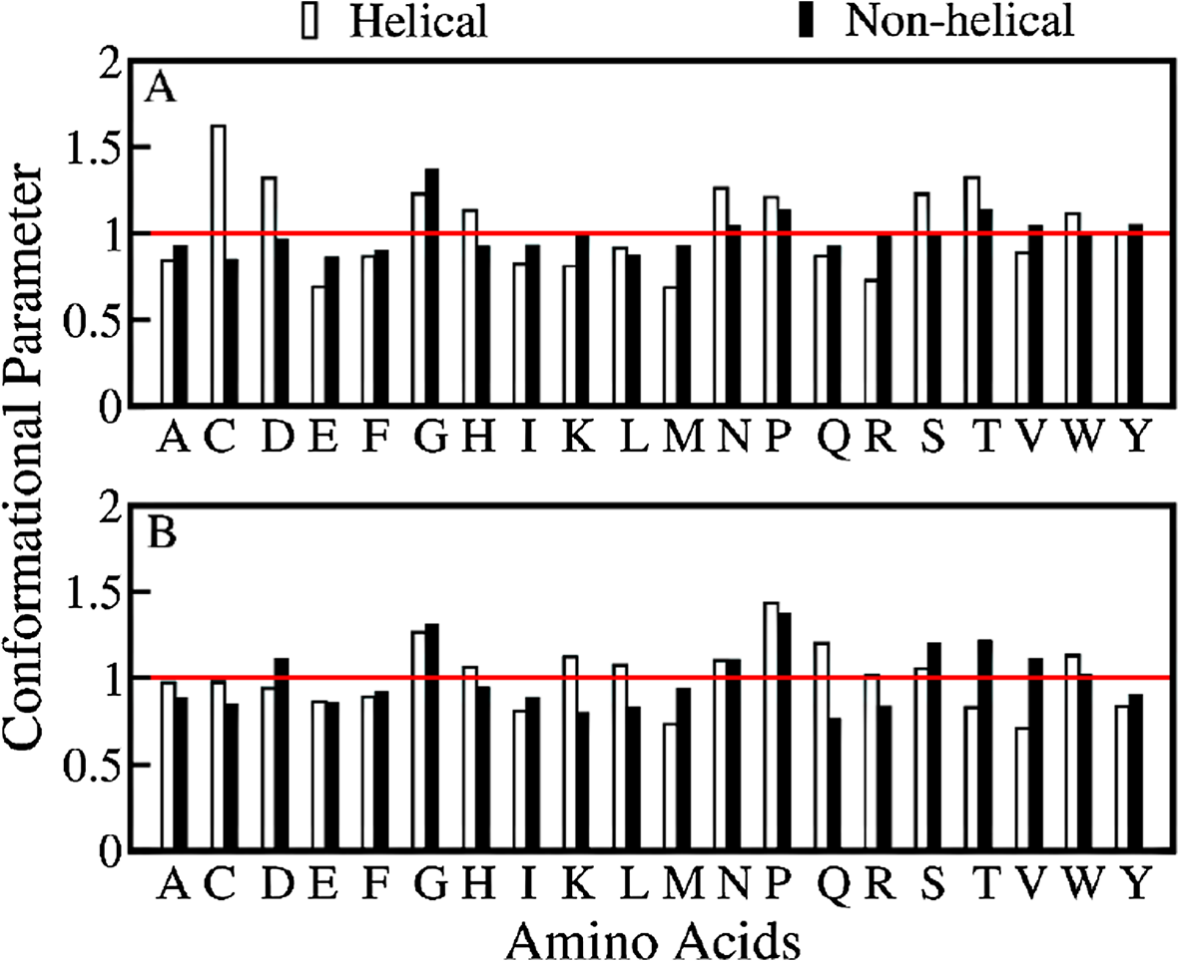
**Conformational parameter of amino acids in flanking sequences.** Conformational parameter of the amino acids in flanking sequences of (**A**) N-terminus and (**B**) C-terminus of helices and their mutated sequences in the non-helical conformations. Conformational parameter here refers to the ration of probability of an amino acid in the flanking sequences to probability of the same amino acid in non-redundant database.

Previous studies have established that context plays a major role in determining the structure of an ambivalent sequences [28,30,34]. It was suggested earlier that the flanking amino acids of ambivalent sequences play an important role in determining the conformations of these sequences. This finding is also validated experimentally [63]. An analysis of the amino acid occurrence frequency in the flanking sequences of the wild type helices and those of the corresponding non-helical conformations are also presented in this work. The flanking sequences are differentiated as N-terminus flanking sequences (four residues preceding the sequences) and C-terminus flanking sequences (four residues succeeding the sequences). Figure 6 shows the distribution of 20 amino acids in these flanking sequences. The distribution is depicted as the conformational parameter which is defined by,

(3) $$\text{CP}=\frac{P_{\text{ij}}}{P_{i}}$$

where *P*_*ij*_ is the probability of *i*^*th*^ residue in *j*^*th*^ flanking sequence while *P*_*i*_ is the probability of *i*^*th*^ residue in the non-redundant database.

Conformational parameter >1 depicts that the amino acid is preferred in that sequence while a value <1 shows it is not preferred.

Minimal differences are noticed for the flanking sequences of the wild type helices and their corresponding non-helical counterparts. This minimal differences in conformational parameters are in sharp contrast to those observed in chameleon/ambivalent sequences earlier [25,28,30,34,64]. In previous studies of chameleon/ambivalent sequences, significant differences in the amino acid conformational parameters were observed in sequences flanking helical and non-helical conformations. In the present study significant differences are found only for the amino acids C, D, H and S at the *N*-terminus and amino acids K, L, Q, T and V for the *C*-terminus flanking sequences. Since Cysteines tend to form disulfide bonds, their high occurrence in the flanking sequences of the wild type helices imparts extra stability to these structures.

**Figure 7:**
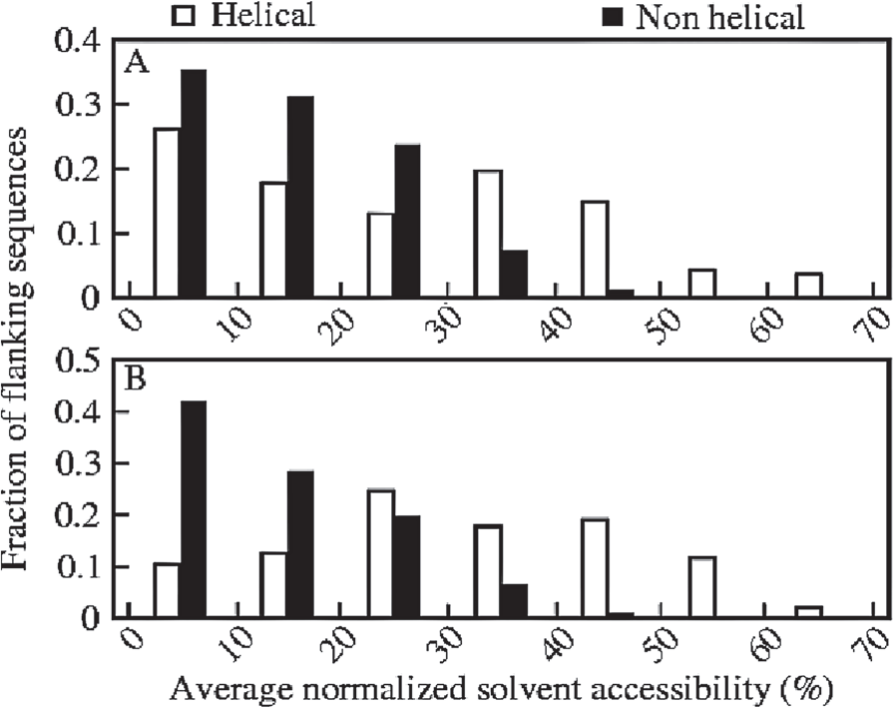
**Average solvent accessibility of flanking sequences.** Fraction of (**A**) N-terminus and (**B**) C-terminus flanking sequences of helices and their mutated sequences in the non-helical conformations with different average normalized solvent accessibility.

The solvent accessibility of the flanking sequences are also studied in the present work. Figure 7 shows the fraction of flanking sequences at different average normalized solvent accessibility. Despite close similarity in their amino acid preferences, these flanking sequences have vast differences in solvent accessibility. The sequences flanking the non-helical conformations at both N- and C-terminus are less solvent accessible compared to those flanking the wild type helices. The significance of the result is validated by the t-test, which gives a P-value of *P*<0.001 and T-value *T*=20.3459. Similar differences in the solvent accessibility of the flanking sequences were observed in earlier studies for chameleon/ambivalent sequences [25,28,30,34].

## Conclusions

Helices from the non-redundant database are point mutated to yield mutated sequences which are mapped in the SCOP database to obtain identical sequences with non-helical conformations. The results of this study confirm that small helices are more prone to non-helical conformations on point mutations. Mutations with helix breaking residues mostly transform the mutated sequence to non-helical conformations, while mutations of helix indifferent residues hardly yield non-helical conformations.

Solvent accessibility of mutating/mutated residues can be a primary factor for the helices to conform to non-helical conformations following point mutations. Most mutated residues are found to differ in the degree of solvent accessibility in helical and non-helical conformations. The non-helical conformations obtained after point mutations are found to be less solvent accessible as compared to the original helical conformations. Minimal differences appear in the amino acid distributions for the flanking sequences of helices in non-redundant database and their corresponding non-helical conformations in the SCOP database. However, the solvent accessibility of the flanking sequences of helices are vastly different from those of the respective non-helical conformations which are buried in the protein interior.

## Competing interests

Both authors declare that they have no competing interests.

## Authors’ contributions

NB and PB have designed research. NB has performed research. PB and NB have analyzed data, written the manuscript and approved the final version. Both authors read and approved the final manuscript.

## Supplementary Material

Additional file 1 **Table S1.** List of source for 28957 sequences. The file contains a table listing the source of all 28957 mutation generated helical sequences which conform into non-helical structures in SCOP database. Click here for file
