# Supplementary material for: Helical ambivalency induced by point mutations
Source: BMC Struct Biol. 2013 May 15;13:9. doi: 10.1186/1472-6807-13-9 (PMC3683331; doi:10.1186/1472-6807-13-9)
Supplement: Additional file 1 — Table S1. List of source for 28957 sequences. The file contains a table listing the source of all 28957 mutation generated helical sequences which conform into non-helical structures in SCOP database. [file 1472-6807-13-9-S1.pdf]

Supplementary Table T1. List of helices with the type of mutation performed to obtained helical ambivalency.

PDB ID With Chain	Helix between residue	Mutation at residue	Type of mutation
1RXTD	226 - 231	226	P-->E
1RXTD	226 - 231	229	L-->E
1RXTD	226 - 231	230	I-->R
1RXTD	245 - 249	245	R-->Q
1RXTD	245 - 249	245	R-->T
1RXTD	245 - 249	245	R-->W
1RXTD	245 - 249	246	T-->E
1RXTD	245 - 249	246	T-->N
1RXTD	245 - 249	246	T-->P
1RXTD	245 - 249	246	T-->Y
1RXTD	245 - 249	247	M-->A
1RXTD	245 - 249	247	M-->G
1RXTD	245 - 249	247	M-->H
1RXTD	245 - 249	247	M-->I
1RXTD	245 - 249	247	M-->L
1RXTD	245 - 249	247	M-->P
1RXTD	245 - 249	247	M-->Q
1RXTD	245 - 249	247	M-->S
1RXTD	245 - 249	247	M-->V
1RXTD	245 - 249	247	M-->W
1RXTD	245 - 249	247	M-->Y
1RXTD	245 - 249	248	K-->G
1RXTD	245 - 249	248	K-->Q

1RXTD	245 - 249	249	L-->D
1RXTD	245 - 249	249	L-->E
1RXTD	245 - 249	249	L-->H
1GQWB	131 - 136	132	I-->K
1GQWB	131 - 136	134	A-->F
2EREA	38 - 43	40	C-->Q
2D7SA	38 - 42	38	L-->A
2D7SA	38 - 42	38	L-->E
2D7SA	38 - 42	38	L-->F
2D7SA	38 - 42	38	L-->G
2D7SA	38 - 42	38	L-->I
2D7SA	38 - 42	38	L-->K
2D7SA	38 - 42	38	L-->M
2D7SA	38 - 42	38	L-->N
2D7SA	38 - 42	38	L-->Q
2D7SA	38 - 42	38	L-->V
2D7SA	38 - 42	38	L-->Y
2D7SA	38 - 42	39	D-->E
2D7SA	38 - 42	39	D-->F
2D7SA	38 - 42	39	D-->G
2D7SA	38 - 42	39	D-->I
2D7SA	38 - 42	39	D-->K
2D7SA	38 - 42	39	D-->Q
2D7SA	38 - 42	39	D-->T
2D7SA	38 - 42	39	D-->V
2D7SA	38 - 42	40	E-->A
2D7SA	38 - 42	40	E-->G

2D7SA	38 - 42	40	E-->I
2D7SA	38 - 42	40	E-->K
2D7SA	38 - 42	40	E-->L
2D7SA	38 - 42	40	E-->N
2D7SA	38 - 42	40	E-->P
2D7SA	38 - 42	40	E-->R
2D7SA	38 - 42	40	E-->S
2D7SA	38 - 42	40	E-->V
2D7SA	38 - 42	40	E-->Y
2D7SA	38 - 42	41	V-->A
2D7SA	38 - 42	41	V-->D
2D7SA	38 - 42	41	V-->E
2D7SA	38 - 42	41	V-->F
2D7SA	38 - 42	41	V-->G
2D7SA	38 - 42	41	V-->K
2D7SA	38 - 42	41	V-->N
2D7SA	38 - 42	41	V-->P
2D7SA	38 - 42	41	V-->S
2D7SA	38 - 42	42	I-->D
2D7SA	38 - 42	42	I-->E
2D7SA	38 - 42	42	I-->F
2D7SA	38 - 42	42	I-->G
2D7SA	38 - 42	42	I-->K
2D7SA	38 - 42	42	I-->N
2D7SA	38 - 42	42	I-->Q
2D7SA	38 - 42	42	I-->R
2D7SA	38 - 42	42	I-->T

2D7SA	170	175	170	M-->V
2D7SA	354	358	354	K-->G
2D7SA	354	358	354	K-->V
2D7SA	354	358	354	K-->W
2D7SA	354	358	355	P-->A
2D7SA	354	358	355	P-->F
2D7SA	354	358	355	P-->I
2D7SA	354	358	355	P-->K
2D7SA	354	358	355	P-->V
2D7SA	354	358	355	P-->Y
2D7SA	354	358	356	H-->A
2D7SA	354	358	356	H-->D
2D7SA	354	358	356	H-->G
2D7SA	354	358	356	H-->I
2D7SA	354	358	356	H-->K
2D7SA	354	358	356	H-->L
2D7SA	354	358	357	F-->K
2D7SA	354	358	357	F-->R
2D7SA	354	358	357	F-->T
2D7SA	354	358	357	F-->V
2D7SA	354	358	358	K-->C
2D7SA	354	358	358	K-->Q
2D7SA	354	358	358	K-->T
2D7SA	468	472	468	G-->D
2D7SA	468	472	468	G-->E
2D7SA	468	472	468	G-->F
2D7SA	468	472	468	G-->H

2D7SA	468	472	468	G-->I
2D7SA	468	472	468	G-->K
2D7SA	468	472	468	G-->L
2D7SA	468	472	468	G-->N
2D7SA	468	472	468	G-->S
2D7SA	468	472	468	G-->V
2D7SA	468	472	469	D-->A
2D7SA	468	472	469	D-->C
2D7SA	468	472	469	D-->F
2D7SA	468	472	469	D-->G
2D7SA	468	472	469	D-->I
2D7SA	468	472	469	D-->K
2D7SA	468	472	469	D-->L
2D7SA	468	472	469	D-->M
2D7SA	468	472	469	D-->N
2D7SA	468	472	469	D-->P
2D7SA	468	472	469	D-->R
2D7SA	468	472	469	D-->S
2D7SA	468	472	469	D-->T
2D7SA	468	472	469	D-->V
2D7SA	468	472	470	A-->C
2D7SA	468	472	470	A-->F
2D7SA	468	472	470	A-->G
2D7SA	468	472	470	A-->K
2D7SA	468	472	470	A-->L
2D7SA	468	472	470	A-->N
2D7SA	468	472	470	A-->R

2D7SA	468	472	470	A-->S
2D7SA	468	472	470	A-->T
2D7SA	468	472	470	A-->V
2D7SA	468	472	470	A-->Y
2D7SA	468	472	471	A-->E
2D7SA	468	472	471	A-->H
2D7SA	468	472	471	A-->K
2D7SA	468	472	471	A-->L
2D7SA	468	472	471	A-->N
2D7SA	468	472	471	A-->P
2D7SA	468	472	471	A-->S
2D7SA	468	472	471	A-->T
2D7SA	468	472	472	A-->D
2D7SA	468	472	472	A-->G
2D7SA	468	472	472	A-->K
2D7SA	468	472	472	A-->L
2D7SA	468	472	472	A-->M
2D7SA	468	472	472	A-->N
2D7SA	468	472	472	A-->P
2D7SA	468	472	472	A-->Q
2D7SA	468	472	472	A-->R
2D7SA	468	472	472	A-->T
2D7SA	468	472	472	A-->V
2J0UB	150	154	150	I-->D
2J0UB	150	154	150	I-->F
2J0UB	150	154	150	I-->G
2J0UB	150	154	150	I-->H

2J0UB	150 - 154	150	I-->M
2J0UB	150 - 154	150	I-->P
2J0UB	150 - 154	150	I-->T
2J0UB	150 - 154	150	I-->W
2J0UB	150 - 154	151	R-->A
2J0UB	150 - 154	151	R-->D
2J0UB	150 - 154	151	R-->E
2J0UB	150 - 154	151	R-->F
2J0UB	150 - 154	151	R-->G
2J0UB	150 - 154	151	R-->L
2J0UB	150 - 154	151	R-->M
2J0UB	150 - 154	151	R-->P
2J0UB	150 - 154	151	R-->T
2J0UB	150 - 154	151	R-->V
2J0UB	150 - 154	151	R-->W
2J0UB	150 - 154	152	K-->A
2J0UB	150 - 154	152	K-->D
2J0UB	150 - 154	152	K-->G
2J0UB	150 - 154	152	K-->H
2J0UB	150 - 154	152	K-->I
2J0UB	150 - 154	152	K-->L
2J0UB	150 - 154	152	K-->N
2J0UB	150 - 154	152	K-->P
2J0UB	150 - 154	152	K-->R
2J0UB	150 - 154	152	K-->T
2J0UB	150 - 154	152	K-->V
2J0UB	150 - 154	152	K-->Y

2J0UB	150 - 154	153	L-->D	
2J0UB	150 - 154	153	L-->G	
2J0UB	150 - 154	153	L-->P	
2J0UB	150 - 154	153	L-->R	
2J0UB	150 - 154	153	L-->S	
2J0UB	150 - 154	153	L-->T	
2J0UB	150 - 154	153	L-->V	
2J0UB	150 - 154	154	D-->K	
2J0UB	150 - 154	154	D-->R	
2J0UB	150 - 154	154	D-->S	
2J0UB	150 - 154	154	D-->V	
2P0YA	77 - 81	77	L-->D	
2P0YA	77 - 81	77	L-->I	
2P0YA	77 - 81	77	L-->K	
2P0YA	77 - 81	77	L-->M	
2P0YA	77 - 81	77	L-->N	
2P0YA	77 - 81	77	L-->R	
2P0YA	77 - 81	77	L-->T	
2P0YA	77 - 81	77	L-->W	
2P0YA	77 - 81	78	Y-->A	
2P0YA	77 - 81	78	Y-->D	
2P0YA	77 - 81	78	Y-->E	
2P0YA	77 - 81	78	Y-->G	
2P0YA	77 - 81	78	Y-->I	
2P0YA	77 - 81	78	Y-->K	
2P0YA	77 - 81	78	Y-->M	
2P0YA	77 - 81	78	Y-->N	

2P0YA	77 - 81	78	Y-->P
2P0YA	77 - 81	78	Y-->R
2P0YA	77 - 81	78	Y-->T
2P0YA	77 - 81	78	Y-->V
2P0YA	77 - 81	79	K-->C
2P0YA	77 - 81	79	K-->D
2P0YA	77 - 81	79	K-->E
2P0YA	77 - 81	79	K-->G
2P0YA	77 - 81	79	K-->L
2P0YA	77 - 81	79	K-->N
2P0YA	77 - 81	79	K-->P
2P0YA	77 - 81	79	K-->S
2P0YA	77 - 81	79	K-->V
2P0YA	77 - 81	80	D-->P
2P0YA	77 - 81	80	D-->V
2P0YA	77 - 81	81	I-->D
2P0YA	77 - 81	81	I-->G
2P0YA	77 - 81	81	I-->M
2P0YA	77 - 81	81	I-->P
2P0YA	77 - 81	81	I-->R
2P0YA	77 - 81	81	I-->T
2P0YA	77 - 81	81	I-->V
2P0YA	77 - 81	81	I-->W
2P0YA	77 - 81	77	F-->A
2P0YA	77 - 81	77	F-->D
2P0YA	77 - 81	77	F-->E
2P0YA	77 - 81	77	F-->I

2P0YA	77 - 81	77	F-->K
2P0YA	77 - 81	77	F-->L
2P0YA	77 - 81	77	F-->N
2P0YA	77 - 81	77	F-->R
2P0YA	77 - 81	77	F-->V
2P0YA	77 - 81	78	R-->A
2P0YA	77 - 81	78	R-->D
2P0YA	77 - 81	78	R-->G
2P0YA	77 - 81	78	R-->I
2P0YA	77 - 81	78	R-->K
2P0YA	77 - 81	78	R-->M
2P0YA	77 - 81	78	R-->N
2P0YA	77 - 81	78	R-->P
2P0YA	77 - 81	78	R-->Q
2P0YA	77 - 81	78	R-->S
2P0YA	77 - 81	78	R-->T
2P0YA	77 - 81	78	R-->V
2P0YA	77 - 81	78	R-->Y
2P0YA	77 - 81	79	G-->E
2P0YA	77 - 81	79	G-->F
2P0YA	77 - 81	79	G-->I
2P0YA	77 - 81	79	G-->P
2P0YA	77 - 81	79	G-->V
2P0YA	77 - 81	79	G-->Y
2P0YA	77 - 81	80	L-->A
2P0YA	77 - 81	80	L-->D
2P0YA	77 - 81	80	L-->F

2P0YA	77 - 81	80	L-->R
2P0YA	77 - 81	80	L-->S
2P0YA	77 - 81	80	L-->V
2P0YA	77 - 81	81	R-->A
2P0YA	77 - 81	81	R-->E
2P0YA	77 - 81	81	R-->F
2P0YA	77 - 81	81	R-->G
2P0YA	77 - 81	81	R-->H
2P0YA	77 - 81	81	R-->M
2P0YA	77 - 81	81	R-->N
2P0YA	77 - 81	81	R-->S
2P0YA	324 - 328	324	N-->D
2P0YA	324 - 328	324	N-->I
2P0YA	324 - 328	324	N-->R
2P0YA	324 - 328	324	N-->T
2P0YA	324 - 328	324	N-->V
2P0YA	324 - 328	324	N-->Y
2P0YA	324 - 328	325	L-->A
2P0YA	324 - 328	325	L-->F
2P0YA	324 - 328	325	L-->G
2P0YA	324 - 328	325	L-->M
2P0YA	324 - 328	326	V-->A
2P0YA	324 - 328	326	V-->H
2P0YA	324 - 328	326	V-->I
2P0YA	324 - 328	326	V-->N
2P0YA	324 - 328	326	V-->Q
2P0YA	324 - 328	326	V-->S

2P0YA	324 - 328	326	V-->T
2P0YA	324 - 328	327	G-->A
2P0YA	324 - 328	327	G-->I
2P0YA	324 - 328	327	G-->Q
2P0YA	324 - 328	327	G-->S
2P0YA	324 - 328	328	H-->A
2P0YA	324 - 328	328	H-->D
2P0YA	324 - 328	328	H-->F
2P0YA	324 - 328	328	H-->G
2P0YA	324 - 328	328	H-->I
2P0YA	324 - 328	328	H-->K
2P0YA	324 - 328	328	H-->L
2P0YA	324 - 328	328	H-->P
2P0YA	324 - 328	328	H-->S
2P0YA	324 - 328	328	H-->T
2P0YA	324 - 328	328	H-->V
1WC4B	1021 - 1025	1021	F-->K
1WC4B	1021 - 1025	1022	T-->L
1WC4B	1021 - 1025	1022	T-->Q
1WC4B	1021 - 1025	1023	R-->D
1WC4B	1021 - 1025	1023	R-->N
1WC4B	1021 - 1025	1024	M-->G
1WC4B	1021 - 1025	1024	M-->I
1WC4B	1021 - 1025	1024	M-->N
1WC4B	1021 - 1025	1024	M-->P
1WC4B	1021 - 1025	1024	M-->Q
1WC4B	1021 - 1025	1024	M-->V

1WC4B	1021	- 1025	1024	M-->Y
2NQMA	1021	- 1025	1021	L-->A
2NQMA	1021	- 1025	1021	L-->E
2NQMA	1021	- 1025	1021	L-->G
2NQMA	1021	- 1025	1021	L-->I
2NQMA	1021	- 1025	1021	L-->K
2NQMA	1021	- 1025	1021	L-->N
2NQMA	1021	- 1025	1021	L-->R
2NQMA	1021	- 1025	1021	L-->S
2NQMA	1021	- 1025	1021	L-->T
2NQMA	1021	- 1025	1021	L-->V
2NQMA	1021	- 1025	1022	P-->A
2NQMA	1021	- 1025	1022	P-->C
2NQMA	1021	- 1025	1022	P-->D
2NQMA	1021	- 1025	1022	P-->G
2NQMA	1021	- 1025	1022	P-->I
2NQMA	1021	- 1025	1022	P-->K
2NQMA	1021	- 1025	1022	P-->L
2NQMA	1021	- 1025	1022	P-->M
2NQMA	1021	- 1025	1022	P-->R
2NQMA	1021	- 1025	1022	P-->S
2NQMA	1021	- 1025	1022	P-->T
2NQMA	1021	- 1025	1022	P-->V
2NQMA	1021	- 1025	1022	P-->Y
2NQMA	1021	- 1025	1023	V-->A
2NQMA	1021	- 1025	1023	V-->D
2NQMA	1021	- 1025	1023	V-->E

2NQMA	1021 - 1025	1023	V-->I
2NQMA	1021 - 1025	1023	V-->L
2NQMA	1021 - 1025	1023	V-->P
2NQMA	1021 - 1025	1024	I-->A
2NQMA	1021 - 1025	1024	I-->C
2NQMA	1021 - 1025	1024	I-->D
2NQMA	1021 - 1025	1024	I-->E
2NQMA	1021 - 1025	1024	I-->F
2NQMA	1021 - 1025	1024	I-->G
2NQMA	1021 - 1025	1024	I-->H
2NQMA	1021 - 1025	1024	I-->K
2NQMA	1021 - 1025	1024	I-->L
2NQMA	1021 - 1025	1024	I-->P
2NQMA	1021 - 1025	1024	I-->S
2NQMA	1021 - 1025	1024	I-->T
2NQMA	1021 - 1025	1024	I-->V
2NQMA	1021 - 1025	1025	A-->D
2NQMA	1021 - 1025	1025	A-->E
2NQMA	1021 - 1025	1025	A-->F
2NQMA	1021 - 1025	1025	A-->G
2NQMA	1021 - 1025	1025	A-->I
2NQMA	1021 - 1025	1025	A-->K
2NQMA	1021 - 1025	1025	A-->L
2NQMA	1021 - 1025	1025	A-->M
2NQMA	1021 - 1025	1025	A-->N
2NQMA	1021 - 1025	1025	A-->P
2NQMA	1021 - 1025	1025	A-->R

2NQMA	1021 - 1025	1025	A-->S
2NQMA	1021 - 1025	1025	A-->T
2NQMA	1021 - 1025	1025	A-->V
1XDMX	67 - 71	68	S-->A
1XDMX	67 - 71	68	S-->F
1XDMX	67 - 71	68	S-->P
1XDMX	67 - 71	68	S-->T
1XDMX	67 - 71	68	S-->V
1XDMX	67 - 71	69	I-->D
1XDMX	67 - 71	69	I-->G
1XDMX	67 - 71	69	I-->N
1XDMX	67 - 71	69	I-->Q
1XDMX	67 - 71	69	I-->Y
1XDMX	67 - 71	70	N-->G
1XDMX	67 - 71	70	N-->L
1XDMX	67 - 71	70	N-->R
1XDMX	67 - 71	70	N-->V
1XDMX	67 - 71	70	N-->Y
1XDMX	67 - 71	71	Q-->A
1XDMX	67 - 71	71	Q-->D
1XDMX	67 - 71	71	Q-->F
1XDMX	67 - 71	71	Q-->I
1XDMX	67 - 71	71	Q-->K
1XDMX	67 - 71	71	Q-->L
1XDMX	67 - 71	71	Q-->N
1XDMX	67 - 71	71	Q-->V
1XDMX	67 - 71	71	Q-->Y

1JQ7A	67 - 71	67	P-->A
1JQ7A	67 - 71	67	P-->F
1JQ7A	67 - 71	67	P-->G
1JQ7A	67 - 71	67	P-->I
1JQ7A	67 - 71	67	P-->S
1JQ7A	67 - 71	67	P-->Y
1JQ7A	67 - 71	68	E-->C
1JQ7A	67 - 71	69	W-->A
1JQ7A	67 - 71	69	W-->C
1JQ7A	67 - 71	69	W-->F
1JQ7A	67 - 71	69	W-->G
1JQ7A	67 - 71	69	W-->I
1JQ7A	67 - 71	69	W-->K
1JQ7A	67 - 71	69	W-->M
1JQ7A	67 - 71	69	W-->N
1JQ7A	67 - 71	69	W-->P
1JQ7A	67 - 71	69	W-->Q
1JQ7A	67 - 71	69	W-->R
1JQ7A	67 - 71	69	W-->S
1JQ7A	67 - 71	69	W-->T
1JQ7A	67 - 71	69	W-->V
1JQ7A	67 - 71	70	V-->A
1JQ7A	67 - 71	70	V-->L
1JQ7A	67 - 71	70	V-->M
1JQ7A	67 - 71	70	V-->N
1JQ7A	67 - 71	71	T-->D
1JQ7A	67 - 71	71	T-->I

1JQ7A	67	71	71	T-->L
1JQ7A	67	71	71	T-->R
1JQ7A	67	71	71	T-->S
1JQ7A	67	71	71	T-->V
1JQ7A	1190	1195	1191	D-->G
1JQ7A	1190	1195	1191	D-->R
1JQ7A	1190	1195	1192	R-->E
1JQ7A	1190	1195	1192	R-->T
1JQ7A	1190	1195	1195	L-->I
1O3SA	10	15	11	L-->N
1O3SA	10	15	14	F-->T
2NZVG	129	134	131	H-->T
2G0BH	129	133	129	D-->A
2G0BH	129	133	129	D-->F
2G0BH	129	133	129	D-->K
2G0BH	129	133	129	D-->T
2G0BH	129	133	130	T-->L
2G0BH	129	133	130	T-->V
2G0BH	129	133	131	F-->A
2G0BH	129	133	131	F-->G
2G0BH	129	133	131	F-->K
2G0BH	129	133	131	F-->R
2G0BH	129	133	131	F-->S
2G0BH	129	133	131	F-->V
2G0BH	129	133	131	F-->Y
2G0BH	129	133	132	Y-->E
2G0BH	129	133	132	Y-->F

2G0BH	129 - 133	132	Y-->L
2G0BH	129 - 133	132	Y-->N
2G0BH	129 - 133	132	Y-->Q
2G0BH	129 - 133	132	Y-->S
2G0BH	129 - 133	133	S-->A
2G0BH	129 - 133	133	S-->D
2G0BH	129 - 133	133	S-->I
2G0BH	129 - 133	133	S-->K
2G0BH	129 - 133	133	S-->M
2G0BH	129 - 133	133	S-->N
2G0BH	129 - 133	133	S-->P
2G0BH	129 - 133	133	S-->V
1KPLA	320 - 324	320	I-->A
1KPLA	320 - 324	320	I-->G
1KPLA	320 - 324	320	I-->K
1KPLA	320 - 324	320	I-->L
1KPLA	320 - 324	320	I-->Q
1KPLA	320 - 324	320	I-->R
1KPLA	320 - 324	320	I-->S
1KPLA	320 - 324	320	I-->T
1KPLA	320 - 324	320	I-->V
1KPLA	320 - 324	321	P-->A
1KPLA	320 - 324	321	P-->E
1KPLA	320 - 324	321	P-->F
1KPLA	320 - 324	321	P-->G
1KPLA	320 - 324	321	P-->I
1KPLA	320 - 324	321	P-->K

1KPLA	320 - 324	321	P-->L
1KPLA	320 - 324	321	P-->R
1KPLA	320 - 324	321	P-->S
1KPLA	320 - 324	321	P-->T
1KPLA	320 - 324	321	P-->V
1KPLA	320 - 324	321	P-->Y
1KPLA	320 - 324	322	I-->A
1KPLA	320 - 324	322	I-->D
1KPLA	320 - 324	322	I-->E
1KPLA	320 - 324	322	I-->F
1KPLA	320 - 324	322	I-->H
1KPLA	320 - 324	322	I-->K
1KPLA	320 - 324	322	I-->L
1KPLA	320 - 324	322	I-->R
1KPLA	320 - 324	322	I-->S
1KPLA	320 - 324	322	I-->T
1KPLA	320 - 324	323	A-->E
1KPLA	320 - 324	323	A-->F
1KPLA	320 - 324	323	A-->G
1KPLA	320 - 324	323	A-->H
1KPLA	320 - 324	323	A-->I
1KPLA	320 - 324	323	A-->K
1KPLA	320 - 324	323	A-->L
1KPLA	320 - 324	323	A-->N
1KPLA	320 - 324	323	A-->P
1KPLA	320 - 324	323	A-->S
1KPLA	320 - 324	323	A-->T

1KPLA	320	324	323	A-->Y
1KPLA	320	324	324	A-->F
1KPLA	320	324	324	A-->G
1KPLA	320	324	324	A-->I
1KPLA	320	324	324	A-->N
1KPLA	320	324	324	A-->P
1KPLA	320	324	324	A-->Q
1KPLA	320	324	324	A-->S
1KPLA	320	324	324	A-->V
1KPLA	320	324	324	A-->W
1KPLA	320	324	324	A-->Y
1KPLA	396	401	397	L-->F
1KPLA	396	401	398	M-->K
1KPLA	396	401	398	M-->L
1R4LA	396	400	396	Y-->A
1R4LA	396	400	396	Y-->D
1R4LA	396	400	396	Y-->G
1R4LA	396	400	396	Y-->H
1R4LA	396	400	396	Y-->L
1R4LA	396	400	396	Y-->N
1R4LA	396	400	396	Y-->S
1R4LA	396	400	396	Y-->T
1R4LA	396	400	396	Y-->W
1R4LA	396	400	397	G-->F
1R4LA	396	400	397	G-->L
1R4LA	396	400	397	G-->R
1R4LA	396	400	397	G-->V

1R4LA	396	400	398	D-->C
1R4LA	396	400	398	D-->F
1R4LA	396	400	398	D-->P
1R4LA	396	400	399	Y-->K
1R4LA	396	400	400	W-->N
1R4LA	396	400	400	W-->Y
1R4LA	582	587	584	L-->E
1R4LA	582	587	587	Y-->S
1FZEF	208	212	208	W-->E
1FZEF	208	212	208	W-->G
1FZEF	208	212	208	W-->N
1FZEF	208	212	208	W-->S
1FZEF	208	212	210	Q-->C
1FZEF	208	212	211	Y-->E
1FZEF	208	212	211	Y-->T
1FZEF	208	212	208	C-->A
1FZEF	208	212	208	C-->D
1FZEF	208	212	208	C-->G
1FZEF	208	212	208	C-->I
1FZEF	208	212	208	C-->P
1FZEF	208	212	208	C-->T
1FZEF	208	212	209	A-->G
1FZEF	208	212	209	A-->P
1FZEF	208	212	209	A-->S
1FZEF	208	212	210	E-->K
1FZEF	208	212	211	Q-->M
1FZEF	208	212	211	Q-->N

1FZEF	208	212	211	Q-->S
1FZEF	208	212	211	Q-->T
2JGTA	140	145	140	G-->V
2JGTA	140	145	141	I-->Q
2JGTA	140	145	141	I-->R
2JGTA	140	145	142	I-->H
2JGTA	140	145	144	T-->N
2JGTA	140	145	145	L-->H
1KO6C	823	828	824	D-->G
1KO6C	823	828	825	R-->I
1KO6C	823	828	825	R-->T
1KO6C	823	828	827	A-->R
1KO6C	823	828	827	A-->V
1MR9C	20	24	20	I-->G
1MR9C	20	24	20	I-->K
1MR9C	20	24	20	I-->L
1MR9C	20	24	20	I-->P
1MR9C	20	24	20	I-->S
1MR9C	20	24	21	K-->C
1MR9C	20	24	21	K-->G
1MR9C	20	24	21	K-->I
1MR9C	20	24	21	K-->V
1MR9C	20	24	22	P-->A
1MR9C	20	24	22	P-->E
1MR9C	20	24	22	P-->F
1MR9C	20	24	22	P-->G
1MR9C	20	24	22	P-->I

1MR9C	20 - 24	22	P-->K
1MR9C	20 - 24	22	P-->L
1MR9C	20 - 24	22	P-->Q
1MR9C	20 - 24	22	P-->R
1MR9C	20 - 24	22	P-->S
1MR9C	20 - 24	22	P-->V
1MR9C	20 - 24	22	P-->Y
1MR9C	20 - 24	23	I-->A
1MR9C	20 - 24	23	I-->C
1MR9C	20 - 24	23	I-->E
1MR9C	20 - 24	23	I-->F
1MR9C	20 - 24	23	I-->K
1MR9C	20 - 24	23	I-->L
1MR9C	20 - 24	23	I-->N
1MR9C	20 - 24	23	I-->R
1MR9C	20 - 24	23	I-->T
1MR9C	20 - 24	23	I-->V
1MR9C	20 - 24	23	I-->Y
1MR9C	20 - 24	24	L-->C
1MR9C	20 - 24	24	L-->D
1MR9C	20 - 24	24	L-->G
1MR9C	20 - 24	24	L-->K
1MR9C	20 - 24	24	L-->M
1MR9C	20 - 24	24	L-->N
1MR9C	20 - 24	24	L-->P
1MR9C	20 - 24	24	L-->S
1MR9C	20 - 24	24	L-->V

1MR9C	20 - 24	24	L-->Y
1MR9C	193 - 197	193	I-->A
1MR9C	193 - 197	193	I-->E
1MR9C	193 - 197	193	I-->F
1MR9C	193 - 197	193	I-->K
1MR9C	193 - 197	193	I-->N
1MR9C	193 - 197	193	I-->Q
1MR9C	193 - 197	193	I-->T
1MR9C	193 - 197	194	D-->G
1MR9C	193 - 197	194	D-->I
1MR9C	193 - 197	194	D-->K
1MR9C	193 - 197	194	D-->M
1MR9C	193 - 197	194	D-->P
1MR9C	193 - 197	194	D-->S
1MR9C	193 - 197	195	K-->A
1MR9C	193 - 197	195	K-->G
1MR9C	193 - 197	195	K-->I
1MR9C	193 - 197	195	K-->T
1MR9C	193 - 197	195	K-->V
1MR9C	193 - 197	195	K-->W
1MR9C	193 - 197	196	I-->A
1MR9C	193 - 197	196	I-->E
1MR9C	193 - 197	196	I-->L
1MR9C	193 - 197	196	I-->N
1MR9C	193 - 197	196	I-->P
1MR9C	193 - 197	196	I-->Q
1MR9C	193 - 197	196	I-->R

1MR9C	193 - 197	196	I-->S	
1MR9C	193 - 197	196	I-->T	
1MR9C	193 - 197	196	I-->V	
1MR9C	193 - 197	196	I-->Y	
1MR9C	193 - 197	197	L-->A	
1MR9C	193 - 197	197	L-->C	
1MR9C	193 - 197	197	L-->D	
1MR9C	193 - 197	197	L-->E	
1MR9C	193 - 197	197	L-->I	
1MR9C	193 - 197	197	L-->N	
1MR9C	193 - 197	197	L-->P	
1MR9C	193 - 197	197	L-->R	
1MR9C	193 - 197	197	L-->S	
1MR9C	193 - 197	197	L-->V	
1MR9C	193 - 197	197	L-->W	
1X3WA	12 - 17	15	I-->P	
1T8IA	681 - 686	681	K-->P	
1T8IA	681 - 686	681	K-->V	
1T8IA	681 - 686	682	K-->A	
1T8IA	681 - 686	682	K-->Y	
1T8IA	681 - 686	683	V-->A	
1T8IA	681 - 686	685	E-->I	
1T8IA	681 - 686	686	S-->K	
1T8IA	681 - 686	686	S-->N	
1T8IA	681 - 686	686	S-->R	
1T8IA	681 - 686	686	S-->W	
1T8IA	717 - 722	718	T-->P	

1T8IA	717	722	718	T-->R
1T8IA	717	722	719	S-->D
1T8IA	717	722	719	S-->F
1T8IA	717	722	720	K-->T
1T8IA	717	722	722	N-->A
1T8IA	717	722	722	N-->D
1T8IA	717	722	722	N-->V
1T8IA	746	751	750	E-->G
1T8IA	753	757	753	A-->E
1T8IA	753	757	753	A-->S
1T8IA	753	757	754	W-->C
1T8IA	753	757	754	W-->F
1T8IA	753	757	754	W-->G
1T8IA	753	757	754	W-->I
1T8IA	753	757	754	W-->L
1T8IA	753	757	754	W-->P
1T8IA	753	757	754	W-->S
1T8IA	753	757	754	W-->T
1T8IA	753	757	754	W-->V
1T8IA	753	757	754	W-->Y
1T8IA	753	757	755	A-->D
1T8IA	753	757	755	A-->H
1T8IA	753	757	755	A-->K
1T8IA	753	757	755	A-->S
1T8IA	753	757	756	I-->K
1T8IA	753	757	756	I-->R
1T8IA	753	757	757	D-->Y

1IJDA	4 - 8	4	G-->P
1IJDA	4 - 8	4	G-->S
1IJDA	4 - 8	4	G-->Y
1IJDA	4 - 8	5	K-->A
1IJDA	4 - 8	5	K-->I
1IJDA	4 - 8	6	I-->E
1IJDA	4 - 8	6	I-->G
1IJDA	4 - 8	6	I-->T
1IJDA	4 - 8	6	I-->V
1IJDA	4 - 8	6	I-->W
1IJDA	4 - 8	6	I-->Y
1IJDA	4 - 8	7	W-->A
1IJDA	4 - 8	7	W-->C
1IJDA	4 - 8	7	W-->D
1IJDA	4 - 8	7	W-->E
1IJDA	4 - 8	7	W-->F
1IJDA	4 - 8	7	W-->G
1IJDA	4 - 8	7	W-->I
1IJDA	4 - 8	7	W-->K
1IJDA	4 - 8	7	W-->L
1IJDA	4 - 8	7	W-->P
1IJDA	4 - 8	7	W-->Q
1IJDA	4 - 8	7	W-->R
1IJDA	4 - 8	7	W-->S
1IJDA	4 - 8	7	W-->T
1IJDA	4 - 8	7	W-->V
1IJDA	4 - 8	7	W-->Y

1IJDA	4 - 8	8	T-->A
1IJDA	4 - 8	8	T-->F
1IJDA	4 - 8	8	T-->I
1IJDA	4 - 8	8	T-->L
1IJDA	4 - 8	4	W-->A
1IJDA	4 - 8	4	W-->H
1IJDA	4 - 8	4	W-->T
1IJDA	4 - 8	5	Y-->G
1IJDA	4 - 8	6	A-->P
1IJDA	4 - 8	7	A-->D
1IJDA	4 - 8	7	A-->I
1BU2A	24 - 29	24	L-->M
1BU2A	24 - 29	26	N-->G
1BU2A	24 - 29	27	L-->N
1BU2A	24 - 29	28	K-->G
1BU2A	24 - 29	29	L-->Q
1BU2A	24 - 29	29	L-->T
1BU2A	116 - 121	117	S-->D
1BU2A	116 - 121	117	S-->T
1BU2A	116 - 121	118	K-->V
1BU2A	116 - 121	120	T-->L
2AN6A	99 - 103	99	K-->F
2AN6A	99 - 103	99	K-->G
2AN6A	99 - 103	99	K-->L
2AN6A	99 - 103	99	K-->Q
2AN6A	99 - 103	99	K-->Y
2AN6A	99 - 103	100	Y-->A

2AN6A	99 -	103	100	Y-->E
2AN6A	99 -	103	100	Y-->F
2AN6A	99 -	103	100	Y-->G
2AN6A	99 -	103	100	Y-->I
2AN6A	99 -	103	100	Y-->K
2AN6A	99 -	103	100	Y-->M
2AN6A	99 -	103	100	Y-->P
2AN6A	99 -	103	100	Y-->Q
2AN6A	99 -	103	100	Y-->S
2AN6A	99 -	103	100	Y-->V
2AN6A	99 -	103	101	A-->H
2AN6A	99 -	103	101	A-->K
2AN6A	99 -	103	101	A-->M
2AN6A	99 -	103	101	A-->P
2AN6A	99 -	103	101	A-->T
2AN6A	99 -	103	101	A-->Y
2AN6A	99 -	103	102	S-->A
2AN6A	99 -	103	102	S-->G
2AN6A	99 -	103	102	S-->K
2AN6A	99 -	103	102	S-->L
2AN6A	99 -	103	102	S-->N
2AN6A	99 -	103	102	S-->P
2AN6A	99 -	103	102	S-->T
2AN6A	99 -	103	103	S-->E
2AN6A	99 -	103	103	S-->F
2AN6A	99 -	103	103	S-->N
2AN6A	99 -	103	103	S-->Q

2AN6A	99 - 103	103	S-->V
2AN6A	99 - 103	103	S-->Y
2B7LA	38 - 43	39	E-->K
2J69A	184 - 188	184	L-->D
2J69A	184 - 188	184	L-->F
2J69A	184 - 188	184	L-->R
2J69A	184 - 188	185	N-->A
2J69A	184 - 188	185	N-->D
2J69A	184 - 188	185	N-->L
2J69A	184 - 188	185	N-->P
2J69A	184 - 188	185	N-->T
2J69A	184 - 188	186	D-->F
2J69A	184 - 188	186	D-->G
2J69A	184 - 188	186	D-->I
2J69A	184 - 188	186	D-->L
2J69A	184 - 188	186	D-->Q
2J69A	184 - 188	186	D-->T
2J69A	184 - 188	186	D-->V
2J69A	184 - 188	187	T-->A
2J69A	184 - 188	187	T-->D
2J69A	184 - 188	187	T-->E
2J69A	184 - 188	187	T-->K
2J69A	184 - 188	187	T-->N
2J69A	184 - 188	187	T-->V
2J69A	184 - 188	188	E-->A
2J69A	184 - 188	188	E-->D
2J69A	184 - 188	188	E-->H

2J69A	184 - 188	188	E-->I
2J69A	184 - 188	188	E-->K
2J69A	184 - 188	188	E-->R
2J69A	184 - 188	188	E-->V
2J69A	428 - 432	428	L-->A
2J69A	428 - 432	428	L-->E
2J69A	428 - 432	428	L-->F
2J69A	428 - 432	428	L-->G
2J69A	428 - 432	428	L-->H
2J69A	428 - 432	428	L-->M
2J69A	428 - 432	428	L-->R
2J69A	428 - 432	428	L-->S
2J69A	428 - 432	428	L-->T
2J69A	428 - 432	428	L-->Y
2J69A	428 - 432	429	F-->A
2J69A	428 - 432	429	F-->K
2J69A	428 - 432	429	F-->L
2J69A	428 - 432	429	F-->N
2J69A	428 - 432	429	F-->R
2J69A	428 - 432	429	F-->S
2J69A	428 - 432	430	D-->A
2J69A	428 - 432	430	D-->Q
2J69A	428 - 432	431	F-->E
2J69A	428 - 432	431	F-->G
2J69A	428 - 432	431	F-->K
2J69A	428 - 432	431	F-->L
2J69A	428 - 432	431	F-->Q

2J69A	428	432	431	F-->R
2J69A	428	432	431	F-->S
2J69A	428	432	431	F-->Y
2J69A	428	432	432	L-->A
2J69A	428	432	432	L-->D
2J69A	428	432	432	L-->K
2J69A	428	432	432	L-->N
2J69A	428	432	432	L-->P
2J69A	428	432	432	L-->S
2J69A	428	432	432	L-->T
2J69A	428	432	432	L-->W
1G21F	134	139	137	M-->P
1G21F	134	139	138	P-->K
1G21F	134	139	139	I-->V
1G21F	216	222	222	I-->C
1IU3C	14	18	14	D-->F
1IU3C	14	18	14	D-->H
1IU3C	14	18	14	D-->L
1IU3C	14	18	14	D-->S
1IU3C	14	18	15	E-->A
1IU3C	14	18	15	E-->D
1IU3C	14	18	15	E-->I
1IU3C	14	18	15	E-->K
1IU3C	14	18	15	E-->L
1IU3C	14	18	15	E-->Q
1IU3C	14	18	15	E-->R
1IU3C	14	18	15	E-->T

1IU3C	14 - 18	15	E-->V
1IU3C	14 - 18	16	Y-->A
1IU3C	14 - 18	16	Y-->H
1IU3C	14 - 18	16	Y-->I
1IU3C	14 - 18	16	Y-->P
1IU3C	14 - 18	16	Y-->Q
1IU3C	14 - 18	16	Y-->S
1IU3C	14 - 18	16	Y-->V
1IU3C	14 - 18	17	A-->E
1IU3C	14 - 18	17	A-->N
1IU3C	14 - 18	18	E-->C
1IU3C	14 - 18	18	E-->G
1IU3C	14 - 18	18	E-->L
1IU3C	14 - 18	18	E-->N
1IU3C	14 - 18	18	E-->R
1IU3C	60 - 65	60	E-->D
1IU3C	60 - 65	62	T-->G
1IU3C	60 - 65	62	T-->K
1IU3C	60 - 65	64	L-->V
1IU3C	60 - 65	65	K-->P
1IU3C	60 - 65	65	K-->Q
1HUXA	149 - 153	149	L-->D
1HUXA	149 - 153	149	L-->E
1HUXA	149 - 153	149	L-->G
1HUXA	149 - 153	149	L-->H
1HUXA	149 - 153	149	L-->I
1HUXA	149 - 153	149	L-->K

1HUXA	149 - 153	149	L-->M
1HUXA	149 - 153	149	L-->N
1HUXA	149 - 153	149	L-->S
1HUXA	149 - 153	149	L-->T
1HUXA	149 - 153	149	L-->V
1HUXA	149 - 153	149	L-->Y
1HUXA	149 - 153	150	A-->D
1HUXA	149 - 153	150	A-->E
1HUXA	149 - 153	150	A-->F
1HUXA	149 - 153	150	A-->I
1HUXA	149 - 153	150	A-->K
1HUXA	149 - 153	150	A-->L
1HUXA	149 - 153	150	A-->Q
1HUXA	149 - 153	150	A-->R
1HUXA	149 - 153	150	A-->S
1HUXA	149 - 153	150	A-->T
1HUXA	149 - 153	150	A-->V
1HUXA	149 - 153	150	A-->Y
1HUXA	149 - 153	151	E-->A
1HUXA	149 - 153	151	E-->C
1HUXA	149 - 153	151	E-->D
1HUXA	149 - 153	151	E-->F
1HUXA	149 - 153	151	E-->G
1HUXA	149 - 153	151	E-->H
1HUXA	149 - 153	151	E-->I
1HUXA	149 - 153	151	E-->K
1HUXA	149 - 153	151	E-->L

1HUXA	149 - 153	151	E-->P
1HUXA	149 - 153	151	E-->Q
1HUXA	149 - 153	151	E-->S
1HUXA	149 - 153	151	E-->T
1HUXA	149 - 153	151	E-->V
1HUXA	149 - 153	151	E-->W
1HUXA	149 - 153	151	E-->Y
1HUXA	149 - 153	152	L-->A
1HUXA	149 - 153	152	L-->D
1HUXA	149 - 153	152	L-->E
1HUXA	149 - 153	152	L-->G
1HUXA	149 - 153	152	L-->I
1HUXA	149 - 153	152	L-->K
1HUXA	149 - 153	152	L-->P
1HUXA	149 - 153	152	L-->R
1HUXA	149 - 153	152	L-->S
1HUXA	149 - 153	152	L-->T
1HUXA	149 - 153	152	L-->V
1HUXA	149 - 153	153	G-->D
1HUXA	149 - 153	153	G-->L
1HUXA	149 - 153	153	G-->N
1HUXA	149 - 153	153	G-->P
1HUXA	149 - 153	153	G-->Q
1HUXA	149 - 153	153	G-->R
1HUXA	149 - 153	153	G-->S
1HUXA	149 - 153	153	G-->T
1QLEA	103 - 107	103	V-->A

1QLEA	103 - 107	103	V-->E
1QLEA	103 - 107	103	V-->G
1QLEA	103 - 107	103	V-->I
1QLEA	103 - 107	103	V-->K
1QLEA	103 - 107	103	V-->L
1QLEA	103 - 107	103	V-->P
1QLEA	103 - 107	103	V-->Q
1QLEA	103 - 107	103	V-->R
1QLEA	103 - 107	103	V-->S
1QLEA	103 - 107	104	I-->A
1QLEA	103 - 107	104	I-->E
1QLEA	103 - 107	104	I-->G
1QLEA	103 - 107	104	I-->H
1QLEA	103 - 107	104	I-->L
1QLEA	103 - 107	104	I-->M
1QLEA	103 - 107	104	I-->N
1QLEA	103 - 107	104	I-->P
1QLEA	103 - 107	104	I-->Q
1QLEA	103 - 107	104	I-->Y
1QLEA	103 - 107	105	P-->D
1QLEA	103 - 107	105	P-->E
1QLEA	103 - 107	105	P-->F
1QLEA	103 - 107	105	P-->I
1QLEA	103 - 107	105	P-->K
1QLEA	103 - 107	105	P-->L
1QLEA	103 - 107	105	P-->R
1QLEA	103 - 107	105	P-->S

1QLEA	103 - 107	105	P-->T
1QLEA	103 - 107	105	P-->V
1QLEA	103 - 107	105	P-->Y
1QLEA	103 - 107	106	A-->D
1QLEA	103 - 107	106	A-->E
1QLEA	103 - 107	106	A-->G
1QLEA	103 - 107	106	A-->I
1QLEA	103 - 107	106	A-->K
1QLEA	103 - 107	106	A-->N
1QLEA	103 - 107	106	A-->P
1QLEA	103 - 107	106	A-->T
1QLEA	103 - 107	106	A-->V
1QLEA	103 - 107	106	A-->W
1QLEA	103 - 107	107	L-->A
1QLEA	103 - 107	107	L-->C
1QLEA	103 - 107	107	L-->D
1QLEA	103 - 107	107	L-->E
1QLEA	103 - 107	107	L-->G
1QLEA	103 - 107	107	L-->P
1QLEA	103 - 107	107	L-->R
1QLEA	103 - 107	107	L-->S
1QLEA	103 - 107	107	L-->Y
1QLEA	432 - 436	432	I-->D
1QLEA	432 - 436	432	I-->F
1QLEA	432 - 436	432	I-->K
1QLEA	432 - 436	432	I-->Q
1QLEA	432 - 436	432	I-->R

1QLEA	432	436	432	I-->T
1QLEA	432	436	433	G-->K
1QLEA	432	436	433	G-->T
1QLEA	432	436	434	K-->D
1QLEA	432	436	434	K-->E
1QLEA	432	436	434	K-->I
1QLEA	432	436	434	K-->S
1QLEA	432	436	435	M-->C
1QLEA	432	436	435	M-->D
1QLEA	432	436	435	M-->G
1QLEA	432	436	435	M-->H
1QLEA	432	436	435	M-->I
1QLEA	432	436	435	M-->N
1QLEA	432	436	435	M-->Q
1QLEA	432	436	435	M-->S
1QLEA	432	436	435	M-->T
1QLEA	432	436	435	M-->Y
1QLEA	432	436	436	S-->R
1QLEA	432	436	436	S-->V
1QLEA	432	436	436	S-->Y
1K8RB	155	160	155	F-->H
1K8RB	155	160	158	L-->A
1NHYA	109	113	109	I-->F
1NHYA	109	113	109	I-->G
1NHYA	109	113	109	I-->K
1NHYA	109	113	110	Q-->D
1NHYA	109	113	110	Q-->E

1NHYA	109 - 113	110	Q-->I
1NHYA	109 - 113	110	Q-->K
1NHYA	109 - 113	110	Q-->L
1NHYA	109 - 113	110	Q-->M
1NHYA	109 - 113	110	Q-->N
1NHYA	109 - 113	110	Q-->P
1NHYA	109 - 113	110	Q-->T
1NHYA	109 - 113	110	Q-->V
1NHYA	109 - 113	111	I-->E
1NHYA	109 - 113	111	I-->L
1NHYA	109 - 113	111	I-->P
1NHYA	109 - 113	111	I-->T
1NHYA	109 - 113	111	I-->Y
1NHYA	109 - 113	112	A-->L
1NHYA	109 - 113	112	A-->Q
1NHYA	109 - 113	112	A-->Y
1NHYA	109 - 113	113	N-->G
1NHYA	109 - 113	113	N-->L
1NHYA	109 - 113	113	N-->T
1NHYA	109 - 113	113	N-->V
1NHYA	179 - 184	179	T-->V
1NHYA	179 - 184	182	R-->A
1FQ1A	81 - 86	81	R-->N
1FQ1A	81 - 86	82	G-->A
1FQ1A	81 - 86	83	E-->Q
1FQ1A	81 - 86	86	K-->C
1FQ1A	92 - 97	92	L-->N

1FQ1A	92 - 97	92	L-->Y
1FQ1A	92 - 97	93	L-->F
2AXTD	101 - 107	103	R-->I
2AXTD	101 - 105	101	I-->A
2AXTD	101 - 105	101	I-->E
2AXTD	101 - 105	101	I-->F
2AXTD	101 - 105	101	I-->G
2AXTD	101 - 105	101	I-->K
2AXTD	101 - 105	101	I-->L
2AXTD	101 - 105	101	I-->P
2AXTD	101 - 105	101	I-->S
2AXTD	101 - 105	101	I-->W
2AXTD	101 - 105	102	Y-->A
2AXTD	101 - 105	102	Y-->E
2AXTD	101 - 105	102	Y-->F
2AXTD	101 - 105	102	Y-->G
2AXTD	101 - 105	102	Y-->K
2AXTD	101 - 105	102	Y-->N
2AXTD	101 - 105	102	Y-->Q
2AXTD	101 - 105	102	Y-->R
2AXTD	101 - 105	102	Y-->S
2AXTD	101 - 105	102	Y-->T
2AXTD	101 - 105	102	Y-->V
2AXTD	101 - 105	103	P-->D
2AXTD	101 - 105	103	P-->G
2AXTD	101 - 105	103	P-->M
2AXTD	101 - 105	103	P-->N

2AXTD	101 - 105	103	P-->R
2AXTD	101 - 105	103	P-->S
2AXTD	101 - 105	103	P-->T
2AXTD	101 - 105	103	P-->V
2AXTD	101 - 105	104	L-->A
2AXTD	101 - 105	104	L-->D
2AXTD	101 - 105	104	L-->E
2AXTD	101 - 105	104	L-->G
2AXTD	101 - 105	104	L-->H
2AXTD	101 - 105	104	L-->I
2AXTD	101 - 105	104	L-->N
2AXTD	101 - 105	104	L-->T
2AXTD	101 - 105	105	G-->D
2AXTD	101 - 105	105	G-->I
2AXTD	101 - 105	105	G-->S
2AXTD	101 - 105	105	G-->T
2AXTE	10 - 15	10	F-->D
2AXTE	10 - 15	12	D-->A
2AXTE	10 - 15	13	I-->K
2AXTE	10 - 15	14	I-->F
2AXTE	10 - 15	14	I-->L
2AXTE	42 - 46	42	L-->D
2AXTE	42 - 46	42	L-->E
2AXTE	42 - 46	42	L-->G
2AXTE	42 - 46	42	L-->K
2AXTE	42 - 46	42	L-->S
2AXTE	42 - 46	42	L-->T

2AXTE	42 - 46	42	L-->V
2AXTE	42 - 46	43	A-->E
2AXTE	42 - 46	43	A-->F
2AXTE	42 - 46	43	A-->G
2AXTE	42 - 46	43	A-->I
2AXTE	42 - 46	43	A-->K
2AXTE	42 - 46	43	A-->N
2AXTE	42 - 46	43	A-->V
2AXTE	42 - 46	43	A-->Y
2AXTE	42 - 46	44	Y-->A
2AXTE	42 - 46	44	Y-->D
2AXTE	42 - 46	44	Y-->E
2AXTE	42 - 46	44	Y-->F
2AXTE	42 - 46	44	Y-->G
2AXTE	42 - 46	44	Y-->I
2AXTE	42 - 46	44	Y-->L
2AXTE	42 - 46	44	Y-->M
2AXTE	42 - 46	44	Y-->N
2AXTE	42 - 46	44	Y-->P
2AXTE	42 - 46	44	Y-->S
2AXTE	42 - 46	44	Y-->T
2AXTE	42 - 46	44	Y-->V
2AXTE	42 - 46	44	Y-->W
2AXTE	42 - 46	45	D-->E
2AXTE	42 - 46	45	D-->K
2AXTE	42 - 46	45	D-->P
2AXTE	42 - 46	45	D-->T

2AXTE	42 - 46	45	D-->Y
2AXTE	42 - 46	46	V-->A
2AXTE	42 - 46	46	V-->E
2AXTE	42 - 46	46	V-->F
2AXTE	42 - 46	46	V-->G
2AXTE	42 - 46	46	V-->L
2AXTE	42 - 46	46	V-->P
2AXTE	42 - 46	46	V-->S
2AXTE	42 - 46	46	V-->W
2AXTH	6 - 11	6	W-->D
2AXTH	6 - 11	6	W-->F
2AXTH	6 - 11	6	W-->H
2AXTH	6 - 11	6	W-->Q
2AXTH	6 - 11	6	W-->R
2AXTH	6 - 11	9	D-->R
2AXTH	6 - 11	11	L-->I
2AXTH	6 - 11	11	L-->V
2AXTK	13 - 17	13	E-->F
2AXTK	13 - 17	13	E-->G
2AXTK	13 - 17	13	E-->H
2AXTK	13 - 17	13	E-->I
2AXTK	13 - 17	13	E-->Q
2AXTK	13 - 17	14	A-->E
2AXTK	13 - 17	14	A-->I
2AXTK	13 - 17	14	A-->K
2AXTK	13 - 17	14	A-->P
2AXTK	13 - 17	14	A-->V

2AXTK	13 - 17	15	Y-->A
2AXTK	13 - 17	15	Y-->D
2AXTK	13 - 17	15	Y-->E
2AXTK	13 - 17	15	Y-->F
2AXTK	13 - 17	15	Y-->G
2AXTK	13 - 17	15	Y-->K
2AXTK	13 - 17	15	Y-->P
2AXTK	13 - 17	15	Y-->R
2AXTK	13 - 17	15	Y-->V
2AXTK	13 - 17	16	A-->L
2AXTK	13 - 17	16	A-->M
2AXTK	13 - 17	16	A-->Y
2AXTK	13 - 17	17	I-->E
2AXTK	13 - 17	17	I-->G
2AXTK	13 - 17	17	I-->V
2AXTK	13 - 17	17	I-->Y
2AXTU	42 - 46	42	V-->A
2AXTU	42 - 46	42	V-->D
2AXTU	42 - 46	42	V-->E
2AXTU	42 - 46	42	V-->F
2AXTU	42 - 46	42	V-->G
2AXTU	42 - 46	42	V-->H
2AXTU	42 - 46	42	V-->I
2AXTU	42 - 46	42	V-->K
2AXTU	42 - 46	42	V-->L
2AXTU	42 - 46	42	V-->P
2AXTU	42 - 46	42	V-->Q

2AXTU	42 - 46	42	V-->R
2AXTU	42 - 46	42	V-->T
2AXTU	42 - 46	43	V-->A
2AXTU	42 - 46	43	V-->D
2AXTU	42 - 46	43	V-->E
2AXTU	42 - 46	43	V-->K
2AXTU	42 - 46	43	V-->L
2AXTU	42 - 46	43	V-->M
2AXTU	42 - 46	43	V-->P
2AXTU	42 - 46	43	V-->S
2AXTU	42 - 46	43	V-->T
2AXTU	42 - 46	43	V-->Y
2AXTU	42 - 46	44	D-->A
2AXTU	42 - 46	44	D-->G
2AXTU	42 - 46	44	D-->H
2AXTU	42 - 46	44	D-->I
2AXTU	42 - 46	44	D-->K
2AXTU	42 - 46	44	D-->L
2AXTU	42 - 46	44	D-->M
2AXTU	42 - 46	44	D-->P
2AXTU	42 - 46	44	D-->S
2AXTU	42 - 46	44	D-->V
2AXTU	42 - 46	45	E-->A
2AXTU	42 - 46	45	E-->D
2AXTU	42 - 46	45	E-->G
2AXTU	42 - 46	45	E-->H
2AXTU	42 - 46	45	E-->I

2AXTU	42	- 46	45	E-->K
2AXTU	42	- 46	45	E-->L
2AXTU	42	- 46	45	E-->S
2AXTU	42	- 46	45	E-->V
2AXTU	42	- 46	46	K-->A
2AXTU	42	- 46	46	K-->D
2AXTU	42	- 46	46	K-->E
2AXTU	42	- 46	46	K-->H
2AXTU	42	- 46	46	K-->I
2AXTU	42	- 46	46	K-->N
2AXTU	42	- 46	46	K-->P
2AXTU	42	- 46	46	K-->T
2AXTU	42	- 46	46	K-->V
2AXTU	42	- 46	46	K-->Y
2AXTU	88	- 93	89	E-->D
2AXTU	88	- 93	90	D-->Q
2AXTU	88	- 93	91	V-->A
2AXTU	119	- 125	121	L-->D
2AXTU	119	- 125	123	E-->T
4HMGA	66	- 71	68	D-->K
4HMGA	66	- 71	68	D-->L
4HMGA	66	- 71	69	A-->N
4HMGA	74	- 79	74	P-->I
4HMGA	74	- 79	76	C-->S
1TI8B	159	- 163	159	H-->D
1TI8B	159	- 163	159	H-->E
1TI8B	159	- 163	159	H-->F

1TI8B	159	163	159	H-->S
1TI8B	159	163	159	H-->V
1TI8B	159	163	160	S-->G
1TI8B	159	163	160	S-->L
1TI8B	159	163	161	R-->V
1TI8B	159	163	162	Y-->A
1TI8B	159	163	162	Y-->K
1TI8B	159	163	162	Y-->T
1TI8B	159	163	163	R-->E
1TI8B	159	163	163	R-->K
1LKXD	159	163	159	E-->A
1LKXD	159	163	159	E-->S
1LKXD	159	163	159	E-->V
1LKXD	159	163	159	E-->W
1LKXD	159	163	159	E-->Y
1LKXD	159	163	160	Y-->D
1LKXD	159	163	160	Y-->E
1LKXD	159	163	160	Y-->F
1LKXD	159	163	160	Y-->I
1LKXD	159	163	160	Y-->K
1LKXD	159	163	160	Y-->L
1LKXD	159	163	160	Y-->N
1LKXD	159	163	160	Y-->V
1LKXD	159	163	161	L-->T
1LKXD	159	163	161	L-->V
1LKXD	159	163	162	K-->P
1LKXD	159	163	162	K-->V

1LKXD	159 - 163	162	K-->Y
1LKXD	159 - 163	163	K-->D
1LKXD	159 - 163	163	K-->E
1LKXD	159 - 163	163	K-->I
1LKXD	159 - 163	163	K-->L
1LKXD	159 - 163	163	K-->S
1LKXD	443 - 447	443	P-->G
1LKXD	443 - 447	443	P-->V
1LKXD	443 - 447	445	C-->E
1LKXD	443 - 447	445	C-->G
1LKXD	443 - 447	445	C-->L
1LKXD	443 - 447	445	C-->M
1LKXD	443 - 447	445	C-->R
1LKXD	443 - 447	446	E-->P
1LKXD	443 - 447	446	E-->T
1LKXD	443 - 447	447	L-->F
1LKXD	443 - 447	447	L-->Q
1LKXD	443 - 447	447	L-->S
1LKXD	443 - 447	447	L-->V
1LKXD	443 - 447	443	F-->A
1LKXD	443 - 447	445	D-->E
1LKXD	443 - 447	446	K-->E
1LKXD	443 - 447	447	N-->P
1LKXD	443 - 447	447	N-->W
1LKXD	541 - 546	541	P-->I
1LKXD	541 - 546	541	P-->V
1LKXD	541 - 546	542	L-->T

1LKXD	541 - 546	543	V-->I
1LKXD	541 - 546	544	Q-->I
1LKXD	541 - 546	544	Q-->N
1LKXD	541 - 546	544	Q-->V
2A7RB	541 - 545	541	T-->A
2A7RB	541 - 545	541	T-->G
2A7RB	541 - 545	541	T-->I
2A7RB	541 - 545	541	T-->K
2A7RB	541 - 545	541	T-->N
2A7RB	541 - 545	541	T-->P
2A7RB	541 - 545	541	T-->S
2A7RB	541 - 545	541	T-->W
2A7RB	541 - 545	542	R-->D
2A7RB	541 - 545	542	R-->G
2A7RB	541 - 545	542	R-->I
2A7RB	541 - 545	542	R-->K
2A7RB	541 - 545	542	R-->N
2A7RB	541 - 545	542	R-->S
2A7RB	541 - 545	542	R-->V
2A7RB	541 - 545	543	K-->D
2A7RB	541 - 545	543	K-->I
2A7RB	541 - 545	543	K-->R
2A7RB	541 - 545	543	K-->V
2A7RB	541 - 545	544	K-->F
2A7RB	541 - 545	544	K-->G
2A7RB	541 - 545	544	K-->I
2A7RB	541 - 545	544	K-->M

2A7RB	541	545	544	K-->P
2A7RB	541	545	544	K-->R
2A7RB	541	545	544	K-->T
2A7RB	541	545	544	K-->V
2A7RB	541	545	544	K-->W
2A7RB	541	545	544	K-->Y
2A7RB	541	545	545	T-->G
2A7RB	541	545	545	T-->I
2A7RB	541	545	545	T-->K
2A7RB	541	545	545	T-->N
2A7RB	541	545	545	T-->R
2A7RB	541	545	545	T-->V
2A7RB	324	329	324	L-->K
2A7RB	324	329	326	E-->N
2A7RB	324	329	327	L-->G
2A7RB	324	329	327	L-->I
2A7RB	324	329	328	S-->E
2A7RB	324	329	329	R-->L
2CB6G	232	237	233	S-->F
2CB6G	232	237	234	T-->K
1N32I	41	47	43	A-->W
1N32I	41	47	46	A-->S
1N32J	41	45	41	K-->A
1N32J	41	45	41	K-->P
1N32J	41	45	42	T-->G
1N32J	41	45	42	T-->H
1N32J	41	45	42	T-->V

1N32J	41 - 45	43	I-->G
1N32J	41 - 45	43	I-->L
1N32J	41 - 45	43	I-->R
1N32J	41 - 45	43	I-->S
1N32J	41 - 45	44	E-->R
1N32J	41 - 45	44	E-->S
1N32J	41 - 45	44	E-->W
1N32J	41 - 45	45	Q-->A
1N32J	41 - 45	45	Q-->C
1N32J	41 - 45	45	Q-->D
1N32J	41 - 45	45	Q-->E
1N32J	41 - 45	45	Q-->F
1N32J	41 - 45	45	Q-->I
1N32J	41 - 45	45	Q-->T
1N32J	41 - 45	45	Q-->V
1H6VC	462 - 467	463	Q-->A
1B26A	462 - 466	462	N-->A
1B26A	462 - 466	462	N-->E
1B26A	462 - 466	462	N-->G
1B26A	462 - 466	462	N-->P
1B26A	462 - 466	462	N-->Q
1B26A	462 - 466	462	N-->S
1B26A	462 - 466	462	N-->V
1B26A	462 - 466	463	E-->D
1B26A	462 - 466	463	E-->F
1B26A	462 - 466	463	E-->G
1B26A	462 - 466	463	E-->I

1B26A	462 - 466	463	E-->K
1B26A	462 - 466	463	E-->N
1B26A	462 - 466	463	E-->P
1B26A	462 - 466	463	E-->S
1B26A	462 - 466	463	E-->V
1B26A	462 - 466	463	E-->Y
1B26A	462 - 466	464	E-->A
1B26A	462 - 466	464	E-->D
1B26A	462 - 466	464	E-->G
1B26A	462 - 466	464	E-->S
1B26A	462 - 466	464	E-->V
1B26A	462 - 466	464	E-->W
1B26A	462 - 466	465	L-->C
1B26A	462 - 466	465	L-->Q
1B26A	462 - 466	465	L-->T
1B26A	462 - 466	465	L-->V
1B26A	462 - 466	466	L-->F
1B26A	462 - 466	466	L-->G
1B26A	462 - 466	466	L-->M
1B26A	462 - 466	466	L-->N
1B26A	462 - 466	466	L-->T
1B26A	462 - 466	466	L-->Y
3VTKA	62 - 67	67	Q-->G
3VTKA	85 - 89	85	M-->A
3VTKA	85 - 89	85	M-->E
3VTKA	85 - 89	85	M-->F
3VTKA	85 - 89	86	T-->G

3VTKA	85	89	87	Y-->G
3VTKA	85	89	88	W-->A
3VTKA	85	89	88	W-->Y
2MEV1	33	37	33	V-->N
2MEV1	33	37	34	A-->F
2MEV1	33	37	34	A-->L
2MEV1	33	37	35	F-->A
2MEV1	33	37	35	F-->E
2MEV1	33	37	35	F-->G
2MEV1	33	37	35	F-->P
2MEV1	33	37	35	F-->S
2MEV1	33	37	36	F-->A
2MEV1	33	37	36	F-->R
2MEV1	33	37	36	F-->S
2MEV1	33	37	36	F-->V
2MEV1	33	37	37	Y-->G
2MEV1	33	37	37	Y-->N
2MEV1	33	37	37	Y-->P
2MEV1	33	37	37	Y-->S
2MEV1	112	116	112	Y-->G
2MEV1	112	116	112	Y-->I
2MEV1	112	116	112	Y-->L
2MEV1	112	116	112	Y-->S
2MEV1	112	116	114	F-->Y
2MEV1	112	116	115	C-->A
2MEV1	112	116	115	C-->D
2MEV1	112	116	115	C-->R

2MEV1	112	116	115	C-->S
2MEV1	112	116	115	C-->T
2MEV1	112	116	115	C-->V
2MEV1	112	116	115	C-->W
2F9JP	112	116	112	D-->A
2F9JP	112	116	112	D-->E
2F9JP	112	116	112	D-->F
2F9JP	112	116	112	D-->I
2F9JP	112	116	112	D-->K
2F9JP	112	116	112	D-->L
2F9JP	112	116	112	D-->S
2F9JP	112	116	112	D-->V
2F9JP	112	116	112	D-->W
2F9JP	112	116	113	E-->A
2F9JP	112	116	113	E-->G
2F9JP	112	116	113	E-->M
2F9JP	112	116	113	E-->N
2F9JP	112	116	113	E-->P
2F9JP	112	116	113	E-->Q
2F9JP	112	116	113	E-->R
2F9JP	112	116	113	E-->T
2F9JP	112	116	114	E-->G
2F9JP	112	116	114	E-->H
2F9JP	112	116	114	E-->P
2F9JP	112	116	114	E-->S
2F9JP	112	116	114	E-->T
2F9JP	112	116	114	E-->V

2F9JP	112 - 116	115	L-->D
2F9JP	112 - 116	115	L-->F
2F9JP	112 - 116	115	L-->G
2F9JP	112 - 116	115	L-->H
2F9JP	112 - 116	115	L-->I
2F9JP	112 - 116	115	L-->N
2F9JP	112 - 116	115	L-->Q
2F9JP	112 - 116	115	L-->T
2F9JP	112 - 116	115	L-->V
2F9JP	112 - 116	115	L-->Y
2F9JP	112 - 116	116	D-->E
2F9JP	112 - 116	116	D-->G
2F9JP	112 - 116	116	D-->K
2F9JP	112 - 116	116	D-->N
2F9JP	112 - 116	116	D-->P
2F9JP	112 - 116	116	D-->R
2F9JP	112 - 116	116	D-->S
2F9JP	112 - 116	116	D-->T
2F9JP	112 - 116	116	D-->V
2EHOH	22 - 26	22	L-->D
2EHOH	22 - 26	22	L-->E
2EHOH	22 - 26	22	L-->M
2EHOH	22 - 26	22	L-->V
2EHOH	22 - 26	23	D-->E
2EHOH	22 - 26	23	D-->G
2EHOH	22 - 26	23	D-->K
2EHOH	22 - 26	23	D-->L

2EHOH	22 - 26	23	D-->M
2EHOH	22 - 26	23	D-->N
2EHOH	22 - 26	23	D-->P
2EHOH	22 - 26	23	D-->Q
2EHOH	22 - 26	23	D-->T
2EHOH	22 - 26	23	D-->V
2EHOH	22 - 26	24	D-->A
2EHOH	22 - 26	24	D-->G
2EHOH	22 - 26	24	D-->I
2EHOH	22 - 26	24	D-->L
2EHOH	22 - 26	24	D-->R
2EHOH	22 - 26	24	D-->S
2EHOH	22 - 26	24	D-->V
2EHOH	22 - 26	25	I-->D
2EHOH	22 - 26	25	I-->E
2EHOH	22 - 26	25	I-->G
2EHOH	22 - 26	25	I-->L
2EHOH	22 - 26	25	I-->M
2EHOH	22 - 26	25	I-->S
2EHOH	22 - 26	25	I-->T
2EHOH	22 - 26	26	L-->E
2EHOH	22 - 26	26	L-->H
2EHOH	22 - 26	26	L-->I
2EHOH	22 - 26	26	L-->N
2EHOH	22 - 26	26	L-->R
2EHOH	22 - 26	26	L-->T
2EHOH	22 - 26	26	L-->V

2EHOH	22 - 26	26	L-->Y
2EHOA	56 - 60	56	E-->S
2EHOA	56 - 60	57	Q-->H
2EHOA	56 - 60	57	Q-->P
2EHOA	56 - 60	57	Q-->V
2EHOA	56 - 60	58	L-->A
2EHOA	56 - 60	58	L-->G
2EHOA	56 - 60	58	L-->I
2EHOA	56 - 60	58	L-->P
2EHOA	56 - 60	59	E-->D
2EHOA	56 - 60	59	E-->Q
2EHOA	56 - 60	59	E-->V
2EHOA	56 - 60	59	E-->Y
2EHOA	56 - 60	60	H-->E
2EHOA	56 - 60	60	H-->G
2EHOA	56 - 60	60	H-->K
2EHOA	56 - 60	60	H-->L
2EHOA	56 - 60	60	H-->N
2EHOA	56 - 60	60	H-->Q
2EHOA	56 - 60	60	H-->R
2EHOA	56 - 60	60	H-->V
2EHOA	56 - 60	60	H-->W
2ZAGD	569 - 573	569	L-->F
2ZAGD	569 - 573	569	L-->I
2ZAGD	569 - 573	570	N-->D
2ZAGD	569 - 573	570	N-->E
2ZAGD	569 - 573	570	N-->V

2ZAGD	569 - 573	571	D-->G
2ZAGD	569 - 573	572	W-->A
2ZAGD	569 - 573	572	W-->G
2ZAGD	569 - 573	572	W-->L
2ZAGD	569 - 573	572	W-->M
2ZAGD	569 - 573	572	W-->N
2ZAGD	569 - 573	572	W-->Q
2ZAGD	569 - 573	572	W-->R
2ZAGD	569 - 573	572	W-->S
2ZAGD	569 - 573	572	W-->T
2ZAGD	569 - 573	572	W-->V
2ZAGD	569 - 573	573	A-->S
2ZAGD	569 - 573	569	R-->A
2ZAGD	569 - 573	569	R-->F
2ZAGD	569 - 573	570	R-->A
2ZAGD	569 - 573	570	R-->D
2ZAGD	569 - 573	570	R-->I
2ZAGD	569 - 573	570	R-->P
2ZAGD	569 - 573	570	R-->T
2ZAGD	569 - 573	571	E-->L
2ZAGD	569 - 573	572	Y-->G
2ZAGD	569 - 573	572	Y-->P
2ZAGD	569 - 573	572	Y-->R
2ZAGD	569 - 573	573	N-->C
2ZAGD	569 - 573	573	N-->F
2ZAGD	569 - 573	573	N-->G
2ZAGD	569 - 573	573	N-->H

2ZAGD	569 - 573	573	N-->I
2ZAGD	569 - 573	573	N-->T
1CJKA	552 - 556	552	A-->E
1CJKA	552 - 556	552	A-->G
1CJKA	552 - 556	552	A-->N
1CJKA	552 - 556	552	A-->P
1CJKA	552 - 556	553	Y-->A
1CJKA	552 - 556	553	Y-->F
1CJKA	552 - 556	553	Y-->K
1CJKA	552 - 556	553	Y-->L
1CJKA	552 - 556	553	Y-->M
1CJKA	552 - 556	553	Y-->P
1CJKA	552 - 556	553	Y-->Q
1CJKA	552 - 556	553	Y-->S
1CJKA	552 - 556	553	Y-->T
1CJKA	552 - 556	553	Y-->V
1CJKA	552 - 556	554	L-->A
1CJKA	552 - 556	554	L-->F
1CJKA	552 - 556	554	L-->I
1CJKA	552 - 556	554	L-->N
1CJKA	552 - 556	554	L-->R
1CJKA	552 - 556	555	K-->L
1CJKA	552 - 556	555	K-->P
1CJKA	552 - 556	555	K-->V
1CJKA	552 - 556	555	K-->Y
1CJKA	552 - 556	556	E-->A
1CJKA	552 - 556	556	E-->K

1CJKA	552 - 556	556	E-->Q	
1CJKA	552 - 556	556	E-->R	
1CJKA	552 - 556	556	E-->S	
1CJKA	552 - 556	556	E-->T	
1P3BC	552 - 556	552	R-->A	
1P3BC	552 - 556	552	R-->D	
1P3BC	552 - 556	552	R-->G	
1P3BC	552 - 556	552	R-->K	
1P3BC	552 - 556	552	R-->L	
1P3BC	552 - 556	552	R-->P	
1P3BC	552 - 556	552	R-->Q	
1P3BC	552 - 556	552	R-->S	
1P3BC	552 - 556	552	R-->T	
1P3BC	552 - 556	552	R-->V	
1P3BC	552 - 556	553	S-->I	
1P3BC	552 - 556	553	S-->T	
1P3BC	552 - 556	553	S-->Y	
1P3BC	552 - 556	554	S-->D	
1P3BC	552 - 556	554	S-->G	
1P3BC	552 - 556	554	S-->V	
1P3BC	552 - 556	554	S-->Y	
1P3BC	552 - 556	555	R-->D	
1P3BC	552 - 556	555	R-->E	
1P3BC	552 - 556	555	R-->G	
1P3BC	552 - 556	555	R-->H	
1P3BC	552 - 556	555	R-->K	
1P3BC	552 - 556	555	R-->L	

1P3BC	552 - 556	555	R-->P
1P3BC	552 - 556	555	R-->Q
1P3BC	552 - 556	555	R-->T
1P3BC	552 - 556	555	R-->Y
1P3BC	552 - 556	556	A-->K
1P3BC	552 - 556	556	A-->L
1P3BC	552 - 556	556	A-->M
1P3BC	552 - 556	556	A-->N
1P3BC	552 - 556	556	A-->P
1P3BC	552 - 556	556	A-->R
1P3BC	552 - 556	556	A-->S
1P3BC	552 - 556	556	A-->V
1P3BC	891 - 896	891	E-->T
2AVUA	72 - 77	72	H-->G
1QB3C	471 - 476	475	R-->E
1QB3C	471 - 476	476	G-->V
3BOPC	240 - 245	240	V-->G
3BOPC	240 - 245	240	V-->H
3BOPC	240 - 245	240	V-->L
3BOPC	240 - 245	240	V-->N
3BOPC	240 - 245	241	L-->G
3BOPC	240 - 245	241	L-->I
3BOPC	240 - 245	242	A-->V
3BOPC	240 - 245	243	L-->D
3BOPC	240 - 245	243	L-->P
3BOPC	240 - 245	244	A-->P
3BOPC	240 - 245	244	A-->R

3B57A	175 - 179	175	E-->C
3B57A	175 - 179	175	E-->I
3B57A	175 - 179	175	E-->K
3B57A	175 - 179	175	E-->L
3B57A	175 - 179	175	E-->M
3B57A	175 - 179	175	E-->Q
3B57A	175 - 179	175	E-->S
3B57A	175 - 179	175	E-->V
3B57A	175 - 179	175	E-->Y
3B57A	175 - 179	176	T-->E
3B57A	175 - 179	176	T-->G
3B57A	175 - 179	176	T-->H
3B57A	175 - 179	176	T-->K
3B57A	175 - 179	176	T-->N
3B57A	175 - 179	176	T-->S
3B57A	175 - 179	176	T-->V
3B57A	175 - 179	177	A-->E
3B57A	175 - 179	177	A-->F
3B57A	175 - 179	177	A-->G
3B57A	175 - 179	177	A-->L
3B57A	175 - 179	177	A-->P
3B57A	175 - 179	177	A-->R
3B57A	175 - 179	177	A-->T
3B57A	175 - 179	177	A-->V
3B57A	175 - 179	178	K-->A
3B57A	175 - 179	178	K-->D
3B57A	175 - 179	178	K-->F

3B57A	175	179	178	K-->G
3B57A	175	179	178	K-->L
3B57A	175	179	178	K-->M
3B57A	175	179	178	K-->S
3B57A	175	179	178	K-->Y
3B57A	175	179	179	T-->G
3B57A	175	179	179	T-->I
3B57A	175	179	179	T-->K
3B57A	175	179	179	T-->R
3B57A	175	179	179	T-->S
3B57A	182	187	183	E-->A
3B57A	182	187	184	K-->T
3B57A	182	187	185	Q-->V
1ZBE1	182	186	182	E-->A
1ZBE1	182	186	182	E-->D
1ZBE1	182	186	182	E-->F
1ZBE1	182	186	182	E-->G
1ZBE1	182	186	182	E-->K
1ZBE1	182	186	182	E-->L
1ZBE1	182	186	182	E-->P
1ZBE1	182	186	182	E-->S
1ZBE1	182	186	182	E-->V
1ZBE1	182	186	182	E-->W
1ZBE1	182	186	183	A-->E
1ZBE1	182	186	183	A-->G
1ZBE1	182	186	183	A-->H
1ZBE1	182	186	183	A-->I

1ZBE1	182 - 186	183	A-->K
1ZBE1	182 - 186	183	A-->L
1ZBE1	182 - 186	183	A-->M
1ZBE1	182 - 186	183	A-->N
1ZBE1	182 - 186	183	A-->S
1ZBE1	182 - 186	183	A-->T
1ZBE1	182 - 186	183	A-->V
1ZBE1	182 - 186	184	A-->C
1ZBE1	182 - 186	184	A-->D
1ZBE1	182 - 186	184	A-->E
1ZBE1	182 - 186	184	A-->I
1ZBE1	182 - 186	184	A-->L
1ZBE1	182 - 186	184	A-->N
1ZBE1	182 - 186	184	A-->P
1ZBE1	182 - 186	184	A-->V
1ZBE1	182 - 186	184	A-->W
1ZBE1	182 - 186	185	L-->A
1ZBE1	182 - 186	185	L-->D
1ZBE1	182 - 186	185	L-->H
1ZBE1	182 - 186	185	L-->I
1ZBE1	182 - 186	185	L-->N
1ZBE1	182 - 186	185	L-->P
1ZBE1	182 - 186	186	S-->A
1ZBE1	182 - 186	186	S-->C
1ZBE1	182 - 186	186	S-->D
1ZBE1	182 - 186	186	S-->F
1ZBE1	182 - 186	186	S-->I

1ZBE1	182 - 186	186	S-->K
1ZBE1	182 - 186	186	S-->L
1ZBE1	182 - 186	186	S-->P
1ZBE1	182 - 186	186	S-->Q
1ZBE1	182 - 186	186	S-->R
1ZBE1	182 - 186	186	S-->T
1ZBE1	182 - 186	186	S-->V
2ASSA	1087 - 1092	1088	W-->K
2ASSA	1150 - 1154	1150	E-->A
2ASSA	1150 - 1154	1150	E-->F
2ASSA	1150 - 1154	1150	E-->G
2ASSA	1150 - 1154	1150	E-->I
2ASSA	1150 - 1154	1150	E-->M
2ASSA	1150 - 1154	1150	E-->R
2ASSA	1150 - 1154	1150	E-->S
2ASSA	1150 - 1154	1150	E-->T
2ASSA	1150 - 1154	1151	A-->D
2ASSA	1150 - 1154	1151	A-->K
2ASSA	1150 - 1154	1151	A-->N
2ASSA	1150 - 1154	1151	A-->P
2ASSA	1150 - 1154	1151	A-->V
2ASSA	1150 - 1154	1152	Q-->D
2ASSA	1150 - 1154	1152	Q-->E
2ASSA	1150 - 1154	1152	Q-->G
2ASSA	1150 - 1154	1152	Q-->H
2ASSA	1150 - 1154	1152	Q-->I
2ASSA	1150 - 1154	1152	Q-->K

2ASSA	1150 - 1154	1152	Q-->P
2ASSA	1150 - 1154	1152	Q-->R
2ASSA	1150 - 1154	1152	Q-->S
2ASSA	1150 - 1154	1153	V-->K
2ASSA	1150 - 1154	1153	V-->M
2ASSA	1150 - 1154	1153	V-->Q
2ASSA	1150 - 1154	1154	R-->I
2ASSA	1150 - 1154	1154	R-->S
2ASSA	1150 - 1154	1154	R-->V
2ASSA	1150 - 1154	1154	R-->Y
2G6TA	52 - 57	55	I-->E
2G6TA	154 - 158	154	D-->G
2G6TA	154 - 158	155	Y-->D
2G6TA	154 - 158	155	Y-->G
2G6TA	154 - 158	155	Y-->H
2G6TA	154 - 158	155	Y-->I
2G6TA	154 - 158	155	Y-->L
2G6TA	154 - 158	155	Y-->M
2G6TA	154 - 158	155	Y-->N
2G6TA	154 - 158	155	Y-->S
2G6TA	154 - 158	155	Y-->T
2G6TA	154 - 158	155	Y-->V
2G6TA	154 - 158	156	Y-->H
2G6TA	154 - 158	156	Y-->Q
2G6TA	154 - 158	156	Y-->T
2G6TA	154 - 158	156	Y-->V
2G6TA	154 - 158	158	E-->F

2G6TA	283	- 288	284	W-->A
2G6TA	283	- 288	284	W-->I
2G6TA	283	- 288	284	W-->S
2BSQA	123	- 129	127	F-->K
2DZOD	368	- 372	368	L-->D
2DZOD	368	- 372	368	L-->E
2DZOD	368	- 372	368	L-->G
2DZOD	368	- 372	368	L-->H
2DZOD	368	- 372	368	L-->K
2DZOD	368	- 372	368	L-->R
2DZOD	368	- 372	368	L-->S
2DZOD	368	- 372	368	L-->T
2DZOD	368	- 372	368	L-->V
2DZOD	368	- 372	369	D-->A
2DZOD	368	- 372	369	D-->E
2DZOD	368	- 372	369	D-->G
2DZOD	368	- 372	369	D-->K
2DZOD	368	- 372	369	D-->S
2DZOD	368	- 372	369	D-->T
2DZOD	368	- 372	370	S-->A
2DZOD	368	- 372	370	S-->G
2DZOD	368	- 372	370	S-->I
2DZOD	368	- 372	370	S-->L
2DZOD	368	- 372	370	S-->M
2DZOD	368	- 372	370	S-->P
2DZOD	368	- 372	370	S-->Q
2DZOD	368	- 372	370	S-->T

2DZOD	368 - 372	370	S-->V
2DZOD	368 - 372	371	L-->D
2DZOD	368 - 372	371	L-->E
2DZOD	368 - 372	371	L-->I
2DZOD	368 - 372	371	L-->S
2DZOD	368 - 372	371	L-->T
2DZOD	368 - 372	371	L-->V
2DZOD	368 - 372	372	I-->A
2DZOD	368 - 372	372	I-->G
2DZOD	368 - 372	372	I-->H
2DZOD	368 - 372	372	I-->L
2DZOD	368 - 372	372	I-->N
2DZOD	368 - 372	372	I-->P
2DZOD	368 - 372	372	I-->Q
2DZOD	368 - 372	372	I-->R
2DZOD	368 - 372	372	I-->S
2DZOD	368 - 372	372	I-->T
2DZOD	368 - 372	372	I-->V
2OJED	171 - 175	171	K-->E
2OJED	171 - 175	171	K-->I
2OJED	171 - 175	171	K-->R
2OJED	171 - 175	171	K-->S
2OJED	171 - 175	171	K-->T
2OJED	171 - 175	171	K-->V
2OJED	171 - 175	171	K-->Y
2OJED	171 - 175	172	N-->E
2OJED	171 - 175	172	N-->I

2OJED	171	-	175	172	N-->L
2OJED	171	-	175	172	N-->S
2OJED	171	-	175	172	N-->T
2OJED	171	-	175	172	N-->V
2OJED	171	-	175	172	N-->Y
2OJED	171	-	175	173	V-->E
2OJED	171	-	175	173	V-->G
2OJED	171	-	175	173	V-->N
2OJED	171	-	175	173	V-->R
2OJED	171	-	175	174	K-->E
2OJED	171	-	175	174	K-->L
2OJED	171	-	175	174	K-->N
2OJED	171	-	175	174	K-->P
2OJED	171	-	175	174	K-->R
2OJED	171	-	175	174	K-->V
2OJED	171	-	175	175	E-->A
2OJED	171	-	175	175	E-->F
2OJED	171	-	175	175	E-->H
2OJED	171	-	175	175	E-->I
2OJED	171	-	175	175	E-->L
2OJED	171	-	175	175	E-->V
2OJED	171	-	175	175	E-->Y
2OJED	205	-	210	205	D-->G
2OJED	205	-	210	205	D-->S
2OJED	205	-	210	208	K-->E
1HLGA	71	-	75	71	A-->K
1HLGA	71	-	75	72	T-->W

1HLGA	71	75	74	W-->F
1HLGA	71	75	74	W-->G
1HLGA	71	75	74	W-->H
1HLGA	71	75	74	W-->I
1HLGA	71	75	74	W-->M
1HLGA	71	75	74	W-->P
1HLGA	71	75	74	W-->R
1HLGA	71	75	74	W-->S
1HLGA	71	75	74	W-->T
1HLGA	71	75	74	W-->V
1HLGA	71	75	74	W-->Y
1HLGA	71	75	75	I-->S
1HLGA	123	128	124	D-->P
1HLGA	123	128	126	M-->H
1HLGA	123	128	126	M-->N
1HLGA	123	128	126	M-->Q
1HLGA	239	244	240	L-->F
2FDMA	9	13	9	S-->G
2FDMA	9	13	10	W-->A
2FDMA	9	13	10	W-->F
2FDMA	9	13	10	W-->G
2FDMA	9	13	10	W-->H
2FDMA	9	13	10	W-->L
2FDMA	9	13	10	W-->Y
2FDMA	9	13	12	Q-->G
2FDMA	9	13	13	Y-->F
2FDMA	9	13	13	Y-->L

2FDMA	9 - 13	13	Y-->N
2AFVA	88 - 94	92	K-->Q
3CSMA	125 - 129	125	I-->E
3CSMA	125 - 129	125	I-->G
3CSMA	125 - 129	125	I-->K
3CSMA	125 - 129	125	I-->L
3CSMA	125 - 129	125	I-->T
3CSMA	125 - 129	125	I-->V
3CSMA	125 - 129	126	I-->A
3CSMA	125 - 129	126	I-->D
3CSMA	125 - 129	126	I-->G
3CSMA	125 - 129	126	I-->L
3CSMA	125 - 129	126	I-->Q
3CSMA	125 - 129	126	I-->R
3CSMA	125 - 129	126	I-->S
3CSMA	125 - 129	126	I-->T
3CSMA	125 - 129	126	I-->V
3CSMA	125 - 129	126	I-->Y
3CSMA	125 - 129	127	P-->E
3CSMA	125 - 129	127	P-->F
3CSMA	125 - 129	127	P-->H
3CSMA	125 - 129	127	P-->L
3CSMA	125 - 129	127	P-->N
3CSMA	125 - 129	128	L-->D
3CSMA	125 - 129	128	L-->F
3CSMA	125 - 129	128	L-->G
3CSMA	125 - 129	128	L-->N

3CSMA	125	129	128	L-->Q
3CSMA	125	129	128	L-->R
3CSMA	125	129	128	L-->Y
3CSMA	125	129	129	I-->D
3CSMA	125	129	129	I-->E
3CSMA	125	129	129	I-->K
3CSMA	125	129	129	I-->L
3CSMA	125	129	129	I-->P
3CSMA	125	129	129	I-->S
3CSMA	125	129	129	I-->Y
1G88B	412	417	416	R-->P
2PROC	137	143	139	A-->G
1WATA	147	151	147	D-->A
1WATA	147	151	147	D-->G
1WATA	147	151	147	D-->P
1WATA	147	151	147	D-->T
1WATA	147	151	147	D-->Y
1WATA	147	151	148	A-->D
1WATA	147	151	148	A-->G
1WATA	147	151	148	A-->I
1WATA	147	151	148	A-->L
1WATA	147	151	148	A-->T
1WATA	147	151	148	A-->V
1WATA	147	151	149	Y-->G
1WATA	147	151	149	Y-->M
1WATA	147	151	149	Y-->N
1WATA	147	151	150	F-->I

1WATA	147 - 151	150	F-->P
1WATA	147 - 151	150	F-->V
1WATA	147 - 151	151	A-->N
1WATA	147 - 151	151	A-->S
1WATA	147 - 151	151	A-->V
1RFBA	28 - 32	28	L-->D
1RFBA	28 - 32	28	L-->E
1RFBA	28 - 32	28	L-->F
1RFBA	28 - 32	28	L-->H
1RFBA	28 - 32	28	L-->I
1RFBA	28 - 32	28	L-->K
1RFBA	28 - 32	28	L-->N
1RFBA	28 - 32	28	L-->T
1RFBA	28 - 32	28	L-->V
1RFBA	28 - 32	29	F-->C
1RFBA	28 - 32	29	F-->D
1RFBA	28 - 32	29	F-->G
1RFBA	28 - 32	29	F-->H
1RFBA	28 - 32	29	F-->N
1RFBA	28 - 32	29	F-->R
1RFBA	28 - 32	29	F-->S
1RFBA	28 - 32	29	F-->V
1RFBA	28 - 32	30	S-->D
1RFBA	28 - 32	30	S-->F
1RFBA	28 - 32	30	S-->G
1RFBA	28 - 32	30	S-->L
1RFBA	28 - 32	30	S-->N

1RFBA	28 - 32	30	S-->V
1RFBA	28 - 32	31	E-->A
1RFBA	28 - 32	31	E-->D
1RFBA	28 - 32	31	E-->F
1RFBA	28 - 32	31	E-->I
1RFBA	28 - 32	31	E-->T
1RFBA	28 - 32	31	E-->V
1RFBA	28 - 32	32	I-->D
1RFBA	28 - 32	32	I-->L
1RFBA	28 - 32	32	I-->M
1RFBA	28 - 32	32	I-->P
1RFBA	28 - 32	32	I-->R
1RFBA	28 - 32	32	I-->T
1RFBA	88 - 92	88	K-->C
1RFBA	88 - 92	88	K-->D
1RFBA	88 - 92	88	K-->I
1RFBA	88 - 92	88	K-->P
1RFBA	88 - 92	88	K-->Q
1RFBA	88 - 92	88	K-->S
1RFBA	88 - 92	89	L-->A
1RFBA	88 - 92	89	L-->D
1RFBA	88 - 92	89	L-->G
1RFBA	88 - 92	89	L-->I
1RFBA	88 - 92	89	L-->K
1RFBA	88 - 92	89	L-->P
1RFBA	88 - 92	89	L-->Q
1RFBA	88 - 92	89	L-->V

1RFBA	88 - 92	89	L-->Y
1RFBA	88 - 92	90	E-->A
1RFBA	88 - 92	90	E-->C
1RFBA	88 - 92	90	E-->G
1RFBA	88 - 92	90	E-->I
1RFBA	88 - 92	90	E-->K
1RFBA	88 - 92	90	E-->L
1RFBA	88 - 92	90	E-->Q
1RFBA	88 - 92	90	E-->R
1RFBA	88 - 92	90	E-->S
1RFBA	88 - 92	90	E-->T
1RFBA	88 - 92	90	E-->V
1RFBA	88 - 92	91	D-->A
1RFBA	88 - 92	91	D-->I
1RFBA	88 - 92	91	D-->L
1RFBA	88 - 92	91	D-->N
1RFBA	88 - 92	91	D-->S
1RFBA	88 - 92	91	D-->V
1RFBA	88 - 92	92	F-->A
1RFBA	88 - 92	92	F-->G
1RFBA	88 - 92	92	F-->H
1RFBA	88 - 92	92	F-->I
1RFBA	88 - 92	92	F-->K
1RFBA	88 - 92	92	F-->L
1RFBA	88 - 92	92	F-->M
1RFBA	88 - 92	92	F-->N
1RFBA	88 - 92	92	F-->T

1RFBA	88	92	92	F-->V
1AUYB	57	63	59	V-->T
1AUYB	104	109	104	P-->K
1AUYB	104	109	108	T-->E
1EQUA	202	206	202	E-->A
1EQUA	202	206	202	E-->F
1EQUA	202	206	202	E-->G
1EQUA	202	206	202	E-->I
1EQUA	202	206	202	E-->K
1EQUA	202	206	202	E-->L
1EQUA	202	206	202	E-->R
1EQUA	202	206	202	E-->T
1EQUA	202	206	202	E-->V
1EQUA	202	206	202	E-->Y
1EQUA	202	206	203	V-->G
1EQUA	202	206	203	V-->I
1EQUA	202	206	203	V-->K
1EQUA	202	206	203	V-->N
1EQUA	202	206	203	V-->P
1EQUA	202	206	203	V-->Q
1EQUA	202	206	204	L-->A
1EQUA	202	206	204	L-->D
1EQUA	202	206	204	L-->E
1EQUA	202	206	204	L-->G
1EQUA	202	206	204	L-->K
1EQUA	202	206	204	L-->N
1EQUA	202	206	204	L-->Q

1EQUA	202 - 206	204	L-->R
1EQUA	202 - 206	204	L-->Y
1EQUA	202 - 206	205	D-->A
1EQUA	202 - 206	205	D-->E
1EQUA	202 - 206	205	D-->F
1EQUA	202 - 206	205	D-->G
1EQUA	202 - 206	205	D-->K
1EQUA	202 - 206	205	D-->L
1EQUA	202 - 206	205	D-->N
1EQUA	202 - 206	205	D-->P
1EQUA	202 - 206	205	D-->S
1EQUA	202 - 206	205	D-->V
1EQUA	202 - 206	206	R-->E
1EQUA	202 - 206	206	R-->G
1EQUA	202 - 206	206	R-->H
1EQUA	202 - 206	206	R-->I
1EQUA	202 - 206	206	R-->K
1EQUA	202 - 206	206	R-->L
1EQUA	202 - 206	206	R-->N
1EQUA	202 - 206	206	R-->P
1EQUA	202 - 206	206	R-->S
1EQUA	202 - 206	206	R-->T
1EQUA	202 - 206	206	R-->V
1EQUA	202 - 206	206	R-->Y
2UWYA	468 - 473	468	Y-->D
2UWYA	468 - 473	468	Y-->P
2UWYA	468 - 473	470	I-->F

2UWYA	468	- 473	470	I-->P
2UWYA	468	- 473	472	I-->E
2UWYA	468	- 473	472	I-->R
2UWYA	468	- 473	473	D-->N
2FHH2	391	- 396	391	L-->W
2JBKA	58	- 63	60	A-->I
2JBKA	522	- 526	522	E-->N
2JBKA	522	- 526	522	E-->S
2JBKA	522	- 526	522	E-->V
2JBKA	522	- 526	524	F-->K
2JBKA	522	- 526	524	F-->L
2JBKA	522	- 526	524	F-->S
2JBKA	522	- 526	525	F-->G
2JBKA	522	- 526	525	F-->H
2JBKA	522	- 526	525	F-->P
2JBKA	522	- 526	525	F-->R
2JBKA	522	- 526	526	Q-->D
2JBKA	522	- 526	526	Q-->I
2JBKA	522	- 526	526	Q-->L
2JBKA	522	- 526	526	Q-->S
2JBKA	522	- 526	526	Q-->V
2JBKA	619	- 624	619	P-->L
2OREF	231	- 236	231	M-->R
1JUMA	25	- 31	30	K-->A
1IM0A	18	- 23	20	A-->G
1IM0A	188	- 192	188	I-->K
1IM0A	188	- 192	189	T-->G

1IM0A	188 - 192	190	K-->A	
1IM0A	188 - 192	190	K-->D	
1IM0A	188 - 192	190	K-->E	
1IM0A	188 - 192	190	K-->S	
1IM0A	188 - 192	190	K-->V	
1IM0A	188 - 192	191	Y-->D	
1IM0A	188 - 192	191	Y-->K	
1IM0A	188 - 192	191	Y-->T	
1IM0A	188 - 192	191	Y-->V	
1IM0A	188 - 192	192	M-->G	
1IM0A	188 - 192	192	M-->S	
1IM0A	188 - 192	192	M-->T	
1KYOI	5 - 10	6	L-->K	
1KYOI	5 - 10	10	F-->D	
1KYOI	49 - 54	51	D-->N	
1KYOI	49 - 54	52	V-->D	
1P8OA	36 - 41	36	L-->P	
1P8OA	36 - 41	36	L-->R	
1P8OA	36 - 41	39	K-->I	
1P8OA	36 - 41	39	K-->P	
1P8OA	36 - 41	41	K-->S	
1P8OA	144 - 148	144	P-->A	
1P8OA	144 - 148	144	P-->D	
1P8OA	144 - 148	144	P-->E	
1P8OA	144 - 148	144	P-->G	
1P8OA	144 - 148	144	P-->H	
1P8OA	144 - 148	144	P-->K	

1P8OA	144 - 148	144	P-->T
1P8OA	144 - 148	145	V-->F
1P8OA	144 - 148	145	V-->G
1P8OA	144 - 148	145	V-->I
1P8OA	144 - 148	145	V-->L
1P8OA	144 - 148	145	V-->R
1P8OA	144 - 148	145	V-->T
1P8OA	144 - 148	145	V-->W
1P8OA	144 - 148	146	A-->E
1P8OA	144 - 148	146	A-->G
1P8OA	144 - 148	146	A-->I
1P8OA	144 - 148	146	A-->K
1P8OA	144 - 148	146	A-->L
1P8OA	144 - 148	146	A-->P
1P8OA	144 - 148	146	A-->R
1P8OA	144 - 148	146	A-->T
1P8OA	144 - 148	146	A-->V
1P8OA	144 - 148	147	F-->A
1P8OA	144 - 148	147	F-->D
1P8OA	144 - 148	147	F-->G
1P8OA	144 - 148	147	F-->H
1P8OA	144 - 148	147	F-->I
1P8OA	144 - 148	147	F-->L
1P8OA	144 - 148	147	F-->M
1P8OA	144 - 148	147	F-->P
1P8OA	144 - 148	147	F-->R
1P8OA	144 - 148	147	F-->T

1P8OA	144 - 148	147	F-->V
1P8OA	144 - 148	147	F-->W
1P8OA	144 - 148	147	F-->Y
1P8OA	144 - 148	148	L-->A
1P8OA	144 - 148	148	L-->D
1P8OA	144 - 148	148	L-->S
1P8OA	144 - 148	148	L-->V
2NWLA	142 - 147	143	F-->E
2NWLA	142 - 147	143	F-->M
2NWLA	142 - 147	143	F-->T
2NWLA	142 - 147	143	F-->V
2NWLA	142 - 147	144	G-->S
2NWLA	142 - 147	145	A-->P
2NWLA	142 - 147	146	L-->R
2CFQA	142 - 146	142	F-->A
2CFQA	105 - 111	105	V-->T
2CFQA	210 - 216	213	A-->E
1KA8A	92 - 96	92	E-->C
1KA8A	92 - 96	92	E-->D
1KA8A	92 - 96	92	E-->I
1KA8A	92 - 96	92	E-->L
1KA8A	92 - 96	92	E-->P
1KA8A	92 - 96	92	E-->S
1KA8A	92 - 96	92	E-->V
1KA8A	92 - 96	92	E-->Y
1KA8A	92 - 96	93	S-->A
1KA8A	92 - 96	93	S-->D

1KA8A	92 - 96	93	S-->E
1KA8A	92 - 96	93	S-->G
1KA8A	92 - 96	93	S-->L
1KA8A	92 - 96	93	S-->W
1KA8A	92 - 96	94	Y-->A
1KA8A	92 - 96	94	Y-->F
1KA8A	92 - 96	94	Y-->G
1KA8A	92 - 96	94	Y-->H
1KA8A	92 - 96	94	Y-->L
1KA8A	92 - 96	94	Y-->N
1KA8A	92 - 96	94	Y-->P
1KA8A	92 - 96	94	Y-->S
1KA8A	92 - 96	94	Y-->V
1KA8A	92 - 96	95	G-->C
1KA8A	92 - 96	95	G-->D
1KA8A	92 - 96	95	G-->E
1KA8A	92 - 96	95	G-->I
1KA8A	92 - 96	95	G-->K
1KA8A	92 - 96	95	G-->N
1KA8A	92 - 96	95	G-->P
1KA8A	92 - 96	95	G-->Q
1KA8A	92 - 96	95	G-->V
1KA8A	92 - 96	96	D-->C
1KA8A	92 - 96	96	D-->T
1KA8A	92 - 96	96	D-->V
1WTHD	200 - 204	200	Y-->D
1WTHD	200 - 204	200	Y-->I

1WTHD	200 - 204	201	D-->M
1WTHD	200 - 204	202	M-->F
1WTHD	200 - 204	202	M-->I
1WTHD	200 - 204	202	M-->S
1WTHD	200 - 204	203	M-->V
1WTHD	200 - 204	204	I-->P
1WTHD	200 - 204	204	I-->R
1WTHD	281 - 285	281	G-->A
1WTHD	281 - 285	281	G-->D
1WTHD	281 - 285	281	G-->L
1WTHD	281 - 285	281	G-->V
1WTHD	281 - 285	282	A-->E
1WTHD	281 - 285	282	A-->G
1WTHD	281 - 285	282	A-->K
1WTHD	281 - 285	282	A-->L
1WTHD	281 - 285	282	A-->P
1WTHD	281 - 285	282	A-->T
1WTHD	281 - 285	283	Y-->A
1WTHD	281 - 285	283	Y-->D
1WTHD	281 - 285	283	Y-->E
1WTHD	281 - 285	283	Y-->G
1WTHD	281 - 285	283	Y-->H
1WTHD	281 - 285	283	Y-->I
1WTHD	281 - 285	283	Y-->K
1WTHD	281 - 285	283	Y-->M
1WTHD	281 - 285	283	Y-->N
1WTHD	281 - 285	283	Y-->P

1WTHD	281	285	283	Y-->R
1WTHD	281	285	283	Y-->S
1WTHD	281	285	283	Y-->T
1WTHD	281	285	283	Y-->V
1WTHD	281	285	283	Y-->W
1WTHD	281	285	284	S-->A
1WTHD	281	285	284	S-->C
1WTHD	281	285	284	S-->D
1WTHD	281	285	284	S-->E
1WTHD	281	285	284	S-->G
1WTHD	281	285	284	S-->L
1WTHD	281	285	284	S-->N
1WTHD	281	285	284	S-->R
1WTHD	281	285	285	E-->A
1WTHD	281	285	285	E-->F
1WTHD	281	285	285	E-->G
1WTHD	281	285	285	E-->K
1WTHD	281	285	285	E-->L
1WTHD	281	285	285	E-->M
1WTHD	281	285	285	E-->N
1WTHD	281	285	285	E-->P
1WTHD	281	285	285	E-->Q
1WTHD	281	285	285	E-->S
1WTHD	281	285	285	E-->V
2R17A	20	25	24	K-->N
2HLDI	32	37	32	A-->N
2HLDI	32	37	33	S-->L

2HLDI	32 - 37	33	S-->R
1L8OA	32 - 37	32	D-->F
1L8OA	32 - 37	33	E-->N
1L8OA	32 - 37	33	E-->S
1L8OA	32 - 37	34	L-->Q
1L8OA	32 - 37	34	L-->T
1L8OA	157 - 162	157	G-->F
1L8OA	157 - 162	157	G-->Q
1L8OA	157 - 162	157	G-->R
1L8OA	157 - 162	157	G-->V
1L8OA	157 - 162	158	K-->G
1L8OA	157 - 162	158	K-->V
1L8OA	157 - 162	159	G-->T
1L8OA	157 - 162	159	G-->V
1L8OA	157 - 162	160	K-->C
1L8OA	157 - 162	160	K-->E
1L8OA	157 - 162	160	K-->G
1L8OA	157 - 162	160	K-->M
1L8OA	157 - 162	160	K-->P
1L8OA	157 - 162	160	K-->Q
1L8OA	157 - 162	161	V-->H
1L8OA	157 - 162	161	V-->I
1L8OA	157 - 162	162	I-->G
1L8OA	157 - 162	162	I-->K
1L8OA	157 - 162	162	I-->L
1Z50A	247 - 252	249	D-->F
1Z50A	314 - 319	316	W-->A

1Z50A	314 - 319	316	W-->I
1KQGB	107 - 111	107	G-->F
1KQGB	107 - 111	107	G-->I
1KQGB	107 - 111	107	G-->K
1KQGB	107 - 111	107	G-->Q
1KQGB	107 - 111	108	C-->A
1KQGB	107 - 111	108	C-->D
1KQGB	107 - 111	108	C-->E
1KQGB	107 - 111	108	C-->F
1KQGB	107 - 111	108	C-->G
1KQGB	107 - 111	108	C-->I
1KQGB	107 - 111	108	C-->K
1KQGB	107 - 111	108	C-->L
1KQGB	107 - 111	108	C-->N
1KQGB	107 - 111	108	C-->Q
1KQGB	107 - 111	108	C-->R
1KQGB	107 - 111	108	C-->S
1KQGB	107 - 111	108	C-->T
1KQGB	107 - 111	108	C-->V
1KQGB	107 - 111	108	C-->Y
1KQGB	107 - 111	109	L-->C
1KQGB	107 - 111	109	L-->R
1KQGB	107 - 111	109	L-->S
1KQGB	107 - 111	110	K-->D
1KQGB	107 - 111	110	K-->F
1KQGB	107 - 111	110	K-->H
1KQGB	107 - 111	110	K-->L

1KQGB	107	-	111	110	K-->P
1KQGB	107	-	111	111	A-->G
1KQGB	107	-	111	111	A-->L
1KQGB	107	-	111	111	A-->T
1KQGB	138	-	142	138	Y-->G
1KQGB	138	-	142	138	Y-->H
1KQGB	138	-	142	138	Y-->Q
1KQGB	138	-	142	138	Y-->S
1KQGB	138	-	142	138	Y-->T
1KQGB	138	-	142	138	Y-->V
1KQGB	138	-	142	139	C-->A
1KQGB	138	-	142	139	C-->E
1KQGB	138	-	142	139	C-->F
1KQGB	138	-	142	139	C-->G
1KQGB	138	-	142	139	C-->I
1KQGB	138	-	142	139	C-->K
1KQGB	138	-	142	139	C-->L
1KQGB	138	-	142	139	C-->N
1KQGB	138	-	142	139	C-->P
1KQGB	138	-	142	139	C-->T
1KQGB	138	-	142	140	I-->A
1KQGB	138	-	142	140	I-->E
1KQGB	138	-	142	140	I-->G
1KQGB	138	-	142	140	I-->L
1KQGB	138	-	142	140	I-->N
1KQGB	138	-	142	140	I-->P
1KQGB	138	-	142	141	A-->C

1KQGB	138	142	141	A-->V
1KQGB	138	142	142	G-->N
1KQGB	174	178	174	A-->E
1KQGB	174	178	174	A-->H
1KQGB	174	178	174	A-->L
1KQGB	174	178	175	C-->D
1KQGB	174	178	175	C-->G
1KQGB	174	178	175	C-->I
1KQGB	174	178	175	C-->L
1KQGB	174	178	175	C-->N
1KQGB	174	178	175	C-->V
1KQGB	174	178	175	C-->W
1KQGB	174	178	177	K-->A
1KQGB	174	178	177	K-->C
1KQGB	174	178	178	T-->A
1KQGB	174	178	178	T-->G
1KQGB	174	178	178	T-->K
2I3CA	55	59	55	P-->F
2I3CA	55	59	55	P-->G
2I3CA	55	59	55	P-->L
2I3CA	55	59	55	P-->R
2I3CA	55	59	55	P-->V
2I3CA	55	59	56	R-->D
2I3CA	55	59	56	R-->E
2I3CA	55	59	56	R-->F
2I3CA	55	59	56	R-->H
2I3CA	55	59	56	R-->I

2I3CA	55 - 59	56	R-->L
2I3CA	55 - 59	56	R-->N
2I3CA	55 - 59	56	R-->Q
2I3CA	55 - 59	56	R-->T
2I3CA	55 - 59	56	R-->V
2I3CA	55 - 59	56	R-->Y
2I3CA	55 - 59	57	A-->D
2I3CA	55 - 59	57	A-->E
2I3CA	55 - 59	57	A-->G
2I3CA	55 - 59	57	A-->I
2I3CA	55 - 59	57	A-->K
2I3CA	55 - 59	57	A-->L
2I3CA	55 - 59	57	A-->P
2I3CA	55 - 59	57	A-->Q
2I3CA	55 - 59	57	A-->S
2I3CA	55 - 59	57	A-->T
2I3CA	55 - 59	57	A-->V
2I3CA	55 - 59	57	A-->Y
2I3CA	55 - 59	58	V-->E
2I3CA	55 - 59	58	V-->K
2I3CA	55 - 59	58	V-->M
2I3CA	55 - 59	59	K-->A
2I3CA	55 - 59	59	K-->D
2I3CA	55 - 59	59	K-->F
2I3CA	55 - 59	59	K-->G
2I3CA	55 - 59	59	K-->L
2I3CA	55 - 59	59	K-->P

2I3CA	55 - 59	59	K-->Q
2I3CA	55 - 59	59	K-->R
2I3CA	55 - 59	59	K-->Y
2I3CA	134 - 138	134	N-->C
2I3CA	134 - 138	134	N-->H
2I3CA	134 - 138	134	N-->K
2I3CA	134 - 138	134	N-->S
2I3CA	134 - 138	135	F-->C
2I3CA	134 - 138	135	F-->E
2I3CA	134 - 138	135	F-->G
2I3CA	134 - 138	135	F-->M
2I3CA	134 - 138	135	F-->S
2I3CA	134 - 138	135	F-->T
2I3CA	134 - 138	135	F-->W
2I3CA	134 - 138	136	L-->I
2I3CA	134 - 138	136	L-->M
2I3CA	134 - 138	136	L-->P
2I3CA	134 - 138	136	L-->R
2I3CA	134 - 138	136	L-->S
2I3CA	134 - 138	137	I-->E
2I3CA	134 - 138	138	Q-->G
2I3CA	134 - 138	138	Q-->P
2I3CA	134 - 138	138	Q-->R
2I3CA	134 - 138	138	Q-->W
2I3CA	189 - 194	194	Q-->I
2I3CA	189 - 194	194	Q-->V
2BPCA	143 - 147	143	F-->E

2BPCA	143 - 147	143	F-->G
2BPCA	143 - 147	143	F-->H
2BPCA	143 - 147	143	F-->I
2BPCA	143 - 147	143	F-->N
2BPCA	143 - 147	143	F-->Q
2BPCA	143 - 147	143	F-->R
2BPCA	143 - 147	143	F-->S
2BPCA	143 - 147	143	F-->T
2BPCA	143 - 147	143	F-->Y
2BPCA	143 - 147	144	E-->G
2BPCA	143 - 147	144	E-->L
2BPCA	143 - 147	144	E-->Q
2BPCA	143 - 147	144	E-->S
2BPCA	143 - 147	145	D-->F
2BPCA	143 - 147	145	D-->G
2BPCA	143 - 147	145	D-->I
2BPCA	143 - 147	145	D-->K
2BPCA	143 - 147	145	D-->Q
2BPCA	143 - 147	145	D-->R
2BPCA	143 - 147	145	D-->T
2BPCA	143 - 147	145	D-->V
2BPCA	143 - 147	146	F-->A
2BPCA	143 - 147	146	F-->G
2BPCA	143 - 147	146	F-->H
2BPCA	143 - 147	146	F-->P
2BPCA	143 - 147	146	F-->S
2BPCA	143 - 147	146	F-->V

2BPCA	143	147	146	F-->Y
2BPCA	143	147	147	E-->A
2BPCA	143	147	147	E-->D
2BPCA	143	147	147	E-->G
2BPCA	143	147	147	E-->P
2BPCA	143	147	147	E-->S
2BPCA	265	271	267	C-->D
2FYIA	101	106	101	H-->E
2C3ZA	216	222	217	N-->I
2C3ZA	234	238	234	S-->Q
2C3ZA	234	238	235	S-->Q
2C3ZA	234	238	236	L-->E
2C3ZA	234	238	236	L-->G
2C3ZA	234	238	236	L-->I
2C3ZA	234	238	237	M-->D
2C3ZA	234	238	237	M-->F
2C3ZA	234	238	237	M-->G
2C3ZA	234	238	237	M-->H
2C3ZA	234	238	237	M-->I
2C3ZA	234	238	237	M-->K
2C3ZA	234	238	237	M-->P
2C3ZA	234	238	237	M-->T
2C3ZA	234	238	237	M-->V
2C3ZA	234	238	238	R-->D
2C3ZA	234	238	238	R-->F
2C3ZA	234	238	238	R-->P
2C3ZA	234	238	238	R-->Y

2RIRE	87	91	87	Q-->R
2RIRE	87	91	87	Q-->T
2RIRE	87	91	88	D-->L
2RIRE	87	91	88	D-->T
2RIRE	87	91	88	D-->V
2RIRE	87	91	89	H-->A
2RIRE	87	91	89	H-->D
2RIRE	87	91	89	H-->E
2RIRE	87	91	89	H-->L
2RIRE	87	91	91	D-->E
2RIRE	87	91	91	D-->R
2RIRE	87	91	91	D-->S
2RIRE	87	91	91	D-->V
2RIRE	267	271	267	L-->C
2RIRE	267	271	267	L-->E
2RIRE	267	271	267	L-->G
2RIRE	267	271	267	L-->N
2RIRE	267	271	267	L-->P
2RIRE	267	271	267	L-->Q
2RIRE	267	271	268	P-->A
2RIRE	267	271	268	P-->C
2RIRE	267	271	268	P-->D
2RIRE	267	271	268	P-->E
2RIRE	267	271	268	P-->F
2RIRE	267	271	268	P-->H
2RIRE	267	271	268	P-->K
2RIRE	267	271	268	P-->Q

2RIRE	267 - 271	268	P-->S	
2RIRE	267 - 271	268	P-->T	
2RIRE	267 - 271	268	P-->V	
2RIRE	267 - 271	269	G-->A	
2RIRE	267 - 271	269	G-->D	
2RIRE	267 - 271	269	G-->H	
2RIRE	267 - 271	269	G-->K	
2RIRE	267 - 271	269	G-->N	
2RIRE	267 - 271	269	G-->Q	
2RIRE	267 - 271	269	G-->R	
2RIRE	267 - 271	269	G-->T	
2RIRE	267 - 271	269	G-->V	
2RIRE	267 - 271	269	G-->Y	
2RIRE	267 - 271	270	I-->A	
2RIRE	267 - 271	270	I-->C	
2RIRE	267 - 271	270	I-->D	
2RIRE	267 - 271	270	I-->E	
2RIRE	267 - 271	270	I-->F	
2RIRE	267 - 271	270	I-->G	
2RIRE	267 - 271	270	I-->K	
2RIRE	267 - 271	270	I-->L	
2RIRE	267 - 271	270	I-->M	
2RIRE	267 - 271	270	I-->N	
2RIRE	267 - 271	270	I-->R	
2RIRE	267 - 271	270	I-->T	
2RIRE	267 - 271	270	I-->V	
2RIRE	267 - 271	270	I-->W	

2RIRE	267 - 271	270	I-->Y
2RIRE	267 - 271	271	V-->D
2RIRE	267 - 271	271	V-->E
2RIRE	267 - 271	271	V-->G
2RIRE	267 - 271	271	V-->I
2RIRE	267 - 271	271	V-->M
2RIRE	267 - 271	271	V-->N
2RIRE	267 - 271	271	V-->P
2RIRE	267 - 271	271	V-->R
2RIRE	267 - 271	271	V-->S
2RIRE	267 - 271	271	V-->T
2RIRE	267 - 271	271	V-->Y
2HWYB	1005 - 1010	1009	Y-->Q
2P2UA	111 - 117	117	Q-->L
2P2UA	111 - 115	111	D-->A
2P2UA	111 - 115	111	D-->F
2P2UA	111 - 115	111	D-->G
2P2UA	111 - 115	111	D-->I
2P2UA	111 - 115	111	D-->K
2P2UA	111 - 115	111	D-->L
2P2UA	111 - 115	111	D-->N
2P2UA	111 - 115	111	D-->P
2P2UA	111 - 115	111	D-->T
2P2UA	111 - 115	112	E-->G
2P2UA	111 - 115	112	E-->H
2P2UA	111 - 115	112	E-->K
2P2UA	111 - 115	112	E-->L

2P2UA	111	115	112	E-->P
2P2UA	111	115	113	R-->D
2P2UA	111	115	113	R-->E
2P2UA	111	115	113	R-->F
2P2UA	111	115	113	R-->G
2P2UA	111	115	113	R-->L
2P2UA	111	115	113	R-->N
2P2UA	111	115	113	R-->P
2P2UA	111	115	113	R-->Q
2P2UA	111	115	113	R-->S
2P2UA	111	115	113	R-->V
2P2UA	111	115	113	R-->W
2P2UA	111	115	114	L-->E
2P2UA	111	115	114	L-->F
2P2UA	111	115	114	L-->P
2P2UA	111	115	114	L-->R
2P2UA	111	115	114	L-->Y
2P2UA	111	115	115	E-->G
2P2UA	111	115	115	E-->H
2P2UA	111	115	115	E-->I
2P2UA	111	115	115	E-->N
2P2UA	111	115	115	E-->P
2P2UA	111	115	115	E-->S
2P2UA	111	115	115	E-->T
2P2UA	111	115	115	E-->V
2P2UA	111	115	115	E-->Y
2J0DB	32	36	32	L-->E

2J0DB	32 - 36	32	L-->G
2J0DB	32 - 36	32	L-->H
2J0DB	32 - 36	32	L-->P
2J0DB	32 - 36	32	L-->S
2J0DB	32 - 36	32	L-->T
2J0DB	32 - 36	33	F-->E
2J0DB	32 - 36	33	F-->G
2J0DB	32 - 36	33	F-->H
2J0DB	32 - 36	33	F-->K
2J0DB	32 - 36	33	F-->N
2J0DB	32 - 36	33	F-->P
2J0DB	32 - 36	33	F-->R
2J0DB	32 - 36	33	F-->S
2J0DB	32 - 36	33	F-->V
2J0DB	32 - 36	34	K-->A
2J0DB	32 - 36	34	K-->D
2J0DB	32 - 36	34	K-->H
2J0DB	32 - 36	34	K-->N
2J0DB	32 - 36	34	K-->Q
2J0DB	32 - 36	34	K-->V
2J0DB	32 - 36	35	K-->A
2J0DB	32 - 36	35	K-->D
2J0DB	32 - 36	35	K-->E
2J0DB	32 - 36	35	K-->G
2J0DB	32 - 36	35	K-->L
2J0DB	32 - 36	35	K-->P
2J0DB	32 - 36	35	K-->Q

2J0DB	32 - 36	35	K-->V
2J0DB	32 - 36	36	L-->D
2J0DB	32 - 36	36	L-->G
2J0DB	32 - 36	36	L-->K
2J0DB	32 - 36	36	L-->N
2J0DB	32 - 36	36	L-->T
2J0DB	32 - 36	36	L-->V
2J0DB	112 - 116	113	F-->E
2J0DB	112 - 116	113	F-->V
2J0DB	112 - 116	114	M-->A
2J0DB	112 - 116	114	M-->E
2J0DB	112 - 116	114	M-->F
2J0DB	112 - 116	114	M-->G
2J0DB	112 - 116	114	M-->N
2J0DB	112 - 116	114	M-->R
2J0DB	112 - 116	114	M-->S
2J0DB	112 - 116	115	K-->D
2J0DB	112 - 116	115	K-->N
2J0DB	112 - 116	115	K-->P
2J0DB	112 - 116	115	K-->S
2J0DB	112 - 116	115	K-->Y
2J0DB	112 - 116	116	S-->A
2J0DB	112 - 116	116	S-->Q
2J0DB	112 - 116	116	S-->R
2J0DB	112 - 116	116	S-->T
2J0DB	204 - 208	204	V-->G
2J0DB	204 - 208	204	V-->K

2J0DB	204 - 208	204	V-->P
2J0DB	204 - 208	204	V-->Y
2J0DB	204 - 208	205	E-->C
2J0DB	204 - 208	205	E-->D
2J0DB	204 - 208	205	E-->G
2J0DB	204 - 208	205	E-->N
2J0DB	204 - 208	205	E-->P
2J0DB	204 - 208	205	E-->S
2J0DB	204 - 208	205	E-->Y
2J0DB	204 - 208	206	N-->C
2J0DB	204 - 208	206	N-->D
2J0DB	204 - 208	206	N-->G
2J0DB	204 - 208	206	N-->I
2J0DB	204 - 208	206	N-->L
2J0DB	204 - 208	206	N-->P
2J0DB	204 - 208	206	N-->T
2J0DB	204 - 208	206	N-->V
2J0DB	204 - 208	206	N-->Y
2J0DB	204 - 208	207	T-->A
2J0DB	204 - 208	207	T-->D
2J0DB	204 - 208	207	T-->E
2J0DB	204 - 208	207	T-->F
2J0DB	204 - 208	207	T-->G
2J0DB	204 - 208	207	T-->K
2J0DB	204 - 208	207	T-->M
2J0DB	204 - 208	207	T-->P
2J0DB	204 - 208	208	K-->D

2J0DB	204 - 208	208	K-->E
2J0DB	204 - 208	208	K-->F
2J0DB	204 - 208	208	K-->H
2J0DB	204 - 208	208	K-->L
2J0DB	204 - 208	208	K-->R
2J0DB	229 - 235	229	L-->K
1Y8RA	216 - 220	216	V-->E
1Y8RA	216 - 220	216	V-->G
1Y8RA	216 - 220	216	V-->H
1Y8RA	216 - 220	216	V-->L
1Y8RA	216 - 220	216	V-->M
1Y8RA	216 - 220	216	V-->N
1Y8RA	216 - 220	216	V-->T
1Y8RA	216 - 220	216	V-->W
1Y8RA	216 - 220	217	K-->A
1Y8RA	216 - 220	217	K-->D
1Y8RA	216 - 220	217	K-->F
1Y8RA	216 - 220	217	K-->G
1Y8RA	216 - 220	217	K-->H
1Y8RA	216 - 220	217	K-->I
1Y8RA	216 - 220	217	K-->L
1Y8RA	216 - 220	217	K-->N
1Y8RA	216 - 220	217	K-->P
1Y8RA	216 - 220	217	K-->S
1Y8RA	216 - 220	217	K-->T
1Y8RA	216 - 220	217	K-->V
1Y8RA	216 - 220	217	K-->Y

1Y8RA	216 - 220	218	E-->F
1Y8RA	216 - 220	218	E-->I
1Y8RA	216 - 220	218	E-->K
1Y8RA	216 - 220	218	E-->N
1Y8RA	216 - 220	218	E-->T
1Y8RA	216 - 220	218	E-->Y
1Y8RA	216 - 220	219	A-->E
1Y8RA	216 - 220	219	A-->F
1Y8RA	216 - 220	219	A-->G
1Y8RA	216 - 220	219	A-->H
1Y8RA	216 - 220	219	A-->I
1Y8RA	216 - 220	219	A-->K
1Y8RA	216 - 220	219	A-->L
1Y8RA	216 - 220	219	A-->P
1Y8RA	216 - 220	219	A-->Q
1Y8RA	216 - 220	219	A-->R
1Y8RA	216 - 220	219	A-->W
1Y8RA	216 - 220	220	L-->A
1Y8RA	216 - 220	220	L-->C
1Y8RA	216 - 220	220	L-->E
1Y8RA	216 - 220	220	L-->F
1Y8RA	216 - 220	220	L-->I
1Y8RA	216 - 220	220	L-->N
1Y8RA	216 - 220	220	L-->P
1Y8RA	216 - 220	220	L-->Q
1Y8RA	216 - 220	220	L-->R
1Y8RA	216 - 220	216	E-->A

1Y8RA	216 - 220	216	E-->C
1Y8RA	216 - 220	216	E-->G
1Y8RA	216 - 220	216	E-->I
1Y8RA	216 - 220	216	E-->S
1Y8RA	216 - 220	216	E-->V
1Y8RA	216 - 220	217	D-->H
1Y8RA	216 - 220	217	D-->L
1Y8RA	216 - 220	217	D-->Y
1Y8RA	216 - 220	218	F-->A
1Y8RA	216 - 220	218	F-->E
1Y8RA	216 - 220	218	F-->G
1Y8RA	216 - 220	218	F-->K
1Y8RA	216 - 220	218	F-->L
1Y8RA	216 - 220	218	F-->Q
1Y8RA	216 - 220	218	F-->R
1Y8RA	216 - 220	218	F-->V
1Y8RA	216 - 220	219	V-->A
1Y8RA	216 - 220	219	V-->F
1Y8RA	216 - 220	219	V-->P
1Y8RA	216 - 220	219	V-->Q
1Y8RA	216 - 220	219	V-->R
1Y8RA	216 - 220	219	V-->T
1Y8RA	216 - 220	219	V-->Y
1Y8RA	216 - 220	220	R-->A
1Y8RA	216 - 220	220	R-->D
1Y8RA	216 - 220	220	R-->F
1Y8RA	216 - 220	220	R-->G

1Y8RA	216 - 220	220	R-->H
1Y8RA	216 - 220	220	R-->I
1Y8RA	216 - 220	220	R-->L
1Y8RA	216 - 220	220	R-->S
1Y8RA	216 - 220	220	R-->T
1Y8RA	216 - 220	220	R-->W
1Y8RA	216 - 220	220	R-->Y
2CEAB	355 - 360	357	I-->P
2CEAB	355 - 360	358	I-->E
2CEAB	355 - 360	358	I-->R
3BRJB	120 - 124	120	H-->A
3BRJB	120 - 124	120	H-->D
3BRJB	120 - 124	120	H-->E
3BRJB	120 - 124	120	H-->G
3BRJB	120 - 124	120	H-->I
3BRJB	120 - 124	120	H-->L
3BRJB	120 - 124	120	H-->M
3BRJB	120 - 124	120	H-->P
3BRJB	120 - 124	120	H-->Q
3BRJB	120 - 124	120	H-->S
3BRJB	120 - 124	120	H-->V
3BRJB	120 - 124	120	H-->W
3BRJB	120 - 124	120	H-->Y
3BRJB	120 - 124	121	L-->G
3BRJB	120 - 124	121	L-->Q
3BRJB	120 - 124	121	L-->R
3BRJB	120 - 124	122	V-->G

3BRJB	120	124	122	V-->P
3BRJB	120	124	122	V-->T
3BRJB	120	124	123	K-->H
3BRJB	120	124	123	K-->S
3BRJB	120	124	124	K-->A
3BRJB	120	124	124	K-->E
3BRJB	120	124	124	K-->F
3BRJB	120	124	124	K-->G
3BRJB	120	124	124	K-->L
3BRJB	120	124	124	K-->S
3BRJB	120	124	124	K-->V
1JC7A	7	12	11	E-->N
1JC7A	7	12	11	E-->S
1JC7A	7	12	11	E-->V
2CH6C	107	113	112	I-->R
2CH6C	210	215	210	K-->T
2CH6C	210	215	211	C-->A
2CH6C	210	215	214	A-->T
2CH6C	255	259	255	P-->E
2CH6C	255	259	255	P-->G
2CH6C	255	259	255	P-->K
2CH6C	255	259	255	P-->Q
2CH6C	255	259	255	P-->R
2CH6C	255	259	255	P-->T
2CH6C	255	259	256	V-->A
2CH6C	255	259	256	V-->D
2CH6C	255	259	256	V-->E

2CH6C	255 - 259	256	V-->I
2CH6C	255 - 259	256	V-->Q
2CH6C	255 - 259	256	V-->Y
2CH6C	255 - 259	257	L-->T
2CH6C	255 - 259	257	L-->V
2CH6C	255 - 259	258	F-->A
2CH6C	255 - 259	258	F-->D
2CH6C	255 - 259	258	F-->I
2CH6C	255 - 259	258	F-->L
2CH6C	255 - 259	258	F-->R
2CH6C	255 - 259	258	F-->S
2CH6C	255 - 259	259	Q-->C
2CH6C	255 - 259	259	Q-->F
2CH6C	255 - 259	259	Q-->G
2CH6C	255 - 259	259	Q-->I
2CH6C	255 - 259	259	Q-->L
2CH6C	255 - 259	259	Q-->M
2CH6C	255 - 259	259	Q-->N
2CH6C	255 - 259	259	Q-->P
2CH6C	255 - 259	259	Q-->S
2CH6C	255 - 259	259	Q-->T
2CH6C	255 - 259	259	Q-->V
1YDOA	62 - 68	62	A-->V
1YDOA	106 - 110	106	E-->C
1YDOA	106 - 110	106	E-->D
1YDOA	106 - 110	106	E-->N
1YDOA	106 - 110	106	E-->R

1YDOA	106 - 110	107	T-->Y
1YDOA	106 - 110	108	H-->L
1YDOA	106 - 110	108	H-->R
1YDOA	106 - 110	108	H-->W
1YDOA	106 - 110	109	N-->A
1YDOA	106 - 110	109	N-->F
1YDOA	106 - 110	109	N-->H
1YDOA	106 - 110	109	N-->R
1YDOA	106 - 110	110	R-->L
1YDOA	106 - 110	110	R-->P
1YDOA	106 - 110	110	R-->T
1YDOA	106 - 110	110	R-->V
1YDOA	220 - 224	220	V-->R
1YDOA	220 - 224	221	T-->A
1YDOA	220 - 224	221	T-->P
1YDOA	220 - 224	221	T-->Q
1YDOA	220 - 224	221	T-->S
1YDOA	220 - 224	222	A-->E
1YDOA	220 - 224	222	A-->F
1YDOA	220 - 224	222	A-->L
1YDOA	220 - 224	222	A-->N
1YDOA	220 - 224	222	A-->P
1YDOA	220 - 224	222	A-->R
1YDOA	220 - 224	222	A-->S
1YDOA	220 - 224	222	A-->T
1YDOA	220 - 224	222	A-->Y
1YDOA	220 - 224	223	L-->C

1YDOA	220	- 224	223	L-->D
1YDOA	220	- 224	223	L-->E
1YDOA	220	- 224	223	L-->G
1YDOA	220	- 224	223	L-->I
1YDOA	220	- 224	223	L-->T
1YDOA	220	- 224	223	L-->V
1YDOA	220	- 224	223	L-->Y
1YDOA	220	- 224	224	Q-->A
1YDOA	220	- 224	224	Q-->D
1YDOA	220	- 224	224	Q-->G
1YDOA	220	- 224	224	Q-->I
1YDOA	220	- 224	224	Q-->K
1YDOA	220	- 224	224	Q-->N
1YDOA	220	- 224	224	Q-->R
1YDOA	220	- 224	224	Q-->S
1YDOA	220	- 224	224	Q-->T
1YDOA	220	- 224	224	Q-->V
1YDOA	220	- 224	224	Q-->W
1YDOA	220	- 224	224	Q-->Y
1YDOA	252	- 257	253	E-->D
1YDOA	252	- 257	255	I-->S
1YDOA	291	- 296	291	R-->P
1YDOA	291	- 296	291	R-->S
1YDOA	291	- 296	293	L-->V
1YDOA	291	- 296	294	Q-->N
2F1VA	116	- 120	116	D-->E
2F1VA	116	- 120	116	D-->H

2F1VA	116 - 120	116	D-->I
2F1VA	116 - 120	116	D-->K
2F1VA	116 - 120	116	D-->P
2F1VA	116 - 120	116	D-->Q
2F1VA	116 - 120	116	D-->R
2F1VA	116 - 120	116	D-->S
2F1VA	116 - 120	117	H-->E
2F1VA	116 - 120	117	H-->G
2F1VA	116 - 120	117	H-->K
2F1VA	116 - 120	117	H-->L
2F1VA	116 - 120	117	H-->N
2F1VA	116 - 120	117	H-->P
2F1VA	116 - 120	117	H-->S
2F1VA	116 - 120	118	G-->K
2F1VA	116 - 120	118	G-->Q
2F1VA	116 - 120	118	G-->W
2F1VA	116 - 120	119	K-->D
2F1VA	116 - 120	119	K-->F
2F1VA	116 - 120	119	K-->R
2F1VA	116 - 120	119	K-->S
2F1VA	116 - 120	120	E-->D
2F1VA	116 - 120	120	E-->L
2F1VA	116 - 120	120	E-->Q
2F1VA	116 - 120	120	E-->Y
1I4LB	176 - 181	176	Y-->E
1I4LB	176 - 181	177	E-->F
1I4LB	176 - 181	177	E-->W

1I4LB	176 - 181	179	L-->Y	
1I4LB	176 - 181	180	R-->A	
1I4LB	176 - 181	181	G-->D	
1NSLC	47 - 52	47	A-->D	
1NSLC	47 - 52	50	Y-->V	
1NSLC	47 - 52	51	R-->L	
1NSLC	47 - 52	51	R-->V	
1VH3C	206 - 211	207	L-->P	
1VH3C	206 - 211	207	L-->V	
1VH3C	206 - 211	209	N-->P	
1VH3C	206 - 211	211	E-->R	
1VH3C	215 - 220	217	R-->M	
1VH3C	215 - 220	220	Y-->D	
1VH3C	215 - 220	220	Y-->L	
1VH3C	241 - 247	246	K-->R	
1HQ6A	2 - 8	8	N-->T	
2Z2SF	2 - 6	2	S-->A	
2Z2SF	2 - 6	2	S-->D	
2Z2SF	2 - 6	2	S-->E	
2Z2SF	2 - 6	2	S-->F	
2Z2SF	2 - 6	2	S-->G	
2Z2SF	2 - 6	2	S-->I	
2Z2SF	2 - 6	2	S-->K	
2Z2SF	2 - 6	2	S-->L	
2Z2SF	2 - 6	2	S-->N	
2Z2SF	2 - 6	2	S-->P	
2Z2SF	2 - 6	2	S-->Q	

2Z2SF	2 - 6	2	S-->R
2Z2SF	2 - 6	2	S-->T
2Z2SF	2 - 6	3	L-->A
2Z2SF	2 - 6	3	L-->G
2Z2SF	2 - 6	3	L-->I
2Z2SF	2 - 6	3	L-->N
2Z2SF	2 - 6	3	L-->P
2Z2SF	2 - 6	3	L-->Q
2Z2SF	2 - 6	3	L-->S
2Z2SF	2 - 6	3	L-->T
2Z2SF	2 - 6	3	L-->V
2Z2SF	2 - 6	4	A-->C
2Z2SF	2 - 6	4	A-->F
2Z2SF	2 - 6	4	A-->G
2Z2SF	2 - 6	4	A-->K
2Z2SF	2 - 6	4	A-->L
2Z2SF	2 - 6	4	A-->M
2Z2SF	2 - 6	4	A-->P
2Z2SF	2 - 6	4	A-->S
2Z2SF	2 - 6	4	A-->V
2Z2SF	2 - 6	4	A-->Y
2Z2SF	2 - 6	5	S-->A
2Z2SF	2 - 6	5	S-->D
2Z2SF	2 - 6	5	S-->E
2Z2SF	2 - 6	5	S-->F
2Z2SF	2 - 6	5	S-->G
2Z2SF	2 - 6	5	S-->I

2Z2SF	2 - 6	5	S-->K
2Z2SF	2 - 6	5	S-->L
2Z2SF	2 - 6	5	S-->M
2Z2SF	2 - 6	5	S-->P
2Z2SF	2 - 6	5	S-->Q
2Z2SF	2 - 6	5	S-->T
2Z2SF	2 - 6	5	S-->V
2Z2SF	2 - 6	6	V-->A
2Z2SF	2 - 6	6	V-->G
2Z2SF	2 - 6	6	V-->I
2Z2SF	2 - 6	6	V-->K
2Z2SF	2 - 6	6	V-->L
2Z2SF	2 - 6	6	V-->P
2Z2SF	2 - 6	6	V-->Q
2Z2SF	2 - 6	6	V-->R
2Z2SF	2 - 6	6	V-->S
2Z2SF	2 - 6	6	V-->T
1QZTA	2 - 6	2	V-->A
1QZTA	2 - 6	2	V-->D
1QZTA	2 - 6	2	V-->I
1QZTA	2 - 6	2	V-->K
1QZTA	2 - 6	2	V-->L
1QZTA	2 - 6	2	V-->M
1QZTA	2 - 6	2	V-->N
1QZTA	2 - 6	2	V-->P
1QZTA	2 - 6	2	V-->Q
1QZTA	2 - 6	2	V-->T

1QZTA	2 - 6	3	D-->A
1QZTA	2 - 6	3	D-->E
1QZTA	2 - 6	3	D-->G
1QZTA	2 - 6	3	D-->H
1QZTA	2 - 6	3	D-->I
1QZTA	2 - 6	3	D-->K
1QZTA	2 - 6	3	D-->L
1QZTA	2 - 6	3	D-->M
1QZTA	2 - 6	3	D-->N
1QZTA	2 - 6	3	D-->P
1QZTA	2 - 6	3	D-->R
1QZTA	2 - 6	3	D-->T
1QZTA	2 - 6	3	D-->V
1QZTA	2 - 6	4	A-->D
1QZTA	2 - 6	4	A-->E
1QZTA	2 - 6	4	A-->F
1QZTA	2 - 6	4	A-->G
1QZTA	2 - 6	4	A-->H
1QZTA	2 - 6	4	A-->K
1QZTA	2 - 6	4	A-->L
1QZTA	2 - 6	4	A-->N
1QZTA	2 - 6	4	A-->S
1QZTA	2 - 6	4	A-->T
1QZTA	2 - 6	4	A-->V
1QZTA	2 - 6	4	A-->Y
1QZTA	2 - 6	5	A-->D
1QZTA	2 - 6	5	A-->E

1QZTA	2	-	6	5	A-->F
1QZTA	2	-	6	5	A-->G
1QZTA	2	-	6	5	A-->H
1QZTA	2	-	6	5	A-->I
1QZTA	2	-	6	5	A-->K
1QZTA	2	-	6	5	A-->L
1QZTA	2	-	6	5	A-->M
1QZTA	2	-	6	5	A-->P
1QZTA	2	-	6	5	A-->R
1QZTA	2	-	6	5	A-->S
1QZTA	2	-	6	5	A-->V
1QZTA	2	-	6	5	A-->Y
1QZTA	2	-	6	6	I-->A
1QZTA	2	-	6	6	I-->D
1QZTA	2	-	6	6	I-->E
1QZTA	2	-	6	6	I-->F
1QZTA	2	-	6	6	I-->K
1QZTA	2	-	6	6	I-->L
1QZTA	2	-	6	6	I-->S
1QZTA	2	-	6	6	I-->T
1QZTA	2	-	6	6	I-->Y
1QZTA	251	-	257	254	A-->G
1DT9A	134	-	139	135	A-->D
1DT9A	204	-	208	204	K-->D
1DT9A	204	-	208	204	K-->E
1DT9A	204	-	208	204	K-->I
1DT9A	204	-	208	204	K-->L

1DT9A	204 - 208	204	K-->M
1DT9A	204 - 208	204	K-->N
1DT9A	204 - 208	204	K-->R
1DT9A	204 - 208	204	K-->V
1DT9A	204 - 208	205	V-->A
1DT9A	204 - 208	205	V-->E
1DT9A	204 - 208	205	V-->F
1DT9A	204 - 208	205	V-->L
1DT9A	204 - 208	205	V-->R
1DT9A	204 - 208	205	V-->T
1DT9A	204 - 208	206	A-->F
1DT9A	204 - 208	206	A-->G
1DT9A	204 - 208	206	A-->K
1DT9A	204 - 208	206	A-->Q
1DT9A	204 - 208	206	A-->R
1DT9A	204 - 208	206	A-->S
1DT9A	204 - 208	206	A-->T
1DT9A	204 - 208	206	A-->V
1DT9A	204 - 208	206	A-->Y
1DT9A	204 - 208	207	E-->A
1DT9A	204 - 208	207	E-->D
1DT9A	204 - 208	207	E-->F
1DT9A	204 - 208	207	E-->G
1DT9A	204 - 208	207	E-->I
1DT9A	204 - 208	207	E-->L
1DT9A	204 - 208	207	E-->M
1DT9A	204 - 208	207	E-->N

1DT9A	204 - 208	207	E-->S
1DT9A	204 - 208	207	E-->T
1DT9A	204 - 208	207	E-->V
1DT9A	204 - 208	207	E-->Y
1DT9A	204 - 208	208	T-->C
1DT9A	204 - 208	208	T-->G
1DT9A	204 - 208	208	T-->I
1DT9A	204 - 208	208	T-->L
1DT9A	204 - 208	208	T-->P
1DT9A	204 - 208	208	T-->Q
1DT9A	204 - 208	208	T-->S
1DT9A	204 - 208	208	T-->V
1TLTA	21 - 26	21	W-->I
1TLTA	21 - 26	21	W-->N
1TLTA	21 - 26	21	W-->Q
1TLTA	21 - 25	21	L-->A
1TLTA	21 - 25	21	L-->E
1TLTA	21 - 25	21	L-->F
1TLTA	21 - 25	21	L-->G
1TLTA	21 - 25	21	L-->H
1TLTA	21 - 25	21	L-->N
1TLTA	21 - 25	21	L-->P
1TLTA	21 - 25	21	L-->R
1TLTA	21 - 25	21	L-->T
1TLTA	21 - 25	21	L-->V
1TLTA	21 - 25	22	S-->A
1TLTA	21 - 25	22	S-->D

1TLTA	21 - 25	22	S-->E
1TLTA	21 - 25	22	S-->F
1TLTA	21 - 25	22	S-->G
1TLTA	21 - 25	22	S-->I
1TLTA	21 - 25	22	S-->N
1TLTA	21 - 25	22	S-->P
1TLTA	21 - 25	22	S-->R
1TLTA	21 - 25	23	S-->A
1TLTA	21 - 25	23	S-->C
1TLTA	21 - 25	23	S-->F
1TLTA	21 - 25	23	S-->G
1TLTA	21 - 25	23	S-->H
1TLTA	21 - 25	23	S-->K
1TLTA	21 - 25	23	S-->L
1TLTA	21 - 25	23	S-->N
1TLTA	21 - 25	23	S-->P
1TLTA	21 - 25	23	S-->V
1TLTA	21 - 25	24	L-->C
1TLTA	21 - 25	24	L-->D
1TLTA	21 - 25	24	L-->E
1TLTA	21 - 25	24	L-->G
1TLTA	21 - 25	24	L-->P
1TLTA	21 - 25	24	L-->Q
1TLTA	21 - 25	24	L-->R
1TLTA	21 - 25	24	L-->S
1TLTA	21 - 25	24	L-->T
1TLTA	21 - 25	24	L-->V

1TLTA	21 - 25	24	L-->W
1TLTA	21 - 25	25	A-->D
1TLTA	21 - 25	25	A-->F
1TLTA	21 - 25	25	A-->G
1TLTA	21 - 25	25	A-->I
1TLTA	21 - 25	25	A-->K
1TLTA	21 - 25	25	A-->N
1TLTA	21 - 25	25	A-->P
1TLTA	21 - 25	25	A-->Q
1TLTA	21 - 25	25	A-->R
1TLTA	21 - 25	25	A-->T
1TLTA	21 - 25	25	A-->V
1TLTA	129 - 134	130	L-->F
1TLTA	129 - 134	131	Y-->V
1TLTA	129 - 134	134	L-->F
1TLTA	159 - 165	163	L-->H
1LV2A	283 - 288	284	F-->R
1LV2A	283 - 288	285	G-->D
1LV2A	283 - 288	287	L-->P
1LV2A	283 - 288	288	L-->H
2D2AA	81 - 87	87	I-->K
2QAZA	81 - 85	81	Q-->A
2QAZA	81 - 85	81	Q-->C
2QAZA	81 - 85	81	Q-->L
2QAZA	81 - 85	81	Q-->N
2QAZA	81 - 85	81	Q-->P
2QAZA	81 - 85	81	Q-->S

2QAZA	81 - 85	81	Q-->Y
2QAZA	81 - 85	82	D-->A
2QAZA	81 - 85	82	D-->C
2QAZA	81 - 85	82	D-->L
2QAZA	81 - 85	82	D-->M
2QAZA	81 - 85	82	D-->V
2QAZA	81 - 85	83	L-->H
2QAZA	81 - 85	83	L-->I
2QAZA	81 - 85	83	L-->K
2QAZA	81 - 85	83	L-->N
2QAZA	81 - 85	83	L-->S
2QAZA	81 - 85	83	L-->T
2QAZA	81 - 85	84	L-->F
2QAZA	81 - 85	84	L-->G
2QAZA	81 - 85	84	L-->V
2QAZA	81 - 85	85	S-->A
2QAZA	81 - 85	85	S-->D
2QAZA	81 - 85	85	S-->F
2QAZA	81 - 85	85	S-->P
2QAZA	81 - 85	85	S-->T
1XHMA	81 - 85	81	L-->A
1XHMA	81 - 85	81	L-->G
1XHMA	81 - 85	81	L-->N
1XHMA	81 - 85	81	L-->Q
1XHMA	81 - 85	81	L-->R
1XHMA	81 - 85	81	L-->S
1XHMA	81 - 85	81	L-->T

1XHMA	81 - 85	81	L-->Y
1XHMA	81 - 85	82	S-->D
1XHMA	81 - 85	82	S-->F
1XHMA	81 - 85	82	S-->G
1XHMA	81 - 85	82	S-->H
1XHMA	81 - 85	82	S-->N
1XHMA	81 - 85	82	S-->R
1XHMA	81 - 85	82	S-->T
1XHMA	81 - 85	83	Q-->A
1XHMA	81 - 85	83	Q-->C
1XHMA	81 - 85	83	Q-->D
1XHMA	81 - 85	83	Q-->E
1XHMA	81 - 85	83	Q-->G
1XHMA	81 - 85	83	Q-->H
1XHMA	81 - 85	83	Q-->I
1XHMA	81 - 85	83	Q-->K
1XHMA	81 - 85	83	Q-->N
1XHMA	81 - 85	83	Q-->P
1XHMA	81 - 85	83	Q-->Y
1XHMA	81 - 85	84	I-->A
1XHMA	81 - 85	84	I-->D
1XHMA	81 - 85	84	I-->G
1XHMA	81 - 85	84	I-->P
1XHMA	81 - 85	84	I-->R
1XHMA	81 - 85	84	I-->Y
1XHMA	81 - 85	85	T-->A
1XHMA	81 - 85	85	T-->K

1XHMA	81	85	85	T-->L
1XHMA	81	85	85	T-->V
1ZUNA	37	41	37	K-->A
1ZUNA	37	41	37	K-->E
1ZUNA	37	41	37	K-->G
1ZUNA	37	41	37	K-->L
1ZUNA	37	41	37	K-->P
1ZUNA	37	41	37	K-->S
1ZUNA	37	41	37	K-->V
1ZUNA	37	41	38	D-->A
1ZUNA	37	41	38	D-->E
1ZUNA	37	41	38	D-->F
1ZUNA	37	41	38	D-->G
1ZUNA	37	41	38	D-->L
1ZUNA	37	41	38	D-->P
1ZUNA	37	41	38	D-->R
1ZUNA	37	41	38	D-->T
1ZUNA	37	41	38	D-->V
1ZUNA	37	41	38	D-->Y
1ZUNA	37	41	39	S-->A
1ZUNA	37	41	39	S-->C
1ZUNA	37	41	39	S-->D
1ZUNA	37	41	39	S-->G
1ZUNA	37	41	39	S-->I
1ZUNA	37	41	39	S-->K
1ZUNA	37	41	39	S-->L
1ZUNA	37	41	39	S-->N

1ZUNA	37 - 41	39	S-->P
1ZUNA	37 - 41	39	S-->T
1ZUNA	37 - 41	40	A-->D
1ZUNA	37 - 41	40	A-->K
1ZUNA	37 - 41	40	A-->L
1ZUNA	37 - 41	40	A-->N
1ZUNA	37 - 41	40	A-->P
1ZUNA	37 - 41	40	A-->R
1ZUNA	37 - 41	40	A-->T
1ZUNA	37 - 41	40	A-->Y
1ZUNA	37 - 41	41	V-->A
1ZUNA	37 - 41	41	V-->E
1ZUNA	37 - 41	41	V-->G
1ZUNA	37 - 41	41	V-->H
1ZUNA	37 - 41	41	V-->I
1ZUNA	37 - 41	41	V-->L
1ZUNA	37 - 41	41	V-->N
1ZUNA	37 - 41	41	V-->Q
1ZUNA	37 - 41	41	V-->R
1ZUNA	37 - 41	41	V-->T
1ZUNA	37 - 41	41	V-->Y
1ZUNA	72 - 77	72	Y-->H
1ZUNA	108 - 112	108	K-->A
1ZUNA	108 - 112	108	K-->C
1ZUNA	108 - 112	108	K-->I
1ZUNA	108 - 112	108	K-->S
1ZUNA	108 - 112	108	K-->V

1ZUNA	108 - 112	109	H-->D
1ZUNA	108 - 112	109	H-->E
1ZUNA	108 - 112	109	H-->G
1ZUNA	108 - 112	109	H-->I
1ZUNA	108 - 112	109	H-->K
1ZUNA	108 - 112	109	H-->L
1ZUNA	108 - 112	109	H-->N
1ZUNA	108 - 112	109	H-->R
1ZUNA	108 - 112	110	T-->S
1ZUNA	108 - 112	110	T-->V
1ZUNA	108 - 112	111	D-->A
1ZUNA	108 - 112	112	I-->G
1ZUNA	108 - 112	112	I-->Y
1ZUNA	140 - 144	140	K-->F
1ZUNA	140 - 144	140	K-->I
1ZUNA	140 - 144	140	K-->V
1ZUNA	140 - 144	141	S-->A
1ZUNA	140 - 144	141	S-->C
1ZUNA	140 - 144	141	S-->D
1ZUNA	140 - 144	141	S-->E
1ZUNA	140 - 144	141	S-->F
1ZUNA	140 - 144	141	S-->G
1ZUNA	140 - 144	141	S-->L
1ZUNA	140 - 144	141	S-->M
1ZUNA	140 - 144	141	S-->R
1ZUNA	140 - 144	141	S-->T
1ZUNA	140 - 144	142	R-->A

1ZUNA	140	144	142	R-->K
1ZUNA	140	144	142	R-->N
1ZUNA	140	144	142	R-->S
1ZUNA	140	144	142	R-->T
1ZUNA	140	144	142	R-->V
1ZUNA	140	144	143	A-->F
1ZUNA	140	144	143	A-->L
1ZUNA	140	144	143	A-->S
1ZUNA	140	144	143	A-->V
1ZUNA	140	144	144	K-->A
1ZUNA	140	144	144	K-->N
1ZUNA	140	144	144	K-->T
1ZUNA	140	144	144	K-->W
2HJ0A	12	17	13	K-->T
2HJ0A	12	17	15	A-->P
2HJ0A	109	114	114	K-->E
2HJ0A	195	199	195	I-->C
2HJ0A	195	199	195	I-->E
2HJ0A	195	199	195	I-->K
2HJ0A	195	199	195	I-->L
2HJ0A	195	199	195	I-->V
2HJ0A	195	199	195	I-->Y
2HJ0A	195	199	196	D-->K
2HJ0A	195	199	196	D-->S
2HJ0A	195	199	197	A-->F
2HJ0A	195	199	197	A-->G
2HJ0A	195	199	197	A-->Q

2HJ0A	195 - 199	197	A-->T
2HJ0A	195 - 199	197	A-->Y
2HJ0A	195 - 199	198	K-->A
2HJ0A	195 - 199	198	K-->D
2HJ0A	195 - 199	198	K-->G
2HJ0A	195 - 199	198	K-->L
2HJ0A	195 - 199	198	K-->V
2HJ0A	195 - 199	199	Y-->C
2HJ0A	195 - 199	199	Y-->D
2HJ0A	195 - 199	199	Y-->E
2HJ0A	195 - 199	199	Y-->F
2HJ0A	195 - 199	199	Y-->G
2HJ0A	195 - 199	199	Y-->P
2HJ0A	195 - 199	199	Y-->Q
2HJ0A	195 - 199	199	Y-->R
2HJ0A	195 - 199	199	Y-->S
2HJ0A	195 - 199	199	Y-->T
2HJ0A	195 - 199	199	Y-->V
2HJ0A	195 - 199	199	Y-->W
2HJ0A	195 - 199	195	V-->A
2HJ0A	195 - 199	195	V-->C
2HJ0A	195 - 199	195	V-->D
2HJ0A	195 - 199	195	V-->F
2HJ0A	195 - 199	195	V-->G
2HJ0A	195 - 199	195	V-->K
2HJ0A	195 - 199	195	V-->L
2HJ0A	195 - 199	195	V-->N

2HJ0A	195 - 199	195	V-->P
2HJ0A	195 - 199	195	V-->Q
2HJ0A	195 - 199	195	V-->S
2HJ0A	195 - 199	196	E-->A
2HJ0A	195 - 199	196	E-->D
2HJ0A	195 - 199	196	E-->H
2HJ0A	195 - 199	196	E-->I
2HJ0A	195 - 199	196	E-->K
2HJ0A	195 - 199	196	E-->L
2HJ0A	195 - 199	196	E-->N
2HJ0A	195 - 199	196	E-->Q
2HJ0A	195 - 199	196	E-->R
2HJ0A	195 - 199	196	E-->S
2HJ0A	195 - 199	196	E-->T
2HJ0A	195 - 199	196	E-->V
2HJ0A	195 - 199	196	E-->W
2HJ0A	195 - 199	196	E-->Y
2HJ0A	195 - 199	197	L-->D
2HJ0A	195 - 199	197	L-->E
2HJ0A	195 - 199	197	L-->G
2HJ0A	195 - 199	197	L-->I
2HJ0A	195 - 199	197	L-->M
2HJ0A	195 - 199	197	L-->N
2HJ0A	195 - 199	197	L-->Q
2HJ0A	195 - 199	197	L-->T
2HJ0A	195 - 199	197	L-->V
2HJ0A	195 - 199	198	L-->D

2HJ0A	195 - 199	198	L-->E
2HJ0A	195 - 199	198	L-->F
2HJ0A	195 - 199	198	L-->G
2HJ0A	195 - 199	198	L-->I
2HJ0A	195 - 199	198	L-->K
2HJ0A	195 - 199	198	L-->P
2HJ0A	195 - 199	198	L-->Q
2HJ0A	195 - 199	198	L-->R
2HJ0A	195 - 199	198	L-->T
2HJ0A	195 - 199	198	L-->V
2HJ0A	195 - 199	198	L-->W
2HJ0A	195 - 199	199	E-->A
2HJ0A	195 - 199	199	E-->D
2HJ0A	195 - 199	199	E-->F
2HJ0A	195 - 199	199	E-->G
2HJ0A	195 - 199	199	E-->H
2HJ0A	195 - 199	199	E-->I
2HJ0A	195 - 199	199	E-->K
2HJ0A	195 - 199	199	E-->N
2HJ0A	195 - 199	199	E-->P
2HJ0A	195 - 199	199	E-->T
2HJ0A	195 - 199	199	E-->Y
2HJ0A	454 - 459	455	D-->G
2HJ0A	454 - 459	456	L-->E
2HJ0A	454 - 459	456	L-->S
2HJ0A	454 - 459	459	Y-->E
1RI2A	143 - 147	143	F-->L

1RI2A	143	147	143	F-->T
1RI2A	143	147	144	H-->G
1RI2A	143	147	144	H-->N
1RI2A	143	147	145	Y-->G
1RI2A	143	147	145	Y-->H
1RI2A	143	147	145	Y-->K
1RI2A	143	147	145	Y-->N
1RI2A	143	147	145	Y-->P
1RI2A	143	147	146	A-->V
1RI2A	143	147	147	F-->C
1RI2A	143	147	147	F-->G
1RI2A	143	147	147	F-->K
1RI2A	143	147	147	F-->N
1RI2A	275	280	275	R-->N
1RI2A	275	280	275	R-->Q
1RI2A	275	280	276	E-->R
2EWJA	152	156	152	T-->A
2EWJA	152	156	152	T-->G
2EWJA	152	156	152	T-->H
2EWJA	152	156	152	T-->S
2EWJA	152	156	152	T-->V
2EWJA	152	156	153	L-->A
2EWJA	152	156	153	L-->H
2EWJA	152	156	153	L-->K
2EWJA	152	156	153	L-->N
2EWJA	152	156	153	L-->V
2EWJA	152	156	153	L-->Y

2EWJA	152 - 156	154	N-->D
2EWJA	152 - 156	154	N-->K
2EWJA	152 - 156	154	N-->P
2EWJA	152 - 156	154	N-->S
2EWJA	152 - 156	155	A-->F
2EWJA	152 - 156	155	A-->L
2EWJA	152 - 156	155	A-->M
2EWJA	152 - 156	155	A-->N
2EWJA	152 - 156	155	A-->P
2EWJA	152 - 156	155	A-->Q
2EWJA	152 - 156	155	A-->R
2EWJA	152 - 156	155	A-->S
2EWJA	152 - 156	155	A-->T
2EWJA	152 - 156	155	A-->V
2EWJA	152 - 156	156	Y-->D
2EWJA	152 - 156	156	Y-->G
2EWJA	152 - 156	156	Y-->R
2EWJA	152 - 156	156	Y-->S
2EWJA	152 - 156	156	Y-->T
2EWJA	152 - 156	156	Y-->V
1I7FA	199 - 204	199	T-->P
1I7FA	199 - 204	200	E-->G
1I7FA	199 - 204	200	E-->L
1I7FA	199 - 204	200	E-->P
1I7FA	199 - 204	200	E-->V
1I7FA	199 - 204	201	E-->P
1I7FA	199 - 204	202	L-->N

1I7FA	199	204	203	L-->R
2VP1A	47	52	48	D-->V
2VP1A	106	111	107	K-->H
2VP1A	327	331	327	S-->A
2VP1A	327	331	327	S-->F
2VP1A	327	331	327	S-->I
2VP1A	327	331	327	S-->K
2VP1A	327	331	327	S-->L
2VP1A	327	331	327	S-->Q
2VP1A	327	331	327	S-->T
2VP1A	327	331	327	S-->V
2VP1A	327	331	328	N-->A
2VP1A	327	331	328	N-->E
2VP1A	327	331	328	N-->F
2VP1A	327	331	328	N-->H
2VP1A	327	331	328	N-->I
2VP1A	327	331	328	N-->L
2VP1A	327	331	328	N-->M
2VP1A	327	331	328	N-->Q
2VP1A	327	331	328	N-->R
2VP1A	327	331	328	N-->S
2VP1A	327	331	328	N-->T
2VP1A	327	331	328	N-->V
2VP1A	327	331	328	N-->W
2VP1A	327	331	328	N-->Y
2VP1A	327	331	329	L-->A
2VP1A	327	331	329	L-->D

2VP1A	327	-	331	329	L-->S
2VP1A	327	-	331	329	L-->T
2VP1A	327	-	331	329	L-->V
2VP1A	327	-	331	330	G-->A
2VP1A	327	-	331	330	G-->D
2VP1A	327	-	331	330	G-->I
2VP1A	327	-	331	330	G-->K
2VP1A	327	-	331	330	G-->P
2VP1A	327	-	331	330	G-->R
2VP1A	327	-	331	330	G-->V
2VP1A	327	-	331	331	R-->F
2VP1A	327	-	331	331	R-->G
2VP1A	327	-	331	331	R-->I
2VP1A	327	-	331	331	R-->K
2VP1A	327	-	331	331	R-->P
2VP1A	327	-	331	331	R-->V
2P9QA	395	-	402	400	E-->V
1VOLA	177	-	181	177	F-->A
1VOLA	177	-	181	177	F-->P
1VOLA	177	-	181	177	F-->V
1VOLA	177	-	181	180	I-->N
1VOLA	177	-	181	180	I-->T
1VOLA	177	-	181	180	I-->Y
1VOLA	177	-	181	181	C-->L
1VOLA	177	-	181	181	C-->R
1VOLA	177	-	181	181	C-->V
2I46A	99	-	104	101	E-->F

2I46A	159 - 163	159	R-->A
2I46A	159 - 163	159	R-->E
2I46A	159 - 163	159	R-->F
2I46A	159 - 163	159	R-->H
2I46A	159 - 163	159	R-->I
2I46A	159 - 163	159	R-->K
2I46A	159 - 163	159	R-->L
2I46A	159 - 163	159	R-->S
2I46A	159 - 163	159	R-->Y
2I46A	159 - 163	160	E-->A
2I46A	159 - 163	160	E-->C
2I46A	159 - 163	160	E-->G
2I46A	159 - 163	160	E-->I
2I46A	159 - 163	160	E-->L
2I46A	159 - 163	160	E-->P
2I46A	159 - 163	160	E-->R
2I46A	159 - 163	160	E-->T
2I46A	159 - 163	160	E-->W
2I46A	159 - 163	161	A-->D
2I46A	159 - 163	161	A-->F
2I46A	159 - 163	161	A-->G
2I46A	159 - 163	161	A-->I
2I46A	159 - 163	161	A-->N
2I46A	159 - 163	161	A-->P
2I46A	159 - 163	161	A-->R
2I46A	159 - 163	161	A-->S
2I46A	159 - 163	161	A-->V

2I46A	159 - 163	161	A-->Y
2I46A	159 - 163	162	L-->F
2I46A	159 - 163	162	L-->G
2I46A	159 - 163	162	L-->S
2I46A	159 - 163	162	L-->V
2I46A	159 - 163	163	D-->A
2I46A	159 - 163	163	D-->E
2I46A	159 - 163	163	D-->K
2I46A	159 - 163	163	D-->L
2I46A	159 - 163	163	D-->N
2I46A	159 - 163	163	D-->P
2I46A	159 - 163	163	D-->S
2I46A	159 - 163	163	D-->T
1ON7A	146 - 150	146	G-->F
1ON7A	146 - 150	146	G-->I
1ON7A	146 - 150	146	G-->K
1ON7A	146 - 150	146	G-->N
1ON7A	146 - 150	146	G-->P
1ON7A	146 - 150	146	G-->Q
1ON7A	146 - 150	146	G-->S
1ON7A	146 - 150	146	G-->V
1ON7A	146 - 150	147	Q-->A
1ON7A	146 - 150	147	Q-->C
1ON7A	146 - 150	147	Q-->D
1ON7A	146 - 150	147	Q-->E
1ON7A	146 - 150	147	Q-->F
1ON7A	146 - 150	147	Q-->G

1ON7A	146 - 150	147	Q-->H
1ON7A	146 - 150	147	Q-->I
1ON7A	146 - 150	147	Q-->L
1ON7A	146 - 150	147	Q-->M
1ON7A	146 - 150	147	Q-->N
1ON7A	146 - 150	147	Q-->P
1ON7A	146 - 150	147	Q-->R
1ON7A	146 - 150	147	Q-->S
1ON7A	146 - 150	147	Q-->Y
1ON7A	146 - 150	148	A-->C
1ON7A	146 - 150	148	A-->D
1ON7A	146 - 150	148	A-->E
1ON7A	146 - 150	148	A-->F
1ON7A	146 - 150	148	A-->G
1ON7A	146 - 150	148	A-->L
1ON7A	146 - 150	148	A-->N
1ON7A	146 - 150	148	A-->P
1ON7A	146 - 150	148	A-->R
1ON7A	146 - 150	148	A-->T
1ON7A	146 - 150	148	A-->V
1ON7A	146 - 150	148	A-->Y
1ON7A	146 - 150	149	A-->F
1ON7A	146 - 150	149	A-->H
1ON7A	146 - 150	149	A-->K
1ON7A	146 - 150	149	A-->L
1ON7A	146 - 150	149	A-->M
1ON7A	146 - 150	149	A-->N

1ON7A	146	- 150	149	A-->P
1ON7A	146	- 150	149	A-->T
1ON7A	146	- 150	149	A-->V
1ON7A	146	- 150	149	A-->Y
1ON7A	146	- 150	150	V-->C
1ON7A	146	- 150	150	V-->D
1ON7A	146	- 150	150	V-->F
1ON7A	146	- 150	150	V-->G
1ON7A	146	- 150	150	V-->K
1ON7A	146	- 150	150	V-->L
1ON7A	146	- 150	150	V-->P
1ON7A	146	- 150	150	V-->S
1ON7A	146	- 150	150	V-->W
1ECBA	210	- 215	210	S-->A
1ECBA	210	- 215	210	S-->E
1ECBA	210	- 215	210	S-->V
1ECBA	210	- 215	211	V-->W
1ECBA	210	- 215	212	A-->T
1ECBA	210	- 215	213	L-->N
1ECBA	210	- 215	213	L-->P
1ECBA	210	- 215	213	L-->Q
1ECBA	210	- 215	215	T-->A
1ECBA	253	- 257	253	L-->G
1ECBA	253	- 257	253	L-->I
1ECBA	253	- 257	253	L-->N
1ECBA	253	- 257	254	F-->A
1ECBA	253	- 257	254	F-->G

1ECBA	253 - 257	254	F-->H
1ECBA	253 - 257	254	F-->L
1ECBA	253 - 257	254	F-->P
1ECBA	253 - 257	254	F-->T
1ECBA	253 - 257	255	E-->D
1ECBA	253 - 257	255	E-->G
1ECBA	253 - 257	255	E-->T
1ECBA	253 - 257	255	E-->W
1ECBA	253 - 257	256	Y-->E
1ECBA	253 - 257	256	Y-->G
1ECBA	253 - 257	256	Y-->I
1ECBA	253 - 257	256	Y-->P
1ECBA	253 - 257	256	Y-->Q
1ECBA	253 - 257	256	Y-->R
1ECBA	253 - 257	256	Y-->T
1ECBA	253 - 257	257	V-->A
1ECBA	253 - 257	257	V-->M
1ECBA	253 - 257	257	V-->N
1VK9A	82 - 87	84	A-->D
1VK9A	82 - 87	86	K-->E
1VK9A	82 - 87	86	K-->R
1VK9A	82 - 87	87	L-->E
1VK9A	82 - 87	87	L-->P
1VK9A	114 - 120	115	F-->S
2C20A	80 - 85	80	V-->A
2C20A	80 - 85	80	V-->H
2C20A	80 - 85	81	G-->N

2C20A	80	- 85	83	S-->E
2C20A	80	- 85	84	M-->A
2C20A	80	- 85	84	M-->G
2QL6A	68	- 73	72	C-->G
2QL6A	68	- 73	72	C-->S
2QL6A	151	- 156	153	K-->Q
2QL6A	151	- 156	154	Y-->M
2INNE	50	- 56	52	A-->Q
2INNE	58	- 62	58	L-->A
2INNE	58	- 62	58	L-->C
2INNE	58	- 62	58	L-->G
2INNE	58	- 62	58	L-->I
2INNE	58	- 62	58	L-->N
2INNE	58	- 62	58	L-->P
2INNE	58	- 62	58	L-->R
2INNE	58	- 62	58	L-->S
2INNE	58	- 62	58	L-->V
2INNE	58	- 62	58	L-->W
2INNE	58	- 62	59	K-->A
2INNE	58	- 62	59	K-->D
2INNE	58	- 62	59	K-->F
2INNE	58	- 62	59	K-->G
2INNE	58	- 62	59	K-->H
2INNE	58	- 62	59	K-->L
2INNE	58	- 62	59	K-->N
2INNE	58	- 62	59	K-->Q
2INNE	58	- 62	59	K-->R

2INNE	58 - 62	59	K-->S
2INNE	58 - 62	59	K-->Y
2INNE	58 - 62	60	P-->A
2INNE	58 - 62	60	P-->D
2INNE	58 - 62	60	P-->I
2INNE	58 - 62	60	P-->K
2INNE	58 - 62	60	P-->L
2INNE	58 - 62	60	P-->N
2INNE	58 - 62	60	P-->Q
2INNE	58 - 62	60	P-->T
2INNE	58 - 62	60	P-->V
2INNE	58 - 62	61	A-->D
2INNE	58 - 62	61	A-->E
2INNE	58 - 62	61	A-->F
2INNE	58 - 62	61	A-->G
2INNE	58 - 62	61	A-->K
2INNE	58 - 62	61	A-->L
2INNE	58 - 62	61	A-->N
2INNE	58 - 62	61	A-->P
2INNE	58 - 62	61	A-->R
2INNE	58 - 62	61	A-->S
2INNE	58 - 62	61	A-->V
2INNE	58 - 62	61	A-->Y
2INNE	58 - 62	62	T-->A
2INNE	58 - 62	62	T-->D
2INNE	58 - 62	62	T-->F
2INNE	58 - 62	62	T-->G

2INNE	58 - 62	62	T-->I
2INNE	58 - 62	62	T-->K
2INNE	58 - 62	62	T-->L
2INNE	58 - 62	62	T-->P
2INNE	58 - 62	62	T-->R
2INNE	58 - 62	62	T-->S
2INNE	58 - 62	62	T-->V
1IPKB	41 - 46	42	Q-->P
1IPKB	41 - 46	42	Q-->S
1IPKB	41 - 46	43	L-->V
1IPKB	41 - 46	45	N-->S
1IPKB	192 - 197	196	R-->K
1IPKB	242 - 247	242	P-->S
1IPKB	242 - 247	245	R-->C
2Q80B	8 - 12	8	V-->D
2Q80B	8 - 12	8	V-->E
2Q80B	8 - 12	8	V-->H
2Q80B	8 - 12	8	V-->T
2Q80B	8 - 12	9	Q-->E
2Q80B	8 - 12	9	Q-->G
2Q80B	8 - 12	9	Q-->H
2Q80B	8 - 12	9	Q-->L
2Q80B	8 - 12	9	Q-->P
2Q80B	8 - 12	9	Q-->T
2Q80B	8 - 12	9	Q-->V
2Q80B	8 - 12	9	Q-->Y
2Q80B	8 - 12	10	R-->E

2Q80B	8 - 12	10	R-->G
2Q80B	8 - 12	10	R-->H
2Q80B	8 - 12	10	R-->L
2Q80B	8 - 12	10	R-->M
2Q80B	8 - 12	10	R-->P
2Q80B	8 - 12	10	R-->V
2Q80B	8 - 12	11	I-->C
2Q80B	8 - 12	11	I-->D
2Q80B	8 - 12	11	I-->G
2Q80B	8 - 12	11	I-->K
2Q80B	8 - 12	11	I-->L
2Q80B	8 - 12	11	I-->P
2Q80B	8 - 12	12	L-->E
2Q80B	8 - 12	12	L-->F
2Q80B	8 - 12	12	L-->I
2Q80B	8 - 12	12	L-->K
2Q80B	8 - 12	12	L-->P
2Q80B	8 - 12	12	L-->T
2Q80B	8 - 12	12	L-->W
2Q80B	14 - 19	16	Y-->A
2Q80B	79 - 83	79	A-->E
2Q80B	79 - 83	79	A-->S
2Q80B	79 - 83	80	H-->A
2Q80B	79 - 83	80	H-->D
2Q80B	79 - 83	80	H-->F
2Q80B	79 - 83	80	H-->N
2Q80B	79 - 83	80	H-->S

2Q80B	79 - 83	81	S-->H
2Q80B	79 - 83	81	S-->I
2Q80B	79 - 83	81	S-->V
2Q80B	79 - 83	82	I-->D
2Q80B	79 - 83	82	I-->F
2Q80B	79 - 83	82	I-->G
2Q80B	79 - 83	82	I-->M
2Q80B	79 - 83	83	Y-->H
2Q80B	79 - 83	83	Y-->T
2Q80B	79 - 83	83	Y-->V
2Q80B	206 - 210	206	E-->A
2Q80B	206 - 210	206	E-->G
2Q80B	206 - 210	206	E-->I
2Q80B	206 - 210	206	E-->K
2Q80B	206 - 210	206	E-->N
2Q80B	206 - 210	206	E-->P
2Q80B	206 - 210	206	E-->V
2Q80B	206 - 210	207	D-->F
2Q80B	206 - 210	207	D-->G
2Q80B	206 - 210	207	D-->K
2Q80B	206 - 210	207	D-->L
2Q80B	206 - 210	207	D-->N
2Q80B	206 - 210	207	D-->P
2Q80B	206 - 210	207	D-->S
2Q80B	206 - 210	207	D-->T
2Q80B	206 - 210	208	L-->C
2Q80B	206 - 210	208	L-->D

2Q80B	206 - 210	208	L-->G
2Q80B	206 - 210	208	L-->H
2Q80B	206 - 210	208	L-->I
2Q80B	206 - 210	208	L-->K
2Q80B	206 - 210	208	L-->T
2Q80B	206 - 210	209	T-->A
2Q80B	206 - 210	209	T-->F
2Q80B	206 - 210	209	T-->G
2Q80B	206 - 210	209	T-->I
2Q80B	206 - 210	209	T-->L
2Q80B	206 - 210	209	T-->N
2Q80B	206 - 210	209	T-->P
2Q80B	206 - 210	210	E-->A
2Q80B	206 - 210	210	E-->C
2Q80B	206 - 210	210	E-->F
2Q80B	206 - 210	210	E-->G
2Q80B	206 - 210	210	E-->H
2Q80B	206 - 210	210	E-->L
2Q80B	206 - 210	210	E-->P
2Q80B	206 - 210	210	E-->Q
2Q80B	206 - 210	210	E-->S
2Q80B	206 - 210	210	E-->V
2Q80B	206 - 210	210	E-->W
1ANVA	199 - 203	199	N-->A
1ANVA	199 - 203	199	N-->D
1ANVA	199 - 203	199	N-->E
1ANVA	199 - 203	199	N-->G

1ANVA	199 - 203	199	N-->P
1ANVA	199 - 203	199	N-->S
1ANVA	199 - 203	199	N-->T
1ANVA	199 - 203	199	N-->V
1ANVA	199 - 203	199	N-->Y
1ANVA	199 - 203	200	D-->A
1ANVA	199 - 203	200	D-->E
1ANVA	199 - 203	200	D-->L
1ANVA	199 - 203	200	D-->N
1ANVA	199 - 203	200	D-->T
1ANVA	199 - 203	201	L-->A
1ANVA	199 - 203	201	L-->G
1ANVA	199 - 203	201	L-->Q
1ANVA	199 - 203	201	L-->S
1ANVA	199 - 203	202	K-->D
1ANVA	199 - 203	202	K-->I
1ANVA	199 - 203	202	K-->L
1ANVA	199 - 203	202	K-->N
1ANVA	199 - 203	202	K-->P
1ANVA	199 - 203	202	K-->S
1ANVA	199 - 203	202	K-->T
1ANVA	199 - 203	202	K-->V
1ANVA	199 - 203	202	K-->Y
1ANVA	199 - 203	203	A-->D
1ANVA	199 - 203	203	A-->E
1ANVA	199 - 203	203	A-->H
1ANVA	199 - 203	203	A-->I

1ANVA	199 - 203	203	A-->P
1ANVA	199 - 203	203	A-->T
1ANVA	199 - 203	203	A-->V
3ZNBB	108 - 113	108	L-->K
3ZNBB	108 - 113	112	Q-->L
2B30A	270 - 275	270	D-->R
2DX8A	391 - 395	392	T-->D
2DX8A	391 - 395	393	C-->G
2DX8A	391 - 395	393	C-->I
2DX8A	391 - 395	394	M-->A
2DX8A	391 - 395	394	M-->G
2DX8A	391 - 395	394	M-->I
2DX8A	391 - 395	394	M-->T
2DX8A	391 - 395	395	A-->G
1EXCA	154 - 158	154	G-->A
1EXCA	154 - 158	154	G-->D
1EXCA	154 - 158	154	G-->E
1EXCA	154 - 158	154	G-->F
1EXCA	154 - 158	154	G-->L
1EXCA	154 - 158	154	G-->N
1EXCA	154 - 158	154	G-->P
1EXCA	154 - 158	154	G-->R
1EXCA	154 - 158	154	G-->S
1EXCA	154 - 158	154	G-->T
1EXCA	154 - 158	154	G-->V
1EXCA	154 - 158	154	G-->Y
1EXCA	154 - 158	155	R-->A

1EXCA	154 - 158	155	R-->C
1EXCA	154 - 158	155	R-->E
1EXCA	154 - 158	155	R-->F
1EXCA	154 - 158	155	R-->G
1EXCA	154 - 158	155	R-->H
1EXCA	154 - 158	155	R-->I
1EXCA	154 - 158	155	R-->K
1EXCA	154 - 158	155	R-->L
1EXCA	154 - 158	155	R-->M
1EXCA	154 - 158	155	R-->N
1EXCA	154 - 158	155	R-->P
1EXCA	154 - 158	155	R-->Q
1EXCA	154 - 158	155	R-->S
1EXCA	154 - 158	155	R-->T
1EXCA	154 - 158	155	R-->V
1EXCA	154 - 158	155	R-->W
1EXCA	154 - 158	156	G-->A
1EXCA	154 - 158	156	G-->D
1EXCA	154 - 158	156	G-->F
1EXCA	154 - 158	156	G-->I
1EXCA	154 - 158	156	G-->L
1EXCA	154 - 158	156	G-->P
1EXCA	154 - 158	156	G-->Q
1EXCA	154 - 158	156	G-->R
1EXCA	154 - 158	156	G-->S
1EXCA	154 - 158	156	G-->V
1EXCA	154 - 158	157	A-->D

1EXCA	154 - 158	157	A-->E
1EXCA	154 - 158	157	A-->G
1EXCA	154 - 158	157	A-->I
1EXCA	154 - 158	157	A-->K
1EXCA	154 - 158	157	A-->L
1EXCA	154 - 158	157	A-->P
1EXCA	154 - 158	157	A-->Q
1EXCA	154 - 158	157	A-->T
1EXCA	154 - 158	157	A-->V
1EXCA	154 - 158	158	L-->A
1EXCA	154 - 158	158	L-->D
1EXCA	154 - 158	158	L-->E
1EXCA	154 - 158	158	L-->F
1EXCA	154 - 158	158	L-->G
1EXCA	154 - 158	158	L-->H
1EXCA	154 - 158	158	L-->I
1EXCA	154 - 158	158	L-->K
1EXCA	154 - 158	158	L-->M
1EXCA	154 - 158	158	L-->N
1EXCA	154 - 158	158	L-->P
1EXCA	154 - 158	158	L-->R
1EXCA	154 - 158	158	L-->V
1EXCA	154 - 158	158	L-->Y
1PEXA	460 - 464	460	A-->D
1PEXA	460 - 464	460	A-->E
1PEXA	460 - 464	460	A-->G
1PEXA	460 - 464	460	A-->K

1PEXA	460 - 464	460	A-->L
1PEXA	460 - 464	460	A-->P
1PEXA	460 - 464	460	A-->S
1PEXA	460 - 464	460	A-->T
1PEXA	460 - 464	461	N-->E
1PEXA	460 - 464	461	N-->F
1PEXA	460 - 464	461	N-->I
1PEXA	460 - 464	461	N-->K
1PEXA	460 - 464	461	N-->R
1PEXA	460 - 464	461	N-->S
1PEXA	460 - 464	461	N-->T
1PEXA	460 - 464	461	N-->V
1PEXA	460 - 464	462	S-->C
1PEXA	460 - 464	462	S-->F
1PEXA	460 - 464	462	S-->G
1PEXA	460 - 464	462	S-->I
1PEXA	460 - 464	462	S-->N
1PEXA	460 - 464	462	S-->P
1PEXA	460 - 464	462	S-->Q
1PEXA	460 - 464	462	S-->V
1PEXA	460 - 464	462	S-->Y
1PEXA	460 - 464	463	I-->F
1PEXA	460 - 464	463	I-->G
1PEXA	460 - 464	463	I-->K
1PEXA	460 - 464	463	I-->L
1PEXA	460 - 464	463	I-->N
1PEXA	460 - 464	463	I-->R

1PEXA	460 - 464	463	I-->V
1PEXA	460 - 464	464	L-->F
1PEXA	460 - 464	464	L-->G
1PEXA	460 - 464	464	L-->I
1PEXA	460 - 464	464	L-->K
1PEXA	460 - 464	464	L-->P
1PEXA	460 - 464	464	L-->R
1PEXA	460 - 464	464	L-->S
1PEXA	460 - 464	464	L-->T
1PEXA	460 - 464	464	L-->V
1PEXA	460 - 464	464	L-->W
1PEXA	460 - 464	464	L-->Y
1ON8A	109 - 114	112	W-->A
1ON8A	109 - 114	112	W-->K
1ON8A	132 - 136	132	M-->C
1ON8A	132 - 136	132	M-->D
1ON8A	132 - 136	132	M-->G
1ON8A	132 - 136	132	M-->S
1ON8A	132 - 136	132	M-->T
1ON8A	132 - 136	132	M-->Y
1ON8A	132 - 136	133	R-->D
1ON8A	132 - 136	133	R-->G
1ON8A	132 - 136	133	R-->K
1ON8A	132 - 136	133	R-->T
1ON8A	132 - 136	134	N-->D
1ON8A	132 - 136	136	L-->T
1XJ5A	103 - 108	107	Q-->V

1XJ5A	110 - 115	110	I-->K
1XJ5A	110 - 115	112	H-->G
1XJ5A	110 - 115	112	H-->L
1XJ5A	110 - 115	115	L-->T
1XJ5A	133 - 138	133	G-->L
1XJ5A	133 - 138	136	R-->L
1XJ5A	133 - 138	136	R-->T
1XJ5A	133 - 138	137	E-->V
1XJ5A	183 - 188	186	F-->R
1XJ5A	183 - 188	188	K-->L
1XJ5A	183 - 188	188	K-->N
1XJ5A	208 - 213	209	A-->G
1XJ5A	208 - 213	209	A-->L
1XJ5A	208 - 213	212	L-->E
1XJ5A	208 - 213	212	L-->T
1XJ5A	312 - 317	312	A-->V
1XJ5A	312 - 317	315	H-->R
1XJ5A	323 - 329	327	K-->A
1KP0A	48 - 55	49	H-->N
1KP0A	86 - 90	86	M-->G
1KP0A	86 - 90	88	W-->D
1KP0A	86 - 90	88	W-->K
1KP0A	86 - 90	89	R-->D
1KP0A	86 - 90	90	R-->L
1KP0A	224 - 228	224	G-->A
1KP0A	224 - 228	224	G-->D
1KP0A	224 - 228	224	G-->F

1KP0A	224 - 228	224	G-->H
1KP0A	224 - 228	224	G-->K
1KP0A	224 - 228	224	G-->S
1KP0A	224 - 228	224	G-->T
1KP0A	224 - 228	225	I-->G
1KP0A	224 - 228	225	I-->H
1KP0A	224 - 228	225	I-->K
1KP0A	224 - 228	225	I-->N
1KP0A	224 - 228	225	I-->P
1KP0A	224 - 228	225	I-->W
1KP0A	224 - 228	225	I-->Y
1KP0A	224 - 228	226	N-->E
1KP0A	224 - 228	226	N-->F
1KP0A	224 - 228	226	N-->G
1KP0A	224 - 228	226	N-->H
1KP0A	224 - 228	226	N-->I
1KP0A	224 - 228	226	N-->K
1KP0A	224 - 228	226	N-->P
1KP0A	224 - 228	226	N-->Q
1KP0A	224 - 228	226	N-->R
1KP0A	224 - 228	226	N-->S
1KP0A	224 - 228	226	N-->T
1KP0A	224 - 228	226	N-->V
1KP0A	224 - 228	226	N-->Y
1KP0A	224 - 228	227	T-->D
1KP0A	224 - 228	227	T-->E
1KP0A	224 - 228	227	T-->F

1KP0A	224 - 228	227	T-->G	
1KP0A	224 - 228	227	T-->H	
1KP0A	224 - 228	227	T-->I	
1KP0A	224 - 228	227	T-->K	
1KP0A	224 - 228	227	T-->L	
1KP0A	224 - 228	227	T-->P	
1KP0A	224 - 228	227	T-->R	
1KP0A	224 - 228	227	T-->S	
1KP0A	224 - 228	227	T-->V	
1KP0A	224 - 228	228	D-->A	
1KP0A	224 - 228	228	D-->E	
1KP0A	224 - 228	228	D-->G	
1KP0A	224 - 228	228	D-->I	
1KP0A	224 - 228	228	D-->L	
1KP0A	224 - 228	228	D-->R	
1KP0A	224 - 228	228	D-->S	
1KP0A	224 - 228	228	D-->T	
1S31A	306 - 310	306	L-->A	
1S31A	306 - 310	306	L-->F	
1S31A	306 - 310	306	L-->G	
1S31A	306 - 310	306	L-->I	
1S31A	306 - 310	306	L-->K	
1S31A	306 - 310	306	L-->S	
1S31A	306 - 310	306	L-->V	
1S31A	306 - 310	307	E-->A	
1S31A	306 - 310	307	E-->C	
1S31A	306 - 310	307	E-->D	

1S31A	306 - 310	307	E-->G
1S31A	306 - 310	307	E-->L
1S31A	306 - 310	307	E-->P
1S31A	306 - 310	307	E-->R
1S31A	306 - 310	308	E-->C
1S31A	306 - 310	308	E-->F
1S31A	306 - 310	308	E-->G
1S31A	306 - 310	308	E-->L
1S31A	306 - 310	308	E-->S
1S31A	306 - 310	309	F-->A
1S31A	306 - 310	309	F-->D
1S31A	306 - 310	309	F-->G
1S31A	306 - 310	309	F-->I
1S31A	306 - 310	309	F-->K
1S31A	306 - 310	309	F-->R
1S31A	306 - 310	309	F-->V
1S31A	306 - 310	309	F-->Y
1S31A	306 - 310	310	A-->C
1S31A	306 - 310	310	A-->D
1S31A	306 - 310	310	A-->G
1S31A	306 - 310	310	A-->I
1S31A	306 - 310	310	A-->N
1S31A	306 - 310	310	A-->P
1S31A	306 - 310	310	A-->Q
1S31A	306 - 310	310	A-->R
1S31A	373 - 377	373	P-->A
1S31A	373 - 377	373	P-->D

1S31A	373 - 377	373	P-->E
1S31A	373 - 377	373	P-->F
1S31A	373 - 377	373	P-->G
1S31A	373 - 377	373	P-->H
1S31A	373 - 377	373	P-->I
1S31A	373 - 377	373	P-->K
1S31A	373 - 377	373	P-->N
1S31A	373 - 377	373	P-->S
1S31A	373 - 377	373	P-->T
1S31A	373 - 377	373	P-->V
1S31A	373 - 377	374	T-->A
1S31A	373 - 377	374	T-->E
1S31A	373 - 377	374	T-->F
1S31A	373 - 377	374	T-->G
1S31A	373 - 377	374	T-->H
1S31A	373 - 377	374	T-->I
1S31A	373 - 377	374	T-->L
1S31A	373 - 377	374	T-->P
1S31A	373 - 377	374	T-->R
1S31A	373 - 377	374	T-->S
1S31A	373 - 377	374	T-->V
1S31A	373 - 377	374	T-->Y
1S31A	373 - 377	375	D-->A
1S31A	373 - 377	375	D-->E
1S31A	373 - 377	375	D-->F
1S31A	373 - 377	375	D-->G
1S31A	373 - 377	375	D-->I

1S31A	373	377	375	D-->P
1S31A	373	377	375	D-->S
1S31A	373	377	375	D-->T
1S31A	373	377	375	D-->W
1S31A	373	377	375	D-->Y
1S31A	373	377	376	L-->F
1S31A	373	377	376	L-->G
1S31A	373	377	376	L-->K
1S31A	373	377	376	L-->N
1S31A	373	377	376	L-->P
1S31A	373	377	376	L-->T
1S31A	373	377	377	S-->D
1S31A	373	377	377	S-->F
1S31A	373	377	377	S-->G
1S31A	373	377	377	S-->H
1S31A	373	377	377	S-->I
1S31A	373	377	377	S-->K
1S31A	373	377	377	S-->M
1S31A	373	377	377	S-->P
1S31A	373	377	377	S-->R
1S31A	373	377	377	S-->V
1S31A	465	470	469	W-->E
1MHZG	75	80	75	V-->A
1MHZG	75	80	75	V-->T
1MHZG	75	80	76	A-->V
1MHZG	75	80	77	D-->M
1MHZG	75	80	77	D-->S

1MHZG	75 - 80	78	L-->E
1MHZG	75 - 80	78	L-->I
1MHZG	153 - 160	155	G-->T
1MHZB	141 - 145	141	P-->T
1MHZB	141 - 145	141	P-->V
1MHZB	141 - 145	142	Y-->D
1MHZB	141 - 145	142	Y-->G
1MHZB	141 - 145	143	W-->A
1MHZB	141 - 145	143	W-->D
1MHZB	141 - 145	143	W-->F
1MHZB	141 - 145	143	W-->G
1MHZB	141 - 145	143	W-->H
1MHZB	141 - 145	143	W-->N
1MHZB	141 - 145	143	W-->R
1MHZB	141 - 145	143	W-->S
1MHZB	141 - 145	143	W-->Y
1MHZB	141 - 145	144	R-->D
1MHZB	141 - 145	144	R-->K
1MHZB	141 - 145	144	R-->Q
1MHZB	141 - 145	145	D-->Q
1MHZB	167 - 172	167	S-->K
1MHZB	167 - 172	169	V-->K
1MHZB	167 - 172	171	R-->P
1MHZB	167 - 172	172	D-->A
1MHZB	268 - 272	268	F-->D
1MHZB	268 - 272	268	F-->T
1MHZB	268 - 272	268	F-->V

1MHZB	268 - 272	269	Q-->A	
1MHZB	268 - 272	269	Q-->C	
1MHZB	268 - 272	269	Q-->F	
1MHZB	268 - 272	269	Q-->G	
1MHZB	268 - 272	269	Q-->P	
1MHZB	268 - 272	269	Q-->T	
1MHZB	268 - 272	270	R-->M	
1MHZB	268 - 272	270	R-->V	
1MHZB	268 - 272	270	R-->Y	
1MHZB	268 - 272	271	L-->E	
1MHZB	268 - 272	271	L-->I	
1MHZB	268 - 272	271	L-->S	
1MHZB	268 - 272	271	L-->T	
1MHZB	268 - 272	272	A-->N	
1MHZB	268 - 272	272	A-->T	
2PK2A	101 - 105	101	L-->E	
2PK2A	101 - 105	101	L-->G	
2PK2A	101 - 105	101	L-->H	
2PK2A	101 - 105	101	L-->K	
2PK2A	101 - 105	101	L-->P	
2PK2A	101 - 105	101	L-->R	
2PK2A	101 - 105	101	L-->V	
2PK2A	101 - 105	102	E-->G	
2PK2A	101 - 105	102	E-->K	
2PK2A	101 - 105	102	E-->L	
2PK2A	101 - 105	102	E-->N	
2PK2A	101 - 105	102	E-->P	

2PK2A	101 - 105	103	H-->A
2PK2A	101 - 105	103	H-->C
2PK2A	101 - 105	103	H-->E
2PK2A	101 - 105	103	H-->G
2PK2A	101 - 105	103	H-->I
2PK2A	101 - 105	103	H-->L
2PK2A	101 - 105	103	H-->S
2PK2A	101 - 105	103	H-->W
2PK2A	101 - 105	104	V-->C
2PK2A	101 - 105	104	V-->H
2PK2A	101 - 105	104	V-->P
2PK2A	101 - 105	104	V-->S
2PK2A	101 - 105	104	V-->W
2PK2A	101 - 105	105	I-->G
2PK2A	101 - 105	105	I-->Q
2PK2A	101 - 105	105	I-->S
2PK2A	108 - 112	108	A-->I
2PK2A	108 - 112	109	H-->G
2PK2A	108 - 112	109	H-->Y
2PK2A	108 - 112	110	T-->M
2PK2A	108 - 112	110	T-->V
2PK2A	108 - 112	111	C-->K
2PK2A	108 - 112	111	C-->L
2PK2A	108 - 112	111	C-->N
2PK2A	108 - 112	111	C-->P
2PK2A	108 - 112	111	C-->Q
2PK2A	108 - 112	112	L-->G

2PK2A	108 - 112	112	L-->I
2PK2A	108 - 112	112	L-->Y
1EF7A	59 - 65	60	Q-->I
2UX8E	165 - 170	166	D-->L
3CKDC	285 - 290	285	L-->E
3CKDC	285 - 290	285	L-->K
3CKDC	285 - 290	285	L-->R
3CKDC	285 - 290	285	L-->S
3CKDC	285 - 290	287	D-->L
3CKDC	285 - 290	287	D-->M
3CKDC	285 - 290	287	D-->Q
3CKDC	285 - 290	288	A-->D
3CKDC	285 - 290	288	A-->R
3CKDC	285 - 290	289	V-->A
3CKDC	285 - 290	290	T-->I
3CKDC	285 - 290	290	T-->L
1OZVB	107 - 111	107	P-->A
1OZVB	107 - 111	107	P-->D
1OZVB	107 - 111	107	P-->E
1OZVB	107 - 111	107	P-->F
1OZVB	107 - 111	107	P-->I
1OZVB	107 - 111	107	P-->L
1OZVB	107 - 111	107	P-->M
1OZVB	107 - 111	107	P-->N
1OZVB	107 - 111	108	D-->E
1OZVB	107 - 111	108	D-->G
1OZVB	107 - 111	108	D-->H

1OZVB	107 - 111	108	D-->K	
1OZVB	107 - 111	108	D-->L	
1OZVB	107 - 111	108	D-->M	
1OZVB	107 - 111	108	D-->N	
1OZVB	107 - 111	108	D-->R	
1OZVB	107 - 111	108	D-->S	
1OZVB	107 - 111	108	D-->T	
1OZVB	107 - 111	108	D-->V	
1OZVB	107 - 111	108	D-->Y	
1OZVB	107 - 111	109	A-->C	
1OZVB	107 - 111	109	A-->D	
1OZVB	107 - 111	109	A-->G	
1OZVB	107 - 111	109	A-->K	
1OZVB	107 - 111	109	A-->R	
1OZVB	107 - 111	109	A-->S	
1OZVB	107 - 111	109	A-->W	
1OZVB	107 - 111	109	A-->Y	
1OZVB	107 - 111	110	V-->D	
1OZVB	107 - 111	110	V-->E	
1OZVB	107 - 111	110	V-->F	
1OZVB	107 - 111	110	V-->G	
1OZVB	107 - 111	110	V-->I	
1OZVB	107 - 111	110	V-->K	
1OZVB	107 - 111	110	V-->L	
1OZVB	107 - 111	110	V-->N	
1OZVB	107 - 111	110	V-->P	
1OZVB	107 - 111	110	V-->S	

1OZVB	107 - 111	110	V-->Y
1OZVB	107 - 111	111	A-->F
1OZVB	107 - 111	111	A-->G
1OZVB	107 - 111	111	A-->I
1OZVB	107 - 111	111	A-->K
1OZVB	107 - 111	111	A-->L
1OZVB	107 - 111	111	A-->M
1OZVB	107 - 111	111	A-->R
1OZVB	107 - 111	111	A-->S
1OZVB	107 - 111	111	A-->V
1OZVB	162 - 166	162	E-->G
1OZVB	162 - 166	162	E-->H
1OZVB	162 - 166	162	E-->I
1OZVB	162 - 166	162	E-->L
1OZVB	162 - 166	162	E-->P
1OZVB	162 - 166	162	E-->S
1OZVB	162 - 166	163	E-->A
1OZVB	162 - 166	163	E-->D
1OZVB	162 - 166	163	E-->L
1OZVB	162 - 166	163	E-->R
1OZVB	162 - 166	163	E-->T
1OZVB	162 - 166	163	E-->V
1OZVB	162 - 166	163	E-->Y
1OZVB	162 - 166	164	E-->C
1OZVB	162 - 166	164	E-->G
1OZVB	162 - 166	164	E-->I
1OZVB	162 - 166	164	E-->P

1OZVB	162 - 166	164	E-->S
1OZVB	162 - 166	164	E-->V
1OZVB	162 - 166	164	E-->Y
1OZVB	162 - 166	165	L-->G
1OZVB	162 - 166	165	L-->I
1OZVB	162 - 166	165	L-->P
1OZVB	162 - 166	165	L-->R
1OZVB	162 - 166	166	Q-->A
1OZVB	162 - 166	166	Q-->D
1OZVB	162 - 166	166	Q-->E
1OZVB	162 - 166	166	Q-->F
1OZVB	162 - 166	166	Q-->I
1OZVB	162 - 166	166	Q-->L
1OZVB	162 - 166	166	Q-->N
1OZVB	162 - 166	166	Q-->P
1OZVB	162 - 166	166	Q-->S
1OZVB	162 - 166	166	Q-->T
1OZVB	162 - 166	166	Q-->V
1OZVB	293 - 299	299	D-->G
1OZVB	464 - 468	464	Y-->D
1OZVB	464 - 468	464	Y-->E
1OZVB	464 - 468	464	Y-->G
1OZVB	464 - 468	464	Y-->V
1OZVB	464 - 468	466	E-->D
1OZVB	464 - 468	466	E-->T
1OZVB	464 - 468	467	R-->S
1OZVB	464 - 468	468	R-->I

1OZVB	464 - 468	468	R-->V
1KG0C	149 - 154	149	L-->A
1KG0C	149 - 154	149	L-->R
1FCVA	213 - 218	215	L-->S
1FCVA	324 - 330	326	P-->G
1FCVA	324 - 330	330	R-->L
1ORHA	43 - 49	45	H-->T
1JFIA	67 - 71	67	T-->E
1JFIA	67 - 71	67	T-->L
1JFIA	67 - 71	67	T-->Q
1JFIA	67 - 71	67	T-->R
1JFIA	67 - 71	67	T-->Y
1JFIA	67 - 71	68	S-->A
1JFIA	67 - 71	68	S-->F
1JFIA	67 - 71	68	S-->G
1JFIA	67 - 71	68	S-->M
1JFIA	67 - 71	68	S-->R
1JFIA	67 - 71	68	S-->V
1JFIA	67 - 71	69	H-->A
1JFIA	67 - 71	69	H-->E
1JFIA	67 - 71	69	H-->F
1JFIA	67 - 71	69	H-->G
1JFIA	67 - 71	69	H-->K
1JFIA	67 - 71	69	H-->P
1JFIA	67 - 71	69	H-->Q
1JFIA	67 - 71	70	L-->M
1JFIA	67 - 71	70	L-->N

1JFIA	67 - 71	71	K-->A
1JFIA	67 - 71	71	K-->G
1JFIA	67 - 71	71	K-->I
1JFIA	67 - 71	71	K-->P
1JFIA	67 - 71	71	K-->T
1JFIA	67 - 71	71	K-->V
2O8AJ	50 - 55	52	N-->Q
2O8AJ	50 - 55	55	A-->E
1G1XH	37 - 41	37	V-->A
1G1XH	37 - 41	37	V-->D
1G1XH	37 - 41	37	V-->G
1G1XH	37 - 41	37	V-->H
1G1XH	37 - 41	37	V-->I
1G1XH	37 - 41	37	V-->K
1G1XH	37 - 41	37	V-->L
1G1XH	37 - 41	37	V-->M
1G1XH	37 - 41	37	V-->P
1G1XH	37 - 41	37	V-->T
1G1XH	37 - 41	37	V-->Y
1G1XH	37 - 41	38	E-->A
1G1XH	37 - 41	38	E-->C
1G1XH	37 - 41	38	E-->D
1G1XH	37 - 41	38	E-->F
1G1XH	37 - 41	38	E-->G
1G1XH	37 - 41	38	E-->I
1G1XH	37 - 41	38	E-->L
1G1XH	37 - 41	38	E-->P

1G1XH	37 - 41	38	E-->Q
1G1XH	37 - 41	38	E-->R
1G1XH	37 - 41	38	E-->T
1G1XH	37 - 41	38	E-->V
1G1XH	37 - 41	38	E-->W
1G1XH	37 - 41	38	E-->Y
1G1XH	37 - 41	39	V-->D
1G1XH	37 - 41	39	V-->E
1G1XH	37 - 41	39	V-->G
1G1XH	37 - 41	39	V-->I
1G1XH	37 - 41	39	V-->K
1G1XH	37 - 41	39	V-->L
1G1XH	37 - 41	39	V-->P
1G1XH	37 - 41	39	V-->S
1G1XH	37 - 41	39	V-->T
1G1XH	37 - 41	39	V-->Y
1G1XH	37 - 41	40	L-->A
1G1XH	37 - 41	40	L-->D
1G1XH	37 - 41	40	L-->F
1G1XH	37 - 41	40	L-->G
1G1XH	37 - 41	40	L-->I
1G1XH	37 - 41	40	L-->K
1G1XH	37 - 41	40	L-->P
1G1XH	37 - 41	40	L-->Q
1G1XH	37 - 41	40	L-->S
1G1XH	37 - 41	40	L-->T
1G1XH	37 - 41	40	L-->V

1G1XH	37	41	40	L-->W
1G1XH	37	41	41	K-->A
1G1XH	37	41	41	K-->D
1G1XH	37	41	41	K-->E
1G1XH	37	41	41	K-->G
1G1XH	37	41	41	K-->H
1G1XH	37	41	41	K-->I
1G1XH	37	41	41	K-->L
1G1XH	37	41	41	K-->N
1G1XH	37	41	41	K-->P
1G1XH	37	41	41	K-->R
1G1XH	37	41	41	K-->S
1G1XH	37	41	41	K-->T
1G1XH	37	41	41	K-->V
1G1XH	37	41	41	K-->W
1P7NA	103	107	103	L-->A
1P7NA	103	107	103	L-->E
1P7NA	103	107	103	L-->P
1P7NA	103	107	104	N-->A
1P7NA	103	107	104	N-->C
1P7NA	103	107	104	N-->D
1P7NA	103	107	104	N-->E
1P7NA	103	107	104	N-->I
1P7NA	103	107	104	N-->L
1P7NA	103	107	104	N-->P
1P7NA	103	107	104	N-->R
1P7NA	103	107	104	N-->S

1P7NA	103	107	104	N-->V
1P7NA	103	107	104	N-->W
1P7NA	103	107	105	R-->A
1P7NA	103	107	105	R-->D
1P7NA	103	107	105	R-->E
1P7NA	103	107	105	R-->G
1P7NA	103	107	105	R-->N
1P7NA	103	107	106	L-->F
1P7NA	103	107	106	L-->K
1P7NA	103	107	106	L-->N
1P7NA	103	107	107	K-->A
1P7NA	103	107	107	K-->E
1P7NA	103	107	107	K-->F
1P7NA	103	107	107	K-->L
1P7NA	103	107	107	K-->Q
1P7NA	103	107	107	K-->R
1P7NA	103	107	107	K-->S
2A5YC	214	219	215	D-->E
2A5YC	407	411	407	V-->K
2A5YC	407	411	407	V-->N
2A5YC	407	411	407	V-->P
2A5YC	407	411	407	V-->S
2A5YC	407	411	408	K-->E
2A5YC	407	411	408	K-->L
2A5YC	407	411	408	K-->M
2A5YC	407	411	409	L-->D
2A5YC	407	411	409	L-->Q

2A5YC	407	- 411	410	W-->A
2A5YC	407	- 411	410	W-->C
2A5YC	407	- 411	410	W-->E
2A5YC	407	- 411	410	W-->I
2A5YC	407	- 411	410	W-->L
2A5YC	407	- 411	410	W-->M
2A5YC	407	- 411	410	W-->N
2A5YC	407	- 411	410	W-->P
2A5YC	407	- 411	410	W-->Q
2A5YC	407	- 411	410	W-->R
2A5YC	407	- 411	410	W-->S
2A5YC	407	- 411	410	W-->T
2A5YC	407	- 411	410	W-->V
2A5YC	407	- 411	410	W-->Y
2A5YC	407	- 411	411	S-->G
2A5YC	407	- 411	411	S-->N
2A5YC	407	- 411	411	S-->P
2A5YC	407	- 411	411	S-->R
2A5YC	407	- 411	411	S-->Y
2R76A	140	- 145	140	E-->R
2R76A	140	- 145	140	E-->S
2R76A	140	- 145	140	E-->T
2R76A	140	- 145	141	L-->G
2R76A	140	- 145	141	L-->K
2R76A	140	- 145	141	L-->P
2R76A	140	- 145	142	L-->K
2R76A	140	- 145	144	K-->S

2R76A	140	145	145	E-->G
2BHHA	87	92	91	M-->G
2BHHA	284	288	284	P-->N
2BHHA	284	288	285	F-->A
2BHHA	284	288	285	F-->E
2BHHA	284	288	285	F-->G
2BHHA	284	288	285	F-->K
2BHHA	284	288	285	F-->Y
2BHHA	284	288	286	F-->D
2BHHA	284	288	287	Q-->A
2BHHA	284	288	287	Q-->D
2BHHA	284	288	287	Q-->K
2BHHA	284	288	287	Q-->N
2BHHA	284	288	287	Q-->P
2BHHA	284	288	287	Q-->T
2BHHA	284	288	288	D-->N
2BHHA	284	288	288	D-->Q
2P7VA	72	77	75	R-->K
2ELBA	241	246	242	S-->V
2ELBA	241	246	245	E-->F
2ELBA	241	246	245	E-->I
2ELBA	241	246	245	E-->L
2ELBA	241	246	245	E-->R
2ELBA	241	246	246	T-->I
1O9KB	752	758	757	N-->G
2H1DA	130	135	134	A-->D
2H1DA	130	135	135	Y-->T

2VG4D	78 - 83	80	R-->P
2VG4D	78 - 83	81	W-->F
2VG4D	78 - 83	81	W-->R
1ML8A	114 - 119	117	L-->I
1ML8A	114 - 119	118	M-->I
1ML8A	114 - 119	118	M-->P
1ML8A	114 - 119	119	L-->G
2AUCC	143 - 147	143	E-->D
2AUCC	143 - 147	143	E-->F
2AUCC	143 - 147	143	E-->G
2AUCC	143 - 147	143	E-->K
2AUCC	143 - 147	143	E-->M
2AUCC	143 - 147	143	E-->P
2AUCC	143 - 147	143	E-->R
2AUCC	143 - 147	143	E-->T
2AUCC	143 - 147	143	E-->V
2AUCC	143 - 147	144	E-->A
2AUCC	143 - 147	144	E-->G
2AUCC	143 - 147	144	E-->H
2AUCC	143 - 147	144	E-->L
2AUCC	143 - 147	144	E-->P
2AUCC	143 - 147	144	E-->Q
2AUCC	143 - 147	144	E-->V
2AUCC	143 - 147	145	L-->G
2AUCC	143 - 147	145	L-->I
2AUCC	143 - 147	145	L-->Q
2AUCC	143 - 147	145	L-->S

2AUCC	143 - 147	145	L-->V
2AUCC	143 - 147	146	I-->A
2AUCC	143 - 147	146	I-->F
2AUCC	143 - 147	146	I-->K
2AUCC	143 - 147	146	I-->P
2AUCC	143 - 147	146	I-->Q
2AUCC	143 - 147	146	I-->R
2AUCC	143 - 147	146	I-->T
2AUCC	143 - 147	146	I-->V
2AUCC	143 - 147	147	K-->A
2AUCC	143 - 147	147	K-->D
2AUCC	143 - 147	147	K-->E
2AUCC	143 - 147	147	K-->F
2AUCC	143 - 147	147	K-->H
2AUCC	143 - 147	147	K-->M
2AUCC	143 - 147	147	K-->N
2AUCC	143 - 147	147	K-->Q
2AUCC	143 - 147	147	K-->V
2AUCC	166 - 171	168	I-->V
2AUCC	166 - 171	169	L-->G
2AUCC	166 - 171	169	L-->N
2AUCC	166 - 171	170	T-->M
2AUCC	166 - 171	170	T-->S
2P4VA	123 - 127	124	M-->E
2P4VA	123 - 127	124	M-->H
2P4VA	123 - 127	124	M-->I
2P4VA	123 - 127	124	M-->P

2P4VA	123 - 127	124	M-->Q
2P4VA	123 - 127	124	M-->R
2P4VA	123 - 127	124	M-->S
2P4VA	123 - 127	124	M-->V
2P4VA	123 - 127	124	M-->Y
2P4VA	123 - 127	125	A-->F
2P4VA	123 - 127	125	A-->G
2P4VA	123 - 127	125	A-->M
2P4VA	123 - 127	126	R-->A
2P4VA	123 - 127	126	R-->F
2P4VA	123 - 127	126	R-->H
2P4VA	123 - 127	126	R-->P
2P4VA	123 - 127	126	R-->V
2P4VA	123 - 127	126	R-->Y
2P4VA	123 - 127	127	A-->D
2P4VA	123 - 127	127	A-->S
1D9UA	105 - 110	108	I-->L
2BKNA	144 - 148	144	L-->D
2BKNA	144 - 148	144	L-->E
2BKNA	144 - 148	144	L-->G
2BKNA	144 - 148	144	L-->I
2BKNA	144 - 148	144	L-->P
2BKNA	144 - 148	144	L-->Q
2BKNA	144 - 148	144	L-->S
2BKNA	144 - 148	144	L-->T
2BKNA	144 - 148	144	L-->V
2BKNA	144 - 148	144	L-->W

2BKNA	144	148	145	A-->C
2BKNA	144	148	145	A-->D
2BKNA	144	148	145	A-->E
2BKNA	144	148	145	A-->F
2BKNA	144	148	145	A-->G
2BKNA	144	148	145	A-->H
2BKNA	144	148	145	A-->L
2BKNA	144	148	145	A-->M
2BKNA	144	148	145	A-->P
2BKNA	144	148	145	A-->Q
2BKNA	144	148	145	A-->R
2BKNA	144	148	145	A-->T
2BKNA	144	148	145	A-->V
2BKNA	144	148	145	A-->W
2BKNA	144	148	146	T-->A
2BKNA	144	148	146	T-->G
2BKNA	144	148	146	T-->L
2BKNA	144	148	146	T-->P
2BKNA	144	148	146	T-->S
2BKNA	144	148	146	T-->V
2BKNA	144	148	146	T-->Y
2BKNA	144	148	147	N-->A
2BKNA	144	148	147	N-->C
2BKNA	144	148	147	N-->D
2BKNA	144	148	147	N-->G
2BKNA	144	148	147	N-->H
2BKNA	144	148	147	N-->I

2BKNA	144 - 148	147	N-->K	
2BKNA	144 - 148	147	N-->L	
2BKNA	144 - 148	147	N-->M	
2BKNA	144 - 148	147	N-->P	
2BKNA	144 - 148	147	N-->S	
2BKNA	144 - 148	147	N-->T	
2BKNA	144 - 148	147	N-->W	
2BKNA	144 - 148	147	N-->Y	
2BKNA	144 - 148	148	T-->A	
2BKNA	144 - 148	148	T-->E	
2BKNA	144 - 148	148	T-->G	
2BKNA	144 - 148	148	T-->I	
2BKNA	144 - 148	148	T-->L	
2BKNA	144 - 148	148	T-->N	
2BKNA	144 - 148	148	T-->P	
2BKNA	144 - 148	148	T-->Q	
2BKNA	144 - 148	148	T-->R	
2BKNA	144 - 148	148	T-->V	
2BKNA	144 - 148	148	T-->Y	
2OTDA	122 - 127	122	G-->L	
2OTDA	122 - 127	122	G-->W	
2OTDA	122 - 127	123	P-->D	
2OTDA	122 - 127	123	P-->R	
2OTDA	122 - 127	124	L-->T	
2OTDA	122 - 127	126	G-->S	
2OTDA	122 - 127	127	K-->T	
1SF8H	551 - 556	551	A-->G	

1SF8H	551 - 556	552	K-->D
1SF8H	551 - 556	554	F-->G
1SF8H	551 - 556	554	F-->R
1SF8H	551 - 556	555	A-->S
1SF8H	551 - 556	556	A-->K
1SF8H	551 - 556	556	A-->P
1SF8H	619 - 623	619	N-->A
1SF8H	619 - 623	619	N-->E
1SF8H	619 - 623	619	N-->G
1SF8H	619 - 623	619	N-->H
1SF8H	619 - 623	619	N-->P
1SF8H	619 - 623	619	N-->Q
1SF8H	619 - 623	619	N-->T
1SF8H	619 - 623	619	N-->V
1SF8H	619 - 623	619	N-->W
1SF8H	619 - 623	620	Q-->D
1SF8H	619 - 623	620	Q-->E
1SF8H	619 - 623	620	Q-->G
1SF8H	619 - 623	620	Q-->I
1SF8H	619 - 623	620	Q-->K
1SF8H	619 - 623	620	Q-->N
1SF8H	619 - 623	620	Q-->P
1SF8H	619 - 623	620	Q-->R
1SF8H	619 - 623	620	Q-->S
1SF8H	619 - 623	620	Q-->T
1SF8H	619 - 623	620	Q-->V
1SF8H	619 - 623	621	L-->A

1SF8H	619 - 623	621	L-->D
1SF8H	619 - 623	621	L-->H
1SF8H	619 - 623	621	L-->I
1SF8H	619 - 623	621	L-->K
1SF8H	619 - 623	621	L-->P
1SF8H	619 - 623	621	L-->R
1SF8H	619 - 623	621	L-->T
1SF8H	619 - 623	621	L-->V
1SF8H	619 - 623	621	L-->Y
1SF8H	619 - 623	622	L-->F
1SF8H	619 - 623	622	L-->S
1SF8H	619 - 623	622	L-->T
1SF8H	619 - 623	622	L-->V
1SF8H	619 - 623	623	V-->A
1SF8H	619 - 623	623	V-->G
1SF8H	619 - 623	623	V-->L
1SF8H	619 - 623	623	V-->P
1SF8H	619 - 623	623	V-->Q
1SF8H	619 - 623	623	V-->S
1JKWA	231 - 235	231	Y-->C
1JKWA	231 - 235	231	Y-->F
1JKWA	231 - 235	231	Y-->K
1JKWA	231 - 235	231	Y-->L
1JKWA	231 - 235	231	Y-->M
1JKWA	231 - 235	231	Y-->N
1JKWA	231 - 235	231	Y-->R
1JKWA	231 - 235	231	Y-->S

1JKWA	231 - 235	231	Y-->V
1JKWA	231 - 235	232	L-->A
1JKWA	231 - 235	232	L-->D
1JKWA	231 - 235	232	L-->G
1JKWA	231 - 235	232	L-->M
1JKWA	231 - 235	232	L-->P
1JKWA	231 - 235	232	L-->R
1JKWA	231 - 235	232	L-->S
1JKWA	231 - 235	233	S-->F
1JKWA	231 - 235	233	S-->G
1JKWA	231 - 235	233	S-->H
1JKWA	231 - 235	233	S-->L
1JKWA	231 - 235	233	S-->N
1JKWA	231 - 235	233	S-->P
1JKWA	231 - 235	233	S-->T
1JKWA	231 - 235	233	S-->V
1JKWA	231 - 235	233	S-->Y
1JKWA	231 - 235	234	E-->C
1JKWA	231 - 235	234	E-->D
1JKWA	231 - 235	234	E-->F
1JKWA	231 - 235	234	E-->G
1JKWA	231 - 235	234	E-->K
1JKWA	231 - 235	234	E-->L
1JKWA	231 - 235	234	E-->R
1JKWA	231 - 235	235	S-->A
1JKWA	231 - 235	235	S-->E
1JKWA	231 - 235	235	S-->M

1JKWA	231	235	235	S-->N
1JKWA	231	235	235	S-->V
1ZHOA	98	103	98	E-->P
1ZHOA	98	103	102	K-->R
1SS9A	228	233	228	P-->V
1SS9A	228	233	230	Y-->F
1SS9A	228	233	231	R-->M
1SS9A	228	233	233	R-->L
1YDFA	212	217	212	D-->G
1YDFA	212	217	213	I-->G
1YDFA	212	217	213	I-->V
1YDFA	212	217	214	R-->T
2HKLA	433	437	433	L-->T
2HKLA	433	437	434	W-->K
2HKLA	433	437	434	W-->P
2HKLA	433	437	434	W-->S
2HKLA	433	437	435	K-->N
2HKLA	433	437	435	K-->V
2HKLA	433	437	436	T-->C
2HKLA	433	437	436	T-->R
2QWVA	19	24	19	S-->D
2QWVA	19	24	21	R-->N
2QWVA	19	24	21	R-->S
2QWVA	19	24	24	D-->F
2QWVA	19	24	24	D-->K
2QWVA	19	24	24	D-->N
1FOD2	90	94	90	V-->D

1FOD2	90 - 94	90	V-->E
1FOD2	90 - 94	90	V-->F
1FOD2	90 - 94	90	V-->G
1FOD2	90 - 94	90	V-->H
1FOD2	90 - 94	90	V-->L
1FOD2	90 - 94	90	V-->S
1FOD2	90 - 94	90	V-->Y
1FOD2	90 - 94	91	Y-->A
1FOD2	90 - 94	91	Y-->D
1FOD2	90 - 94	91	Y-->E
1FOD2	90 - 94	91	Y-->G
1FOD2	90 - 94	91	Y-->H
1FOD2	90 - 94	91	Y-->I
1FOD2	90 - 94	91	Y-->K
1FOD2	90 - 94	91	Y-->N
1FOD2	90 - 94	91	Y-->P
1FOD2	90 - 94	91	Y-->Q
1FOD2	90 - 94	91	Y-->R
1FOD2	90 - 94	91	Y-->S
1FOD2	90 - 94	91	Y-->T
1FOD2	90 - 94	91	Y-->V
1FOD2	90 - 94	92	G-->D
1FOD2	90 - 94	92	G-->E
1FOD2	90 - 94	92	G-->L
1FOD2	90 - 94	92	G-->N
1FOD2	90 - 94	92	G-->P
1FOD2	90 - 94	92	G-->V

1FOD2	90	94	92	G-->Y
1FOD2	90	94	93	S-->E
1FOD2	90	94	93	S-->G
1FOD2	90	94	93	S-->P
1FOD2	90	94	93	S-->Q
1FOD2	90	94	93	S-->T
1FOD2	90	94	93	S-->V
1FOD2	90	94	94	L-->D
1FOD2	90	94	94	L-->E
1FOD2	90	94	94	L-->F
1FOD2	90	94	94	L-->G
1FOD2	90	94	94	L-->I
1FOD2	90	94	94	L-->N
1FOD2	90	94	94	L-->P
1FOD2	90	94	94	L-->Q
1FOD2	90	94	94	L-->R
1FOD2	90	94	94	L-->S
1FOD2	90	94	94	L-->T
2CA1A	219	223	219	A-->R
2CA1A	219	223	220	D-->K
2CA1A	219	223	220	D-->Q
2CA1A	219	223	221	E-->A
2CA1A	219	223	221	E-->F
2CA1A	219	223	221	E-->R
2CA1A	219	223	222	M-->E
2CA1A	219	223	222	M-->H
2CA1A	219	223	222	M-->I

2CA1A	219	- 223	222	M-->K
2CA1A	219	- 223	222	M-->L
2CA1A	219	- 223	222	M-->Q
2CA1A	219	- 223	222	M-->R
2CA1A	219	- 223	222	M-->S
2CA1A	219	- 223	222	M-->T
2CA1A	219	- 223	222	M-->V
2CA1A	219	- 223	222	M-->Y
2CA1A	219	- 223	223	A-->G
2CA1A	219	- 223	223	A-->V
2B7FA	103	- 108	105	A-->H
2B7FA	103	- 108	106	L-->R
2A8QA	23	- 27	23	R-->A
2A8QA	23	- 27	23	R-->D
2A8QA	23	- 27	23	R-->E
2A8QA	23	- 27	23	R-->F
2A8QA	23	- 27	23	R-->G
2A8QA	23	- 27	23	R-->K
2A8QA	23	- 27	23	R-->P
2A8QA	23	- 27	23	R-->S
2A8QA	23	- 27	23	R-->T
2A8QA	23	- 27	24	E-->A
2A8QA	23	- 27	24	E-->C
2A8QA	23	- 27	24	E-->D
2A8QA	23	- 27	24	E-->G
2A8QA	23	- 27	24	E-->I
2A8QA	23	- 27	24	E-->K

2A8QA	23 - 27	24	E-->L
2A8QA	23 - 27	24	E-->N
2A8QA	23 - 27	24	E-->R
2A8QA	23 - 27	24	E-->S
2A8QA	23 - 27	24	E-->T
2A8QA	23 - 27	24	E-->V
2A8QA	23 - 27	25	E-->A
2A8QA	23 - 27	25	E-->C
2A8QA	23 - 27	25	E-->F
2A8QA	23 - 27	25	E-->G
2A8QA	23 - 27	25	E-->I
2A8QA	23 - 27	25	E-->K
2A8QA	23 - 27	25	E-->M
2A8QA	23 - 27	25	E-->P
2A8QA	23 - 27	25	E-->S
2A8QA	23 - 27	25	E-->V
2A8QA	23 - 27	26	S-->D
2A8QA	23 - 27	26	S-->E
2A8QA	23 - 27	26	S-->G
2A8QA	23 - 27	26	S-->H
2A8QA	23 - 27	26	S-->K
2A8QA	23 - 27	26	S-->N
2A8QA	23 - 27	26	S-->P
2A8QA	23 - 27	26	S-->T
2A8QA	23 - 27	26	S-->V
2A8QA	23 - 27	26	S-->Y
2A8QA	23 - 27	27	L-->P

2A8QA	23 - 27	27	L-->V
2A8QA	96 - 100	96	P-->D
2A8QA	96 - 100	96	P-->E
2A8QA	96 - 100	96	P-->F
2A8QA	96 - 100	96	P-->G
2A8QA	96 - 100	96	P-->H
2A8QA	96 - 100	96	P-->I
2A8QA	96 - 100	96	P-->L
2A8QA	96 - 100	96	P-->Q
2A8QA	96 - 100	96	P-->S
2A8QA	96 - 100	96	P-->T
2A8QA	96 - 100	96	P-->V
2A8QA	96 - 100	96	P-->W
2A8QA	96 - 100	97	A-->C
2A8QA	96 - 100	97	A-->I
2A8QA	96 - 100	97	A-->L
2A8QA	96 - 100	97	A-->M
2A8QA	96 - 100	97	A-->P
2A8QA	96 - 100	97	A-->R
2A8QA	96 - 100	97	A-->S
2A8QA	96 - 100	97	A-->T
2A8QA	96 - 100	97	A-->V
2A8QA	96 - 100	97	A-->Y
2A8QA	96 - 100	98	L-->A
2A8QA	96 - 100	98	L-->D
2A8QA	96 - 100	98	L-->G
2A8QA	96 - 100	98	L-->N

2A8QA	96 - 100	98	L-->P
2A8QA	96 - 100	98	L-->S
2A8QA	96 - 100	98	L-->T
2A8QA	96 - 100	99	A-->D
2A8QA	96 - 100	99	A-->G
2A8QA	96 - 100	99	A-->I
2A8QA	96 - 100	99	A-->L
2A8QA	96 - 100	99	A-->N
2A8QA	96 - 100	99	A-->T
2A8QA	96 - 100	99	A-->V
2A8QA	96 - 100	100	T-->A
2A8QA	96 - 100	100	T-->D
2A8QA	96 - 100	100	T-->E
2A8QA	96 - 100	100	T-->F
2A8QA	96 - 100	100	T-->G
2A8QA	96 - 100	100	T-->K
2A8QA	96 - 100	100	T-->L
2A8QA	96 - 100	100	T-->P
2A8QA	96 - 100	100	T-->R
2A8QA	96 - 100	100	T-->S
2A8QA	96 - 100	100	T-->V
2A8QA	96 - 100	100	T-->Y
1YMFA	230 - 235	230	R-->F
1YMFA	230 - 235	230	R-->S
1YMFA	230 - 235	230	R-->V
1YMFA	230 - 235	234	S-->G
1YMFA	230 - 235	234	S-->N

1YMFA	230 - 235	235	E-->L	
1YMFA	230 - 235	235	E-->N	
1YMFA	230 - 235	235	E-->P	
1YMFA	230 - 235	235	E-->V	
1YMFA	370 - 375	372	A-->Q	
1YMFA	370 - 375	372	A-->V	
1YMFA	370 - 375	373	A-->F	
1YMFA	370 - 375	373	A-->V	
1YMFA	370 - 375	374	N-->M	
1YMFA	377 - 382	377	A-->W	
1YMFA	377 - 382	382	K-->M	
1YMFA	377 - 382	382	K-->V	
1IMDA	70 - 74	70	E-->D	
1IMDA	70 - 74	70	E-->G	
1IMDA	70 - 74	70	E-->I	
1IMDA	70 - 74	70	E-->P	
1IMDA	70 - 74	70	E-->Q	
1IMDA	70 - 74	70	E-->R	
1IMDA	70 - 74	70	E-->T	
1IMDA	70 - 74	71	E-->D	
1IMDA	70 - 74	71	E-->F	
1IMDA	70 - 74	71	E-->G	
1IMDA	70 - 74	71	E-->K	
1IMDA	70 - 74	71	E-->L	
1IMDA	70 - 74	71	E-->N	
1IMDA	70 - 74	71	E-->Q	
1IMDA	70 - 74	71	E-->S	

1IMDA	70 - 74	71	E-->V
1IMDA	70 - 74	72	S-->A
1IMDA	70 - 74	72	S-->D
1IMDA	70 - 74	72	S-->F
1IMDA	70 - 74	72	S-->H
1IMDA	70 - 74	72	S-->I
1IMDA	70 - 74	72	S-->N
1IMDA	70 - 74	72	S-->R
1IMDA	70 - 74	72	S-->V
1IMDA	70 - 74	73	V-->G
1IMDA	70 - 74	73	V-->I
1IMDA	70 - 74	73	V-->K
1IMDA	70 - 74	73	V-->N
1IMDA	70 - 74	73	V-->S
1IMDA	70 - 74	73	V-->Y
1IMDA	70 - 74	74	A-->D
1IMDA	70 - 74	74	A-->G
1IMDA	70 - 74	74	A-->I
1IMDA	70 - 74	74	A-->L
1IMDA	70 - 74	74	A-->R
1IMDA	95 - 100	95	T-->M
1IMDA	95 - 100	98	F-->K
1ULJA	19 - 23	19	D-->A
1ULJA	19 - 23	19	D-->C
1ULJA	19 - 23	19	D-->E
1ULJA	19 - 23	19	D-->F
1ULJA	19 - 23	19	D-->G

1ULJA	19 - 23	19	D-->I
1ULJA	19 - 23	19	D-->K
1ULJA	19 - 23	19	D-->L
1ULJA	19 - 23	19	D-->Q
1ULJA	19 - 23	19	D-->R
1ULJA	19 - 23	19	D-->T
1ULJA	19 - 23	19	D-->V
1ULJA	19 - 23	19	D-->Y
1ULJA	19 - 23	20	A-->D
1ULJA	19 - 23	20	A-->F
1ULJA	19 - 23	20	A-->G
1ULJA	19 - 23	20	A-->I
1ULJA	19 - 23	20	A-->N
1ULJA	19 - 23	20	A-->P
1ULJA	19 - 23	20	A-->Q
1ULJA	19 - 23	20	A-->R
1ULJA	19 - 23	20	A-->S
1ULJA	19 - 23	20	A-->V
1ULJA	19 - 23	20	A-->W
1ULJA	19 - 23	20	A-->Y
1ULJA	19 - 23	21	D-->A
1ULJA	19 - 23	21	D-->C
1ULJA	19 - 23	21	D-->E
1ULJA	19 - 23	21	D-->I
1ULJA	19 - 23	21	D-->K
1ULJA	19 - 23	21	D-->N
1ULJA	19 - 23	21	D-->S

1ULJA	19 - 23	21	D-->V
1ULJA	19 - 23	21	D-->Y
1ULJA	19 - 23	22	I-->A
1ULJA	19 - 23	22	I-->L
1ULJA	19 - 23	22	I-->N
1ULJA	19 - 23	22	I-->R
1ULJA	19 - 23	22	I-->V
1ULJA	19 - 23	22	I-->W
1ULJA	19 - 23	22	I-->Y
1ULJA	19 - 23	23	A-->E
1ULJA	19 - 23	23	A-->F
1ULJA	19 - 23	23	A-->G
1ULJA	19 - 23	23	A-->K
1ULJA	19 - 23	23	A-->N
1ULJA	19 - 23	23	A-->P
1ULJA	19 - 23	23	A-->R
1ULJA	19 - 23	23	A-->T
1ULJA	19 - 23	23	A-->V
1ULJA	19 - 23	23	A-->W
1ULJA	174 - 178	174	L-->A
1ULJA	174 - 178	174	L-->F
1ULJA	174 - 178	174	L-->G
1ULJA	174 - 178	174	L-->P
1ULJA	174 - 178	175	D-->F
1ULJA	174 - 178	175	D-->K
1ULJA	174 - 178	175	D-->P
1ULJA	174 - 178	175	D-->T

1ULJA	174 - 178	176	T-->G
1ULJA	174 - 178	176	T-->K
1ULJA	174 - 178	176	T-->L
1ULJA	174 - 178	176	T-->M
1ULJA	174 - 178	176	T-->S
1ULJA	174 - 178	176	T-->Y
1ULJA	174 - 178	177	Y-->A
1ULJA	174 - 178	177	Y-->D
1ULJA	174 - 178	177	Y-->G
1ULJA	174 - 178	177	Y-->H
1ULJA	174 - 178	177	Y-->K
1ULJA	174 - 178	177	Y-->L
1ULJA	174 - 178	177	Y-->N
1ULJA	174 - 178	177	Y-->P
1ULJA	174 - 178	177	Y-->S
1ULJA	174 - 178	177	Y-->V
1ULJA	174 - 178	178	L-->A
1ULJA	174 - 178	178	L-->G
1ULJA	174 - 178	178	L-->N
1ULJA	174 - 178	178	L-->P
1ULJA	174 - 178	178	L-->Q
1ULJA	174 - 178	178	L-->S
1ULJA	303 - 307	303	T-->A
1ULJA	303 - 307	303	T-->E
1ULJA	303 - 307	303	T-->I
1ULJA	303 - 307	303	T-->L
1ULJA	303 - 307	303	T-->Q

1ULJA	303 - 307	303	T-->V	
1ULJA	303 - 307	304	E-->G	
1ULJA	303 - 307	304	E-->I	
1ULJA	303 - 307	304	E-->L	
1ULJA	303 - 307	304	E-->R	
1ULJA	303 - 307	304	E-->V	
1ULJA	303 - 307	305	R-->D	
1ULJA	303 - 307	305	R-->E	
1ULJA	303 - 307	305	R-->H	
1ULJA	303 - 307	305	R-->M	
1ULJA	303 - 307	305	R-->P	
1ULJA	303 - 307	305	R-->S	
1ULJA	303 - 307	305	R-->T	
1ULJA	303 - 307	306	G-->M	
1ULJA	303 - 307	306	G-->P	
1ULJA	303 - 307	306	G-->R	
1ULJA	303 - 307	306	G-->V	
1ULJA	303 - 307	307	Q-->C	
1ULJA	303 - 307	307	Q-->G	
1ULJA	303 - 307	307	Q-->I	
1ULJA	303 - 307	307	Q-->L	
1ULJA	303 - 307	307	Q-->M	
1ULJA	303 - 307	307	Q-->S	
1ULJA	303 - 307	307	Q-->T	
1ULJA	303 - 307	307	Q-->V	
1ULJA	303 - 307	307	Q-->W	
1ULJA	303 - 307	307	Q-->Y	

1ULJA	444 - 448	444	W-->D
1ULJA	444 - 448	444	W-->E
1ULJA	444 - 448	444	W-->F
1ULJA	444 - 448	444	W-->G
1ULJA	444 - 448	444	W-->I
1ULJA	444 - 448	444	W-->P
1ULJA	444 - 448	444	W-->R
1ULJA	444 - 448	444	W-->T
1ULJA	444 - 448	444	W-->V
1ULJA	444 - 448	445	A-->H
1ULJA	444 - 448	445	A-->Q
1ULJA	444 - 448	445	A-->S
1ULJA	444 - 448	445	A-->Y
1ULJA	444 - 448	446	A-->H
1ULJA	444 - 448	446	A-->R
1ULJA	444 - 448	447	L-->T
1ULJA	444 - 448	448	D-->G
1ULJA	444 - 448	448	D-->L
1ULJA	444 - 448	448	D-->N
1ULJA	444 - 448	448	D-->S
2VGLS	100 - 105	100	E-->L
2VGLS	100 - 105	101	L-->F
2VGLS	100 - 105	102	D-->Y
2VGLS	100 - 105	103	L-->D
2VGLS	100 - 105	104	V-->D
2VGLS	100 - 105	104	V-->Q
2VGLS	100 - 105	105	F-->H

1UTBA	138	143	142	M-->E
1UTBA	138	143	142	M-->K
1UTBA	138	143	143	E-->S
1G5QA	38	42	38	P-->A
1G5QA	38	42	38	P-->C
1G5QA	38	42	38	P-->G
1G5QA	38	42	38	P-->K
1G5QA	38	42	38	P-->M
1G5QA	38	42	38	P-->S
1G5QA	38	42	39	S-->A
1G5QA	38	42	39	S-->D
1G5QA	38	42	39	S-->E
1G5QA	38	42	39	S-->G
1G5QA	38	42	39	S-->K
1G5QA	38	42	39	S-->L
1G5QA	38	42	39	S-->N
1G5QA	38	42	39	S-->P
1G5QA	38	42	39	S-->V
1G5QA	38	42	40	S-->K
1G5QA	38	42	40	S-->Q
1G5QA	38	42	40	S-->R
1G5QA	38	42	40	S-->T
1G5QA	38	42	40	S-->V
1G5QA	38	42	41	K-->A
1G5QA	38	42	41	K-->F
1G5QA	38	42	41	K-->H
1G5QA	38	42	41	K-->I

1G5QA	38 - 42	41	K-->N
1G5QA	38 - 42	41	K-->P
1G5QA	38 - 42	41	K-->Q
1G5QA	38 - 42	41	K-->T
1G5QA	38 - 42	41	K-->V
1G5QA	38 - 42	41	K-->Y
1G5QA	38 - 42	42	N-->C
1G5QA	38 - 42	42	N-->D
1G5QA	38 - 42	42	N-->E
1G5QA	38 - 42	42	N-->G
1G5QA	38 - 42	42	N-->K
1G5QA	38 - 42	42	N-->L
1G5QA	38 - 42	42	N-->S
1G5QA	38 - 42	42	N-->T
1G5QA	46 - 51	46	T-->K
1G5QA	46 - 51	47	D-->A
1G5QA	46 - 51	47	D-->R
1G5QA	46 - 51	47	D-->Y
1G5QA	46 - 51	50	K-->A
1G5QA	46 - 51	51	L-->A
1G5QA	46 - 51	51	L-->S
1G5QA	46 - 51	51	L-->V
1G5QA	67 - 72	70	I-->G
1G5QA	67 - 72	70	I-->V
1G5QA	67 - 72	72	E-->T
2DZYA	-2 - 3	0	A-->K
2DZYA	-2 - 3	1	M-->G

2DZYA	-2 - 3	1	M-->V
2DZYA	-2 - 3	2	S-->G
2DZYA	-2 - 3	3	S-->E
2DZYA	35 - 39	35	A-->D
2DZYA	35 - 39	35	A-->E
2DZYA	35 - 39	35	A-->I
2DZYA	35 - 39	35	A-->N
2DZYA	35 - 39	35	A-->P
2DZYA	35 - 39	35	A-->S
2DZYA	35 - 39	35	A-->V
2DZYA	35 - 39	36	D-->A
2DZYA	35 - 39	36	D-->C
2DZYA	35 - 39	36	D-->E
2DZYA	35 - 39	36	D-->F
2DZYA	35 - 39	36	D-->G
2DZYA	35 - 39	36	D-->H
2DZYA	35 - 39	36	D-->I
2DZYA	35 - 39	36	D-->K
2DZYA	35 - 39	36	D-->L
2DZYA	35 - 39	36	D-->P
2DZYA	35 - 39	36	D-->S
2DZYA	35 - 39	36	D-->T
2DZYA	35 - 39	36	D-->V
2DZYA	35 - 39	36	D-->Y
2DZYA	35 - 39	37	D-->A
2DZYA	35 - 39	37	D-->E
2DZYA	35 - 39	37	D-->G

2DZYA	35 - 39	37	D-->I
2DZYA	35 - 39	37	D-->L
2DZYA	35 - 39	37	D-->P
2DZYA	35 - 39	37	D-->Q
2DZYA	35 - 39	37	D-->S
2DZYA	35 - 39	37	D-->T
2DZYA	35 - 39	37	D-->V
2DZYA	35 - 39	37	D-->Y
2DZYA	35 - 39	38	A-->E
2DZYA	35 - 39	38	A-->F
2DZYA	35 - 39	38	A-->G
2DZYA	35 - 39	38	A-->L
2DZYA	35 - 39	38	A-->N
2DZYA	35 - 39	38	A-->Q
2DZYA	35 - 39	38	A-->S
2DZYA	35 - 39	38	A-->W
2DZYA	35 - 39	38	A-->Y
2DZYA	35 - 39	39	L-->D
2DZYA	35 - 39	39	L-->E
2DZYA	35 - 39	39	L-->H
2DZYA	35 - 39	39	L-->N
2DZYA	35 - 39	39	L-->P
2DZYA	35 - 39	39	L-->S
2DZYA	35 - 39	39	L-->T
2DZYA	35 - 39	39	L-->V
2DZYA	356 - 361	357	A-->D
2DZYA	356 - 361	359	R-->L

2Q05D	121 - 125	121	I-->D
2Q05D	121 - 125	121	I-->G
2Q05D	121 - 125	121	I-->K
2Q05D	121 - 125	121	I-->L
2Q05D	121 - 125	121	I-->P
2Q05D	121 - 125	121	I-->V
2Q05D	121 - 125	122	L-->G
2Q05D	121 - 125	122	L-->K
2Q05D	121 - 125	122	L-->M
2Q05D	121 - 125	122	L-->T
2Q05D	121 - 125	123	A-->Q
2Q05D	121 - 125	123	A-->S
2Q05D	121 - 125	124	Y-->D
2Q05D	121 - 125	124	Y-->E
2Q05D	121 - 125	124	Y-->G
2Q05D	121 - 125	124	Y-->L
2Q05D	121 - 125	124	Y-->P
2Q05D	121 - 125	124	Y-->T
2Q05D	121 - 125	124	Y-->V
2Q05D	121 - 125	124	Y-->W
2Q05D	121 - 125	125	L-->A
2Q05D	121 - 125	125	L-->D
2Q05D	121 - 125	125	L-->E
2Q05D	121 - 125	125	L-->G
2Q05D	121 - 125	125	L-->K
2Q05D	121 - 125	125	L-->Q
2Q05D	121 - 125	125	L-->S

2Q05D	121 -	125	125	L-->T
2Q05D	121 -	125	125	L-->V
2HO5B	36 -	40	36	L-->H
2HO5B	36 -	40	36	L-->M
2HO5B	36 -	40	36	L-->N
2HO5B	36 -	40	36	L-->R
2HO5B	36 -	40	36	L-->T
2HO5B	36 -	40	36	L-->V
2HO5B	36 -	40	37	E-->A
2HO5B	36 -	40	37	E-->D
2HO5B	36 -	40	37	E-->F
2HO5B	36 -	40	37	E-->G
2HO5B	36 -	40	37	E-->I
2HO5B	36 -	40	37	E-->L
2HO5B	36 -	40	37	E-->P
2HO5B	36 -	40	37	E-->Q
2HO5B	36 -	40	37	E-->R
2HO5B	36 -	40	37	E-->S
2HO5B	36 -	40	37	E-->T
2HO5B	36 -	40	37	E-->V
2HO5B	36 -	40	38	T-->D
2HO5B	36 -	40	38	T-->E
2HO5B	36 -	40	38	T-->I
2HO5B	36 -	40	38	T-->K
2HO5B	36 -	40	38	T-->L
2HO5B	36 -	40	38	T-->P
2HO5B	36 -	40	38	T-->R

2HO5B	36 - 40	38	T-->V
2HO5B	36 - 40	39	A-->D
2HO5B	36 - 40	39	A-->G
2HO5B	36 - 40	39	A-->K
2HO5B	36 - 40	39	A-->P
2HO5B	36 - 40	39	A-->Q
2HO5B	36 - 40	39	A-->R
2HO5B	36 - 40	39	A-->S
2HO5B	36 - 40	39	A-->T
2HO5B	36 - 40	39	A-->Y
2HO5B	36 - 40	40	A-->D
2HO5B	36 - 40	40	A-->E
2HO5B	36 - 40	40	A-->R
2HO5B	36 - 40	40	A-->S
2HO5B	55 - 59	55	L-->A
2HO5B	55 - 59	55	L-->D
2HO5B	55 - 59	55	L-->G
2HO5B	55 - 59	55	L-->N
2HO5B	55 - 59	55	L-->T
2HO5B	55 - 59	56	E-->G
2HO5B	55 - 59	56	E-->S
2HO5B	55 - 59	57	V-->A
2HO5B	55 - 59	57	V-->C
2HO5B	55 - 59	57	V-->G
2HO5B	55 - 59	57	V-->L
2HO5B	55 - 59	57	V-->P
2HO5B	55 - 59	57	V-->Q

2HO5B	55	59	57	V-->T
2HO5B	55	59	57	V-->Y
2HO5B	55	59	58	F-->D
2HO5B	55	59	58	F-->E
2HO5B	55	59	58	F-->G
2HO5B	55	59	58	F-->L
2HO5B	55	59	58	F-->P
2HO5B	55	59	58	F-->R
2HO5B	55	59	58	F-->S
2HO5B	55	59	58	F-->T
2HO5B	55	59	58	F-->W
2HO5B	55	59	59	F-->C
2HO5B	55	59	59	F-->E
2HO5B	55	59	59	F-->G
2HO5B	55	59	59	F-->K
2HO5B	55	59	59	F-->L
2HO5B	55	59	59	F-->M
2HO5B	55	59	59	F-->N
2HO5B	55	59	59	F-->P
2HO5B	55	59	59	F-->S
2HO5B	55	59	59	F-->V
2QGXD	20	24	20	Q-->A
2QGXD	20	24	20	Q-->K
2QGXD	20	24	20	Q-->M
2QGXD	20	24	20	Q-->P
2QGXD	20	24	20	Q-->S
2QGXD	20	24	20	Q-->T

2QGXD	20 - 24	20	Q-->Y
2QGXD	20 - 24	21	S-->D
2QGXD	20 - 24	21	S-->G
2QGXD	20 - 24	21	S-->K
2QGXD	20 - 24	21	S-->N
2QGXD	20 - 24	21	S-->Q
2QGXD	20 - 24	21	S-->R
2QGXD	20 - 24	22	F-->G
2QGXD	20 - 24	22	F-->I
2QGXD	20 - 24	22	F-->Q
2QGXD	20 - 24	22	F-->S
2QGXD	20 - 24	23	K-->D
2QGXD	20 - 24	23	K-->F
2QGXD	20 - 24	23	K-->P
2QGXD	20 - 24	23	K-->Q
2QGXD	20 - 24	23	K-->T
2QGXD	20 - 24	23	K-->V
2QGXD	20 - 24	24	G-->P
2QGXD	20 - 24	24	G-->V
1J5QA	88 - 92	88	P-->A
1J5QA	88 - 92	88	P-->D
1J5QA	88 - 92	88	P-->F
1J5QA	88 - 92	88	P-->I
1J5QA	88 - 92	88	P-->L
1J5QA	88 - 92	88	P-->N
1J5QA	88 - 92	88	P-->R
1J5QA	88 - 92	88	P-->V

1J5QA	88 - 92	89	A-->D
1J5QA	88 - 92	89	A-->E
1J5QA	88 - 92	89	A-->G
1J5QA	88 - 92	89	A-->I
1J5QA	88 - 92	89	A-->L
1J5QA	88 - 92	89	A-->P
1J5QA	88 - 92	89	A-->R
1J5QA	88 - 92	89	A-->V
1J5QA	88 - 92	90	E-->A
1J5QA	88 - 92	90	E-->D
1J5QA	88 - 92	90	E-->F
1J5QA	88 - 92	90	E-->G
1J5QA	88 - 92	90	E-->L
1J5QA	88 - 92	90	E-->P
1J5QA	88 - 92	90	E-->R
1J5QA	88 - 92	90	E-->S
1J5QA	88 - 92	90	E-->V
1J5QA	88 - 92	91	E-->C
1J5QA	88 - 92	91	E-->D
1J5QA	88 - 92	91	E-->I
1J5QA	88 - 92	91	E-->K
1J5QA	88 - 92	91	E-->L
1J5QA	88 - 92	91	E-->N
1J5QA	88 - 92	91	E-->P
1J5QA	88 - 92	91	E-->R
1J5QA	88 - 92	91	E-->S
1J5QA	88 - 92	91	E-->V

1J5QA	88	92	91	E-->W
1J5QA	88	92	91	E-->Y
1J5QA	88	92	92	L-->A
1J5QA	88	92	92	L-->E
1J5QA	88	92	92	L-->G
1J5QA	88	92	92	L-->I
1J5QA	88	92	92	L-->K
1J5QA	88	92	92	L-->P
1J5QA	88	92	92	L-->R
1J5QA	88	92	92	L-->T
1J5QA	88	92	92	L-->V
1J5QA	88	92	92	L-->Y
1J5QA	331	335	331	G-->H
1J5QA	331	335	331	G-->K
1J5QA	331	335	331	G-->L
1J5QA	331	335	331	G-->R
1J5QA	331	335	332	S-->F
1J5QA	331	335	332	S-->H
1J5QA	331	335	332	S-->I
1J5QA	331	335	332	S-->L
1J5QA	331	335	332	S-->P
1J5QA	331	335	332	S-->T
1J5QA	331	335	333	Y-->D
1J5QA	331	335	333	Y-->E
1J5QA	331	335	333	Y-->H
1J5QA	331	335	333	Y-->I
1J5QA	331	335	333	Y-->K

1J5QA	331	335	333	Y-->M
1J5QA	331	335	333	Y-->S
1J5QA	331	335	333	Y-->V
1J5QA	331	335	334	F-->C
1J5QA	331	335	334	F-->D
1J5QA	331	335	334	F-->E
1J5QA	331	335	334	F-->L
1J5QA	331	335	334	F-->N
1J5QA	331	335	334	F-->R
1J5QA	331	335	334	F-->S
1J5QA	331	335	334	F-->V
1J5QA	331	335	335	N-->A
1J5QA	331	335	335	N-->C
1J5QA	331	335	335	N-->G
1J5QA	331	335	335	N-->L
1J5QA	331	335	335	N-->M
1J5QA	331	335	335	N-->P
1J5QA	331	335	335	N-->V
1J5QA	337	342	337	V-->W
1J5QA	337	342	338	Q-->K
1J5QA	337	342	340	Y-->S
1J5QA	337	342	342	S-->D
1J5QA	406	410	406	N-->A
1J5QA	406	410	406	N-->C
1J5QA	406	410	406	N-->G
1J5QA	406	410	406	N-->K
1J5QA	406	410	406	N-->L

1J5QA	406 - 410	406	N-->M
1J5QA	406 - 410	406	N-->P
1J5QA	406 - 410	406	N-->R
1J5QA	406 - 410	406	N-->S
1J5QA	406 - 410	406	N-->T
1J5QA	406 - 410	406	N-->V
1J5QA	406 - 410	407	T-->A
1J5QA	406 - 410	407	T-->C
1J5QA	406 - 410	407	T-->D
1J5QA	406 - 410	407	T-->F
1J5QA	406 - 410	407	T-->G
1J5QA	406 - 410	407	T-->I
1J5QA	406 - 410	407	T-->K
1J5QA	406 - 410	407	T-->L
1J5QA	406 - 410	407	T-->M
1J5QA	406 - 410	407	T-->N
1J5QA	406 - 410	407	T-->Q
1J5QA	406 - 410	407	T-->S
1J5QA	406 - 410	407	T-->V
1J5QA	406 - 410	407	T-->Y
1J5QA	406 - 410	408	A-->D
1J5QA	406 - 410	408	A-->E
1J5QA	406 - 410	408	A-->H
1J5QA	406 - 410	408	A-->L
1J5QA	406 - 410	408	A-->M
1J5QA	406 - 410	408	A-->N
1J5QA	406 - 410	408	A-->P

1J5QA	406 - 410	408	A-->R
1J5QA	406 - 410	408	A-->S
1J5QA	406 - 410	408	A-->V
1J5QA	406 - 410	408	A-->Y
1J5QA	406 - 410	409	T-->D
1J5QA	406 - 410	409	T-->E
1J5QA	406 - 410	409	T-->F
1J5QA	406 - 410	409	T-->G
1J5QA	406 - 410	409	T-->I
1J5QA	406 - 410	409	T-->K
1J5QA	406 - 410	409	T-->L
1J5QA	406 - 410	409	T-->M
1J5QA	406 - 410	409	T-->N
1J5QA	406 - 410	409	T-->P
1J5QA	406 - 410	409	T-->Q
1J5QA	406 - 410	409	T-->S
1J5QA	406 - 410	409	T-->Y
1J5QA	406 - 410	410	L-->I
1J5QA	406 - 410	410	L-->K
1J5QA	406 - 410	410	L-->N
1J5QA	406 - 410	410	L-->P
1J5QA	406 - 410	410	L-->T
1J5QA	406 - 410	410	L-->W
2O6IB	204 - 208	204	L-->A
2O6IB	204 - 208	204	L-->D
2O6IB	204 - 208	204	L-->F
2O6IB	204 - 208	204	L-->G

2O6IB	204 - 208	204	L-->H
2O6IB	204 - 208	204	L-->I
2O6IB	204 - 208	204	L-->K
2O6IB	204 - 208	204	L-->N
2O6IB	204 - 208	204	L-->P
2O6IB	204 - 208	204	L-->Q
2O6IB	204 - 208	204	L-->V
2O6IB	204 - 208	204	L-->Y
2O6IB	204 - 208	205	T-->D
2O6IB	204 - 208	205	T-->G
2O6IB	204 - 208	205	T-->K
2O6IB	204 - 208	205	T-->L
2O6IB	204 - 208	205	T-->P
2O6IB	204 - 208	205	T-->R
2O6IB	204 - 208	205	T-->V
2O6IB	204 - 208	205	T-->Y
2O6IB	204 - 208	206	R-->A
2O6IB	204 - 208	206	R-->D
2O6IB	204 - 208	206	R-->E
2O6IB	204 - 208	206	R-->F
2O6IB	204 - 208	206	R-->G
2O6IB	204 - 208	206	R-->L
2O6IB	204 - 208	206	R-->V
2O6IB	204 - 208	206	R-->Y
2O6IB	204 - 208	207	I-->F
2O6IB	204 - 208	207	I-->G
2O6IB	204 - 208	207	I-->L

2O6IB	204 - 208	207	I-->M
2O6IB	204 - 208	207	I-->P
2O6IB	204 - 208	207	I-->R
2O6IB	204 - 208	207	I-->S
2O6IB	204 - 208	207	I-->Y
2O6IB	204 - 208	208	L-->D
2O6IB	204 - 208	208	L-->E
2O6IB	204 - 208	208	L-->N
2O6IB	204 - 208	208	L-->T
2O6IB	281 - 285	281	V-->D
2O6IB	281 - 285	281	V-->E
2O6IB	281 - 285	281	V-->G
2O6IB	281 - 285	281	V-->L
2O6IB	281 - 285	281	V-->T
2O6IB	281 - 285	281	V-->Y
2O6IB	281 - 285	282	P-->K
2O6IB	281 - 285	282	P-->L
2O6IB	281 - 285	282	P-->M
2O6IB	281 - 285	282	P-->R
2O6IB	281 - 285	283	F-->A
2O6IB	281 - 285	283	F-->E
2O6IB	281 - 285	283	F-->G
2O6IB	281 - 285	283	F-->H
2O6IB	281 - 285	283	F-->K
2O6IB	281 - 285	283	F-->M
2O6IB	281 - 285	283	F-->Q
2O6IB	281 - 285	283	F-->T

2O6IB	281	285	283	F-->V
2O6IB	281	285	283	F-->Y
2O6IB	281	285	284	F-->A
2O6IB	281	285	284	F-->E
2O6IB	281	285	284	F-->K
2O6IB	281	285	284	F-->P
2O6IB	281	285	284	F-->V
2O6IB	281	285	285	K-->A
2O6IB	281	285	285	K-->D
2O6IB	281	285	285	K-->E
2O6IB	281	285	285	K-->G
2O6IB	281	285	285	K-->S
2O6IB	281	285	285	K-->T
2O6IB	281	285	285	K-->V
2O6IB	403	407	403	P-->A
2O6IB	403	407	403	P-->D
2O6IB	403	407	403	P-->E
2O6IB	403	407	403	P-->F
2O6IB	403	407	403	P-->G
2O6IB	403	407	403	P-->H
2O6IB	403	407	403	P-->I
2O6IB	403	407	403	P-->K
2O6IB	403	407	403	P-->L
2O6IB	403	407	403	P-->N
2O6IB	403	407	403	P-->Q
2O6IB	403	407	403	P-->R
2O6IB	403	407	403	P-->S

2O6IB	403	407	403	P-->T
2O6IB	403	407	403	P-->V
2O6IB	403	407	403	P-->Y
2O6IB	403	407	404	L-->A
2O6IB	403	407	404	L-->C
2O6IB	403	407	404	L-->D
2O6IB	403	407	404	L-->F
2O6IB	403	407	404	L-->G
2O6IB	403	407	404	L-->H
2O6IB	403	407	404	L-->N
2O6IB	403	407	404	L-->R
2O6IB	403	407	404	L-->S
2O6IB	403	407	404	L-->T
2O6IB	403	407	404	L-->V
2O6IB	403	407	404	L-->W
2O6IB	403	407	405	V-->D
2O6IB	403	407	405	V-->G
2O6IB	403	407	405	V-->H
2O6IB	403	407	405	V-->L
2O6IB	403	407	405	V-->M
2O6IB	403	407	405	V-->P
2O6IB	403	407	405	V-->S
2O6IB	403	407	406	A-->D
2O6IB	403	407	406	A-->F
2O6IB	403	407	406	A-->G
2O6IB	403	407	406	A-->I
2O6IB	403	407	406	A-->K

2O6IB	403 - 407	406	A-->L
2O6IB	403 - 407	406	A-->P
2O6IB	403 - 407	406	A-->Q
2O6IB	403 - 407	406	A-->R
2O6IB	403 - 407	406	A-->S
2O6IB	403 - 407	406	A-->V
2O6IB	403 - 407	406	A-->Y
2O6IB	403 - 407	407	A-->E
2O6IB	403 - 407	407	A-->G
2O6IB	403 - 407	407	A-->I
2O6IB	403 - 407	407	A-->L
2O6IB	403 - 407	407	A-->M
2O6IB	403 - 407	407	A-->N
2O6IB	403 - 407	407	A-->P
2O6IB	403 - 407	407	A-->R
2O6IB	403 - 407	407	A-->S
2O6IB	403 - 407	407	A-->T
2O6IB	403 - 407	407	A-->V
2O6IB	403 - 407	407	A-->Y
1HJQA	12 - 17	13	M-->D
1HJQA	12 - 17	13	M-->R
1HJQA	12 - 17	14	V-->L
1HJQA	12 - 17	16	E-->I
1HJQA	32 - 38	34	Y-->L
2H56A	9 - 15	9	P-->S
2H56A	9 - 15	14	I-->V
2H56A	83 - 88	87	R-->L

2H56A	136	140	137	W-->A
2H56A	136	140	137	W-->F
2H56A	136	140	137	W-->G
2H56A	136	140	137	W-->I
2H56A	136	140	137	W-->P
2H56A	136	140	139	A-->Y
2H56A	136	140	140	E-->A
2H56A	136	140	140	E-->R
2H56A	203	207	203	T-->A
2H56A	203	207	203	T-->G
2H56A	203	207	203	T-->I
2H56A	203	207	203	T-->S
2H56A	203	207	203	T-->V
2H56A	203	207	204	F-->A
2H56A	203	207	204	F-->G
2H56A	203	207	204	F-->L
2H56A	203	207	204	F-->M
2H56A	203	207	204	F-->R
2H56A	203	207	204	F-->V
2H56A	203	207	205	A-->P
2H56A	203	207	205	A-->R
2H56A	203	207	205	A-->T
2H56A	203	207	206	E-->A
2H56A	203	207	206	E-->F
2H56A	203	207	206	E-->L
2H56A	203	207	206	E-->P
2H56A	203	207	206	E-->S

2H56A	203 - 207	206	E-->T
2H56A	203 - 207	206	E-->V
2H56A	203 - 207	207	E-->A
2H56A	203 - 207	207	E-->D
2H56A	203 - 207	207	E-->G
2H56A	203 - 207	207	E-->P
2H56A	203 - 207	207	E-->Q
2H56A	203 - 207	207	E-->S
2H56A	203 - 207	207	E-->V
2IZVA	278 - 283	278	L-->F
2IZVA	278 - 283	278	L-->G
2IZVA	278 - 283	278	L-->N
2IZVA	278 - 283	278	L-->Y
2IZVA	278 - 283	279	L-->F
2IZVA	278 - 283	280	Q-->L
2IZVA	278 - 283	282	N-->S
2IZVA	278 - 283	282	N-->V
2IZVA	278 - 283	283	N-->E
2IZVA	278 - 283	283	N-->S
2IZVA	403 - 407	403	Y-->D
2IZVA	403 - 407	403	Y-->E
2IZVA	403 - 407	403	Y-->F
2IZVA	403 - 407	403	Y-->G
2IZVA	403 - 407	403	Y-->I
2IZVA	403 - 407	403	Y-->K
2IZVA	403 - 407	403	Y-->L
2IZVA	403 - 407	403	Y-->P

2IZVA	403 - 407	403	Y-->S
2IZVA	403 - 407	403	Y-->T
2IZVA	403 - 407	403	Y-->V
2IZVA	403 - 407	404	D-->A
2IZVA	403 - 407	404	D-->E
2IZVA	403 - 407	404	D-->K
2IZVA	403 - 407	404	D-->L
2IZVA	403 - 407	404	D-->N
2IZVA	403 - 407	404	D-->P
2IZVA	403 - 407	404	D-->R
2IZVA	403 - 407	405	G-->D
2IZVA	403 - 407	405	G-->F
2IZVA	403 - 407	405	G-->H
2IZVA	403 - 407	405	G-->P
2IZVA	403 - 407	405	G-->S
2IZVA	403 - 407	405	G-->T
2IZVA	403 - 407	405	G-->V
2IZVA	403 - 407	406	I-->C
2IZVA	403 - 407	406	I-->D
2IZVA	403 - 407	406	I-->E
2IZVA	403 - 407	406	I-->G
2IZVA	403 - 407	406	I-->K
2IZVA	403 - 407	406	I-->N
2IZVA	403 - 407	406	I-->Q
2IZVA	403 - 407	406	I-->R
2IZVA	403 - 407	406	I-->S
2IZVA	403 - 407	406	I-->V

2IZVA	403 - 407	406	I-->W
2IZVA	403 - 407	406	I-->Y
2IZVA	403 - 407	407	D-->C
2IZVA	403 - 407	407	D-->K
2IZVA	403 - 407	407	D-->L
2IZVA	403 - 407	407	D-->P
2IZVA	403 - 407	407	D-->Q
2IZVA	403 - 407	407	D-->R
2IZVA	403 - 407	407	D-->S
2IZVA	403 - 407	407	D-->T
2IZVA	403 - 407	407	D-->V
1O67A	83 - 88	85	Q-->G
1O67A	216 - 221	218	R-->A
2FA3A	289 - 293	289	L-->D
2FA3A	289 - 293	289	L-->P
2FA3A	289 - 293	289	L-->Q
2FA3A	289 - 293	289	L-->S
2FA3A	289 - 293	290	D-->A
2FA3A	289 - 293	290	D-->E
2FA3A	289 - 293	290	D-->L
2FA3A	289 - 293	290	D-->M
2FA3A	289 - 293	290	D-->N
2FA3A	289 - 293	290	D-->S
2FA3A	289 - 293	290	D-->T
2FA3A	289 - 293	290	D-->V
2FA3A	289 - 293	291	E-->D
2FA3A	289 - 293	291	E-->F

2FA3A	289 - 293	291	E-->G
2FA3A	289 - 293	291	E-->N
2FA3A	289 - 293	291	E-->P
2FA3A	289 - 293	291	E-->S
2FA3A	289 - 293	291	E-->V
2FA3A	289 - 293	292	S-->A
2FA3A	289 - 293	292	S-->G
2FA3A	289 - 293	292	S-->N
2FA3A	289 - 293	292	S-->T
2FA3A	289 - 293	292	S-->Y
2FA3A	289 - 293	293	Y-->C
2FA3A	289 - 293	293	Y-->D
2FA3A	289 - 293	293	Y-->E
2FA3A	289 - 293	293	Y-->G
2FA3A	289 - 293	293	Y-->I
2FA3A	289 - 293	293	Y-->L
2FA3A	289 - 293	293	Y-->P
2FA3A	289 - 293	293	Y-->V
2FA3A	289 - 293	293	Y-->W
2FA3A	319 - 324	320	V-->A
2FA3A	319 - 324	321	P-->G
2FA3A	319 - 324	321	P-->V
2FA3A	319 - 324	322	K-->I
2FA3A	319 - 324	324	V-->T
2FA3A	388 - 392	388	V-->A
2FA3A	388 - 392	388	V-->C
2FA3A	388 - 392	388	V-->D

2FA3A	388 - 392	388	V-->E
2FA3A	388 - 392	388	V-->F
2FA3A	388 - 392	388	V-->I
2FA3A	388 - 392	388	V-->K
2FA3A	388 - 392	388	V-->L
2FA3A	388 - 392	388	V-->M
2FA3A	388 - 392	388	V-->N
2FA3A	388 - 392	388	V-->P
2FA3A	388 - 392	388	V-->Q
2FA3A	388 - 392	388	V-->R
2FA3A	388 - 392	388	V-->S
2FA3A	388 - 392	388	V-->T
2FA3A	388 - 392	388	V-->W
2FA3A	388 - 392	388	V-->Y
2FA3A	388 - 392	389	G-->A
2FA3A	388 - 392	389	G-->C
2FA3A	388 - 392	389	G-->D
2FA3A	388 - 392	389	G-->E
2FA3A	388 - 392	389	G-->I
2FA3A	388 - 392	389	G-->K
2FA3A	388 - 392	389	G-->N
2FA3A	388 - 392	389	G-->P
2FA3A	388 - 392	389	G-->Q
2FA3A	388 - 392	389	G-->R
2FA3A	388 - 392	389	G-->S
2FA3A	388 - 392	389	G-->T
2FA3A	388 - 392	389	G-->V

2FA3A	388 - 392	390	G-->A
2FA3A	388 - 392	390	G-->D
2FA3A	388 - 392	390	G-->E
2FA3A	388 - 392	390	G-->F
2FA3A	388 - 392	390	G-->H
2FA3A	388 - 392	390	G-->I
2FA3A	388 - 392	390	G-->M
2FA3A	388 - 392	390	G-->N
2FA3A	388 - 392	390	G-->Q
2FA3A	388 - 392	390	G-->R
2FA3A	388 - 392	390	G-->S
2FA3A	388 - 392	390	G-->T
2FA3A	388 - 392	390	G-->V
2FA3A	388 - 392	390	G-->Y
2FA3A	388 - 392	391	K-->A
2FA3A	388 - 392	391	K-->C
2FA3A	388 - 392	391	K-->D
2FA3A	388 - 392	391	K-->E
2FA3A	388 - 392	391	K-->F
2FA3A	388 - 392	391	K-->G
2FA3A	388 - 392	391	K-->I
2FA3A	388 - 392	391	K-->L
2FA3A	388 - 392	391	K-->N
2FA3A	388 - 392	391	K-->R
2FA3A	388 - 392	391	K-->S
2FA3A	388 - 392	391	K-->T
2FA3A	388 - 392	391	K-->V

2FA3A	388	392	391	K-->Y
2FA3A	388	392	392	L-->A
2FA3A	388	392	392	L-->C
2FA3A	388	392	392	L-->D
2FA3A	388	392	392	L-->G
2FA3A	388	392	392	L-->I
2FA3A	388	392	392	L-->K
2FA3A	388	392	392	L-->P
2FA3A	388	392	392	L-->Q
2FA3A	388	392	392	L-->S
2FA3A	388	392	392	L-->T
2FA3A	388	392	392	L-->V
1H29D	244	248	244	C-->P
1H29D	244	248	244	C-->T
1H29D	244	248	245	A-->I
1H29D	244	248	246	G-->A
1H29D	244	248	246	G-->Q
1H29D	244	248	246	G-->S
1H29D	244	248	246	G-->T
1H29D	244	248	247	C-->F
1H29D	244	248	247	C-->K
1H29D	244	248	248	H-->F
1H29D	244	248	248	H-->G
1H29D	336	341	340	M-->N
1H29D	464	469	465	L-->V
1H29D	464	469	469	F-->I
1CC5A	12	17	12	D-->F

1CC5A	12 - 17	17	K-->L
1CC5A	53 - 58	54	A-->G
1CC5A	53 - 58	55	Q-->H
1CC5A	53 - 58	55	Q-->V
1CC5A	53 - 58	57	L-->V
1MDAH	8 - 14	13	S-->A
1MDAH	8 - 14	14	A-->C
1MDAH	8 - 14	14	A-->T
1RXVA	117 - 122	121	Q-->A
1RXVA	297 - 304	301	I-->K
2E2XA	1635 - 1640	1635	T-->I
2E2XA	1635 - 1640	1635	T-->V
2E2XA	1635 - 1640	1636	D-->S
2E2XA	1635 - 1640	1637	F-->T
2E2XA	1635 - 1640	1638	L-->M
2E2XA	1635 - 1640	1640	K-->I
2E2XA	1647 - 1651	1647	G-->A
2E2XA	1647 - 1651	1647	G-->H
2E2XA	1647 - 1651	1647	G-->L
2E2XA	1647 - 1651	1647	G-->Q
2E2XA	1647 - 1651	1648	F-->A
2E2XA	1647 - 1651	1648	F-->E
2E2XA	1647 - 1651	1648	F-->G
2E2XA	1647 - 1651	1648	F-->I
2E2XA	1647 - 1651	1648	F-->R
2E2XA	1647 - 1651	1648	F-->T
2E2XA	1647 - 1651	1648	F-->V

2E2XA	1647	- 1651	1648	F-->W
2E2XA	1647	- 1651	1649	A-->D
2E2XA	1647	- 1651	1649	A-->E
2E2XA	1647	- 1651	1649	A-->H
2E2XA	1647	- 1651	1649	A-->I
2E2XA	1647	- 1651	1649	A-->M
2E2XA	1647	- 1651	1649	A-->Q
2E2XA	1647	- 1651	1649	A-->R
2E2XA	1647	- 1651	1649	A-->S
2E2XA	1647	- 1651	1649	A-->Y
2E2XA	1647	- 1651	1650	Y-->F
2E2XA	1647	- 1651	1650	Y-->G
2E2XA	1647	- 1651	1650	Y-->I
2E2XA	1647	- 1651	1650	Y-->L
2E2XA	1647	- 1651	1650	Y-->M
2E2XA	1647	- 1651	1650	Y-->T
2E2XA	1647	- 1651	1650	Y-->V
2E2XA	1647	- 1651	1651	D-->A
2E2XA	1647	- 1651	1651	D-->F
2E2XA	1647	- 1651	1651	D-->I
2E2XA	1647	- 1651	1651	D-->K
2E2XA	1647	- 1651	1651	D-->S
2E2XA	1647	- 1651	1651	D-->T
2E2XA	1647	- 1651	1651	D-->V
2E2XA	1647	- 1651	1651	D-->W
2E2XA	1691	- 1695	1691	P-->A
2E2XA	1691	- 1695	1691	P-->D

2E2XA	1691 - 1695	1691	P-->E
2E2XA	1691 - 1695	1691	P-->F
2E2XA	1691 - 1695	1691	P-->G
2E2XA	1691 - 1695	1691	P-->I
2E2XA	1691 - 1695	1691	P-->K
2E2XA	1691 - 1695	1691	P-->M
2E2XA	1691 - 1695	1691	P-->N
2E2XA	1691 - 1695	1691	P-->Q
2E2XA	1691 - 1695	1691	P-->R
2E2XA	1691 - 1695	1691	P-->S
2E2XA	1691 - 1695	1691	P-->T
2E2XA	1691 - 1695	1691	P-->V
2E2XA	1691 - 1695	1691	P-->Y
2E2XA	1691 - 1695	1692	G-->A
2E2XA	1691 - 1695	1692	G-->F
2E2XA	1691 - 1695	1692	G-->H
2E2XA	1691 - 1695	1692	G-->I
2E2XA	1691 - 1695	1692	G-->K
2E2XA	1691 - 1695	1692	G-->L
2E2XA	1691 - 1695	1692	G-->M
2E2XA	1691 - 1695	1692	G-->R
2E2XA	1691 - 1695	1692	G-->S
2E2XA	1691 - 1695	1692	G-->T
2E2XA	1691 - 1695	1692	G-->V
2E2XA	1691 - 1695	1693	K-->D
2E2XA	1691 - 1695	1693	K-->E
2E2XA	1691 - 1695	1693	K-->F

2E2XA	1691	- 1695	1693	K-->G
2E2XA	1691	- 1695	1693	K-->H
2E2XA	1691	- 1695	1693	K-->I
2E2XA	1691	- 1695	1693	K-->L
2E2XA	1691	- 1695	1693	K-->P
2E2XA	1691	- 1695	1693	K-->Q
2E2XA	1691	- 1695	1693	K-->R
2E2XA	1691	- 1695	1693	K-->S
2E2XA	1691	- 1695	1693	K-->V
2E2XA	1691	- 1695	1693	K-->Y
2E2XA	1691	- 1695	1694	L-->C
2E2XA	1691	- 1695	1694	L-->D
2E2XA	1691	- 1695	1694	L-->G
2E2XA	1691	- 1695	1694	L-->H
2E2XA	1691	- 1695	1694	L-->I
2E2XA	1691	- 1695	1694	L-->K
2E2XA	1691	- 1695	1694	L-->N
2E2XA	1691	- 1695	1694	L-->P
2E2XA	1691	- 1695	1694	L-->S
2E2XA	1691	- 1695	1694	L-->T
2E2XA	1691	- 1695	1694	L-->Y
2E2XA	1691	- 1695	1695	A-->D
2E2XA	1691	- 1695	1695	A-->G
2E2XA	1691	- 1695	1695	A-->I
2E2XA	1691	- 1695	1695	A-->K
2E2XA	1691	- 1695	1695	A-->L
2E2XA	1691	- 1695	1695	A-->P

2E2XA	1691 - 1695	1695	A-->Q
2E2XA	1691 - 1695	1695	A-->R
2E2XA	1691 - 1695	1695	A-->S
2E2XA	1691 - 1695	1695	A-->V
2E2XA	1691 - 1695	1695	A-->Y
2E2XA	1707 - 1711	1707	A-->C
2E2XA	1707 - 1711	1707	A-->D
2E2XA	1707 - 1711	1707	A-->E
2E2XA	1707 - 1711	1707	A-->G
2E2XA	1707 - 1711	1707	A-->H
2E2XA	1707 - 1711	1707	A-->I
2E2XA	1707 - 1711	1707	A-->K
2E2XA	1707 - 1711	1707	A-->L
2E2XA	1707 - 1711	1707	A-->N
2E2XA	1707 - 1711	1707	A-->P
2E2XA	1707 - 1711	1707	A-->S
2E2XA	1707 - 1711	1707	A-->T
2E2XA	1707 - 1711	1707	A-->V
2E2XA	1707 - 1711	1708	A-->D
2E2XA	1707 - 1711	1708	A-->F
2E2XA	1707 - 1711	1708	A-->G
2E2XA	1707 - 1711	1708	A-->I
2E2XA	1707 - 1711	1708	A-->K
2E2XA	1707 - 1711	1708	A-->P
2E2XA	1707 - 1711	1708	A-->Q
2E2XA	1707 - 1711	1708	A-->R
2E2XA	1707 - 1711	1708	A-->W

2E2XA	1707 - 1711	1708	A-->Y
2E2XA	1707 - 1711	1709	T-->A
2E2XA	1707 - 1711	1709	T-->D
2E2XA	1707 - 1711	1709	T-->G
2E2XA	1707 - 1711	1709	T-->H
2E2XA	1707 - 1711	1709	T-->I
2E2XA	1707 - 1711	1709	T-->M
2E2XA	1707 - 1711	1709	T-->P
2E2XA	1707 - 1711	1709	T-->S
2E2XA	1707 - 1711	1709	T-->V
2E2XA	1707 - 1711	1710	L-->A
2E2XA	1707 - 1711	1710	L-->C
2E2XA	1707 - 1711	1710	L-->D
2E2XA	1707 - 1711	1710	L-->E
2E2XA	1707 - 1711	1710	L-->F
2E2XA	1707 - 1711	1710	L-->G
2E2XA	1707 - 1711	1710	L-->I
2E2XA	1707 - 1711	1710	L-->M
2E2XA	1707 - 1711	1710	L-->Q
2E2XA	1707 - 1711	1710	L-->R
2E2XA	1707 - 1711	1710	L-->S
2E2XA	1707 - 1711	1710	L-->T
2E2XA	1707 - 1711	1710	L-->V
2E2XA	1707 - 1711	1711	A-->D
2E2XA	1707 - 1711	1711	A-->E
2E2XA	1707 - 1711	1711	A-->F
2E2XA	1707 - 1711	1711	A-->G

2E2XA	1707	- 1711	1711	A-->H
2E2XA	1707	- 1711	1711	A-->I
2E2XA	1707	- 1711	1711	A-->L
2E2XA	1707	- 1711	1711	A-->P
2E2XA	1707	- 1711	1711	A-->Q
2E2XA	1707	- 1711	1711	A-->R
2E2XA	1707	- 1711	1711	A-->S
2E2XA	1707	- 1711	1711	A-->T
2E2XA	1707	- 1711	1711	A-->V
2CJRB	271	- 275	271	V-->A
2CJRB	271	- 275	271	V-->G
2CJRB	271	- 275	271	V-->N
2CJRB	271	- 275	272	T-->G
2CJRB	271	- 275	272	T-->L
2CJRB	271	- 275	272	T-->M
2CJRB	271	- 275	272	T-->R
2CJRB	271	- 275	272	T-->S
2CJRB	271	- 275	273	Q-->L
2CJRB	271	- 275	273	Q-->P
2CJRB	271	- 275	273	Q-->V
2CJRB	271	- 275	274	A-->D
2CJRB	271	- 275	274	A-->E
2CJRB	271	- 275	274	A-->M
2CJRB	271	- 275	274	A-->Q
2CJRB	271	- 275	274	A-->S
2CJRB	271	- 275	274	A-->T
2CJRB	271	- 275	275	F-->A

2CJRB	271 - 275	275	F-->D
2CJRB	271 - 275	275	F-->G
2CJRB	271 - 275	275	F-->N
2CJRB	271 - 275	275	F-->S
2CJRB	271 - 275	275	F-->V
2CJRB	302 - 306	302	W-->E
2CJRB	302 - 306	302	W-->I
2CJRB	302 - 306	302	W-->N
2CJRB	302 - 306	302	W-->V
2CJRB	302 - 306	302	W-->Y
2CJRB	302 - 306	303	P-->T
2CJRB	302 - 306	304	Q-->D
2CJRB	302 - 306	304	Q-->T
2B25B	73 - 77	73	L-->E
2B25B	73 - 77	73	L-->G
2B25B	73 - 77	73	L-->H
2B25B	73 - 77	73	L-->K
2B25B	73 - 77	73	L-->M
2B25B	73 - 77	73	L-->P
2B25B	73 - 77	73	L-->R
2B25B	73 - 77	73	L-->S
2B25B	73 - 77	74	E-->A
2B25B	73 - 77	74	E-->D
2B25B	73 - 77	74	E-->F
2B25B	73 - 77	74	E-->G
2B25B	73 - 77	74	E-->K
2B25B	73 - 77	74	E-->P

2B25B	73 - 77	74	E-->S
2B25B	73 - 77	74	E-->V
2B25B	73 - 77	74	E-->Y
2B25B	73 - 77	75	D-->I
2B25B	73 - 77	75	D-->R
2B25B	73 - 77	75	D-->T
2B25B	73 - 77	75	D-->V
2B25B	73 - 77	76	Y-->A
2B25B	73 - 77	76	Y-->D
2B25B	73 - 77	76	Y-->E
2B25B	73 - 77	76	Y-->F
2B25B	73 - 77	76	Y-->G
2B25B	73 - 77	76	Y-->I
2B25B	73 - 77	76	Y-->K
2B25B	73 - 77	76	Y-->L
2B25B	73 - 77	76	Y-->P
2B25B	73 - 77	76	Y-->R
2B25B	73 - 77	76	Y-->T
2B25B	73 - 77	77	V-->H
2B25B	73 - 77	77	V-->K
2B25B	73 - 77	77	V-->L
2B25B	73 - 77	77	V-->R
2B25B	73 - 77	77	V-->S
2B25B	73 - 77	77	V-->Y
1TD6A	101 - 105	101	A-->D
1TD6A	101 - 105	101	A-->E
1TD6A	101 - 105	101	A-->F

1TD6A	101 - 105	101	A-->G
1TD6A	101 - 105	101	A-->I
1TD6A	101 - 105	101	A-->K
1TD6A	101 - 105	101	A-->L
1TD6A	101 - 105	101	A-->M
1TD6A	101 - 105	101	A-->N
1TD6A	101 - 105	101	A-->P
1TD6A	101 - 105	101	A-->Q
1TD6A	101 - 105	101	A-->S
1TD6A	101 - 105	101	A-->V
1TD6A	101 - 105	102	A-->D
1TD6A	101 - 105	102	A-->E
1TD6A	101 - 105	102	A-->F
1TD6A	101 - 105	102	A-->G
1TD6A	101 - 105	102	A-->I
1TD6A	101 - 105	102	A-->L
1TD6A	101 - 105	102	A-->M
1TD6A	101 - 105	102	A-->N
1TD6A	101 - 105	102	A-->P
1TD6A	101 - 105	102	A-->R
1TD6A	101 - 105	102	A-->S
1TD6A	101 - 105	102	A-->T
1TD6A	101 - 105	102	A-->V
1TD6A	101 - 105	103	L-->A
1TD6A	101 - 105	103	L-->C
1TD6A	101 - 105	103	L-->D
1TD6A	101 - 105	103	L-->E

1TD6A	101 - 105	103	L-->G
1TD6A	101 - 105	103	L-->P
1TD6A	101 - 105	103	L-->Q
1TD6A	101 - 105	103	L-->R
1TD6A	101 - 105	103	L-->S
1TD6A	101 - 105	103	L-->T
1TD6A	101 - 105	103	L-->V
1TD6A	101 - 105	103	L-->Y
1TD6A	101 - 105	104	A-->F
1TD6A	101 - 105	104	A-->G
1TD6A	101 - 105	104	A-->H
1TD6A	101 - 105	104	A-->K
1TD6A	101 - 105	104	A-->L
1TD6A	101 - 105	104	A-->M
1TD6A	101 - 105	104	A-->P
1TD6A	101 - 105	104	A-->S
1TD6A	101 - 105	104	A-->V
1TD6A	101 - 105	105	S-->A
1TD6A	101 - 105	105	S-->D
1TD6A	101 - 105	105	S-->E
1TD6A	101 - 105	105	S-->F
1TD6A	101 - 105	105	S-->G
1TD6A	101 - 105	105	S-->H
1TD6A	101 - 105	105	S-->K
1TD6A	101 - 105	105	S-->L
1TD6A	101 - 105	105	S-->M
1TD6A	101 - 105	105	S-->P

1TD6A	101 - 105	105	S-->V
1TD6A	139 - 144	144	R-->A
1R9GA	53 - 58	53	R-->Y
1R9GA	53 - 58	54	R-->S
1R9GA	53 - 58	58	T-->G
1R9GA	80 - 85	80	A-->Y
1R9GA	80 - 85	81	G-->N
1R9GA	80 - 85	81	G-->Q
1R9GA	80 - 85	82	L-->D
1R9GA	80 - 85	82	L-->E
1R9GA	80 - 85	82	L-->Q
1R9GA	80 - 85	83	I-->A
1R9GA	80 - 85	83	I-->R
1R9GA	80 - 85	84	I-->A
1R9GA	80 - 85	84	I-->K
1R9GA	80 - 85	84	I-->L
1R9GA	80 - 85	84	I-->N
1R9GA	80 - 85	85	L-->S
1R9GA	187 - 191	187	V-->F
1R9GA	187 - 191	187	V-->M
1R9GA	187 - 191	187	V-->S
1R9GA	187 - 191	188	E-->D
1R9GA	187 - 191	188	E-->F
1R9GA	187 - 191	188	E-->G
1R9GA	187 - 191	188	E-->H
1R9GA	187 - 191	188	E-->I
1R9GA	187 - 191	188	E-->V

1R9GA	187 - 191	189	E-->D
1R9GA	187 - 191	189	E-->G
1R9GA	187 - 191	189	E-->L
1R9GA	187 - 191	189	E-->R
1R9GA	187 - 191	189	E-->T
1R9GA	187 - 191	190	Y-->D
1R9GA	187 - 191	190	Y-->F
1R9GA	187 - 191	190	Y-->I
1R9GA	187 - 191	190	Y-->L
1R9GA	187 - 191	190	Y-->P
1R9GA	187 - 191	190	Y-->R
1R9GA	187 - 191	190	Y-->S
1R9GA	187 - 191	190	Y-->V
1R9GA	187 - 191	191	K-->D
1R9GA	187 - 191	191	K-->E
1R9GA	187 - 191	191	K-->I
1R9GA	187 - 191	191	K-->N
1R9GA	187 - 191	191	K-->S
1R9GA	187 - 191	191	K-->T
1SKNP	474 - 479	475	F-->M
1UFQB	198 - 203	199	P-->A
1UFQB	198 - 203	199	P-->D
1UFQB	198 - 203	203	Y-->S
1JLSC	236 - 241	236	F-->N
1JLSC	236 - 241	237	G-->L
1JLSC	236 - 241	238	D-->V
1JLSC	236 - 241	240	Y-->D

2EMQA	77 - 81	77	F-->A
2EMQA	77 - 81	77	F-->D
2EMQA	77 - 81	77	F-->G
2EMQA	77 - 81	77	F-->I
2EMQA	77 - 81	77	F-->K
2EMQA	77 - 81	77	F-->L
2EMQA	77 - 81	77	F-->N
2EMQA	77 - 81	77	F-->P
2EMQA	77 - 81	77	F-->T
2EMQA	77 - 81	78	E-->A
2EMQA	77 - 81	78	E-->I
2EMQA	77 - 81	78	E-->L
2EMQA	77 - 81	78	E-->N
2EMQA	77 - 81	78	E-->S
2EMQA	77 - 81	78	E-->V
2EMQA	77 - 81	78	E-->W
2EMQA	77 - 81	79	R-->A
2EMQA	77 - 81	79	R-->C
2EMQA	77 - 81	79	R-->E
2EMQA	77 - 81	79	R-->G
2EMQA	77 - 81	79	R-->I
2EMQA	77 - 81	79	R-->K
2EMQA	77 - 81	79	R-->L
2EMQA	77 - 81	79	R-->P
2EMQA	77 - 81	80	L-->A
2EMQA	77 - 81	80	L-->D
2EMQA	77 - 81	80	L-->E

2EMQA	77	81	80	L-->F
2EMQA	77	81	80	L-->G
2EMQA	77	81	80	L-->I
2EMQA	77	81	80	L-->V
2EMQA	77	81	81	E-->D
2EMQA	77	81	81	E-->G
2EMQA	77	81	81	E-->S
1VRLA	68	73	69	E-->A
1VRLA	68	73	69	E-->L
1VRLA	68	73	70	A-->V
1VRLA	68	73	70	A-->W
1VRLA	68	73	71	L-->D
1VRLA	68	73	72	A-->F
1VRLA	114	118	114	P-->G
1VRLA	114	118	114	P-->N
1VRLA	114	118	114	P-->V
1VRLA	114	118	115	D-->A
1VRLA	114	118	115	D-->E
1VRLA	114	118	115	D-->M
1VRLA	114	118	115	D-->P
1VRLA	114	118	115	D-->S
1VRLA	114	118	115	D-->V
1VRLA	114	118	116	E-->A
1VRLA	114	118	116	E-->G
1VRLA	114	118	116	E-->I
1VRLA	114	118	116	E-->K
1VRLA	114	118	116	E-->L

1VRLA	114 - 118	116	E-->N	
1VRLA	114 - 118	116	E-->Q	
1VRLA	114 - 118	116	E-->R	
1VRLA	114 - 118	116	E-->S	
1VRLA	114 - 118	116	E-->Y	
1VRLA	114 - 118	117	F-->D	
1VRLA	114 - 118	117	F-->E	
1VRLA	114 - 118	117	F-->I	
1VRLA	114 - 118	117	F-->M	
1VRLA	114 - 118	117	F-->V	
1VRLA	114 - 118	117	F-->Y	
1VRLA	114 - 118	118	S-->D	
1VRLA	114 - 118	118	S-->I	
1VRLA	114 - 118	118	S-->L	
1VRLA	114 - 118	118	S-->N	
1VRLA	114 - 118	118	S-->P	
1VRLA	114 - 118	118	S-->R	
1VRLA	114 - 118	118	S-->V	
1VRLA	336 - 341	336	E-->P	
1VRLA	336 - 341	338	E-->V	
1QHHC	442 - 446	442	F-->A	
1QHHC	442 - 446	442	F-->G	
1QHHC	442 - 446	442	F-->L	
1QHHC	442 - 446	442	F-->M	
1QHHC	442 - 446	442	F-->Q	
1QHHC	442 - 446	442	F-->S	
1QHHC	442 - 446	442	F-->T	

1QHHC	442 - 446	442	F-->V
1QHHC	442 - 446	442	F-->Y
1QHHC	442 - 446	443	E-->A
1QHHC	442 - 446	443	E-->D
1QHHC	442 - 446	443	E-->F
1QHHC	442 - 446	443	E-->I
1QHHC	442 - 446	443	E-->K
1QHHC	442 - 446	443	E-->L
1QHHC	442 - 446	443	E-->P
1QHHC	442 - 446	443	E-->Q
1QHHC	442 - 446	443	E-->S
1QHHC	442 - 446	443	E-->T
1QHHC	442 - 446	443	E-->Y
1QHHC	442 - 446	444	A-->E
1QHHC	442 - 446	444	A-->F
1QHHC	442 - 446	444	A-->K
1QHHC	442 - 446	444	A-->L
1QHHC	442 - 446	444	A-->R
1QHHC	442 - 446	444	A-->V
1QHHC	442 - 446	445	L-->D
1QHHC	442 - 446	445	L-->E
1QHHC	442 - 446	445	L-->I
1QHHC	442 - 446	445	L-->K
1QHHC	442 - 446	445	L-->M
1QHHC	442 - 446	445	L-->N
1QHHC	442 - 446	445	L-->P
1QHHC	442 - 446	445	L-->R

1QHHC	442	446	445	L-->S
1QHHC	442	446	445	L-->T
1QHHC	442	446	445	L-->V
1QHHC	442	446	446	G-->C
1QHHC	442	446	446	G-->D
1QHHC	442	446	446	G-->E
1QHHC	442	446	446	G-->L
1QHHC	442	446	446	G-->P
1UT4A	32	36	32	L-->G
1UT4A	32	36	32	L-->W
1UT4A	32	36	33	C-->A
1UT4A	32	36	33	C-->E
1UT4A	32	36	33	C-->G
1UT4A	32	36	33	C-->K
1UT4A	32	36	33	C-->M
1UT4A	32	36	33	C-->N
1UT4A	32	36	33	C-->P
1UT4A	32	36	33	C-->Q
1UT4A	32	36	33	C-->S
1UT4A	32	36	33	C-->T
1UT4A	32	36	33	C-->Y
1UT4A	32	36	34	R-->F
1UT4A	32	36	34	R-->K
1UT4A	32	36	34	R-->P
1UT4A	32	36	34	R-->Q
1UT4A	32	36	34	R-->S
1UT4A	32	36	34	R-->V

1UT4A	32 - 36	35	K-->N
1UT4A	32 - 36	35	K-->S
1UT4A	32 - 36	35	K-->V
1UT4A	32 - 36	36	A-->P
1WYZC	24 - 29	24	Y-->A
1WYZC	24 - 29	26	T-->A
1WYZC	24 - 29	27	E-->S
1WYZC	70 - 76	71	E-->L
1WYZC	123 - 128	126	L-->D
1WYZC	175 - 180	177	R-->A
1XO1A	27 - 31	27	G-->A
1XO1A	27 - 31	27	G-->F
1XO1A	27 - 31	27	G-->L
1XO1A	27 - 31	27	G-->M
1XO1A	27 - 31	27	G-->P
1XO1A	27 - 31	27	G-->S
1XO1A	27 - 31	27	G-->T
1XO1A	27 - 31	27	G-->V
1XO1A	27 - 31	27	G-->Y
1XO1A	27 - 31	28	T-->A
1XO1A	27 - 31	28	T-->D
1XO1A	27 - 31	28	T-->E
1XO1A	27 - 31	28	T-->F
1XO1A	27 - 31	28	T-->G
1XO1A	27 - 31	28	T-->I
1XO1A	27 - 31	28	T-->L
1XO1A	27 - 31	28	T-->M

1XO1A	27 - 31	28	T-->N
1XO1A	27 - 31	28	T-->P
1XO1A	27 - 31	28	T-->R
1XO1A	27 - 31	28	T-->S
1XO1A	27 - 31	28	T-->V
1XO1A	27 - 31	28	T-->W
1XO1A	27 - 31	28	T-->Y
1XO1A	27 - 31	29	N-->A
1XO1A	27 - 31	29	N-->C
1XO1A	27 - 31	29	N-->D
1XO1A	27 - 31	29	N-->F
1XO1A	27 - 31	29	N-->G
1XO1A	27 - 31	29	N-->I
1XO1A	27 - 31	29	N-->K
1XO1A	27 - 31	29	N-->L
1XO1A	27 - 31	29	N-->P
1XO1A	27 - 31	29	N-->Q
1XO1A	27 - 31	29	N-->R
1XO1A	27 - 31	29	N-->V
1XO1A	27 - 31	29	N-->Y
1XO1A	27 - 31	30	L-->F
1XO1A	27 - 31	30	L-->G
1XO1A	27 - 31	30	L-->N
1XO1A	27 - 31	30	L-->P
1XO1A	27 - 31	30	L-->S
1XO1A	27 - 31	30	L-->T
1XO1A	27 - 31	30	L-->V

1XO1A	27	31	30	L-->W
1XO1A	27	31	31	G-->A
1XO1A	27	31	31	G-->E
1XO1A	27	31	31	G-->F
1XO1A	27	31	31	G-->H
1XO1A	27	31	31	G-->I
1XO1A	27	31	31	G-->L
1XO1A	27	31	31	G-->R
1XO1A	27	31	31	G-->S
1XO1A	27	31	31	G-->V
1XO1A	27	31	31	G-->Y
1XO1A	154	159	157	D-->I
1TIYB	104	108	104	H-->A
1TIYB	104	108	104	H-->C
1TIYB	104	108	104	H-->D
1TIYB	104	108	104	H-->E
1TIYB	104	108	104	H-->F
1TIYB	104	108	104	H-->G
1TIYB	104	108	104	H-->L
1TIYB	104	108	104	H-->P
1TIYB	104	108	104	H-->Q
1TIYB	104	108	104	H-->S
1TIYB	104	108	104	H-->T
1TIYB	104	108	104	H-->V
1TIYB	104	108	105	T-->I
1TIYB	104	108	105	T-->L
1TIYB	104	108	105	T-->N

1TIYB	104	108	105	T-->P
1TIYB	104	108	105	T-->V
1TIYB	104	108	105	T-->Y
1TIYB	104	108	106	D-->G
1TIYB	104	108	106	D-->H
1TIYB	104	108	106	D-->L
1TIYB	104	108	106	D-->N
1TIYB	104	108	106	D-->R
1TIYB	104	108	106	D-->S
1TIYB	104	108	106	D-->T
1TIYB	104	108	107	A-->F
1TIYB	104	108	107	A-->G
1TIYB	104	108	107	A-->I
1TIYB	104	108	107	A-->K
1TIYB	104	108	107	A-->S
1TIYB	104	108	107	A-->W
1TIYB	104	108	108	A-->I
1TIYB	104	108	108	A-->M
1TIYB	104	108	108	A-->V
1YBEA	9	14	9	T-->H
1YBEA	9	14	9	T-->V
1YBEA	9	14	10	K-->F
1YBEA	9	14	13	I-->V
1YBEA	110	117	115	W-->V
1YBEA	294	299	296	A-->K
1YBEA	294	299	298	L-->Y
1YBEA	294	299	299	R-->S

1YBEA	303 - 307	303	E-->G
1YBEA	303 - 307	303	E-->Q
1YBEA	303 - 307	303	E-->S
1YBEA	303 - 307	303	E-->Y
1YBEA	303 - 307	304	W-->A
1YBEA	303 - 307	304	W-->C
1YBEA	303 - 307	304	W-->E
1YBEA	303 - 307	304	W-->G
1YBEA	303 - 307	304	W-->I
1YBEA	303 - 307	304	W-->L
1YBEA	303 - 307	304	W-->M
1YBEA	303 - 307	304	W-->P
1YBEA	303 - 307	304	W-->Q
1YBEA	303 - 307	304	W-->T
1YBEA	303 - 307	304	W-->V
1YBEA	303 - 307	304	W-->Y
1YBEA	303 - 307	305	V-->G
1YBEA	303 - 307	305	V-->K
1YBEA	303 - 307	305	V-->L
1YBEA	303 - 307	305	V-->P
1YBEA	303 - 307	305	V-->Q
1YBEA	303 - 307	305	V-->R
1YBEA	303 - 307	305	V-->S
1YBEA	303 - 307	305	V-->T
1YBEA	303 - 307	306	A-->D
1YBEA	303 - 307	306	A-->S
1YBEA	303 - 307	307	D-->A

1YBEA	303	- 307	307	D-->C
1YBEA	303	- 307	307	D-->E
1YBEA	303	- 307	307	D-->F
1YBEA	303	- 307	307	D-->G
1YBEA	303	- 307	307	D-->I
1YBEA	303	- 307	307	D-->L
1YBEA	303	- 307	307	D-->M
1YBEA	303	- 307	307	D-->Q
1YBEA	303	- 307	307	D-->R
1YBEA	303	- 307	307	D-->S
1YBEA	303	- 307	307	D-->T
1YBEA	303	- 307	307	D-->V
1YBEA	303	- 307	307	D-->Y
2OL5A	60	- 64	60	P-->F
2OL5A	60	- 64	60	P-->L
2OL5A	60	- 64	61	Q-->E
2OL5A	60	- 64	61	Q-->G
2OL5A	60	- 64	61	Q-->V
2OL5A	60	- 64	62	W-->C
2OL5A	60	- 64	62	W-->D
2OL5A	60	- 64	62	W-->G
2OL5A	60	- 64	62	W-->I
2OL5A	60	- 64	62	W-->N
2OL5A	60	- 64	62	W-->P
2OL5A	60	- 64	62	W-->R
2OL5A	60	- 64	62	W-->S
2OL5A	60	- 64	63	N-->T

2OL5A	60	64	64	D-->C
2OL5A	60	64	64	D-->E
2OL5A	117	121	117	Q-->G
2OL5A	117	121	117	Q-->L
2OL5A	117	121	117	Q-->N
2OL5A	117	121	117	Q-->R
2OL5A	117	121	117	Q-->S
2OL5A	117	121	117	Q-->Y
2OL5A	117	121	118	S-->F
2OL5A	117	121	118	S-->H
2OL5A	117	121	119	L-->G
2OL5A	117	121	120	H-->L
2OL5A	117	121	120	H-->P
2OL5A	117	121	120	H-->R
2OL5A	117	121	121	D-->F
2OL5A	117	121	121	D-->G
2OL5A	117	121	121	D-->I
2OL5A	117	121	121	D-->T
2OL5A	117	121	121	D-->V
2OL5A	117	121	121	D-->Y
2R86A	56	61	58	E-->D
2R86A	56	61	58	E-->I
2R86A	56	61	59	L-->E
2R86A	56	61	59	L-->S
2R86A	56	61	60	L-->E
2R86A	56	61	61	N-->I
2R86A	72	76	72	F-->G

2R86A	72 - 76	72	F-->L
2R86A	72 - 76	72	F-->P
2R86A	72 - 76	72	F-->Q
2R86A	72 - 76	72	F-->R
2R86A	72 - 76	73	V-->N
2R86A	72 - 76	74	A-->D
2R86A	72 - 76	74	A-->G
2R86A	72 - 76	74	A-->M
2R86A	72 - 76	74	A-->N
2R86A	72 - 76	74	A-->P
2R86A	72 - 76	75	H-->A
2R86A	72 - 76	75	H-->D
2R86A	72 - 76	75	H-->E
2R86A	72 - 76	75	H-->G
2R86A	72 - 76	75	H-->N
2R86A	72 - 76	76	L-->E
2R86A	72 - 76	76	L-->S
2R86A	72 - 76	76	L-->V
2R86A	78 - 82	78	I-->A
2R86A	78 - 82	78	I-->D
2R86A	78 - 82	78	I-->G
2R86A	78 - 82	78	I-->K
2R86A	78 - 82	78	I-->L
2R86A	78 - 82	78	I-->M
2R86A	78 - 82	78	I-->N
2R86A	78 - 82	78	I-->P
2R86A	78 - 82	78	I-->R

2R86A	78 - 82	78	I-->T
2R86A	78 - 82	78	I-->V
2R86A	78 - 82	79	E-->A
2R86A	78 - 82	79	E-->C
2R86A	78 - 82	79	E-->D
2R86A	78 - 82	79	E-->F
2R86A	78 - 82	79	E-->K
2R86A	78 - 82	79	E-->L
2R86A	78 - 82	79	E-->M
2R86A	78 - 82	79	E-->N
2R86A	78 - 82	79	E-->R
2R86A	78 - 82	79	E-->T
2R86A	78 - 82	80	L-->A
2R86A	78 - 82	80	L-->D
2R86A	78 - 82	80	L-->E
2R86A	78 - 82	80	L-->F
2R86A	78 - 82	80	L-->G
2R86A	78 - 82	80	L-->K
2R86A	78 - 82	80	L-->P
2R86A	78 - 82	80	L-->R
2R86A	78 - 82	80	L-->T
2R86A	78 - 82	80	L-->V
2R86A	78 - 82	80	L-->W
2R86A	78 - 82	81	V-->D
2R86A	78 - 82	81	V-->I
2R86A	78 - 82	81	V-->K
2R86A	78 - 82	81	V-->M

2R86A	78	82	81	V-->N
2R86A	78	82	81	V-->P
2R86A	78	82	81	V-->T
2R86A	78	82	81	V-->W
2R86A	78	82	82	E-->D
2R86A	78	82	82	E-->G
2R86A	78	82	82	E-->I
2R86A	78	82	82	E-->K
2R86A	78	82	82	E-->L
2R86A	78	82	82	E-->N
2R86A	78	82	82	E-->P
2R86A	78	82	82	E-->R
2R86A	78	82	82	E-->S
2R86A	78	82	82	E-->V
2R86A	78	82	82	E-->W
2R86A	78	82	82	E-->Y
2R86A	94	99	94	V-->T
2R86A	94	99	97	W-->R
2R86A	215	219	215	A-->C
2R86A	215	219	215	A-->D
2R86A	215	219	215	A-->L
2R86A	215	219	215	A-->M
2R86A	215	219	215	A-->P
2R86A	215	219	215	A-->Q
2R86A	215	219	215	A-->R
2R86A	215	219	215	A-->T
2R86A	215	219	216	K-->A

2R86A	215	219	216	K-->D
2R86A	215	219	216	K-->L
2R86A	215	219	216	K-->V
2R86A	215	219	217	D-->E
2R86A	215	219	217	D-->G
2R86A	215	219	217	D-->I
2R86A	215	219	217	D-->K
2R86A	215	219	217	D-->L
2R86A	215	219	217	D-->Q
2R86A	215	219	217	D-->R
2R86A	215	219	217	D-->S
2R86A	215	219	217	D-->T
2R86A	215	219	217	D-->W
2R86A	215	219	217	D-->Y
2R86A	215	219	218	Q-->A
2R86A	215	219	218	Q-->E
2R86A	215	219	218	Q-->F
2R86A	215	219	218	Q-->G
2R86A	215	219	218	Q-->K
2R86A	215	219	218	Q-->L
2R86A	215	219	218	Q-->M
2R86A	215	219	218	Q-->N
2R86A	215	219	218	Q-->S
2R86A	215	219	218	Q-->T
2R86A	215	219	218	Q-->Y
2R86A	215	219	219	L-->E
2R86A	290	295	290	A-->D

2R86A	290 - 295	294	I-->N
2R86A	290 - 295	294	I-->Y
2R86A	290 - 295	295	F-->L
2R86A	290 - 295	295	F-->T
1YLFA	49 - 53	49	S-->A
1YLFA	49 - 53	49	S-->L
1YLFA	49 - 53	50	Y-->D
1YLFA	49 - 53	50	Y-->H
1YLFA	49 - 53	50	Y-->K
1YLFA	49 - 53	50	Y-->P
1YLFA	49 - 53	51	L-->A
1YLFA	49 - 53	51	L-->K
1YLFA	49 - 53	51	L-->R
1YLFA	49 - 53	52	K-->D
1YLFA	49 - 53	52	K-->F
1YLFA	49 - 53	52	K-->H
1YLFA	49 - 53	52	K-->I
1YLFA	49 - 53	52	K-->N
1YLFA	49 - 53	52	K-->V
1YLFA	49 - 53	53	Q-->A
1YLFA	49 - 53	53	Q-->C
1YLFA	49 - 53	53	Q-->G
1YLFA	49 - 53	53	Q-->K
1YLFA	49 - 53	53	Q-->R
1YLFA	49 - 53	53	Q-->S
1YLFA	49 - 53	53	Q-->V
1YLFA	49 - 53	53	Q-->Y

1YLFA	125 - 129	125	E-->A
1YLFA	125 - 129	125	E-->G
1YLFA	125 - 129	125	E-->I
1YLFA	125 - 129	125	E-->M
1YLFA	125 - 129	125	E-->P
1YLFA	125 - 129	125	E-->S
1YLFA	125 - 129	125	E-->V
1YLFA	125 - 129	126	E-->I
1YLFA	125 - 129	126	E-->L
1YLFA	125 - 129	126	E-->M
1YLFA	125 - 129	126	E-->P
1YLFA	125 - 129	126	E-->Q
1YLFA	125 - 129	126	E-->V
1YLFA	125 - 129	127	V-->A
1YLFA	125 - 129	127	V-->D
1YLFA	125 - 129	127	V-->G
1YLFA	125 - 129	127	V-->K
1YLFA	125 - 129	127	V-->N
1YLFA	125 - 129	127	V-->P
1YLFA	125 - 129	127	V-->T
1YLFA	125 - 129	127	V-->Y
1YLFA	125 - 129	128	L-->C
1YLFA	125 - 129	128	L-->E
1YLFA	125 - 129	128	L-->I
1YLFA	125 - 129	128	L-->P
1YLFA	125 - 129	128	L-->R
1YLFA	125 - 129	129	R-->A

1YLFA	125	- 129	129	R-->D
1YLFA	125	- 129	129	R-->E
1YLFA	125	- 129	129	R-->F
1YLFA	125	- 129	129	R-->G
1YLFA	125	- 129	129	R-->I
1YLFA	125	- 129	129	R-->L
1YLFA	125	- 129	129	R-->M
1YLFA	125	- 129	129	R-->P
1YLFA	125	- 129	129	R-->Q
1YLFA	125	- 129	129	R-->V
1YLFA	125	- 129	129	R-->W
2HREA	355	- 360	360	C-->E
2HREA	355	- 360	360	C-->R
2Q37A	17	- 22	20	A-->Y
2Q37A	148	- 153	149	I-->D
2Q37A	148	- 153	149	I-->G
2Q37A	148	- 153	150	T-->L
2Q37A	148	- 153	151	E-->L
2Q37A	148	- 153	153	R-->D
2Q37A	148	- 153	153	R-->E
1T5YA	6	- 11	6	E-->S
1T5YA	6	- 11	7	E-->A
1T5YA	6	- 11	7	E-->P
1T5YA	6	- 11	8	E-->T
1T5YA	6	- 11	9	T-->C
1T5YA	6	- 11	9	T-->I
1T5YA	6	- 11	10	R-->D

1T5YA	24	28	24	L-->A
1T5YA	24	28	24	L-->E
1T5YA	24	28	24	L-->G
1T5YA	24	28	24	L-->H
1T5YA	24	28	24	L-->N
1T5YA	24	28	24	L-->P
1T5YA	24	28	24	L-->Q
1T5YA	24	28	24	L-->T
1T5YA	24	28	24	L-->V
1T5YA	24	28	24	L-->W
1T5YA	24	28	25	Q-->A
1T5YA	24	28	25	Q-->D
1T5YA	24	28	25	Q-->E
1T5YA	24	28	25	Q-->F
1T5YA	24	28	25	Q-->G
1T5YA	24	28	25	Q-->K
1T5YA	24	28	25	Q-->L
1T5YA	24	28	25	Q-->N
1T5YA	24	28	25	Q-->P
1T5YA	24	28	25	Q-->R
1T5YA	24	28	25	Q-->S
1T5YA	24	28	25	Q-->T
1T5YA	24	28	25	Q-->Y
1T5YA	24	28	26	L-->E
1T5YA	24	28	26	L-->G
1T5YA	24	28	26	L-->H
1T5YA	24	28	26	L-->S

1T5YA	24 - 28	26	L-->V
1T5YA	24 - 28	27	L-->A
1T5YA	24 - 28	27	L-->C
1T5YA	24 - 28	27	L-->D
1T5YA	24 - 28	27	L-->F
1T5YA	24 - 28	27	L-->G
1T5YA	24 - 28	27	L-->K
1T5YA	24 - 28	27	L-->M
1T5YA	24 - 28	27	L-->N
1T5YA	24 - 28	27	L-->P
1T5YA	24 - 28	27	L-->R
1T5YA	24 - 28	27	L-->V
1T5YA	24 - 28	27	L-->W
1T5YA	24 - 28	28	V-->A
1T5YA	24 - 28	28	V-->E
1T5YA	24 - 28	28	V-->G
1T5YA	24 - 28	28	V-->L
1T5YA	24 - 28	28	V-->P
1T5YA	24 - 28	28	V-->S
1T5YA	150 - 155	154	R-->N
2HJWA	301 - 305	301	P-->E
2HJWA	301 - 305	301	P-->G
2HJWA	301 - 305	301	P-->I
2HJWA	301 - 305	301	P-->N
2HJWA	301 - 305	301	P-->S
2HJWA	301 - 305	301	P-->T
2HJWA	301 - 305	301	P-->V

2HJWA	301 - 305	301	P-->Y
2HJWA	301 - 305	302	E-->F
2HJWA	301 - 305	302	E-->M
2HJWA	301 - 305	302	E-->N
2HJWA	301 - 305	302	E-->P
2HJWA	301 - 305	302	E-->Q
2HJWA	301 - 305	302	E-->R
2HJWA	301 - 305	302	E-->S
2HJWA	301 - 305	302	E-->T
2HJWA	301 - 305	302	E-->V
2HJWA	301 - 305	303	D-->E
2HJWA	301 - 305	303	D-->F
2HJWA	301 - 305	303	D-->G
2HJWA	301 - 305	303	D-->H
2HJWA	301 - 305	303	D-->I
2HJWA	301 - 305	303	D-->L
2HJWA	301 - 305	303	D-->M
2HJWA	301 - 305	303	D-->N
2HJWA	301 - 305	303	D-->Q
2HJWA	301 - 305	303	D-->R
2HJWA	301 - 305	303	D-->T
2HJWA	301 - 305	303	D-->V
2HJWA	301 - 305	303	D-->Y
2HJWA	301 - 305	304	L-->D
2HJWA	301 - 305	304	L-->E
2HJWA	301 - 305	304	L-->F
2HJWA	301 - 305	304	L-->G

2HJWA	301 - 305	304	L-->K
2HJWA	301 - 305	304	L-->P
2HJWA	301 - 305	304	L-->R
2HJWA	301 - 305	304	L-->S
2HJWA	301 - 305	304	L-->V
2HJWA	301 - 305	305	K-->A
2HJWA	301 - 305	305	K-->D
2HJWA	301 - 305	305	K-->E
2HJWA	301 - 305	305	K-->G
2HJWA	301 - 305	305	K-->L
2HJWA	301 - 305	305	K-->M
2HJWA	301 - 305	305	K-->N
2HJWA	301 - 305	305	K-->P
2HJWA	301 - 305	305	K-->S
2HJWA	301 - 305	305	K-->T
2HJWA	301 - 305	305	K-->V
2HJWA	309 - 313	310	Y-->S
2HJWA	309 - 313	310	Y-->T
2HJWA	309 - 313	311	I-->A
2HJWA	309 - 313	312	K-->R
2HJWA	309 - 313	313	M-->D
2HJWA	309 - 313	313	M-->E
2HJWA	309 - 313	313	M-->L
2HJWA	309 - 313	313	M-->T
2HJWA	376 - 380	377	E-->F
2HJWA	376 - 380	377	E-->R
2HJWA	376 - 380	379	M-->G

2HJWA	376	380	380	W-->H
2HJWA	617	622	617	K-->G
2HJWA	617	622	619	I-->L
2HJWA	617	622	620	R-->A
2HJWA	617	622	620	R-->K
2HJWA	617	622	620	R-->L
2HJWA	617	622	620	R-->Q
2HJWA	617	622	621	L-->N
1GUOC	41	46	44	V-->D
1GUOC	41	46	44	V-->E
1GUOC	41	46	45	E-->F
1GUOC	41	46	45	E-->I
1GUOC	41	46	46	E-->C
1GUOC	41	46	46	E-->I
2HTIA	109	115	109	I-->F
2HTIA	109	115	109	I-->N
1AIHB	208	212	208	W-->D
1AIHB	208	212	208	W-->E
1AIHB	208	212	208	W-->F
1AIHB	208	212	208	W-->G
1AIHB	208	212	208	W-->K
1AIHB	208	212	208	W-->M
1AIHB	208	212	208	W-->N
1AIHB	208	212	208	W-->P
1AIHB	208	212	208	W-->Q
1AIHB	208	212	208	W-->S
1AIHB	208	212	209	S-->F

1AIHB	208 - 212	209	S-->L
1AIHB	208 - 212	209	S-->P
1AIHB	208 - 212	209	S-->V
1AIHB	208 - 212	210	E-->I
1AIHB	208 - 212	210	E-->S
1AIHB	208 - 212	212	E-->G
1AIHB	208 - 212	212	E-->N
1AIHB	208 - 212	212	E-->R
1AIHB	208 - 212	212	E-->V
1AIHB	309 - 313	311	M-->I
1AIHB	309 - 313	311	M-->V
1AIHB	309 - 313	313	M-->N
1R4WA	90 - 94	90	E-->I
1R4WA	90 - 94	90	E-->L
1R4WA	90 - 94	90	E-->T
1R4WA	90 - 94	90	E-->V
1R4WA	90 - 94	91	H-->D
1R4WA	90 - 94	91	H-->E
1R4WA	90 - 94	91	H-->G
1R4WA	90 - 94	91	H-->I
1R4WA	90 - 94	91	H-->K
1R4WA	90 - 94	91	H-->L
1R4WA	90 - 94	91	H-->N
1R4WA	90 - 94	91	H-->Q
1R4WA	90 - 94	91	H-->R
1R4WA	90 - 94	91	H-->V
1R4WA	90 - 94	91	H-->Y

1R4WA	90 - 94	92	V-->G
1R4WA	90 - 94	92	V-->R
1R4WA	90 - 94	93	K-->T
1R4WA	90 - 94	93	K-->Y
1R4WA	90 - 94	94	K-->F
1R4WA	90 - 94	94	K-->S
1R4WA	90 - 94	94	K-->Y
1NW3A	122 - 126	122	P-->D
1NW3A	122 - 126	122	P-->E
1NW3A	122 - 126	122	P-->G
1NW3A	122 - 126	122	P-->L
1NW3A	122 - 126	122	P-->R
1NW3A	122 - 126	122	P-->S
1NW3A	122 - 126	122	P-->T
1NW3A	122 - 126	122	P-->Y
1NW3A	122 - 126	123	E-->A
1NW3A	122 - 126	123	E-->K
1NW3A	122 - 126	123	E-->L
1NW3A	122 - 126	123	E-->M
1NW3A	122 - 126	123	E-->S
1NW3A	122 - 126	123	E-->V
1NW3A	122 - 126	124	K-->D
1NW3A	122 - 126	124	K-->E
1NW3A	122 - 126	124	K-->I
1NW3A	122 - 126	124	K-->N
1NW3A	122 - 126	124	K-->R
1NW3A	122 - 126	124	K-->T

1NW3A	122	- 126	125	L-->D
1NW3A	122	- 126	125	L-->G
1NW3A	122	- 126	125	L-->Q
1NW3A	122	- 126	125	L-->V
1NW3A	122	- 126	126	N-->D
1NW3A	122	- 126	126	N-->E
1NW3A	122	- 126	126	N-->G
1NW3A	122	- 126	126	N-->P
1NW3A	122	- 126	126	N-->Q
1NW3A	122	- 126	126	N-->R
1NW3A	122	- 126	126	N-->S
1NW3A	122	- 126	126	N-->Y
1GU6A	74	- 78	74	R-->A
1GU6A	74	- 78	74	R-->G
1GU6A	74	- 78	74	R-->L
1GU6A	74	- 78	74	R-->N
1GU6A	74	- 78	74	R-->P
1GU6A	74	- 78	74	R-->T
1GU6A	74	- 78	74	R-->V
1GU6A	74	- 78	74	R-->W
1GU6A	74	- 78	75	L-->A
1GU6A	74	- 78	75	L-->D
1GU6A	74	- 78	75	L-->E
1GU6A	74	- 78	75	L-->G
1GU6A	74	- 78	75	L-->I
1GU6A	74	- 78	75	L-->K
1GU6A	74	- 78	75	L-->M

1GU6A	74 - 78	75	L-->N	
1GU6A	74 - 78	75	L-->P	
1GU6A	74 - 78	75	L-->Q	
1GU6A	74 - 78	75	L-->R	
1GU6A	74 - 78	75	L-->S	
1GU6A	74 - 78	75	L-->V	
1GU6A	74 - 78	76	V-->A	
1GU6A	74 - 78	76	V-->C	
1GU6A	74 - 78	76	V-->E	
1GU6A	74 - 78	76	V-->P	
1GU6A	74 - 78	76	V-->Q	
1GU6A	74 - 78	76	V-->R	
1GU6A	74 - 78	76	V-->T	
1GU6A	74 - 78	77	I-->A	
1GU6A	74 - 78	77	I-->D	
1GU6A	74 - 78	77	I-->G	
1GU6A	74 - 78	77	I-->H	
1GU6A	74 - 78	77	I-->K	
1GU6A	74 - 78	77	I-->L	
1GU6A	74 - 78	77	I-->N	
1GU6A	74 - 78	77	I-->P	
1GU6A	74 - 78	77	I-->R	
1GU6A	74 - 78	77	I-->S	
1GU6A	74 - 78	77	I-->V	
1GU6A	74 - 78	77	I-->Y	
1GU6A	74 - 78	78	L-->A	
1GU6A	74 - 78	78	L-->D	

1GU6A	74	78	78	L-->E
1GU6A	74	78	78	L-->G
1GU6A	74	78	78	L-->H
1GU6A	74	78	78	L-->M
1GU6A	74	78	78	L-->P
1GU6A	74	78	78	L-->Q
1GU6A	74	78	78	L-->R
1GU6A	74	78	78	L-->T
1GU6A	282	286	282	C-->H
1GU6A	282	286	283	I-->L
1GU6A	282	286	284	D-->G
1GU6A	282	286	284	D-->Q
1GU6A	282	286	285	C-->S
1GU6A	282	286	286	H-->A
1GU6A	282	286	286	H-->G
1EWAA	29	34	32	R-->D
1EWAA	29	34	33	R-->G
1EWAA	29	34	33	R-->Y
1EWAA	43	48	47	K-->D
1EWAA	43	48	48	S-->G
1JB0J	2	7	3	H-->Y
2Q83A	181	185	181	E-->A
2Q83A	181	185	181	E-->F
2Q83A	181	185	181	E-->G
2Q83A	181	185	181	E-->N
2Q83A	181	185	181	E-->P
2Q83A	181	185	181	E-->R

2Q83A	181 - 185	181	E-->T
2Q83A	181 - 185	182	T-->D
2Q83A	181 - 185	182	T-->E
2Q83A	181 - 185	182	T-->P
2Q83A	181 - 185	182	T-->V
2Q83A	181 - 185	183	W-->G
2Q83A	181 - 185	183	W-->I
2Q83A	181 - 185	183	W-->K
2Q83A	181 - 185	183	W-->Q
2Q83A	181 - 185	183	W-->S
2Q83A	181 - 185	183	W-->T
2Q83A	181 - 185	183	W-->V
2Q83A	181 - 185	184	K-->D
2Q83A	181 - 185	184	K-->I
2Q83A	181 - 185	184	K-->Q
2Q83A	181 - 185	184	K-->Y
2Q83A	181 - 185	185	L-->G
2Q83A	267 - 272	267	I-->Y
2Q83A	267 - 272	270	L-->T
2Q83A	267 - 272	271	R-->T
2Q83A	267 - 272	272	K-->L
2Q83A	286 - 291	287	E-->W
2Q83A	286 - 291	289	F-->R
2Q83A	293 - 299	297	E-->T
2Q83A	304 - 309	305	E-->G
2Q83A	304 - 309	305	E-->K
2Q83A	304 - 309	308	Q-->T

2OU4B	196	201	198	D-->R
2OU4B	254	258	254	S-->A
2OU4B	254	258	254	S-->C
2OU4B	254	258	254	S-->G
2OU4B	254	258	254	S-->I
2OU4B	254	258	254	S-->Q
2OU4B	254	258	254	S-->V
2OU4B	254	258	255	V-->A
2OU4B	254	258	255	V-->C
2OU4B	254	258	255	V-->D
2OU4B	254	258	255	V-->G
2OU4B	254	258	255	V-->L
2OU4B	254	258	255	V-->M
2OU4B	254	258	255	V-->R
2OU4B	254	258	255	V-->S
2OU4B	254	258	256	S-->D
2OU4B	254	258	256	S-->F
2OU4B	254	258	256	S-->G
2OU4B	254	258	256	S-->L
2OU4B	254	258	256	S-->N
2OU4B	254	258	256	S-->R
2OU4B	254	258	256	S-->T
2OU4B	254	258	256	S-->V
2OU4B	254	258	257	R-->A
2OU4B	254	258	257	R-->D
2OU4B	254	258	257	R-->E
2OU4B	254	258	257	R-->G

2OU4B	254	258	257	R-->K
2OU4B	254	258	257	R-->L
2OU4B	254	258	257	R-->P
2OU4B	254	258	257	R-->S
2OU4B	254	258	257	R-->T
2OU4B	254	258	257	R-->V
2OU4B	254	258	257	R-->W
2OU4B	254	258	258	A-->E
2OU4B	254	258	258	A-->G
2OU4B	254	258	258	A-->H
2OU4B	254	258	258	A-->K
2OU4B	254	258	258	A-->L
2OU4B	254	258	258	A-->N
2OU4B	254	258	258	A-->P
2OU4B	254	258	258	A-->Q
2OU4B	254	258	258	A-->R
2OU4B	254	258	258	A-->S
2OU4B	254	258	258	A-->T
2OU4B	254	258	258	A-->V
1JB0L	60	65	62	W-->A
1JB0L	60	65	62	W-->S
1JB0L	60	65	65	L-->A
1NHEB	298	303	298	K-->R
1NHEB	298	303	299	Q-->P
1NHEB	298	303	300	Q-->L
2G36A	15	19	15	I-->A
2G36A	15	19	15	I-->D

2G36A	15 - 19	15	I-->G
2G36A	15 - 19	15	I-->K
2G36A	15 - 19	15	I-->L
2G36A	15 - 19	15	I-->N
2G36A	15 - 19	15	I-->Q
2G36A	15 - 19	15	I-->S
2G36A	15 - 19	15	I-->T
2G36A	15 - 19	15	I-->V
2G36A	15 - 19	16	G-->H
2G36A	15 - 19	16	G-->K
2G36A	15 - 19	16	G-->L
2G36A	15 - 19	16	G-->N
2G36A	15 - 19	17	H-->A
2G36A	15 - 19	17	H-->D
2G36A	15 - 19	17	H-->E
2G36A	15 - 19	17	H-->G
2G36A	15 - 19	17	H-->K
2G36A	15 - 19	17	H-->L
2G36A	15 - 19	17	H-->M
2G36A	15 - 19	17	H-->N
2G36A	15 - 19	17	H-->P
2G36A	15 - 19	17	H-->R
2G36A	15 - 19	17	H-->S
2G36A	15 - 19	17	H-->V
2G36A	15 - 19	18	L-->F
2G36A	15 - 19	18	L-->I
2G36A	15 - 19	18	L-->K

2G36A	15 - 19	18	L-->P
2G36A	15 - 19	18	L-->R
2G36A	15 - 19	18	L-->S
2G36A	15 - 19	18	L-->V
2G36A	15 - 19	19	V-->A
2G36A	15 - 19	19	V-->C
2G36A	15 - 19	19	V-->D
2G36A	15 - 19	19	V-->I
2G36A	15 - 19	19	V-->K
2G36A	15 - 19	19	V-->L
2G36A	100 - 104	100	V-->C
2G36A	100 - 104	100	V-->E
2G36A	100 - 104	100	V-->F
2G36A	100 - 104	100	V-->G
2G36A	100 - 104	100	V-->I
2G36A	100 - 104	100	V-->N
2G36A	100 - 104	100	V-->P
2G36A	100 - 104	100	V-->Y
2G36A	100 - 104	101	S-->D
2G36A	100 - 104	101	S-->F
2G36A	100 - 104	101	S-->G
2G36A	100 - 104	101	S-->I
2G36A	100 - 104	101	S-->L
2G36A	100 - 104	101	S-->M
2G36A	100 - 104	101	S-->P
2G36A	100 - 104	101	S-->Q
2G36A	100 - 104	101	S-->R

2G36A	100 - 104	101	S-->T
2G36A	100 - 104	101	S-->V
2G36A	100 - 104	101	S-->W
2G36A	100 - 104	101	S-->Y
2G36A	100 - 104	102	R-->E
2G36A	100 - 104	102	R-->G
2G36A	100 - 104	102	R-->I
2G36A	100 - 104	102	R-->L
2G36A	100 - 104	102	R-->N
2G36A	100 - 104	102	R-->Q
2G36A	100 - 104	102	R-->S
2G36A	100 - 104	102	R-->V
2G36A	100 - 104	102	R-->W
2G36A	100 - 104	103	L-->D
2G36A	100 - 104	103	L-->E
2G36A	100 - 104	103	L-->P
2G36A	100 - 104	103	L-->Q
2G36A	100 - 104	103	L-->R
2G36A	100 - 104	103	L-->T
2G36A	100 - 104	103	L-->V
2G36A	100 - 104	104	E-->D
2G36A	100 - 104	104	E-->F
2G36A	100 - 104	104	E-->G
2G36A	100 - 104	104	E-->H
2G36A	100 - 104	104	E-->I
2G36A	100 - 104	104	E-->T
2G36A	100 - 104	104	E-->V

2OGGA	187 - 192	189	D-->A
2OGGA	187 - 192	191	E-->I
2OGGA	187 - 192	192	L-->N
1ZTPB	206 - 210	206	D-->E
1ZTPB	206 - 210	206	D-->G
1ZTPB	206 - 210	206	D-->K
1ZTPB	206 - 210	206	D-->L
1ZTPB	206 - 210	206	D-->R
1ZTPB	206 - 210	207	V-->D
1ZTPB	206 - 210	207	V-->E
1ZTPB	206 - 210	207	V-->H
1ZTPB	206 - 210	207	V-->I
1ZTPB	206 - 210	207	V-->N
1ZTPB	206 - 210	207	V-->P
1ZTPB	206 - 210	207	V-->S
1ZTPB	206 - 210	207	V-->T
1ZTPB	206 - 210	208	Y-->A
1ZTPB	206 - 210	208	Y-->F
1ZTPB	206 - 210	208	Y-->I
1ZTPB	206 - 210	208	Y-->N
1ZTPB	206 - 210	208	Y-->R
1ZTPB	206 - 210	208	Y-->V
1ZTPB	206 - 210	209	T-->G
1ZTPB	206 - 210	209	T-->I
1ZTPB	206 - 210	209	T-->K
1ZTPB	206 - 210	209	T-->P
1ZTPB	206 - 210	209	T-->V

1ZTPB	206 - 210	209	T-->W
1ZTPB	206 - 210	210	Y-->A
1ZTPB	206 - 210	210	Y-->C
1ZTPB	206 - 210	210	Y-->D
1ZTPB	206 - 210	210	Y-->E
1ZTPB	206 - 210	210	Y-->F
1ZTPB	206 - 210	210	Y-->G
1ZTPB	206 - 210	210	Y-->H
1ZTPB	206 - 210	210	Y-->I
1ZTPB	206 - 210	210	Y-->K
1ZTPB	206 - 210	210	Y-->N
1ZTPB	206 - 210	210	Y-->P
1ZTPB	206 - 210	210	Y-->Q
1ZTPB	206 - 210	210	Y-->R
1ZTPB	206 - 210	210	Y-->S
1ZTPB	206 - 210	210	Y-->T
1ZTPB	206 - 210	210	Y-->V
2HDIB	285 - 291	285	P-->R
2HDIB	285 - 291	291	V-->E
1POIB	138 - 144	140	G-->I
1POIB	218 - 223	219	E-->I
1POIB	218 - 223	219	E-->T
1POIB	218 - 223	220	D-->L
1POIB	218 - 223	220	D-->W
1Y5NC	116 - 120	116	P-->A
1Y5NC	116 - 120	116	P-->C
1Y5NC	116 - 120	116	P-->D

1Y5NC	116 - 120	116	P-->E
1Y5NC	116 - 120	116	P-->G
1Y5NC	116 - 120	116	P-->I
1Y5NC	116 - 120	116	P-->R
1Y5NC	116 - 120	116	P-->S
1Y5NC	116 - 120	116	P-->T
1Y5NC	116 - 120	117	R-->A
1Y5NC	116 - 120	117	R-->D
1Y5NC	116 - 120	117	R-->E
1Y5NC	116 - 120	117	R-->G
1Y5NC	116 - 120	117	R-->I
1Y5NC	116 - 120	117	R-->L
1Y5NC	116 - 120	117	R-->Y
1Y5NC	116 - 120	118	V-->A
1Y5NC	116 - 120	118	V-->K
1Y5NC	116 - 120	118	V-->L
1Y5NC	116 - 120	118	V-->T
1Y5NC	116 - 120	118	V-->W
1Y5NC	116 - 120	119	R-->A
1Y5NC	116 - 120	119	R-->E
1Y5NC	116 - 120	119	R-->F
1Y5NC	116 - 120	119	R-->G
1Y5NC	116 - 120	119	R-->I
1Y5NC	116 - 120	119	R-->K
1Y5NC	116 - 120	119	R-->L
1Y5NC	116 - 120	119	R-->P
1Y5NC	116 - 120	119	R-->T

1Y5NC	116 - 120	120	A-->F
1Y5NC	116 - 120	120	A-->G
1Y5NC	116 - 120	120	A-->I
1Y5NC	116 - 120	120	A-->K
1Y5NC	116 - 120	120	A-->L
1Y5NC	116 - 120	120	A-->M
1Y5NC	116 - 120	120	A-->R
1Y5NC	116 - 120	120	A-->S
1Y5NC	116 - 120	120	A-->Y
1Y5NC	211 - 215	211	V-->D
1Y5NC	211 - 215	211	V-->E
1Y5NC	211 - 215	211	V-->I
1Y5NC	211 - 215	211	V-->K
1Y5NC	211 - 215	211	V-->N
1Y5NC	211 - 215	211	V-->R
1Y5NC	211 - 215	211	V-->T
1Y5NC	211 - 215	212	E-->G
1Y5NC	211 - 215	212	E-->K
1Y5NC	211 - 215	212	E-->R
1Y5NC	211 - 215	212	E-->S
1Y5NC	211 - 215	212	E-->Y
1Y5NC	211 - 215	213	Y-->A
1Y5NC	211 - 215	213	Y-->C
1Y5NC	211 - 215	213	Y-->D
1Y5NC	211 - 215	213	Y-->E
1Y5NC	211 - 215	213	Y-->G
1Y5NC	211 - 215	213	Y-->K

1Y5NC	211	- 215	213	Y-->L
1Y5NC	211	- 215	213	Y-->N
1Y5NC	211	- 215	213	Y-->P
1Y5NC	211	- 215	213	Y-->R
1Y5NC	211	- 215	213	Y-->V
1Y5NC	211	- 215	214	L-->D
1Y5NC	211	- 215	214	L-->E
1Y5NC	211	- 215	214	L-->F
1Y5NC	211	- 215	214	L-->I
1Y5NC	211	- 215	214	L-->K
1Y5NC	211	- 215	214	L-->P
1Y5NC	211	- 215	214	L-->R
1Y5NC	211	- 215	214	L-->S
1Y5NC	211	- 215	214	L-->Y
1Y5NC	211	- 215	215	T-->D
1Y5NC	211	- 215	215	T-->G
1Y5NC	211	- 215	215	T-->K
1Y5NC	211	- 215	215	T-->L
1Y5NC	211	- 215	215	T-->N
1Y5NC	211	- 215	215	T-->P
1Y5NC	211	- 215	215	T-->Q
1Y5NC	211	- 215	215	T-->S
1Y5NC	211	- 215	215	T-->V
1Y5NC	211	- 215	215	T-->Y
2QTXA	19	- 23	19	Y-->A
2QTXA	19	- 23	19	Y-->G
2QTXA	19	- 23	19	Y-->I

2QTXA	19	- 23	19	Y-->M
2QTXA	19	- 23	19	Y-->N
2QTXA	19	- 23	19	Y-->P
2QTXA	19	- 23	19	Y-->S
2QTXA	19	- 23	20	A-->D
2QTXA	19	- 23	20	A-->G
2QTXA	19	- 23	20	A-->I
2QTXA	19	- 23	20	A-->L
2QTXA	19	- 23	20	A-->N
2QTXA	19	- 23	20	A-->P
2QTXA	19	- 23	20	A-->R
2QTXA	19	- 23	20	A-->S
2QTXA	19	- 23	20	A-->Y
2QTXA	19	- 23	21	R-->A
2QTXA	19	- 23	21	R-->D
2QTXA	19	- 23	21	R-->G
2QTXA	19	- 23	21	R-->L
2QTXA	19	- 23	21	R-->M
2QTXA	19	- 23	21	R-->P
2QTXA	19	- 23	21	R-->V
2QTXA	19	- 23	22	R-->A
2QTXA	19	- 23	22	R-->S
2QTXA	19	- 23	22	R-->W
2QTXA	19	- 23	23	L-->G
2QTXA	19	- 23	23	L-->K
2QTXA	19	- 23	23	L-->W
2PNRB	294	- 299	295	R-->I

2PNRB	294 - 299	296	K-->L	
2PNRB	294 - 299	297	I-->G	
2PNRB	374 - 378	374	K-->G	
2PNRB	374 - 378	374	K-->S	
2PNRB	374 - 378	375	S-->L	
2PNRB	374 - 378	376	A-->P	
2PNRB	374 - 378	377	W-->D	
2PNRB	374 - 378	377	W-->I	
2PNRB	374 - 378	377	W-->K	
2PNRB	374 - 378	377	W-->L	
2PNRB	374 - 378	377	W-->S	
2PNRB	374 - 378	377	W-->V	
2PNRB	374 - 378	377	W-->Y	
2PNRB	374 - 378	378	R-->G	
2NUUD	63 - 68	63	G-->K	
2NUUD	63 - 68	67	A-->T	
2NUUD	63 - 68	68	F-->E	
2NUUD	301 - 306	303	K-->T	
2NUUD	301 - 306	304	R-->M	
2NUUD	314 - 318	314	V-->L	
2NUUD	314 - 318	314	V-->P	
2NUUD	314 - 318	314	V-->T	
2NUUD	314 - 318	315	F-->D	
2NUUD	314 - 318	315	F-->E	
2NUUD	314 - 318	315	F-->G	
2NUUD	314 - 318	315	F-->I	
2NUUD	314 - 318	315	F-->L	

2NUUD	314 - 318	315	F-->N	
2NUUD	314 - 318	315	F-->Q	
2NUUD	314 - 318	315	F-->S	
2NUUD	314 - 318	315	F-->T	
2NUUD	314 - 318	315	F-->V	
2NUUD	314 - 318	315	F-->Y	
2NUUD	314 - 318	316	G-->E	
2NUUD	314 - 318	316	G-->R	
2NUUD	314 - 318	316	G-->V	
2NUUD	314 - 318	317	V-->D	
2NUUD	314 - 318	317	V-->L	
2NUUD	314 - 318	317	V-->Q	
2NUUD	314 - 318	317	V-->Y	
2NUUD	314 - 318	318	H-->A	
2NUUD	314 - 318	318	H-->C	
2NUUD	314 - 318	318	H-->D	
2NUUD	314 - 318	318	H-->E	
2NUUD	314 - 318	318	H-->G	
2NUUD	314 - 318	318	H-->L	
2NUUD	314 - 318	318	H-->M	
2NUUD	314 - 318	318	H-->N	
2NUUD	314 - 318	318	H-->P	
2NUUD	314 - 318	318	H-->T	
2NUUD	314 - 318	318	H-->V	
2NUUD	314 - 318	318	H-->Y	
2NUUD	394 - 399	397	N-->G	
2NUUD	394 - 399	399	H-->V	

2A96B	142 - 148	143	E-->N
4MDHA	208 - 212	208	V-->E
4MDHA	208 - 212	208	V-->H
4MDHA	208 - 212	208	V-->P
4MDHA	208 - 212	208	V-->Q
4MDHA	208 - 212	208	V-->R
4MDHA	208 - 212	208	V-->T
4MDHA	208 - 212	208	V-->Y
4MDHA	208 - 212	209	Y-->A
4MDHA	208 - 212	209	Y-->C
4MDHA	208 - 212	209	Y-->E
4MDHA	208 - 212	209	Y-->F
4MDHA	208 - 212	209	Y-->G
4MDHA	208 - 212	209	Y-->I
4MDHA	208 - 212	209	Y-->L
4MDHA	208 - 212	209	Y-->N
4MDHA	208 - 212	209	Y-->T
4MDHA	208 - 212	209	Y-->V
4MDHA	208 - 212	210	E-->G
4MDHA	208 - 212	210	E-->R
4MDHA	208 - 212	210	E-->V
4MDHA	208 - 212	210	E-->Y
4MDHA	208 - 212	211	A-->C
4MDHA	208 - 212	211	A-->D
4MDHA	208 - 212	211	A-->G
4MDHA	208 - 212	211	A-->I
4MDHA	208 - 212	211	A-->K

4MDHA	208 - 212	211	A-->L
4MDHA	208 - 212	211	A-->N
4MDHA	208 - 212	211	A-->R
4MDHA	208 - 212	211	A-->T
4MDHA	208 - 212	211	A-->V
4MDHA	208 - 212	212	V-->C
4MDHA	208 - 212	212	V-->D
4MDHA	208 - 212	212	V-->E
4MDHA	208 - 212	212	V-->L
4MDHA	208 - 212	212	V-->N
4MDHA	208 - 212	212	V-->P
4MDHA	215 - 219	217	W-->A
4MDHA	215 - 219	217	W-->E
4MDHA	215 - 219	217	W-->F
4MDHA	215 - 219	217	W-->G
4MDHA	215 - 219	217	W-->K
4MDHA	215 - 219	217	W-->L
4MDHA	215 - 219	217	W-->P
4MDHA	215 - 219	217	W-->S
4MDHA	215 - 219	218	L-->G
4MDHA	215 - 219	218	L-->N
4MDHA	215 - 219	218	L-->V
4MDHA	215 - 219	218	L-->W
4MDHA	215 - 219	219	K-->D
1JNZB	720 - 724	720	A-->G
1JNZB	720 - 724	721	C-->D
1JNZB	720 - 724	721	C-->G

1JNZB	720 - 724	721	C-->T
1JNZB	720 - 724	722	E-->G
1JNZB	720 - 724	723	Y-->V
1JNZB	752 - 756	752	S-->P
1JNZB	752 - 756	753	C-->K
1JNZB	752 - 756	753	C-->S
1JNZB	752 - 756	753	C-->T
1JNZB	752 - 756	753	C-->V
1JNZB	752 - 756	755	K-->L
1JNZB	752 - 756	755	K-->R
1JNZB	752 - 756	756	M-->F
1JNZB	752 - 756	756	M-->K
1JNZB	752 - 756	756	M-->R
1JNZB	752 - 756	756	M-->S
2QEAA	52 - 56	52	D-->A
2QEAA	52 - 56	52	D-->F
2QEAA	52 - 56	52	D-->G
2QEAA	52 - 56	52	D-->I
2QEAA	52 - 56	52	D-->K
2QEAA	52 - 56	52	D-->L
2QEAA	52 - 56	52	D-->M
2QEAA	52 - 56	52	D-->N
2QEAA	52 - 56	52	D-->P
2QEAA	52 - 56	52	D-->S
2QEAA	52 - 56	52	D-->T
2QEAA	52 - 56	52	D-->W
2QEAA	52 - 56	53	L-->C

2QEAA	52 - 56	53	L-->I
2QEAA	52 - 56	53	L-->K
2QEAA	52 - 56	53	L-->N
2QEAA	52 - 56	53	L-->P
2QEAA	52 - 56	53	L-->S
2QEAA	52 - 56	53	L-->T
2QEAA	52 - 56	53	L-->V
2QEAA	52 - 56	53	L-->W
2QEAA	52 - 56	54	A-->E
2QEAA	52 - 56	54	A-->F
2QEAA	52 - 56	54	A-->G
2QEAA	52 - 56	54	A-->H
2QEAA	52 - 56	54	A-->K
2QEAA	52 - 56	54	A-->L
2QEAA	52 - 56	54	A-->P
2QEAA	52 - 56	54	A-->R
2QEAA	52 - 56	54	A-->S
2QEAA	52 - 56	54	A-->T
2QEAA	52 - 56	54	A-->Y
2QEAA	52 - 56	55	K-->C
2QEAA	52 - 56	55	K-->D
2QEAA	52 - 56	55	K-->E
2QEAA	52 - 56	55	K-->G
2QEAA	52 - 56	55	K-->L
2QEAA	52 - 56	55	K-->N
2QEAA	52 - 56	55	K-->P
2QEAA	52 - 56	55	K-->R

2QEAA	52	56	55	K-->S
2QEAA	52	56	55	K-->T
2QEAA	52	56	55	K-->V
2QEAA	52	56	55	K-->Y
2QEAA	52	56	56	G-->A
2QEAA	52	56	56	G-->D
2QEAA	52	56	56	G-->K
2QEAA	52	56	56	G-->L
2QEAA	52	56	56	G-->P
2QEAA	52	56	56	G-->V
2QEAA	89	94	92	L-->F
2QEAA	89	94	93	D-->A
2QEAA	98	103	99	V-->S
2QEAA	98	103	100	A-->Y
2GM3A	71	76	75	R-->P
2GM3A	71	76	76	D-->Y
1LPBA	75	80	75	L-->E
1LPBA	75	80	76	V-->A
1LPBA	75	80	76	V-->F
1LPBA	75	80	77	G-->F
1LPBA	75	80	77	G-->H
1LPBA	75	80	77	G-->P
1LPBA	75	80	77	G-->Y
1LPBA	75	80	78	S-->I
1LPBA	75	80	78	S-->K
1LPBA	75	80	78	S-->P
1LPBA	75	80	78	S-->R

1LPBA	75	80	79	I-->E
1LPBA	75	80	79	I-->T
1CY0A	67	71	68	V-->L
1CY0A	67	71	68	V-->P
1CY0A	67	71	69	N-->A
1CY0A	67	71	69	N-->G
1CY0A	67	71	69	N-->R
1CY0A	67	71	69	N-->V
1CY0A	67	71	70	R-->I
1CY0A	67	71	71	M-->A
1CY0A	67	71	71	M-->D
1CY0A	67	71	71	M-->E
1CY0A	67	71	71	M-->G
1CY0A	67	71	71	M-->H
1CY0A	67	71	71	M-->I
1CY0A	67	71	71	M-->K
1CY0A	67	71	71	M-->R
1CY0A	433	437	433	G-->H
1CY0A	433	437	433	G-->N
1CY0A	433	437	433	G-->P
1CY0A	433	437	434	W-->A
1CY0A	433	437	434	W-->D
1CY0A	433	437	434	W-->E
1CY0A	433	437	434	W-->F
1CY0A	433	437	434	W-->G
1CY0A	433	437	434	W-->H
1CY0A	433	437	434	W-->I

1CY0A	433 - 437	434	W-->K
1CY0A	433 - 437	434	W-->L
1CY0A	433 - 437	434	W-->N
1CY0A	433 - 437	434	W-->P
1CY0A	433 - 437	434	W-->R
1CY0A	433 - 437	434	W-->T
1CY0A	433 - 437	434	W-->V
1CY0A	433 - 437	435	T-->A
1CY0A	433 - 437	435	T-->F
1CY0A	433 - 437	435	T-->H
1CY0A	433 - 437	435	T-->L
1CY0A	433 - 437	435	T-->R
1CY0A	433 - 437	435	T-->S
1CY0A	433 - 437	436	K-->A
1CY0A	433 - 437	436	K-->D
1CY0A	433 - 437	436	K-->G
1CY0A	433 - 437	436	K-->N
1CY0A	433 - 437	436	K-->V
1CY0A	433 - 437	437	V-->A
1CY0A	433 - 437	437	V-->D
1CY0A	433 - 437	437	V-->E
1CY0A	433 - 437	437	V-->F
1CY0A	433 - 437	437	V-->G
1CY0A	433 - 437	437	V-->I
1CY0A	433 - 437	437	V-->P
1UVXA	2 - 6	2	L-->D
1UVXA	2 - 6	2	L-->F

1UVXA	2 - 6	2	L-->S
1UVXA	2 - 6	2	L-->V
1UVXA	2 - 6	3	F-->A
1UVXA	2 - 6	3	F-->D
1UVXA	2 - 6	3	F-->E
1UVXA	2 - 6	3	F-->G
1UVXA	2 - 6	3	F-->H
1UVXA	2 - 6	3	F-->I
1UVXA	2 - 6	3	F-->K
1UVXA	2 - 6	3	F-->L
1UVXA	2 - 6	3	F-->N
1UVXA	2 - 6	3	F-->P
1UVXA	2 - 6	3	F-->T
1UVXA	2 - 6	3	F-->V
1UVXA	2 - 6	4	A-->D
1UVXA	2 - 6	4	A-->H
1UVXA	2 - 6	4	A-->N
1UVXA	2 - 6	4	A-->Q
1UVXA	2 - 6	4	A-->V
1UVXA	2 - 6	5	K-->D
1UVXA	2 - 6	5	K-->F
1UVXA	2 - 6	5	K-->G
1UVXA	2 - 6	5	K-->L
1UVXA	2 - 6	5	K-->N
1UVXA	2 - 6	5	K-->P
1UVXA	2 - 6	5	K-->Q
1UVXA	2 - 6	5	K-->R

1UVXA	2 -	6	6	L-->D
1UVXA	2 -	6	6	L-->G
1UVXA	2 -	6	6	L-->H
1UVXA	64 -	68	64	M-->G
1UVXA	64 -	68	64	M-->P
1UVXA	64 -	68	65	R-->V
1UVXA	64 -	68	66	T-->F
1UVXA	64 -	68	66	T-->G
1UVXA	64 -	68	67	A-->F
1UVXA	64 -	68	68	H-->C
1UVXA	64 -	68	68	H-->G
1UVXA	64 -	68	68	H-->L
1EODA	81 -	85	81	L-->A
1EODA	81 -	85	81	L-->D
1EODA	81 -	85	81	L-->H
1EODA	81 -	85	81	L-->N
1EODA	81 -	85	81	L-->R
1EODA	81 -	85	81	L-->T
1EODA	81 -	85	81	L-->V
1EODA	81 -	85	82	K-->D
1EODA	81 -	85	82	K-->I
1EODA	81 -	85	82	K-->L
1EODA	81 -	85	82	K-->N
1EODA	81 -	85	82	K-->Q
1EODA	81 -	85	82	K-->S
1EODA	81 -	85	82	K-->T
1EODA	81 -	85	82	K-->V

1EODA	81 - 85	82	K-->Y	
1EODA	81 - 85	83	T-->A	
1EODA	81 - 85	83	T-->D	
1EODA	81 - 85	83	T-->E	
1EODA	81 - 85	83	T-->G	
1EODA	81 - 85	83	T-->H	
1EODA	81 - 85	83	T-->I	
1EODA	81 - 85	83	T-->K	
1EODA	81 - 85	83	T-->L	
1EODA	81 - 85	83	T-->M	
1EODA	81 - 85	83	T-->N	
1EODA	81 - 85	83	T-->P	
1EODA	81 - 85	83	T-->S	
1EODA	81 - 85	83	T-->V	
1EODA	81 - 85	83	T-->W	
1EODA	81 - 85	84	L-->A	
1EODA	81 - 85	84	L-->E	
1EODA	81 - 85	84	L-->G	
1EODA	81 - 85	84	L-->N	
1EODA	81 - 85	84	L-->P	
1EODA	81 - 85	84	L-->S	
1EODA	81 - 85	84	L-->Y	
1EODA	81 - 85	85	N-->A	
1EODA	81 - 85	85	N-->D	
1EODA	81 - 85	85	N-->E	
1EODA	81 - 85	85	N-->G	
1EODA	81 - 85	85	N-->H	

1EODA	81 - 85	85	N-->L
1EODA	81 - 85	85	N-->P
1EODA	81 - 85	85	N-->Q
1EODA	81 - 85	85	N-->R
1EODA	81 - 85	85	N-->S
1EODA	81 - 85	85	N-->T
1EODA	81 - 85	85	N-->V
1EODA	81 - 85	85	N-->W
1EODA	81 - 85	85	N-->Y
2DG2A	216 - 220	216	K-->H
2DG2A	216 - 220	216	K-->I
2DG2A	216 - 220	216	K-->M
2DG2A	216 - 220	216	K-->T
2DG2A	216 - 220	217	S-->A
2DG2A	216 - 220	217	S-->V
2DG2A	216 - 220	218	A-->K
2DG2A	216 - 220	218	A-->W
2DG2A	216 - 220	219	T-->K
2DG2A	216 - 220	219	T-->L
2DG2A	216 - 220	219	T-->N
2DG2A	216 - 220	220	H-->F
2DG2A	216 - 220	220	H-->G
2DG2A	216 - 220	220	H-->L
2DG2A	216 - 220	220	H-->V
2DG2A	235 - 240	235	P-->L
2DG2A	235 - 240	236	A-->E
2DG2A	235 - 240	237	L-->S

2DG2A	235	- 240	238	E-->A
2DG2A	235	- 240	240	K-->A
2DG2A	235	- 240	240	K-->G
1G5XA	37	- 42	37	Q-->G
1G5XA	133	- 137	133	V-->G
1G5XA	133	- 137	133	V-->I
1G5XA	133	- 137	133	V-->L
1G5XA	133	- 137	133	V-->N
1G5XA	133	- 137	133	V-->T
1G5XA	133	- 137	134	V-->D
1G5XA	133	- 137	134	V-->G
1G5XA	133	- 137	134	V-->K
1G5XA	133	- 137	134	V-->R
1G5XA	133	- 137	135	T-->A
1G5XA	133	- 137	135	T-->D
1G5XA	133	- 137	135	T-->F
1G5XA	133	- 137	135	T-->G
1G5XA	133	- 137	135	T-->H
1G5XA	133	- 137	135	T-->I
1G5XA	133	- 137	135	T-->K
1G5XA	133	- 137	135	T-->L
1G5XA	133	- 137	135	T-->N
1G5XA	133	- 137	135	T-->P
1G5XA	133	- 137	135	T-->R
1G5XA	133	- 137	135	T-->V
1G5XA	133	- 137	135	T-->Y
1G5XA	133	- 137	136	K-->A

1G5XA	133	137	136	K-->D
1G5XA	133	137	136	K-->E
1G5XA	133	137	136	K-->F
1G5XA	133	137	136	K-->G
1G5XA	133	137	136	K-->H
1G5XA	133	137	136	K-->I
1G5XA	133	137	136	K-->L
1G5XA	133	137	136	K-->N
1G5XA	133	137	136	K-->P
1G5XA	133	137	136	K-->Q
1G5XA	133	137	136	K-->R
1G5XA	133	137	136	K-->T
1G5XA	133	137	136	K-->V
1G5XA	133	137	137	A-->D
1G5XA	133	137	137	A-->E
1G5XA	133	137	137	A-->F
1G5XA	133	137	137	A-->G
1G5XA	133	137	137	A-->I
1G5XA	133	137	137	A-->K
1G5XA	133	137	137	A-->L
1G5XA	133	137	137	A-->N
1G5XA	133	137	137	A-->P
1G5XA	133	137	137	A-->R
1G5XA	133	137	137	A-->S
1G5XA	133	137	137	A-->T
1G5XA	243	248	243	L-->S
1G5XA	243	248	244	E-->A

1G5XA	243	248	244	E-->G
1PP7U	6	11	6	L-->K
1PP7U	6	11	6	L-->T
1PP7U	6	11	8	A-->I
1PP7U	6	11	9	S-->A
1PP7U	6	11	9	S-->F
1PP7U	6	11	11	T-->K
1WVUA	239	244	241	N-->P
2CX6A	60	65	62	T-->R
1GZ5A	84	88	85	W-->A
1GZ5A	84	88	85	W-->E
1GZ5A	84	88	85	W-->G
1GZ5A	84	88	85	W-->H
1GZ5A	84	88	85	W-->K
1GZ5A	84	88	85	W-->M
1GZ5A	84	88	85	W-->N
1GZ5A	84	88	85	W-->P
1GZ5A	84	88	85	W-->R
1GZ5A	84	88	85	W-->S
1GZ5A	84	88	87	A-->D
1GZ5A	84	88	87	A-->K
1GZ5A	84	88	87	A-->R
1GZ5A	346	350	346	K-->A
1GZ5A	346	350	346	K-->D
1GZ5A	346	350	346	K-->L
1GZ5A	346	350	347	I-->G
1GZ5A	346	350	348	F-->E

1GZ5A	346 - 350	348	F-->V
1GZ5A	346 - 350	348	F-->Y
1GZ5A	346 - 350	349	R-->A
1GZ5A	346 - 350	349	R-->D
1GZ5A	346 - 350	349	R-->G
1GZ5A	346 - 350	349	R-->H
1GZ5A	346 - 350	349	R-->I
1GZ5A	346 - 350	349	R-->L
1GZ5A	346 - 350	349	R-->Q
1GZ5A	346 - 350	349	R-->S
1GZ5A	346 - 350	349	R-->T
1GZ5A	346 - 350	350	Y-->G
1GZ5A	346 - 350	350	Y-->K
1GZ5A	346 - 350	350	Y-->V
1GZ5A	431 - 436	431	L-->A
1GZ5A	431 - 436	431	L-->F
1GZ5A	431 - 436	431	L-->H
1GZ5A	431 - 436	432	D-->P
1GZ5A	431 - 436	434	I-->Q
3B77E	28 - 32	28	A-->D
3B77E	28 - 32	28	A-->E
3B77E	28 - 32	28	A-->G
3B77E	28 - 32	28	A-->K
3B77E	28 - 32	28	A-->L
3B77E	28 - 32	28	A-->N
3B77E	28 - 32	28	A-->P
3B77E	28 - 32	28	A-->S

3B77E	28 - 32	28	A-->T
3B77E	28 - 32	28	A-->V
3B77E	28 - 32	28	A-->Y
3B77E	28 - 32	29	A-->D
3B77E	28 - 32	29	A-->G
3B77E	28 - 32	29	A-->Q
3B77E	28 - 32	29	A-->T
3B77E	28 - 32	29	A-->V
3B77E	28 - 32	30	D-->E
3B77E	28 - 32	30	D-->G
3B77E	28 - 32	30	D-->H
3B77E	28 - 32	30	D-->L
3B77E	28 - 32	30	D-->M
3B77E	28 - 32	30	D-->P
3B77E	28 - 32	31	D-->G
3B77E	28 - 32	31	D-->H
3B77E	28 - 32	31	D-->I
3B77E	28 - 32	31	D-->L
3B77E	28 - 32	31	D-->N
3B77E	28 - 32	31	D-->P
3B77E	28 - 32	31	D-->T
3B77E	28 - 32	31	D-->V
3B77E	28 - 32	32	Y-->A
3B77E	28 - 32	32	Y-->C
3B77E	28 - 32	32	Y-->D
3B77E	28 - 32	32	Y-->E
3B77E	28 - 32	32	Y-->F

3B77E	28 - 32	32	Y-->G
3B77E	28 - 32	32	Y-->L
3B77E	28 - 32	32	Y-->P
3B77E	28 - 32	32	Y-->R
3B77E	28 - 32	32	Y-->S
3B77E	28 - 32	32	Y-->T
3B77E	28 - 32	32	Y-->V
3B77E	28 - 32	32	Y-->W
2Q1ZC	140 - 146	146	F-->V
1CJMA	22 - 27	24	F-->D
1CJMA	116 - 120	116	Q-->C
1CJMA	116 - 120	116	Q-->F
1CJMA	116 - 120	116	Q-->G
1CJMA	116 - 120	116	Q-->I
1CJMA	116 - 120	116	Q-->L
1CJMA	116 - 120	116	Q-->M
1CJMA	116 - 120	116	Q-->N
1CJMA	116 - 120	116	Q-->T
1CJMA	116 - 120	116	Q-->V
1CJMA	116 - 120	117	T-->D
1CJMA	116 - 120	117	T-->L
1CJMA	116 - 120	117	T-->N
1CJMA	116 - 120	117	T-->R
1CJMA	116 - 120	117	T-->S
1CJMA	116 - 120	117	T-->Y
1CJMA	116 - 120	118	L-->C
1CJMA	116 - 120	118	L-->G

1CJMA	116	120	118	L-->H
1CJMA	116	120	118	L-->N
1CJMA	116	120	118	L-->P
1CJMA	116	120	118	L-->R
1CJMA	116	120	118	L-->S
1CJMA	116	120	119	L-->K
1CJMA	116	120	119	L-->N
1CJMA	116	120	119	L-->S
1CJMA	116	120	120	D-->E
1CJMA	116	120	120	D-->G
1CJMA	116	120	120	D-->K
1CJMA	116	120	120	D-->L
1CJMA	116	120	120	D-->N
1CJMA	116	120	120	D-->P
1CJMA	116	120	120	D-->Q
1CJMA	116	120	120	D-->T
1CJMA	193	198	194	E-->R
1CJMA	193	198	198	E-->G
2F44C	33	38	33	G-->A
2F44C	33	38	35	H-->A
2F44C	33	38	35	H-->F
2F44C	33	38	35	H-->V
2F44C	33	38	37	P-->N
2F44C	33	38	37	P-->Y
2F44C	41	46	42	E-->V
2F44C	41	46	45	V-->D
2F44C	41	46	46	E-->G

2BHMA	151 - 155	151	L-->G	
2BHMA	151 - 155	152	D-->E	
2BHMA	151 - 155	152	D-->G	
2BHMA	151 - 155	152	D-->N	
2BHMA	151 - 155	152	D-->R	
2BHMA	151 - 155	152	D-->Y	
2BHMA	151 - 155	153	K-->D	
2BHMA	151 - 155	153	K-->I	
2BHMA	151 - 155	153	K-->L	
2BHMA	151 - 155	153	K-->R	
2BHMA	151 - 155	153	K-->S	
2BHMA	151 - 155	153	K-->V	
2BHMA	151 - 155	153	K-->W	
2BHMA	151 - 155	154	Q-->A	
2BHMA	151 - 155	154	Q-->D	
2BHMA	151 - 155	154	Q-->E	
2BHMA	151 - 155	154	Q-->I	
2BHMA	151 - 155	154	Q-->L	
2BHMA	151 - 155	154	Q-->P	
2BHMA	151 - 155	154	Q-->V	
2BHMA	151 - 155	155	Y-->G	
2BHMA	151 - 155	155	Y-->K	
2BHMA	151 - 155	155	Y-->Q	
2BHMA	151 - 155	155	Y-->R	
2BHMA	151 - 155	155	Y-->T	
2BHMA	151 - 155	155	Y-->V	
1T94A	141 - 145	141	Y-->G	

1T94A	141 - 145	141	Y-->I
1T94A	141 - 145	142	H-->C
1T94A	141 - 145	142	H-->R
1T94A	141 - 145	145	R-->A
1T94A	205 - 211	207	H-->I
1T94A	236 - 241	238	V-->E
1T94A	236 - 241	238	V-->F
1T94A	236 - 241	238	V-->K
1T94A	236 - 241	241	L-->G
1T94A	373 - 378	378	Q-->H
2Z8OA	141 - 145	141	E-->F
2Z8OA	141 - 145	141	E-->K
2Z8OA	141 - 145	141	E-->N
2Z8OA	141 - 145	142	K-->D
2Z8OA	141 - 145	142	K-->E
2Z8OA	141 - 145	142	K-->I
2Z8OA	141 - 145	142	K-->L
2Z8OA	141 - 145	142	K-->N
2Z8OA	141 - 145	142	K-->V
2Z8OA	141 - 145	143	V-->A
2Z8OA	141 - 145	143	V-->E
2Z8OA	141 - 145	143	V-->K
2Z8OA	141 - 145	143	V-->L
2Z8OA	141 - 145	143	V-->N
2Z8OA	141 - 145	143	V-->P
2Z8OA	141 - 145	144	V-->D
2Z8OA	141 - 145	144	V-->F

2Z8OA	141 - 145	144	V-->G
2Z8OA	141 - 145	144	V-->I
2Z8OA	141 - 145	145	Q-->A
2Z8OA	141 - 145	145	Q-->D
2Z8OA	141 - 145	145	Q-->E
2Z8OA	141 - 145	145	Q-->F
2Z8OA	141 - 145	145	Q-->G
2Z8OA	141 - 145	145	Q-->I
2Z8OA	141 - 145	145	Q-->L
2Z8OA	141 - 145	145	Q-->N
2Z8OA	141 - 145	145	Q-->P
2Z8OA	141 - 145	145	Q-->S
2Z8OA	141 - 145	145	Q-->V
2Z8OA	141 - 145	145	Q-->W
2Z8OA	141 - 145	145	Q-->Y
2Z8OA	226 - 231	228	Q-->I
2Z8OA	234 - 238	234	P-->K
2Z8OA	234 - 238	234	P-->S
2Z8OA	234 - 238	235	F-->E
2Z8OA	234 - 238	235	F-->G
2Z8OA	234 - 238	235	F-->N
2Z8OA	234 - 238	235	F-->S
2Z8OA	234 - 238	235	F-->Y
2Z8OA	234 - 238	236	Y-->A
2Z8OA	234 - 238	236	Y-->D
2Z8OA	234 - 238	236	Y-->G
2Z8OA	234 - 238	236	Y-->L

2Z8OA	234 - 238	236	Y-->N
2Z8OA	234 - 238	236	Y-->P
2Z8OA	234 - 238	236	Y-->S
2Z8OA	234 - 238	236	Y-->V
2Z8OA	234 - 238	237	R-->A
2Z8OA	234 - 238	237	R-->D
2Z8OA	234 - 238	237	R-->G
2Z8OA	234 - 238	237	R-->P
2Z8OA	234 - 238	237	R-->Q
2Z8OA	234 - 238	237	R-->V
2Z8OA	234 - 238	238	L-->A
2Z8OA	234 - 238	238	L-->G
2Z8OA	234 - 238	238	L-->Q
2Z8OA	234 - 238	238	L-->T
2Z8OA	234 - 238	238	L-->Y
2PCSA	116 - 120	116	K-->A
2PCSA	116 - 120	116	K-->E
2PCSA	116 - 120	116	K-->F
2PCSA	116 - 120	116	K-->G
2PCSA	116 - 120	116	K-->H
2PCSA	116 - 120	116	K-->I
2PCSA	116 - 120	116	K-->L
2PCSA	116 - 120	116	K-->P
2PCSA	116 - 120	116	K-->Q
2PCSA	116 - 120	116	K-->R
2PCSA	116 - 120	116	K-->S
2PCSA	116 - 120	116	K-->T

2PCSA	116 - 120	116	K-->V
2PCSA	116 - 120	116	K-->W
2PCSA	116 - 120	117	V-->A
2PCSA	116 - 120	117	V-->D
2PCSA	116 - 120	117	V-->E
2PCSA	116 - 120	117	V-->F
2PCSA	116 - 120	117	V-->I
2PCSA	116 - 120	117	V-->P
2PCSA	116 - 120	117	V-->S
2PCSA	116 - 120	117	V-->T
2PCSA	116 - 120	118	A-->D
2PCSA	116 - 120	118	A-->E
2PCSA	116 - 120	118	A-->H
2PCSA	116 - 120	118	A-->I
2PCSA	116 - 120	118	A-->K
2PCSA	116 - 120	118	A-->N
2PCSA	116 - 120	118	A-->P
2PCSA	116 - 120	118	A-->Q
2PCSA	116 - 120	118	A-->T
2PCSA	116 - 120	118	A-->V
2PCSA	116 - 120	119	A-->D
2PCSA	116 - 120	119	A-->E
2PCSA	116 - 120	119	A-->F
2PCSA	116 - 120	119	A-->G
2PCSA	116 - 120	119	A-->I
2PCSA	116 - 120	119	A-->K
2PCSA	116 - 120	119	A-->L

2PCSA	116 - 120	119	A-->M
2PCSA	116 - 120	119	A-->N
2PCSA	116 - 120	119	A-->P
2PCSA	116 - 120	119	A-->S
2PCSA	116 - 120	119	A-->T
2PCSA	116 - 120	119	A-->V
2PCSA	116 - 120	119	A-->Y
2PCSA	116 - 120	120	I-->A
2PCSA	116 - 120	120	I-->C
2PCSA	116 - 120	120	I-->F
2PCSA	116 - 120	120	I-->G
2PCSA	116 - 120	120	I-->H
2PCSA	116 - 120	120	I-->K
2PCSA	116 - 120	120	I-->N
2PCSA	116 - 120	120	I-->P
2PCSA	116 - 120	120	I-->R
2PCSA	116 - 120	120	I-->S
2PCSA	116 - 120	120	I-->T
2PCSA	116 - 120	120	I-->V
2Z7JA	118 - 123	118	P-->L
2Z7JA	118 - 123	120	W-->I
2Z7JA	118 - 123	120	W-->P
2Z7JA	118 - 123	122	A-->I
2FBVA	122 - 128	126	M-->I
2O5NA	122 - 126	122	E-->A
2O5NA	122 - 126	122	E-->C
2O5NA	122 - 126	122	E-->D

2O5NA	122 - 126	122	E-->F
2O5NA	122 - 126	122	E-->G
2O5NA	122 - 126	122	E-->I
2O5NA	122 - 126	122	E-->K
2O5NA	122 - 126	122	E-->N
2O5NA	122 - 126	122	E-->P
2O5NA	122 - 126	122	E-->Q
2O5NA	122 - 126	122	E-->R
2O5NA	122 - 126	122	E-->T
2O5NA	122 - 126	122	E-->V
2O5NA	122 - 126	122	E-->Y
2O5NA	122 - 126	123	D-->A
2O5NA	122 - 126	123	D-->E
2O5NA	122 - 126	123	D-->F
2O5NA	122 - 126	123	D-->G
2O5NA	122 - 126	123	D-->H
2O5NA	122 - 126	123	D-->I
2O5NA	122 - 126	123	D-->K
2O5NA	122 - 126	123	D-->L
2O5NA	122 - 126	123	D-->Q
2O5NA	122 - 126	123	D-->R
2O5NA	122 - 126	123	D-->T
2O5NA	122 - 126	123	D-->V
2O5NA	122 - 126	124	T-->A
2O5NA	122 - 126	124	T-->D
2O5NA	122 - 126	124	T-->E
2O5NA	122 - 126	124	T-->F

2O5NA	122 - 126	124	T-->G
2O5NA	122 - 126	124	T-->I
2O5NA	122 - 126	124	T-->K
2O5NA	122 - 126	124	T-->L
2O5NA	122 - 126	124	T-->P
2O5NA	122 - 126	124	T-->V
2O5NA	122 - 126	125	L-->A
2O5NA	122 - 126	125	L-->E
2O5NA	122 - 126	125	L-->I
2O5NA	122 - 126	125	L-->M
2O5NA	122 - 126	125	L-->P
2O5NA	122 - 126	125	L-->R
2O5NA	122 - 126	125	L-->V
2O5NA	122 - 126	126	V-->D
2O5NA	122 - 126	126	V-->F
2O5NA	122 - 126	126	V-->K
2O5NA	122 - 126	126	V-->P
2O5NA	122 - 126	126	V-->R
2O5NA	122 - 126	126	V-->S
2O5NA	122 - 126	126	V-->Y
2O5NA	122 - 126	122	T-->A
2O5NA	122 - 126	122	T-->D
2O5NA	122 - 126	122	T-->G
2O5NA	122 - 126	122	T-->I
2O5NA	122 - 126	122	T-->K
2O5NA	122 - 126	122	T-->L
2O5NA	122 - 126	122	T-->P

2O5NA	122	126	122	T-->Q
2O5NA	122	126	122	T-->R
2O5NA	122	126	122	T-->S
2O5NA	122	126	122	T-->V
2O5NA	122	126	122	T-->Y
2O5NA	122	126	123	L-->H
2O5NA	122	126	123	L-->N
2O5NA	122	126	123	L-->T
2O5NA	122	126	123	L-->V
2O5NA	122	126	124	L-->D
2O5NA	122	126	124	L-->E
2O5NA	122	126	124	L-->F
2O5NA	122	126	124	L-->G
2O5NA	122	126	124	L-->I
2O5NA	122	126	124	L-->N
2O5NA	122	126	124	L-->T
2O5NA	122	126	124	L-->Y
2O5NA	122	126	125	T-->A
2O5NA	122	126	125	T-->C
2O5NA	122	126	125	T-->D
2O5NA	122	126	125	T-->E
2O5NA	122	126	125	T-->F
2O5NA	122	126	125	T-->G
2O5NA	122	126	125	T-->K
2O5NA	122	126	125	T-->L
2O5NA	122	126	125	T-->M
2O5NA	122	126	125	T-->N

2O5NA	122 - 126	125	T-->P
2O5NA	122 - 126	125	T-->S
2O5NA	122 - 126	125	T-->V
2O5NA	122 - 126	125	T-->Y
2O5NA	122 - 126	126	D-->E
2O5NA	122 - 126	126	D-->F
2O5NA	122 - 126	126	D-->G
2O5NA	122 - 126	126	D-->L
2O5NA	122 - 126	126	D-->N
2O5NA	122 - 126	126	D-->P
2O5NA	122 - 126	126	D-->R
2O5NA	122 - 126	126	D-->V
2PH7A	37 - 42	37	G-->P
2PH7A	37 - 42	38	A-->F
2PH7A	37 - 42	38	A-->L
2PH7A	37 - 42	38	A-->S
2PH7A	37 - 42	38	A-->V
2PH7A	37 - 42	39	R-->D
2PH7A	37 - 42	39	R-->E
2PH7A	37 - 42	39	R-->F
2PH7A	37 - 42	39	R-->L
2PH7A	37 - 42	39	R-->T
2PH7A	37 - 42	40	L-->A
2PH7A	37 - 42	40	L-->G
2PH7A	37 - 42	40	L-->R
2PH7A	37 - 42	40	L-->S
2PH7A	37 - 42	40	L-->Y

2PH7A	37 - 42	41	V-->T
2PH7A	37 - 42	42	G-->A
2PH7A	37 - 42	42	G-->V
2PH7A	37 - 41	37	I-->A
2PH7A	37 - 41	37	I-->S
2PH7A	37 - 41	38	D-->L
2PH7A	37 - 41	39	D-->H
2PH7A	218 - 223	219	R-->V
2PH7A	218 - 223	220	L-->D
2PH7A	218 - 223	220	L-->P
2PH7A	218 - 223	222	K-->E
2PH7A	218 - 223	223	G-->R
1YCOB	218 - 222	218	L-->A
1YCOB	218 - 222	218	L-->F
1YCOB	218 - 222	218	L-->H
1YCOB	218 - 222	218	L-->I
1YCOB	218 - 222	218	L-->Q
1YCOB	218 - 222	218	L-->R
1YCOB	218 - 222	218	L-->S
1YCOB	218 - 222	218	L-->V
1YCOB	218 - 222	218	L-->Y
1YCOB	218 - 222	219	D-->A
1YCOB	218 - 222	219	D-->C
1YCOB	218 - 222	219	D-->E
1YCOB	218 - 222	219	D-->F
1YCOB	218 - 222	219	D-->G
1YCOB	218 - 222	219	D-->H

1YCOB	218 - 222	219	D-->K
1YCOB	218 - 222	219	D-->L
1YCOB	218 - 222	219	D-->Q
1YCOB	218 - 222	219	D-->S
1YCOB	218 - 222	219	D-->T
1YCOB	218 - 222	219	D-->V
1YCOB	218 - 222	220	L-->A
1YCOB	218 - 222	220	L-->D
1YCOB	218 - 222	220	L-->E
1YCOB	218 - 222	220	L-->F
1YCOB	218 - 222	220	L-->G
1YCOB	218 - 222	220	L-->H
1YCOB	218 - 222	220	L-->I
1YCOB	218 - 222	220	L-->K
1YCOB	218 - 222	220	L-->P
1YCOB	218 - 222	220	L-->T
1YCOB	218 - 222	220	L-->V
1YCOB	218 - 222	221	A-->D
1YCOB	218 - 222	221	A-->G
1YCOB	218 - 222	221	A-->K
1YCOB	218 - 222	221	A-->N
1YCOB	218 - 222	221	A-->P
1YCOB	218 - 222	221	A-->Q
1YCOB	218 - 222	221	A-->R
1YCOB	218 - 222	221	A-->S
1YCOB	218 - 222	221	A-->V
1YCOB	218 - 222	222	T-->A

1YCOB	218 - 222	222	T-->C
1YCOB	218 - 222	222	T-->D
1YCOB	218 - 222	222	T-->E
1YCOB	218 - 222	222	T-->G
1YCOB	218 - 222	222	T-->I
1YCOB	218 - 222	222	T-->L
1YCOB	218 - 222	222	T-->N
1YCOB	218 - 222	222	T-->R
1YCOB	218 - 222	222	T-->S
1YCOB	218 - 222	222	T-->V
1YCOB	218 - 222	222	T-->W
1YCOB	218 - 222	222	T-->Y
1YCOB	197 - 202	198	E-->K
1YCOB	197 - 202	199	A-->I
1YCOB	197 - 202	202	H-->L
1YCOB	197 - 202	202	H-->S
1YCOB	197 - 202	202	H-->V
2OASA	9 - 13	9	A-->D
2OASA	9 - 13	9	A-->E
2OASA	9 - 13	9	A-->G
2OASA	9 - 13	9	A-->H
2OASA	9 - 13	9	A-->I
2OASA	9 - 13	9	A-->K
2OASA	9 - 13	9	A-->L
2OASA	9 - 13	9	A-->P
2OASA	9 - 13	9	A-->Q
2OASA	9 - 13	9	A-->R

2OASA	9 - 13	9	A-->S
2OASA	9 - 13	9	A-->V
2OASA	9 - 13	9	A-->W
2OASA	9 - 13	10	L-->C
2OASA	9 - 13	10	L-->D
2OASA	9 - 13	10	L-->E
2OASA	9 - 13	10	L-->G
2OASA	9 - 13	10	L-->I
2OASA	9 - 13	10	L-->M
2OASA	9 - 13	10	L-->N
2OASA	9 - 13	10	L-->P
2OASA	9 - 13	10	L-->Q
2OASA	9 - 13	10	L-->R
2OASA	9 - 13	10	L-->T
2OASA	9 - 13	10	L-->V
2OASA	9 - 13	11	E-->A
2OASA	9 - 13	11	E-->C
2OASA	9 - 13	11	E-->D
2OASA	9 - 13	11	E-->G
2OASA	9 - 13	11	E-->I
2OASA	9 - 13	11	E-->L
2OASA	9 - 13	11	E-->M
2OASA	9 - 13	11	E-->N
2OASA	9 - 13	11	E-->P
2OASA	9 - 13	11	E-->T
2OASA	9 - 13	11	E-->V
2OASA	9 - 13	12	A-->D

2OASA	9 - 13	12	A-->F
2OASA	9 - 13	12	A-->G
2OASA	9 - 13	12	A-->H
2OASA	9 - 13	12	A-->L
2OASA	9 - 13	12	A-->N
2OASA	9 - 13	12	A-->Q
2OASA	9 - 13	12	A-->R
2OASA	9 - 13	12	A-->T
2OASA	9 - 13	12	A-->V
2OASA	9 - 13	13	V-->A
2OASA	9 - 13	13	V-->D
2OASA	9 - 13	13	V-->G
2OASA	9 - 13	13	V-->K
2OASA	9 - 13	13	V-->M
2OASA	9 - 13	13	V-->S
2OASA	9 - 13	13	V-->T
2OASA	82 - 86	82	R-->D
2OASA	82 - 86	82	R-->G
2OASA	82 - 86	82	R-->H
2OASA	82 - 86	82	R-->I
2OASA	82 - 86	82	R-->K
2OASA	82 - 86	82	R-->L
2OASA	82 - 86	82	R-->N
2OASA	82 - 86	82	R-->S
2OASA	82 - 86	82	R-->V
2OASA	82 - 86	83	P-->F
2OASA	82 - 86	83	P-->L

2OASA	82 - 86	83	P-->M	
2OASA	82 - 86	83	P-->N	
2OASA	82 - 86	83	P-->R	
2OASA	82 - 86	83	P-->Y	
2OASA	82 - 86	84	L-->D	
2OASA	82 - 86	84	L-->I	
2OASA	82 - 86	84	L-->N	
2OASA	82 - 86	84	L-->R	
2OASA	82 - 86	85	L-->F	
2OASA	82 - 86	85	L-->P	
2OASA	82 - 86	85	L-->Q	
2OASA	82 - 86	85	L-->S	
2OASA	82 - 86	85	L-->V	
2OASA	82 - 86	86	Q-->A	
2OASA	82 - 86	86	Q-->D	
2OASA	82 - 86	86	Q-->G	
2OASA	82 - 86	86	Q-->I	
2OASA	82 - 86	86	Q-->L	
2OASA	82 - 86	86	Q-->N	
2OASA	82 - 86	86	Q-->P	
2OASA	82 - 86	86	Q-->S	
2OASA	82 - 86	86	Q-->V	
2OASA	136 - 142	137	L-->T	
3B8OA	290 - 295	290	L-->K	
3B8OA	290 - 295	291	N-->D	
3B8OA	290 - 295	292	T-->V	
3B8OA	290 - 295	293	L-->N	

3B8OA	290	295	293	L-->Q
3B8OA	290	295	293	L-->S
3B8OA	290	295	295	V-->N
3B8OA	290	295	295	V-->Y
2QV0A	144	149	147	A-->V
2QV0A	144	149	148	F-->D
1F6OA	95	101	99	A-->I
2P9JE	110	115	110	V-->E
2P9JE	110	115	110	V-->F
2P9JE	110	115	110	V-->I
2P9JE	110	115	110	V-->P
2P9JE	110	115	110	V-->Q
2P9JE	110	115	110	V-->S
2P9JE	110	115	111	V-->A
2P9JE	110	115	112	D-->T
2P9JE	110	115	113	I-->C
2P9JE	110	115	113	I-->G
2P9JE	110	115	114	E-->K
2P9JE	110	115	114	E-->W
2P9JE	110	115	115	V-->H
2CO1A	110	114	110	S-->A
2CO1A	110	114	110	S-->G
2CO1A	110	114	110	S-->I
2CO1A	110	114	110	S-->K
2CO1A	110	114	110	S-->L
2CO1A	110	114	110	S-->M
2CO1A	110	114	110	S-->P

2CO1A	110 - 114	110	S-->R
2CO1A	110 - 114	110	S-->W
2CO1A	110 - 114	111	D-->A
2CO1A	110 - 114	111	D-->E
2CO1A	110 - 114	111	D-->F
2CO1A	110 - 114	111	D-->G
2CO1A	110 - 114	111	D-->H
2CO1A	110 - 114	111	D-->I
2CO1A	110 - 114	111	D-->L
2CO1A	110 - 114	111	D-->M
2CO1A	110 - 114	111	D-->P
2CO1A	110 - 114	111	D-->Q
2CO1A	110 - 114	111	D-->T
2CO1A	110 - 114	111	D-->Y
2CO1A	110 - 114	112	S-->A
2CO1A	110 - 114	112	S-->E
2CO1A	110 - 114	112	S-->K
2CO1A	110 - 114	112	S-->L
2CO1A	110 - 114	112	S-->T
2CO1A	110 - 114	112	S-->V
2CO1A	110 - 114	113	L-->D
2CO1A	110 - 114	113	L-->G
2CO1A	110 - 114	113	L-->N
2CO1A	110 - 114	113	L-->R
2CO1A	110 - 114	113	L-->S
2CO1A	110 - 114	113	L-->V
2CO1A	110 - 114	113	L-->Y

2CO1A	110 - 114	114	R-->D
2CO1A	110 - 114	114	R-->E
2CO1A	110 - 114	114	R-->F
2CO1A	110 - 114	114	R-->G
2CO1A	110 - 114	114	R-->I
2CO1A	110 - 114	114	R-->K
2CO1A	110 - 114	114	R-->N
2CO1A	110 - 114	114	R-->P
2CO1A	110 - 114	114	R-->T
2CO1A	110 - 114	114	R-->Y
1HULA	110 - 114	110	R-->A
1HULA	110 - 114	110	R-->D
1HULA	110 - 114	110	R-->E
1HULA	110 - 114	110	R-->G
1HULA	110 - 114	110	R-->N
1HULA	110 - 114	110	R-->P
1HULA	110 - 114	110	R-->S
1HULA	110 - 114	110	R-->T
1HULA	110 - 114	111	T-->G
1HULA	110 - 114	111	T-->I
1HULA	110 - 114	111	T-->K
1HULA	110 - 114	111	T-->L
1HULA	110 - 114	111	T-->M
1HULA	110 - 114	111	T-->P
1HULA	110 - 114	112	L-->A
1HULA	110 - 114	112	L-->C
1HULA	110 - 114	112	L-->G

1HULA	110	114	112	L-->I
1HULA	110	114	112	L-->N
1HULA	110	114	112	L-->P
1HULA	110	114	112	L-->S
1HULA	110	114	112	L-->T
1HULA	110	114	112	L-->V
1HULA	110	114	113	L-->A
1HULA	110	114	113	L-->E
1HULA	110	114	113	L-->F
1HULA	110	114	113	L-->G
1HULA	110	114	113	L-->K
1HULA	110	114	113	L-->N
1HULA	110	114	113	L-->P
1HULA	110	114	113	L-->R
1HULA	110	114	113	L-->S
1HULA	110	114	113	L-->T
1HULA	110	114	113	L-->V
1HULA	110	114	113	L-->W
1HULA	110	114	114	I-->E
1HULA	110	114	114	I-->F
1HULA	110	114	114	I-->M
1HULA	110	114	114	I-->S
1HULA	110	114	114	I-->V
2FXAD	127	131	127	A-->E
2FXAD	127	131	127	A-->G
2FXAD	127	131	127	A-->I
2FXAD	127	131	127	A-->L

2FXAD	127 - 131	127	A-->M
2FXAD	127 - 131	127	A-->N
2FXAD	127 - 131	127	A-->R
2FXAD	127 - 131	127	A-->T
2FXAD	127 - 131	127	A-->W
2FXAD	127 - 131	127	A-->Y
2FXAD	127 - 131	128	V-->A
2FXAD	127 - 131	128	V-->D
2FXAD	127 - 131	128	V-->E
2FXAD	127 - 131	128	V-->G
2FXAD	127 - 131	128	V-->H
2FXAD	127 - 131	128	V-->K
2FXAD	127 - 131	128	V-->N
2FXAD	127 - 131	128	V-->Q
2FXAD	127 - 131	128	V-->R
2FXAD	127 - 131	128	V-->S
2FXAD	127 - 131	128	V-->T
2FXAD	127 - 131	129	F-->D
2FXAD	127 - 131	129	F-->G
2FXAD	127 - 131	129	F-->I
2FXAD	127 - 131	129	F-->K
2FXAD	127 - 131	129	F-->L
2FXAD	127 - 131	129	F-->M
2FXAD	127 - 131	129	F-->N
2FXAD	127 - 131	129	F-->R
2FXAD	127 - 131	129	F-->T
2FXAD	127 - 131	129	F-->V

2FXAD	127 - 131	129	F-->Y
2FXAD	127 - 131	130	K-->C
2FXAD	127 - 131	130	K-->D
2FXAD	127 - 131	130	K-->F
2FXAD	127 - 131	130	K-->G
2FXAD	127 - 131	130	K-->I
2FXAD	127 - 131	130	K-->L
2FXAD	127 - 131	130	K-->N
2FXAD	127 - 131	130	K-->S
2FXAD	127 - 131	130	K-->T
2FXAD	127 - 131	130	K-->V
2FXAD	127 - 131	131	G-->I
2FXAD	127 - 131	131	G-->K
2FXAD	127 - 131	131	G-->L
2FXAD	127 - 131	131	G-->P
2FXAD	127 - 131	131	G-->R
2FXAD	127 - 131	131	G-->S
2FXAD	127 - 131	131	G-->V
2FXAD	152 - 156	152	I-->S
2FXAD	152 - 156	153	R-->D
2FXAD	152 - 156	153	R-->I
2FXAD	152 - 156	153	R-->N
2FXAD	152 - 156	154	H-->D
2FXAD	152 - 156	154	H-->E
2FXAD	152 - 156	154	H-->F
2FXAD	152 - 156	154	H-->K
2FXAD	152 - 156	154	H-->R

2FXAD	152	156	154	H-->Y
2FXAD	152	156	155	I-->A
2FXAD	152	156	155	I-->D
2FXAD	152	156	155	I-->Q
2FXAD	152	156	155	I-->V
2FXAD	152	156	156	Y-->A
2FXAD	152	156	156	Y-->S
2FXAD	152	156	156	Y-->V
1ID1A	146	151	148	M-->A
1ID1A	146	151	148	M-->G
1ID1A	146	151	148	M-->S
2QYBA	94	99	95	L-->E
2QYBA	94	99	95	L-->N
2QYBA	94	99	96	T-->D
2QYBA	94	99	96	T-->S
2QYBA	94	99	97	R-->L
2QYBA	94	99	99	L-->G
2J3TB	73	78	74	W-->D
2J3TB	73	78	74	W-->L
2J3TB	73	78	74	W-->V
1F7CA	372	377	376	F-->V
2FDOA	64	69	64	E-->I
2FDOA	64	69	65	D-->E
2FDOA	64	69	67	I-->D
2FDOA	64	69	67	I-->E
2FDOA	64	69	67	I-->V
2FDOA	85	89	85	E-->A

2FDOA	85 - 89	85	E-->C
2FDOA	85 - 89	85	E-->H
2FDOA	85 - 89	85	E-->I
2FDOA	85 - 89	85	E-->P
2FDOA	85 - 89	85	E-->Q
2FDOA	85 - 89	85	E-->S
2FDOA	85 - 89	85	E-->T
2FDOA	85 - 89	85	E-->V
2FDOA	85 - 89	86	A-->K
2FDOA	85 - 89	86	A-->L
2FDOA	85 - 89	86	A-->N
2FDOA	85 - 89	86	A-->P
2FDOA	85 - 89	87	G-->E
2FDOA	85 - 89	87	G-->K
2FDOA	85 - 89	87	G-->P
2FDOA	85 - 89	87	G-->Q
2FDOA	85 - 89	88	R-->A
2FDOA	85 - 89	88	R-->D
2FDOA	85 - 89	88	R-->E
2FDOA	85 - 89	88	R-->F
2FDOA	85 - 89	88	R-->I
2FDOA	85 - 89	88	R-->N
2FDOA	85 - 89	88	R-->P
2FDOA	85 - 89	88	R-->T
2FDOA	85 - 89	88	R-->V
2FDOA	85 - 89	88	R-->Y
2FDOA	85 - 89	89	N-->A

2FDOA	85	89	89	N-->D
2FDOA	85	89	89	N-->E
2FDOA	85	89	89	N-->F
2FDOA	85	89	89	N-->G
2FDOA	85	89	89	N-->P
2FDOA	85	89	89	N-->R
2FDOA	85	89	89	N-->V
2FDOA	85	89	89	N-->Y
1AVQC	22	26	22	D-->F
1AVQC	22	26	23	A-->P
1AVQC	22	26	24	W-->F
1AVQC	22	26	24	W-->S
1AVQC	22	26	24	W-->V
1AVQC	22	26	25	H-->V
1KZ7A	673	677	673	T-->H
1KZ7A	673	677	673	T-->I
1KZ7A	673	677	673	T-->K
1KZ7A	673	677	673	T-->N
1KZ7A	673	677	673	T-->P
1KZ7A	673	677	673	T-->Q
1KZ7A	673	677	674	G-->C
1KZ7A	673	677	674	G-->D
1KZ7A	673	677	674	G-->F
1KZ7A	673	677	674	G-->K
1KZ7A	673	677	674	G-->L
1KZ7A	673	677	674	G-->N
1KZ7A	673	677	674	G-->P

1KZ7A	673 - 677	674	G-->S	
1KZ7A	673 - 677	674	G-->V	
1KZ7A	673 - 677	674	G-->Y	
1KZ7A	673 - 677	675	L-->A	
1KZ7A	673 - 677	675	L-->G	
1KZ7A	673 - 677	675	L-->H	
1KZ7A	673 - 677	675	L-->I	
1KZ7A	673 - 677	675	L-->R	
1KZ7A	673 - 677	675	L-->T	
1KZ7A	673 - 677	676	Q-->A	
1KZ7A	673 - 677	676	Q-->F	
1KZ7A	673 - 677	676	Q-->G	
1KZ7A	673 - 677	676	Q-->H	
1KZ7A	673 - 677	676	Q-->I	
1KZ7A	673 - 677	676	Q-->L	
1KZ7A	673 - 677	676	Q-->R	
1KZ7A	673 - 677	676	Q-->S	
1KZ7A	673 - 677	676	Q-->V	
1KZ7A	673 - 677	677	N-->D	
1KZ7A	673 - 677	677	N-->P	
1KZ7A	673 - 677	677	N-->Q	
1KZ7A	673 - 677	677	N-->S	
1KZ7A	673 - 677	677	N-->Y	
1KZ7A	679 - 683	679	K-->A	
1KZ7A	679 - 683	679	K-->D	
1KZ7A	679 - 683	679	K-->E	
1KZ7A	679 - 683	679	K-->H	

1KZ7A	679 - 683	679	K-->I
1KZ7A	679 - 683	679	K-->N
1KZ7A	679 - 683	679	K-->P
1KZ7A	679 - 683	679	K-->T
1KZ7A	679 - 683	679	K-->V
1KZ7A	679 - 683	679	K-->Y
1KZ7A	679 - 683	680	N-->A
1KZ7A	679 - 683	680	N-->F
1KZ7A	679 - 683	680	N-->G
1KZ7A	679 - 683	680	N-->K
1KZ7A	679 - 683	680	N-->L
1KZ7A	679 - 683	680	N-->R
1KZ7A	679 - 683	680	N-->S
1KZ7A	679 - 683	680	N-->V
1KZ7A	679 - 683	681	I-->D
1KZ7A	679 - 683	681	I-->G
1KZ7A	679 - 683	681	I-->H
1KZ7A	679 - 683	681	I-->K
1KZ7A	679 - 683	681	I-->L
1KZ7A	679 - 683	681	I-->M
1KZ7A	679 - 683	681	I-->Q
1KZ7A	679 - 683	681	I-->T
1KZ7A	679 - 683	681	I-->V
1KZ7A	679 - 683	681	I-->W
1KZ7A	679 - 683	682	L-->A
1KZ7A	679 - 683	682	L-->D
1KZ7A	679 - 683	682	L-->F

1KZ7A	679 - 683	682	L-->H
1KZ7A	679 - 683	682	L-->I
1KZ7A	679 - 683	682	L-->M
1KZ7A	679 - 683	682	L-->N
1KZ7A	679 - 683	682	L-->P
1KZ7A	679 - 683	682	L-->S
1KZ7A	679 - 683	682	L-->V
1KZ7A	679 - 683	683	F-->A
1KZ7A	679 - 683	683	F-->I
1KZ7A	679 - 683	683	F-->K
1KZ7A	679 - 683	683	F-->L
1KZ7A	679 - 683	683	F-->M
1KZ7A	679 - 683	683	F-->N
1KZ7A	679 - 683	683	F-->P
1KZ7A	679 - 683	683	F-->Q
1KZ7A	679 - 683	683	F-->R
1KZ7A	679 - 683	683	F-->S
1KZ7A	679 - 683	683	F-->V
1KZ7A	679 - 683	683	F-->Y
1KZ7A	697 - 702	699	R-->D
1KZ7A	697 - 702	701	L-->N
1KZ7A	761 - 765	761	L-->D
1KZ7A	761 - 765	761	L-->F
1KZ7A	761 - 765	761	L-->G
1KZ7A	761 - 765	761	L-->H
1KZ7A	761 - 765	761	L-->P
1KZ7A	761 - 765	762	D-->A

1KZ7A	761 - 765	762	D-->G
1KZ7A	761 - 765	762	D-->I
1KZ7A	761 - 765	762	D-->P
1KZ7A	761 - 765	762	D-->Q
1KZ7A	761 - 765	762	D-->T
1KZ7A	761 - 765	762	D-->V
1KZ7A	761 - 765	763	S-->G
1KZ7A	761 - 765	763	S-->K
1KZ7A	761 - 765	763	S-->L
1KZ7A	761 - 765	763	S-->M
1KZ7A	761 - 765	763	S-->Y
1KZ7A	761 - 765	764	Y-->A
1KZ7A	761 - 765	764	Y-->C
1KZ7A	761 - 765	764	Y-->D
1KZ7A	761 - 765	764	Y-->G
1KZ7A	761 - 765	764	Y-->H
1KZ7A	761 - 765	764	Y-->I
1KZ7A	761 - 765	764	Y-->K
1KZ7A	761 - 765	764	Y-->L
1KZ7A	761 - 765	764	Y-->N
1KZ7A	761 - 765	764	Y-->P
1KZ7A	761 - 765	764	Y-->R
1KZ7A	761 - 765	764	Y-->S
1KZ7A	761 - 765	765	L-->E
1KZ7A	761 - 765	765	L-->G
1KZ7A	761 - 765	765	L-->P
1KZ7A	761 - 765	765	L-->V

1KZ7A	761 - 765	765	L-->Y	
1VYQA	92 - 96	92	S-->E	
1VYQA	92 - 96	92	S-->K	
1VYQA	92 - 96	92	S-->L	
1VYQA	92 - 96	92	S-->R	
1VYQA	92 - 96	92	S-->T	
1VYQA	92 - 96	92	S-->V	
1VYQA	92 - 96	93	S-->E	
1VYQA	92 - 96	93	S-->G	
1VYQA	92 - 96	93	S-->H	
1VYQA	92 - 96	93	S-->L	
1VYQA	92 - 96	93	S-->N	
1VYQA	92 - 96	93	S-->P	
1VYQA	92 - 96	93	S-->V	
1VYQA	92 - 96	93	S-->Y	
1VYQA	92 - 96	94	I-->D	
1VYQA	92 - 96	94	I-->E	
1VYQA	92 - 96	94	I-->F	
1VYQA	92 - 96	94	I-->G	
1VYQA	92 - 96	94	I-->K	
1VYQA	92 - 96	94	I-->N	
1VYQA	92 - 96	94	I-->P	
1VYQA	92 - 96	94	I-->S	
1VYQA	92 - 96	94	I-->T	
1VYQA	92 - 96	94	I-->V	
1VYQA	92 - 96	94	I-->W	
1VYQA	92 - 96	94	I-->Y	

1VYQA	92 - 96	95	S-->D
1VYQA	92 - 96	95	S-->E
1VYQA	92 - 96	95	S-->G
1VYQA	92 - 96	95	S-->H
1VYQA	92 - 96	95	S-->I
1VYQA	92 - 96	95	S-->K
1VYQA	92 - 96	95	S-->L
1VYQA	92 - 96	95	S-->N
1VYQA	92 - 96	95	S-->T
1VYQA	92 - 96	95	S-->Y
1VYQA	92 - 96	96	K-->A
1VYQA	92 - 96	96	K-->E
1VYQA	92 - 96	96	K-->F
1VYQA	92 - 96	96	K-->G
1VYQA	92 - 96	96	K-->I
1VYQA	92 - 96	96	K-->L
1VYQA	92 - 96	96	K-->M
1VYQA	92 - 96	96	K-->S
1VYQA	92 - 96	96	K-->T
1VYQA	92 - 96	96	K-->V
1VYQA	92 - 96	96	K-->W
1VYQA	92 - 96	96	K-->Y
2AAWC	120 - 125	121	T-->V
2PG3A	120 - 124	120	T-->A
2PG3A	120 - 124	120	T-->D
2PG3A	120 - 124	122	S-->A
2PG3A	120 - 124	122	S-->M

2PG3A	120 - 124	123	L-->G	
2PG3A	120 - 124	123	L-->S	
2PG3A	222 - 228	225	K-->G	
3CEBA	45 - 50	45	L-->K	
3CEBA	45 - 50	46	A-->I	
3CEBA	45 - 50	46	A-->L	
3CEBA	45 - 50	49	I-->Y	
3CEBA	175 - 180	178	L-->V	
2BE3B	49 - 54	50	E-->G	
2BE3B	49 - 54	54	E-->T	
2AYTA	71 - 76	74	T-->N	
2AYTA	125 - 131	131	L-->V	
2AYTA	179 - 184	179	Q-->V	
2AYTA	179 - 184	184	Q-->R	
2AYTA	179 - 184	184	Q-->V	
1LTGA	13 - 18	15	E-->L	
1LTGA	13 - 18	16	I-->G	
1LTGA	41 - 45	41	L-->H	
1LTGA	41 - 45	41	L-->R	
1LTGA	41 - 45	42	Y-->I	
1LTGA	41 - 45	42	Y-->L	
1LTGA	41 - 45	42	Y-->S	
1LTGA	41 - 45	43	D-->R	
1LTGA	41 - 45	43	D-->S	
1LTGA	41 - 45	44	H-->D	
1LTGA	41 - 45	44	H-->G	
1LTGA	41 - 45	44	H-->I	

1LTGA	41	- 45	44	H-->K
1LTGA	41	- 45	44	H-->L
1LTGA	41	- 45	44	H-->P
1LTGA	41	- 45	44	H-->Y
1LTGA	41	- 45	45	A-->N
1LTGA	41	- 45	45	A-->R
1LTGA	41	- 45	45	A-->S
1LTGA	158	- 164	160	D-->K
2IXBA	349	- 354	353	K-->P
2ANUA	179	- 183	179	N-->A
2ANUA	179	- 183	179	N-->C
2ANUA	179	- 183	179	N-->G
2ANUA	179	- 183	179	N-->H
2ANUA	179	- 183	179	N-->I
2ANUA	179	- 183	179	N-->K
2ANUA	179	- 183	179	N-->L
2ANUA	179	- 183	179	N-->P
2ANUA	179	- 183	179	N-->Q
2ANUA	179	- 183	179	N-->R
2ANUA	179	- 183	179	N-->S
2ANUA	179	- 183	179	N-->V
2ANUA	179	- 183	179	N-->W
2ANUA	179	- 183	180	S-->A
2ANUA	179	- 183	180	S-->G
2ANUA	179	- 183	180	S-->L
2ANUA	179	- 183	180	S-->V
2ANUA	179	- 183	181	V-->A

2ANUA	179 - 183	181	V-->E
2ANUA	179 - 183	181	V-->F
2ANUA	179 - 183	181	V-->G
2ANUA	179 - 183	181	V-->I
2ANUA	179 - 183	181	V-->K
2ANUA	179 - 183	181	V-->L
2ANUA	179 - 183	181	V-->N
2ANUA	179 - 183	181	V-->Q
2ANUA	179 - 183	181	V-->S
2ANUA	179 - 183	181	V-->Y
2ANUA	179 - 183	182	G-->A
2ANUA	179 - 183	182	G-->E
2ANUA	179 - 183	182	G-->H
2ANUA	179 - 183	182	G-->I
2ANUA	179 - 183	182	G-->L
2ANUA	179 - 183	182	G-->R
2ANUA	179 - 183	182	G-->S
2ANUA	179 - 183	182	G-->T
2ANUA	179 - 183	182	G-->V
2ANUA	179 - 183	183	V-->D
2ANUA	179 - 183	183	V-->F
2ANUA	179 - 183	183	V-->K
2ANUA	179 - 183	183	V-->L
2ANUA	179 - 183	183	V-->N
2ANUA	179 - 183	183	V-->P
2ANUA	179 - 183	183	V-->Q
2ANUA	179 - 183	183	V-->R

1Y9KA	125 - 129	125	Y-->R	
1Y9KA	125 - 129	125	Y-->T	
1Y9KA	125 - 129	127	S-->P	
1Y9KA	125 - 129	128	K-->A	
1Y9KA	125 - 129	128	K-->Y	
1Y9KA	125 - 129	129	H-->D	
1Y9KA	125 - 129	129	H-->E	
1Y9KA	125 - 129	129	H-->G	
1Y9KA	125 - 129	129	H-->K	
1Y9KA	125 - 129	129	H-->R	
1Y9KA	125 - 129	129	H-->T	
1Y9KA	125 - 129	129	H-->V	
2GSHA	177 - 182	178	D-->L	
2GSHA	177 - 182	178	D-->V	
2GSHA	177 - 182	179	L-->K	
2GSHA	177 - 182	179	L-->T	
2GSHA	177 - 182	180	A-->G	
2GSHA	177 - 182	181	K-->D	
2GSHA	177 - 182	181	K-->F	
1VLIA	177 - 183	177	P-->A	
1VLIA	177 - 183	181	K-->G	
1VLIA	177 - 183	181	K-->L	
1VLIA	177 - 183	181	K-->V	
1VLIA	177 - 183	182	E-->A	
1VLIA	177 - 183	182	E-->C	
2Q2CA	246 - 250	246	L-->F	
2Q2CA	246 - 250	246	L-->K	

2Q2CA	246 - 250	246	L-->P
2Q2CA	246 - 250	246	L-->S
2Q2CA	246 - 250	246	L-->W
2Q2CA	246 - 250	247	D-->A
2Q2CA	246 - 250	247	D-->C
2Q2CA	246 - 250	247	D-->E
2Q2CA	246 - 250	247	D-->I
2Q2CA	246 - 250	247	D-->L
2Q2CA	246 - 250	247	D-->P
2Q2CA	246 - 250	247	D-->R
2Q2CA	246 - 250	247	D-->S
2Q2CA	246 - 250	247	D-->V
2Q2CA	246 - 250	247	D-->W
2Q2CA	246 - 250	248	R-->D
2Q2CA	246 - 250	248	R-->E
2Q2CA	246 - 250	248	R-->F
2Q2CA	246 - 250	248	R-->G
2Q2CA	246 - 250	248	R-->K
2Q2CA	246 - 250	248	R-->L
2Q2CA	246 - 250	248	R-->N
2Q2CA	246 - 250	248	R-->T
2Q2CA	246 - 250	248	R-->V
2Q2CA	246 - 250	249	L-->E
2Q2CA	246 - 250	249	L-->F
2Q2CA	246 - 250	249	L-->G
2Q2CA	246 - 250	249	L-->P
2Q2CA	246 - 250	249	L-->T

2Q2CA	246 - 250	249	L-->Y
2Q2CA	246 - 250	250	K-->G
2Q2CA	246 - 250	250	K-->L
2Q2CA	246 - 250	250	K-->N
2Q2CA	246 - 250	250	K-->R
2Q2CA	246 - 250	250	K-->T
2Q2CA	246 - 250	250	K-->Y
3B6YB	93 - 99	98	S-->R
3B6YB	105 - 110	109	C-->D
3B6YB	105 - 110	109	C-->S
1K0KA	105 - 109	105	P-->A
1K0KA	105 - 109	105	P-->G
1K0KA	105 - 109	105	P-->K
1K0KA	105 - 109	105	P-->L
1K0KA	105 - 109	105	P-->Q
1K0KA	105 - 109	105	P-->R
1K0KA	105 - 109	105	P-->V
1K0KA	105 - 109	105	P-->Y
1K0KA	105 - 109	106	A-->D
1K0KA	105 - 109	106	A-->E
1K0KA	105 - 109	106	A-->H
1K0KA	105 - 109	106	A-->L
1K0KA	105 - 109	106	A-->N
1K0KA	105 - 109	106	A-->Q
1K0KA	105 - 109	106	A-->S
1K0KA	105 - 109	106	A-->V
1K0KA	105 - 109	107	G-->D

1K0KA	105 - 109	107	G-->H	
1K0KA	105 - 109	107	G-->L	
1K0KA	105 - 109	107	G-->V	
1K0KA	105 - 109	108	L-->A	
1K0KA	105 - 109	108	L-->C	
1K0KA	105 - 109	108	L-->K	
1K0KA	105 - 109	108	L-->M	
1K0KA	105 - 109	108	L-->R	
1K0KA	105 - 109	108	L-->T	
1K0KA	105 - 109	108	L-->V	
1K0KA	105 - 109	108	L-->W	
1K0KA	105 - 109	109	Q-->D	
1K0KA	105 - 109	109	Q-->E	
1K0KA	105 - 109	109	Q-->G	
1K0KA	105 - 109	109	Q-->I	
1K0KA	105 - 109	109	Q-->K	
1K0KA	105 - 109	109	Q-->L	
1K0KA	105 - 109	109	Q-->P	
1K0KA	105 - 109	109	Q-->T	
1K0KA	105 - 109	109	Q-->V	
2QPVA	127 - 132	127	L-->G	
2QPVA	127 - 132	131	L-->S	
2QPVA	127 - 132	132	E-->L	
2QPVA	127 - 132	132	E-->P	
2QPVA	127 - 132	132	E-->S	
2QPVA	127 - 132	132	E-->V	
2AUAA	92 - 96	92	V-->P	

2AUAA	92 - 96	93	R-->A	
2AUAA	92 - 96	93	R-->D	
2AUAA	92 - 96	93	R-->E	
2AUAA	92 - 96	93	R-->G	
2AUAA	92 - 96	93	R-->H	
2AUAA	92 - 96	93	R-->I	
2AUAA	92 - 96	93	R-->K	
2AUAA	92 - 96	93	R-->L	
2AUAA	92 - 96	93	R-->N	
2AUAA	92 - 96	93	R-->Q	
2AUAA	92 - 96	93	R-->S	
2AUAA	92 - 96	93	R-->T	
2AUAA	92 - 96	93	R-->V	
2AUAA	92 - 96	93	R-->Y	
2AUAA	92 - 96	94	L-->N	
2AUAA	92 - 96	94	L-->Q	
2AUAA	92 - 96	94	L-->T	
2AUAA	92 - 96	94	L-->V	
2AUAA	92 - 96	94	L-->Y	
2AUAA	92 - 96	95	Q-->A	
2AUAA	92 - 96	95	Q-->D	
2AUAA	92 - 96	95	Q-->E	
2AUAA	92 - 96	95	Q-->G	
2AUAA	92 - 96	95	Q-->L	
2AUAA	92 - 96	95	Q-->N	
2AUAA	92 - 96	95	Q-->R	
2AUAA	92 - 96	95	Q-->S	

2AUAA	92 - 96	95	Q-->T
2AUAA	92 - 96	95	Q-->V
2AUAA	92 - 96	96	E-->F
2AUAA	92 - 96	96	E-->G
2AUAA	92 - 96	96	E-->H
2AUAA	92 - 96	96	E-->L
2AUAA	92 - 96	96	E-->N
2AUAA	92 - 96	96	E-->Q
2AUAA	92 - 96	96	E-->S
2AUAA	92 - 96	96	E-->T
1WAMA	221 - 226	223	E-->Q
1WAMA	221 - 226	224	R-->G
1WAMA	221 - 226	225	T-->A
1WAMA	221 - 226	225	T-->V
1WAMA	221 - 226	226	H-->G
1WAMA	221 - 226	226	H-->Y
2IYBG	414 - 418	414	V-->E
2IYBG	414 - 418	414	V-->G
2IYBG	414 - 418	414	V-->L
2IYBG	414 - 418	414	V-->N
2IYBG	414 - 418	415	E-->D
2IYBG	414 - 418	415	E-->N
2IYBG	414 - 418	416	C-->A
2IYBG	414 - 418	416	C-->I
2IYBG	414 - 418	416	C-->L
2IYBG	414 - 418	416	C-->M
2IYBG	414 - 418	416	C-->N

2IYBG	414	418	416	C-->P
2IYBG	414	418	416	C-->R
2IYBG	414	418	416	C-->S
2IYBG	414	418	416	C-->V
2IYBG	414	418	417	K-->T
2IYBG	414	418	418	K-->C
2IYBG	414	418	418	K-->G
2IYBG	414	418	418	K-->M
2IYBG	414	418	418	K-->T
2IYBG	414	418	418	K-->V
1YYVA	48	53	48	F-->G
1YYVA	48	53	48	F-->L
1YYVA	48	53	50	D-->G
1YYVA	48	53	51	L-->N
1YYVA	48	53	51	L-->Y
1YYVA	48	53	53	R-->E
1VLDN	103	108	103	K-->I
1VLDN	103	108	103	K-->L
1VLDN	103	108	104	E-->A
1VLDN	103	108	104	E-->F
1VLDN	103	108	104	E-->S
1VLDN	103	108	107	D-->H
1VLDN	103	107	103	R-->A
1VLDN	103	107	103	R-->K
1VLDN	103	107	103	R-->S
1VLDN	103	107	104	C-->F
1VLDN	103	107	104	C-->G

1VLDN	103	107	105	A-->P
1VLDN	103	107	107	N-->F
2CZGA	55	59	55	P-->G
2CZGA	55	59	56	A-->C
2CZGA	55	59	56	A-->D
2CZGA	55	59	56	A-->E
2CZGA	55	59	56	A-->G
2CZGA	55	59	56	A-->M
2CZGA	55	59	57	M-->A
2CZGA	55	59	57	M-->G
2CZGA	55	59	57	M-->H
2CZGA	55	59	57	M-->K
2CZGA	55	59	57	M-->N
2CZGA	55	59	57	M-->P
2CZGA	55	59	57	M-->V
2CZGA	55	59	57	M-->Y
2CZGA	55	59	58	Q-->F
2CZGA	55	59	58	Q-->T
2CZGA	55	59	58	Q-->V
2CZGA	55	59	58	Q-->Y
2CZGA	55	59	59	V-->F
2HJ9C	232	236	232	V-->A
2HJ9C	232	236	232	V-->D
2HJ9C	232	236	232	V-->E
2HJ9C	232	236	232	V-->F
2HJ9C	232	236	232	V-->G
2HJ9C	232	236	232	V-->K

2HJ9C	232 - 236	232	V-->L
2HJ9C	232 - 236	232	V-->P
2HJ9C	232 - 236	232	V-->R
2HJ9C	232 - 236	232	V-->S
2HJ9C	232 - 236	232	V-->T
2HJ9C	232 - 236	233	A-->D
2HJ9C	232 - 236	233	A-->E
2HJ9C	232 - 236	233	A-->F
2HJ9C	232 - 236	233	A-->G
2HJ9C	232 - 236	233	A-->H
2HJ9C	232 - 236	233	A-->I
2HJ9C	232 - 236	233	A-->L
2HJ9C	232 - 236	233	A-->N
2HJ9C	232 - 236	233	A-->P
2HJ9C	232 - 236	233	A-->Q
2HJ9C	232 - 236	233	A-->R
2HJ9C	232 - 236	233	A-->V
2HJ9C	232 - 236	234	D-->A
2HJ9C	232 - 236	234	D-->E
2HJ9C	232 - 236	234	D-->G
2HJ9C	232 - 236	234	D-->I
2HJ9C	232 - 236	234	D-->K
2HJ9C	232 - 236	234	D-->L
2HJ9C	232 - 236	234	D-->N
2HJ9C	232 - 236	234	D-->R
2HJ9C	232 - 236	234	D-->S
2HJ9C	232 - 236	234	D-->T

2HJ9C	232 - 236	234	D-->V
2HJ9C	232 - 236	235	V-->A
2HJ9C	232 - 236	235	V-->D
2HJ9C	232 - 236	235	V-->F
2HJ9C	232 - 236	235	V-->G
2HJ9C	232 - 236	235	V-->P
2HJ9C	232 - 236	235	V-->T
2HJ9C	232 - 236	236	L-->E
2HJ9C	232 - 236	236	L-->G
2HJ9C	232 - 236	236	L-->H
2HJ9C	232 - 236	236	L-->N
2HJ9C	232 - 236	236	L-->P
2HJ9C	232 - 236	236	L-->R
2HJ9C	232 - 236	236	L-->S
2HJ9C	232 - 236	236	L-->T
2HJ9C	232 - 236	236	L-->V
2HJ9C	232 - 236	236	L-->Y
1TU3F	841 - 847	844	I-->Y
2QEOA	18 - 22	18	L-->D
2QEOA	18 - 22	18	L-->G
2QEOA	18 - 22	18	L-->S
2QEOA	18 - 22	19	C-->G
2QEOA	18 - 22	19	C-->K
2QEOA	18 - 22	19	C-->L
2QEOA	18 - 22	19	C-->Q
2QEOA	18 - 22	19	C-->S
2QEOA	18 - 22	19	C-->Y

2QEOA	18	22	20	E-->D
2QEOA	18	22	20	E-->Y
2QEOA	18	22	21	I-->F
1NDIA	248	253	249	G-->I
1NDIA	248	253	249	G-->Y
1NDIA	248	253	250	I-->D
1NDIA	248	253	250	I-->P
1NDIA	248	253	251	L-->V
1NDIA	248	253	252	T-->G
1NDIA	420	424	420	D-->R
1NDIA	420	424	420	D-->V
1NDIA	420	424	420	D-->Y
1NDIA	420	424	421	F-->G
1NDIA	420	424	421	F-->V
1NDIA	420	424	422	P-->D
1NDIA	420	424	422	P-->N
1NDIA	420	424	422	P-->R
1NDIA	420	424	422	P-->W
1NDIA	420	424	423	K-->A
1NDIA	420	424	423	K-->D
1NDIA	420	424	423	K-->E
1NDIA	420	424	423	K-->L
1NDIA	420	424	423	K-->Q
1NDIA	420	424	423	K-->R
1NDIA	420	424	423	K-->T
1NDIA	420	424	423	K-->V
1NDIA	420	424	424	S-->G

1NDIA	420 - 424	424	S-->N
1NDIA	420 - 424	424	S-->W
1NDIA	420 - 424	424	S-->Y
1NDIA	541 - 546	545	I-->P
1R5XA	78 - 83	80	D-->Y
1R5XA	78 - 83	83	L-->I
1R5XA	78 - 83	83	L-->S
2BWBI	361 - 367	364	A-->S
2Z99A	171 - 177	171	E-->C
2PIFB	129 - 133	129	E-->F
2PIFB	129 - 133	129	E-->G
2PIFB	129 - 133	129	E-->I
2PIFB	129 - 133	129	E-->S
2PIFB	129 - 133	129	E-->V
2PIFB	129 - 133	130	T-->E
2PIFB	129 - 133	130	T-->F
2PIFB	129 - 133	130	T-->G
2PIFB	129 - 133	130	T-->K
2PIFB	129 - 133	130	T-->N
2PIFB	129 - 133	130	T-->Q
2PIFB	129 - 133	130	T-->R
2PIFB	129 - 133	130	T-->V
2PIFB	129 - 133	131	P-->E
2PIFB	129 - 133	131	P-->R
2PIFB	129 - 133	132	L-->A
2PIFB	129 - 133	132	L-->F
2PIFB	129 - 133	132	L-->H

2PIFB	129 - 133	132	L-->I
2PIFB	129 - 133	132	L-->K
2PIFB	129 - 133	132	L-->S
2PIFB	129 - 133	132	L-->Y
2PIFB	129 - 133	133	Q-->F
2PIFB	129 - 133	133	Q-->G
2PIFB	129 - 133	133	Q-->I
2PIFB	129 - 133	133	Q-->K
2PIFB	129 - 133	133	Q-->P
2PIFB	129 - 133	133	Q-->R
2PIFB	129 - 133	133	Q-->V
2PIFB	129 - 133	133	Q-->Y
2PIFB	129 - 133	129	T-->A
2PIFB	129 - 133	131	Q-->T
2PIFB	129 - 133	132	A-->Q
2PIFB	129 - 133	133	A-->R
2EGIG	44 - 49	46	K-->A
1N5LA	143 - 148	144	E-->K
1N5LA	143 - 148	144	E-->L
1N5LA	143 - 148	144	E-->V
1N5LA	143 - 148	146	A-->L
1N5LA	143 - 148	146	A-->V
1N5LA	143 - 148	147	G-->V
2Q89A	115 - 119	115	Y-->I
2Q89A	115 - 119	115	Y-->L
2Q89A	115 - 119	115	Y-->N
2Q89A	115 - 119	115	Y-->P

2Q89A	115 - 119	115	Y-->R
2Q89A	115 - 119	115	Y-->T
2Q89A	115 - 119	115	Y-->V
2Q89A	115 - 119	116	K-->A
2Q89A	115 - 119	116	K-->F
2Q89A	115 - 119	116	K-->I
2Q89A	115 - 119	116	K-->N
2Q89A	115 - 119	116	K-->Q
2Q89A	115 - 119	117	D-->G
2Q89A	115 - 119	117	D-->H
2Q89A	115 - 119	117	D-->N
2Q89A	115 - 119	117	D-->V
2Q89A	115 - 119	118	I-->D
2Q89A	115 - 119	118	I-->G
2Q89A	115 - 119	118	I-->K
2Q89A	115 - 119	118	I-->N
2Q89A	115 - 119	118	I-->P
2Q89A	115 - 119	118	I-->T
2Q89A	115 - 119	118	I-->V
2Q89A	115 - 119	118	I-->Y
2Q89A	115 - 119	119	A-->D
2Q89A	115 - 119	119	A-->H
2Q89A	115 - 119	119	A-->K
2Q89A	115 - 119	119	A-->L
2Q89A	115 - 119	119	A-->N
2Q89A	115 - 119	119	A-->T
2Q89A	115 - 119	119	A-->Y

2Q89A	241 - 245	241	A-->H	
2Q89A	241 - 245	241	A-->K	
2Q89A	241 - 245	242	K-->A	
2Q89A	241 - 245	242	K-->F	
2Q89A	241 - 245	242	K-->I	
2Q89A	241 - 245	242	K-->L	
2Q89A	241 - 245	242	K-->T	
2Q89A	241 - 245	243	A-->G	
2Q89A	241 - 245	243	A-->N	
2Q89A	241 - 245	243	A-->R	
2Q89A	241 - 245	243	A-->T	
2Q89A	241 - 245	243	A-->V	
2Q89A	241 - 245	244	A-->G	
2Q89A	241 - 245	244	A-->I	
2Q89A	241 - 245	244	A-->L	
2Q89A	241 - 245	244	A-->N	
2Q89A	241 - 245	245	M-->A	
2Q89A	241 - 245	245	M-->G	
2Q89A	241 - 245	245	M-->H	
2Q89A	241 - 245	245	M-->I	
2Q89A	241 - 245	245	M-->K	
2Q89A	241 - 245	245	M-->L	
2Q89A	241 - 245	245	M-->P	
2Q89A	241 - 245	245	M-->Y	
2Q89A	249 - 253	249	R-->E	
2Q89A	249 - 253	249	R-->L	
2Q89A	249 - 253	249	R-->T	

2Q89A	249 - 253	249	R-->V
2Q89A	249 - 253	250	E-->C
2Q89A	249 - 253	250	E-->D
2Q89A	249 - 253	250	E-->S
2Q89A	249 - 253	252	L-->G
2Q89A	249 - 253	252	L-->H
2Q89A	249 - 253	252	L-->Y
2Q89A	249 - 253	253	C-->A
2Q89A	249 - 253	253	C-->L
2Q89A	249 - 253	253	C-->N
2Q89A	249 - 253	253	C-->P
2Q89A	249 - 253	253	C-->R
2Q89A	249 - 253	253	C-->T
2Q89A	249 - 253	253	C-->V
2OB9A	4 - 9	7	Q-->R
2OB9A	4 - 9	9	A-->G
1FQKC	148 - 152	148	A-->D
1FQKC	148 - 152	148	A-->E
1FQKC	148 - 152	148	A-->F
1FQKC	148 - 152	148	A-->I
1FQKC	148 - 152	148	A-->L
1FQKC	148 - 152	148	A-->P
1FQKC	148 - 152	148	A-->R
1FQKC	148 - 152	148	A-->T
1FQKC	148 - 152	148	A-->V
1FQKC	148 - 152	148	A-->Y
1FQKC	148 - 152	149	G-->R

1FQKC	148 - 152	150	Y-->D
1FQKC	148 - 152	150	Y-->E
1FQKC	148 - 152	150	Y-->F
1FQKC	148 - 152	150	Y-->K
1FQKC	148 - 152	150	Y-->L
1FQKC	148 - 152	150	Y-->M
1FQKC	148 - 152	150	Y-->P
1FQKC	148 - 152	150	Y-->Q
1FQKC	148 - 152	150	Y-->R
1FQKC	148 - 152	150	Y-->S
1FQKC	148 - 152	150	Y-->T
1FQKC	148 - 152	150	Y-->V
1FQKC	148 - 152	151	Y-->A
1FQKC	148 - 152	151	Y-->D
1FQKC	148 - 152	151	Y-->E
1FQKC	148 - 152	151	Y-->F
1FQKC	148 - 152	151	Y-->G
1FQKC	148 - 152	151	Y-->I
1FQKC	148 - 152	151	Y-->K
1FQKC	148 - 152	151	Y-->L
1FQKC	148 - 152	151	Y-->N
1FQKC	148 - 152	151	Y-->P
1FQKC	148 - 152	151	Y-->R
1FQKC	148 - 152	151	Y-->S
1FQKC	148 - 152	151	Y-->V
1FQKC	148 - 152	152	L-->C
1FQKC	148 - 152	152	L-->H

1FQKC	148	152	152	L-->N
1FQKC	148	152	152	L-->T
1FQKC	148	152	152	L-->V
1FQKC	148	152	152	L-->Y
1FQKC	167	172	170	V-->H
1FQKC	167	172	170	V-->P
1FQKC	223	227	223	L-->A
1FQKC	223	227	223	L-->D
1FQKC	223	227	223	L-->K
1FQKC	223	227	223	L-->M
1FQKC	223	227	223	L-->Q
1FQKC	223	227	223	L-->V
1FQKC	223	227	224	S-->D
1FQKC	223	227	224	S-->F
1FQKC	223	227	224	S-->G
1FQKC	223	227	224	S-->I
1FQKC	223	227	224	S-->K
1FQKC	223	227	224	S-->P
1FQKC	223	227	224	S-->Q
1FQKC	223	227	224	S-->R
1FQKC	223	227	224	S-->T
1FQKC	223	227	224	S-->V
1FQKC	223	227	224	S-->Y
1FQKC	223	227	225	D-->F
1FQKC	223	227	225	D-->I
1FQKC	223	227	225	D-->K
1FQKC	223	227	225	D-->L

1FQKC	223 - 227	225	D-->N
1FQKC	223 - 227	225	D-->P
1FQKC	223 - 227	226	Y-->D
1FQKC	223 - 227	226	Y-->G
1FQKC	223 - 227	226	Y-->I
1FQKC	223 - 227	226	Y-->L
1FQKC	223 - 227	226	Y-->N
1FQKC	223 - 227	226	Y-->P
1FQKC	223 - 227	226	Y-->R
1FQKC	223 - 227	226	Y-->S
1FQKC	223 - 227	226	Y-->T
1FQKC	223 - 227	227	D-->F
1FQKC	223 - 227	227	D-->G
1FQKC	223 - 227	227	D-->I
1FQKC	223 - 227	227	D-->N
1FQKC	223 - 227	227	D-->P
1FQKC	223 - 227	227	D-->T
1FQKC	223 - 227	227	D-->V
1FQKC	223 - 227	227	D-->W
1FQKC	223 - 227	227	D-->Y
1CR5A	104 - 108	104	L-->G
1CR5A	104 - 108	104	L-->N
1CR5A	104 - 108	104	L-->R
1CR5A	104 - 108	104	L-->T
1CR5A	104 - 108	105	F-->E
1CR5A	104 - 108	105	F-->H
1CR5A	104 - 108	105	F-->K

1CR5A	104	108	105	F-->L
1CR5A	104	108	105	F-->N
1CR5A	104	108	105	F-->T
1CR5A	104	108	105	F-->V
1CR5A	104	108	106	K-->A
1CR5A	104	108	106	K-->G
1CR5A	104	108	106	K-->T
1CR5A	104	108	106	K-->V
1CR5A	104	108	107	Y-->D
1CR5A	104	108	107	Y-->F
1CR5A	104	108	107	Y-->G
1CR5A	104	108	107	Y-->H
1CR5A	104	108	107	Y-->I
1CR5A	104	108	107	Y-->S
1CR5A	104	108	107	Y-->T
1CR5A	104	108	107	Y-->V
1CR5A	104	108	108	S-->D
1CR5A	104	108	108	S-->K
1CR5A	104	108	108	S-->Q
2PMYB	69	73	69	R-->A
2PMYB	69	73	69	R-->D
2PMYB	69	73	69	R-->E
2PMYB	69	73	69	R-->G
2PMYB	69	73	69	R-->H
2PMYB	69	73	69	R-->K
2PMYB	69	73	69	R-->L
2PMYB	69	73	69	R-->Q

2PMYB	69 - 73	69	R-->T
2PMYB	69 - 73	69	R-->V
2PMYB	69 - 73	70	G-->C
2PMYB	69 - 73	70	G-->M
2PMYB	69 - 73	70	G-->R
2PMYB	69 - 73	70	G-->V
2PMYB	69 - 73	71	F-->A
2PMYB	69 - 73	71	F-->D
2PMYB	69 - 73	71	F-->E
2PMYB	69 - 73	71	F-->G
2PMYB	69 - 73	71	F-->I
2PMYB	69 - 73	71	F-->K
2PMYB	69 - 73	71	F-->L
2PMYB	69 - 73	71	F-->M
2PMYB	69 - 73	71	F-->N
2PMYB	69 - 73	71	F-->P
2PMYB	69 - 73	71	F-->Q
2PMYB	69 - 73	71	F-->R
2PMYB	69 - 73	71	F-->S
2PMYB	69 - 73	71	F-->V
2PMYB	69 - 73	72	L-->A
2PMYB	69 - 73	72	L-->D
2PMYB	69 - 73	72	L-->F
2PMYB	69 - 73	72	L-->G
2PMYB	69 - 73	72	L-->H
2PMYB	69 - 73	72	L-->P
2PMYB	69 - 73	72	L-->Q

2PMYB	69 - 73	72	L-->S
2PMYB	69 - 73	72	L-->T
2PMYB	69 - 73	72	L-->V
2PMYB	69 - 73	73	G-->A
2PMYB	69 - 73	73	G-->E
2PMYB	69 - 73	73	G-->I
2PMYB	69 - 73	73	G-->L
2PMYB	69 - 73	73	G-->P
2PMYB	69 - 73	73	G-->S
2PMYB	69 - 73	73	G-->T
2PMYB	69 - 73	73	G-->Y
2OZZB	119 - 123	119	A-->D
2OZZB	119 - 123	119	A-->L
2OZZB	119 - 123	119	A-->N
2OZZB	119 - 123	119	A-->P
2OZZB	119 - 123	119	A-->T
2OZZB	119 - 123	120	D-->E
2OZZB	119 - 123	120	D-->G
2OZZB	119 - 123	120	D-->K
2OZZB	119 - 123	120	D-->Q
2OZZB	119 - 123	120	D-->R
2OZZB	119 - 123	120	D-->T
2OZZB	119 - 123	120	D-->W
2OZZB	119 - 123	121	Q-->A
2OZZB	119 - 123	121	Q-->E
2OZZB	119 - 123	121	Q-->F
2OZZB	119 - 123	121	Q-->G

2OZZB	119 - 123	121	Q-->I
2OZZB	119 - 123	121	Q-->K
2OZZB	119 - 123	121	Q-->L
2OZZB	119 - 123	121	Q-->P
2OZZB	119 - 123	121	Q-->T
2OZZB	119 - 123	121	Q-->V
2OZZB	119 - 123	121	Q-->Y
2OZZB	119 - 123	122	K-->A
2OZZB	119 - 123	122	K-->D
2OZZB	119 - 123	122	K-->I
2OZZB	119 - 123	122	K-->R
2OZZB	119 - 123	122	K-->Y
2OZZB	119 - 123	123	I-->E
2OZZB	119 - 123	123	I-->K
2OZZB	119 - 123	123	I-->M
2OZZB	119 - 123	123	I-->N
2OZZB	119 - 123	123	I-->P
2OZZB	119 - 123	123	I-->T
2OZZB	119 - 123	123	I-->V
2OZZB	119 - 123	123	I-->W
2OZZB	119 - 123	123	I-->Y
2OZZB	125 - 129	125	T-->C
2OZZB	125 - 129	125	T-->E
2OZZB	125 - 129	125	T-->G
2OZZB	125 - 129	125	T-->Q
2OZZB	125 - 129	125	T-->V
2OZZB	125 - 129	126	D-->E

2OZZB	125 - 129	126	D-->K
2OZZB	125 - 129	126	D-->P
2OZZB	125 - 129	126	D-->Q
2OZZB	125 - 129	126	D-->Y
2OZZB	125 - 129	127	V-->D
2OZZB	125 - 129	127	V-->E
2OZZB	125 - 129	127	V-->G
2OZZB	125 - 129	128	F-->D
2OZZB	125 - 129	128	F-->E
2OZZB	125 - 129	128	F-->K
2OZZB	125 - 129	128	F-->N
2OZZB	125 - 129	128	F-->T
2OZZB	125 - 129	128	F-->W
2OZZB	125 - 129	129	F-->D
2OZZB	125 - 129	129	F-->I
2OZZB	125 - 129	129	F-->L
2OZZB	125 - 129	129	F-->N
2OZZB	125 - 129	129	F-->S
2OZZB	125 - 129	129	F-->V
2OZZB	180 - 184	180	P-->D
2OZZB	180 - 184	180	P-->L
2OZZB	180 - 184	180	P-->T
2OZZB	180 - 184	180	P-->V
2OZZB	180 - 184	181	R-->G
2OZZB	180 - 184	182	F-->D
2OZZB	180 - 184	182	F-->I
2OZZB	180 - 184	182	F-->K

2OZZB	180	-	184	182	F-->T
2OZZB	180	-	184	182	F-->Y
2OZZB	180	-	184	183	L-->D
2OZZB	180	-	184	183	L-->H
2OZZB	180	-	184	184	Q-->C
2OZZB	180	-	184	184	Q-->E
2OZZB	180	-	184	184	Q-->F
2OZZB	180	-	184	184	Q-->K
2OZZB	180	-	184	184	Q-->R
2OZZB	180	-	184	184	Q-->V
2OZZB	180	-	184	184	Q-->W
1S7JA	127	-	133	129	Y-->K
1S7JA	153	-	158	153	E-->K
1S7JA	153	-	158	155	T-->I
1S7JA	153	-	158	155	T-->V
1S7JA	153	-	158	157	A-->N
1S7JA	163	-	168	163	F-->P
1S7JA	163	-	168	164	S-->G
1S7JA	163	-	168	164	S-->V
1S7JA	163	-	168	165	A-->G
1S7JA	163	-	168	166	L-->E
1S7JA	163	-	168	168	A-->K
1T3GB	425	-	431	430	A-->T
1T3GB	479	-	484	479	P-->S
1T3GB	479	-	484	480	N-->D
1T3GB	479	-	484	480	N-->G
1T3GB	479	-	484	481	Y-->A

1T3GB	479 - 484	481	Y-->V
1T3GB	479 - 484	483	V-->I
1T3GB	479 - 484	484	R-->A
1T3GB	488 - 492	488	S-->C
1T3GB	488 - 492	488	S-->E
1T3GB	488 - 492	488	S-->F
1T3GB	488 - 492	488	S-->G
1T3GB	488 - 492	488	S-->I
1T3GB	488 - 492	488	S-->R
1T3GB	488 - 492	488	S-->Y
1T3GB	488 - 492	489	I-->A
1T3GB	488 - 492	489	I-->D
1T3GB	488 - 492	489	I-->F
1T3GB	488 - 492	489	I-->R
1T3GB	488 - 492	489	I-->Y
1T3GB	488 - 492	490	F-->A
1T3GB	488 - 492	490	F-->G
1T3GB	488 - 492	490	F-->I
1T3GB	488 - 492	490	F-->N
1T3GB	488 - 492	490	F-->P
1T3GB	488 - 492	490	F-->V
1T3GB	488 - 492	491	E-->A
1T3GB	488 - 492	491	E-->D
1T3GB	488 - 492	491	E-->G
1T3GB	488 - 492	491	E-->I
1T3GB	488 - 492	491	E-->L
1T3GB	488 - 492	491	E-->T

1T3GB	488 - 492	491	E-->V
1T3GB	488 - 492	492	L-->E
1T3GB	488 - 492	492	L-->F
1T3GB	488 - 492	492	L-->K
1T3GB	488 - 492	492	L-->N
1T3GB	488 - 492	492	L-->R
1T3GB	488 - 492	492	L-->S
1T3GB	494 - 498	494	T-->A
1T3GB	494 - 498	494	T-->F
1T3GB	494 - 498	494	T-->G
1T3GB	494 - 498	494	T-->I
1T3GB	494 - 498	494	T-->K
1T3GB	494 - 498	494	T-->L
1T3GB	494 - 498	494	T-->S
1T3GB	494 - 498	494	T-->V
1T3GB	494 - 498	495	R-->C
1T3GB	494 - 498	495	R-->G
1T3GB	494 - 498	495	R-->I
1T3GB	494 - 498	495	R-->L
1T3GB	494 - 498	495	R-->N
1T3GB	494 - 498	495	R-->T
1T3GB	494 - 498	495	R-->W
1T3GB	494 - 498	496	L-->H
1T3GB	494 - 498	496	L-->N
1T3GB	494 - 498	496	L-->Q
1T3GB	494 - 498	497	R-->D
1T3GB	494 - 498	497	R-->E

1T3GB	494	- 498	497	R-->G
1T3GB	494	- 498	497	R-->H
1T3GB	494	- 498	497	R-->I
1T3GB	494	- 498	497	R-->L
1T3GB	494	- 498	497	R-->P
1T3GB	494	- 498	498	N-->G
1T3GB	494	- 498	498	N-->I
1T3GB	494	- 498	498	N-->L
1T3GB	494	- 498	498	N-->R
1T3GB	494	- 498	498	N-->S
1T3GB	494	- 498	498	N-->T
1T3GB	494	- 498	498	N-->V
1WMIA	9	- 15	12	V-->A
1Q67B	171	- 176	171	A-->L
1Q67B	171	- 176	171	A-->S
1Q67B	171	- 176	173	E-->G
1Q67B	171	- 176	174	D-->I
1Q67B	171	- 176	174	D-->S
2JEVA	148	- 152	148	L-->E
2JEVA	148	- 152	148	L-->I
2JEVA	148	- 152	148	L-->K
2JEVA	148	- 152	148	L-->P
2JEVA	148	- 152	148	L-->R
2JEVA	148	- 152	148	L-->S
2JEVA	148	- 152	148	L-->Y
2JEVA	148	- 152	149	S-->D
2JEVA	148	- 152	149	S-->E

2JEVA	148 - 152	149	S-->G
2JEVA	148 - 152	149	S-->K
2JEVA	148 - 152	149	S-->L
2JEVA	148 - 152	149	S-->Q
2JEVA	148 - 152	149	S-->T
2JEVA	148 - 152	149	S-->V
2JEVA	148 - 152	149	S-->Y
2JEVA	148 - 152	150	S-->A
2JEVA	148 - 152	150	S-->D
2JEVA	148 - 152	150	S-->E
2JEVA	148 - 152	150	S-->F
2JEVA	148 - 152	150	S-->G
2JEVA	148 - 152	150	S-->I
2JEVA	148 - 152	150	S-->K
2JEVA	148 - 152	150	S-->L
2JEVA	148 - 152	150	S-->N
2JEVA	148 - 152	150	S-->R
2JEVA	148 - 152	150	S-->V
2JEVA	148 - 152	150	S-->Y
2JEVA	148 - 152	151	E-->F
2JEVA	148 - 152	151	E-->I
2JEVA	148 - 152	151	E-->N
2JEVA	148 - 152	151	E-->P
2JEVA	148 - 152	151	E-->T
2JEVA	148 - 152	151	E-->V
2JEVA	148 - 152	151	E-->Y
2JEVA	148 - 152	152	E-->A

2JEVA	148	- 152	152	E-->D
2JEVA	148	- 152	152	E-->I
2JEVA	148	- 152	152	E-->K
2JEVA	148	- 152	152	E-->L
2JEVA	148	- 152	152	E-->M
2JEVA	148	- 152	152	E-->P
2JEVA	148	- 152	152	E-->R
2JEVA	148	- 152	152	E-->T
1WSUC	554	- 559	557	L-->Q
1NB8A	319	- 324	319	T-->P
1NB8A	319	- 324	321	P-->L
1NB8A	319	- 324	323	L-->G
1NB8A	319	- 324	324	F-->T
2BDQA	72	- 77	72	D-->V
2BDQA	72	- 77	73	L-->R
2BDQA	72	- 77	73	L-->S
2BDQA	72	- 77	74	E-->F
2CZ3A	77	- 82	77	E-->G
2CZ3A	77	- 82	81	E-->G
2CZ3A	116	- 120	116	L-->C
2CZ3A	116	- 120	116	L-->G
2CZ3A	116	- 120	116	L-->I
2CZ3A	116	- 120	116	L-->K
2CZ3A	116	- 120	116	L-->P
2CZ3A	116	- 120	116	L-->Q
2CZ3A	116	- 120	116	L-->R
2CZ3A	116	- 120	116	L-->S

2CZ3A	116 - 120	116	L-->W
2CZ3A	116 - 120	116	L-->Y
2CZ3A	116 - 120	117	S-->A
2CZ3A	116 - 120	117	S-->C
2CZ3A	116 - 120	117	S-->D
2CZ3A	116 - 120	117	S-->E
2CZ3A	116 - 120	117	S-->G
2CZ3A	116 - 120	117	S-->H
2CZ3A	116 - 120	117	S-->K
2CZ3A	116 - 120	117	S-->P
2CZ3A	116 - 120	117	S-->T
2CZ3A	116 - 120	117	S-->Y
2CZ3A	116 - 120	118	V-->A
2CZ3A	116 - 120	118	V-->D
2CZ3A	116 - 120	118	V-->E
2CZ3A	116 - 120	118	V-->F
2CZ3A	116 - 120	118	V-->H
2CZ3A	116 - 120	118	V-->K
2CZ3A	116 - 120	118	V-->L
2CZ3A	116 - 120	118	V-->N
2CZ3A	116 - 120	118	V-->P
2CZ3A	116 - 120	118	V-->Q
2CZ3A	116 - 120	118	V-->R
2CZ3A	116 - 120	118	V-->S
2CZ3A	116 - 120	118	V-->T
2CZ3A	116 - 120	119	L-->E
2CZ3A	116 - 120	119	L-->G

2CZ3A	116	- 120	119	L-->I
2CZ3A	116	- 120	119	L-->M
2CZ3A	116	- 120	119	L-->Q
2CZ3A	116	- 120	119	L-->R
2CZ3A	116	- 120	119	L-->S
2CZ3A	116	- 120	119	L-->T
2CZ3A	116	- 120	119	L-->V
2CZ3A	116	- 120	119	L-->W
2CZ3A	116	- 120	120	K-->A
2CZ3A	116	- 120	120	K-->D
2CZ3A	116	- 120	120	K-->E
2CZ3A	116	- 120	120	K-->G
2CZ3A	116	- 120	120	K-->H
2CZ3A	116	- 120	120	K-->L
2CZ3A	116	- 120	120	K-->P
2CZ3A	116	- 120	120	K-->S
2CZ3A	116	- 120	120	K-->T
2CZ3A	116	- 120	120	K-->V
2CZ3A	116	- 120	120	K-->Y
1HMWA	126	- 130	127	W-->F
1HMWA	126	- 130	127	W-->L
1HMWA	126	- 130	127	W-->Q
1HMWA	126	- 130	128	H-->E
1HMWA	126	- 130	129	N-->P
1HMWA	126	- 130	130	E-->N
1HMWA	287	- 291	287	G-->A
1HMWA	287	- 291	287	G-->D

1HMWA	287 - 291	287	G-->E
1HMWA	287 - 291	287	G-->F
1HMWA	287 - 291	287	G-->I
1HMWA	287 - 291	287	G-->K
1HMWA	287 - 291	287	G-->L
1HMWA	287 - 291	287	G-->N
1HMWA	287 - 291	287	G-->P
1HMWA	287 - 291	287	G-->Q
1HMWA	287 - 291	287	G-->R
1HMWA	287 - 291	287	G-->T
1HMWA	287 - 291	287	G-->Y
1HMWA	287 - 291	288	R-->A
1HMWA	287 - 291	288	R-->C
1HMWA	287 - 291	288	R-->D
1HMWA	287 - 291	288	R-->E
1HMWA	287 - 291	288	R-->F
1HMWA	287 - 291	288	R-->G
1HMWA	287 - 291	288	R-->H
1HMWA	287 - 291	288	R-->I
1HMWA	287 - 291	288	R-->K
1HMWA	287 - 291	288	R-->L
1HMWA	287 - 291	288	R-->M
1HMWA	287 - 291	288	R-->P
1HMWA	287 - 291	288	R-->Q
1HMWA	287 - 291	288	R-->S
1HMWA	287 - 291	288	R-->T
1HMWA	287 - 291	288	R-->V

1HMWA	287 - 291	288	R-->W
1HMWA	287 - 291	288	R-->Y
1HMWA	287 - 291	289	G-->A
1HMWA	287 - 291	289	G-->C
1HMWA	287 - 291	289	G-->D
1HMWA	287 - 291	289	G-->H
1HMWA	287 - 291	289	G-->I
1HMWA	287 - 291	289	G-->K
1HMWA	287 - 291	289	G-->L
1HMWA	287 - 291	289	G-->P
1HMWA	287 - 291	289	G-->R
1HMWA	287 - 291	289	G-->S
1HMWA	287 - 291	289	G-->V
1HMWA	287 - 291	289	G-->Y
1HMWA	287 - 291	290	V-->D
1HMWA	287 - 291	290	V-->E
1HMWA	287 - 291	290	V-->F
1HMWA	287 - 291	290	V-->G
1HMWA	287 - 291	290	V-->H
1HMWA	287 - 291	290	V-->K
1HMWA	287 - 291	290	V-->L
1HMWA	287 - 291	290	V-->M
1HMWA	287 - 291	290	V-->N
1HMWA	287 - 291	290	V-->P
1HMWA	287 - 291	290	V-->R
1HMWA	287 - 291	290	V-->S
1HMWA	287 - 291	290	V-->T

1HMWA	287	-	291	290	V-->Y
1HMWA	287	-	291	291	S-->A
1HMWA	287	-	291	291	S-->C
1HMWA	287	-	291	291	S-->E
1HMWA	287	-	291	291	S-->F
1HMWA	287	-	291	291	S-->G
1HMWA	287	-	291	291	S-->I
1HMWA	287	-	291	291	S-->L
1HMWA	287	-	291	291	S-->M
1HMWA	287	-	291	291	S-->N
1HMWA	287	-	291	291	S-->P
1HMWA	287	-	291	291	S-->R
1HMWA	287	-	291	291	S-->T
1HMWA	287	-	291	291	S-->V
1HMWA	589	-	593	589	P-->A
1HMWA	589	-	593	589	P-->D
1HMWA	589	-	593	589	P-->G
1HMWA	589	-	593	589	P-->L
1HMWA	589	-	593	589	P-->Q
1HMWA	589	-	593	589	P-->V
1HMWA	589	-	593	590	E-->F
1HMWA	589	-	593	590	E-->I
1HMWA	589	-	593	590	E-->K
1HMWA	589	-	593	590	E-->L
1HMWA	589	-	593	590	E-->Q
1HMWA	589	-	593	590	E-->T
1HMWA	589	-	593	591	E-->F

1HMWA	589	-	593	591	E-->G
1HMWA	589	-	593	591	E-->K
1HMWA	589	-	593	591	E-->L
1HMWA	589	-	593	591	E-->N
1HMWA	589	-	593	591	E-->P
1HMWA	589	-	593	591	E-->R
1HMWA	589	-	593	591	E-->S
1HMWA	589	-	593	591	E-->Y
1HMWA	589	-	593	592	I-->A
1HMWA	589	-	593	592	I-->F
1HMWA	589	-	593	592	I-->G
1HMWA	589	-	593	592	I-->K
1HMWA	589	-	593	592	I-->L
1HMWA	589	-	593	592	I-->N
1HMWA	589	-	593	592	I-->P
1HMWA	589	-	593	592	I-->S
1HMWA	589	-	593	592	I-->W
1HMWA	589	-	593	593	K-->E
1HMWA	589	-	593	593	K-->I
1HMWA	589	-	593	593	K-->L
1HMWA	589	-	593	593	K-->P
1HMWA	589	-	593	593	K-->Q
1HMWA	589	-	593	593	K-->R
1HMWA	589	-	593	593	K-->V
1HMWA	589	-	593	593	K-->Y
2AEGA	85	-	90	85	I-->T
2AEGA	85	-	90	89	I-->P

2CFAA	41 - 46	41	A-->G
2CFAA	41 - 46	41	A-->H
2CFAA	41 - 46	43	G-->F
2CFAA	41 - 46	43	G-->P
2CFAA	41 - 46	44	L-->G
2CFAA	41 - 46	44	L-->K
2CFAA	41 - 46	45	L-->D
2CFAA	41 - 46	45	L-->E
2CFAA	41 - 46	45	L-->P
2CFAA	41 - 46	46	K-->F
2CFAA	54 - 58	54	W-->A
2CFAA	54 - 58	54	W-->C
2CFAA	54 - 58	54	W-->D
2CFAA	54 - 58	54	W-->G
2CFAA	54 - 58	54	W-->R
2CFAA	54 - 58	54	W-->V
2CFAA	54 - 58	56	I-->N
2CFAA	54 - 58	56	I-->Q
2CFAA	54 - 58	57	F-->A
2CFAA	54 - 58	58	E-->A
2CFAA	70 - 76	76	V-->P
2CFAA	151 - 157	155	R-->N
2A8CA	49 - 53	49	A-->C
2A8CA	49 - 53	49	A-->D
2A8CA	49 - 53	49	A-->E
2A8CA	49 - 53	49	A-->F
2A8CA	49 - 53	49	A-->G

2A8CA	49 - 53	49	A-->I
2A8CA	49 - 53	49	A-->K
2A8CA	49 - 53	49	A-->L
2A8CA	49 - 53	49	A-->N
2A8CA	49 - 53	49	A-->Q
2A8CA	49 - 53	49	A-->R
2A8CA	49 - 53	49	A-->S
2A8CA	49 - 53	49	A-->V
2A8CA	49 - 53	49	A-->Y
2A8CA	49 - 53	50	E-->A
2A8CA	49 - 53	50	E-->G
2A8CA	49 - 53	50	E-->K
2A8CA	49 - 53	50	E-->P
2A8CA	49 - 53	50	E-->S
2A8CA	49 - 53	50	E-->T
2A8CA	49 - 53	50	E-->W
2A8CA	49 - 53	50	E-->Y
2A8CA	49 - 53	51	K-->C
2A8CA	49 - 53	51	K-->D
2A8CA	49 - 53	51	K-->G
2A8CA	49 - 53	51	K-->H
2A8CA	49 - 53	51	K-->I
2A8CA	49 - 53	51	K-->L
2A8CA	49 - 53	51	K-->P
2A8CA	49 - 53	51	K-->Q
2A8CA	49 - 53	51	K-->V
2A8CA	49 - 53	51	K-->W

2A8CA	49 - 53	52	L-->I
2A8CA	49 - 53	52	L-->Q
2A8CA	49 - 53	52	L-->R
2A8CA	49 - 53	52	L-->S
2A8CA	49 - 53	52	L-->T
2A8CA	49 - 53	52	L-->V
2A8CA	49 - 53	53	T-->E
2A8CA	49 - 53	53	T-->F
2A8CA	49 - 53	53	T-->G
2A8CA	49 - 53	53	T-->L
2A8CA	49 - 53	53	T-->P
2A8CA	49 - 53	53	T-->Q
2A8CA	49 - 53	53	T-->R
2A8CA	49 - 53	53	T-->S
2A8CA	49 - 53	53	T-->V
2A8CA	131 - 135	131	G-->A
2A8CA	131 - 135	131	G-->D
2A8CA	131 - 135	131	G-->F
2A8CA	131 - 135	131	G-->I
2A8CA	131 - 135	131	G-->M
2A8CA	131 - 135	131	G-->P
2A8CA	131 - 135	131	G-->R
2A8CA	131 - 135	131	G-->S
2A8CA	131 - 135	131	G-->V
2A8CA	131 - 135	131	G-->Y
2A8CA	131 - 135	132	H-->A
2A8CA	131 - 135	132	H-->E

2A8CA	131 - 135	132	H-->F	
2A8CA	131 - 135	132	H-->I	
2A8CA	131 - 135	132	H-->K	
2A8CA	131 - 135	132	H-->L	
2A8CA	131 - 135	132	H-->M	
2A8CA	131 - 135	132	H-->N	
2A8CA	131 - 135	132	H-->P	
2A8CA	131 - 135	132	H-->Q	
2A8CA	131 - 135	132	H-->R	
2A8CA	131 - 135	132	H-->S	
2A8CA	131 - 135	132	H-->T	
2A8CA	131 - 135	132	H-->V	
2A8CA	131 - 135	132	H-->Y	
2A8CA	131 - 135	133	L-->A	
2A8CA	131 - 135	133	L-->E	
2A8CA	131 - 135	133	L-->G	
2A8CA	131 - 135	133	L-->H	
2A8CA	131 - 135	133	L-->K	
2A8CA	131 - 135	133	L-->P	
2A8CA	131 - 135	133	L-->R	
2A8CA	131 - 135	133	L-->S	
2A8CA	131 - 135	133	L-->T	
2A8CA	131 - 135	133	L-->Y	
2A8CA	131 - 135	134	L-->A	
2A8CA	131 - 135	134	L-->D	
2A8CA	131 - 135	134	L-->F	
2A8CA	131 - 135	134	L-->G	

2A8CA	131 - 135	134	L-->N
2A8CA	131 - 135	134	L-->P
2A8CA	131 - 135	134	L-->V
2A8CA	131 - 135	134	L-->Y
2A8CA	131 - 135	135	G-->A
2A8CA	131 - 135	135	G-->C
2A8CA	131 - 135	135	G-->E
2A8CA	131 - 135	135	G-->F
2A8CA	131 - 135	135	G-->L
2A8CA	131 - 135	135	G-->M
2A8CA	131 - 135	135	G-->R
2A8CA	131 - 135	135	G-->S
2A8CA	131 - 135	135	G-->T
2A8CA	131 - 135	135	G-->V
1ZWJB	324 - 329	324	G-->A
1ZWJB	324 - 329	325	F-->V
1ZWJB	324 - 329	329	T-->S
2F2AC	74 - 78	74	Q-->A
2F2AC	74 - 78	74	Q-->D
2F2AC	74 - 78	74	Q-->G
2F2AC	74 - 78	74	Q-->I
2F2AC	74 - 78	74	Q-->K
2F2AC	74 - 78	74	Q-->L
2F2AC	74 - 78	74	Q-->N
2F2AC	74 - 78	74	Q-->P
2F2AC	74 - 78	74	Q-->R
2F2AC	74 - 78	74	Q-->V

2F2AC	74 - 78	75	E-->H
2F2AC	74 - 78	75	E-->K
2F2AC	74 - 78	75	E-->L
2F2AC	74 - 78	75	E-->P
2F2AC	74 - 78	75	E-->Q
2F2AC	74 - 78	75	E-->R
2F2AC	74 - 78	75	E-->T
2F2AC	74 - 78	75	E-->V
2F2AC	74 - 78	75	E-->Y
2F2AC	74 - 78	76	L-->D
2F2AC	74 - 78	76	L-->E
2F2AC	74 - 78	76	L-->K
2F2AC	74 - 78	76	L-->N
2F2AC	74 - 78	76	L-->S
2F2AC	74 - 78	77	A-->E
2F2AC	74 - 78	77	A-->H
2F2AC	74 - 78	77	A-->I
2F2AC	74 - 78	77	A-->K
2F2AC	74 - 78	77	A-->L
2F2AC	74 - 78	77	A-->N
2F2AC	74 - 78	77	A-->Q
2F2AC	74 - 78	77	A-->R
2F2AC	74 - 78	77	A-->Y
2F2AC	74 - 78	78	L-->A
2F2AC	74 - 78	78	L-->I
2F2AC	74 - 78	78	L-->P
2F2AC	74 - 78	78	L-->V

2H92A	54 - 59	54	F-->G
2H92A	54 - 59	54	F-->N
2H92A	54 - 59	54	F-->T
2H92A	54 - 59	54	F-->W
2J2ZB	54 - 58	54	V-->D
2J2ZB	54 - 58	54	V-->G
2J2ZB	54 - 58	54	V-->P
2J2ZB	54 - 58	54	V-->T
2J2ZB	54 - 58	54	V-->W
2J2ZB	54 - 58	54	V-->Y
2J2ZB	54 - 58	55	R-->A
2J2ZB	54 - 58	55	R-->D
2J2ZB	54 - 58	55	R-->G
2J2ZB	54 - 58	55	R-->I
2J2ZB	54 - 58	55	R-->L
2J2ZB	54 - 58	55	R-->N
2J2ZB	54 - 58	55	R-->P
2J2ZB	54 - 58	55	R-->Q
2J2ZB	54 - 58	55	R-->V
2J2ZB	54 - 58	56	D-->F
2J2ZB	54 - 58	56	D-->G
2J2ZB	54 - 58	56	D-->K
2J2ZB	54 - 58	56	D-->L
2J2ZB	54 - 58	56	D-->P
2J2ZB	54 - 58	56	D-->Q
2J2ZB	54 - 58	56	D-->R
2J2ZB	54 - 58	56	D-->S

2J2ZB	54 - 58	56	D-->V
2J2ZB	54 - 58	57	L-->G
2J2ZB	54 - 58	57	L-->P
2J2ZB	54 - 58	57	L-->T
2J2ZB	54 - 58	57	L-->V
2J2ZB	54 - 58	58	Q-->A
2J2ZB	54 - 58	58	Q-->D
2J2ZB	54 - 58	58	Q-->G
2J2ZB	54 - 58	58	Q-->K
2J2ZB	54 - 58	58	Q-->L
2J2ZB	54 - 58	58	Q-->P
2J2ZB	54 - 58	58	Q-->R
2J2ZB	54 - 58	58	Q-->T
2J2ZB	54 - 58	58	Q-->V
2J2ZB	54 - 58	58	Q-->Y
2J3LB	78 - 83	80	W-->L
2J3LB	78 - 83	82	E-->D
2J3LB	348 - 352	348	L-->D
2J3LB	348 - 352	348	L-->E
2J3LB	348 - 352	348	L-->F
2J3LB	348 - 352	348	L-->G
2J3LB	348 - 352	348	L-->S
2J3LB	348 - 352	349	A-->F
2J3LB	348 - 352	349	A-->G
2J3LB	348 - 352	349	A-->K
2J3LB	348 - 352	349	A-->L
2J3LB	348 - 352	349	A-->T

2J3LB	348 - 352	349	A-->V
2J3LB	348 - 352	349	A-->Y
2J3LB	348 - 352	350	V-->C
2J3LB	348 - 352	350	V-->F
2J3LB	348 - 352	350	V-->G
2J3LB	348 - 352	350	V-->L
2J3LB	348 - 352	350	V-->P
2J3LB	348 - 352	350	V-->Q
2J3LB	348 - 352	350	V-->Y
2J3LB	348 - 352	351	Q-->A
2J3LB	348 - 352	351	Q-->C
2J3LB	348 - 352	351	Q-->F
2J3LB	348 - 352	351	Q-->G
2J3LB	348 - 352	351	Q-->I
2J3LB	348 - 352	351	Q-->K
2J3LB	348 - 352	351	Q-->L
2J3LB	348 - 352	351	Q-->M
2J3LB	348 - 352	351	Q-->N
2J3LB	348 - 352	351	Q-->P
2J3LB	348 - 352	351	Q-->R
2J3LB	348 - 352	351	Q-->S
2J3LB	348 - 352	351	Q-->T
2J3LB	348 - 352	351	Q-->V
2J3LB	348 - 352	351	Q-->W
2J3LB	348 - 352	351	Q-->Y
2J3LB	348 - 352	352	D-->I
2J3LB	348 - 352	352	D-->P

2J3LB	417 - 422	420	D-->F
2J3LB	532 - 536	532	K-->A
2J3LB	532 - 536	532	K-->C
2J3LB	532 - 536	532	K-->E
2J3LB	532 - 536	532	K-->F
2J3LB	532 - 536	532	K-->G
2J3LB	532 - 536	532	K-->N
2J3LB	532 - 536	532	K-->T
2J3LB	532 - 536	532	K-->V
2J3LB	532 - 536	533	K-->D
2J3LB	532 - 536	533	K-->G
2J3LB	532 - 536	533	K-->H
2J3LB	532 - 536	533	K-->I
2J3LB	532 - 536	533	K-->L
2J3LB	532 - 536	533	K-->M
2J3LB	532 - 536	533	K-->R
2J3LB	532 - 536	533	K-->S
2J3LB	532 - 536	533	K-->T
2J3LB	532 - 536	534	A-->D
2J3LB	532 - 536	534	A-->E
2J3LB	532 - 536	534	A-->G
2J3LB	532 - 536	534	A-->I
2J3LB	532 - 536	534	A-->K
2J3LB	532 - 536	534	A-->L
2J3LB	532 - 536	534	A-->N
2J3LB	532 - 536	534	A-->P
2J3LB	532 - 536	534	A-->Q

2J3LB	532	536	534	A-->R
2J3LB	532	536	534	A-->V
2J3LB	532	536	535	V-->A
2J3LB	532	536	535	V-->D
2J3LB	532	536	535	V-->F
2J3LB	532	536	535	V-->G
2J3LB	532	536	535	V-->I
2J3LB	532	536	535	V-->K
2J3LB	532	536	535	V-->L
2J3LB	532	536	535	V-->P
2J3LB	532	536	535	V-->Y
2J3LB	532	536	536	D-->A
2J3LB	532	536	536	D-->E
2J3LB	532	536	536	D-->I
2J3LB	532	536	536	D-->K
2J3LB	532	536	536	D-->L
2J3LB	532	536	536	D-->S
2J3LB	532	536	536	D-->T
2J3LB	532	536	536	D-->V
1KR2F	201	205	201	D-->E
1KR2F	201	205	201	D-->F
1KR2F	201	205	201	D-->P
1KR2F	201	205	202	V-->G
1KR2F	201	205	202	V-->L
1KR2F	201	205	202	V-->Q
1KR2F	201	205	203	L-->V
1KR2F	201	205	204	W-->A

1KR2F	201 - 205	204	W-->F
1KR2F	201 - 205	204	W-->I
1KR2F	201 - 205	204	W-->P
1KR2F	201 - 205	204	W-->S
1KR2F	201 - 205	205	K-->G
1KR2F	201 - 205	205	K-->L
1KR2F	201 - 205	205	K-->N
1KR2F	201 - 205	205	K-->P
1KR2F	201 - 205	205	K-->V
1L1SA	45 - 49	45	G-->D
1L1SA	45 - 49	45	G-->H
1L1SA	45 - 49	45	G-->L
1L1SA	45 - 49	45	G-->N
1L1SA	45 - 49	45	G-->P
1L1SA	45 - 49	45	G-->Q
1L1SA	45 - 49	45	G-->R
1L1SA	45 - 49	45	G-->S
1L1SA	45 - 49	45	G-->T
1L1SA	45 - 49	45	G-->V
1L1SA	45 - 49	46	V-->A
1L1SA	45 - 49	46	V-->D
1L1SA	45 - 49	46	V-->F
1L1SA	45 - 49	46	V-->G
1L1SA	45 - 49	46	V-->H
1L1SA	45 - 49	46	V-->I
1L1SA	45 - 49	46	V-->K
1L1SA	45 - 49	46	V-->L

1L1SA	45	49	46	V-->M
1L1SA	45	49	46	V-->N
1L1SA	45	49	46	V-->Q
1L1SA	45	49	46	V-->R
1L1SA	45	49	46	V-->S
1L1SA	45	49	46	V-->T
1L1SA	45	49	46	V-->W
1L1SA	45	49	47	N-->A
1L1SA	45	49	47	N-->C
1L1SA	45	49	47	N-->D
1L1SA	45	49	47	N-->E
1L1SA	45	49	47	N-->F
1L1SA	45	49	47	N-->G
1L1SA	45	49	47	N-->I
1L1SA	45	49	47	N-->K
1L1SA	45	49	47	N-->L
1L1SA	45	49	47	N-->M
1L1SA	45	49	47	N-->P
1L1SA	45	49	47	N-->Q
1L1SA	45	49	47	N-->R
1L1SA	45	49	47	N-->S
1L1SA	45	49	47	N-->T
1L1SA	45	49	47	N-->V
1L1SA	45	49	47	N-->Y
1L1SA	45	49	48	V-->A
1L1SA	45	49	48	V-->G
1L1SA	45	49	48	V-->I

1L1SA	45	-	49	48	V-->L
1L1SA	45	-	49	48	V-->N
1L1SA	45	-	49	48	V-->P
1L1SA	45	-	49	48	V-->Q
1L1SA	45	-	49	48	V-->Y
1L1SA	45	-	49	49	L-->A
1L1SA	45	-	49	49	L-->E
1L1SA	45	-	49	49	L-->F
1L1SA	45	-	49	49	L-->G
1L1SA	45	-	49	49	L-->H
1L1SA	45	-	49	49	L-->I
1L1SA	45	-	49	49	L-->K
1L1SA	45	-	49	49	L-->M
1L1SA	45	-	49	49	L-->N
1L1SA	45	-	49	49	L-->P
1L1SA	45	-	49	49	L-->Q
1L1SA	45	-	49	49	L-->R
1L1SA	45	-	49	49	L-->S
1L1SA	45	-	49	49	L-->T
1L1SA	45	-	49	49	L-->V
1L1SA	45	-	49	49	L-->Y
1L1SA	73	-	78	74	N-->P
1L1SA	73	-	78	74	N-->W
1L1SA	73	-	78	78	A-->I
2CCGB	78	-	83	79	I-->N
2CCGB	78	-	83	81	A-->G
2ISVB	51	-	56	56	Y-->G

1L6L5	51	- 57	51	P-->I
3CEUA	98	- 102	98	V-->A
3CEUA	98	- 102	98	V-->D
3CEUA	98	- 102	98	V-->G
3CEUA	98	- 102	98	V-->I
3CEUA	98	- 102	98	V-->K
3CEUA	98	- 102	98	V-->M
3CEUA	98	- 102	98	V-->Q
3CEUA	98	- 102	99	E-->D
3CEUA	98	- 102	99	E-->G
3CEUA	98	- 102	99	E-->I
3CEUA	98	- 102	99	E-->K
3CEUA	98	- 102	99	E-->L
3CEUA	98	- 102	99	E-->P
3CEUA	98	- 102	99	E-->R
3CEUA	98	- 102	99	E-->S
3CEUA	98	- 102	99	E-->T
3CEUA	98	- 102	99	E-->V
3CEUA	98	- 102	99	E-->W
3CEUA	98	- 102	100	E-->A
3CEUA	98	- 102	100	E-->I
3CEUA	98	- 102	100	E-->K
3CEUA	98	- 102	100	E-->L
3CEUA	98	- 102	100	E-->M
3CEUA	98	- 102	100	E-->P
3CEUA	98	- 102	100	E-->Q
3CEUA	98	- 102	100	E-->R

3CEUA	98 - 102	100	E-->S
3CEUA	98 - 102	100	E-->V
3CEUA	98 - 102	100	E-->Y
3CEUA	98 - 102	101	V-->D
3CEUA	98 - 102	101	V-->F
3CEUA	98 - 102	101	V-->I
3CEUA	98 - 102	101	V-->L
3CEUA	98 - 102	101	V-->P
3CEUA	98 - 102	101	V-->R
3CEUA	98 - 102	101	V-->S
3CEUA	98 - 102	101	V-->Y
3CEUA	98 - 102	102	K-->A
3CEUA	98 - 102	102	K-->D
3CEUA	98 - 102	102	K-->E
3CEUA	98 - 102	102	K-->F
3CEUA	98 - 102	102	K-->G
3CEUA	98 - 102	102	K-->H
3CEUA	98 - 102	102	K-->I
3CEUA	98 - 102	102	K-->N
3CEUA	98 - 102	102	K-->R
3CEUA	98 - 102	102	K-->S
3CEUA	98 - 102	102	K-->V
3CEUA	158 - 163	158	L-->D
3CEUA	158 - 163	158	L-->G
3CEUA	158 - 163	158	L-->I
3CEUA	158 - 163	159	L-->G
3CEUA	158 - 163	161	I-->E

3CEUA	158 - 163	161	I-->F
3CEUA	158 - 163	162	K-->A
3CEUA	158 - 163	163	D-->A
3CEUA	158 - 163	163	D-->K
2F7YB	97 - 102	97	P-->Q
2F7YB	97 - 102	99	F-->P
2F7YB	97 - 102	99	F-->T
2F7YB	97 - 102	100	G-->L
2F7YB	97 - 102	100	G-->V
2F7YB	97 - 102	102	L-->P
2F7YB	97 - 102	102	L-->S
2F7YB	112 - 116	112	P-->D
2F7YB	112 - 116	112	P-->E
2F7YB	112 - 116	112	P-->G
2F7YB	112 - 116	112	P-->K
2F7YB	112 - 116	112	P-->L
2F7YB	112 - 116	112	P-->N
2F7YB	112 - 116	112	P-->Q
2F7YB	112 - 116	112	P-->T
2F7YB	112 - 116	112	P-->V
2F7YB	112 - 116	113	T-->A
2F7YB	112 - 116	113	T-->F
2F7YB	112 - 116	113	T-->G
2F7YB	112 - 116	113	T-->I
2F7YB	112 - 116	113	T-->L
2F7YB	112 - 116	113	T-->N
2F7YB	112 - 116	113	T-->V

2F7YB	112 - 116	114	A-->C
2F7YB	112 - 116	114	A-->F
2F7YB	112 - 116	114	A-->G
2F7YB	112 - 116	114	A-->H
2F7YB	112 - 116	114	A-->I
2F7YB	112 - 116	114	A-->K
2F7YB	112 - 116	114	A-->L
2F7YB	112 - 116	114	A-->P
2F7YB	112 - 116	114	A-->Q
2F7YB	112 - 116	114	A-->R
2F7YB	112 - 116	114	A-->V
2F7YB	112 - 116	115	I-->A
2F7YB	112 - 116	115	I-->D
2F7YB	112 - 116	115	I-->E
2F7YB	112 - 116	115	I-->F
2F7YB	112 - 116	115	I-->G
2F7YB	112 - 116	115	I-->H
2F7YB	112 - 116	115	I-->K
2F7YB	112 - 116	115	I-->M
2F7YB	112 - 116	115	I-->N
2F7YB	112 - 116	115	I-->P
2F7YB	112 - 116	115	I-->Q
2F7YB	112 - 116	115	I-->R
2F7YB	112 - 116	115	I-->S
2F7YB	112 - 116	115	I-->T
2F7YB	112 - 116	115	I-->V
2F7YB	112 - 116	115	I-->Y

2F7YB	112 - 116	116	L-->E
2F7YB	112 - 116	116	L-->F
2F7YB	112 - 116	116	L-->M
2F7YB	112 - 116	116	L-->R
2F7YB	112 - 116	116	L-->T
2F7YB	112 - 116	116	L-->V
2F7YB	112 - 116	116	L-->Y
1P19D	187 - 193	192	E-->Q
2P73A	50 - 55	54	H-->A
2O7VA	74 - 78	74	R-->G
2O7VA	74 - 78	74	R-->N
2O7VA	74 - 78	74	R-->T
2O7VA	74 - 78	74	R-->W
2O7VA	74 - 78	75	H-->D
2O7VA	74 - 78	75	H-->G
2O7VA	74 - 78	75	H-->M
2O7VA	74 - 78	75	H-->N
2O7VA	74 - 78	75	H-->T
2O7VA	74 - 78	75	H-->V
2O7VA	74 - 78	75	H-->W
2O7VA	74 - 78	76	A-->P
2O7VA	74 - 78	76	A-->S
2O7VA	74 - 78	76	A-->T
2O7VA	74 - 78	76	A-->V
2O7VA	74 - 78	77	L-->A
2O7VA	74 - 78	77	L-->S
2O7VA	74 - 78	78	Y-->P

2O7VA	74	78	78	Y-->V
2O7VA	211	215	211	G-->D
2O7VA	211	215	211	G-->F
2O7VA	211	215	211	G-->H
2O7VA	211	215	211	G-->R
2O7VA	211	215	211	G-->V
2O7VA	211	215	212	S-->A
2O7VA	211	215	212	S-->C
2O7VA	211	215	212	S-->E
2O7VA	211	215	212	S-->G
2O7VA	211	215	212	S-->H
2O7VA	211	215	212	S-->I
2O7VA	211	215	212	S-->K
2O7VA	211	215	212	S-->L
2O7VA	211	215	212	S-->P
2O7VA	211	215	212	S-->R
2O7VA	211	215	212	S-->T
2O7VA	211	215	212	S-->V
2O7VA	211	215	212	S-->Y
2O7VA	211	215	213	E-->C
2O7VA	211	215	213	E-->D
2O7VA	211	215	213	E-->F
2O7VA	211	215	213	E-->G
2O7VA	211	215	213	E-->K
2O7VA	211	215	213	E-->L
2O7VA	211	215	213	E-->N
2O7VA	211	215	213	E-->P

2O7VA	211 - 215	213	E-->Q
2O7VA	211 - 215	213	E-->S
2O7VA	211 - 215	213	E-->V
2O7VA	211 - 215	214	L-->A
2O7VA	211 - 215	214	L-->F
2O7VA	211 - 215	214	L-->G
2O7VA	211 - 215	214	L-->I
2O7VA	211 - 215	214	L-->K
2O7VA	211 - 215	214	L-->P
2O7VA	211 - 215	214	L-->Q
2O7VA	211 - 215	214	L-->R
2O7VA	211 - 215	214	L-->T
2O7VA	211 - 215	214	L-->V
2O7VA	211 - 215	214	L-->Y
2O7VA	211 - 215	215	R-->C
2O7VA	211 - 215	215	R-->D
2O7VA	211 - 215	215	R-->E
2O7VA	211 - 215	215	R-->G
2O7VA	211 - 215	215	R-->H
2O7VA	211 - 215	215	R-->I
2O7VA	211 - 215	215	R-->K
2O7VA	211 - 215	215	R-->M
2O7VA	211 - 215	215	R-->N
2O7VA	211 - 215	215	R-->P
2O7VA	211 - 215	215	R-->S
2O7VA	211 - 215	215	R-->T
2O7VA	211 - 215	215	R-->V

2O7VA	211 - 215	215	R-->W
2O7VA	211 - 215	215	R-->Y
1T9ZA	250 - 254	250	V-->A
1T9ZA	250 - 254	250	V-->C
1T9ZA	250 - 254	250	V-->K
1T9ZA	250 - 254	250	V-->R
1T9ZA	250 - 254	250	V-->S
1T9ZA	250 - 254	250	V-->T
1T9ZA	250 - 254	250	V-->W
1T9ZA	250 - 254	250	V-->Y
1T9ZA	250 - 254	251	Y-->A
1T9ZA	250 - 254	251	Y-->D
1T9ZA	250 - 254	251	Y-->H
1T9ZA	250 - 254	251	Y-->I
1T9ZA	250 - 254	251	Y-->K
1T9ZA	250 - 254	251	Y-->M
1T9ZA	250 - 254	251	Y-->Q
1T9ZA	250 - 254	251	Y-->S
1T9ZA	250 - 254	251	Y-->T
1T9ZA	250 - 254	251	Y-->V
1T9ZA	250 - 254	252	S-->E
1T9ZA	250 - 254	252	S-->G
1T9ZA	250 - 254	252	S-->K
1T9ZA	250 - 254	252	S-->Q
1T9ZA	250 - 254	252	S-->R
1T9ZA	250 - 254	252	S-->T
1T9ZA	250 - 254	252	S-->V

1T9ZA	250 - 254	253	V-->F	
1T9ZA	250 - 254	253	V-->G	
1T9ZA	250 - 254	253	V-->H	
1T9ZA	250 - 254	253	V-->K	
1T9ZA	250 - 254	253	V-->N	
1T9ZA	250 - 254	253	V-->P	
1T9ZA	250 - 254	253	V-->Q	
1T9ZA	250 - 254	253	V-->T	
1T9ZA	250 - 254	254	L-->D	
1T9ZA	250 - 254	254	L-->P	
1T9ZA	250 - 254	254	L-->S	
1T9ZA	250 - 254	254	L-->T	
1T9ZA	250 - 254	254	L-->V	
1T9ZA	250 - 254	254	L-->W	
2PW6A	149 - 154	151	W-->V	
2PW6A	149 - 154	152	H-->P	
2ACAA	22 - 27	22	H-->D	
2ACAA	22 - 27	25	F-->G	
2ACAA	93 - 98	93	L-->C	
2ACAA	93 - 98	94	D-->Q	
2ACAA	93 - 98	96	A-->D	
2ACAA	93 - 98	97	Q-->A	
2ACAA	93 - 98	97	Q-->M	
1ZCWA	132 - 136	132	V-->A	
1ZCWA	132 - 136	132	V-->E	
1ZCWA	132 - 136	132	V-->G	
1ZCWA	132 - 136	132	V-->H	

1ZCWA	132 - 136	132	V-->L
1ZCWA	132 - 136	132	V-->N
1ZCWA	132 - 136	132	V-->R
1ZCWA	132 - 136	132	V-->Y
1ZCWA	132 - 136	133	A-->G
1ZCWA	132 - 136	133	A-->H
1ZCWA	132 - 136	133	A-->L
1ZCWA	132 - 136	133	A-->Q
1ZCWA	132 - 136	133	A-->R
1ZCWA	132 - 136	133	A-->V
1ZCWA	132 - 136	133	A-->Y
1ZCWA	132 - 136	134	E-->A
1ZCWA	132 - 136	134	E-->C
1ZCWA	132 - 136	134	E-->D
1ZCWA	132 - 136	134	E-->F
1ZCWA	132 - 136	134	E-->G
1ZCWA	132 - 136	134	E-->K
1ZCWA	132 - 136	134	E-->L
1ZCWA	132 - 136	134	E-->M
1ZCWA	132 - 136	134	E-->N
1ZCWA	132 - 136	134	E-->P
1ZCWA	132 - 136	134	E-->R
1ZCWA	132 - 136	134	E-->S
1ZCWA	132 - 136	134	E-->T
1ZCWA	132 - 136	134	E-->V
1ZCWA	132 - 136	134	E-->W
1ZCWA	132 - 136	134	E-->Y

1ZCWA	132	-	136	135	L-->D
1ZCWA	132	-	136	135	L-->F
1ZCWA	132	-	136	135	L-->G
1ZCWA	132	-	136	135	L-->I
1ZCWA	132	-	136	135	L-->P
1ZCWA	132	-	136	135	L-->T
1ZCWA	132	-	136	135	L-->V
1ZCWA	132	-	136	135	L-->Y
1ZCWA	132	-	136	136	S-->A
1ZCWA	132	-	136	136	S-->E
1ZCWA	132	-	136	136	S-->G
1ZCWA	132	-	136	136	S-->N
1ZCWA	132	-	136	136	S-->P
1ZCWA	132	-	136	136	S-->Q
1ZCWA	132	-	136	136	S-->V
1ZCWA	143	-	147	143	P-->A
1ZCWA	143	-	147	143	P-->F
1ZCWA	143	-	147	143	P-->G
1ZCWA	143	-	147	143	P-->I
1ZCWA	143	-	147	143	P-->K
1ZCWA	143	-	147	143	P-->L
1ZCWA	143	-	147	143	P-->N
1ZCWA	143	-	147	143	P-->R
1ZCWA	143	-	147	143	P-->S
1ZCWA	143	-	147	143	P-->V
1ZCWA	143	-	147	143	P-->Y
1ZCWA	143	-	147	144	A-->C

1ZCWA	143 - 147	144	A-->D
1ZCWA	143 - 147	144	A-->E
1ZCWA	143 - 147	144	A-->F
1ZCWA	143 - 147	144	A-->G
1ZCWA	143 - 147	144	A-->I
1ZCWA	143 - 147	144	A-->K
1ZCWA	143 - 147	144	A-->L
1ZCWA	143 - 147	144	A-->N
1ZCWA	143 - 147	144	A-->Q
1ZCWA	143 - 147	144	A-->R
1ZCWA	143 - 147	144	A-->T
1ZCWA	143 - 147	144	A-->V
1ZCWA	143 - 147	144	A-->Y
1ZCWA	143 - 147	145	V-->D
1ZCWA	143 - 147	145	V-->E
1ZCWA	143 - 147	145	V-->G
1ZCWA	143 - 147	145	V-->L
1ZCWA	143 - 147	145	V-->M
1ZCWA	143 - 147	145	V-->S
1ZCWA	143 - 147	145	V-->T
1ZCWA	143 - 147	146	A-->G
1ZCWA	143 - 147	146	A-->I
1ZCWA	143 - 147	146	A-->L
1ZCWA	143 - 147	146	A-->S
1ZCWA	143 - 147	147	E-->A
1ZCWA	143 - 147	147	E-->C
1ZCWA	143 - 147	147	E-->D

1ZCWA	143 - 147	147	E-->G
1ZCWA	143 - 147	147	E-->I
1ZCWA	143 - 147	147	E-->K
1ZCWA	143 - 147	147	E-->L
1ZCWA	143 - 147	147	E-->M
1ZCWA	143 - 147	147	E-->N
1ZCWA	143 - 147	147	E-->S
1ZCWA	143 - 147	147	E-->V
1ZCWA	149 - 154	150	E-->R
1ZCWA	149 - 154	150	E-->V
1ZCWA	149 - 154	152	F-->Q
1Y8QB	54 - 58	54	V-->A
1Y8QB	54 - 58	54	V-->E
1Y8QB	54 - 58	54	V-->G
1Y8QB	54 - 58	54	V-->I
1Y8QB	54 - 58	54	V-->L
1Y8QB	54 - 58	54	V-->M
1Y8QB	54 - 58	54	V-->P
1Y8QB	54 - 58	54	V-->Q
1Y8QB	54 - 58	54	V-->S
1Y8QB	54 - 58	54	V-->T
1Y8QB	54 - 58	55	S-->A
1Y8QB	54 - 58	55	S-->C
1Y8QB	54 - 58	55	S-->E
1Y8QB	54 - 58	55	S-->F
1Y8QB	54 - 58	55	S-->I
1Y8QB	54 - 58	55	S-->K

1Y8QB	54 - 58	55	S-->L
1Y8QB	54 - 58	55	S-->Q
1Y8QB	54 - 58	55	S-->R
1Y8QB	54 - 58	55	S-->T
1Y8QB	54 - 58	55	S-->V
1Y8QB	54 - 58	55	S-->W
1Y8QB	54 - 58	56	N-->F
1Y8QB	54 - 58	56	N-->G
1Y8QB	54 - 58	56	N-->K
1Y8QB	54 - 58	56	N-->L
1Y8QB	54 - 58	56	N-->M
1Y8QB	54 - 58	56	N-->Q
1Y8QB	54 - 58	56	N-->S
1Y8QB	54 - 58	56	N-->T
1Y8QB	54 - 58	56	N-->V
1Y8QB	54 - 58	57	L-->E
1Y8QB	54 - 58	57	L-->F
1Y8QB	54 - 58	57	L-->Q
1Y8QB	54 - 58	57	L-->S
1Y8QB	54 - 58	57	L-->V
1Y8QB	54 - 58	57	L-->W
1Y8QB	54 - 58	57	L-->Y
1Y8QB	54 - 58	58	N-->A
1Y8QB	54 - 58	58	N-->C
1Y8QB	54 - 58	58	N-->E
1Y8QB	54 - 58	58	N-->F
1Y8QB	54 - 58	58	N-->G

1Y8QB	54	58	58	N-->I
1Y8QB	54	58	58	N-->K
1Y8QB	54	58	58	N-->M
1Y8QB	54	58	58	N-->P
1Y8QB	54	58	58	N-->R
1Y8QB	54	58	58	N-->T
1Y8QB	54	58	58	N-->V
1Y8QB	241	246	242	E-->P
1Y8QB	241	246	243	W-->G
1Y8QB	284	289	285	A-->C
1Y8QB	461	466	461	V-->R
1Y8QB	461	466	463	T-->D
1Y8QB	461	466	463	T-->K
1Y8QB	461	466	463	T-->Q
1Y8QB	461	466	465	Q-->E
1Y8QB	461	466	465	Q-->S
1Y8QB	461	466	465	Q-->T
1Y8QB	461	466	466	D-->A
1Y8QB	461	466	466	D-->V
2J58A	196	200	196	D-->C
2J58A	196	200	196	D-->F
2J58A	196	200	196	D-->G
2J58A	196	200	196	D-->I
2J58A	196	200	196	D-->L
2J58A	196	200	196	D-->N
2J58A	196	200	196	D-->P
2J58A	196	200	196	D-->V

2J58A	196 - 200	197	A-->K
2J58A	196 - 200	197	A-->L
2J58A	196 - 200	197	A-->N
2J58A	196 - 200	197	A-->P
2J58A	196 - 200	197	A-->Q
2J58A	196 - 200	197	A-->R
2J58A	196 - 200	197	A-->Y
2J58A	196 - 200	198	I-->D
2J58A	196 - 200	198	I-->F
2J58A	196 - 200	198	I-->G
2J58A	196 - 200	198	I-->K
2J58A	196 - 200	198	I-->P
2J58A	196 - 200	198	I-->R
2J58A	196 - 200	198	I-->W
2J58A	196 - 200	199	N-->A
2J58A	196 - 200	199	N-->E
2J58A	196 - 200	199	N-->H
2J58A	196 - 200	199	N-->I
2J58A	196 - 200	199	N-->L
2J58A	196 - 200	199	N-->M
2J58A	196 - 200	199	N-->P
2J58A	196 - 200	199	N-->Q
2J58A	196 - 200	199	N-->R
2J58A	196 - 200	199	N-->S
2J58A	196 - 200	199	N-->V
2J58A	196 - 200	200	A-->F
2J58A	196 - 200	200	A-->G

2J58A	196 - 200	200	A-->N
2J58A	196 - 200	200	A-->R
2J58A	196 - 200	200	A-->T
1ZCOA	120 - 126	124	K-->N
1HL9A	22 - 27	27	A-->R
1HL9A	188 - 192	188	P-->A
1HL9A	188 - 192	188	P-->D
1HL9A	188 - 192	188	P-->E
1HL9A	188 - 192	188	P-->F
1HL9A	188 - 192	188	P-->G
1HL9A	188 - 192	188	P-->H
1HL9A	188 - 192	188	P-->K
1HL9A	188 - 192	188	P-->L
1HL9A	188 - 192	188	P-->N
1HL9A	188 - 192	188	P-->R
1HL9A	188 - 192	188	P-->T
1HL9A	188 - 192	188	P-->V
1HL9A	188 - 192	188	P-->Y
1HL9A	188 - 192	189	E-->A
1HL9A	188 - 192	189	E-->F
1HL9A	188 - 192	189	E-->G
1HL9A	188 - 192	189	E-->H
1HL9A	188 - 192	189	E-->I
1HL9A	188 - 192	189	E-->L
1HL9A	188 - 192	189	E-->P
1HL9A	188 - 192	189	E-->R
1HL9A	188 - 192	189	E-->S

1HL9A	188 - 192	189	E-->V
1HL9A	188 - 192	189	E-->Y
1HL9A	188 - 192	190	D-->A
1HL9A	188 - 192	190	D-->E
1HL9A	188 - 192	190	D-->F
1HL9A	188 - 192	190	D-->G
1HL9A	188 - 192	190	D-->H
1HL9A	188 - 192	190	D-->K
1HL9A	188 - 192	190	D-->L
1HL9A	188 - 192	190	D-->P
1HL9A	188 - 192	190	D-->Q
1HL9A	188 - 192	190	D-->R
1HL9A	188 - 192	190	D-->S
1HL9A	188 - 192	190	D-->Y
1HL9A	188 - 192	191	L-->A
1HL9A	188 - 192	191	L-->E
1HL9A	188 - 192	191	L-->G
1HL9A	188 - 192	191	L-->H
1HL9A	188 - 192	191	L-->I
1HL9A	188 - 192	191	L-->N
1HL9A	188 - 192	191	L-->P
1HL9A	188 - 192	191	L-->R
1HL9A	188 - 192	191	L-->S
1HL9A	188 - 192	191	L-->V
1HL9A	188 - 192	192	S-->A
1HL9A	188 - 192	192	S-->D
1HL9A	188 - 192	192	S-->E

1HL9A	188 - 192	192	S-->G
1HL9A	188 - 192	192	S-->L
1HL9A	188 - 192	192	S-->M
1HL9A	188 - 192	192	S-->N
1HL9A	188 - 192	192	S-->P
1HL9A	188 - 192	192	S-->T
1HL9A	188 - 192	192	S-->V
1JKOC	162 - 167	164	Q-->P
1JKOC	162 - 167	167	I-->L
2ODMB	67 - 73	72	Q-->K
3BMAF	51 - 55	51	I-->A
3BMAF	51 - 55	51	I-->E
3BMAF	51 - 55	51	I-->K
3BMAF	51 - 55	51	I-->L
3BMAF	51 - 55	51	I-->P
3BMAF	51 - 55	51	I-->Q
3BMAF	51 - 55	51	I-->R
3BMAF	51 - 55	51	I-->S
3BMAF	51 - 55	51	I-->Y
3BMAF	51 - 55	52	D-->E
3BMAF	51 - 55	52	D-->H
3BMAF	51 - 55	52	D-->I
3BMAF	51 - 55	53	S-->D
3BMAF	51 - 55	53	S-->G
3BMAF	51 - 55	53	S-->I
3BMAF	51 - 55	53	S-->L
3BMAF	51 - 55	53	S-->V

3BMAF	51 -	55	54	F-->I
3BMAF	51 -	55	54	F-->K
3BMAF	51 -	55	54	F-->L
3BMAF	51 -	55	54	F-->R
3BMAF	51 -	55	54	F-->S
3BMAF	51 -	55	54	F-->V
3BMAF	51 -	55	55	K-->G
3BMAF	51 -	55	55	K-->L
3BMAF	51 -	55	55	K-->T
3BMAF	51 -	55	55	K-->V
3BMAF	88 -	94	88	P-->K
3BMAF	88 -	94	88	P-->R
3BMAF	88 -	94	92	A-->T
1GH2A	65 -	70	65	Q-->G
1GH2A	65 -	70	65	Q-->I
1GH2A	65 -	70	65	Q-->S
1GH2A	65 -	70	65	Q-->T
1GH2A	65 -	70	65	Q-->V
1GH2A	65 -	70	66	G-->Y
1GH2A	65 -	70	67	T-->G
1GH2A	65 -	70	67	T-->V
1GH2A	65 -	70	68	A-->V
1GH2A	65 -	70	70	T-->G
1S89A	143 -	148	143	Y-->G
1S89A	143 -	148	145	R-->N
2DG8C	44 -	49	46	M-->T
2P28A	43 -	47	43	R-->D

2P28A	43	- 47	43	R-->L
2P28A	43	- 47	43	R-->P
2P28A	43	- 47	43	R-->S
2P28A	43	- 47	43	R-->T
2P28A	43	- 47	43	R-->V
2P28A	43	- 47	44	P-->G
2P28A	43	- 47	44	P-->I
2P28A	43	- 47	44	P-->L
2P28A	43	- 47	44	P-->Q
2P28A	43	- 47	44	P-->R
2P28A	43	- 47	44	P-->S
2P28A	43	- 47	44	P-->V
2P28A	43	- 47	44	P-->Y
2P28A	43	- 47	45	Q-->D
2P28A	43	- 47	45	Q-->G
2P28A	43	- 47	45	Q-->H
2P28A	43	- 47	45	Q-->I
2P28A	43	- 47	45	Q-->K
2P28A	43	- 47	45	Q-->L
2P28A	43	- 47	45	Q-->S
2P28A	43	- 47	45	Q-->V
2P28A	43	- 47	46	L-->A
2P28A	43	- 47	46	L-->G
2P28A	43	- 47	46	L-->N
2P28A	43	- 47	46	L-->Q
2P28A	43	- 47	46	L-->R
2P28A	43	- 47	46	L-->T

2P28A	43	47	47	L-->A
2P28A	43	47	47	L-->H
2P28A	43	47	47	L-->P
2P28A	43	47	47	L-->S
2P28A	43	47	47	L-->T
2E7DB	31	35	31	V-->G
2E7DB	31	35	31	V-->Q
2E7DB	31	35	32	M-->A
2E7DB	31	35	32	M-->G
2E7DB	31	35	32	M-->I
2E7DB	31	35	32	M-->K
2E7DB	31	35	32	M-->L
2E7DB	31	35	32	M-->P
2E7DB	31	35	32	M-->R
2E7DB	31	35	32	M-->S
2E7DB	31	35	32	M-->W
2E7DB	31	35	33	D-->G
2E7DB	31	35	33	D-->P
2E7DB	31	35	33	D-->T
2E7DB	31	35	33	D-->W
2E7DB	31	35	34	G-->D
2E7DB	31	35	35	F-->A
2E7DB	31	35	35	F-->E
2E7DB	31	35	35	F-->N
2E7DB	31	35	35	F-->R
2E7DB	31	35	35	F-->V
2QPSA	324	328	324	Y-->N

2QPSA	324	328	324	Y-->V
2QPSA	324	328	325	D-->G
2QPSA	324	328	326	H-->D
2QPSA	324	328	326	H-->T
2QPSA	324	328	328	F-->D
2NR9A	44	48	44	E-->A
2NR9A	44	48	44	E-->D
2NR9A	44	48	44	E-->I
2NR9A	44	48	44	E-->Q
2NR9A	44	48	44	E-->R
2NR9A	44	48	44	E-->T
2NR9A	44	48	44	E-->V
2NR9A	44	48	45	E-->L
2NR9A	44	48	45	E-->M
2NR9A	44	48	45	E-->P
2NR9A	44	48	45	E-->S
2NR9A	44	48	45	E-->T
2NR9A	44	48	46	Q-->D
2NR9A	44	48	46	Q-->F
2NR9A	44	48	46	Q-->G
2NR9A	44	48	46	Q-->H
2NR9A	44	48	46	Q-->K
2NR9A	44	48	46	Q-->L
2NR9A	44	48	46	Q-->N
2NR9A	44	48	46	Q-->Y
2NR9A	44	48	47	D-->A
2NR9A	44	48	47	D-->G

2NR9A	44 - 48	47	D-->L
2NR9A	44 - 48	47	D-->N
2NR9A	44 - 48	47	D-->R
2NR9A	44 - 48	47	D-->Y
2NR9A	44 - 48	48	S-->A
2NR9A	44 - 48	48	S-->L
2NR9A	44 - 48	48	S-->R
2IO6A	294 - 298	294	R-->G
2IO6A	294 - 298	294	R-->K
2IO6A	294 - 298	294	R-->P
2IO6A	294 - 298	294	R-->Q
2IO6A	294 - 298	294	R-->T
2IO6A	294 - 298	294	R-->Y
2IO6A	294 - 298	295	Y-->D
2IO6A	294 - 298	295	Y-->F
2IO6A	294 - 298	295	Y-->M
2IO6A	294 - 298	295	Y-->P
2IO6A	294 - 298	295	Y-->S
2IO6A	294 - 298	296	T-->D
2IO6A	294 - 298	296	T-->E
2IO6A	294 - 298	296	T-->L
2IO6A	294 - 298	296	T-->S
2IO6A	294 - 298	297	T-->C
2IO6A	294 - 298	297	T-->P
2IO6A	294 - 298	297	T-->R
2IO6A	294 - 298	298	E-->A
2IO6A	294 - 298	298	E-->F

2IO6A	294	298	298	E-->P
2IO6A	294	298	298	E-->Q
2IO6A	294	298	298	E-->R
2IO6A	294	298	298	E-->S
2IO6A	294	298	298	E-->T
2IO6A	560	564	560	A-->D
2IO6A	560	564	560	A-->G
2IO6A	560	564	560	A-->N
2IO6A	560	564	560	A-->S
2IO6A	560	564	560	A-->V
2IO6A	560	564	561	M-->D
2IO6A	560	564	561	M-->F
2IO6A	560	564	561	M-->G
2IO6A	560	564	561	M-->I
2IO6A	560	564	561	M-->K
2IO6A	560	564	561	M-->L
2IO6A	560	564	561	M-->N
2IO6A	560	564	561	M-->R
2IO6A	560	564	561	M-->S
2IO6A	560	564	561	M-->T
2IO6A	560	564	561	M-->V
2IO6A	560	564	562	A-->V
2IO6A	560	564	562	A-->W
2IO6A	560	564	563	L-->K
2IO6A	560	564	563	L-->R
2IO6A	560	564	563	L-->T
2IO6A	560	564	563	L-->V

2IO6A	560 - 564	564	V-->K
2IO6A	560 - 564	564	V-->P
2IO6A	560 - 564	564	V-->S
2IO6A	560 - 564	564	V-->T
2IO6A	560 - 564	564	V-->Y
1QC7A	238 - 243	238	F-->G
1QC7A	238 - 243	239	E-->Q
1QC7A	238 - 243	240	D-->I
1QC7A	238 - 243	242	L-->K
1EYSH	216 - 221	216	A-->G
1Y9BB	59 - 64	59	A-->R
1Y9BB	59 - 64	60	D-->A
1Y9BB	59 - 64	60	D-->E
1Y9BB	59 - 64	60	D-->S
1Y9BB	59 - 64	60	D-->T
1Y9BB	59 - 64	61	A-->G
1Y9BB	59 - 64	61	A-->L
1Y9BB	59 - 64	61	A-->P
1Y9BB	59 - 64	61	A-->T
1Y9BB	59 - 64	61	A-->V
1Y9BB	59 - 64	62	V-->C
1Y9BB	59 - 64	62	V-->E
1Y9BB	59 - 64	62	V-->L
1Y9BB	59 - 64	63	L-->A
1Y9BB	59 - 64	63	L-->I
1Y9BB	59 - 64	63	L-->T
1Y9BB	59 - 64	63	L-->Y

1Y9BB	59 - 64	64	L-->A
1Y9BB	59 - 64	64	L-->F
1Y9BB	59 - 64	64	L-->H
1Y9BB	59 - 64	64	L-->I
2YX1B	208 - 212	208	P-->A
2YX1B	208 - 212	208	P-->D
2YX1B	208 - 212	208	P-->E
2YX1B	208 - 212	208	P-->F
2YX1B	208 - 212	208	P-->G
2YX1B	208 - 212	208	P-->I
2YX1B	208 - 212	208	P-->R
2YX1B	208 - 212	208	P-->S
2YX1B	208 - 212	208	P-->Y
2YX1B	208 - 212	209	F-->A
2YX1B	208 - 212	209	F-->G
2YX1B	208 - 212	209	F-->K
2YX1B	208 - 212	209	F-->L
2YX1B	208 - 212	209	F-->N
2YX1B	208 - 212	209	F-->P
2YX1B	208 - 212	209	F-->S
2YX1B	208 - 212	209	F-->T
2YX1B	208 - 212	209	F-->V
2YX1B	208 - 212	210	S-->A
2YX1B	208 - 212	210	S-->D
2YX1B	208 - 212	210	S-->R
2YX1B	208 - 212	210	S-->V
2YX1B	208 - 212	211	I-->D

2YX1B	208 - 212	211	I-->E
2YX1B	208 - 212	211	I-->L
2YX1B	208 - 212	211	I-->M
2YX1B	208 - 212	211	I-->P
2YX1B	208 - 212	211	I-->R
2YX1B	208 - 212	211	I-->S
2YX1B	208 - 212	211	I-->V
2YX1B	208 - 212	211	I-->Y
2YX1B	208 - 212	212	A-->E
2YX1B	208 - 212	212	A-->G
2YX1B	208 - 212	212	A-->R
2YX1B	208 - 212	212	A-->T
1NOYA	161 - 165	161	A-->D
1NOYA	161 - 165	161	A-->E
1NOYA	161 - 165	161	A-->F
1NOYA	161 - 165	161	A-->G
1NOYA	161 - 165	161	A-->K
1NOYA	161 - 165	161	A-->L
1NOYA	161 - 165	161	A-->N
1NOYA	161 - 165	161	A-->P
1NOYA	161 - 165	161	A-->R
1NOYA	161 - 165	161	A-->S
1NOYA	161 - 165	161	A-->V
1NOYA	161 - 165	161	A-->Y
1NOYA	161 - 165	162	K-->A
1NOYA	161 - 165	162	K-->D
1NOYA	161 - 165	162	K-->E

1NOYA	161 - 165	162	K-->I
1NOYA	161 - 165	162	K-->L
1NOYA	161 - 165	162	K-->N
1NOYA	161 - 165	162	K-->P
1NOYA	161 - 165	162	K-->R
1NOYA	161 - 165	162	K-->S
1NOYA	161 - 165	162	K-->V
1NOYA	161 - 165	163	L-->A
1NOYA	161 - 165	163	L-->D
1NOYA	161 - 165	163	L-->E
1NOYA	161 - 165	163	L-->F
1NOYA	161 - 165	163	L-->G
1NOYA	161 - 165	163	L-->I
1NOYA	161 - 165	163	L-->K
1NOYA	161 - 165	163	L-->R
1NOYA	161 - 165	163	L-->S
1NOYA	161 - 165	163	L-->V
1NOYA	161 - 165	164	A-->E
1NOYA	161 - 165	164	A-->F
1NOYA	161 - 165	164	A-->G
1NOYA	161 - 165	164	A-->K
1NOYA	161 - 165	164	A-->L
1NOYA	161 - 165	164	A-->P
1NOYA	161 - 165	164	A-->R
1NOYA	161 - 165	164	A-->S
1NOYA	161 - 165	164	A-->T
1NOYA	161 - 165	164	A-->V

1NOYA	161	- 165	165	A-->D
1NOYA	161	- 165	165	A-->F
1NOYA	161	- 165	165	A-->G
1NOYA	161	- 165	165	A-->L
1NOYA	161	- 165	165	A-->N
1NOYA	161	- 165	165	A-->P
1NOYA	161	- 165	165	A-->Q
1NOYA	161	- 165	165	A-->T
1NOYA	352	- 356	352	F-->H
1NOYA	352	- 356	352	F-->I
1NOYA	352	- 356	352	F-->K
1NOYA	352	- 356	352	F-->L
1NOYA	352	- 356	352	F-->N
1NOYA	352	- 356	352	F-->Q
1NOYA	352	- 356	352	F-->S
1NOYA	352	- 356	353	S-->E
1NOYA	352	- 356	353	S-->T
1NOYA	352	- 356	354	G-->C
1NOYA	352	- 356	354	G-->F
1NOYA	352	- 356	354	G-->Q
1NOYA	352	- 356	355	V-->H
1NOYA	352	- 356	355	V-->Q
1NOYA	352	- 356	356	M-->C
1NOYA	352	- 356	356	M-->E
1NOYA	352	- 356	356	M-->G
1NOYA	352	- 356	356	M-->H
1NOYA	352	- 356	356	M-->I

1NOYA	352	- 356	356	M-->K
1NOYA	352	- 356	356	M-->N
1NOYA	352	- 356	356	M-->P
1NOYA	352	- 356	356	M-->T
1PSUB	17	- 21	17	A-->S
1PSUB	17	- 21	18	C-->G
1PSUB	17	- 21	18	C-->H
1PSUB	17	- 21	18	C-->I
1PSUB	17	- 21	18	C-->K
1PSUB	17	- 21	18	C-->L
1PSUB	17	- 21	18	C-->N
1PSUB	17	- 21	18	C-->R
1PSUB	17	- 21	18	C-->T
1PSUB	17	- 21	18	C-->V
1PSUB	17	- 21	19	A-->G
1PSUB	17	- 21	19	A-->T
1PSUB	17	- 21	19	A-->V
1PSUB	17	- 21	20	K-->D
1PSUB	17	- 21	20	K-->G
1PSUB	17	- 21	20	K-->I
1PSUB	17	- 21	20	K-->N
1PSUB	17	- 21	20	K-->S
1PSUB	17	- 21	21	A-->E
1PSUB	17	- 21	21	A-->H
1PSUB	17	- 21	21	A-->S
1PSUB	17	- 21	21	A-->V
1DEVD	686	- 690	686	P-->K

1DEVD	686	690	686	P-->R
1DEVD	686	690	686	P-->S
1DEVD	686	690	686	P-->T
1DEVD	686	690	686	P-->W
1DEVD	686	690	687	L-->P
1DEVD	686	690	687	L-->S
1DEVD	686	690	688	Q-->G
1DEVD	686	690	688	Q-->L
1DEVD	686	690	688	Q-->P
1DEVD	686	690	688	Q-->S
1DEVD	686	690	688	Q-->V
1DEVD	686	690	689	Q-->D
1DEVD	686	690	689	Q-->I
1DEVD	686	690	689	Q-->K
1DEVD	686	690	689	Q-->N
1DEVD	686	690	689	Q-->Y
1DEVD	686	690	690	A-->L
1DEVD	686	690	690	A-->N
1DEVD	686	690	690	A-->Q
1V1JA	81	85	81	A-->N
1V1JA	81	85	81	A-->Y
1V1JA	81	85	82	A-->I
1V1JA	81	85	82	A-->L
1V1JA	81	85	82	A-->S
1V1JA	81	85	82	A-->T
1V1JA	81	85	83	Y-->A
1V1JA	81	85	83	Y-->C

1V1JA	81 - 85	83	Y-->D
1V1JA	81 - 85	83	Y-->E
1V1JA	81 - 85	83	Y-->G
1V1JA	81 - 85	83	Y-->P
1V1JA	81 - 85	83	Y-->Q
1V1JA	81 - 85	83	Y-->S
1V1JA	81 - 85	83	Y-->W
1V1JA	81 - 85	84	S-->E
1V1JA	81 - 85	84	S-->G
1V1JA	81 - 85	84	S-->K
1V1JA	81 - 85	84	S-->L
1V1JA	81 - 85	84	S-->M
1V1JA	81 - 85	84	S-->N
1V1JA	81 - 85	85	H-->A
1V1JA	81 - 85	85	H-->D
1V1JA	81 - 85	85	H-->E
1V1JA	81 - 85	85	H-->K
1V1JA	81 - 85	85	H-->L
1V1JA	81 - 85	85	H-->Y
1ZVPC	56 - 60	56	A-->G
1ZVPC	56 - 60	56	A-->I
1ZVPC	56 - 60	57	E-->I
1ZVPC	56 - 60	57	E-->K
1ZVPC	56 - 60	57	E-->N
1ZVPC	56 - 60	57	E-->Q
1ZVPC	56 - 60	57	E-->Y
1ZVPC	56 - 60	58	K-->A

1ZVPC	56 - 60	58	K-->E
1ZVPC	56 - 60	58	K-->I
1ZVPC	56 - 60	58	K-->T
1ZVPC	56 - 60	59	A-->K
1ZVPC	56 - 60	59	A-->L
1ZVPC	56 - 60	59	A-->N
1ZVPC	56 - 60	59	A-->R
1ZVPC	56 - 60	59	A-->V
1ZVPC	56 - 60	59	A-->W
1ZVPC	56 - 60	60	Q-->D
1ZVPC	56 - 60	60	Q-->F
1ZVPC	56 - 60	60	Q-->G
1ZVPC	56 - 60	60	Q-->I
1ZVPC	56 - 60	60	Q-->K
1ZVPC	56 - 60	60	Q-->L
1ZVPC	56 - 60	60	Q-->N
1ZVPC	56 - 60	60	Q-->P
1ZVPC	56 - 60	60	Q-->S
1ZVPC	56 - 60	60	Q-->V
2AAMC	46 - 50	46	P-->A
2AAMC	46 - 50	46	P-->E
2AAMC	46 - 50	46	P-->F
2AAMC	46 - 50	46	P-->G
2AAMC	46 - 50	46	P-->H
2AAMC	46 - 50	46	P-->I
2AAMC	46 - 50	46	P-->K
2AAMC	46 - 50	46	P-->N

2AAMC	46 - 50	46	P-->Q
2AAMC	46 - 50	46	P-->S
2AAMC	46 - 50	46	P-->T
2AAMC	46 - 50	47	V-->D
2AAMC	46 - 50	47	V-->L
2AAMC	46 - 50	47	V-->M
2AAMC	46 - 50	47	V-->P
2AAMC	46 - 50	47	V-->Q
2AAMC	46 - 50	47	V-->R
2AAMC	46 - 50	48	E-->D
2AAMC	46 - 50	48	E-->F
2AAMC	46 - 50	48	E-->G
2AAMC	46 - 50	48	E-->H
2AAMC	46 - 50	48	E-->I
2AAMC	46 - 50	48	E-->K
2AAMC	46 - 50	48	E-->L
2AAMC	46 - 50	48	E-->N
2AAMC	46 - 50	48	E-->Q
2AAMC	46 - 50	48	E-->S
2AAMC	46 - 50	48	E-->T
2AAMC	46 - 50	48	E-->V
2AAMC	46 - 50	48	E-->Y
2AAMC	46 - 50	49	I-->A
2AAMC	46 - 50	49	I-->E
2AAMC	46 - 50	49	I-->F
2AAMC	46 - 50	49	I-->G
2AAMC	46 - 50	49	I-->H

2AAMC	46	- 50	49	I-->K
2AAMC	46	- 50	49	I-->S
2AAMC	46	- 50	49	I-->T
2AAMC	46	- 50	49	I-->V
2AAMC	46	- 50	50	S-->A
2AAMC	46	- 50	50	S-->C
2AAMC	46	- 50	50	S-->D
2AAMC	46	- 50	50	S-->E
2AAMC	46	- 50	50	S-->G
2AAMC	46	- 50	50	S-->L
2AAMC	46	- 50	50	S-->M
2AAMC	46	- 50	50	S-->N
2AAMC	46	- 50	50	S-->P
2AAMC	46	- 50	50	S-->Q
2AAMC	46	- 50	50	S-->R
2AAMC	46	- 50	50	S-->V
2AAMC	46	- 50	50	S-->W
2AAMC	73	- 78	73	P-->D
2AAMC	73	- 78	75	E-->N
2AAMC	73	- 78	75	E-->R
2AAMC	73	- 78	76	I-->K
2AAMC	73	- 78	77	K-->I
2AAMC	205	- 210	207	I-->G
2AAMC	205	- 210	207	I-->V
2AAMC	205	- 210	209	D-->G
2AAMC	205	- 210	209	D-->S
2AAMC	214	- 218	214	Q-->A

2AAMC	214 - 218	214	Q-->E
2AAMC	214 - 218	214	Q-->G
2AAMC	214 - 218	214	Q-->K
2AAMC	214 - 218	214	Q-->L
2AAMC	214 - 218	214	Q-->P
2AAMC	214 - 218	214	Q-->R
2AAMC	214 - 218	214	Q-->S
2AAMC	214 - 218	214	Q-->T
2AAMC	214 - 218	214	Q-->V
2AAMC	214 - 218	214	Q-->Y
2AAMC	214 - 218	215	L-->Q
2AAMC	214 - 218	215	L-->Y
2AAMC	214 - 218	216	A-->C
2AAMC	214 - 218	216	A-->F
2AAMC	214 - 218	216	A-->G
2AAMC	214 - 218	216	A-->H
2AAMC	214 - 218	216	A-->L
2AAMC	214 - 218	216	A-->S
2AAMC	214 - 218	216	A-->T
2AAMC	214 - 218	217	S-->I
2AAMC	214 - 218	217	S-->K
2AAMC	214 - 218	217	S-->L
2AAMC	214 - 218	217	S-->P
2AAMC	214 - 218	217	S-->Q
2AAMC	214 - 218	217	S-->V
2AAMC	214 - 218	218	T-->F
2AAMC	214 - 218	218	T-->G

2AAMC	214	218	218	T-->H
2AAMC	214	218	218	T-->N
2AAMC	214	218	218	T-->P
2AAMC	214	218	218	T-->V
1VQO2	23	28	24	W-->P
1VQO2	23	28	24	W-->S
1VQO2	23	28	26	M-->S
2OBBA	103	108	103	W-->I
2OBBA	116	120	116	F-->A
2OBBA	116	120	116	F-->E
2OBBA	116	120	116	F-->H
2OBBA	116	120	116	F-->K
2OBBA	116	120	116	F-->L
2OBBA	116	120	116	F-->R
2OBBA	116	120	116	F-->V
2OBBA	116	120	117	A-->D
2OBBA	116	120	117	A-->G
2OBBA	116	120	118	D-->A
2OBBA	116	120	118	D-->K
2OBBA	116	120	118	D-->M
2OBBA	116	120	118	D-->S
2OBBA	116	120	118	D-->V
2OBBA	116	120	119	I-->E
2OBBA	116	120	119	I-->Y
2OBBA	116	120	120	Y-->A
2OBBA	116	120	120	Y-->C
2OBBA	116	120	120	Y-->D

2OBBA	116 - 120	120	Y-->E
2OBBA	116 - 120	120	Y-->G
2OBBA	116 - 120	120	Y-->P
2OBBA	116 - 120	120	Y-->V
1NMPA	98 - 102	98	L-->D
1NMPA	98 - 102	98	L-->F
1NMPA	98 - 102	98	L-->G
1NMPA	98 - 102	98	L-->I
1NMPA	98 - 102	98	L-->K
1NMPA	98 - 102	98	L-->N
1NMPA	98 - 102	98	L-->Q
1NMPA	98 - 102	98	L-->R
1NMPA	98 - 102	98	L-->T
1NMPA	98 - 102	98	L-->Y
1NMPA	98 - 102	99	P-->A
1NMPA	98 - 102	99	P-->C
1NMPA	98 - 102	99	P-->D
1NMPA	98 - 102	99	P-->F
1NMPA	98 - 102	99	P-->G
1NMPA	98 - 102	99	P-->H
1NMPA	98 - 102	99	P-->I
1NMPA	98 - 102	99	P-->K
1NMPA	98 - 102	99	P-->L
1NMPA	98 - 102	99	P-->N
1NMPA	98 - 102	99	P-->Q
1NMPA	98 - 102	99	P-->R
1NMPA	98 - 102	99	P-->S

1NMPA	98	102	99	P-->V
1NMPA	98	102	100	L-->A
1NMPA	98	102	100	L-->D
1NMPA	98	102	100	L-->E
1NMPA	98	102	100	L-->F
1NMPA	98	102	100	L-->G
1NMPA	98	102	100	L-->I
1NMPA	98	102	100	L-->M
1NMPA	98	102	100	L-->P
1NMPA	98	102	100	L-->Q
1NMPA	98	102	100	L-->R
1NMPA	98	102	100	L-->S
1NMPA	98	102	100	L-->V
1NMPA	98	102	100	L-->Y
1NMPA	98	102	101	D-->A
1NMPA	98	102	101	D-->C
1NMPA	98	102	101	D-->E
1NMPA	98	102	101	D-->F
1NMPA	98	102	101	D-->G
1NMPA	98	102	101	D-->H
1NMPA	98	102	101	D-->I
1NMPA	98	102	101	D-->L
1NMPA	98	102	101	D-->M
1NMPA	98	102	101	D-->P
1NMPA	98	102	101	D-->Q
1NMPA	98	102	101	D-->R
1NMPA	98	102	101	D-->S

1NMPA	98 - 102	101	D-->T
1NMPA	98 - 102	101	D-->V
1NMPA	98 - 102	101	D-->W
1NMPA	98 - 102	102	A-->E
1NMPA	98 - 102	102	A-->F
1NMPA	98 - 102	102	A-->I
1NMPA	98 - 102	102	A-->K
1NMPA	98 - 102	102	A-->L
1NMPA	98 - 102	102	A-->M
1NMPA	98 - 102	102	A-->N
1NMPA	98 - 102	102	A-->P
1NMPA	98 - 102	102	A-->Q
1NMPA	98 - 102	102	A-->R
1NMPA	98 - 102	102	A-->S
1NMPA	98 - 102	102	A-->V
1FROA	12 - 16	12	D-->A
1FROA	12 - 16	12	D-->E
1FROA	12 - 16	12	D-->G
1FROA	12 - 16	12	D-->K
1FROA	12 - 16	12	D-->N
1FROA	12 - 16	12	D-->P
1FROA	12 - 16	12	D-->S
1FROA	12 - 16	12	D-->V
1FROA	12 - 16	13	E-->A
1FROA	12 - 16	13	E-->C
1FROA	12 - 16	13	E-->D
1FROA	12 - 16	13	E-->F

1FROA	12 - 16	13	E-->I
1FROA	12 - 16	13	E-->K
1FROA	12 - 16	13	E-->M
1FROA	12 - 16	13	E-->N
1FROA	12 - 16	13	E-->P
1FROA	12 - 16	13	E-->R
1FROA	12 - 16	13	E-->T
1FROA	12 - 16	13	E-->V
1FROA	12 - 16	14	A-->C
1FROA	12 - 16	14	A-->E
1FROA	12 - 16	14	A-->G
1FROA	12 - 16	14	A-->I
1FROA	12 - 16	14	A-->K
1FROA	12 - 16	14	A-->L
1FROA	12 - 16	14	A-->N
1FROA	12 - 16	14	A-->Q
1FROA	12 - 16	14	A-->V
1FROA	12 - 16	14	A-->Y
1FROA	12 - 16	15	A-->D
1FROA	12 - 16	15	A-->E
1FROA	12 - 16	15	A-->F
1FROA	12 - 16	15	A-->G
1FROA	12 - 16	15	A-->I
1FROA	12 - 16	15	A-->L
1FROA	12 - 16	15	A-->N
1FROA	12 - 16	15	A-->P
1FROA	12 - 16	15	A-->Q

1FROA	12	16	15	A-->R
1FROA	12	16	15	A-->T
1FROA	12	16	15	A-->V
1FROA	12	16	15	A-->Y
1FROA	12	16	16	L-->E
1FROA	12	16	16	L-->F
1FROA	12	16	16	L-->H
1FROA	12	16	16	L-->K
1FROA	12	16	16	L-->P
1FROA	12	16	16	L-->R
1FROA	12	16	16	L-->V
1YLNA	29	34	29	S-->E
1YLNA	29	34	29	S-->R
1YLNA	29	34	30	T-->I
1YLNA	29	34	31	D-->G
1YLNA	29	34	31	D-->K
1YLNA	29	34	31	D-->T
1YLNA	29	34	32	A-->Y
1YLNA	29	34	33	L-->G
1YLNA	29	34	34	A-->M
2BL7A	41	45	41	Q-->A
2BL7A	41	45	41	Q-->D
2BL7A	41	45	41	Q-->E
2BL7A	41	45	41	Q-->G
2BL7A	41	45	41	Q-->K
2BL7A	41	45	41	Q-->L
2BL7A	41	45	41	Q-->N

2BL7A	41 -	45	41	Q-->R
2BL7A	41 -	45	41	Q-->W
2BL7A	41 -	45	42	K-->E
2BL7A	41 -	45	42	K-->F
2BL7A	41 -	45	42	K-->G
2BL7A	41 -	45	42	K-->I
2BL7A	41 -	45	42	K-->Q
2BL7A	41 -	45	43	Y-->E
2BL7A	41 -	45	43	Y-->I
2BL7A	41 -	45	43	Y-->L
2BL7A	41 -	45	43	Y-->T
2BL7A	41 -	45	43	Y-->V
2BL7A	41 -	45	44	E-->D
2BL7A	41 -	45	44	E-->F
2BL7A	41 -	45	44	E-->G
2BL7A	41 -	45	44	E-->L
2BL7A	41 -	45	44	E-->R
2BL7A	41 -	45	44	E-->V
2BL7A	41 -	45	45	L-->D
2BL7A	41 -	45	45	L-->K
2BL7A	41 -	45	45	L-->N
1VPQA	75 -	79	75	G-->H
1VPQA	75 -	79	75	G-->M
1VPQA	75 -	79	75	G-->N
1VPQA	75 -	79	75	G-->P
1VPQA	75 -	79	75	G-->Q
1VPQA	75 -	79	75	G-->S

1VPQA	75 - 79	75	G-->V	
1VPQA	75 - 79	76	S-->A	
1VPQA	75 - 79	76	S-->D	
1VPQA	75 - 79	76	S-->E	
1VPQA	75 - 79	76	S-->F	
1VPQA	75 - 79	76	S-->H	
1VPQA	75 - 79	77	V-->C	
1VPQA	75 - 79	77	V-->I	
1VPQA	75 - 79	77	V-->L	
1VPQA	75 - 79	77	V-->P	
1VPQA	75 - 79	77	V-->Q	
1VPQA	75 - 79	77	V-->R	
1VPQA	75 - 79	77	V-->W	
1VPQA	75 - 79	78	T-->D	
1VPQA	75 - 79	78	T-->H	
1VPQA	75 - 79	78	T-->L	
1VPQA	75 - 79	78	T-->P	
1VPQA	75 - 79	78	T-->R	
1VPQA	75 - 79	78	T-->V	
1VPQA	75 - 79	79	H-->A	
1VPQA	75 - 79	79	H-->C	
1VPQA	75 - 79	79	H-->D	
1VPQA	75 - 79	79	H-->E	
1VPQA	75 - 79	79	H-->F	
1VPQA	75 - 79	79	H-->G	
1VPQA	75 - 79	79	H-->I	
1VPQA	75 - 79	79	H-->L	

1VPQA	75	79	79	H-->M
1VPQA	75	79	79	H-->N
1VPQA	75	79	79	H-->P
1VPQA	75	79	79	H-->R
1VPQA	75	79	79	H-->S
1VPQA	75	79	79	H-->T
1VPQA	75	79	79	H-->V
1VPQA	75	79	79	H-->W
1VPQA	75	79	79	H-->Y
2AB5A	338	343	340	H-->K
2AB5A	345	351	351	K-->I
2AB5A	392	396	392	T-->C
2AB5A	392	396	392	T-->D
2AB5A	392	396	392	T-->G
2AB5A	392	396	392	T-->L
2AB5A	392	396	392	T-->N
2AB5A	392	396	392	T-->V
2AB5A	392	396	393	E-->A
2AB5A	392	396	393	E-->F
2AB5A	392	396	393	E-->H
2AB5A	392	396	393	E-->I
2AB5A	392	396	393	E-->K
2AB5A	392	396	393	E-->P
2AB5A	392	396	393	E-->Q
2AB5A	392	396	393	E-->S
2AB5A	392	396	393	E-->V
2AB5A	392	396	393	E-->Y

2AB5A	392 - 396	394	D-->E
2AB5A	392 - 396	394	D-->F
2AB5A	392 - 396	394	D-->H
2AB5A	392 - 396	394	D-->L
2AB5A	392 - 396	394	D-->N
2AB5A	392 - 396	394	D-->P
2AB5A	392 - 396	394	D-->R
2AB5A	392 - 396	394	D-->S
2AB5A	392 - 396	394	D-->T
2AB5A	392 - 396	394	D-->Y
2AB5A	392 - 396	395	I-->D
2AB5A	392 - 396	395	I-->E
2AB5A	392 - 396	395	I-->P
2AB5A	392 - 396	395	I-->R
2AB5A	392 - 396	396	L-->N
2AB5A	392 - 396	396	L-->P
2AB5A	392 - 396	396	L-->Q
2AB5A	442 - 447	444	I-->N
2AB5A	442 - 447	445	K-->P
2AB5A	442 - 447	445	K-->Y
2QKUA	617 - 622	621	S-->Y
2QKUA	617 - 622	622	K-->Y
1N7GD	119 - 124	119	V-->G
1N7GD	119 - 124	119	V-->L
1N7GD	119 - 124	119	V-->Q
1N7GD	119 - 124	124	E-->D
1N7GD	119 - 124	124	E-->S

1N7GD	326 - 331	326	S-->G
1N7GD	326 - 331	326	S-->L
1N7GD	326 - 331	330	E-->D
1N7GD	326 - 331	330	E-->I
1N7GD	326 - 331	330	E-->R
2PQFA	601 - 605	601	A-->N
2PQFA	601 - 605	601	A-->Y
2PQFA	601 - 605	602	A-->I
2PQFA	601 - 605	602	A-->L
2PQFA	601 - 605	602	A-->S
2PQFA	601 - 605	602	A-->T
2PQFA	601 - 605	603	Y-->A
2PQFA	601 - 605	603	Y-->C
2PQFA	601 - 605	603	Y-->D
2PQFA	601 - 605	603	Y-->E
2PQFA	601 - 605	603	Y-->G
2PQFA	601 - 605	603	Y-->P
2PQFA	601 - 605	603	Y-->Q
2PQFA	601 - 605	603	Y-->S
2PQFA	601 - 605	603	Y-->W
2PQFA	601 - 605	604	S-->E
2PQFA	601 - 605	604	S-->G
2PQFA	601 - 605	604	S-->K
2PQFA	601 - 605	604	S-->L
2PQFA	601 - 605	604	S-->M
2PQFA	601 - 605	604	S-->N
2PQFA	601 - 605	605	H-->A

2PQFA	601	- 605	605	H-->D
2PQFA	601	- 605	605	H-->E
2PQFA	601	- 605	605	H-->K
2PQFA	601	- 605	605	H-->L
2PQFA	601	- 605	605	H-->Y
1DKUA	137	- 142	141	G-->R
1DKUA	260	- 265	261	V-->N
1DKUA	260	- 265	262	E-->F
1DKUA	260	- 265	262	E-->K
1DKUA	260	- 265	265	N-->D
1DKUA	260	- 265	265	N-->E
2RJZB	68	- 74	74	T-->G
3CI0K	98	- 102	98	L-->A
3CI0K	98	- 102	98	L-->D
3CI0K	98	- 102	98	L-->E
3CI0K	98	- 102	98	L-->I
3CI0K	98	- 102	98	L-->K
3CI0K	98	- 102	98	L-->P
3CI0K	98	- 102	98	L-->R
3CI0K	98	- 102	98	L-->T
3CI0K	98	- 102	98	L-->V
3CI0K	98	- 102	98	L-->Y
3CI0K	98	- 102	99	N-->A
3CI0K	98	- 102	99	N-->D
3CI0K	98	- 102	99	N-->F
3CI0K	98	- 102	99	N-->H
3CI0K	98	- 102	99	N-->I

3CI0K	98 - 102	99	N-->L
3CI0K	98 - 102	99	N-->P
3CI0K	98 - 102	99	N-->S
3CI0K	98 - 102	99	N-->V
3CI0K	98 - 102	100	A-->D
3CI0K	98 - 102	100	A-->F
3CI0K	98 - 102	100	A-->G
3CI0K	98 - 102	100	A-->I
3CI0K	98 - 102	100	A-->K
3CI0K	98 - 102	100	A-->N
3CI0K	98 - 102	100	A-->P
3CI0K	98 - 102	100	A-->R
3CI0K	98 - 102	100	A-->S
3CI0K	98 - 102	101	L-->D
3CI0K	98 - 102	101	L-->G
3CI0K	98 - 102	101	L-->P
3CI0K	98 - 102	101	L-->R
3CI0K	98 - 102	101	L-->S
3CI0K	98 - 102	101	L-->T
3CI0K	98 - 102	101	L-->V
3CI0K	98 - 102	102	A-->C
3CI0K	98 - 102	102	A-->E
3CI0K	98 - 102	102	A-->K
3CI0K	98 - 102	102	A-->L
3CI0K	98 - 102	102	A-->P
3CI0K	98 - 102	102	A-->S
3CI0K	98 - 102	102	A-->T

3CI0K	98 - 102	102	A-->V	
3CI0K	155 - 159	155	D-->E	
3CI0K	155 - 159	155	D-->H	
3CI0K	155 - 159	155	D-->L	
3CI0K	155 - 159	155	D-->Q	
3CI0K	155 - 159	155	D-->T	
3CI0K	155 - 159	155	D-->Y	
3CI0K	155 - 159	156	S-->A	
3CI0K	155 - 159	156	S-->E	
3CI0K	155 - 159	156	S-->F	
3CI0K	155 - 159	156	S-->G	
3CI0K	155 - 159	156	S-->H	
3CI0K	155 - 159	156	S-->K	
3CI0K	155 - 159	156	S-->M	
3CI0K	155 - 159	156	S-->P	
3CI0K	155 - 159	157	E-->A	
3CI0K	155 - 159	157	E-->I	
3CI0K	155 - 159	157	E-->S	
3CI0K	155 - 159	157	E-->T	
3CI0K	155 - 159	158	Y-->A	
3CI0K	155 - 159	158	Y-->D	
3CI0K	155 - 159	158	Y-->E	
3CI0K	155 - 159	158	Y-->G	
3CI0K	155 - 159	158	Y-->I	
3CI0K	155 - 159	158	Y-->L	
3CI0K	155 - 159	158	Y-->N	
3CI0K	155 - 159	158	Y-->P	

3CI0K	155 - 159	158	Y-->S
3CI0K	155 - 159	158	Y-->T
3CI0K	155 - 159	158	Y-->V
3CI0K	155 - 159	159	L-->A
3CI0K	155 - 159	159	L-->F
3CI0K	155 - 159	159	L-->I
3CI0K	155 - 159	159	L-->P
3CI0K	155 - 159	159	L-->S
3CI0K	155 - 159	159	L-->T
3CI0K	217 - 222	218	I-->L
3CI0K	217 - 222	219	L-->P
3CI0K	217 - 222	222	L-->D
3CI0K	217 - 222	222	L-->G
3CI0K	217 - 222	222	L-->K
3CI0K	247 - 251	247	V-->G
3CI0K	247 - 251	247	V-->H
3CI0K	247 - 251	247	V-->I
3CI0K	247 - 251	247	V-->P
3CI0K	247 - 251	247	V-->R
3CI0K	247 - 251	247	V-->S
3CI0K	247 - 251	248	D-->A
3CI0K	247 - 251	248	D-->G
3CI0K	247 - 251	248	D-->T
3CI0K	247 - 251	249	Q-->C
3CI0K	247 - 251	249	Q-->E
3CI0K	247 - 251	249	Q-->G
3CI0K	247 - 251	249	Q-->I

3CI0K	247 - 251	249	Q-->K
3CI0K	247 - 251	249	Q-->L
3CI0K	247 - 251	249	Q-->M
3CI0K	247 - 251	249	Q-->P
3CI0K	247 - 251	249	Q-->V
3CI0K	247 - 251	249	Q-->Y
3CI0K	247 - 251	250	F-->D
3CI0K	247 - 251	250	F-->E
3CI0K	247 - 251	250	F-->G
3CI0K	247 - 251	250	F-->K
3CI0K	247 - 251	250	F-->L
3CI0K	247 - 251	250	F-->P
3CI0K	247 - 251	250	F-->S
3CI0K	247 - 251	250	F-->V
3CI0K	247 - 251	251	L-->P
3CI0K	247 - 251	251	L-->T
1S20A	33 - 38	33	F-->G
1S20A	33 - 38	34	A-->I
1S20A	33 - 38	35	R-->A
1S20A	33 - 38	35	R-->G
1S20A	183 - 188	188	L-->I
1S20A	183 - 188	188	L-->R
1S20A	183 - 188	188	L-->V
1S20A	224 - 228	224	W-->A
1S20A	224 - 228	224	W-->C
1S20A	224 - 228	224	W-->D
1S20A	224 - 228	224	W-->E

1S20A	224 - 228	224	W-->F
1S20A	224 - 228	224	W-->H
1S20A	224 - 228	224	W-->I
1S20A	224 - 228	224	W-->K
1S20A	224 - 228	224	W-->L
1S20A	224 - 228	224	W-->T
1S20A	224 - 228	224	W-->Y
1S20A	224 - 228	225	K-->A
1S20A	224 - 228	225	K-->G
1S20A	224 - 228	225	K-->P
1S20A	224 - 228	225	K-->S
1S20A	224 - 228	225	K-->T
1S20A	224 - 228	225	K-->Y
1S20A	224 - 228	226	G-->D
1S20A	224 - 228	226	G-->R
1S20A	224 - 228	226	G-->S
1S20A	224 - 228	227	S-->E
1S20A	224 - 228	227	S-->K
1S20A	224 - 228	227	S-->L
1S20A	224 - 228	227	S-->P
1S20A	224 - 228	227	S-->T
1S20A	224 - 228	228	G-->E
1S20A	224 - 228	228	G-->F
1S20A	224 - 228	228	G-->M
1S20A	224 - 228	228	G-->N
1S20A	224 - 228	228	G-->W
1S20A	230 - 234	230	S-->A

1S20A	230 - 234	230	S-->D
1S20A	230 - 234	230	S-->E
1S20A	230 - 234	230	S-->F
1S20A	230 - 234	230	S-->G
1S20A	230 - 234	230	S-->H
1S20A	230 - 234	230	S-->I
1S20A	230 - 234	230	S-->K
1S20A	230 - 234	230	S-->L
1S20A	230 - 234	230	S-->M
1S20A	230 - 234	230	S-->P
1S20A	230 - 234	230	S-->Q
1S20A	230 - 234	230	S-->V
1S20A	230 - 234	230	S-->Y
1S20A	230 - 234	231	I-->A
1S20A	230 - 234	231	I-->E
1S20A	230 - 234	231	I-->F
1S20A	230 - 234	231	I-->G
1S20A	230 - 234	231	I-->K
1S20A	230 - 234	231	I-->L
1S20A	230 - 234	231	I-->N
1S20A	230 - 234	231	I-->P
1S20A	230 - 234	231	I-->Q
1S20A	230 - 234	231	I-->S
1S20A	230 - 234	231	I-->T
1S20A	230 - 234	231	I-->V
1S20A	230 - 234	231	I-->Y
1S20A	230 - 234	232	V-->A

1S20A	230 - 234	232	V-->C
1S20A	230 - 234	232	V-->E
1S20A	230 - 234	232	V-->F
1S20A	230 - 234	232	V-->G
1S20A	230 - 234	232	V-->I
1S20A	230 - 234	232	V-->K
1S20A	230 - 234	232	V-->L
1S20A	230 - 234	232	V-->P
1S20A	230 - 234	232	V-->S
1S20A	230 - 234	232	V-->T
1S20A	230 - 234	232	V-->Y
1S20A	230 - 234	233	L-->A
1S20A	230 - 234	233	L-->D
1S20A	230 - 234	233	L-->G
1S20A	230 - 234	233	L-->I
1S20A	230 - 234	233	L-->K
1S20A	230 - 234	233	L-->N
1S20A	230 - 234	233	L-->P
1S20A	230 - 234	233	L-->S
1S20A	230 - 234	233	L-->V
1S20A	230 - 234	234	D-->A
1S20A	230 - 234	234	D-->E
1S20A	230 - 234	234	D-->F
1S20A	230 - 234	234	D-->G
1S20A	230 - 234	234	D-->N
1S20A	230 - 234	234	D-->Q
1S20A	230 - 234	234	D-->R

1S20A	230 - 234	234	D-->T
1S20A	230 - 234	234	D-->V
1S20A	236 - 241	238	T-->A
1S20A	236 - 241	239	L-->G
1S20A	236 - 241	241	S-->L
1S20A	236 - 241	241	S-->N
1S20A	236 - 241	241	S-->V
1U83A	191 - 195	191	V-->D
1U83A	191 - 195	191	V-->F
1U83A	191 - 195	191	V-->I
1U83A	191 - 195	191	V-->K
1U83A	191 - 195	191	V-->L
1U83A	191 - 195	191	V-->S
1U83A	191 - 195	192	D-->A
1U83A	191 - 195	192	D-->E
1U83A	191 - 195	192	D-->F
1U83A	191 - 195	192	D-->H
1U83A	191 - 195	192	D-->I
1U83A	191 - 195	192	D-->K
1U83A	191 - 195	192	D-->Q
1U83A	191 - 195	192	D-->R
1U83A	191 - 195	192	D-->S
1U83A	191 - 195	192	D-->V
1U83A	191 - 195	193	D-->A
1U83A	191 - 195	193	D-->C
1U83A	191 - 195	193	D-->E
1U83A	191 - 195	193	D-->F

1U83A	191 - 195	193	D-->G
1U83A	191 - 195	193	D-->I
1U83A	191 - 195	193	D-->K
1U83A	191 - 195	193	D-->L
1U83A	191 - 195	193	D-->P
1U83A	191 - 195	193	D-->S
1U83A	191 - 195	193	D-->V
1U83A	191 - 195	193	D-->Y
1U83A	191 - 195	194	I-->A
1U83A	191 - 195	194	I-->D
1U83A	191 - 195	194	I-->E
1U83A	191 - 195	194	I-->G
1U83A	191 - 195	194	I-->K
1U83A	191 - 195	194	I-->L
1U83A	191 - 195	194	I-->S
1U83A	191 - 195	194	I-->V
1U83A	191 - 195	194	I-->Y
1U83A	191 - 195	195	I-->D
1U83A	191 - 195	195	I-->E
1U83A	191 - 195	195	I-->F
1U83A	191 - 195	195	I-->L
1U83A	191 - 195	195	I-->Q
1U83A	191 - 195	195	I-->V
1IM8B	48 - 52	48	L-->E
1IM8B	48 - 52	48	L-->K
1IM8B	48 - 52	48	L-->P
1IM8B	48 - 52	48	L-->S

1IM8B	48 - 52	48	L-->V
1IM8B	48 - 52	49	A-->D
1IM8B	48 - 52	49	A-->G
1IM8B	48 - 52	49	A-->H
1IM8B	48 - 52	49	A-->K
1IM8B	48 - 52	49	A-->Q
1IM8B	48 - 52	49	A-->S
1IM8B	48 - 52	49	A-->W
1IM8B	48 - 52	50	E-->D
1IM8B	48 - 52	50	E-->F
1IM8B	48 - 52	50	E-->I
1IM8B	48 - 52	50	E-->L
1IM8B	48 - 52	50	E-->P
1IM8B	48 - 52	50	E-->Q
1IM8B	48 - 52	50	E-->S
1IM8B	48 - 52	50	E-->T
1IM8B	48 - 52	50	E-->W
1IM8B	48 - 52	50	E-->Y
1IM8B	48 - 52	51	R-->D
1IM8B	48 - 52	51	R-->E
1IM8B	48 - 52	51	R-->G
1IM8B	48 - 52	51	R-->H
1IM8B	48 - 52	51	R-->M
1IM8B	48 - 52	51	R-->Y
1IM8B	48 - 52	52	F-->A
1IM8B	48 - 52	52	F-->D
1IM8B	48 - 52	52	F-->G

1IM8B	48	52	52	F-->I
1IM8B	48	52	52	F-->K
1IM8B	48	52	52	F-->R
1IM8B	48	52	52	F-->S
1IM8B	48	52	52	F-->V
1IM8B	68	74	74	R-->E
1UZRA	166	170	166	L-->G
1UZRA	166	170	166	L-->I
1UZRA	166	170	166	L-->P
1UZRA	166	170	166	L-->Q
1UZRA	166	170	166	L-->T
1UZRA	166	170	166	L-->Y
1UZRA	166	170	167	F-->A
1UZRA	166	170	167	F-->D
1UZRA	166	170	167	F-->E
1UZRA	166	170	167	F-->G
1UZRA	166	170	167	F-->I
1UZRA	166	170	167	F-->L
1UZRA	166	170	167	F-->P
1UZRA	166	170	167	F-->V
1UZRA	166	170	167	F-->W
1UZRA	166	170	168	Y-->A
1UZRA	166	170	168	Y-->E
1UZRA	166	170	168	Y-->F
1UZRA	166	170	168	Y-->G
1UZRA	166	170	168	Y-->H
1UZRA	166	170	168	Y-->I

1UZRA	166	170	168	Y-->K
1UZRA	166	170	168	Y-->N
1UZRA	166	170	168	Y-->P
1UZRA	166	170	168	Y-->S
1UZRA	166	170	168	Y-->V
1UZRA	166	170	169	S-->A
1UZRA	166	170	169	S-->E
1UZRA	166	170	169	S-->G
1UZRA	166	170	169	S-->P
1UZRA	166	170	169	S-->R
1UZRA	166	170	169	S-->V
1UZRA	166	170	169	S-->Y
1UZRA	166	170	170	G-->A
1UZRA	166	170	170	G-->D
1UZRA	166	170	170	G-->M
1UZRA	166	170	170	G-->P
1UZRA	285	290	286	A-->N
1UZRA	285	290	286	A-->V
1UZRA	285	290	287	I-->D
1UZRA	285	290	287	I-->N
1UZRA	285	290	287	I-->S
1UZRA	285	290	288	L-->M
1UZRA	285	290	288	L-->P
1UZRA	285	290	290	A-->V
2FPSB	110	115	110	K-->G
2FPSB	110	115	113	E-->F
2FPSB	110	115	115	Y-->L

1WQLB	41	45	41	Y-->A
1WQLB	41	45	41	Y-->G
1WQLB	41	45	41	Y-->M
1WQLB	41	45	41	Y-->P
1WQLB	41	45	41	Y-->S
1WQLB	41	45	42	E-->A
1WQLB	41	45	42	E-->L
1WQLB	41	45	42	E-->P
1WQLB	41	45	43	A-->V
1WQLB	41	45	44	W-->D
1WQLB	41	45	44	W-->E
1WQLB	41	45	44	W-->H
1WQLB	41	45	44	W-->I
1WQLB	41	45	44	W-->Y
1WQLB	41	45	45	L-->F
1WQLB	64	69	69	M-->I
1CL2B	352	357	353	E-->L
1BG8A	18	22	18	C-->D
1BG8A	18	22	18	C-->E
1BG8A	18	22	18	C-->K
1BG8A	18	22	18	C-->P
1BG8A	18	22	19	E-->G
1BG8A	18	22	20	D-->L
1BG8A	18	22	21	F-->I
1BG8A	18	22	21	F-->L
1B4UA	57	62	57	E-->R
1B4UA	57	62	57	E-->V

1B4UA	57 - 62	58	S-->G	
1B4UA	57 - 62	59	A-->N	
1B4UA	57 - 62	62	D-->V	
1B4UA	79 - 84	82	M-->N	
1B4UA	79 - 84	82	M-->Q	
1Z6GA	104 - 110	110	L-->P	
1IPIA	109 - 113	109	L-->A	
1IPIA	109 - 113	109	L-->D	
1IPIA	109 - 113	109	L-->E	
1IPIA	109 - 113	109	L-->F	
1IPIA	109 - 113	109	L-->G	
1IPIA	109 - 113	109	L-->H	
1IPIA	109 - 113	109	L-->K	
1IPIA	109 - 113	109	L-->N	
1IPIA	109 - 113	109	L-->P	
1IPIA	109 - 113	109	L-->Q	
1IPIA	109 - 113	109	L-->R	
1IPIA	109 - 113	109	L-->S	
1IPIA	109 - 113	109	L-->V	
1IPIA	109 - 113	109	L-->Y	
1IPIA	109 - 113	110	E-->A	
1IPIA	109 - 113	110	E-->C	
1IPIA	109 - 113	110	E-->D	
1IPIA	109 - 113	110	E-->F	
1IPIA	109 - 113	110	E-->G	
1IPIA	109 - 113	110	E-->H	
1IPIA	109 - 113	110	E-->I	

1IPIA	109 - 113	110	E-->K
1IPIA	109 - 113	110	E-->L
1IPIA	109 - 113	110	E-->M
1IPIA	109 - 113	110	E-->N
1IPIA	109 - 113	110	E-->P
1IPIA	109 - 113	110	E-->R
1IPIA	109 - 113	110	E-->S
1IPIA	109 - 113	110	E-->T
1IPIA	109 - 113	110	E-->V
1IPIA	109 - 113	111	V-->A
1IPIA	109 - 113	111	V-->D
1IPIA	109 - 113	111	V-->G
1IPIA	109 - 113	111	V-->I
1IPIA	109 - 113	111	V-->K
1IPIA	109 - 113	111	V-->L
1IPIA	109 - 113	111	V-->M
1IPIA	109 - 113	111	V-->N
1IPIA	109 - 113	111	V-->P
1IPIA	109 - 113	111	V-->R
1IPIA	109 - 113	112	L-->A
1IPIA	109 - 113	112	L-->D
1IPIA	109 - 113	112	L-->E
1IPIA	109 - 113	112	L-->F
1IPIA	109 - 113	112	L-->G
1IPIA	109 - 113	112	L-->H
1IPIA	109 - 113	112	L-->I
1IPIA	109 - 113	112	L-->K

1IPIA	109	- 113	112	L-->N
1IPIA	109	- 113	112	L-->P
1IPIA	109	- 113	112	L-->Q
1IPIA	109	- 113	112	L-->R
1IPIA	109	- 113	112	L-->S
1IPIA	109	- 113	112	L-->T
1IPIA	109	- 113	112	L-->V
1IPIA	109	- 113	113	L-->A
1IPIA	109	- 113	113	L-->D
1IPIA	109	- 113	113	L-->E
1IPIA	109	- 113	113	L-->F
1IPIA	109	- 113	113	L-->G
1IPIA	109	- 113	113	L-->H
1IPIA	109	- 113	113	L-->K
1IPIA	109	- 113	113	L-->N
1IPIA	109	- 113	113	L-->P
1IPIA	109	- 113	113	L-->Q
1IPIA	109	- 113	113	L-->V
2F7CA	98	- 102	98	S-->K
2F7CA	98	- 102	98	S-->L
2F7CA	98	- 102	98	S-->N
2F7CA	98	- 102	98	S-->Q
2F7CA	98	- 102	98	S-->T
2F7CA	98	- 102	98	S-->V
2F7CA	98	- 102	98	S-->Y
2F7CA	98	- 102	99	S-->E
2F7CA	98	- 102	99	S-->K

2F7CA	98	- 102	99	S-->Q
2F7CA	98	- 102	99	S-->T
2F7CA	98	- 102	99	S-->V
2F7CA	98	- 102	100	L-->D
2F7CA	98	- 102	100	L-->E
2F7CA	98	- 102	100	L-->G
2F7CA	98	- 102	100	L-->H
2F7CA	98	- 102	100	L-->K
2F7CA	98	- 102	100	L-->N
2F7CA	98	- 102	100	L-->P
2F7CA	98	- 102	100	L-->T
2F7CA	98	- 102	100	L-->V
2F7CA	98	- 102	101	L-->A
2F7CA	98	- 102	101	L-->E
2F7CA	98	- 102	101	L-->K
2F7CA	98	- 102	101	L-->M
2F7CA	98	- 102	101	L-->P
2F7CA	98	- 102	101	L-->Q
2F7CA	98	- 102	101	L-->R
2F7CA	98	- 102	101	L-->S
2F7CA	98	- 102	101	L-->W
2F7CA	98	- 102	102	Y-->A
2F7CA	98	- 102	102	Y-->D
2F7CA	98	- 102	102	Y-->E
2F7CA	98	- 102	102	Y-->F
2F7CA	98	- 102	102	Y-->G
2F7CA	98	- 102	102	Y-->H

2F7CA	98	102	102	Y-->I
2F7CA	98	102	102	Y-->K
2F7CA	98	102	102	Y-->N
2F7CA	98	102	102	Y-->P
2F7CA	98	102	102	Y-->Q
2F7CA	98	102	102	Y-->S
2F7CA	98	102	102	Y-->V
2F7CA	245	249	245	A-->C
2F7CA	245	249	245	A-->D
2F7CA	245	249	245	A-->G
2F7CA	245	249	245	A-->L
2F7CA	245	249	245	A-->N
2F7CA	245	249	245	A-->P
2F7CA	245	249	245	A-->S
2F7CA	245	249	245	A-->Y
2F7CA	245	249	246	S-->F
2F7CA	245	249	246	S-->R
2F7CA	245	249	247	A-->F
2F7CA	245	249	247	A-->G
2F7CA	245	249	247	A-->L
2F7CA	245	249	248	M-->D
2F7CA	245	249	248	M-->F
2F7CA	245	249	248	M-->G
2F7CA	245	249	248	M-->H
2F7CA	245	249	248	M-->I
2F7CA	245	249	248	M-->K
2F7CA	245	249	248	M-->L

2F7CA	245 - 249	248	M-->N
2F7CA	245 - 249	248	M-->P
2F7CA	245 - 249	248	M-->Q
2F7CA	245 - 249	248	M-->S
2F7CA	245 - 249	248	M-->V
2F7CA	245 - 249	248	M-->W
2F7CA	245 - 249	249	D-->G
2A2OD	197 - 201	197	N-->E
2A2OD	197 - 201	197	N-->F
2A2OD	197 - 201	197	N-->I
2A2OD	197 - 201	197	N-->L
2A2OD	197 - 201	198	G-->T
2A2OD	197 - 201	198	G-->V
2A2OD	197 - 201	198	G-->W
2A2OD	197 - 201	199	A-->E
2A2OD	197 - 201	199	A-->H
2A2OD	197 - 201	199	A-->K
2A2OD	197 - 201	199	A-->L
2A2OD	197 - 201	199	A-->Q
2A2OD	197 - 201	199	A-->S
2A2OD	197 - 201	199	A-->T
2A2OD	197 - 201	199	A-->V
2A2OD	197 - 201	200	Y-->A
2A2OD	197 - 201	200	Y-->F
2A2OD	197 - 201	200	Y-->G
2A2OD	197 - 201	200	Y-->M
2A2OD	197 - 201	200	Y-->N

2A2OD	197 - 201	200	Y-->W
2A2OD	197 - 201	201	Q-->A
2A2OD	197 - 201	201	Q-->D
2A2OD	197 - 201	201	Q-->F
2A2OD	197 - 201	201	Q-->K
2A2OD	197 - 201	201	Q-->L
2A2OD	197 - 201	201	Q-->P
2A2OD	197 - 201	201	Q-->S
2A2OD	197 - 201	201	Q-->T
2A2OD	197 - 201	201	Q-->Y
1VH9A	9 - 14	10	D-->K
1VH9A	9 - 14	10	D-->T
1VH9A	9 - 14	11	E-->N
1VH9A	9 - 14	12	L-->N
1VH9A	9 - 14	14	A-->L
1SX8A	153 - 158	155	E-->N
1SX8A	153 - 158	156	L-->E
1SX8A	153 - 158	157	N-->V
1SX8A	196 - 201	199	F-->K
2IG3A	68 - 73	69	L-->D
2IG3A	68 - 73	72	H-->P
1R8GA	182 - 186	182	R-->H
1R8GA	182 - 186	182	R-->K
1R8GA	182 - 186	182	R-->N
1R8GA	182 - 186	182	R-->T
1R8GA	182 - 186	182	R-->V
1R8GA	182 - 186	183	P-->E

1R8GA	182	186	183	P-->K
1R8GA	182	186	184	N-->A
1R8GA	182	186	184	N-->F
1R8GA	182	186	184	N-->G
1R8GA	182	186	184	N-->I
1R8GA	182	186	184	N-->L
1R8GA	182	186	184	N-->P
1R8GA	182	186	184	N-->R
1R8GA	182	186	184	N-->T
1R8GA	182	186	184	N-->V
1R8GA	182	186	185	I-->L
1R8GA	182	186	185	I-->T
1R8GA	182	186	186	F-->G
1R8GA	182	186	186	F-->I
1R8GA	182	186	186	F-->L
1R8GA	182	186	186	F-->P
1R8GA	182	186	186	F-->R
1R8GA	182	186	186	F-->Y
1R8GA	247	252	247	L-->G
1R8GA	247	252	248	S-->L
1R8GA	247	252	249	H-->E
1R8GA	247	252	249	H-->G
1R8GA	247	252	249	H-->I
1R8GA	349	353	349	R-->A
1R8GA	349	353	349	R-->D
1R8GA	349	353	349	R-->E
1R8GA	349	353	349	R-->G

1R8GA	349 - 353	349	R-->K	
1R8GA	349 - 353	349	R-->L	
1R8GA	349 - 353	349	R-->N	
1R8GA	349 - 353	349	R-->S	
1R8GA	349 - 353	349	R-->V	
1R8GA	349 - 353	349	R-->Y	
1R8GA	349 - 353	350	D-->A	
1R8GA	349 - 353	350	D-->E	
1R8GA	349 - 353	350	D-->F	
1R8GA	349 - 353	350	D-->I	
1R8GA	349 - 353	350	D-->L	
1R8GA	349 - 353	350	D-->V	
1R8GA	349 - 353	351	F-->D	
1R8GA	349 - 353	351	F-->G	
1R8GA	349 - 353	351	F-->K	
1R8GA	349 - 353	351	F-->L	
1R8GA	349 - 353	351	F-->R	
1R8GA	349 - 353	351	F-->S	
1R8GA	349 - 353	351	F-->V	
1R8GA	349 - 353	352	V-->A	
1R8GA	349 - 353	352	V-->D	
1R8GA	349 - 353	352	V-->I	
1R8GA	349 - 353	352	V-->L	
1R8GA	349 - 353	352	V-->P	
1R8GA	349 - 353	352	V-->R	
1R8GA	349 - 353	353	A-->D	
1R8GA	349 - 353	353	A-->E	

1R8GA	349 - 353	353	A-->I
1R8GA	349 - 353	353	A-->L
1R8GA	349 - 353	353	A-->N
1R8GA	349 - 353	353	A-->P
1R8GA	349 - 353	353	A-->S
1R8GA	349 - 353	353	A-->T
1R8GA	349 - 353	353	A-->W
1R8GA	349 - 353	353	A-->Y
2RCFB	57 - 61	57	A-->E
2RCFB	57 - 61	57	A-->G
2RCFB	57 - 61	57	A-->L
2RCFB	57 - 61	57	A-->N
2RCFB	57 - 61	57	A-->P
2RCFB	57 - 61	57	A-->R
2RCFB	57 - 61	57	A-->T
2RCFB	57 - 61	57	A-->V
2RCFB	57 - 61	57	A-->Y
2RCFB	57 - 61	58	A-->C
2RCFB	57 - 61	58	A-->D
2RCFB	57 - 61	58	A-->F
2RCFB	57 - 61	58	A-->I
2RCFB	57 - 61	58	A-->K
2RCFB	57 - 61	58	A-->L
2RCFB	57 - 61	58	A-->N
2RCFB	57 - 61	58	A-->Q
2RCFB	57 - 61	58	A-->V
2RCFB	57 - 61	59	R-->A

2RCFB	57 - 61	59	R-->C	
2RCFB	57 - 61	59	R-->E	
2RCFB	57 - 61	59	R-->F	
2RCFB	57 - 61	59	R-->G	
2RCFB	57 - 61	59	R-->H	
2RCFB	57 - 61	59	R-->S	
2RCFB	57 - 61	59	R-->T	
2RCFB	57 - 61	60	E-->A	
2RCFB	57 - 61	60	E-->G	
2RCFB	57 - 61	60	E-->I	
2RCFB	57 - 61	60	E-->K	
2RCFB	57 - 61	60	E-->R	
2RCFB	57 - 61	60	E-->T	
2RCFB	57 - 61	60	E-->Y	
2RCFB	57 - 61	61	A-->D	
2RCFB	57 - 61	61	A-->E	
2RCFB	57 - 61	61	A-->G	
2RCFB	57 - 61	61	A-->H	
2RCFB	57 - 61	61	A-->I	
2RCFB	57 - 61	61	A-->V	
1ZK8A	99 - 104	100	G-->N	
1ZK8A	99 - 104	101	L-->D	
1ZK8A	99 - 104	103	E-->G	
1ZK8A	99 - 104	103	E-->I	
3CSVA	136 - 141	138	T-->P	
3CSVA	236 - 241	238	V-->G	
3CSVA	236 - 241	241	Q-->E	

3CSVA	236 - 241	241	Q-->G
2EWCK	50 - 54	50	E-->A
2EWCK	50 - 54	50	E-->D
2EWCK	50 - 54	50	E-->K
2EWCK	50 - 54	50	E-->L
2EWCK	50 - 54	50	E-->M
2EWCK	50 - 54	50	E-->S
2EWCK	50 - 54	50	E-->T
2EWCK	50 - 54	51	R-->E
2EWCK	50 - 54	51	R-->F
2EWCK	50 - 54	51	R-->G
2EWCK	50 - 54	51	R-->I
2EWCK	50 - 54	51	R-->K
2EWCK	50 - 54	51	R-->L
2EWCK	50 - 54	51	R-->N
2EWCK	50 - 54	51	R-->P
2EWCK	50 - 54	51	R-->V
2EWCK	50 - 54	52	R-->G
2EWCK	50 - 54	52	R-->K
2EWCK	50 - 54	52	R-->L
2EWCK	50 - 54	52	R-->P
2EWCK	50 - 54	52	R-->S
2EWCK	50 - 54	52	R-->V
2EWCK	50 - 54	53	L-->E
2EWCK	50 - 54	53	L-->G
2EWCK	50 - 54	53	L-->I
2EWCK	50 - 54	53	L-->M

2EWCK	50 - 54	53	L-->P
2EWCK	50 - 54	53	L-->Q
2EWCK	50 - 54	53	L-->V
2EWCK	50 - 54	53	L-->Y
2EWCK	50 - 54	54	A-->E
2EWCK	50 - 54	54	A-->K
2EWCK	50 - 54	54	A-->N
2EWCK	50 - 54	54	A-->P
2EWCK	50 - 54	54	A-->S
2EWCK	50 - 54	54	A-->V
2EWCK	73 - 78	74	W-->T
2EWCK	73 - 78	75	N-->K
2EWCK	73 - 78	76	I-->G
2IJPB	83 - 87	83	K-->A
2IJPB	83 - 87	83	K-->F
2IJPB	83 - 87	83	K-->L
2IJPB	83 - 87	83	K-->N
2IJPB	83 - 87	83	K-->P
2IJPB	83 - 87	83	K-->S
2IJPB	83 - 87	83	K-->V
2IJPB	83 - 87	83	K-->Y
2IJPB	83 - 87	84	S-->E
2IJPB	83 - 87	84	S-->F
2IJPB	83 - 87	84	S-->G
2IJPB	83 - 87	84	S-->K
2IJPB	83 - 87	84	S-->N
2IJPB	83 - 87	84	S-->R

2IJPB	83 - 87	85	I-->D
2IJPB	83 - 87	85	I-->E
2IJPB	83 - 87	85	I-->F
2IJPB	83 - 87	85	I-->G
2IJPB	83 - 87	85	I-->H
2IJPB	83 - 87	85	I-->P
2IJPB	83 - 87	85	I-->S
2IJPB	83 - 87	85	I-->V
2IJPB	83 - 87	86	K-->G
2IJPB	83 - 87	86	K-->P
2IJPB	83 - 87	86	K-->Q
2IJPB	83 - 87	86	K-->R
2IJPB	83 - 87	86	K-->V
2IJPB	83 - 87	86	K-->Y
2IJPB	83 - 87	87	S-->A
2IJPB	83 - 87	87	S-->D
2IJPB	83 - 87	87	S-->E
2IJPB	83 - 87	87	S-->F
2IJPB	83 - 87	87	S-->T
2IJPB	83 - 87	87	S-->V
1H1OA	58 - 63	58	Q-->V
1H1OA	65 - 69	65	W-->A
1H1OA	65 - 69	65	W-->D
1H1OA	65 - 69	65	W-->P
1H1OA	65 - 69	65	W-->R
1H1OA	65 - 69	65	W-->V
1H1OA	65 - 69	65	W-->Y

1H1OA	65 - 69	66	P-->D	
1H1OA	65 - 69	66	P-->V	
1H1OA	65 - 69	67	V-->K	
1H1OA	65 - 69	67	V-->L	
1H1OA	65 - 69	67	V-->T	
1H1OA	65 - 69	69	Q-->F	
1H1OA	65 - 69	69	Q-->G	
1H1OA	65 - 69	69	Q-->L	
1H1OA	65 - 69	69	Q-->R	
2O35B	75 - 80	76	E-->F	
2B59A	114 - 119	118	K-->M	
2P04B	34 - 38	34	E-->A	
2P04B	34 - 38	34	E-->D	
2P04B	34 - 38	34	E-->G	
2P04B	34 - 38	34	E-->I	
2P04B	34 - 38	34	E-->L	
2P04B	34 - 38	34	E-->P	
2P04B	34 - 38	34	E-->R	
2P04B	34 - 38	34	E-->S	
2P04B	34 - 38	34	E-->T	
2P04B	34 - 38	35	V-->A	
2P04B	34 - 38	35	V-->D	
2P04B	34 - 38	35	V-->I	
2P04B	34 - 38	35	V-->K	
2P04B	34 - 38	35	V-->P	
2P04B	34 - 38	35	V-->Q	
2P04B	34 - 38	35	V-->R	

2P04B	34 - 38	35	V-->Y
2P04B	34 - 38	36	L-->A
2P04B	34 - 38	36	L-->E
2P04B	34 - 38	36	L-->G
2P04B	34 - 38	36	L-->I
2P04B	34 - 38	36	L-->N
2P04B	34 - 38	36	L-->P
2P04B	34 - 38	36	L-->R
2P04B	34 - 38	36	L-->V
2P04B	34 - 38	36	L-->Y
2P04B	34 - 38	37	E-->A
2P04B	34 - 38	37	E-->F
2P04B	34 - 38	37	E-->G
2P04B	34 - 38	37	E-->K
2P04B	34 - 38	37	E-->L
2P04B	34 - 38	37	E-->N
2P04B	34 - 38	37	E-->P
2P04B	34 - 38	37	E-->S
2P04B	34 - 38	37	E-->V
2P04B	34 - 38	38	R-->A
2P04B	34 - 38	38	R-->C
2P04B	34 - 38	38	R-->D
2P04B	34 - 38	38	R-->E
2P04B	34 - 38	38	R-->F
2P04B	34 - 38	38	R-->G
2P04B	34 - 38	38	R-->H
2P04B	34 - 38	38	R-->I

2P04B	34 - 38	38	R-->K
2P04B	34 - 38	38	R-->L
2P04B	34 - 38	38	R-->P
2P04B	34 - 38	38	R-->Q
2P04B	34 - 38	38	R-->S
2P04B	34 - 38	38	R-->V
2P04B	34 - 38	38	R-->W
2P04B	34 - 38	38	R-->Y
2P04B	64 - 68	64	F-->A
2P04B	64 - 68	64	F-->K
2P04B	64 - 68	64	F-->N
2P04B	64 - 68	64	F-->R
2P04B	64 - 68	64	F-->V
2P04B	64 - 68	64	F-->Y
2P04B	64 - 68	65	D-->A
2P04B	64 - 68	65	D-->C
2P04B	64 - 68	65	D-->G
2P04B	64 - 68	65	D-->P
2P04B	64 - 68	65	D-->R
2P04B	64 - 68	65	D-->V
2P04B	64 - 68	65	D-->Y
2P04B	64 - 68	66	A-->K
2P04B	64 - 68	66	A-->L
2P04B	64 - 68	66	A-->N
2P04B	64 - 68	66	A-->P
2P04B	64 - 68	66	A-->T
2P04B	64 - 68	66	A-->V

2P04B	64 - 68	66	A-->Y
2P04B	64 - 68	67	I-->D
2P04B	64 - 68	67	I-->F
2P04B	64 - 68	67	I-->G
2P04B	64 - 68	67	I-->H
2P04B	64 - 68	67	I-->K
2P04B	64 - 68	67	I-->M
2P04B	64 - 68	67	I-->N
2P04B	64 - 68	67	I-->P
2P04B	64 - 68	67	I-->R
2P04B	64 - 68	67	I-->T
2P04B	64 - 68	67	I-->V
2P04B	64 - 68	68	S-->A
2P04B	64 - 68	68	S-->D
2P04B	64 - 68	68	S-->F
2P04B	64 - 68	68	S-->G
2P04B	64 - 68	68	S-->I
2P04B	64 - 68	68	S-->R
2P04B	64 - 68	68	S-->T
2P04B	64 - 68	68	S-->V
1XQXA	204 - 208	204	V-->D
1XQXA	204 - 208	204	V-->H
1XQXA	204 - 208	204	V-->R
1XQXA	204 - 208	205	Y-->A
1XQXA	204 - 208	205	Y-->C
1XQXA	204 - 208	205	Y-->L
1XQXA	204 - 208	205	Y-->M

1XQXA	204 - 208	205	Y-->N
1XQXA	204 - 208	205	Y-->R
1XQXA	204 - 208	205	Y-->T
1XQXA	204 - 208	206	R-->F
1XQXA	204 - 208	206	R-->Q
1XQXA	204 - 208	207	I-->M
1XQXA	204 - 208	208	M-->E
1XQXA	204 - 208	208	M-->G
1XQXA	204 - 208	208	M-->I
1XQXA	204 - 208	208	M-->L
1XQXA	204 - 208	208	M-->P
1XQXA	204 - 208	208	M-->R
1XQXA	204 - 208	208	M-->S
2I7TA	80 - 86	81	P-->H
2I7TA	80 - 86	81	P-->T
2I7TA	363 - 367	363	L-->A
2I7TA	363 - 367	363	L-->G
2I7TA	363 - 367	363	L-->N
2I7TA	363 - 367	363	L-->T
2I7TA	363 - 367	364	A-->G
2I7TA	363 - 367	364	A-->I
2I7TA	363 - 367	364	A-->K
2I7TA	363 - 367	364	A-->W
2I7TA	363 - 367	365	K-->G
2I7TA	363 - 367	365	K-->I
2I7TA	363 - 367	365	K-->P
2I7TA	363 - 367	366	H-->A

2I7TA	363 - 367	366	H-->D
2I7TA	363 - 367	366	H-->K
2I7TA	363 - 367	366	H-->N
2I7TA	363 - 367	366	H-->P
2I7TA	363 - 367	366	H-->R
2I7TA	363 - 367	366	H-->S
2I7TA	363 - 367	366	H-->T
2I7TA	363 - 367	366	H-->W
2I7TA	363 - 367	367	I-->P
2I7TA	363 - 367	367	I-->S
1J1VA	391 - 395	391	V-->A
1J1VA	391 - 395	391	V-->D
1J1VA	391 - 395	391	V-->E
1J1VA	391 - 395	391	V-->G
1J1VA	391 - 395	391	V-->H
1J1VA	391 - 395	391	V-->K
1J1VA	391 - 395	391	V-->L
1J1VA	391 - 395	391	V-->N
1J1VA	391 - 395	391	V-->P
1J1VA	391 - 395	391	V-->R
1J1VA	391 - 395	391	V-->S
1J1VA	391 - 395	391	V-->T
1J1VA	391 - 395	391	V-->W
1J1VA	391 - 395	392	A-->C
1J1VA	391 - 395	392	A-->D
1J1VA	391 - 395	392	A-->F
1J1VA	391 - 395	392	A-->I

1J1VA	391 - 395	392	A-->L
1J1VA	391 - 395	392	A-->R
1J1VA	391 - 395	392	A-->W
1J1VA	391 - 395	392	A-->Y
1J1VA	391 - 395	393	D-->A
1J1VA	391 - 395	393	D-->F
1J1VA	391 - 395	393	D-->G
1J1VA	391 - 395	393	D-->H
1J1VA	391 - 395	393	D-->I
1J1VA	391 - 395	393	D-->K
1J1VA	391 - 395	393	D-->N
1J1VA	391 - 395	393	D-->P
1J1VA	391 - 395	393	D-->Q
1J1VA	391 - 395	393	D-->R
1J1VA	391 - 395	393	D-->S
1J1VA	391 - 395	393	D-->V
1J1VA	391 - 395	394	L-->A
1J1VA	391 - 395	394	L-->D
1J1VA	391 - 395	394	L-->F
1J1VA	391 - 395	394	L-->G
1J1VA	391 - 395	394	L-->P
1J1VA	391 - 395	394	L-->T
1J1VA	391 - 395	395	L-->C
1J1VA	391 - 395	395	L-->D
1J1VA	391 - 395	395	L-->E
1J1VA	391 - 395	395	L-->F
1J1VA	391 - 395	395	L-->G

1J1VA	391 - 395	395	L-->K	
1J1VA	391 - 395	395	L-->P	
1J1VA	391 - 395	395	L-->Q	
1J1VA	391 - 395	395	L-->S	
1J1VA	391 - 395	395	L-->T	
1J1VA	391 - 395	395	L-->V	
1J1VA	412 - 417	412	A-->P	
1J1VA	412 - 417	413	L-->K	
1J1VA	412 - 417	415	K-->L	
1TUEA	515 - 520	515	F-->G	
1TUEA	515 - 520	515	F-->S	
1TUEA	515 - 520	516	W-->E	
1TUEA	515 - 520	516	W-->V	
1TUEA	544 - 548	544	R-->G	
1TUEA	544 - 548	544	R-->H	
1TUEA	544 - 548	544	R-->I	
1TUEA	544 - 548	544	R-->P	
1TUEA	544 - 548	544	R-->V	
1TUEA	544 - 548	545	N-->A	
1TUEA	544 - 548	545	N-->C	
1TUEA	544 - 548	545	N-->G	
1TUEA	544 - 548	545	N-->I	
1TUEA	544 - 548	545	N-->L	
1TUEA	544 - 548	545	N-->P	
1TUEA	544 - 548	545	N-->R	
1TUEA	544 - 548	545	N-->T	
1TUEA	544 - 548	545	N-->W	

1TUEA	544 - 548	546	A-->E
1TUEA	544 - 548	546	A-->L
1TUEA	544 - 548	546	A-->N
1TUEA	544 - 548	546	A-->R
1TUEA	544 - 548	546	A-->T
1TUEA	544 - 548	547	L-->D
1TUEA	544 - 548	547	L-->M
1TUEA	544 - 548	547	L-->Q
1TUEA	544 - 548	548	D-->A
1TUEA	544 - 548	548	D-->N
1TUEA	544 - 548	548	D-->P
1TUEA	544 - 548	548	D-->R
1TUEA	544 - 548	548	D-->S
1TUEA	544 - 548	548	D-->T
1TUEA	544 - 548	548	D-->Y
1US8B	857 - 862	858	E-->V
1US8B	857 - 862	862	A-->G
1S7MA	647 - 651	647	V-->E
1S7MA	647 - 651	647	V-->K
1S7MA	647 - 651	647	V-->L
1S7MA	647 - 651	647	V-->P
1S7MA	647 - 651	647	V-->Q
1S7MA	647 - 651	647	V-->R
1S7MA	647 - 651	647	V-->S
1S7MA	647 - 651	648	G-->C
1S7MA	647 - 651	648	G-->D
1S7MA	647 - 651	648	G-->E

1S7MA	647 - 651	648	G-->F
1S7MA	647 - 651	648	G-->I
1S7MA	647 - 651	648	G-->K
1S7MA	647 - 651	648	G-->L
1S7MA	647 - 651	648	G-->N
1S7MA	647 - 651	648	G-->P
1S7MA	647 - 651	648	G-->R
1S7MA	647 - 651	648	G-->T
1S7MA	647 - 651	648	G-->V
1S7MA	647 - 651	649	D-->A
1S7MA	647 - 651	649	D-->E
1S7MA	647 - 651	649	D-->F
1S7MA	647 - 651	649	D-->G
1S7MA	647 - 651	649	D-->I
1S7MA	647 - 651	649	D-->K
1S7MA	647 - 651	649	D-->L
1S7MA	647 - 651	649	D-->N
1S7MA	647 - 651	649	D-->R
1S7MA	647 - 651	649	D-->S
1S7MA	647 - 651	649	D-->T
1S7MA	647 - 651	649	D-->V
1S7MA	647 - 651	649	D-->Y
1S7MA	647 - 651	650	L-->D
1S7MA	647 - 651	650	L-->K
1S7MA	647 - 651	650	L-->M
1S7MA	647 - 651	650	L-->N
1S7MA	647 - 651	650	L-->P

1S7MA	647 - 651	650	L-->R
1S7MA	647 - 651	650	L-->S
1S7MA	647 - 651	650	L-->T
1S7MA	647 - 651	650	L-->V
1S7MA	647 - 651	651	R-->E
1S7MA	647 - 651	651	R-->G
1S7MA	647 - 651	651	R-->H
1S7MA	647 - 651	651	R-->I
1S7MA	647 - 651	651	R-->K
1S7MA	647 - 651	651	R-->N
1S7MA	647 - 651	651	R-->P
1S7MA	647 - 651	651	R-->Q
1S7MA	647 - 651	651	R-->S
1S7MA	647 - 651	651	R-->T
1S7MA	647 - 651	651	R-->V
1S7MA	647 - 651	651	R-->Y
3BM2B	3 - 7	3	A-->D
3BM2B	3 - 7	3	A-->E
3BM2B	3 - 7	3	A-->G
3BM2B	3 - 7	3	A-->H
3BM2B	3 - 7	3	A-->K
3BM2B	3 - 7	3	A-->L
3BM2B	3 - 7	3	A-->Q
3BM2B	3 - 7	3	A-->S
3BM2B	3 - 7	3	A-->T
3BM2B	3 - 7	4	L-->A
3BM2B	3 - 7	4	L-->D

3BM2B	3 - 7	4	L-->E
3BM2B	3 - 7	4	L-->F
3BM2B	3 - 7	4	L-->G
3BM2B	3 - 7	4	L-->I
3BM2B	3 - 7	4	L-->K
3BM2B	3 - 7	4	L-->P
3BM2B	3 - 7	4	L-->Q
3BM2B	3 - 7	5	E-->C
3BM2B	3 - 7	5	E-->G
3BM2B	3 - 7	5	E-->H
3BM2B	3 - 7	5	E-->K
3BM2B	3 - 7	5	E-->P
3BM2B	3 - 7	5	E-->S
3BM2B	3 - 7	5	E-->T
3BM2B	3 - 7	5	E-->V
3BM2B	3 - 7	5	E-->W
3BM2B	3 - 7	5	E-->Y
3BM2B	3 - 7	6	L-->D
3BM2B	3 - 7	6	L-->E
3BM2B	3 - 7	6	L-->G
3BM2B	3 - 7	6	L-->H
3BM2B	3 - 7	6	L-->I
3BM2B	3 - 7	6	L-->K
3BM2B	3 - 7	6	L-->N
3BM2B	3 - 7	6	L-->P
3BM2B	3 - 7	6	L-->Q
3BM2B	3 - 7	6	L-->R

3BM2B	3 - 7	6	L-->T
3BM2B	3 - 7	6	L-->V
3BM2B	3 - 7	6	L-->W
3BM2B	3 - 7	7	L-->A
3BM2B	3 - 7	7	L-->D
3BM2B	3 - 7	7	L-->E
3BM2B	3 - 7	7	L-->F
3BM2B	3 - 7	7	L-->G
3BM2B	3 - 7	7	L-->H
3BM2B	3 - 7	7	L-->I
3BM2B	3 - 7	7	L-->M
3BM2B	3 - 7	7	L-->N
3BM2B	3 - 7	7	L-->P
3BM2B	3 - 7	7	L-->R
3BM2B	3 - 7	7	L-->S
3BM2B	3 - 7	7	L-->V
3BM2B	104 - 108	104	R-->S
3BM2B	104 - 108	104	R-->T
3BM2B	104 - 108	105	W-->G
3BM2B	104 - 108	105	W-->I
3BM2B	104 - 108	106	E-->G
3BM2B	104 - 108	106	E-->S
3BM2B	104 - 108	107	Q-->A
3BM2B	104 - 108	107	Q-->E
3BM2B	104 - 108	107	Q-->F
3BM2B	104 - 108	107	Q-->P
3BM2B	104 - 108	108	E-->T

3BM2B	110	115	110	S-->T
3BM2B	110	115	110	S-->V
3BM2B	110	115	113	C-->E
3BM2B	110	115	113	C-->F
3BM2B	110	115	113	C-->L
3BM2B	110	115	113	C-->V
3BM2B	140	145	140	P-->I
3BM2B	140	145	141	V-->T
3BM2B	140	145	143	R-->S
1OKGA	110	115	111	C-->R
1OKGA	110	115	115	W-->D
1OKGA	110	115	115	W-->G
1OKGA	110	115	115	W-->T
2A9UB	17	22	18	K-->N
2A9UB	17	22	19	D-->W
2A9UB	17	22	20	L-->E
2A9UB	17	22	20	L-->I
2A9UB	17	22	21	N-->I
2A9UB	17	22	21	N-->K
2A9UB	17	22	21	N-->L
2PKWA	65	70	69	K-->Y
2PKWA	77	81	77	A-->F
2PKWA	77	81	77	A-->G
2PKWA	77	81	77	A-->K
2PKWA	77	81	77	A-->L
2PKWA	77	81	77	A-->Q
2PKWA	77	81	77	A-->R

2PKWA	77 - 81	77	A-->T
2PKWA	77 - 81	77	A-->V
2PKWA	77 - 81	78	V-->G
2PKWA	77 - 81	78	V-->H
2PKWA	77 - 81	78	V-->I
2PKWA	77 - 81	78	V-->K
2PKWA	77 - 81	78	V-->L
2PKWA	77 - 81	78	V-->N
2PKWA	77 - 81	78	V-->R
2PKWA	77 - 81	78	V-->T
2PKWA	77 - 81	79	A-->D
2PKWA	77 - 81	79	A-->E
2PKWA	77 - 81	79	A-->H
2PKWA	77 - 81	79	A-->I
2PKWA	77 - 81	79	A-->L
2PKWA	77 - 81	79	A-->R
2PKWA	77 - 81	79	A-->S
2PKWA	77 - 81	79	A-->V
2PKWA	77 - 81	80	K-->A
2PKWA	77 - 81	80	K-->C
2PKWA	77 - 81	80	K-->D
2PKWA	77 - 81	80	K-->G
2PKWA	77 - 81	80	K-->I
2PKWA	77 - 81	80	K-->L
2PKWA	77 - 81	80	K-->N
2PKWA	77 - 81	80	K-->P
2PKWA	77 - 81	80	K-->Q

2PKWA	77 - 81	80	K-->S
2PKWA	77 - 81	80	K-->T
2PKWA	77 - 81	80	K-->V
2PKWA	77 - 81	81	A-->D
2PKWA	77 - 81	81	A-->F
2PKWA	77 - 81	81	A-->H
2PKWA	77 - 81	81	A-->I
2PKWA	77 - 81	81	A-->N
2PKWA	77 - 81	81	A-->R
2PKWA	77 - 81	81	A-->S
2PKWA	77 - 81	81	A-->T
2IDOD	38 - 43	39	E-->F
2IDOD	38 - 43	43	R-->P
2FI0A	23 - 29	24	E-->S
2FI0A	23 - 29	28	I-->G
2FI0A	23 - 29	29	L-->K
3C2BB	57 - 63	62	A-->I
3C2BB	65 - 70	67	F-->E
3C2BB	65 - 70	68	Q-->I
3C2BB	65 - 70	69	Q-->I
3C2BB	65 - 70	69	Q-->S
3C2BB	206 - 211	211	E-->K
2PKPA	30 - 34	30	P-->D
2PKPA	30 - 34	30	P-->M
2PKPA	30 - 34	30	P-->T
2PKPA	30 - 34	30	P-->W
2PKPA	30 - 34	31	Y-->E

2PKPA	30 - 34	31	Y-->G
2PKPA	30 - 34	31	Y-->L
2PKPA	30 - 34	31	Y-->P
2PKPA	30 - 34	31	Y-->R
2PKPA	30 - 34	31	Y-->V
2PKPA	30 - 34	32	E-->A
2PKPA	30 - 34	32	E-->C
2PKPA	30 - 34	32	E-->D
2PKPA	30 - 34	32	E-->L
2PKPA	30 - 34	32	E-->M
2PKPA	30 - 34	32	E-->N
2PKPA	30 - 34	32	E-->R
2PKPA	30 - 34	32	E-->T
2PKPA	30 - 34	32	E-->V
2PKPA	30 - 34	33	L-->V
2PKPA	30 - 34	34	A-->D
2PKPA	30 - 34	34	A-->E
2PKPA	30 - 34	34	A-->G
2PKPA	30 - 34	34	A-->K
2PKPA	30 - 34	34	A-->P
2PKPA	30 - 34	34	A-->Q
2PKPA	30 - 34	34	A-->R
2PKPA	30 - 34	34	A-->T
2ZMFA	355 - 360	355	E-->V
2ZMFA	355 - 360	356	V-->S
2ZMFA	355 - 360	357	D-->S
2ZMFA	355 - 360	357	D-->T

2ZMFA	355 - 360	358	L-->I
2ZMFA	355 - 360	359	Y-->V
1VJQA	50 - 55	52	W-->G
1SVVA	28 - 33	28	P-->V
1SVVA	28 - 33	30	I-->A
1SVVA	187 - 191	187	L-->A
1SVVA	187 - 191	187	L-->C
1SVVA	187 - 191	187	L-->D
1SVVA	187 - 191	187	L-->E
1SVVA	187 - 191	187	L-->F
1SVVA	187 - 191	187	L-->G
1SVVA	187 - 191	187	L-->H
1SVVA	187 - 191	187	L-->I
1SVVA	187 - 191	187	L-->N
1SVVA	187 - 191	187	L-->S
1SVVA	187 - 191	187	L-->T
1SVVA	187 - 191	187	L-->V
1SVVA	187 - 191	188	A-->C
1SVVA	187 - 191	188	A-->D
1SVVA	187 - 191	188	A-->F
1SVVA	187 - 191	188	A-->G
1SVVA	187 - 191	188	A-->I
1SVVA	187 - 191	188	A-->L
1SVVA	187 - 191	188	A-->Q
1SVVA	187 - 191	188	A-->R
1SVVA	187 - 191	188	A-->T
1SVVA	187 - 191	188	A-->Y

1SVVA	187 - 191	189	S-->A
1SVVA	187 - 191	189	S-->F
1SVVA	187 - 191	189	S-->G
1SVVA	187 - 191	189	S-->H
1SVVA	187 - 191	189	S-->I
1SVVA	187 - 191	189	S-->K
1SVVA	187 - 191	189	S-->L
1SVVA	187 - 191	189	S-->M
1SVVA	187 - 191	189	S-->P
1SVVA	187 - 191	189	S-->V
1SVVA	187 - 191	189	S-->W
1SVVA	187 - 191	190	A-->D
1SVVA	187 - 191	190	A-->E
1SVVA	187 - 191	190	A-->H
1SVVA	187 - 191	190	A-->K
1SVVA	187 - 191	190	A-->N
1SVVA	187 - 191	190	A-->R
1SVVA	187 - 191	190	A-->S
1SVVA	187 - 191	190	A-->T
1SVVA	187 - 191	190	A-->W
1SVVA	187 - 191	190	A-->Y
1SVVA	187 - 191	191	L-->A
1SVVA	187 - 191	191	L-->D
1SVVA	187 - 191	191	L-->E
1SVVA	187 - 191	191	L-->F
1SVVA	187 - 191	191	L-->G
1SVVA	187 - 191	191	L-->H

1SVVA	187	191	191	L-->I
1SVVA	187	191	191	L-->P
1SVVA	187	191	191	L-->Q
1SVVA	187	191	191	L-->R
1SVVA	187	191	191	L-->S
1SVVA	187	191	191	L-->V
1SVVA	187	191	191	L-->Y
2PQ6A	159	164	164	E-->L
2PQ6A	258	263	258	L-->F
2PQ6A	258	263	258	L-->G
2PQ6A	258	263	261	L-->E
2PQ6A	258	263	262	L-->M
2PQ6A	284	289	288	W-->D
2PQ6A	363	367	363	Q-->A
2PQ6A	363	367	363	Q-->C
2PQ6A	363	367	363	Q-->E
2PQ6A	363	367	363	Q-->F
2PQ6A	363	367	363	Q-->G
2PQ6A	363	367	363	Q-->H
2PQ6A	363	367	363	Q-->I
2PQ6A	363	367	363	Q-->K
2PQ6A	363	367	363	Q-->L
2PQ6A	363	367	363	Q-->R
2PQ6A	363	367	363	Q-->S
2PQ6A	363	367	363	Q-->V
2PQ6A	363	367	363	Q-->W
2PQ6A	363	367	364	D-->A

2PQ6A	363 - 367	364	D-->C
2PQ6A	363 - 367	364	D-->K
2PQ6A	363 - 367	364	D-->P
2PQ6A	363 - 367	364	D-->R
2PQ6A	363 - 367	364	D-->S
2PQ6A	363 - 367	364	D-->T
2PQ6A	363 - 367	364	D-->Y
2PQ6A	363 - 367	365	K-->A
2PQ6A	363 - 367	365	K-->F
2PQ6A	363 - 367	365	K-->G
2PQ6A	363 - 367	365	K-->H
2PQ6A	363 - 367	365	K-->I
2PQ6A	363 - 367	365	K-->L
2PQ6A	363 - 367	365	K-->M
2PQ6A	363 - 367	365	K-->N
2PQ6A	363 - 367	365	K-->P
2PQ6A	363 - 367	365	K-->R
2PQ6A	363 - 367	365	K-->T
2PQ6A	363 - 367	365	K-->V
2PQ6A	363 - 367	366	V-->H
2PQ6A	363 - 367	366	V-->I
2PQ6A	363 - 367	366	V-->K
2PQ6A	363 - 367	366	V-->P
2PQ6A	363 - 367	366	V-->Q
2PQ6A	363 - 367	366	V-->R
2PQ6A	363 - 367	366	V-->Y
2PQ6A	363 - 367	367	L-->A

2PQ6A	363 - 367	367	L-->C
2PQ6A	363 - 367	367	L-->N
2PQ6A	363 - 367	367	L-->R
2PQ6A	363 - 367	367	L-->T
2F1RB	118 - 124	118	P-->F
2FUCA	96 - 100	96	I-->F
2FUCA	96 - 100	96	I-->H
2FUCA	96 - 100	98	N-->G
2FUCA	96 - 100	98	N-->H
2FUCA	96 - 100	98	N-->L
2FUCA	96 - 100	98	N-->S
2FUCA	96 - 100	99	H-->E
2FUCA	96 - 100	99	H-->S
2FUCA	96 - 100	99	H-->T
2FUCA	96 - 100	100	L-->F
2FUCA	200 - 205	201	C-->E
2FUCA	227 - 231	227	F-->E
2FUCA	227 - 231	227	F-->K
2FUCA	227 - 231	227	F-->N
2FUCA	227 - 231	227	F-->S
2FUCA	227 - 231	228	E-->T
2FUCA	227 - 231	229	I-->D
2FUCA	227 - 231	229	I-->E
2FUCA	227 - 231	229	I-->L
2FUCA	227 - 231	229	I-->N
2FUCA	227 - 231	229	I-->Q
2FUCA	227 - 231	229	I-->S

2FUCA	227	- 231	229	I-->V
2FUCA	227	- 231	230	F-->K
2FUCA	227	- 231	230	F-->L
2FUCA	227	- 231	230	F-->P
2FUCA	227	- 231	230	F-->R
2FUCA	227	- 231	230	F-->T
2FUCA	227	- 231	230	F-->W
2FUCA	227	- 231	231	A-->D
2FUCA	227	- 231	231	A-->E
2FUCA	227	- 231	231	A-->G
2FUCA	227	- 231	231	A-->K
2FUCA	227	- 231	231	A-->P
2FUCA	227	- 231	231	A-->T
1RZ4A	155	- 160	157	L-->G
1RZ4A	155	- 160	158	L-->P
1RZ4A	155	- 160	159	A-->N
1RZ4A	155	- 160	160	E-->G
1RZ4A	167	- 173	170	L-->I
2A5HB	31	- 35	31	V-->A
2A5HB	31	- 35	31	V-->D
2A5HB	31	- 35	31	V-->F
2A5HB	31	- 35	31	V-->G
2A5HB	31	- 35	31	V-->K
2A5HB	31	- 35	31	V-->P
2A5HB	31	- 35	31	V-->Q
2A5HB	31	- 35	31	V-->R
2A5HB	31	- 35	31	V-->S

2A5HB	31 - 35	32	E-->G
2A5HB	31 - 35	32	E-->H
2A5HB	31 - 35	32	E-->I
2A5HB	31 - 35	32	E-->K
2A5HB	31 - 35	32	E-->L
2A5HB	31 - 35	32	E-->P
2A5HB	31 - 35	32	E-->R
2A5HB	31 - 35	32	E-->V
2A5HB	31 - 35	32	E-->W
2A5HB	31 - 35	33	E-->D
2A5HB	31 - 35	33	E-->G
2A5HB	31 - 35	33	E-->I
2A5HB	31 - 35	33	E-->K
2A5HB	31 - 35	33	E-->L
2A5HB	31 - 35	33	E-->P
2A5HB	31 - 35	33	E-->S
2A5HB	31 - 35	33	E-->T
2A5HB	31 - 35	33	E-->V
2A5HB	31 - 35	33	E-->Y
2A5HB	31 - 35	34	L-->D
2A5HB	31 - 35	34	L-->F
2A5HB	31 - 35	34	L-->I
2A5HB	31 - 35	34	L-->P
2A5HB	31 - 35	34	L-->R
2A5HB	31 - 35	34	L-->S
2A5HB	31 - 35	34	L-->V
2A5HB	31 - 35	34	L-->Y

2A5HB	31 - 35	35	K-->A
2A5HB	31 - 35	35	K-->D
2A5HB	31 - 35	35	K-->E
2A5HB	31 - 35	35	K-->G
2A5HB	31 - 35	35	K-->I
2A5HB	31 - 35	35	K-->L
2A5HB	31 - 35	35	K-->P
2A5HB	31 - 35	35	K-->T
2A5HB	75 - 80	78	K-->N
2A5HB	213 - 217	213	P-->A
2A5HB	213 - 217	213	P-->D
2A5HB	213 - 217	213	P-->E
2A5HB	213 - 217	213	P-->G
2A5HB	213 - 217	213	P-->I
2A5HB	213 - 217	213	P-->N
2A5HB	213 - 217	213	P-->S
2A5HB	213 - 217	213	P-->T
2A5HB	213 - 217	213	P-->V
2A5HB	213 - 217	214	E-->A
2A5HB	213 - 217	214	E-->D
2A5HB	213 - 217	214	E-->H
2A5HB	213 - 217	214	E-->P
2A5HB	213 - 217	214	E-->Q
2A5HB	213 - 217	214	E-->R
2A5HB	213 - 217	214	E-->V
2A5HB	213 - 217	214	E-->W
2A5HB	213 - 217	215	L-->E

2A5HB	213 - 217	215	L-->G
2A5HB	213 - 217	215	L-->I
2A5HB	213 - 217	215	L-->K
2A5HB	213 - 217	215	L-->R
2A5HB	213 - 217	215	L-->S
2A5HB	213 - 217	215	L-->Y
2A5HB	213 - 217	216	V-->A
2A5HB	213 - 217	216	V-->K
2A5HB	213 - 217	216	V-->P
2A5HB	213 - 217	216	V-->Q
2A5HB	213 - 217	216	V-->S
2A5HB	213 - 217	216	V-->T
2A5HB	213 - 217	217	N-->C
2A5HB	213 - 217	217	N-->E
2A5HB	213 - 217	217	N-->F
2A5HB	213 - 217	217	N-->G
2A5HB	213 - 217	217	N-->K
2A5HB	213 - 217	217	N-->L
2A5HB	213 - 217	217	N-->R
2A5HB	213 - 217	217	N-->S
2A5HB	390 - 395	390	G-->K
2A5HB	390 - 395	390	G-->P
2A5HB	390 - 395	391	V-->L
2A5HB	390 - 395	391	V-->Y
2A5HB	390 - 395	392	A-->I
2A5HB	390 - 395	392	A-->L
2A5HB	390 - 395	393	G-->F

2A5HB	390 - 395	394	L-->A
2A5HB	390 - 395	394	L-->P
2A5HB	390 - 395	394	L-->V
2A5HB	390 - 395	395	L-->A
2A5HB	390 - 395	395	L-->P
2A5HB	390 - 395	395	L-->V
3BCZA	124 - 129	126	T-->E
3BCZA	124 - 129	127	D-->A
3BCZA	124 - 129	128	E-->F
2HH9B	64 - 69	68	W-->D
2P19A	66 - 71	67	D-->G
2P19A	66 - 71	67	D-->L
2P19A	66 - 71	67	D-->P
2P19A	66 - 71	67	D-->T
2P19A	66 - 71	69	L-->N
2P19A	66 - 71	69	L-->V
2P19A	66 - 71	70	E-->D
2P19A	66 - 71	71	K-->N
2P19A	66 - 71	71	K-->S
2P19A	101 - 105	101	E-->A
2P19A	101 - 105	101	E-->C
2P19A	101 - 105	101	E-->D
2P19A	101 - 105	101	E-->F
2P19A	101 - 105	101	E-->G
2P19A	101 - 105	101	E-->M
2P19A	101 - 105	101	E-->N
2P19A	101 - 105	101	E-->P

2P19A	101 - 105	101	E-->Q
2P19A	101 - 105	101	E-->R
2P19A	101 - 105	101	E-->S
2P19A	101 - 105	101	E-->T
2P19A	101 - 105	102	S-->E
2P19A	101 - 105	102	S-->F
2P19A	101 - 105	102	S-->G
2P19A	101 - 105	102	S-->I
2P19A	101 - 105	102	S-->K
2P19A	101 - 105	102	S-->N
2P19A	101 - 105	102	S-->P
2P19A	101 - 105	102	S-->Q
2P19A	101 - 105	102	S-->T
2P19A	101 - 105	102	S-->V
2P19A	101 - 105	103	T-->G
2P19A	101 - 105	103	T-->I
2P19A	101 - 105	103	T-->K
2P19A	101 - 105	103	T-->L
2P19A	101 - 105	103	T-->P
2P19A	101 - 105	103	T-->R
2P19A	101 - 105	103	T-->V
2P19A	101 - 105	104	L-->A
2P19A	101 - 105	104	L-->C
2P19A	101 - 105	104	L-->E
2P19A	101 - 105	104	L-->G
2P19A	101 - 105	104	L-->K
2P19A	101 - 105	104	L-->N

2P19A	101 - 105	104	L-->P
2P19A	101 - 105	104	L-->Q
2P19A	101 - 105	104	L-->S
2P19A	101 - 105	105	L-->F
2P19A	101 - 105	105	L-->H
2P19A	101 - 105	105	L-->K
2P19A	101 - 105	105	L-->M
2P19A	101 - 105	105	L-->P
2P19A	101 - 105	105	L-->S
2P19A	101 - 105	105	L-->Y
1OTGA	120 - 124	120	N-->A
1OTGA	120 - 124	120	N-->G
1OTGA	120 - 124	120	N-->P
1OTGA	120 - 124	120	N-->T
1OTGA	120 - 124	120	N-->V
1OTGA	120 - 124	120	N-->Y
1OTGA	120 - 124	121	V-->A
1OTGA	120 - 124	121	V-->G
1OTGA	120 - 124	121	V-->L
1OTGA	120 - 124	122	H-->A
1OTGA	120 - 124	122	H-->G
1OTGA	120 - 124	122	H-->I
1OTGA	120 - 124	122	H-->K
1OTGA	120 - 124	122	H-->N
1OTGA	120 - 124	122	H-->P
1OTGA	120 - 124	122	H-->T
1OTGA	120 - 124	122	H-->V

1OTGA	120	124	123	A-->K
1OTGA	120	124	123	A-->V
1OTGA	120	124	124	L-->R
1OTGA	120	124	124	L-->V
1OTGA	120	124	124	L-->Y
1T4WA	308	312	308	R-->K
1T4WA	308	312	308	R-->L
1T4WA	308	312	309	S-->G
1T4WA	308	312	310	H-->E
1T4WA	308	312	310	H-->G
1T4WA	308	312	310	H-->L
1T4WA	308	312	310	H-->P
1T4WA	308	312	310	H-->Q
1T4WA	308	312	310	H-->T
1T4WA	308	312	310	H-->V
1T4WA	308	312	311	T-->V
1T4WA	308	312	312	E-->D
1T4WA	308	312	312	E-->V
1XMXA	142	148	146	L-->V
2G43B	111	116	114	H-->N
2G43B	111	116	115	L-->F
2G43B	111	116	116	S-->L
2QM2B	32	37	34	I-->S
2QM2B	32	37	36	D-->F
2QM2B	32	37	36	D-->K
2QM2B	32	37	36	D-->L
2QM2B	82	86	82	G-->R

2QM2B	82 - 86	82	G-->S
2QM2B	82 - 86	82	G-->V
2QM2B	82 - 86	83	R-->G
2QM2B	82 - 86	83	R-->K
2QM2B	82 - 86	83	R-->P
2QM2B	82 - 86	83	R-->T
2QM2B	82 - 86	84	F-->A
2QM2B	82 - 86	84	F-->C
2QM2B	82 - 86	84	F-->D
2QM2B	82 - 86	84	F-->E
2QM2B	82 - 86	84	F-->K
2QM2B	82 - 86	84	F-->T
2QM2B	82 - 86	84	F-->V
2QM2B	82 - 86	84	F-->Y
2QM2B	82 - 86	85	Y-->D
2QM2B	82 - 86	85	Y-->E
2QM2B	82 - 86	85	Y-->I
2QM2B	82 - 86	85	Y-->L
2QM2B	82 - 86	85	Y-->Q
2QM2B	82 - 86	86	R-->A
2QM2B	82 - 86	86	R-->P
2QM2B	82 - 86	86	R-->Q
2QM2B	82 - 86	86	R-->Y
2QM2B	99 - 104	99	Q-->I
2QM2B	106 - 111	106	V-->K
2QM2B	106 - 111	107	T-->E
2QM2B	106 - 111	109	W-->K

2QM2B	106 - 111	109	W-->S
2QM2B	106 - 111	110	E-->R
2QM2B	106 - 111	111	G-->L
1VR4A	29 - 33	29	A-->C
1VR4A	29 - 33	29	A-->D
1VR4A	29 - 33	29	A-->G
1VR4A	29 - 33	29	A-->I
1VR4A	29 - 33	29	A-->K
1VR4A	29 - 33	29	A-->L
1VR4A	29 - 33	29	A-->N
1VR4A	29 - 33	29	A-->P
1VR4A	29 - 33	30	N-->E
1VR4A	29 - 33	30	N-->G
1VR4A	29 - 33	30	N-->K
1VR4A	29 - 33	30	N-->T
1VR4A	29 - 33	30	N-->V
1VR4A	29 - 33	31	I-->F
1VR4A	29 - 33	31	I-->G
1VR4A	29 - 33	31	I-->H
1VR4A	29 - 33	31	I-->L
1VR4A	29 - 33	31	I-->P
1VR4A	29 - 33	31	I-->S
1VR4A	29 - 33	31	I-->T
1VR4A	29 - 33	31	I-->Y
1VR4A	29 - 33	32	V-->D
1VR4A	29 - 33	32	V-->E
1VR4A	29 - 33	33	R-->A

1VR4A	29 - 33	33	R-->E
1VR4A	29 - 33	33	R-->G
1VR4A	29 - 33	33	R-->H
1VR4A	29 - 33	33	R-->I
1VR4A	29 - 33	33	R-->K
1VR4A	29 - 33	33	R-->N
1VR4A	29 - 33	33	R-->P
1VR4A	29 - 33	33	R-->T
1VR4A	29 - 33	33	R-->V
2GF4A	32 - 37	34	Y-->N
2GF4A	32 - 37	36	E-->F
2GF4A	67 - 71	67	S-->D
2GF4A	67 - 71	67	S-->H
2GF4A	67 - 71	67	S-->I
2GF4A	67 - 71	67	S-->L
2GF4A	67 - 71	67	S-->P
2GF4A	67 - 71	67	S-->R
2GF4A	67 - 71	67	S-->T
2GF4A	67 - 71	68	E-->D
2GF4A	67 - 71	68	E-->F
2GF4A	67 - 71	68	E-->V
2GF4A	67 - 71	69	D-->G
2GF4A	67 - 71	69	D-->H
2GF4A	67 - 71	69	D-->K
2GF4A	67 - 71	69	D-->L
2GF4A	67 - 71	69	D-->V
2GF4A	67 - 71	70	E-->A

2GF4A	67 - 71	70	E-->C
2GF4A	67 - 71	70	E-->G
2GF4A	67 - 71	70	E-->H
2GF4A	67 - 71	70	E-->T
2GF4A	67 - 71	70	E-->V
2GF4A	67 - 71	71	F-->C
2GF4A	67 - 71	71	F-->E
2GF4A	67 - 71	71	F-->G
2GF4A	67 - 71	71	F-->L
2GF4A	67 - 71	71	F-->N
2GF4A	67 - 71	71	F-->P
2GF4A	67 - 71	71	F-->S
2GF4A	67 - 71	71	F-->V
2GF4A	67 - 71	71	F-->Y
2GF4A	82 - 87	83	E-->L
2GF4A	82 - 87	86	D-->K
1E9MA	23 - 28	23	V-->W
1E9MA	23 - 28	24	M-->D
1E9MA	23 - 28	28	R-->G
1E9MA	23 - 28	28	R-->N
2A6QA	15 - 20	15	Y-->A
2A6QA	15 - 20	16	S-->D
2OFWE	173 - 178	177	A-->N
2OFWE	173 - 178	178	R-->G
2OFWE	173 - 178	178	R-->L
2OVIA	29 - 34	29	L-->S
2OVIA	29 - 34	31	E-->L

2OVIA	29 - 34	33	V-->K
2OVIA	29 - 34	34	R-->E
2OVIA	29 - 34	34	R-->S
1LW5D	61 - 66	62	N-->D
1LW5D	61 - 66	63	Q-->L
1LW5D	61 - 66	63	Q-->S
1LW5D	61 - 66	64	V-->A
1LW5D	61 - 66	65	S-->R
1LW5D	61 - 66	65	S-->T
1LW5D	183 - 187	183	V-->C
1LW5D	183 - 187	183	V-->F
1LW5D	183 - 187	183	V-->G
1LW5D	183 - 187	183	V-->K
1LW5D	183 - 187	183	V-->R
1LW5D	183 - 187	183	V-->T
1LW5D	183 - 187	184	K-->C
1LW5D	183 - 187	184	K-->F
1LW5D	183 - 187	184	K-->G
1LW5D	183 - 187	184	K-->L
1LW5D	183 - 187	184	K-->M
1LW5D	183 - 187	184	K-->P
1LW5D	183 - 187	184	K-->R
1LW5D	183 - 187	184	K-->S
1LW5D	183 - 187	185	E-->F
1LW5D	183 - 187	185	E-->G
1LW5D	183 - 187	185	E-->L
1LW5D	183 - 187	185	E-->R

1LW5D	183 - 187	185	E-->T
1LW5D	183 - 187	185	E-->V
1LW5D	183 - 187	186	Y-->A
1LW5D	183 - 187	186	Y-->D
1LW5D	183 - 187	186	Y-->E
1LW5D	183 - 187	186	Y-->F
1LW5D	183 - 187	186	Y-->G
1LW5D	183 - 187	186	Y-->I
1LW5D	183 - 187	186	Y-->L
1LW5D	183 - 187	186	Y-->P
1LW5D	183 - 187	186	Y-->S
1LW5D	183 - 187	186	Y-->V
1LW5D	183 - 187	187	A-->V
3BJQJ	212 - 216	212	L-->E
3BJQJ	212 - 216	213	K-->F
3BJQJ	212 - 216	214	A-->E
3BJQJ	212 - 216	214	A-->T
3BJQJ	212 - 216	216	W-->D
3BJQJ	212 - 216	216	W-->F
3BJQJ	212 - 216	216	W-->G
3BJQJ	212 - 216	216	W-->H
3BJQJ	212 - 216	216	W-->L
3BJQJ	212 - 216	216	W-->N
3BJQJ	212 - 216	216	W-->P
3BJQJ	212 - 216	216	W-->R
3BJQJ	212 - 216	216	W-->S
3BJQJ	212 - 216	216	W-->V

2P0LA	125 - 131	129	R-->M
2P0LA	186 - 191	188	G-->D
2P0LA	231 - 237	233	D-->V
2EPMX	232 - 237	234	L-->Y
1FE8C	941 - 946	943	Y-->T
1FQJC	69 - 73	70	W-->F
1FQJC	69 - 73	70	W-->G
1FQJC	69 - 73	70	W-->K
1FQJC	69 - 73	70	W-->L
1FQJC	69 - 73	70	W-->P
1FQJC	69 - 73	70	W-->V
1FQJC	69 - 73	71	E-->L
1FQJC	69 - 73	71	E-->V
1FQJC	69 - 73	71	E-->Y
1FQJC	69 - 73	72	A-->I
1FQJC	69 - 73	73	F-->G
1FQJC	69 - 73	73	F-->I
1FQJC	69 - 73	73	F-->S
1FQJC	69 - 73	73	F-->V
1FQJC	78 - 83	80	E-->G
2H1QA	3 - 7	3	E-->A
2H1QA	3 - 7	3	E-->D
2H1QA	3 - 7	3	E-->K
2H1QA	3 - 7	3	E-->L
2H1QA	3 - 7	3	E-->P
2H1QA	3 - 7	3	E-->R
2H1QA	3 - 7	3	E-->V

2H1QA	3	7	4	I-->A
2H1QA	3	7	4	I-->P
2H1QA	3	7	4	I-->S
2H1QA	3	7	5	Y-->A
2H1QA	3	7	5	Y-->G
2H1QA	3	7	5	Y-->K
2H1QA	3	7	5	Y-->L
2H1QA	3	7	5	Y-->M
2H1QA	3	7	5	Y-->P
2H1QA	3	7	5	Y-->R
2H1QA	3	7	5	Y-->S
2H1QA	3	7	5	Y-->T
2H1QA	3	7	6	D-->G
2H1QA	3	7	7	A-->C
2H1QA	3	7	7	A-->E
2H1QA	3	7	7	A-->F
2H1QA	3	7	7	A-->G
2H1QA	3	7	7	A-->L
2H1QA	3	7	7	A-->T
2H1QA	3	7	7	A-->V
2H1QA	51	55	51	L-->D
2H1QA	51	55	51	L-->I
2H1QA	51	55	51	L-->K
2H1QA	51	55	51	L-->P
2H1QA	51	55	51	L-->V
2H1QA	51	55	52	T-->A
2H1QA	51	55	52	T-->D

2H1QA	51 - 55	52	T-->G
2H1QA	51 - 55	52	T-->N
2H1QA	51 - 55	52	T-->P
2H1QA	51 - 55	52	T-->S
2H1QA	51 - 55	53	Q-->D
2H1QA	51 - 55	53	Q-->G
2H1QA	51 - 55	53	Q-->H
2H1QA	51 - 55	53	Q-->L
2H1QA	51 - 55	53	Q-->N
2H1QA	51 - 55	53	Q-->S
2H1QA	51 - 55	53	Q-->T
2H1QA	51 - 55	53	Q-->W
2H1QA	51 - 55	53	Q-->Y
2H1QA	51 - 55	54	N-->A
2H1QA	51 - 55	54	N-->H
2H1QA	51 - 55	54	N-->K
2H1QA	51 - 55	54	N-->R
2H1QA	89 - 94	92	A-->D
2H1QA	89 - 94	92	A-->S
2H1QA	89 - 94	94	E-->V
2H1QA	134 - 138	134	L-->A
2H1QA	134 - 138	134	L-->D
2H1QA	134 - 138	134	L-->G
2H1QA	134 - 138	134	L-->I
2H1QA	134 - 138	134	L-->K
2H1QA	134 - 138	134	L-->N
2H1QA	134 - 138	134	L-->S

2H1QA	134 - 138	135	E-->C
2H1QA	134 - 138	135	E-->D
2H1QA	134 - 138	135	E-->G
2H1QA	134 - 138	135	E-->I
2H1QA	134 - 138	135	E-->K
2H1QA	134 - 138	135	E-->L
2H1QA	134 - 138	135	E-->N
2H1QA	134 - 138	135	E-->Q
2H1QA	134 - 138	135	E-->R
2H1QA	134 - 138	135	E-->V
2H1QA	134 - 138	135	E-->W
2H1QA	134 - 138	135	E-->Y
2H1QA	134 - 138	136	S-->A
2H1QA	134 - 138	136	S-->D
2H1QA	134 - 138	136	S-->G
2H1QA	134 - 138	136	S-->I
2H1QA	134 - 138	136	S-->K
2H1QA	134 - 138	136	S-->L
2H1QA	134 - 138	136	S-->M
2H1QA	134 - 138	136	S-->N
2H1QA	134 - 138	136	S-->P
2H1QA	134 - 138	136	S-->R
2H1QA	134 - 138	136	S-->V
2H1QA	134 - 138	137	L-->C
2H1QA	134 - 138	137	L-->D
2H1QA	134 - 138	137	L-->G
2H1QA	134 - 138	137	L-->H

2H1QA	134	138	137	L-->K
2H1QA	134	138	137	L-->P
2H1QA	134	138	137	L-->S
2H1QA	134	138	137	L-->T
2H1QA	134	138	137	L-->V
2H1QA	134	138	138	L-->A
2H1QA	134	138	138	L-->D
2H1QA	134	138	138	L-->E
2H1QA	134	138	138	L-->I
2H1QA	134	138	138	L-->K
2H1QA	134	138	138	L-->S
2H1QA	134	138	138	L-->T
2H1QA	134	138	138	L-->V
2H1QA	134	138	138	L-->W
2H1QA	134	138	138	L-->Y
2H1QA	176	180	176	A-->E
2H1QA	176	180	176	A-->F
2H1QA	176	180	176	A-->G
2H1QA	176	180	176	A-->I
2H1QA	176	180	176	A-->L
2H1QA	176	180	176	A-->P
2H1QA	176	180	176	A-->S
2H1QA	176	180	176	A-->V
2H1QA	176	180	176	A-->W
2H1QA	176	180	176	A-->Y
2H1QA	176	180	177	S-->A
2H1QA	176	180	177	S-->C

2H1QA	176	180	177	S-->D
2H1QA	176	180	177	S-->E
2H1QA	176	180	177	S-->F
2H1QA	176	180	177	S-->G
2H1QA	176	180	177	S-->I
2H1QA	176	180	177	S-->K
2H1QA	176	180	177	S-->L
2H1QA	176	180	177	S-->M
2H1QA	176	180	177	S-->N
2H1QA	176	180	177	S-->P
2H1QA	176	180	177	S-->Q
2H1QA	176	180	177	S-->T
2H1QA	176	180	177	S-->V
2H1QA	176	180	178	V-->A
2H1QA	176	180	178	V-->D
2H1QA	176	180	178	V-->E
2H1QA	176	180	178	V-->F
2H1QA	176	180	178	V-->G
2H1QA	176	180	178	V-->K
2H1QA	176	180	178	V-->L
2H1QA	176	180	178	V-->Y
2H1QA	176	180	179	V-->C
2H1QA	176	180	179	V-->D
2H1QA	176	180	179	V-->E
2H1QA	176	180	179	V-->G
2H1QA	176	180	179	V-->I
2H1QA	176	180	179	V-->L

2H1QA	176	180	179	V-->N
2H1QA	176	180	179	V-->P
2H1QA	176	180	179	V-->Q
2H1QA	176	180	179	V-->S
2H1QA	176	180	179	V-->T
2H1QA	176	180	180	D-->A
2H1QA	176	180	180	D-->C
2H1QA	176	180	180	D-->E
2H1QA	176	180	180	D-->F
2H1QA	176	180	180	D-->G
2H1QA	176	180	180	D-->H
2H1QA	176	180	180	D-->K
2H1QA	176	180	180	D-->L
2H1QA	176	180	180	D-->N
2H1QA	176	180	180	D-->S
2H1QA	176	180	180	D-->T
2H1QA	176	180	180	D-->V
2H1QA	176	180	180	D-->W
2H1QA	183	189	183	L-->S
2H1QA	183	189	184	P-->E
2H1QA	183	189	184	P-->Y
2H1QA	183	189	185	R-->G
2H1QA	183	189	187	L-->Q
2H1QA	183	189	188	E-->V
2H1QA	207	211	207	P-->A
2H1QA	207	211	207	P-->D
2H1QA	207	211	207	P-->E

2H1QA	207 - 211	207	P-->G
2H1QA	207 - 211	207	P-->L
2H1QA	207 - 211	207	P-->T
2H1QA	207 - 211	207	P-->V
2H1QA	207 - 211	207	P-->W
2H1QA	207 - 211	207	P-->Y
2H1QA	207 - 211	208	V-->D
2H1QA	207 - 211	208	V-->E
2H1QA	207 - 211	208	V-->H
2H1QA	207 - 211	208	V-->K
2H1QA	207 - 211	208	V-->L
2H1QA	207 - 211	208	V-->M
2H1QA	207 - 211	208	V-->N
2H1QA	207 - 211	209	L-->D
2H1QA	207 - 211	209	L-->F
2H1QA	207 - 211	209	L-->I
2H1QA	207 - 211	209	L-->N
2H1QA	207 - 211	209	L-->Q
2H1QA	207 - 211	209	L-->S
2H1QA	207 - 211	209	L-->V
2H1QA	207 - 211	209	L-->Y
2H1QA	207 - 211	210	F-->A
2H1QA	207 - 211	210	F-->D
2H1QA	207 - 211	210	F-->E
2H1QA	207 - 211	210	F-->G
2H1QA	207 - 211	210	F-->I
2H1QA	207 - 211	210	F-->K

2H1QA	207 - 211	210	F-->L
2H1QA	207 - 211	210	F-->N
2H1QA	207 - 211	210	F-->P
2H1QA	207 - 211	210	F-->R
2H1QA	207 - 211	210	F-->V
2H1QA	207 - 211	211	E-->C
2H1QA	207 - 211	211	E-->F
2H1QA	207 - 211	211	E-->G
2H1QA	207 - 211	211	E-->I
2H1QA	207 - 211	211	E-->L
2H1QA	207 - 211	211	E-->M
2H1QA	207 - 211	211	E-->N
2H1QA	207 - 211	211	E-->P
2H1QA	207 - 211	211	E-->Q
2H1QA	207 - 211	211	E-->S
2H1QA	207 - 211	211	E-->T
2H1QA	207 - 211	211	E-->V
2DR3A	78 - 83	81	E-->R
2DR3A	78 - 83	82	E-->T
2DR3A	78 - 83	82	E-->V
2DR3A	78 - 83	83	K-->V
1SFUA	32 - 38	38	R-->T
1ZNOB	117 - 121	117	P-->A
1ZNOB	117 - 121	117	P-->D
1ZNOB	117 - 121	117	P-->E
1ZNOB	117 - 121	117	P-->I
1ZNOB	117 - 121	117	P-->L

1ZNOB	117	121	117	P-->M
1ZNOB	117	121	117	P-->R
1ZNOB	117	121	117	P-->S
1ZNOB	117	121	117	P-->T
1ZNOB	117	121	117	P-->V
1ZNOB	117	121	117	P-->W
1ZNOB	117	121	118	L-->A
1ZNOB	117	121	118	L-->C
1ZNOB	117	121	118	L-->D
1ZNOB	117	121	118	L-->E
1ZNOB	117	121	118	L-->G
1ZNOB	117	121	118	L-->H
1ZNOB	117	121	118	L-->I
1ZNOB	117	121	118	L-->N
1ZNOB	117	121	118	L-->P
1ZNOB	117	121	118	L-->Q
1ZNOB	117	121	118	L-->R
1ZNOB	117	121	118	L-->S
1ZNOB	117	121	118	L-->V
1ZNOB	117	121	119	V-->D
1ZNOB	117	121	119	V-->H
1ZNOB	117	121	119	V-->I
1ZNOB	117	121	119	V-->N
1ZNOB	117	121	119	V-->P
1ZNOB	117	121	119	V-->Q
1ZNOB	117	121	119	V-->R
1ZNOB	117	121	119	V-->S

1ZNOB	117	-	121	119	V-->T
1ZNOB	117	-	121	119	V-->W
1ZNOB	117	-	121	120	L-->A
1ZNOB	117	-	121	120	L-->C
1ZNOB	117	-	121	120	L-->F
1ZNOB	117	-	121	120	L-->G
1ZNOB	117	-	121	120	L-->I
1ZNOB	117	-	121	120	L-->K
1ZNOB	117	-	121	120	L-->N
1ZNOB	117	-	121	120	L-->P
1ZNOB	117	-	121	120	L-->R
1ZNOB	117	-	121	120	L-->S
1ZNOB	117	-	121	120	L-->T
1ZNOB	117	-	121	120	L-->V
1ZNOB	117	-	121	120	L-->Y
1ZNOB	117	-	121	121	E-->A
1ZNOB	117	-	121	121	E-->C
1ZNOB	117	-	121	121	E-->D
1ZNOB	117	-	121	121	E-->G
1ZNOB	117	-	121	121	E-->H
1ZNOB	117	-	121	121	E-->I
1ZNOB	117	-	121	121	E-->K
1ZNOB	117	-	121	121	E-->L
1ZNOB	117	-	121	121	E-->M
1ZNOB	117	-	121	121	E-->N
1ZNOB	117	-	121	121	E-->P
1ZNOB	117	-	121	121	E-->Q

1ZNOB	117 - 121	121	E-->S
1ZNOB	117 - 121	121	E-->V
1ZNOB	117 - 121	121	E-->W
1ZNOB	147 - 152	148	A-->G
1ZNOB	147 - 152	148	A-->L
1ZNOB	147 - 152	148	A-->V
1ZNOB	147 - 152	149	I-->H
1ZNOB	147 - 152	149	I-->R
1ZNOB	147 - 152	150	G-->R
1ZNOB	147 - 152	150	G-->Y
1ZNOB	147 - 152	151	L-->M
1ZNOB	147 - 152	151	L-->P
1ZNOB	147 - 152	151	L-->R
1ZNOB	147 - 152	152	L-->E
3B46A	188 - 192	188	F-->G
3B46A	188 - 192	188	F-->R
3B46A	188 - 192	188	F-->T
3B46A	188 - 192	189	E-->G
3B46A	188 - 192	189	E-->V
3B46A	188 - 192	190	Q-->D
3B46A	188 - 192	190	Q-->F
3B46A	188 - 192	190	Q-->G
3B46A	188 - 192	190	Q-->I
3B46A	188 - 192	190	Q-->K
3B46A	188 - 192	190	Q-->R
3B46A	188 - 192	190	Q-->T
3B46A	188 - 192	190	Q-->V

3B46A	188 - 192	191	F-->K
3B46A	188 - 192	191	F-->M
3B46A	188 - 192	191	F-->Y
3B46A	188 - 192	192	E-->A
1ROWA	88 - 92	88	A-->D
1ROWA	88 - 92	88	A-->E
1ROWA	88 - 92	88	A-->G
1ROWA	88 - 92	88	A-->I
1ROWA	88 - 92	88	A-->K
1ROWA	88 - 92	88	A-->T
1ROWA	88 - 92	89	Q-->A
1ROWA	88 - 92	89	Q-->D
1ROWA	88 - 92	89	Q-->F
1ROWA	88 - 92	89	Q-->G
1ROWA	88 - 92	89	Q-->I
1ROWA	88 - 92	89	Q-->L
1ROWA	88 - 92	89	Q-->V
1ROWA	88 - 92	89	Q-->Y
1ROWA	88 - 92	90	A-->F
1ROWA	88 - 92	90	A-->P
1ROWA	88 - 92	91	A-->G
1ROWA	88 - 92	91	A-->I
1ROWA	88 - 92	91	A-->T
1ROWA	88 - 92	92	F-->A
1ROWA	88 - 92	92	F-->G
1ROWA	88 - 92	92	F-->I
1ROWA	88 - 92	92	F-->K

1ROWA	88 - 92	92	F-->L
1ROWA	88 - 92	92	F-->N
1ROWA	88 - 92	92	F-->P
1DTJB	14 - 19	14	E-->K
1DTJB	14 - 19	15	N-->E
1DTJB	14 - 19	15	N-->G
1DTJB	14 - 19	18	G-->R
1DTJB	14 - 19	19	A-->D
3BRNB	15 - 20	16	Y-->D
3BRNB	15 - 20	18	S-->L
2YQYB	10 - 14	10	W-->A
2YQYB	10 - 14	10	W-->E
2YQYB	10 - 14	10	W-->G
2YQYB	10 - 14	10	W-->I
2YQYB	10 - 14	10	W-->P
2YQYB	10 - 14	10	W-->R
2YQYB	10 - 14	10	W-->S
2YQYB	10 - 14	10	W-->V
2YQYB	10 - 14	11	D-->G
2YQYB	10 - 14	11	D-->K
2YQYB	10 - 14	11	D-->V
2YQYB	10 - 14	12	E-->L
2YQYB	10 - 14	12	E-->R
2YQYB	10 - 14	12	E-->S
2YQYB	10 - 14	13	A-->E
2YQYB	10 - 14	13	A-->K
2YQYB	10 - 14	13	A-->R

2YQYB	10 - 14	14	L-->A
2YQYB	10 - 14	14	L-->K
2A8IA	294 - 299	296	H-->N
2A8IA	294 - 299	296	H-->S
2A8IA	294 - 299	296	H-->V
2A8IA	294 - 299	297	L-->Y
1OA9A	134 - 138	134	C-->D
1OA9A	134 - 138	134	C-->L
1OA9A	134 - 138	134	C-->P
1OA9A	134 - 138	134	C-->W
1OA9A	134 - 138	135	T-->L
1OA9A	134 - 138	135	T-->N
1OA9A	134 - 138	138	F-->C
1OA9A	152 - 157	152	R-->Y
3BS5A	64 - 69	65	S-->V
3BS5A	64 - 69	67	L-->G
3BS5A	64 - 69	67	L-->N
3BS5A	64 - 69	68	Q-->D
3BS5A	64 - 69	68	Q-->T
3BS5A	64 - 69	69	R-->D
3BS5A	64 - 69	69	R-->Q
2NOGA	792 - 796	792	S-->A
2NOGA	792 - 796	792	S-->G
2NOGA	792 - 796	792	S-->H
2NOGA	792 - 796	793	A-->L
2NOGA	792 - 796	794	Q-->G
2NOGA	792 - 796	794	Q-->L

2NOGA	792 - 796	794	Q-->S
2NOGA	792 - 796	794	Q-->T
2NOGA	792 - 796	794	Q-->V
2NOGA	792 - 796	794	Q-->Y
2NOGA	792 - 796	795	V-->A
2NOGA	792 - 796	795	V-->K
2NOGA	792 - 796	795	V-->P
2NOGA	792 - 796	795	V-->R
2NOGA	792 - 796	795	V-->S
2NOGA	792 - 796	795	V-->Y
2NOGA	792 - 796	796	Q-->H
2NOGA	792 - 796	796	Q-->I
2NOGA	792 - 796	796	Q-->L
2NOGA	792 - 796	796	Q-->R
2NOGA	792 - 796	796	Q-->T
2NOGA	792 - 796	796	Q-->V
2NOGA	792 - 796	796	Q-->Y
2NOGA	849 - 855	852	I-->A
2FQZA	42 - 47	45	F-->K
2FQZA	166 - 171	169	Y-->S
1DFAA	161 - 166	161	A-->G
1DFAA	161 - 166	162	R-->I
1DFAA	161 - 166	162	R-->N
1DFAA	161 - 166	165	S-->I
1DFAA	161 - 166	165	S-->R
1DFAA	161 - 166	165	S-->T
1DFAA	169 - 174	173	K-->H

1DFAA	287 - 292	289	A-->E	
1PN9A	41 - 46	42	E-->R	
1PN9A	41 - 46	46	L-->A	
1MZGB	94 - 98	94	P-->A	
1MZGB	94 - 98	94	P-->E	
1MZGB	94 - 98	94	P-->G	
1MZGB	94 - 98	94	P-->I	
1MZGB	94 - 98	94	P-->K	
1MZGB	94 - 98	94	P-->R	
1MZGB	94 - 98	95	Q-->A	
1MZGB	94 - 98	95	Q-->D	
1MZGB	94 - 98	95	Q-->E	
1MZGB	94 - 98	95	Q-->F	
1MZGB	94 - 98	95	Q-->G	
1MZGB	94 - 98	95	Q-->I	
1MZGB	94 - 98	95	Q-->L	
1MZGB	94 - 98	95	Q-->R	
1MZGB	94 - 98	95	Q-->S	
1MZGB	94 - 98	95	Q-->T	
1MZGB	94 - 98	96	D-->A	
1MZGB	94 - 98	96	D-->F	
1MZGB	94 - 98	96	D-->I	
1MZGB	94 - 98	96	D-->L	
1MZGB	94 - 98	96	D-->P	
1MZGB	94 - 98	96	D-->R	
1MZGB	94 - 98	97	I-->A	
1MZGB	94 - 98	97	I-->D	

1MZGB	94 - 98	97	I-->G
1MZGB	94 - 98	97	I-->K
1MZGB	94 - 98	97	I-->P
1MZGB	94 - 98	98	V-->M
1MZGB	94 - 98	98	V-->P
1MZGB	94 - 98	98	V-->T
1MZGB	103 - 108	105	W-->L
1I7WD	706 - 710	706	D-->I
1I7WD	706 - 710	706	D-->L
1I7WD	706 - 710	706	D-->P
1I7WD	706 - 710	706	D-->Q
1I7WD	706 - 710	706	D-->R
1I7WD	706 - 710	706	D-->S
1I7WD	706 - 710	706	D-->Y
1I7WD	706 - 710	707	Y-->A
1I7WD	706 - 710	707	Y-->D
1I7WD	706 - 710	707	Y-->G
1I7WD	706 - 710	707	Y-->S
1I7WD	706 - 710	707	Y-->T
1I7WD	706 - 710	707	Y-->V
1I7WD	706 - 710	708	L-->E
1I7WD	706 - 710	708	L-->I
1I7WD	706 - 710	708	L-->S
1I7WD	706 - 710	708	L-->V
1I7WD	706 - 710	709	N-->A
1I7WD	706 - 710	709	N-->D
1I7WD	706 - 710	709	N-->G

1I7WD	706	710	709	N-->I
1I7WD	706	710	709	N-->Q
1I7WD	706	710	709	N-->T
1I7WD	706	710	709	N-->Y
1I7WD	706	710	710	E-->A
1I7WD	706	710	710	E-->G
1I7WD	706	710	710	E-->L
1I7WD	706	710	710	E-->R
1I7WD	706	710	710	E-->S
1I7WD	706	710	710	E-->T
2UZ8A	65	69	65	A-->D
2UZ8A	65	69	65	A-->G
2UZ8A	65	69	65	A-->H
2UZ8A	65	69	65	A-->L
2UZ8A	65	69	65	A-->M
2UZ8A	65	69	65	A-->P
2UZ8A	65	69	65	A-->Q
2UZ8A	65	69	65	A-->R
2UZ8A	65	69	65	A-->T
2UZ8A	65	69	65	A-->V
2UZ8A	65	69	66	E-->D
2UZ8A	65	69	66	E-->F
2UZ8A	65	69	66	E-->G
2UZ8A	65	69	66	E-->H
2UZ8A	65	69	66	E-->L
2UZ8A	65	69	66	E-->P
2UZ8A	65	69	66	E-->Q

2UZ8A	65 - 69	66	E-->V
2UZ8A	65 - 69	66	E-->Y
2UZ8A	65 - 69	67	E-->F
2UZ8A	65 - 69	67	E-->G
2UZ8A	65 - 69	67	E-->N
2UZ8A	65 - 69	67	E-->Q
2UZ8A	65 - 69	67	E-->T
2UZ8A	65 - 69	68	K-->A
2UZ8A	65 - 69	68	K-->E
2UZ8A	65 - 69	68	K-->F
2UZ8A	65 - 69	68	K-->I
2UZ8A	65 - 69	68	K-->L
2UZ8A	65 - 69	68	K-->N
2UZ8A	65 - 69	68	K-->P
2UZ8A	65 - 69	68	K-->V
2UZ8A	65 - 69	69	A-->D
2UZ8A	65 - 69	69	A-->E
2UZ8A	65 - 69	69	A-->I
2UZ8A	65 - 69	69	A-->N
2UZ8A	65 - 69	69	A-->P
2UZ8A	65 - 69	69	A-->Q
2UZ8A	65 - 69	69	A-->R
2UZ8A	65 - 69	69	A-->S
2UZ8A	65 - 69	69	A-->V
2UZ8A	90 - 95	91	I-->A
2UZ8A	90 - 95	92	H-->A
2UZ8A	90 - 95	92	H-->L

2UZ8A	90 - 95	92	H-->R
2UZ8A	133 - 138	133	V-->P
2UZ8A	133 - 138	134	Q-->H
2UZ8A	133 - 138	135	E-->G
2UZ8A	133 - 138	135	E-->N
2UZ8A	133 - 138	136	K-->L
2UZ8A	133 - 138	137	E-->S
2UZ8A	133 - 138	137	E-->Y
1YD6A	88 - 92	88	K-->D
1YD6A	88 - 92	88	K-->Q
1YD6A	88 - 92	89	Y-->A
1YD6A	88 - 92	89	Y-->E
1YD6A	88 - 92	89	Y-->G
1YD6A	88 - 92	89	Y-->L
1YD6A	88 - 92	89	Y-->T
1YD6A	88 - 92	89	Y-->W
1YD6A	88 - 92	90	N-->M
1YD6A	88 - 92	90	N-->Q
1YD6A	88 - 92	91	V-->I
1YD6A	88 - 92	91	V-->R
1YD6A	88 - 92	92	M-->A
1YD6A	88 - 92	92	M-->K
1YD6A	88 - 92	92	M-->S
1YD6A	88 - 92	92	M-->T
1BYSA	114 - 118	114	K-->A
1BYSA	114 - 118	114	K-->D
1BYSA	114 - 118	114	K-->E

1BYSA	114 - 118	114	K-->F
1BYSA	114 - 118	114	K-->L
1BYSA	114 - 118	114	K-->N
1BYSA	114 - 118	114	K-->T
1BYSA	114 - 118	114	K-->Y
1BYSA	114 - 118	115	A-->E
1BYSA	114 - 118	115	A-->F
1BYSA	114 - 118	115	A-->L
1BYSA	114 - 118	115	A-->R
1BYSA	114 - 118	115	A-->T
1BYSA	114 - 118	116	A-->E
1BYSA	114 - 118	116	A-->F
1BYSA	114 - 118	116	A-->G
1BYSA	114 - 118	116	A-->V
1BYSA	114 - 118	117	E-->D
1BYSA	114 - 118	117	E-->G
1BYSA	114 - 118	117	E-->I
1BYSA	114 - 118	117	E-->L
1BYSA	114 - 118	117	E-->R
1BYSA	114 - 118	117	E-->V
1BYSA	114 - 118	117	E-->Y
1BYSA	114 - 118	118	T-->E
1BYSA	114 - 118	118	T-->G
1BYSA	114 - 118	118	T-->L
1BYSA	114 - 118	118	T-->W
2ZEJB	1425 - 1429	1425	Q-->A
2ZEJB	1425 - 1429	1425	Q-->D

2ZEJB	1425 - 1429	1425	Q-->E
2ZEJB	1425 - 1429	1425	Q-->G
2ZEJB	1425 - 1429	1425	Q-->H
2ZEJB	1425 - 1429	1425	Q-->K
2ZEJB	1425 - 1429	1425	Q-->L
2ZEJB	1425 - 1429	1425	Q-->M
2ZEJB	1425 - 1429	1425	Q-->N
2ZEJB	1425 - 1429	1425	Q-->P
2ZEJB	1425 - 1429	1425	Q-->R
2ZEJB	1425 - 1429	1425	Q-->S
2ZEJB	1425 - 1429	1425	Q-->W
2ZEJB	1425 - 1429	1425	Q-->Y
2ZEJB	1425 - 1429	1426	A-->G
2ZEJB	1425 - 1429	1426	A-->N
2ZEJB	1425 - 1429	1426	A-->P
2ZEJB	1425 - 1429	1426	A-->S
2ZEJB	1425 - 1429	1426	A-->V
2ZEJB	1425 - 1429	1427	E-->I
2ZEJB	1425 - 1429	1427	E-->K
2ZEJB	1425 - 1429	1427	E-->L
2ZEJB	1425 - 1429	1427	E-->M
2ZEJB	1425 - 1429	1427	E-->P
2ZEJB	1425 - 1429	1427	E-->Q
2ZEJB	1425 - 1429	1427	E-->R
2ZEJB	1425 - 1429	1428	V-->D
2ZEJB	1425 - 1429	1428	V-->E
2ZEJB	1425 - 1429	1428	V-->F

2ZEJB	1425 - 1429	1428	V-->G
2ZEJB	1425 - 1429	1428	V-->H
2ZEJB	1425 - 1429	1428	V-->N
2ZEJB	1425 - 1429	1428	V-->P
2ZEJB	1425 - 1429	1428	V-->S
2ZEJB	1425 - 1429	1428	V-->W
2ZEJB	1425 - 1429	1429	D-->E
2ZEJB	1425 - 1429	1429	D-->G
2ZEJB	1425 - 1429	1429	D-->I
2ZEJB	1425 - 1429	1429	D-->L
2ZEJB	1425 - 1429	1429	D-->N
2ZEJB	1425 - 1429	1429	D-->R
2ZEJB	1425 - 1429	1429	D-->T
2ZEJB	1425 - 1429	1429	D-->V
2ZEJB	1460 - 1465	1463	K-->S
2ZEJB	1467 - 1472	1467	S-->N
2ZEJB	1467 - 1472	1468	K-->Q
2ZEJB	1467 - 1472	1470	T-->D
2ZEJB	1467 - 1472	1471	K-->V
2QQYA	124 - 130	130	H-->L
2HQ7B	58 - 63	59	E-->A
2HQ7B	58 - 63	59	E-->K
2HQ7B	58 - 63	59	E-->V
2HQ7B	58 - 63	60	R-->E
2HQ7B	58 - 63	60	R-->K
2HQ7B	92 - 96	92	R-->F
2HQ7B	92 - 96	92	R-->G

2HQ7B	92 - 96	92	R-->I
2HQ7B	92 - 96	92	R-->K
2HQ7B	92 - 96	92	R-->P
2HQ7B	92 - 96	92	R-->Y
2HQ7B	92 - 96	93	A-->G
2HQ7B	92 - 96	93	A-->P
2HQ7B	92 - 96	93	A-->Q
2HQ7B	92 - 96	93	A-->Y
2HQ7B	92 - 96	94	S-->A
2HQ7B	92 - 96	94	S-->H
2HQ7B	92 - 96	94	S-->K
2HQ7B	92 - 96	94	S-->L
2HQ7B	92 - 96	94	S-->P
2HQ7B	92 - 96	94	S-->Q
2HQ7B	92 - 96	95	K-->C
2HQ7B	92 - 96	95	K-->D
2HQ7B	92 - 96	95	K-->F
2HQ7B	92 - 96	95	K-->G
2HQ7B	92 - 96	95	K-->N
2HQ7B	92 - 96	95	K-->Q
2HQ7B	92 - 96	95	K-->T
2HQ7B	92 - 96	96	E-->K
2HQ7B	92 - 96	96	E-->N
2HQ7B	92 - 96	96	E-->P
2HQ7B	92 - 96	96	E-->Q
2HQ7B	92 - 96	96	E-->S
2HQ7B	92 - 96	96	E-->T

2HQ7B	92 - 96	96	E-->Y
2D28C	22 - 27	24	D-->L
2D28C	22 - 27	25	L-->D
2D28C	22 - 27	26	V-->G
2D28C	22 - 27	27	R-->L
1FVIA	124 - 128	124	P-->H
1FVIA	124 - 128	124	P-->I
1FVIA	124 - 128	124	P-->L
1FVIA	124 - 128	124	P-->S
1FVIA	124 - 128	125	H-->E
1FVIA	124 - 128	125	H-->F
1FVIA	124 - 128	125	H-->K
1FVIA	124 - 128	125	H-->L
1FVIA	124 - 128	125	H-->M
1FVIA	124 - 128	125	H-->N
1FVIA	124 - 128	125	H-->R
1FVIA	124 - 128	125	H-->V
1FVIA	124 - 128	125	H-->Y
1FVIA	124 - 128	126	I-->G
1FVIA	124 - 128	126	I-->H
1FVIA	124 - 128	126	I-->K
1FVIA	124 - 128	126	I-->R
1FVIA	124 - 128	126	I-->V
1FVIA	124 - 128	126	I-->Y
1FVIA	124 - 128	127	L-->P
1FVIA	124 - 128	127	L-->V
1FVIA	124 - 128	127	L-->W

1FVIA	124 - 128	128	E-->I
1FVIA	124 - 128	128	E-->L
1FVIA	124 - 128	128	E-->S
1FVIA	124 - 128	128	E-->T
1JEOA	121 - 125	121	N-->A
1JEOA	121 - 125	121	N-->D
1JEOA	121 - 125	121	N-->E
1JEOA	121 - 125	121	N-->G
1JEOA	121 - 125	121	N-->I
1JEOA	121 - 125	121	N-->K
1JEOA	121 - 125	121	N-->L
1JEOA	121 - 125	121	N-->R
1JEOA	121 - 125	121	N-->S
1JEOA	121 - 125	121	N-->T
1JEOA	121 - 125	121	N-->V
1JEOA	121 - 125	121	N-->Y
1JEOA	121 - 125	122	V-->D
1JEOA	121 - 125	122	V-->G
1JEOA	121 - 125	122	V-->I
1JEOA	121 - 125	122	V-->L
1JEOA	121 - 125	122	V-->N
1JEOA	121 - 125	122	V-->Y
1JEOA	121 - 125	123	V-->I
1JEOA	121 - 125	123	V-->K
1JEOA	121 - 125	123	V-->N
1JEOA	121 - 125	123	V-->P
1JEOA	121 - 125	123	V-->T

1JEOA	121 - 125	124	E-->D
1JEOA	121 - 125	124	E-->F
1JEOA	121 - 125	124	E-->I
1JEOA	121 - 125	124	E-->K
1JEOA	121 - 125	124	E-->N
1JEOA	121 - 125	124	E-->P
1JEOA	121 - 125	124	E-->Q
1JEOA	121 - 125	124	E-->R
1JEOA	121 - 125	124	E-->V
1JEOA	121 - 125	125	F-->A
1JEOA	121 - 125	125	F-->E
1JEOA	121 - 125	125	F-->G
1JEOA	121 - 125	125	F-->I
1JEOA	121 - 125	125	F-->L
1JEOA	121 - 125	125	F-->P
1JEOA	121 - 125	125	F-->Q
1JEOA	121 - 125	125	F-->R
1JEOA	121 - 125	125	F-->S
1JEOA	121 - 125	125	F-->T
1JEOA	121 - 125	125	F-->V
2BW3B	109 - 113	109	F-->A
2BW3B	109 - 113	109	F-->G
2BW3B	109 - 113	109	F-->I
2BW3B	109 - 113	109	F-->P
2BW3B	109 - 113	109	F-->Q
2BW3B	109 - 113	109	F-->S
2BW3B	109 - 113	109	F-->T

2BW3B	109 - 113	109	F-->V
2BW3B	109 - 113	109	F-->Y
2BW3B	109 - 113	110	S-->A
2BW3B	109 - 113	110	S-->C
2BW3B	109 - 113	110	S-->D
2BW3B	109 - 113	110	S-->E
2BW3B	109 - 113	110	S-->F
2BW3B	109 - 113	110	S-->G
2BW3B	109 - 113	110	S-->K
2BW3B	109 - 113	110	S-->L
2BW3B	109 - 113	110	S-->P
2BW3B	109 - 113	110	S-->Q
2BW3B	109 - 113	110	S-->T
2BW3B	109 - 113	110	S-->V
2BW3B	109 - 113	110	S-->Y
2BW3B	109 - 113	111	A-->E
2BW3B	109 - 113	111	A-->H
2BW3B	109 - 113	111	A-->K
2BW3B	109 - 113	111	A-->L
2BW3B	109 - 113	111	A-->Q
2BW3B	109 - 113	111	A-->S
2BW3B	109 - 113	111	A-->V
2BW3B	109 - 113	111	A-->Y
2BW3B	109 - 113	112	V-->A
2BW3B	109 - 113	112	V-->E
2BW3B	109 - 113	112	V-->F
2BW3B	109 - 113	112	V-->H

2BW3B	109 - 113	112	V-->K
2BW3B	109 - 113	112	V-->L
2BW3B	109 - 113	112	V-->P
2BW3B	109 - 113	112	V-->Q
2BW3B	109 - 113	112	V-->R
2BW3B	109 - 113	112	V-->T
2BW3B	109 - 113	112	V-->Y
2BW3B	109 - 113	113	S-->E
2BW3B	109 - 113	113	S-->K
2BW3B	109 - 113	113	S-->N
2BW3B	109 - 113	113	S-->Q
2BW3B	109 - 113	113	S-->V
2BW3B	115 - 119	115	S-->D
2BW3B	115 - 119	115	S-->E
2BW3B	115 - 119	115	S-->F
2BW3B	115 - 119	115	S-->H
2BW3B	115 - 119	115	S-->I
2BW3B	115 - 119	115	S-->K
2BW3B	115 - 119	115	S-->P
2BW3B	115 - 119	115	S-->Q
2BW3B	115 - 119	116	G-->K
2BW3B	115 - 119	116	G-->L
2BW3B	115 - 119	116	G-->P
2BW3B	115 - 119	116	G-->S
2BW3B	115 - 119	116	G-->T
2BW3B	115 - 119	116	G-->V
2BW3B	115 - 119	116	G-->W

2BW3B	115 - 119	116	G-->Y
2BW3B	115 - 119	117	F-->A
2BW3B	115 - 119	117	F-->D
2BW3B	115 - 119	117	F-->E
2BW3B	115 - 119	117	F-->G
2BW3B	115 - 119	117	F-->I
2BW3B	115 - 119	117	F-->K
2BW3B	115 - 119	117	F-->M
2BW3B	115 - 119	117	F-->N
2BW3B	115 - 119	117	F-->P
2BW3B	115 - 119	117	F-->Q
2BW3B	115 - 119	117	F-->T
2BW3B	115 - 119	118	I-->E
2BW3B	115 - 119	118	I-->G
2BW3B	115 - 119	118	I-->N
2BW3B	115 - 119	118	I-->P
2BW3B	115 - 119	118	I-->R
2BW3B	115 - 119	118	I-->W
2BW3B	115 - 119	119	D-->A
2BW3B	115 - 119	119	D-->E
2BW3B	115 - 119	119	D-->F
2BW3B	115 - 119	119	D-->H
2BW3B	115 - 119	119	D-->K
2BW3B	115 - 119	119	D-->L
2BW3B	115 - 119	119	D-->P
2BW3B	115 - 119	119	D-->R
2BW3B	115 - 119	119	D-->S

2BW3B	115 - 119	119	D-->V
1MKBA	65 - 69	65	W-->G
1MKBA	65 - 69	65	W-->T
1MKBA	65 - 69	68	G-->H
1MKBA	65 - 69	69	C-->K
1MKBA	65 - 69	69	C-->N
3C26A	12 - 18	13	S-->F
3BDZA	209 - 214	210	M-->P
3BDZA	209 - 214	212	R-->A
3BDZA	209 - 214	212	R-->D
2I9CA	104 - 109	104	Q-->G
2I9CA	104 - 109	104	Q-->L
2I9CA	104 - 109	108	A-->V
1XERA	50 - 54	50	S-->E
1XERA	50 - 54	50	S-->G
1XERA	50 - 54	51	C-->D
1XERA	50 - 54	51	C-->G
1XERA	50 - 54	51	C-->I
1XERA	50 - 54	51	C-->K
1XERA	50 - 54	51	C-->L
1XERA	50 - 54	51	C-->M
1XERA	50 - 54	51	C-->P
1XERA	50 - 54	51	C-->S
1XERA	50 - 54	51	C-->Y
1XERA	50 - 54	52	I-->C
1XERA	50 - 54	52	I-->E
1XERA	50 - 54	53	N-->A

1XERA	50 - 54	53	N-->C	
1XERA	50 - 54	53	N-->M	
1XERA	50 - 54	53	N-->S	
1XERA	50 - 54	53	N-->V	
1XERA	50 - 54	54	A-->D	
1XERA	50 - 54	54	A-->L	
1XERA	50 - 54	54	A-->R	
1XERA	88 - 92	88	A-->N	
1XERA	88 - 92	88	A-->P	
1XERA	88 - 92	88	A-->V	
1XERA	88 - 92	89	C-->A	
1XERA	88 - 92	89	C-->D	
1XERA	88 - 92	89	C-->E	
1XERA	88 - 92	89	C-->F	
1XERA	88 - 92	89	C-->G	
1XERA	88 - 92	89	C-->I	
1XERA	88 - 92	89	C-->K	
1XERA	88 - 92	89	C-->L	
1XERA	88 - 92	89	C-->M	
1XERA	88 - 92	89	C-->N	
1XERA	88 - 92	89	C-->P	
1XERA	88 - 92	89	C-->Q	
1XERA	88 - 92	89	C-->S	
1XERA	88 - 92	89	C-->T	
1XERA	88 - 92	89	C-->V	
1XERA	88 - 92	90	V-->G	
1XERA	88 - 92	90	V-->L	

1XERA	88 - 92	90	V-->T
1XERA	88 - 92	91	N-->A
1XERA	88 - 92	91	N-->C
1XERA	88 - 92	91	N-->H
1XERA	88 - 92	91	N-->L
1XERA	88 - 92	91	N-->S
1XERA	88 - 92	91	N-->V
1XERA	88 - 92	92	V-->E
1XERA	88 - 92	92	V-->G
1XERA	88 - 92	92	V-->I
1XERA	88 - 92	92	V-->L
2FW5A	18 - 23	21	R-->F
2FW5A	18 - 23	22	T-->I
2FW5A	18 - 23	22	T-->V
1HJLA	58 - 62	58	Q-->L
1HJLA	58 - 62	58	Q-->N
1HJLA	58 - 62	58	Q-->T
1HJLA	58 - 62	59	E-->G
1HJLA	58 - 62	60	I-->D
1HJLA	58 - 62	61	Y-->E
1HJLA	58 - 62	61	Y-->P
1HJLA	58 - 62	61	Y-->R
1HJLA	58 - 62	61	Y-->S
1HJLA	58 - 62	61	Y-->V
1HJLA	58 - 62	62	Q-->I
1HJLA	58 - 62	62	Q-->R
1HJLA	58 - 62	58	P-->A

1HJLA	58 - 62	58	P-->E
1HJLA	58 - 62	58	P-->K
1HJLA	58 - 62	58	P-->S
1HJLA	58 - 62	58	P-->V
1HJLA	58 - 62	58	P-->Y
1HJLA	58 - 62	59	E-->G
1HJLA	58 - 62	59	E-->L
1HJLA	58 - 62	59	E-->P
1HJLA	58 - 62	59	E-->R
1HJLA	58 - 62	59	E-->V
1HJLA	58 - 62	59	E-->Y
1HJLA	58 - 62	60	E-->D
1HJLA	58 - 62	60	E-->G
1HJLA	58 - 62	60	E-->I
1HJLA	58 - 62	60	E-->L
1HJLA	58 - 62	60	E-->P
1HJLA	58 - 62	60	E-->Q
1HJLA	58 - 62	60	E-->T
1HJLA	58 - 62	60	E-->V
1HJLA	58 - 62	60	E-->W
1HJLA	58 - 62	60	E-->Y
1HJLA	58 - 62	61	L-->A
1HJLA	58 - 62	61	L-->E
1HJLA	58 - 62	61	L-->K
1HJLA	58 - 62	61	L-->N
1HJLA	58 - 62	61	L-->P
1HJLA	58 - 62	61	L-->R

1HJLA	58 - 62	61	L-->T
1HJLA	58 - 62	62	A-->G
1HJLA	58 - 62	62	A-->H
1HJLA	58 - 62	62	A-->K
1HJLA	58 - 62	62	A-->L
1HJLA	58 - 62	62	A-->M
1HJLA	58 - 62	62	A-->N
1HJLA	58 - 62	62	A-->P
1HJLA	58 - 62	62	A-->Q
1HJLA	58 - 62	62	A-->R
1HJLA	58 - 62	62	A-->S
1HJLA	58 - 62	62	A-->T
1HJLA	58 - 62	62	A-->V
1HJLA	58 - 62	62	A-->Y
2OO3A	65 - 70	66	N-->A
2OO3A	65 - 70	70	L-->N
2FEAA	65 - 69	65	N-->A
2FEAA	65 - 69	65	N-->D
2FEAA	65 - 69	65	N-->E
2FEAA	65 - 69	65	N-->H
2FEAA	65 - 69	65	N-->I
2FEAA	65 - 69	65	N-->K
2FEAA	65 - 69	65	N-->R
2FEAA	65 - 69	65	N-->S
2FEAA	65 - 69	65	N-->W
2FEAA	65 - 69	66	I-->F
2FEAA	65 - 69	66	I-->G

2FEAA	65 - 69	66	I-->N
2FEAA	65 - 69	66	I-->Q
2FEAA	65 - 69	66	I-->S
2FEAA	65 - 69	66	I-->T
2FEAA	65 - 69	67	I-->C
2FEAA	65 - 69	67	I-->E
2FEAA	65 - 69	67	I-->G
2FEAA	65 - 69	67	I-->K
2FEAA	65 - 69	67	I-->L
2FEAA	65 - 69	67	I-->M
2FEAA	65 - 69	67	I-->P
2FEAA	65 - 69	67	I-->S
2FEAA	65 - 69	67	I-->V
2FEAA	65 - 69	68	N-->A
2FEAA	65 - 69	68	N-->E
2FEAA	65 - 69	68	N-->G
2FEAA	65 - 69	68	N-->P
2FEAA	65 - 69	69	I-->D
2FEAA	65 - 69	69	I-->G
2FEAA	65 - 69	69	I-->L
2HQ4A	141 - 146	144	K-->S
3BHSA	245 - 250	245	A-->G
3BHSA	245 - 250	245	A-->I
3BHSA	245 - 250	245	A-->M
3BHSA	245 - 250	246	G-->D
3BHSA	245 - 250	246	G-->E
3BHSA	245 - 250	246	G-->Q

3BHSA	245	- 250	247	G-->D
3BHSA	245	- 250	247	G-->K
3BHSA	245	- 250	248	V-->E
3BHSA	245	- 250	248	V-->S
3BHSA	245	- 250	249	A-->M
3BHSA	245	- 250	249	A-->Q
3BHSA	245	- 250	250	I-->A
3BHSA	245	- 250	250	I-->V
3CNEB	78	- 83	82	Q-->A
3CNEB	88	- 93	89	Y-->R
3CNEB	88	- 93	92	D-->S
3CNEB	160	- 165	164	W-->I
3CNEB	160	- 165	164	W-->R
2D0WA	156	- 161	160	A-->T
2D0WA	156	- 161	160	A-->Y
2D0WA	156	- 161	161	E-->A
2D0WA	156	- 161	161	E-->R
2D0WA	156	- 161	161	E-->W
2BBEA	38	- 42	38	I-->A
2BBEA	38	- 42	38	I-->K
2BBEA	38	- 42	38	I-->N
2BBEA	38	- 42	38	I-->P
2BBEA	38	- 42	38	I-->S
2BBEA	38	- 42	38	I-->V
2BBEA	38	- 42	39	P-->A
2BBEA	38	- 42	39	P-->G
2BBEA	38	- 42	39	P-->L

2BBEA	38 - 42	39	P-->Q
2BBEA	38 - 42	39	P-->R
2BBEA	38 - 42	39	P-->T
2BBEA	38 - 42	39	P-->V
2BBEA	38 - 42	40	D-->A
2BBEA	38 - 42	40	D-->C
2BBEA	38 - 42	40	D-->F
2BBEA	38 - 42	40	D-->G
2BBEA	38 - 42	40	D-->R
2BBEA	38 - 42	40	D-->T
2BBEA	38 - 42	41	T-->F
2BBEA	38 - 42	41	T-->G
2BBEA	38 - 42	41	T-->P
2BBEA	38 - 42	42	R-->A
2BBEA	38 - 42	42	R-->G
2BBEA	38 - 42	42	R-->I
2BBEA	38 - 42	42	R-->L
2BBEA	38 - 42	42	R-->N
2BBEA	38 - 42	42	R-->V
2BBEA	38 - 42	42	R-->W
1LG7A	201 - 206	203	E-->F
1LG7A	201 - 206	206	L-->P
1LG7A	201 - 206	206	L-->Y
2R11C	131 - 136	133	G-->P
2R11C	131 - 136	133	G-->R
2R11C	131 - 136	133	G-->S
2R11C	131 - 136	134	L-->T

2R11C	131 - 136	136	T-->H
2R11C	131 - 136	136	T-->N
2R11C	131 - 136	136	T-->V
2R11C	138 - 142	138	N-->C
2R11C	138 - 142	138	N-->D
2R11C	138 - 142	138	N-->F
2R11C	138 - 142	138	N-->G
2R11C	138 - 142	138	N-->I
2R11C	138 - 142	138	N-->K
2R11C	138 - 142	138	N-->L
2R11C	138 - 142	138	N-->R
2R11C	138 - 142	138	N-->T
2R11C	138 - 142	139	F-->C
2R11C	138 - 142	139	F-->G
2R11C	138 - 142	139	F-->K
2R11C	138 - 142	139	F-->L
2R11C	138 - 142	139	F-->M
2R11C	138 - 142	139	F-->P
2R11C	138 - 142	139	F-->R
2R11C	138 - 142	139	F-->S
2R11C	138 - 142	139	F-->T
2R11C	138 - 142	140	L-->C
2R11C	138 - 142	140	L-->D
2R11C	138 - 142	140	L-->H
2R11C	138 - 142	140	L-->K
2R11C	138 - 142	140	L-->Q
2R11C	138 - 142	140	L-->R

2R11C	138 - 142	141	L-->G
2R11C	138 - 142	141	L-->I
2R11C	138 - 142	141	L-->K
2R11C	138 - 142	141	L-->P
2R11C	138 - 142	141	L-->V
2R11C	138 - 142	142	R-->F
2R11C	138 - 142	142	R-->H
2R11C	138 - 142	142	R-->L
2R11C	138 - 142	142	R-->P
2R11C	138 - 142	142	R-->T
2R11C	227 - 231	227	D-->G
2R11C	227 - 231	227	D-->L
2R11C	227 - 231	227	D-->M
2R11C	227 - 231	227	D-->P
2R11C	227 - 231	227	D-->R
2R11C	227 - 231	227	D-->S
2R11C	227 - 231	227	D-->T
2R11C	227 - 231	228	E-->A
2R11C	227 - 231	228	E-->D
2R11C	227 - 231	228	E-->F
2R11C	227 - 231	228	E-->G
2R11C	227 - 231	228	E-->H
2R11C	227 - 231	228	E-->K
2R11C	227 - 231	228	E-->P
2R11C	227 - 231	228	E-->R
2R11C	227 - 231	228	E-->S
2R11C	227 - 231	228	E-->T

2R11C	227 - 231	228	E-->V
2R11C	227 - 231	229	E-->D
2R11C	227 - 231	229	E-->G
2R11C	227 - 231	229	E-->L
2R11C	227 - 231	229	E-->N
2R11C	227 - 231	229	E-->P
2R11C	227 - 231	230	L-->A
2R11C	227 - 231	230	L-->G
2R11C	227 - 231	230	L-->K
2R11C	227 - 231	230	L-->N
2R11C	227 - 231	230	L-->Y
2R11C	227 - 231	231	R-->D
2R11C	227 - 231	231	R-->E
2R11C	227 - 231	231	R-->G
2R11C	227 - 231	231	R-->K
2R11C	227 - 231	231	R-->N
2R11C	227 - 231	231	R-->P
2R11C	227 - 231	231	R-->S
2R11C	227 - 231	231	R-->T
2R11C	227 - 231	231	R-->V
1NZEA	63 - 67	63	K-->A
1NZEA	63 - 67	63	K-->D
1NZEA	63 - 67	63	K-->E
1NZEA	63 - 67	63	K-->N
1NZEA	63 - 67	64	Q-->G
1NZEA	63 - 67	64	Q-->I
1NZEA	63 - 67	64	Q-->L

1NZEA	63	67	64	Q-->T
1NZEA	63	67	65	F-->A
1NZEA	63	67	65	F-->D
1NZEA	63	67	65	F-->G
1NZEA	63	67	65	F-->K
1NZEA	63	67	65	F-->V
1NZEA	63	67	66	I-->K
1NZEA	63	67	66	I-->P
1NZEA	63	67	67	D-->H
1NZEA	63	67	67	D-->P
2O4TA	63	67	63	V-->A
2O4TA	63	67	63	V-->D
2O4TA	63	67	63	V-->F
2O4TA	63	67	63	V-->G
2O4TA	63	67	63	V-->H
2O4TA	63	67	63	V-->I
2O4TA	63	67	63	V-->K
2O4TA	63	67	63	V-->L
2O4TA	63	67	63	V-->N
2O4TA	63	67	63	V-->Q
2O4TA	63	67	63	V-->R
2O4TA	63	67	63	V-->S
2O4TA	63	67	63	V-->T
2O4TA	63	67	63	V-->Y
2O4TA	63	67	64	L-->A
2O4TA	63	67	64	L-->E
2O4TA	63	67	64	L-->F

204TA	63 - 67	64	L-->G
204TA	63 - 67	64	L-->H
204TA	63 - 67	64	L-->I
204TA	63 - 67	64	L-->K
204TA	63 - 67	64	L-->M
204TA	63 - 67	64	L-->P
204TA	63 - 67	64	L-->Q
204TA	63 - 67	64	L-->S
204TA	63 - 67	64	L-->V
204TA	63 - 67	64	L-->W
204TA	63 - 67	64	L-->Y
204TA	63 - 67	65	D-->C
204TA	63 - 67	65	D-->E
204TA	63 - 67	65	D-->F
204TA	63 - 67	65	D-->G
204TA	63 - 67	65	D-->H
204TA	63 - 67	65	D-->I
204TA	63 - 67	65	D-->L
204TA	63 - 67	65	D-->M
204TA	63 - 67	65	D-->N
204TA	63 - 67	65	D-->P
204TA	63 - 67	65	D-->Q
204TA	63 - 67	65	D-->R
204TA	63 - 67	65	D-->S
204TA	63 - 67	65	D-->T
204TA	63 - 67	65	D-->V
204TA	63 - 67	65	D-->Y

204TA	63 - 67	66	V-->A
204TA	63 - 67	66	V-->C
204TA	63 - 67	66	V-->E
204TA	63 - 67	66	V-->F
204TA	63 - 67	66	V-->G
204TA	63 - 67	66	V-->I
204TA	63 - 67	66	V-->K
204TA	63 - 67	66	V-->N
204TA	63 - 67	66	V-->P
204TA	63 - 67	66	V-->R
204TA	63 - 67	66	V-->S
204TA	63 - 67	66	V-->W
204TA	63 - 67	67	T-->A
204TA	63 - 67	67	T-->D
204TA	63 - 67	67	T-->F
204TA	63 - 67	67	T-->G
204TA	63 - 67	67	T-->K
204TA	63 - 67	67	T-->L
204TA	63 - 67	67	T-->M
204TA	63 - 67	67	T-->N
204TA	63 - 67	67	T-->P
204TA	63 - 67	67	T-->R
204TA	63 - 67	67	T-->S
204TA	63 - 67	67	T-->V
204TA	63 - 67	67	T-->W
204TA	63 - 67	67	T-->Y
204TA	78 - 84	82	C-->I

2OUWA	9 - 13	9	D-->A
2OUWA	9 - 13	9	D-->G
2OUWA	9 - 13	9	D-->H
2OUWA	9 - 13	9	D-->I
2OUWA	9 - 13	9	D-->S
2OUWA	9 - 13	9	D-->V
2OUWA	9 - 13	10	A-->C
2OUWA	9 - 13	10	A-->E
2OUWA	9 - 13	10	A-->G
2OUWA	9 - 13	10	A-->I
2OUWA	9 - 13	10	A-->K
2OUWA	9 - 13	10	A-->M
2OUWA	9 - 13	10	A-->N
2OUWA	9 - 13	10	A-->P
2OUWA	9 - 13	10	A-->S
2OUWA	9 - 13	11	E-->A
2OUWA	9 - 13	11	E-->C
2OUWA	9 - 13	11	E-->G
2OUWA	9 - 13	11	E-->I
2OUWA	9 - 13	11	E-->L
2OUWA	9 - 13	11	E-->P
2OUWA	9 - 13	11	E-->R
2OUWA	9 - 13	11	E-->V
2OUWA	9 - 13	12	I-->A
2OUWA	9 - 13	12	I-->E
2OUWA	9 - 13	12	I-->P
2OUWA	9 - 13	12	I-->V

2OUWA	9 - 13	13	S-->A
2OUWA	9 - 13	13	S-->G
2OUWA	9 - 13	13	S-->I
2OUWA	9 - 13	13	S-->K
2OUWA	9 - 13	13	S-->L
2OUWA	9 - 13	13	S-->P
2OUWA	9 - 13	13	S-->Q
2OUWA	9 - 13	13	S-->R
2OUWA	9 - 13	13	S-->V
2OUWA	9 - 13	13	S-->W
2OUWA	36 - 41	38	W-->T
2OUWA	36 - 40	36	P-->A
2OUWA	36 - 40	36	P-->C
2OUWA	36 - 40	36	P-->E
2OUWA	36 - 40	36	P-->G
2OUWA	36 - 40	36	P-->H
2OUWA	36 - 40	36	P-->K
2OUWA	36 - 40	36	P-->Q
2OUWA	36 - 40	36	P-->R
2OUWA	36 - 40	36	P-->T
2OUWA	36 - 40	37	L-->A
2OUWA	36 - 40	37	L-->C
2OUWA	36 - 40	37	L-->E
2OUWA	36 - 40	37	L-->F
2OUWA	36 - 40	37	L-->G
2OUWA	36 - 40	37	L-->N
2OUWA	36 - 40	37	L-->P

2OUWA	36 - 40	37	L-->R
2OUWA	36 - 40	37	L-->T
2OUWA	36 - 40	38	T-->D
2OUWA	36 - 40	38	T-->G
2OUWA	36 - 40	38	T-->L
2OUWA	36 - 40	38	T-->P
2OUWA	36 - 40	38	T-->S
2OUWA	36 - 40	38	T-->V
2OUWA	36 - 40	38	T-->Y
2OUWA	36 - 40	39	R-->A
2OUWA	36 - 40	39	R-->D
2OUWA	36 - 40	39	R-->E
2OUWA	36 - 40	39	R-->F
2OUWA	36 - 40	39	R-->I
2OUWA	36 - 40	39	R-->L
2OUWA	36 - 40	39	R-->P
2OUWA	36 - 40	39	R-->Q
2OUWA	36 - 40	39	R-->T
2OUWA	36 - 40	39	R-->V
2OUWA	36 - 40	39	R-->Y
2OUWA	36 - 40	40	E-->A
2OUWA	36 - 40	40	E-->G
2OUWA	36 - 40	40	E-->I
2OUWA	36 - 40	40	E-->L
2OUWA	36 - 40	40	E-->M
2OUWA	36 - 40	40	E-->P
2OUWA	36 - 40	40	E-->Q

2OUWA	36 - 40	40	E-->S
2OUWA	36 - 40	40	E-->V
1ZDEA	28 - 33	28	I-->L
1ZDEA	28 - 33	30	K-->V
1KLXA	29 - 34	32	S-->A
1KLXA	129 - 134	131	A-->V
2VGXA	52 - 56	52	Y-->H
2VGXA	52 - 56	52	Y-->M
2VGXA	52 - 56	53	E-->L
2VGXA	52 - 56	53	E-->T
2VGXA	52 - 56	54	D-->F
2VGXA	52 - 56	54	D-->T
2VGXA	52 - 56	55	A-->D
2VGXA	52 - 56	55	A-->E
2VGXA	52 - 56	55	A-->F
2VGXA	52 - 56	56	H-->P
2VGXA	52 - 56	56	H-->S
2VGXA	52 - 56	56	H-->T
1VCHE	52 - 56	52	R-->A
1VCHE	52 - 56	52	R-->L
1VCHE	52 - 56	52	R-->P
1VCHE	52 - 56	52	R-->Q
1VCHE	52 - 56	52	R-->T
1VCHE	52 - 56	52	R-->Y
1VCHE	52 - 56	53	R-->F
1VCHE	52 - 56	53	R-->K
1VCHE	52 - 56	54	F-->E

1VCHE	52 - 56	54	F-->G
1VCHE	52 - 56	54	F-->I
1VCHE	52 - 56	54	F-->R
1VCHE	52 - 56	54	F-->V
1VCHE	52 - 56	55	A-->K
1VCHE	52 - 56	55	A-->Q
1VCHE	52 - 56	55	A-->V
1VCHE	52 - 56	56	E-->A
1VCHE	52 - 56	56	E-->H
1VCHE	52 - 56	56	E-->I
1VCHE	52 - 56	56	E-->L
1VCHE	52 - 56	56	E-->P
1VCHE	52 - 56	56	E-->Q
1VCHE	52 - 56	56	E-->S
2C49B	162 - 167	163	Q-->F
2C49B	162 - 167	163	Q-->L
2C49B	162 - 166	162	I-->A
2C49B	162 - 166	162	I-->D
2C49B	162 - 166	162	I-->F
2C49B	162 - 166	162	I-->G
2C49B	162 - 166	162	I-->N
2C49B	162 - 166	162	I-->P
2C49B	162 - 166	162	I-->Q
2C49B	162 - 166	162	I-->R
2C49B	162 - 166	162	I-->Y
2C49B	162 - 166	163	D-->N
2C49B	162 - 166	163	D-->S

2C49B	162 - 166	164	D-->G
2C49B	162 - 166	164	D-->H
2C49B	162 - 166	164	D-->I
2C49B	162 - 166	164	D-->K
2C49B	162 - 166	164	D-->L
2C49B	162 - 166	164	D-->P
2C49B	162 - 166	165	Y-->A
2C49B	162 - 166	165	Y-->G
2C49B	162 - 166	165	Y-->N
2C49B	162 - 166	165	Y-->R
2C49B	162 - 166	165	Y-->S
2C49B	162 - 166	165	Y-->T
2C49B	162 - 166	165	Y-->V
2C49B	162 - 166	166	L-->A
2C49B	162 - 166	166	L-->K
2C49B	162 - 166	166	L-->N
2C49B	162 - 166	166	L-->P
2C49B	162 - 166	166	L-->T
2GUKB	8 - 13	8	S-->N
2GUKB	8 - 13	8	S-->P
2GUKB	8 - 13	10	L-->G
2GUKB	8 - 13	10	L-->Q
2GUKB	8 - 13	11	R-->I
2GUKB	8 - 13	11	R-->Q
2GUKB	8 - 13	11	R-->W
1LR0A	118 - 123	119	E-->P
1LR0A	118 - 123	119	E-->Y

1LR0A	118	123	120	D-->E
1LR0A	118	123	120	D-->K
1LR0A	118	123	120	D-->L
1LR0A	118	123	121	L-->P
1LR0A	118	123	121	L-->R
1LR0A	118	123	122	S-->D
1LR0A	118	123	123	L-->G
2QHKA	37	42	38	P-->H
2QHKA	37	42	40	Y-->F
2QHKA	37	42	42	S-->Y
1S2XA	37	41	37	T-->C
1S2XA	37	41	37	T-->H
1S2XA	37	41	37	T-->I
1S2XA	37	41	37	T-->N
1S2XA	37	41	37	T-->P
1S2XA	37	41	37	T-->R
1S2XA	37	41	37	T-->S
1S2XA	37	41	37	T-->V
1S2XA	37	41	38	S-->D
1S2XA	37	41	38	S-->E
1S2XA	37	41	38	S-->F
1S2XA	37	41	38	S-->H
1S2XA	37	41	38	S-->L
1S2XA	37	41	38	S-->M
1S2XA	37	41	38	S-->N
1S2XA	37	41	38	S-->P
1S2XA	37	41	38	S-->R

1S2XA	37 - 41	38	S-->Y
1S2XA	37 - 41	39	L-->A
1S2XA	37 - 41	39	L-->D
1S2XA	37 - 41	39	L-->E
1S2XA	37 - 41	39	L-->G
1S2XA	37 - 41	39	L-->H
1S2XA	37 - 41	39	L-->M
1S2XA	37 - 41	39	L-->P
1S2XA	37 - 41	39	L-->S
1S2XA	37 - 41	39	L-->V
1S2XA	37 - 41	40	K-->A
1S2XA	37 - 41	40	K-->D
1S2XA	37 - 41	40	K-->E
1S2XA	37 - 41	40	K-->F
1S2XA	37 - 41	40	K-->L
1S2XA	37 - 41	40	K-->P
1S2XA	37 - 41	40	K-->R
1S2XA	37 - 41	40	K-->S
1S2XA	37 - 41	40	K-->V
1S2XA	37 - 41	40	K-->Y
1S2XA	37 - 41	41	D-->A
1S2XA	37 - 41	41	D-->G
1S2XA	37 - 41	41	D-->H
1S2XA	37 - 41	41	D-->K
1S2XA	37 - 41	41	D-->L
1S2XA	37 - 41	41	D-->N
1S2XA	37 - 41	41	D-->P

1S2XA	37 - 41	41	D-->T
1S2XA	146 - 151	147	V-->D
1S2XA	146 - 151	150	Q-->K
1GU9C	146 - 150	146	K-->A
1GU9C	146 - 150	146	K-->E
1GU9C	146 - 150	146	K-->G
1GU9C	146 - 150	146	K-->I
1GU9C	146 - 150	146	K-->N
1GU9C	146 - 150	146	K-->R
1GU9C	146 - 150	146	K-->S
1GU9C	146 - 150	146	K-->T
1GU9C	146 - 150	146	K-->V
1GU9C	146 - 150	147	L-->A
1GU9C	146 - 150	147	L-->C
1GU9C	146 - 150	147	L-->G
1GU9C	146 - 150	147	L-->H
1GU9C	146 - 150	147	L-->K
1GU9C	146 - 150	147	L-->N
1GU9C	146 - 150	147	L-->P
1GU9C	146 - 150	147	L-->Q
1GU9C	146 - 150	147	L-->T
1GU9C	146 - 150	147	L-->Y
1GU9C	146 - 150	148	K-->A
1GU9C	146 - 150	148	K-->D
1GU9C	146 - 150	148	K-->F
1GU9C	146 - 150	148	K-->G
1GU9C	146 - 150	148	K-->I

1GU9C	146 - 150	148	K-->L
1GU9C	146 - 150	148	K-->P
1GU9C	146 - 150	148	K-->Q
1GU9C	146 - 150	148	K-->R
1GU9C	146 - 150	148	K-->T
1GU9C	146 - 150	148	K-->V
1GU9C	146 - 150	148	K-->Y
1GU9C	146 - 150	149	A-->D
1GU9C	146 - 150	149	A-->E
1GU9C	146 - 150	149	A-->F
1GU9C	146 - 150	149	A-->H
1GU9C	146 - 150	149	A-->I
1GU9C	146 - 150	149	A-->K
1GU9C	146 - 150	149	A-->Q
1GU9C	146 - 150	149	A-->R
1GU9C	146 - 150	149	A-->S
1GU9C	146 - 150	149	A-->T
1GU9C	146 - 150	149	A-->V
1GU9C	146 - 150	150	A-->D
1GU9C	146 - 150	150	A-->E
1GU9C	146 - 150	150	A-->G
1GU9C	146 - 150	150	A-->I
1GU9C	146 - 150	150	A-->L
1GU9C	146 - 150	150	A-->N
1GU9C	146 - 150	150	A-->P
1GU9C	146 - 150	150	A-->S
1GU9C	146 - 150	150	A-->V

1GU9C	146	150	150	A-->Y
1XBWA	16	21	18	D-->A
1XBWA	16	21	19	I-->A
1XBWA	16	21	20	I-->S
1EF1C	544	548	544	I-->V
1EF1C	544	548	544	I-->Y
1EF1C	544	548	545	H-->A
1EF1C	544	548	545	H-->C
1EF1C	544	548	545	H-->D
1EF1C	544	548	545	H-->E
1EF1C	544	548	545	H-->F
1EF1C	544	548	545	H-->G
1EF1C	544	548	545	H-->I
1EF1C	544	548	545	H-->K
1EF1C	544	548	545	H-->N
1EF1C	544	548	545	H-->P
1EF1C	544	548	545	H-->Q
1EF1C	544	548	545	H-->S
1EF1C	544	548	545	H-->T
1EF1C	544	548	545	H-->V
1EF1C	544	548	546	A-->F
1EF1C	544	548	546	A-->I
1EF1C	544	548	546	A-->L
1EF1C	544	548	546	A-->P
1EF1C	544	548	546	A-->T
1EF1C	544	548	546	A-->V
1EF1C	544	548	547	E-->A

1EF1C	544	548	547	E-->D
1EF1C	544	548	547	E-->F
1EF1C	544	548	547	E-->G
1EF1C	544	548	548	N-->A
1EF1C	544	548	548	N-->E
1EF1C	544	548	548	N-->I
1EF1C	544	548	548	N-->L
1EF1C	544	548	548	N-->P
1EF1C	544	548	548	N-->S
1EF1C	544	548	548	N-->W
2V94B	84	89	88	I-->V
1R0DA	806	811	810	H-->V
1X9RB	51	55	51	K-->G
1X9RB	51	55	51	K-->H
1X9RB	51	55	51	K-->P
1X9RB	51	55	51	K-->Q
1X9RB	51	55	51	K-->S
1X9RB	51	55	51	K-->T
1X9RB	51	55	51	K-->V
1X9RB	51	55	52	D-->H
1X9RB	51	55	52	D-->K
1X9RB	51	55	52	D-->P
1X9RB	51	55	52	D-->S
1X9RB	51	55	52	D-->T
1X9RB	51	55	53	A-->D
1X9RB	51	55	53	A-->E
1X9RB	51	55	53	A-->G

1X9RB	51 - 55	53	A-->I
1X9RB	51 - 55	53	A-->K
1X9RB	51 - 55	53	A-->R
1X9RB	51 - 55	54	F-->E
1X9RB	51 - 55	54	F-->G
1X9RB	51 - 55	54	F-->K
1X9RB	51 - 55	54	F-->M
1X9RB	51 - 55	54	F-->N
1X9RB	51 - 55	54	F-->P
1X9RB	51 - 55	54	F-->R
1X9RB	51 - 55	54	F-->T
1X9RB	51 - 55	54	F-->V
1X9RB	51 - 55	54	F-->W
1X9RB	51 - 55	54	F-->Y
1X9RB	51 - 55	55	D-->G
1X9RB	51 - 55	55	D-->K
1X9RB	51 - 55	55	D-->L
1X9RB	51 - 55	55	D-->S
1X9RB	51 - 55	55	D-->V
1X9RB	51 - 55	55	D-->Y
2FBLA	119 - 124	120	D-->G
2FBLA	119 - 124	123	Q-->E
2FBLA	119 - 124	123	Q-->H
2FBLA	119 - 124	119	N-->A
2FBLA	119 - 124	119	N-->Q
2FBLA	119 - 124	119	N-->V
2FBLA	119 - 124	121	A-->T

2FBLA	119	- 124	123	A-->D
2FBLA	119	- 124	124	L-->I
1XXMC	1006	- 1011	1011	Q-->I
1XXMC	1085	- 1090	1086	A-->E
1XXMC	1085	- 1090	1086	A-->I
1QR0A	220	- 224	220	Y-->A
1QR0A	220	- 224	220	Y-->E
1QR0A	220	- 224	220	Y-->G
1QR0A	220	- 224	220	Y-->P
1QR0A	220	- 224	220	Y-->Q
1QR0A	220	- 224	220	Y-->S
1QR0A	220	- 224	220	Y-->V
1QR0A	220	- 224	221	E-->G
1QR0A	220	- 224	221	E-->M
1QR0A	220	- 224	221	E-->P
1QR0A	220	- 224	222	E-->D
1QR0A	220	- 224	222	E-->G
1QR0A	220	- 224	222	E-->H
1QR0A	220	- 224	222	E-->T
1QR0A	220	- 224	222	E-->V
1QR0A	220	- 224	223	L-->D
1QR0A	220	- 224	223	L-->E
1QR0A	220	- 224	223	L-->G
1QR0A	220	- 224	223	L-->H
1QR0A	220	- 224	223	L-->P
1QR0A	220	- 224	223	L-->V
1QR0A	220	- 224	224	L-->A

1QR0A	220 - 224	224	L-->I
1QR0A	220 - 224	224	L-->P
1QR0A	220 - 224	224	L-->T
1QR0A	220 - 224	224	L-->V
2CY7A	371 - 376	371	S-->V
2CY7A	371 - 376	373	D-->T
1QA7A	106 - 111	108	D-->L
1QA7A	106 - 111	110	P-->V
1STMA	106 - 110	106	P-->A
1STMA	106 - 110	106	P-->D
1STMA	106 - 110	106	P-->E
1STMA	106 - 110	106	P-->F
1STMA	106 - 110	106	P-->G
1STMA	106 - 110	106	P-->K
1STMA	106 - 110	106	P-->L
1STMA	106 - 110	106	P-->S
1STMA	106 - 110	106	P-->V
1STMA	106 - 110	106	P-->W
1STMA	106 - 110	107	T-->A
1STMA	106 - 110	107	T-->D
1STMA	106 - 110	107	T-->G
1STMA	106 - 110	107	T-->I
1STMA	106 - 110	107	T-->K
1STMA	106 - 110	107	T-->P
1STMA	106 - 110	107	T-->Q
1STMA	106 - 110	107	T-->S
1STMA	106 - 110	107	T-->W

1STMA	106 - 110	108	L-->D
1STMA	106 - 110	108	L-->E
1STMA	106 - 110	108	L-->F
1STMA	106 - 110	108	L-->I
1STMA	106 - 110	108	L-->N
1STMA	106 - 110	108	L-->V
1STMA	106 - 110	109	K-->A
1STMA	106 - 110	109	K-->D
1STMA	106 - 110	109	K-->E
1STMA	106 - 110	109	K-->H
1STMA	106 - 110	109	K-->I
1STMA	106 - 110	109	K-->M
1STMA	106 - 110	109	K-->N
1STMA	106 - 110	109	K-->P
1STMA	106 - 110	109	K-->Q
1STMA	106 - 110	109	K-->S
1STMA	106 - 110	109	K-->T
1STMA	106 - 110	109	K-->V
1STMA	106 - 110	110	G-->C
1STMA	106 - 110	110	G-->D
1STMA	106 - 110	110	G-->H
1STMA	106 - 110	110	G-->I
1STMA	106 - 110	110	G-->L
1STMA	106 - 110	110	G-->N
1STMA	106 - 110	110	G-->P
1STMA	106 - 110	110	G-->Q
1STMA	106 - 110	110	G-->R

1STMA	106 - 110	110	G-->T
1STMA	106 - 110	110	G-->V
1QCSA	14 - 19	14	D-->E
1QCSA	14 - 19	14	D-->N
1QCSA	14 - 19	14	D-->S
1QCSA	14 - 19	17	S-->E
1QCSA	14 - 19	17	S-->G
1QCSA	14 - 19	17	S-->N
1QCSA	14 - 19	17	S-->Q
1QCSA	14 - 19	18	L-->T
1QCSA	14 - 19	18	L-->V
1QCSA	14 - 19	19	S-->N
1QCSA	14 - 19	19	S-->V
2G5GX	202 - 207	202	A-->I
2G5GX	202 - 207	205	L-->I
2G5GX	202 - 207	206	V-->P
2G5GX	202 - 207	207	H-->I
2G5GX	202 - 207	207	H-->V
2QCKA	9 - 14	9	E-->F
2QCKA	9 - 14	9	E-->V
2QCKA	9 - 14	10	G-->C
2QCKA	9 - 14	10	G-->N
2QCKA	9 - 14	11	T-->L
2QCKA	9 - 14	12	F-->S
2QCKA	9 - 14	12	F-->V
2QCKA	9 - 14	13	K-->E
2QCKA	9 - 14	13	K-->R

2QCKA	63 - 68	66	I-->P
2H9AB	63 - 69	68	P-->N
2H9AB	246 - 250	246	I-->A
2H9AB	246 - 250	246	I-->D
2H9AB	246 - 250	246	I-->G
2H9AB	246 - 250	246	I-->K
2H9AB	246 - 250	246	I-->L
2H9AB	246 - 250	246	I-->M
2H9AB	246 - 250	246	I-->T
2H9AB	246 - 250	246	I-->V
2H9AB	246 - 250	247	G-->L
2H9AB	246 - 250	247	G-->S
2H9AB	246 - 250	248	Q-->D
2H9AB	246 - 250	248	Q-->G
2H9AB	246 - 250	248	Q-->H
2H9AB	246 - 250	248	Q-->I
2H9AB	246 - 250	248	Q-->L
2H9AB	246 - 250	248	Q-->N
2H9AB	246 - 250	248	Q-->P
2H9AB	246 - 250	248	Q-->S
2H9AB	246 - 250	248	Q-->T
2H9AB	246 - 250	249	E-->F
2H9AB	246 - 250	249	E-->G
2H9AB	246 - 250	249	E-->P
2H9AB	246 - 250	249	E-->S
2H9AB	246 - 250	249	E-->T
2H9AB	246 - 250	250	A-->D

2H9AB	246 - 250	250	A-->I
2H9AB	246 - 250	250	A-->T
1FX4A	1063 - 1067	1063	G-->F
1FX4A	1063 - 1067	1063	G-->Q
1FX4A	1063 - 1067	1063	G-->R
1FX4A	1063 - 1067	1063	G-->S
1FX4A	1063 - 1067	1064	E-->A
1FX4A	1063 - 1067	1064	E-->K
1FX4A	1063 - 1067	1064	E-->L
1FX4A	1063 - 1067	1064	E-->M
1FX4A	1063 - 1067	1064	E-->T
1FX4A	1063 - 1067	1064	E-->V
1FX4A	1063 - 1067	1064	E-->Y
1FX4A	1063 - 1067	1065	D-->A
1FX4A	1063 - 1067	1065	D-->I
1FX4A	1063 - 1067	1065	D-->S
1FX4A	1063 - 1067	1065	D-->T
1FX4A	1063 - 1067	1065	D-->V
1FX4A	1063 - 1067	1065	D-->Y
1FX4A	1063 - 1067	1066	R-->D
1FX4A	1063 - 1067	1066	R-->E
1FX4A	1063 - 1067	1066	R-->G
1FX4A	1063 - 1067	1066	R-->K
1FX4A	1063 - 1067	1066	R-->L
1FX4A	1063 - 1067	1066	R-->V
1FX4A	1063 - 1067	1067	N-->G
1FX4A	1063 - 1067	1067	N-->I

1FX4A	1063 - 1067	1067	N-->L
1FX4A	1063 - 1067	1067	N-->P
1FX4A	1063 - 1067	1067	N-->S
1FX4A	1063 - 1067	1067	N-->V
1FX4A	1063 - 1067	1067	N-->Y
1V83A	262 - 267	264	L-->A
1V83A	262 - 267	265	I-->E
1V83A	262 - 267	265	I-->P
1V83A	262 - 267	265	I-->V
1V83A	262 - 267	267	Q-->G
1V83A	262 - 267	267	Q-->L
1V83A	262 - 267	267	Q-->S
1V83A	283 - 288	284	E-->G
1V83A	283 - 288	284	E-->K
1V83A	283 - 288	284	E-->N
2IY2A	5 - 11	6	P-->N
2IY2A	5 - 11	10	I-->L
1LSHB	1473 - 1479	1474	A-->V
1D7BA	181 - 185	181	Y-->A
1D7BA	181 - 185	181	Y-->D
1D7BA	181 - 185	181	Y-->K
1D7BA	181 - 185	182	Q-->P
1D7BA	181 - 185	183	N-->S
1D7BA	181 - 185	185	L-->D
2IHYB	73 - 77	73	A-->E
2IHYB	73 - 77	73	A-->G
2IHYB	73 - 77	73	A-->K

2IHYB	73 - 77	73	A-->L
2IHYB	73 - 77	73	A-->N
2IHYB	73 - 77	73	A-->P
2IHYB	73 - 77	73	A-->Q
2IHYB	73 - 77	73	A-->R
2IHYB	73 - 77	73	A-->S
2IHYB	73 - 77	73	A-->T
2IHYB	73 - 77	73	A-->V
2IHYB	73 - 77	73	A-->Y
2IHYB	73 - 77	74	E-->A
2IHYB	73 - 77	74	E-->D
2IHYB	73 - 77	74	E-->G
2IHYB	73 - 77	74	E-->K
2IHYB	73 - 77	74	E-->L
2IHYB	73 - 77	74	E-->N
2IHYB	73 - 77	74	E-->S
2IHYB	73 - 77	74	E-->T
2IHYB	73 - 77	74	E-->V
2IHYB	73 - 77	74	E-->Y
2IHYB	73 - 77	75	T-->A
2IHYB	73 - 77	75	T-->D
2IHYB	73 - 77	75	T-->I
2IHYB	73 - 77	75	T-->L
2IHYB	73 - 77	75	T-->P
2IHYB	73 - 77	75	T-->R
2IHYB	73 - 77	75	T-->V
2IHYB	73 - 77	75	T-->W

2IHYB	73	77	75	T-->Y
2IHYB	73	77	76	V-->G
2IHYB	73	77	76	V-->H
2IHYB	73	77	76	V-->Q
2IHYB	73	77	76	V-->T
2IHYB	73	77	77	R-->A
2IHYB	73	77	77	R-->D
2IHYB	73	77	77	R-->E
2IHYB	73	77	77	R-->F
2IHYB	73	77	77	R-->L
2IHYB	73	77	77	R-->S
2IHYB	73	77	77	R-->T
2IHYB	97	102	97	V-->D
2IHYB	97	102	97	V-->K
2IHYB	97	102	97	V-->M
2IHYB	97	102	98	I-->A
2IHYB	97	102	98	I-->L
2IHYB	97	102	98	I-->P
2IHYB	97	102	98	I-->R
2IHYB	97	102	99	D-->T
2IHYB	97	102	101	V-->A
2IHYB	97	102	101	V-->G
1YS3B	387	393	390	R-->V
2P0AA	104	108	104	W-->G
2P0AA	104	108	104	W-->K
2P0AA	104	108	104	W-->S
2P0AA	104	108	104	W-->T

2P0AA	104	- 108	105	S-->M
2P0AA	104	- 108	107	Y-->A
2P0AA	104	- 108	107	Y-->P
2P0AA	104	- 108	107	Y-->T
2P0AA	104	- 108	108	F-->A
2P0AA	104	- 108	108	F-->L
2P0AA	104	- 108	108	F-->N
2P0AA	104	- 108	108	F-->S
2P0AA	195	- 200	199	Y-->D
2P0AA	195	- 200	199	Y-->S
2FY6A	34	- 38	34	P-->A
2FY6A	34	- 38	34	P-->G
2FY6A	34	- 38	34	P-->K
2FY6A	34	- 38	34	P-->N
2FY6A	34	- 38	34	P-->Q
2FY6A	34	- 38	34	P-->V
2FY6A	34	- 38	35	H-->A
2FY6A	34	- 38	35	H-->D
2FY6A	34	- 38	35	H-->F
2FY6A	34	- 38	35	H-->G
2FY6A	34	- 38	35	H-->K
2FY6A	34	- 38	35	H-->L
2FY6A	34	- 38	35	H-->M
2FY6A	34	- 38	35	H-->N
2FY6A	34	- 38	35	H-->P
2FY6A	34	- 38	35	H-->R
2FY6A	34	- 38	35	H-->S

2FY6A	34 - 38	35	H-->T
2FY6A	34 - 38	36	T-->A
2FY6A	34 - 38	36	T-->D
2FY6A	34 - 38	36	T-->E
2FY6A	34 - 38	36	T-->G
2FY6A	34 - 38	36	T-->I
2FY6A	34 - 38	36	T-->N
2FY6A	34 - 38	36	T-->W
2FY6A	34 - 38	36	T-->Y
2FY6A	34 - 38	37	L-->G
2FY6A	34 - 38	37	L-->M
2FY6A	34 - 38	37	L-->S
2FY6A	34 - 38	37	L-->V
2FY6A	34 - 38	38	S-->L
2FY6A	34 - 38	38	S-->M
2FY6A	107 - 112	112	Y-->A
2FY6A	129 - 133	129	T-->G
2FY6A	129 - 133	129	T-->P
2FY6A	129 - 133	130	I-->A
2FY6A	129 - 133	130	I-->E
2FY6A	129 - 133	130	I-->G
2FY6A	129 - 133	130	I-->P
2FY6A	129 - 133	131	A-->D
2FY6A	129 - 133	131	A-->G
2FY6A	129 - 133	131	A-->I
2FY6A	129 - 133	131	A-->P
2FY6A	129 - 133	131	A-->Q

2FY6A	129	- 133	131	A-->T
2FY6A	129	- 133	132	Q-->A
2FY6A	129	- 133	132	Q-->C
2FY6A	129	- 133	132	Q-->E
2FY6A	129	- 133	132	Q-->F
2FY6A	129	- 133	132	Q-->G
2FY6A	129	- 133	132	Q-->K
2FY6A	129	- 133	132	Q-->L
2FY6A	129	- 133	132	Q-->P
2FY6A	129	- 133	132	Q-->S
2FY6A	129	- 133	132	Q-->T
2FY6A	129	- 133	132	Q-->V
2FY6A	129	- 133	132	Q-->Y
2FY6A	129	- 133	133	S-->A
2FY6A	129	- 133	133	S-->D
2FY6A	129	- 133	133	S-->G
2FY6A	129	- 133	133	S-->I
2FY6A	129	- 133	133	S-->L
2FY6A	129	- 133	133	S-->N
2FY6A	129	- 133	133	S-->V
2EO5A	144	- 149	144	F-->A
2EO5A	144	- 149	144	F-->D
2EO5A	144	- 149	145	G-->A
2EO5A	144	- 149	145	G-->P
2EO5A	144	- 149	147	I-->D
2EO5A	144	- 149	147	I-->F
2EO5A	266	- 270	266	A-->K

2EO5A	266 - 270	266	A-->R
2EO5A	266 - 270	266	A-->S
2EO5A	266 - 270	267	I-->D
2EO5A	266 - 270	267	I-->K
2EO5A	266 - 270	267	I-->P
2EO5A	266 - 270	267	I-->S
2EO5A	266 - 270	267	I-->V
2EO5A	266 - 270	268	E-->A
2EO5A	266 - 270	268	E-->I
2EO5A	266 - 270	268	E-->L
2EO5A	266 - 270	268	E-->N
2EO5A	266 - 270	268	E-->R
2EO5A	266 - 270	269	N-->D
2EO5A	266 - 270	269	N-->E
2EO5A	266 - 270	269	N-->L
2EO5A	266 - 270	269	N-->P
2EO5A	266 - 270	269	N-->T
2EO5A	266 - 270	269	N-->Y
2EO5A	266 - 270	270	F-->G
2EO5A	266 - 270	270	F-->M
2EO5A	266 - 270	270	F-->T
2EO5A	266 - 270	270	F-->V
2H0UA	94 - 99	95	Y-->P
2H0UA	94 - 99	97	K-->S
2OKUB	471 - 475	471	P-->F
2OKUB	471 - 475	471	P-->I
2OKUB	471 - 475	471	P-->K

2OKUB	471 - 475	471	P-->R
2OKUB	471 - 475	472	E-->A
2OKUB	471 - 475	472	E-->D
2OKUB	471 - 475	472	E-->G
2OKUB	471 - 475	472	E-->L
2OKUB	471 - 475	472	E-->N
2OKUB	471 - 475	472	E-->S
2OKUB	471 - 475	473	L-->A
2OKUB	471 - 475	473	L-->C
2OKUB	471 - 475	473	L-->D
2OKUB	471 - 475	473	L-->F
2OKUB	471 - 475	473	L-->H
2OKUB	471 - 475	473	L-->I
2OKUB	471 - 475	473	L-->N
2OKUB	471 - 475	473	L-->Q
2OKUB	471 - 475	473	L-->R
2OKUB	471 - 475	473	L-->S
2OKUB	471 - 475	473	L-->Y
2OKUB	471 - 475	474	Q-->A
2OKUB	471 - 475	474	Q-->C
2OKUB	471 - 475	474	Q-->D
2OKUB	471 - 475	474	Q-->F
2OKUB	471 - 475	474	Q-->G
2OKUB	471 - 475	474	Q-->H
2OKUB	471 - 475	474	Q-->K
2OKUB	471 - 475	474	Q-->L
2OKUB	471 - 475	474	Q-->P

2OKUB	471 - 475	475	P-->A
2OKUB	471 - 475	475	P-->D
2OKUB	471 - 475	475	P-->E
2OKUB	471 - 475	475	P-->F
2OKUB	471 - 475	475	P-->G
2OKUB	471 - 475	475	P-->I
2OKUB	471 - 475	475	P-->K
2OKUB	471 - 475	475	P-->L
2OKUB	471 - 475	475	P-->N
2OKUB	471 - 475	475	P-->Q
2OKUB	471 - 475	475	P-->T
2OKUB	471 - 475	475	P-->W
2OKUB	477 - 482	477	K-->V
2OKUB	477 - 482	479	A-->V
2OKUB	477 - 482	480	L-->V
2OKUB	477 - 482	481	A-->P
2OKUB	525 - 529	525	L-->A
2OKUB	525 - 529	525	L-->D
2OKUB	525 - 529	525	L-->E
2OKUB	525 - 529	525	L-->V
2OKUB	525 - 529	526	A-->G
2OKUB	525 - 529	526	A-->I
2OKUB	525 - 529	526	A-->K
2OKUB	525 - 529	526	A-->L
2OKUB	525 - 529	526	A-->N
2OKUB	525 - 529	526	A-->P
2OKUB	525 - 529	526	A-->R

2OKUB	525 - 529	526	A-->S
2OKUB	525 - 529	526	A-->T
2OKUB	525 - 529	526	A-->V
2OKUB	525 - 529	527	A-->E
2OKUB	525 - 529	527	A-->G
2OKUB	525 - 529	527	A-->I
2OKUB	525 - 529	527	A-->N
2OKUB	525 - 529	527	A-->S
2OKUB	525 - 529	527	A-->V
2OKUB	525 - 529	528	N-->A
2OKUB	525 - 529	528	N-->C
2OKUB	525 - 529	528	N-->D
2OKUB	525 - 529	528	N-->E
2OKUB	525 - 529	528	N-->F
2OKUB	525 - 529	528	N-->G
2OKUB	525 - 529	528	N-->I
2OKUB	525 - 529	528	N-->M
2OKUB	525 - 529	528	N-->P
2OKUB	525 - 529	528	N-->R
2OKUB	525 - 529	528	N-->S
2OKUB	525 - 529	528	N-->W
2OKUB	525 - 529	528	N-->Y
2OKUB	525 - 529	529	D-->E
2OKUB	525 - 529	529	D-->F
2OKUB	525 - 529	529	D-->G
2OKUB	525 - 529	529	D-->I
2OKUB	525 - 529	529	D-->N

2OKUB	525 - 529	529	D-->P
2OKUB	525 - 529	529	D-->Q
2OKUB	525 - 529	529	D-->R
2OKUB	525 - 529	529	D-->S
2OKUB	525 - 529	529	D-->T
2OKUB	525 - 529	529	D-->Y
1USPA	115 - 120	115	H-->F
1USPA	115 - 120	115	H-->Y
1USPA	122 - 127	127	T-->M
1H9RA	193 - 198	195	V-->F
1H9RA	193 - 198	198	N-->Y
2CWQC	7 - 11	7	R-->A
2CWQC	7 - 11	7	R-->D
2CWQC	7 - 11	7	R-->E
2CWQC	7 - 11	7	R-->H
2CWQC	7 - 11	7	R-->I
2CWQC	7 - 11	7	R-->K
2CWQC	7 - 11	7	R-->L
2CWQC	7 - 11	7	R-->N
2CWQC	7 - 11	7	R-->V
2CWQC	7 - 11	7	R-->W
2CWQC	7 - 11	8	V-->C
2CWQC	7 - 11	8	V-->D
2CWQC	7 - 11	8	V-->R
2CWQC	7 - 11	8	V-->S
2CWQC	7 - 11	8	V-->Y
2CWQC	7 - 11	9	L-->F

2CWQC	7 - 11	9	L-->G
2CWQC	7 - 11	9	L-->I
2CWQC	7 - 11	9	L-->P
2CWQC	7 - 11	9	L-->S
2CWQC	7 - 11	9	L-->T
2CWQC	7 - 11	10	Q-->A
2CWQC	7 - 11	10	Q-->D
2CWQC	7 - 11	10	Q-->E
2CWQC	7 - 11	10	Q-->G
2CWQC	7 - 11	10	Q-->I
2CWQC	7 - 11	10	Q-->L
2CWQC	7 - 11	10	Q-->P
2CWQC	7 - 11	10	Q-->R
2CWQC	7 - 11	10	Q-->S
2CWQC	7 - 11	10	Q-->V
2CWQC	7 - 11	10	Q-->Y
2CWQC	7 - 11	11	A-->G
2CWQC	7 - 11	11	A-->I
2CWQC	7 - 11	11	A-->L
2CWQC	7 - 11	11	A-->P
2CWQC	7 - 11	11	A-->R
2CWQC	7 - 11	11	A-->T
2CWQC	7 - 11	11	A-->V
2CWQC	13 - 19	14	E-->C
2CWQC	71 - 76	71	E-->A
2CWQC	71 - 76	71	E-->S
2CWQC	71 - 76	71	E-->V

2CWQC	111 - 115	111	A-->C
2CWQC	111 - 115	111	A-->D
2CWQC	111 - 115	111	A-->E
2CWQC	111 - 115	111	A-->F
2CWQC	111 - 115	111	A-->K
2CWQC	111 - 115	111	A-->L
2CWQC	111 - 115	111	A-->N
2CWQC	111 - 115	111	A-->P
2CWQC	111 - 115	111	A-->S
2CWQC	111 - 115	111	A-->Y
2CWQC	111 - 115	112	P-->A
2CWQC	111 - 115	112	P-->C
2CWQC	111 - 115	112	P-->D
2CWQC	111 - 115	112	P-->E
2CWQC	111 - 115	112	P-->F
2CWQC	111 - 115	112	P-->G
2CWQC	111 - 115	112	P-->H
2CWQC	111 - 115	112	P-->I
2CWQC	111 - 115	112	P-->L
2CWQC	111 - 115	112	P-->N
2CWQC	111 - 115	112	P-->S
2CWQC	111 - 115	112	P-->V
2CWQC	111 - 115	112	P-->W
2CWQC	111 - 115	112	P-->Y
2CWQC	111 - 115	113	L-->R
2CWQC	111 - 115	113	L-->W
2CWQC	111 - 115	114	L-->A

2CWQC	111 - 115	114	L-->C
2CWQC	111 - 115	114	L-->D
2CWQC	111 - 115	114	L-->F
2CWQC	111 - 115	114	L-->I
2CWQC	111 - 115	114	L-->K
2CWQC	111 - 115	114	L-->P
2CWQC	111 - 115	114	L-->R
2CWQC	111 - 115	115	E-->A
2CWQC	111 - 115	115	E-->D
2CWQC	111 - 115	115	E-->F
2CWQC	111 - 115	115	E-->G
2CWQC	111 - 115	115	E-->I
2CWQC	111 - 115	115	E-->K
2CWQC	111 - 115	115	E-->M
2CWQC	111 - 115	115	E-->P
2CWQC	111 - 115	115	E-->R
2CWQC	111 - 115	115	E-->S
2CWQC	111 - 115	115	E-->T
1VKBA	122 - 126	123	E-->G
1VKBA	122 - 126	123	E-->V
1VKBA	122 - 126	124	W-->A
1VKBA	122 - 126	124	W-->G
1VKBA	122 - 126	124	W-->I
1VKBA	122 - 126	124	W-->L
1VKBA	122 - 126	124	W-->N
1VKBA	122 - 126	124	W-->P
1VKBA	122 - 126	124	W-->T

1VKBA	122	126	124	W-->V
1VKBA	122	126	125	L-->E
1VKBA	122	126	125	L-->Y
1VKBA	122	126	126	F-->A
1VKBA	122	126	126	F-->E
1VKBA	122	126	126	F-->G
1VKBA	122	126	126	F-->T
1O1XA	92	97	94	L-->F
1O1XA	92	97	94	L-->S
1O1XA	92	97	96	R-->A
2GIAB	168	172	168	F-->G
2GIAB	168	172	170	Q-->I
2GIAB	168	172	170	Q-->Y
2GIAB	168	172	171	H-->G
2GIAB	168	172	172	Y-->K
2GIAB	168	172	172	Y-->T
2VRYA	36	41	36	P-->L
2VRYA	36	41	37	S-->K
2VRYA	36	41	37	S-->V
2VRYA	36	41	39	L-->K
2VRYA	36	41	40	P-->L
2VRYA	36	41	40	P-->T
2VRYA	36	41	41	L-->A
2VRYA	36	41	41	L-->G
2VRYA	36	41	41	L-->H
2VRYA	36	41	41	L-->I
2VRYA	36	41	41	L-->K

2VRYA	36 - 41	41	L-->P
2VRYA	36 - 41	41	L-->R
2VRYA	36 - 41	41	L-->S
1J1IA	51 - 55	51	S-->E
1J1IA	51 - 55	51	S-->G
1J1IA	51 - 55	51	S-->L
1J1IA	51 - 55	51	S-->P
1J1IA	51 - 55	52	E-->D
1J1IA	51 - 55	52	E-->M
1J1IA	51 - 55	52	E-->P
1J1IA	51 - 55	52	E-->S
1J1IA	51 - 55	52	E-->W
1J1IA	51 - 55	53	G-->K
1J1IA	51 - 55	54	N-->D
1J1IA	51 - 55	54	N-->K
1J1IA	51 - 55	54	N-->L
1J1IA	51 - 55	54	N-->T
1J1IA	51 - 55	54	N-->W
1J1IA	51 - 55	55	W-->E
1J1IA	51 - 55	55	W-->H
1J1IA	51 - 55	55	W-->I
1J1IA	51 - 55	55	W-->P
1J1IA	51 - 55	55	W-->R
1J1IA	51 - 55	55	W-->S
1J1IA	51 - 55	55	W-->T
1J1IA	51 - 55	55	W-->V
1YB0B	10 - 16	10	P-->R

1YB0B	10 - 16	13	Y-->D
1YB0B	81 - 85	81	P-->E
1YB0B	81 - 85	81	P-->G
1YB0B	81 - 85	81	P-->L
1YB0B	81 - 85	81	P-->T
1YB0B	81 - 85	81	P-->Y
1YB0B	81 - 85	82	G-->A
1YB0B	81 - 85	82	G-->F
1YB0B	81 - 85	82	G-->K
1YB0B	81 - 85	82	G-->N
1YB0B	81 - 85	82	G-->S
1YB0B	81 - 85	82	G-->T
1YB0B	81 - 85	82	G-->Y
1YB0B	81 - 85	83	N-->E
1YB0B	81 - 85	83	N-->F
1YB0B	81 - 85	83	N-->Q
1YB0B	81 - 85	83	N-->S
1YB0B	81 - 85	83	N-->T
1YB0B	81 - 85	83	N-->V
1YB0B	81 - 85	84	R-->G
1YB0B	81 - 85	84	R-->I
1YB0B	81 - 85	84	R-->N
1YB0B	81 - 85	84	R-->P
1YB0B	81 - 85	84	R-->T
1YB0B	81 - 85	84	R-->V
1YB0B	81 - 85	85	E-->D
1YB0B	81 - 85	85	E-->F

1YB0B	81 - 85	85	E-->L	
1UZXA	115 - 119	115	P-->T	
1UZXA	115 - 119	115	P-->Y	
1UZXA	115 - 119	116	I-->A	
1UZXA	115 - 119	116	I-->E	
1UZXA	115 - 119	116	I-->G	
1UZXA	115 - 119	117	Q-->D	
1UZXA	115 - 119	117	Q-->E	
1UZXA	115 - 119	117	Q-->G	
1UZXA	115 - 119	118	E-->I	
1UZXA	115 - 119	118	E-->L	
1UZXA	115 - 119	119	Y-->G	
1UZXA	144 - 148	144	V-->A	
1UZXA	144 - 148	144	V-->E	
1UZXA	144 - 148	144	V-->G	
1UZXA	144 - 148	144	V-->P	
1UZXA	144 - 148	144	V-->Q	
1UZXA	144 - 148	144	V-->T	
1UZXA	144 - 148	145	V-->D	
1UZXA	144 - 148	145	V-->E	
1UZXA	144 - 148	145	V-->G	
1UZXA	144 - 148	145	V-->L	
1UZXA	144 - 148	145	V-->T	
1UZXA	144 - 148	146	Q-->F	
1UZXA	144 - 148	146	Q-->G	
1UZXA	144 - 148	146	Q-->H	
1UZXA	144 - 148	146	Q-->I	

1UZXA	144	148	146	Q-->L
1UZXA	144	148	146	Q-->M
1UZXA	144	148	146	Q-->R
1UZXA	144	148	146	Q-->V
1UZXA	144	148	146	Q-->Y
1UZXA	144	148	147	E-->D
1UZXA	144	148	147	E-->M
1UZXA	144	148	147	E-->N
1UZXA	144	148	147	E-->R
1UZXA	144	148	147	E-->T
1UZXA	144	148	148	L-->A
1UZXA	144	148	148	L-->F
1UZXA	144	148	148	L-->G
1UZXA	144	148	148	L-->H
1UZXA	144	148	148	L-->M
1UZXA	144	148	148	L-->V
1JMVC	52	56	52	V-->F
1JMVC	52	56	52	V-->I
1JMVC	52	56	53	N-->A
1JMVC	52	56	53	N-->D
1JMVC	52	56	53	N-->E
1JMVC	52	56	53	N-->K
1JMVC	52	56	53	N-->Y
1JMVC	52	56	54	M-->D
1JMVC	52	56	54	M-->E
1JMVC	52	56	54	M-->G
1JMVC	52	56	54	M-->I

1JMVC	52	56	54	M-->N
1JMVC	52	56	54	M-->Q
1JMVC	52	56	54	M-->R
1JMVC	52	56	54	M-->V
1JMVC	52	56	54	M-->Y
1JMVC	52	56	55	S-->A
1JMVC	52	56	55	S-->G
1JMVC	52	56	55	S-->P
1JMVC	52	56	55	S-->R
1JMVC	52	56	56	S-->A
1JMVC	52	56	56	S-->Q
1JMVC	52	56	56	S-->T
1T0FC	510	515	514	D-->S
1T0FC	521	526	523	Y-->I
1T15A	1748	1753	1749	P-->Q
1T15A	1748	1753	1750	K-->C
2OD6B	87	92	87	P-->T
2OD6B	87	92	88	V-->G
2OD6B	87	92	89	F-->I
2OD6B	87	92	89	F-->L
2OD6B	87	92	89	F-->R
2IS5D	36	41	40	Q-->A
2IS5D	36	41	40	Q-->K
2IS5D	90	95	92	N-->E
2IS5D	90	95	92	N-->L
1O6AB	92	97	96	L-->N
1O6AB	92	97	96	L-->P

1O6AB	92 - 97	97	E-->P
1O6AB	92 - 97	97	E-->W
2P7HB	54 - 59	56	T-->F
2P7HB	165 - 169	165	E-->A
2P7HB	165 - 169	165	E-->G
2P7HB	165 - 169	165	E-->L
2P7HB	165 - 169	165	E-->N
2P7HB	165 - 169	165	E-->T
2P7HB	165 - 169	165	E-->V
2P7HB	165 - 169	166	A-->L
2P7HB	165 - 169	166	A-->R
2P7HB	165 - 169	166	A-->Y
2P7HB	165 - 169	167	E-->A
2P7HB	165 - 169	167	E-->F
2P7HB	165 - 169	167	E-->G
2P7HB	165 - 169	167	E-->K
2P7HB	165 - 169	167	E-->Q
2P7HB	165 - 169	167	E-->R
2P7HB	165 - 169	167	E-->V
2P7HB	165 - 169	167	E-->W
2P7HB	165 - 169	168	F-->A
2P7HB	165 - 169	168	F-->D
2P7HB	165 - 169	168	F-->E
2P7HB	165 - 169	168	F-->G
2P7HB	165 - 169	168	F-->I
2P7HB	165 - 169	168	F-->L
2P7HB	165 - 169	168	F-->N

2P7HB	165	169	168	F-->W
2P7HB	165	169	169	A-->D
2P7HB	165	169	169	A-->F
2P7HB	165	169	169	A-->G
2P7HB	165	169	169	A-->K
2P7HB	165	169	169	A-->S
2P7HB	165	169	169	A-->Y
1TZ0A	18	24	21	V-->T
1YLMA	73	77	73	D-->A
1YLMA	73	77	73	D-->F
1YLMA	73	77	73	D-->G
1YLMA	73	77	73	D-->H
1YLMA	73	77	73	D-->I
1YLMA	73	77	73	D-->K
1YLMA	73	77	73	D-->L
1YLMA	73	77	73	D-->N
1YLMA	73	77	73	D-->P
1YLMA	73	77	73	D-->R
1YLMA	73	77	73	D-->S
1YLMA	73	77	73	D-->T
1YLMA	73	77	73	D-->V
1YLMA	73	77	73	D-->W
1YLMA	73	77	74	I-->D
1YLMA	73	77	74	I-->F
1YLMA	73	77	74	I-->G
1YLMA	73	77	74	I-->L
1YLMA	73	77	74	I-->N

1YLMA	73 - 77	74	I-->R
1YLMA	73 - 77	74	I-->S
1YLMA	73 - 77	74	I-->T
1YLMA	73 - 77	74	I-->V
1YLMA	73 - 77	74	I-->W
1YLMA	73 - 77	75	L-->A
1YLMA	73 - 77	75	L-->D
1YLMA	73 - 77	75	L-->E
1YLMA	73 - 77	75	L-->G
1YLMA	73 - 77	75	L-->I
1YLMA	73 - 77	75	L-->N
1YLMA	73 - 77	75	L-->P
1YLMA	73 - 77	75	L-->S
1YLMA	73 - 77	75	L-->T
1YLMA	73 - 77	75	L-->V
1YLMA	73 - 77	75	L-->W
1YLMA	73 - 77	75	L-->Y
1YLMA	73 - 77	76	V-->A
1YLMA	73 - 77	76	V-->D
1YLMA	73 - 77	76	V-->H
1YLMA	73 - 77	76	V-->K
1YLMA	73 - 77	76	V-->R
1YLMA	73 - 77	76	V-->T
1YLMA	73 - 77	77	D-->E
1YLMA	73 - 77	77	D-->G
1YLMA	73 - 77	77	D-->H
1YLMA	73 - 77	77	D-->I

1YLMA	73	- 77	77	D-->K
1YLMA	73	- 77	77	D-->L
1YLMA	73	- 77	77	D-->N
1YLMA	73	- 77	77	D-->P
1YLMA	73	- 77	77	D-->Q
1YLMA	73	- 77	77	D-->R
1YLMA	73	- 77	77	D-->S
1YLMA	73	- 77	77	D-->V
1YLMA	73	- 77	77	D-->W
1YLMA	73	- 77	77	D-->Y
1YLMA	96	- 100	96	R-->A
1YLMA	96	- 100	96	R-->D
1YLMA	96	- 100	96	R-->E
1YLMA	96	- 100	96	R-->G
1YLMA	96	- 100	96	R-->H
1YLMA	96	- 100	96	R-->I
1YLMA	96	- 100	96	R-->K
1YLMA	96	- 100	96	R-->L
1YLMA	96	- 100	96	R-->N
1YLMA	96	- 100	96	R-->S
1YLMA	96	- 100	96	R-->T
1YLMA	96	- 100	96	R-->V
1YLMA	96	- 100	97	K-->A
1YLMA	96	- 100	97	K-->D
1YLMA	96	- 100	97	K-->E
1YLMA	96	- 100	97	K-->F
1YLMA	96	- 100	97	K-->G

1YLMA	96 - 100	97	K-->H
1YLMA	96 - 100	97	K-->I
1YLMA	96 - 100	97	K-->L
1YLMA	96 - 100	97	K-->M
1YLMA	96 - 100	97	K-->P
1YLMA	96 - 100	97	K-->S
1YLMA	96 - 100	97	K-->T
1YLMA	96 - 100	97	K-->V
1YLMA	96 - 100	98	T-->D
1YLMA	96 - 100	98	T-->E
1YLMA	96 - 100	98	T-->I
1YLMA	96 - 100	98	T-->K
1YLMA	96 - 100	98	T-->L
1YLMA	96 - 100	98	T-->P
1YLMA	96 - 100	98	T-->R
1YLMA	96 - 100	98	T-->W
1YLMA	96 - 100	98	T-->Y
1YLMA	96 - 100	99	L-->D
1YLMA	96 - 100	99	L-->G
1YLMA	96 - 100	99	L-->R
1YLMA	96 - 100	99	L-->S
1YLMA	96 - 100	99	L-->T
1YLMA	96 - 100	99	L-->V
1YLMA	96 - 100	99	L-->Y
1YLMA	96 - 100	100	V-->F
1YLMA	96 - 100	100	V-->I
1YLMA	96 - 100	100	V-->K

1YLMA	96 - 100	100	V-->L
1YLMA	96 - 100	100	V-->N
1YLMA	96 - 100	100	V-->P
1YLMA	96 - 100	100	V-->Q
1YLMA	96 - 100	100	V-->S
1YLMA	96 - 100	100	V-->Y
2RBBA	86 - 90	86	E-->A
2RBBA	86 - 90	86	E-->D
2RBBA	86 - 90	86	E-->G
2RBBA	86 - 90	86	E-->R
2RBBA	86 - 90	87	A-->D
2RBBA	86 - 90	87	A-->E
2RBBA	86 - 90	87	A-->F
2RBBA	86 - 90	87	A-->G
2RBBA	86 - 90	87	A-->H
2RBBA	86 - 90	87	A-->K
2RBBA	86 - 90	87	A-->M
2RBBA	86 - 90	87	A-->N
2RBBA	86 - 90	87	A-->P
2RBBA	86 - 90	87	A-->Q
2RBBA	86 - 90	87	A-->R
2RBBA	86 - 90	87	A-->S
2RBBA	86 - 90	87	A-->W
2RBBA	86 - 90	88	Y-->A
2RBBA	86 - 90	88	Y-->C
2RBBA	86 - 90	88	Y-->F
2RBBA	86 - 90	88	Y-->G

2RBBA	86 - 90	88	Y-->H	
2RBBA	86 - 90	88	Y-->I	
2RBBA	86 - 90	88	Y-->K	
2RBBA	86 - 90	88	Y-->L	
2RBBA	86 - 90	88	Y-->P	
2RBBA	86 - 90	88	Y-->R	
2RBBA	86 - 90	88	Y-->V	
2RBBA	86 - 90	89	E-->C	
2RBBA	86 - 90	89	E-->S	
2RBBA	86 - 90	89	E-->V	
2RBBA	86 - 90	90	L-->A	
2RBBA	86 - 90	90	L-->D	
2RBBA	86 - 90	90	L-->R	
2RBBA	86 - 90	90	L-->V	
2R2AB	135 - 140	136	N-->I	
2R2AB	135 - 140	137	L-->I	
2R2AB	135 - 140	139	T-->I	
2GAXA	28 - 33	28	S-->N	
2GAXA	28 - 33	30	I-->A	
2GAXA	28 - 33	32	A-->P	
2GAXA	28 - 33	33	Q-->A	
2GAXA	28 - 33	33	Q-->T	
2AMJB	154 - 158	154	G-->A	
2AMJB	154 - 158	154	G-->D	
2AMJB	154 - 158	154	G-->E	
2AMJB	154 - 158	154	G-->I	
2AMJB	154 - 158	154	G-->K	

2AMJB	154 - 158	154	G-->L
2AMJB	154 - 158	154	G-->N
2AMJB	154 - 158	154	G-->P
2AMJB	154 - 158	154	G-->Q
2AMJB	154 - 158	154	G-->R
2AMJB	154 - 158	154	G-->S
2AMJB	154 - 158	154	G-->T
2AMJB	154 - 158	154	G-->V
2AMJB	154 - 158	154	G-->Y
2AMJB	154 - 158	155	V-->A
2AMJB	154 - 158	155	V-->C
2AMJB	154 - 158	155	V-->D
2AMJB	154 - 158	155	V-->E
2AMJB	154 - 158	155	V-->F
2AMJB	154 - 158	155	V-->G
2AMJB	154 - 158	155	V-->H
2AMJB	154 - 158	155	V-->I
2AMJB	154 - 158	155	V-->K
2AMJB	154 - 158	155	V-->L
2AMJB	154 - 158	155	V-->N
2AMJB	154 - 158	155	V-->P
2AMJB	154 - 158	155	V-->R
2AMJB	154 - 158	155	V-->S
2AMJB	154 - 158	155	V-->T
2AMJB	154 - 158	155	V-->Y
2AMJB	154 - 158	156	D-->A
2AMJB	154 - 158	156	D-->C

2AMJB	154 - 158	156	D-->E
2AMJB	154 - 158	156	D-->F
2AMJB	154 - 158	156	D-->G
2AMJB	154 - 158	156	D-->H
2AMJB	154 - 158	156	D-->I
2AMJB	154 - 158	156	D-->K
2AMJB	154 - 158	156	D-->L
2AMJB	154 - 158	156	D-->N
2AMJB	154 - 158	156	D-->P
2AMJB	154 - 158	156	D-->Q
2AMJB	154 - 158	156	D-->S
2AMJB	154 - 158	156	D-->T
2AMJB	154 - 158	156	D-->V
2AMJB	154 - 158	156	D-->W
2AMJB	154 - 158	156	D-->Y
2AMJB	154 - 158	157	G-->A
2AMJB	154 - 158	157	G-->D
2AMJB	154 - 158	157	G-->E
2AMJB	154 - 158	157	G-->F
2AMJB	154 - 158	157	G-->I
2AMJB	154 - 158	157	G-->K
2AMJB	154 - 158	157	G-->L
2AMJB	154 - 158	157	G-->N
2AMJB	154 - 158	157	G-->P
2AMJB	154 - 158	157	G-->Q
2AMJB	154 - 158	157	G-->R
2AMJB	154 - 158	157	G-->S

2AMJB	154	158	157	G-->T
2AMJB	154	158	157	G-->V
2AMJB	154	158	157	G-->W
2AMJB	154	158	157	G-->Y
2AMJB	154	158	158	V-->A
2AMJB	154	158	158	V-->D
2AMJB	154	158	158	V-->E
2AMJB	154	158	158	V-->F
2AMJB	154	158	158	V-->G
2AMJB	154	158	158	V-->I
2AMJB	154	158	158	V-->K
2AMJB	154	158	158	V-->L
2AMJB	154	158	158	V-->N
2AMJB	154	158	158	V-->P
2AMJB	154	158	158	V-->Q
2AMJB	154	158	158	V-->S
2AMJB	154	158	158	V-->T
2AMJB	154	158	158	V-->W
2AMJB	154	158	158	V-->Y
2QH9B	102	106	102	E-->A
2QH9B	102	106	102	E-->D
2QH9B	102	106	102	E-->G
2QH9B	102	106	102	E-->I
2QH9B	102	106	102	E-->K
2QH9B	102	106	102	E-->N
2QH9B	102	106	102	E-->Q
2QH9B	102	106	102	E-->R

2QH9B	102 - 106	102	E-->T
2QH9B	102 - 106	102	E-->V
2QH9B	102 - 106	102	E-->W
2QH9B	102 - 106	102	E-->Y
2QH9B	102 - 106	103	E-->K
2QH9B	102 - 106	103	E-->L
2QH9B	102 - 106	103	E-->R
2QH9B	102 - 106	103	E-->Y
2QH9B	102 - 106	104	F-->D
2QH9B	102 - 106	104	F-->G
2QH9B	102 - 106	104	F-->H
2QH9B	102 - 106	104	F-->K
2QH9B	102 - 106	104	F-->L
2QH9B	102 - 106	104	F-->N
2QH9B	102 - 106	104	F-->Y
2QH9B	102 - 106	105	D-->C
2QH9B	102 - 106	105	D-->I
2QH9B	102 - 106	105	D-->M
2QH9B	102 - 106	105	D-->P
2QH9B	102 - 106	105	D-->R
2QH9B	102 - 106	106	S-->C
2QH9B	102 - 106	106	S-->D
2QH9B	102 - 106	106	S-->E
2QH9B	102 - 106	106	S-->I
2QH9B	102 - 106	106	S-->L
2QH9B	102 - 106	106	S-->N
2QH9B	102 - 106	106	S-->P

2QH9B	102 - 106	106	S-->T
2QH9B	102 - 106	106	S-->V
1S7IA	106 - 110	106	P-->A
1S7IA	106 - 110	106	P-->D
1S7IA	106 - 110	106	P-->E
1S7IA	106 - 110	106	P-->F
1S7IA	106 - 110	106	P-->G
1S7IA	106 - 110	106	P-->H
1S7IA	106 - 110	106	P-->I
1S7IA	106 - 110	106	P-->K
1S7IA	106 - 110	106	P-->L
1S7IA	106 - 110	106	P-->M
1S7IA	106 - 110	106	P-->Q
1S7IA	106 - 110	106	P-->S
1S7IA	106 - 110	106	P-->T
1S7IA	106 - 110	106	P-->V
1S7IA	106 - 110	106	P-->W
1S7IA	106 - 110	107	P-->A
1S7IA	106 - 110	107	P-->C
1S7IA	106 - 110	107	P-->D
1S7IA	106 - 110	107	P-->E
1S7IA	106 - 110	107	P-->F
1S7IA	106 - 110	107	P-->G
1S7IA	106 - 110	107	P-->I
1S7IA	106 - 110	107	P-->K
1S7IA	106 - 110	107	P-->N
1S7IA	106 - 110	107	P-->Q

1S7IA	106 - 110	107	P-->R
1S7IA	106 - 110	107	P-->T
1S7IA	106 - 110	107	P-->V
1S7IA	106 - 110	107	P-->W
1S7IA	106 - 110	107	P-->Y
1S7IA	106 - 110	108	G-->L
1S7IA	106 - 110	108	G-->P
1S7IA	106 - 110	108	G-->R
1S7IA	106 - 110	108	G-->T
1S7IA	106 - 110	109	R-->E
1S7IA	106 - 110	109	R-->G
1S7IA	106 - 110	109	R-->L
1S7IA	106 - 110	109	R-->P
1S7IA	106 - 110	109	R-->S
1S7IA	106 - 110	109	R-->T
1S7IA	106 - 110	109	R-->Y
1S7IA	106 - 110	110	L-->A
1S7IA	106 - 110	110	L-->E
1S7IA	106 - 110	110	L-->F
1S7IA	106 - 110	110	L-->G
1S7IA	106 - 110	110	L-->I
1S7IA	106 - 110	110	L-->K
1S7IA	106 - 110	110	L-->M
1S7IA	106 - 110	110	L-->P
1S7IA	106 - 110	110	L-->R
1S7IA	106 - 110	110	L-->S
1S7IA	106 - 110	110	L-->Y

1RO7A	89	- 93	89	E-->D
1RO7A	89	- 93	89	E-->F
1RO7A	89	- 93	89	E-->L
1RO7A	89	- 93	89	E-->Q
1RO7A	89	- 93	89	E-->T
1RO7A	89	- 93	90	N-->E
1RO7A	89	- 93	90	N-->G
1RO7A	89	- 93	90	N-->L
1RO7A	89	- 93	90	N-->S
1RO7A	89	- 93	91	F-->G
1RO7A	89	- 93	91	F-->H
1RO7A	89	- 93	91	F-->I
1RO7A	89	- 93	91	F-->K
1RO7A	89	- 93	91	F-->P
1RO7A	89	- 93	91	F-->Q
1RO7A	89	- 93	91	F-->T
1RO7A	89	- 93	91	F-->V
1RO7A	89	- 93	92	V-->D
1RO7A	89	- 93	92	V-->K
1RO7A	89	- 93	92	V-->Q
1RO7A	89	- 93	93	K-->A
1RO7A	89	- 93	93	K-->D
1RO7A	89	- 93	93	K-->F
1RO7A	89	- 93	93	K-->L
1RO7A	89	- 93	93	K-->S
1RO7A	89	- 93	93	K-->Y
1RO7A	137	- 143	143	L-->P

1RO7A	169	- 174	170	N-->Y
1RO7A	169	- 174	172	L-->T
1RO7A	169	- 174	173	K-->D
1RO7A	169	- 174	173	K-->L
1RO7A	169	- 174	173	K-->R
2J22A	33	- 37	33	S-->A
2J22A	33	- 37	33	S-->E
2J22A	33	- 37	33	S-->F
2J22A	33	- 37	33	S-->G
2J22A	33	- 37	33	S-->H
2J22A	33	- 37	33	S-->I
2J22A	33	- 37	33	S-->L
2J22A	33	- 37	33	S-->N
2J22A	33	- 37	33	S-->P
2J22A	33	- 37	33	S-->R
2J22A	33	- 37	33	S-->V
2J22A	33	- 37	33	S-->Y
2J22A	33	- 37	34	R-->A
2J22A	33	- 37	34	R-->C
2J22A	33	- 37	34	R-->D
2J22A	33	- 37	34	R-->G
2J22A	33	- 37	34	R-->H
2J22A	33	- 37	34	R-->I
2J22A	33	- 37	34	R-->K
2J22A	33	- 37	34	R-->L
2J22A	33	- 37	34	R-->P
2J22A	33	- 37	34	R-->Q

2J22A	33 - 37	34	R-->S
2J22A	33 - 37	34	R-->T
2J22A	33 - 37	34	R-->V
2J22A	33 - 37	34	R-->W
2J22A	33 - 37	34	R-->Y
2J22A	33 - 37	35	L-->A
2J22A	33 - 37	35	L-->C
2J22A	33 - 37	35	L-->D
2J22A	33 - 37	35	L-->E
2J22A	33 - 37	35	L-->F
2J22A	33 - 37	35	L-->G
2J22A	33 - 37	35	L-->I
2J22A	33 - 37	35	L-->N
2J22A	33 - 37	35	L-->P
2J22A	33 - 37	35	L-->Q
2J22A	33 - 37	35	L-->S
2J22A	33 - 37	35	L-->Y
2J22A	33 - 37	36	A-->D
2J22A	33 - 37	36	A-->E
2J22A	33 - 37	36	A-->F
2J22A	33 - 37	36	A-->H
2J22A	33 - 37	36	A-->I
2J22A	33 - 37	36	A-->K
2J22A	33 - 37	36	A-->L
2J22A	33 - 37	36	A-->M
2J22A	33 - 37	36	A-->R
2J22A	33 - 37	36	A-->S

2J22A	33 - 37	36	A-->T
2J22A	33 - 37	36	A-->V
2J22A	33 - 37	36	A-->Y
2J22A	33 - 37	37	V-->C
2J22A	33 - 37	37	V-->D
2J22A	33 - 37	37	V-->E
2J22A	33 - 37	37	V-->F
2J22A	33 - 37	37	V-->G
2J22A	33 - 37	37	V-->S
2J22A	33 - 37	37	V-->W
3BOCA	397 - 402	398	W-->S
3BOCA	397 - 402	398	W-->Y
3BOCA	397 - 402	400	A-->G
1ZN6A	97 - 101	97	R-->H
1ZN6A	97 - 101	99	A-->I
1ZN6A	97 - 101	99	A-->N
1ZN6A	97 - 101	100	W-->A
1ZN6A	97 - 101	100	W-->D
1ZN6A	97 - 101	100	W-->G
1ZN6A	97 - 101	100	W-->L
1ZN6A	97 - 101	100	W-->Q
1ZN6A	97 - 101	100	W-->T
1ZN6A	97 - 101	100	W-->V
1ZN6A	97 - 101	101	A-->C
1XFKA	17 - 22	18	E-->R
1XFKA	17 - 22	21	Q-->D
1XFKA	35 - 40	36	G-->A

1XFKA	35 - 40	37	E-->F	
1XFKA	35 - 40	38	L-->P	
1XFKA	35 - 40	39	A-->L	
1XFKA	35 - 40	39	A-->T	
1XFKA	35 - 40	40	N-->H	
1XFKA	54 - 58	54	A-->C	
1XFKA	54 - 58	54	A-->D	
1XFKA	54 - 58	54	A-->E	
1XFKA	54 - 58	54	A-->H	
1XFKA	54 - 58	54	A-->L	
1XFKA	54 - 58	54	A-->N	
1XFKA	54 - 58	54	A-->P	
1XFKA	54 - 58	54	A-->S	
1XFKA	54 - 58	54	A-->V	
1XFKA	54 - 58	55	G-->A	
1XFKA	54 - 58	55	G-->H	
1XFKA	54 - 58	55	G-->I	
1XFKA	54 - 58	55	G-->K	
1XFKA	54 - 58	55	G-->S	
1XFKA	54 - 58	55	G-->T	
1XFKA	54 - 58	55	G-->V	
1XFKA	54 - 58	55	G-->Y	
1XFKA	54 - 58	56	V-->A	
1XFKA	54 - 58	56	V-->F	
1XFKA	54 - 58	56	V-->G	
1XFKA	54 - 58	56	V-->I	
1XFKA	54 - 58	56	V-->K	

1XFKA	54 - 58	56	V-->L
1XFKA	54 - 58	56	V-->N
1XFKA	54 - 58	56	V-->P
1XFKA	54 - 58	56	V-->Q
1XFKA	54 - 58	56	V-->S
1XFKA	54 - 58	56	V-->T
1XFKA	54 - 58	57	E-->A
1XFKA	54 - 58	57	E-->D
1XFKA	54 - 58	57	E-->F
1XFKA	54 - 58	57	E-->G
1XFKA	54 - 58	57	E-->H
1XFKA	54 - 58	57	E-->K
1XFKA	54 - 58	57	E-->L
1XFKA	54 - 58	57	E-->N
1XFKA	54 - 58	57	E-->P
1XFKA	54 - 58	57	E-->R
1XFKA	54 - 58	57	E-->S
1XFKA	54 - 58	57	E-->V
1XFKA	54 - 58	57	E-->Y
1XFKA	54 - 58	58	R-->A
1XFKA	54 - 58	58	R-->E
1XFKA	54 - 58	58	R-->F
1XFKA	54 - 58	58	R-->G
1XFKA	54 - 58	58	R-->H
1XFKA	54 - 58	58	R-->I
1XFKA	54 - 58	58	R-->K
1XFKA	54 - 58	58	R-->L

1XFKA	54 - 58	58	R-->M
1XFKA	54 - 58	58	R-->N
1XFKA	54 - 58	58	R-->P
1XFKA	54 - 58	58	R-->Q
1XFKA	54 - 58	58	R-->T
1XFKA	54 - 58	58	R-->V
1XFKA	54 - 58	58	R-->W
2IGPA	166 - 170	166	K-->H
2IGPA	166 - 170	166	K-->N
2IGPA	166 - 170	166	K-->P
2IGPA	166 - 170	167	S-->L
2IGPA	166 - 170	168	S-->C
2IGPA	166 - 170	168	S-->D
2IGPA	166 - 170	168	S-->R
2IGPA	166 - 170	169	M-->A
2IGPA	166 - 170	169	M-->F
2IGPA	166 - 170	169	M-->G
2IGPA	166 - 170	169	M-->K
2IGPA	166 - 170	169	M-->P
2IGPA	166 - 170	169	M-->R
2IGPA	166 - 170	169	M-->T
2IGPA	166 - 170	169	M-->V
2IGPA	166 - 170	169	M-->Y
2IGPA	166 - 170	170	Y-->V
2FYGA	75 - 79	76	Y-->T
2FYGA	75 - 79	77	C-->Q
2FYGA	75 - 79	78	R-->L

2FYGA	75 - 79	78	R-->T
2FYGA	75 - 79	79	C-->A
2FYGA	75 - 79	79	C-->T
2P57A	102 - 106	102	A-->D
2P57A	102 - 106	102	A-->E
2P57A	102 - 106	102	A-->F
2P57A	102 - 106	102	A-->G
2P57A	102 - 106	102	A-->K
2P57A	102 - 106	102	A-->N
2P57A	102 - 106	102	A-->R
2P57A	102 - 106	102	A-->T
2P57A	102 - 106	102	A-->W
2P57A	102 - 106	102	A-->Y
2P57A	102 - 106	103	N-->A
2P57A	102 - 106	103	N-->G
2P57A	102 - 106	103	N-->R
2P57A	102 - 106	104	T-->A
2P57A	102 - 106	104	T-->G
2P57A	102 - 106	104	T-->I
2P57A	102 - 106	104	T-->L
2P57A	102 - 106	104	T-->P
2P57A	102 - 106	105	K-->A
2P57A	102 - 106	105	K-->D
2P57A	102 - 106	105	K-->G
2P57A	102 - 106	105	K-->I
2P57A	102 - 106	105	K-->N
2P57A	102 - 106	105	K-->R

2P57A	102 - 106	105	K-->S
2P57A	102 - 106	105	K-->V
2P57A	102 - 106	106	Y-->E
2P57A	102 - 106	106	Y-->F
2P57A	102 - 106	106	Y-->G
2P57A	102 - 106	106	Y-->T
2P57A	102 - 106	106	Y-->V
2YVRA	30 - 34	30	R-->I
2YVRA	30 - 34	31	D-->L
2YVRA	30 - 34	31	D-->S
2YVRA	30 - 34	32	C-->F
2YVRA	30 - 34	32	C-->G
2YVRA	30 - 34	32	C-->N
2YVRA	30 - 34	32	C-->P
2YVRA	30 - 34	32	C-->S
2YVRA	30 - 34	32	C-->V
2YVRA	30 - 34	33	Q-->D
2YVRA	30 - 34	33	Q-->F
2YVRA	30 - 34	33	Q-->G
2YVRA	30 - 34	33	Q-->R
2YVRA	30 - 34	33	Q-->V
2YVRA	30 - 34	34	L-->V
1XIYA	71 - 75	71	Y-->D
1XIYA	71 - 75	71	Y-->F
1XIYA	71 - 75	71	Y-->I
1XIYA	71 - 75	71	Y-->P
1XIYA	71 - 75	71	Y-->Q

1XIYA	71 - 75	71	Y-->R
1XIYA	71 - 75	72	D-->A
1XIYA	71 - 75	72	D-->G
1XIYA	71 - 75	72	D-->H
1XIYA	71 - 75	72	D-->L
1XIYA	71 - 75	73	Y-->D
1XIYA	71 - 75	73	Y-->F
1XIYA	71 - 75	73	Y-->G
1XIYA	71 - 75	73	Y-->K
1XIYA	71 - 75	73	Y-->L
1XIYA	71 - 75	74	F-->D
1XIYA	71 - 75	74	F-->I
1XIYA	71 - 75	74	F-->L
1XIYA	71 - 75	74	F-->T
1XIYA	71 - 75	74	F-->V
1XIYA	71 - 75	74	F-->Y
1XIYA	71 - 75	75	I-->C
1XIYA	71 - 75	75	I-->D
1XIYA	71 - 75	75	I-->P
1XIYA	71 - 75	75	I-->Q
1XIYA	115 - 119	115	S-->A
1XIYA	115 - 119	115	S-->C
1XIYA	115 - 119	115	S-->D
1XIYA	115 - 119	115	S-->G
1XIYA	115 - 119	115	S-->I
1XIYA	115 - 119	115	S-->L
1XIYA	115 - 119	115	S-->Q

1XIYA	115 - 119	115	S-->V
1XIYA	115 - 119	116	F-->D
1XIYA	115 - 119	116	F-->G
1XIYA	115 - 119	116	F-->I
1XIYA	115 - 119	116	F-->K
1XIYA	115 - 119	116	F-->M
1XIYA	115 - 119	116	F-->N
1XIYA	115 - 119	116	F-->P
1XIYA	115 - 119	116	F-->Q
1XIYA	115 - 119	116	F-->R
1XIYA	115 - 119	116	F-->S
1XIYA	115 - 119	116	F-->V
1XIYA	115 - 119	116	F-->W
1XIYA	115 - 119	117	T-->D
1XIYA	115 - 119	117	T-->E
1XIYA	115 - 119	117	T-->G
1XIYA	115 - 119	117	T-->H
1XIYA	115 - 119	117	T-->I
1XIYA	115 - 119	117	T-->K
1XIYA	115 - 119	117	T-->L
1XIYA	115 - 119	117	T-->M
1XIYA	115 - 119	117	T-->N
1XIYA	115 - 119	117	T-->P
1XIYA	115 - 119	117	T-->R
1XIYA	115 - 119	117	T-->S
1XIYA	115 - 119	117	T-->V
1XIYA	115 - 119	118	D-->A

1XIYA	115 - 119	118	D-->C
1XIYA	115 - 119	118	D-->F
1XIYA	115 - 119	118	D-->G
1XIYA	115 - 119	118	D-->I
1XIYA	115 - 119	118	D-->N
1XIYA	115 - 119	118	D-->P
1XIYA	115 - 119	118	D-->S
1XIYA	115 - 119	118	D-->T
1XIYA	115 - 119	118	D-->V
1XIYA	115 - 119	118	D-->W
1XIYA	115 - 119	119	S-->A
1XIYA	115 - 119	119	S-->E
1XIYA	115 - 119	119	S-->G
1XIYA	115 - 119	119	S-->K
1XIYA	115 - 119	119	S-->N
1XIYA	115 - 119	119	S-->P
1XIYA	115 - 119	119	S-->T
1XIYA	115 - 119	119	S-->V
1XIYA	115 - 119	119	S-->Y
2C2IA	7 - 12	7	V-->G
2C2IA	7 - 12	8	A-->P
2C2IA	7 - 12	8	A-->R
2C2IA	7 - 12	8	A-->S
2C2IA	7 - 12	9	D-->V
2C2IA	7 - 12	10	L-->N
2C2IA	7 - 12	11	A-->F
2C2IA	7 - 12	11	A-->K

2C2IA	48 - 52	48	P-->D
2C2IA	48 - 52	48	P-->G
2C2IA	48 - 52	48	P-->I
2C2IA	48 - 52	48	P-->L
2C2IA	48 - 52	48	P-->R
2C2IA	48 - 52	48	P-->S
2C2IA	48 - 52	48	P-->T
2C2IA	48 - 52	48	P-->V
2C2IA	48 - 52	48	P-->Y
2C2IA	48 - 52	49	E-->A
2C2IA	48 - 52	49	E-->D
2C2IA	48 - 52	49	E-->F
2C2IA	48 - 52	49	E-->Q
2C2IA	48 - 52	49	E-->S
2C2IA	48 - 52	49	E-->T
2C2IA	48 - 52	50	R-->A
2C2IA	48 - 52	50	R-->E
2C2IA	48 - 52	50	R-->F
2C2IA	48 - 52	50	R-->I
2C2IA	48 - 52	50	R-->K
2C2IA	48 - 52	50	R-->L
2C2IA	48 - 52	50	R-->M
2C2IA	48 - 52	50	R-->N
2C2IA	48 - 52	50	R-->S
2C2IA	48 - 52	51	A-->E
2C2IA	48 - 52	51	A-->F
2C2IA	48 - 52	51	A-->I

2C2IA	48 - 52	51	A-->M
2C2IA	48 - 52	51	A-->P
2C2IA	48 - 52	51	A-->T
2C2IA	48 - 52	51	A-->V
2C2IA	48 - 52	51	A-->Y
2C2IA	48 - 52	52	A-->E
2C2IA	48 - 52	52	A-->F
2C2IA	48 - 52	52	A-->I
2C2IA	48 - 52	52	A-->L
2C2IA	48 - 52	52	A-->R
2C2IA	48 - 52	52	A-->T
2C2IA	48 - 52	52	A-->V
2C2IA	70 - 75	70	L-->A
2C2IA	70 - 75	73	L-->G
2C2IA	70 - 75	75	H-->A
2C2IA	70 - 75	75	H-->D
2C2IA	70 - 75	75	H-->E
2C2IA	70 - 75	75	H-->G
2C2IA	70 - 75	75	H-->K
2C2IA	70 - 75	75	H-->Q
2IP1A	197 - 203	200	K-->A
2IP1A	259 - 264	259	G-->I
2IP1A	259 - 264	260	S-->T
2IP1A	259 - 264	261	T-->P
2IP1A	259 - 264	262	A-->L
2IP1A	259 - 264	262	A-->V
2IP1A	259 - 264	263	K-->E

2IP1A	274 - 278	274	I-->H
2IP1A	274 - 278	274	I-->L
2IP1A	274 - 278	274	I-->T
2IP1A	274 - 278	275	G-->P
2IP1A	274 - 278	275	G-->W
2IP1A	274 - 278	276	K-->T
2IP1A	274 - 278	277	F-->E
2IP1A	274 - 278	277	F-->H
2IP1A	274 - 278	277	F-->L
2IP1A	274 - 278	277	F-->W
2IP1A	274 - 278	278	H-->C
2IP1A	274 - 278	278	H-->P
2IP1A	274 - 278	278	H-->Q
2IP1A	274 - 278	278	H-->R
2IP1A	274 - 278	278	H-->V
2IP1A	280 - 285	283	Q-->A
2IP1A	280 - 285	283	Q-->E
2IP1A	280 - 285	283	Q-->I
2HQYA	31 - 36	31	F-->M
2HQYA	31 - 36	35	C-->D
2HQYA	31 - 36	35	C-->L
2HQYA	127 - 132	131	A-->Y
2I02A	106 - 110	106	P-->F
2I02A	106 - 110	106	P-->K
2I02A	106 - 110	106	P-->V
2I02A	106 - 110	107	E-->K
2I02A	106 - 110	107	E-->S

2I02A	106 - 110	107	E-->V
2I02A	106 - 110	108	L-->G
2I02A	106 - 110	108	L-->K
2I02A	106 - 110	108	L-->N
2I02A	106 - 110	108	L-->P
2I02A	106 - 110	108	L-->Q
2I02A	106 - 110	108	L-->Y
2I02A	106 - 110	109	Q-->D
2I02A	106 - 110	109	Q-->G
2I02A	106 - 110	109	Q-->K
2I02A	106 - 110	109	Q-->L
2I02A	106 - 110	109	Q-->R
2I02A	106 - 110	110	T-->A
2I02A	106 - 110	110	T-->D
2I02A	106 - 110	110	T-->E
2I02A	106 - 110	110	T-->F
2I02A	106 - 110	110	T-->G
2I02A	106 - 110	110	T-->I
2I02A	106 - 110	110	T-->K
2I02A	106 - 110	110	T-->L
2I02A	106 - 110	110	T-->N
2I02A	106 - 110	110	T-->Q
2I02A	106 - 110	110	T-->W
2BUYA	17 - 21	17	V-->A
2BUYA	17 - 21	17	V-->G
2BUYA	17 - 21	17	V-->K
2BUYA	17 - 21	17	V-->L

2BUYA	17 - 21	17	V-->P
2BUYA	17 - 21	17	V-->S
2BUYA	17 - 21	18	H-->A
2BUYA	17 - 21	18	H-->D
2BUYA	17 - 21	18	H-->E
2BUYA	17 - 21	18	H-->G
2BUYA	17 - 21	18	H-->I
2BUYA	17 - 21	18	H-->K
2BUYA	17 - 21	18	H-->L
2BUYA	17 - 21	18	H-->M
2BUYA	17 - 21	18	H-->N
2BUYA	17 - 21	18	H-->P
2BUYA	17 - 21	18	H-->Q
2BUYA	17 - 21	18	H-->S
2BUYA	17 - 21	18	H-->V
2BUYA	17 - 21	18	H-->W
2BUYA	17 - 21	18	H-->Y
2BUYA	17 - 21	19	I-->E
2BUYA	17 - 21	19	I-->G
2BUYA	17 - 21	19	I-->H
2BUYA	17 - 21	19	I-->K
2BUYA	17 - 21	20	G-->C
2BUYA	17 - 21	20	G-->I
2BUYA	17 - 21	20	G-->K
2BUYA	17 - 21	20	G-->Q
2BUYA	17 - 21	20	G-->T
2BUYA	17 - 21	20	G-->Y

2BUYA	17 - 21	21	L-->D
2BUYA	17 - 21	21	L-->F
2BUYA	17 - 21	21	L-->H
2BUYA	17 - 21	21	L-->N
2BUYA	17 - 21	21	L-->P
2BUYA	17 - 21	21	L-->S
2BUYA	17 - 21	21	L-->T
2BUYA	17 - 21	21	L-->W
2BUYA	17 - 21	21	L-->Y
2BUYA	149 - 153	149	A-->G
2BUYA	149 - 153	149	A-->I
2BUYA	149 - 153	149	A-->L
2BUYA	149 - 153	149	A-->P
2BUYA	149 - 153	149	A-->S
2BUYA	149 - 153	150	E-->A
2BUYA	149 - 153	150	E-->C
2BUYA	149 - 153	150	E-->D
2BUYA	149 - 153	150	E-->F
2BUYA	149 - 153	150	E-->I
2BUYA	149 - 153	150	E-->M
2BUYA	149 - 153	150	E-->P
2BUYA	149 - 153	150	E-->T
2BUYA	149 - 153	150	E-->V
2BUYA	149 - 153	150	E-->Y
2BUYA	149 - 153	151	A-->D
2BUYA	149 - 153	151	A-->E
2BUYA	149 - 153	151	A-->I

2BUYA	149 - 153	151	A-->K
2BUYA	149 - 153	151	A-->L
2BUYA	149 - 153	151	A-->Q
2BUYA	149 - 153	151	A-->T
2BUYA	149 - 153	152	N-->A
2BUYA	149 - 153	152	N-->C
2BUYA	149 - 153	152	N-->H
2BUYA	149 - 153	152	N-->I
2BUYA	149 - 153	152	N-->M
2BUYA	149 - 153	152	N-->P
2BUYA	149 - 153	152	N-->S
2BUYA	149 - 153	152	N-->T
2BUYA	149 - 153	152	N-->W
2BUYA	149 - 153	152	N-->Y
2BUYA	149 - 153	153	A-->G
2BUYA	149 - 153	153	A-->I
1XOVA	128 - 132	128	R-->C
1XOVA	128 - 132	128	R-->D
1XOVA	128 - 132	128	R-->F
1XOVA	128 - 132	128	R-->K
1XOVA	128 - 132	128	R-->P
1XOVA	128 - 132	128	R-->S
1XOVA	128 - 132	128	R-->Y
1XOVA	128 - 132	129	F-->I
1XOVA	128 - 132	129	F-->L
1XOVA	128 - 132	129	F-->N
1XOVA	128 - 132	129	F-->Q

1XOVA	128 - 132	129	F-->R
1XOVA	128 - 132	129	F-->S
1XOVA	128 - 132	129	F-->V
1XOVA	128 - 132	130	L-->D
1XOVA	128 - 132	130	L-->T
1XOVA	128 - 132	130	L-->V
1XOVA	128 - 132	131	N-->L
1XOVA	128 - 132	131	N-->P
1XOVA	128 - 132	131	N-->T
1XOVA	128 - 132	132	S-->N
1XOVA	128 - 132	132	S-->P
1XOVA	128 - 132	132	S-->V
1YX1B	164 - 168	164	N-->R
1YX1B	164 - 168	165	W-->I
1YX1B	164 - 168	165	W-->K
1YX1B	164 - 168	167	W-->V
2RGQB	27 - 31	27	V-->D
2RGQB	27 - 31	27	V-->L
2RGQB	27 - 31	29	N-->D
2RGQB	27 - 31	29	N-->G
2RGQB	27 - 31	29	N-->I
2RGQB	27 - 31	29	N-->L
2RGQB	27 - 31	29	N-->P
2RGQB	27 - 31	29	N-->R
2RGQB	27 - 31	29	N-->T
2RGQB	27 - 31	29	N-->Y
2RGQB	27 - 31	30	Y-->D

2RGQB	27 - 31	30	Y-->E
2RGQB	27 - 31	30	Y-->F
2RGQB	27 - 31	30	Y-->G
2RGQB	27 - 31	30	Y-->I
2RGQB	27 - 31	30	Y-->L
2RGQB	27 - 31	30	Y-->N
2RGQB	27 - 31	30	Y-->T
2RGQB	27 - 31	30	Y-->V
2RGQB	27 - 31	30	Y-->W
2RGQB	27 - 31	31	L-->D
2RGQB	27 - 31	31	L-->G
2RGQB	27 - 31	31	L-->N
2RGQB	27 - 31	31	L-->Q
2RGQB	27 - 31	31	L-->R
1KP6A	2 - 8	2	N-->D
1KP6A	2 - 8	3	A-->M
1KP6A	28 - 32	28	R-->D
1KP6A	28 - 32	28	R-->E
1KP6A	28 - 32	28	R-->G
1KP6A	28 - 32	28	R-->K
1KP6A	28 - 32	28	R-->P
1KP6A	28 - 32	28	R-->S
1KP6A	28 - 32	28	R-->T
1KP6A	28 - 32	28	R-->V
1KP6A	28 - 32	29	Y-->F
1KP6A	28 - 32	29	Y-->G
1KP6A	28 - 32	29	Y-->I

1KP6A	28 - 32	29	Y-->K
1KP6A	28 - 32	29	Y-->L
1KP6A	28 - 32	29	Y-->N
1KP6A	28 - 32	29	Y-->P
1KP6A	28 - 32	29	Y-->S
1KP6A	28 - 32	30	A-->D
1KP6A	28 - 32	30	A-->G
1KP6A	28 - 32	30	A-->I
1KP6A	28 - 32	30	A-->L
1KP6A	28 - 32	30	A-->P
1KP6A	28 - 32	30	A-->R
1KP6A	28 - 32	30	A-->T
1KP6A	28 - 32	30	A-->V
1KP6A	28 - 32	30	A-->Y
1KP6A	28 - 32	31	T-->A
1KP6A	28 - 32	31	T-->D
1KP6A	28 - 32	31	T-->F
1KP6A	28 - 32	31	T-->G
1KP6A	28 - 32	31	T-->I
1KP6A	28 - 32	31	T-->L
1KP6A	28 - 32	31	T-->N
1KP6A	28 - 32	31	T-->S
1KP6A	28 - 32	31	T-->V
1KP6A	28 - 32	32	A-->D
1KP6A	28 - 32	32	A-->G
1KP6A	28 - 32	32	A-->I
1KP6A	28 - 32	32	A-->K

1KP6A	28 - 32	32	A-->W	
1ZVCA	177 - 181	177	P-->E	
1ZVCA	177 - 181	177	P-->F	
1ZVCA	177 - 181	177	P-->G	
1ZVCA	177 - 181	177	P-->I	
1ZVCA	177 - 181	177	P-->L	
1ZVCA	177 - 181	177	P-->M	
1ZVCA	177 - 181	177	P-->T	
1ZVCA	177 - 181	178	E-->D	
1ZVCA	177 - 181	178	E-->K	
1ZVCA	177 - 181	178	E-->N	
1ZVCA	177 - 181	179	A-->E	
1ZVCA	177 - 181	179	A-->F	
1ZVCA	177 - 181	179	A-->I	
1ZVCA	177 - 181	179	A-->K	
1ZVCA	177 - 181	179	A-->L	
1ZVCA	177 - 181	179	A-->M	
1ZVCA	177 - 181	179	A-->N	
1ZVCA	177 - 181	179	A-->Q	
1ZVCA	177 - 181	179	A-->S	
1ZVCA	177 - 181	179	A-->T	
1ZVCA	177 - 181	179	A-->V	
1ZVCA	177 - 181	179	A-->Y	
1ZVCA	177 - 181	180	K-->A	
1ZVCA	177 - 181	180	K-->E	
1ZVCA	177 - 181	180	K-->G	
1ZVCA	177 - 181	180	K-->I	

1ZVCA	177 - 181	180	K-->N
1ZVCA	177 - 181	180	K-->P
1ZVCA	177 - 181	180	K-->R
1ZVCA	177 - 181	180	K-->S
1ZVCA	177 - 181	180	K-->T
1ZVCA	177 - 181	180	K-->V
1ZVCA	177 - 181	181	A-->D
1ZVCA	177 - 181	181	A-->F
1ZVCA	177 - 181	181	A-->I
1ZVCA	177 - 181	181	A-->K
1ZVCA	177 - 181	181	A-->L
1ZVCA	177 - 181	181	A-->N
1ZVCA	177 - 181	181	A-->Q
1ZVCA	177 - 181	181	A-->S
1ZVCA	177 - 181	181	A-->W
1ZVCA	177 - 181	181	A-->Y
1DQPA	26 - 31	26	E-->A
1DQPA	26 - 31	26	E-->I
1DQPA	26 - 31	29	V-->G
1DQPA	26 - 31	30	K-->N
1DQPA	154 - 158	154	V-->A
1DQPA	154 - 158	154	V-->C
1DQPA	154 - 158	154	V-->D
1DQPA	154 - 158	154	V-->G
1DQPA	154 - 158	154	V-->I
1DQPA	154 - 158	154	V-->K
1DQPA	154 - 158	154	V-->L

1DQPA	154 - 158	154	V-->T
1DQPA	154 - 158	155	D-->E
1DQPA	154 - 158	155	D-->F
1DQPA	154 - 158	155	D-->I
1DQPA	154 - 158	155	D-->K
1DQPA	154 - 158	155	D-->L
1DQPA	154 - 158	155	D-->N
1DQPA	154 - 158	155	D-->T
1DQPA	154 - 158	155	D-->V
1DQPA	154 - 158	156	A-->G
1DQPA	154 - 158	156	A-->K
1DQPA	154 - 158	156	A-->L
1DQPA	154 - 158	156	A-->P
1DQPA	154 - 158	156	A-->S
1DQPA	154 - 158	156	A-->V
1DQPA	154 - 158	157	I-->A
1DQPA	154 - 158	157	I-->D
1DQPA	154 - 158	157	I-->F
1DQPA	154 - 158	157	I-->G
1DQPA	154 - 158	157	I-->K
1DQPA	154 - 158	157	I-->P
1DQPA	154 - 158	157	I-->S
1DQPA	154 - 158	157	I-->T
1DQPA	154 - 158	158	K-->A
1DQPA	154 - 158	158	K-->C
1DQPA	154 - 158	158	K-->D
1DQPA	154 - 158	158	K-->F

1DQPA	154 - 158	158	K-->G	
1DQPA	154 - 158	158	K-->I	
1DQPA	154 - 158	158	K-->L	
1DQPA	154 - 158	158	K-->M	
1DQPA	154 - 158	158	K-->S	
1DQPA	154 - 158	158	K-->V	
1DQPA	154 - 158	158	K-->Y	
2D1GA	47 - 51	47	F-->G	
2D1GA	47 - 51	47	F-->L	
2D1GA	47 - 51	48	D-->P	
2D1GA	47 - 51	48	D-->V	
2D1GA	47 - 51	49	R-->E	
2D1GA	47 - 51	49	R-->F	
2D1GA	47 - 51	49	R-->I	
2D1GA	47 - 51	49	R-->K	
2D1GA	47 - 51	49	R-->L	
2D1GA	47 - 51	49	R-->T	
2D1GA	47 - 51	50	Y-->D	
2D1GA	47 - 51	50	Y-->I	
2D1GA	47 - 51	50	Y-->L	
2D1GA	47 - 51	50	Y-->S	
2D1GA	47 - 51	50	Y-->T	
2D1GA	47 - 51	50	Y-->V	
2D1GA	47 - 51	51	F-->H	
2D1GA	47 - 51	51	F-->R	
2D1GA	47 - 51	51	F-->Y	
2D1GA	80 - 84	80	K-->C	

2D1GA	80	- 84	80	K-->F
2D1GA	80	- 84	80	K-->G
2D1GA	80	- 84	80	K-->L
2D1GA	80	- 84	80	K-->V
2D1GA	80	- 84	80	K-->Y
2D1GA	80	- 84	81	Q-->E
2D1GA	80	- 84	81	Q-->G
2D1GA	80	- 84	81	Q-->H
2D1GA	80	- 84	81	Q-->L
2D1GA	80	- 84	81	Q-->M
2D1GA	80	- 84	81	Q-->P
2D1GA	80	- 84	81	Q-->R
2D1GA	80	- 84	81	Q-->S
2D1GA	80	- 84	81	Q-->T
2D1GA	80	- 84	82	L-->A
2D1GA	80	- 84	82	L-->G
2D1GA	80	- 84	82	L-->P
2D1GA	80	- 84	82	L-->Q
2D1GA	80	- 84	82	L-->R
2D1GA	80	- 84	82	L-->T
2D1GA	80	- 84	82	L-->V
2D1GA	80	- 84	82	L-->W
2D1GA	80	- 84	83	L-->A
2D1GA	80	- 84	83	L-->E
2D1GA	80	- 84	83	L-->G
2D1GA	80	- 84	83	L-->H
2D1GA	80	- 84	83	L-->I

2D1GA	80	84	83	L-->K
2D1GA	80	84	83	L-->P
2D1GA	80	84	84	E-->A
2D1GA	80	84	84	E-->C
2D1GA	80	84	84	E-->F
2D1GA	80	84	84	E-->L
2D1GA	80	84	84	E-->P
2D1GA	80	84	84	E-->T
2D1GA	80	84	84	E-->Y
2D1GA	123	127	123	F-->A
2D1GA	123	127	123	F-->D
2D1GA	123	127	123	F-->P
2D1GA	123	127	124	V-->D
2D1GA	123	127	124	V-->L
2D1GA	123	127	124	V-->M
2D1GA	123	127	125	E-->F
2D1GA	123	127	125	E-->L
2D1GA	123	127	126	H-->D
2D1GA	123	127	126	H-->E
2D1GA	123	127	126	H-->G
2D1GA	123	127	126	H-->I
2D1GA	123	127	126	H-->L
2D1GA	123	127	126	H-->M
2D1GA	123	127	126	H-->P
2D1GA	123	127	126	H-->T
2D1GA	123	127	126	H-->V
2D1GA	123	127	127	G-->D

2D1GA	123 - 127	127	G-->I
2D1GA	123 - 127	127	G-->P
2EX4A	75 - 79	75	R-->A
2EX4A	75 - 79	75	R-->I
2EX4A	75 - 79	75	R-->L
2EX4A	75 - 79	75	R-->T
2EX4A	75 - 79	76	I-->C
2EX4A	75 - 79	76	I-->K
2EX4A	75 - 79	76	I-->N
2EX4A	75 - 79	76	I-->Q
2EX4A	75 - 79	76	I-->R
2EX4A	75 - 79	76	I-->Y
2EX4A	75 - 79	77	T-->D
2EX4A	75 - 79	77	T-->N
2EX4A	75 - 79	77	T-->V
2EX4A	75 - 79	77	T-->Y
2EX4A	75 - 79	78	K-->A
2EX4A	75 - 79	78	K-->L
2EX4A	75 - 79	78	K-->R
2EX4A	75 - 79	78	K-->T
2EX4A	75 - 79	78	K-->V
2EX4A	75 - 79	78	K-->Y
2EX4A	75 - 79	79	R-->D
2EX4A	75 - 79	79	R-->E
2EX4A	75 - 79	79	R-->I
2EX4A	75 - 79	79	R-->M
2EX4A	75 - 79	79	R-->N

2EX4A	106 - 111	107	E-->N
2EX4A	106 - 111	108	E-->C
2EX4A	106 - 111	108	E-->N
2EX4A	106 - 111	109	G-->T
2ETEA	163 - 169	163	V-->G
2FK9A	26 - 30	26	R-->P
2FK9A	26 - 30	27	W-->D
2FK9A	26 - 30	27	W-->E
2FK9A	26 - 30	27	W-->I
2FK9A	26 - 30	27	W-->L
2FK9A	26 - 30	27	W-->P
2FK9A	26 - 30	27	W-->S
2FK9A	26 - 30	27	W-->T
2FK9A	26 - 30	27	W-->Y
2FK9A	26 - 30	29	L-->C
2FK9A	26 - 30	30	R-->E
2FK9A	26 - 30	30	R-->K
2FK9A	26 - 30	30	R-->Q
1ZUWA	119 - 123	119	E-->A
1ZUWA	119 - 123	119	E-->C
1ZUWA	119 - 123	119	E-->D
1ZUWA	119 - 123	119	E-->F
1ZUWA	119 - 123	119	E-->G
1ZUWA	119 - 123	119	E-->L
1ZUWA	119 - 123	119	E-->M
1ZUWA	119 - 123	119	E-->N
1ZUWA	119 - 123	119	E-->V

1ZUWA	119 - 123	120	N-->D
1ZUWA	119 - 123	120	N-->F
1ZUWA	119 - 123	120	N-->G
1ZUWA	119 - 123	120	N-->I
1ZUWA	119 - 123	120	N-->L
1ZUWA	119 - 123	120	N-->T
1ZUWA	119 - 123	120	N-->V
1ZUWA	119 - 123	121	T-->E
1ZUWA	119 - 123	121	T-->K
1ZUWA	119 - 123	121	T-->Q
1ZUWA	119 - 123	121	T-->S
1ZUWA	119 - 123	122	I-->F
1ZUWA	119 - 123	122	I-->G
1ZUWA	119 - 123	122	I-->L
1ZUWA	119 - 123	122	I-->P
1ZUWA	119 - 123	122	I-->R
1ZUWA	119 - 123	122	I-->S
1ZUWA	119 - 123	122	I-->V
1ZUWA	119 - 123	123	K-->G
1ZUWA	119 - 123	123	K-->I
1ZUWA	119 - 123	123	K-->N
1ZUWA	119 - 123	123	K-->R
1ZUWA	119 - 123	123	K-->S
1ZUWA	119 - 123	123	K-->T
1ZUWA	119 - 123	123	K-->Y
1ZUWA	148 - 152	148	L-->A
1ZUWA	148 - 152	148	L-->D

1ZUWA	148 - 152	148	L-->E
1ZUWA	148 - 152	148	L-->F
1ZUWA	148 - 152	148	L-->H
1ZUWA	148 - 152	148	L-->N
1ZUWA	148 - 152	148	L-->P
1ZUWA	148 - 152	148	L-->R
1ZUWA	148 - 152	148	L-->S
1ZUWA	148 - 152	148	L-->T
1ZUWA	148 - 152	149	V-->A
1ZUWA	148 - 152	149	V-->D
1ZUWA	148 - 152	149	V-->G
1ZUWA	148 - 152	149	V-->I
1ZUWA	148 - 152	149	V-->P
1ZUWA	148 - 152	149	V-->Y
1ZUWA	148 - 152	150	P-->A
1ZUWA	148 - 152	150	P-->C
1ZUWA	148 - 152	150	P-->D
1ZUWA	148 - 152	150	P-->F
1ZUWA	148 - 152	150	P-->G
1ZUWA	148 - 152	150	P-->I
1ZUWA	148 - 152	150	P-->K
1ZUWA	148 - 152	150	P-->L
1ZUWA	148 - 152	150	P-->M
1ZUWA	148 - 152	150	P-->Q
1ZUWA	148 - 152	150	P-->R
1ZUWA	148 - 152	150	P-->V
1ZUWA	148 - 152	151	F-->A

1ZUWA	148 - 152	151	F-->C
1ZUWA	148 - 152	151	F-->E
1ZUWA	148 - 152	151	F-->G
1ZUWA	148 - 152	151	F-->I
1ZUWA	148 - 152	151	F-->K
1ZUWA	148 - 152	151	F-->L
1ZUWA	148 - 152	151	F-->M
1ZUWA	148 - 152	151	F-->P
1ZUWA	148 - 152	151	F-->R
1ZUWA	148 - 152	151	F-->S
1ZUWA	148 - 152	151	F-->V
1ZUWA	148 - 152	151	F-->W
1ZUWA	148 - 152	151	F-->Y
1ZUWA	148 - 152	152	V-->G
1ZUWA	148 - 152	152	V-->I
1ZUWA	148 - 152	152	V-->L
1ZUWA	148 - 152	152	V-->S
1ZUWA	148 - 152	152	V-->T
1KPTA	9 - 13	9	S-->H
1KPTA	9 - 13	9	S-->W
1KPTA	9 - 13	10	Q-->L
1KPTA	9 - 13	10	Q-->S
1KPTA	9 - 13	10	Q-->W
1KPTA	9 - 13	11	C-->D
1KPTA	9 - 13	11	C-->E
1KPTA	9 - 13	11	C-->K
1KPTA	9 - 13	11	C-->L

1KPTA	9 - 13	11	C-->N
1KPTA	9 - 13	11	C-->P
1KPTA	9 - 13	11	C-->Y
1KPTA	9 - 13	12	G-->D
1KPTA	9 - 13	12	G-->L
1KPTA	9 - 13	13	L-->E
1KPTA	9 - 13	13	L-->F
1KPTA	9 - 13	13	L-->N
1MDCA	15 - 20	15	F-->T
1MDCA	15 - 20	18	F-->K
1MDCA	15 - 20	19	L-->R
1MDCA	15 - 20	20	K-->V
2I6HA	52 - 56	52	D-->E
2I6HA	52 - 56	52	D-->L
2I6HA	52 - 56	52	D-->T
2I6HA	52 - 56	53	G-->E
2I6HA	52 - 56	53	G-->N
2I6HA	52 - 56	54	W-->E
2I6HA	52 - 56	54	W-->G
2I6HA	52 - 56	54	W-->K
2I6HA	52 - 56	54	W-->N
2I6HA	52 - 56	54	W-->Q
2I6HA	52 - 56	54	W-->R
2I6HA	52 - 56	54	W-->S
2I6HA	52 - 56	54	W-->T
2I6HA	52 - 56	54	W-->V
2I6HA	52 - 56	54	W-->Y

2I6HA	52	-	56	55	L-->G
2I6HA	52	-	56	55	L-->K
2I6HA	52	-	56	55	L-->T
2I6HA	52	-	56	55	L-->V
2I6HA	52	-	56	56	E-->A
2I6HA	52	-	56	56	E-->F
2I6HA	52	-	56	56	E-->H
2I6HA	52	-	56	56	E-->T
2I6HA	58	-	64	59	E-->T
2I6HA	71	-	75	71	F-->G
2I6HA	71	-	75	71	F-->V
2I6HA	71	-	75	72	P-->I
2I6HA	71	-	75	72	P-->V
2I6HA	71	-	75	75	C-->A
2I6HA	71	-	75	75	C-->D
2I6HA	71	-	75	75	C-->F
2I6HA	71	-	75	75	C-->I
2I6HA	71	-	75	75	C-->K
2I6HA	71	-	75	75	C-->L
2I6HA	71	-	75	75	C-->S
2I6HA	71	-	75	75	C-->T
2I6HA	107	-	113	110	R-->G
3CLAA	42	-	49	42	T-->G
1VR8A	31	-	36	34	A-->N
1VR8A	31	-	36	35	I-->G
1VR8A	31	-	36	35	I-->R
1VR8A	31	-	36	36	F-->N

2I8GB	132	-	137	132	S-->Q
2I8GB	132	-	137	136	E-->P
3CK1A	24	-	29	24	Y-->D
3CK1A	24	-	29	24	Y-->W
3CK1A	24	-	29	25	P-->N
3CK1A	127	-	132	127	D-->L
3CK1A	127	-	132	128	P-->W
2P0BA	20	-	24	20	P-->M
2P0BA	20	-	24	20	P-->S
2P0BA	20	-	24	21	D-->A
2P0BA	20	-	24	21	D-->S
2P0BA	20	-	24	21	D-->V
2P0BA	20	-	24	22	A-->I
2P0BA	20	-	24	22	A-->K
2P0BA	20	-	24	22	A-->Q
2P0BA	20	-	24	22	A-->R
2P0BA	20	-	24	23	A-->H
2P0BA	20	-	24	23	A-->M
2P0BA	20	-	24	23	A-->N
2P0BA	20	-	24	24	C-->E
2P0BA	20	-	24	24	C-->F
2P0BA	20	-	24	24	C-->G
2P0BA	20	-	24	24	C-->I
2P0BA	20	-	24	24	C-->K
2P0BA	20	-	24	24	C-->L
2P0BA	20	-	24	24	C-->N
2P0BA	20	-	24	24	C-->T

2P0BA	20 - 24	24	C-->V
2P0BA	104 - 108	104	H-->A
2P0BA	104 - 108	104	H-->D
2P0BA	104 - 108	104	H-->E
2P0BA	104 - 108	104	H-->P
2P0BA	104 - 108	104	H-->Y
2P0BA	104 - 108	105	D-->E
2P0BA	104 - 108	105	D-->L
2P0BA	104 - 108	105	D-->Y
2P0BA	104 - 108	106	V-->K
2P0BA	104 - 108	107	H-->A
2P0BA	104 - 108	107	H-->D
2P0BA	104 - 108	107	H-->E
2P0BA	104 - 108	107	H-->G
2P0BA	104 - 108	107	H-->I
2P0BA	104 - 108	107	H-->L
2P0BA	104 - 108	107	H-->P
2P0BA	104 - 108	107	H-->Y
2P0BA	104 - 108	108	V-->A
2P0BA	104 - 108	108	V-->G
2P0BA	104 - 108	108	V-->T
2QS9B	76 - 81	76	S-->K
2QS9B	76 - 81	77	G-->Q
2QS9B	76 - 81	78	A-->I
2QS9B	76 - 81	79	I-->C
2QS9B	76 - 81	79	I-->L
2QS9B	76 - 81	79	I-->P

2QS9B	76 - 81	80	A-->I
2QS9B	76 - 81	81	A-->G
2QS9B	76 - 81	81	A-->Q
2QS9B	106 - 110	106	E-->G
2QS9B	106 - 110	107	N-->I
2QS9B	106 - 110	107	N-->K
2QS9B	106 - 110	107	N-->P
2QS9B	106 - 110	107	N-->V
2QS9B	106 - 110	108	E-->G
2QS9B	106 - 110	108	E-->L
2QS9B	106 - 110	108	E-->S
2QS9B	106 - 110	108	E-->T
2QS9B	106 - 110	108	E-->Y
2QS9B	106 - 110	109	R-->E
2QS9B	106 - 110	109	R-->G
2QS9B	106 - 110	109	R-->H
2QS9B	106 - 110	109	R-->M
2QS9B	106 - 110	110	A-->E
2QS9B	106 - 110	110	A-->H
2QS9B	106 - 110	110	A-->I
2QS9B	106 - 110	110	A-->K
2QS9B	106 - 110	110	A-->R
2QS9B	106 - 110	110	A-->W
1D0QA	19 - 23	19	I-->A
1D0QA	19 - 23	19	I-->D
1D0QA	19 - 23	19	I-->E
1D0QA	19 - 23	19	I-->F

1D0QA	19 - 23	19	I-->G
1D0QA	19 - 23	19	I-->K
1D0QA	19 - 23	19	I-->L
1D0QA	19 - 23	19	I-->N
1D0QA	19 - 23	19	I-->P
1D0QA	19 - 23	19	I-->Q
1D0QA	19 - 23	19	I-->R
1D0QA	19 - 23	19	I-->S
1D0QA	19 - 23	19	I-->T
1D0QA	19 - 23	19	I-->V
1D0QA	19 - 23	19	I-->Y
1D0QA	19 - 23	20	V-->A
1D0QA	19 - 23	20	V-->D
1D0QA	19 - 23	20	V-->I
1D0QA	19 - 23	20	V-->K
1D0QA	19 - 23	20	V-->N
1D0QA	19 - 23	20	V-->P
1D0QA	19 - 23	20	V-->R
1D0QA	19 - 23	20	V-->S
1D0QA	19 - 23	20	V-->T
1D0QA	19 - 23	20	V-->Y
1D0QA	19 - 23	21	D-->A
1D0QA	19 - 23	21	D-->H
1D0QA	19 - 23	21	D-->K
1D0QA	19 - 23	21	D-->L
1D0QA	19 - 23	21	D-->N
1D0QA	19 - 23	21	D-->P

1D0QA	19 - 23	21	D-->S
1D0QA	19 - 23	22	V-->A
1D0QA	19 - 23	22	V-->D
1D0QA	19 - 23	22	V-->H
1D0QA	19 - 23	22	V-->K
1D0QA	19 - 23	22	V-->P
1D0QA	19 - 23	23	I-->A
1D0QA	19 - 23	23	I-->D
1D0QA	19 - 23	23	I-->G
1D0QA	19 - 23	23	I-->K
1D0QA	19 - 23	23	I-->L
1D0QA	19 - 23	23	I-->N
1D0QA	19 - 23	23	I-->P
1D0QA	19 - 23	23	I-->R
1D0QA	19 - 23	23	I-->S
1D0QA	19 - 23	23	I-->T
1D0QA	19 - 23	23	I-->V
1D0QA	19 - 23	23	I-->Y
3BZLB	249 - 255	255	K-->E
1Z7AC	172 - 176	172	T-->A
1Z7AC	172 - 176	172	T-->E
1Z7AC	172 - 176	172	T-->G
1Z7AC	172 - 176	172	T-->L
1Z7AC	172 - 176	172	T-->N
1Z7AC	172 - 176	172	T-->P
1Z7AC	172 - 176	172	T-->Q
1Z7AC	172 - 176	172	T-->R

1Z7AC	172 - 176	172	T-->S
1Z7AC	172 - 176	172	T-->V
1Z7AC	172 - 176	172	T-->Y
1Z7AC	172 - 176	173	R-->A
1Z7AC	172 - 176	173	R-->G
1Z7AC	172 - 176	173	R-->K
1Z7AC	172 - 176	173	R-->L
1Z7AC	172 - 176	173	R-->N
1Z7AC	172 - 176	173	R-->P
1Z7AC	172 - 176	173	R-->T
1Z7AC	172 - 176	173	R-->V
1Z7AC	172 - 176	173	R-->Y
1Z7AC	172 - 176	174	R-->A
1Z7AC	172 - 176	174	R-->C
1Z7AC	172 - 176	174	R-->D
1Z7AC	172 - 176	174	R-->E
1Z7AC	172 - 176	174	R-->G
1Z7AC	172 - 176	174	R-->I
1Z7AC	172 - 176	174	R-->L
1Z7AC	172 - 176	174	R-->M
1Z7AC	172 - 176	174	R-->T
1Z7AC	172 - 176	174	R-->V
1Z7AC	172 - 176	175	L-->D
1Z7AC	172 - 176	175	L-->I
1Z7AC	172 - 176	175	L-->S
1Z7AC	172 - 176	175	L-->T
1Z7AC	172 - 176	175	L-->V

1Z7AC	172 - 176	176	V-->A
1Z7AC	172 - 176	176	V-->D
1Z7AC	172 - 176	176	V-->E
1Z7AC	172 - 176	176	V-->G
1Z7AC	172 - 176	176	V-->K
1Z7AC	172 - 176	176	V-->T
1Z7AC	172 - 176	176	V-->W
1TSFA	7 - 11	7	G-->D
1TSFA	7 - 11	7	G-->H
1TSFA	7 - 11	7	G-->K
1TSFA	7 - 11	7	G-->L
1TSFA	7 - 11	7	G-->P
1TSFA	7 - 11	7	G-->Q
1TSFA	7 - 11	7	G-->R
1TSFA	7 - 11	7	G-->S
1TSFA	7 - 11	7	G-->T
1TSFA	7 - 11	7	G-->Y
1TSFA	7 - 11	8	V-->C
1TSFA	7 - 11	8	V-->D
1TSFA	7 - 11	8	V-->E
1TSFA	7 - 11	8	V-->F
1TSFA	7 - 11	8	V-->G
1TSFA	7 - 11	8	V-->I
1TSFA	7 - 11	8	V-->K
1TSFA	7 - 11	8	V-->L
1TSFA	7 - 11	8	V-->N
1TSFA	7 - 11	8	V-->P

1TSFA	7 - 11	8	V-->S
1TSFA	7 - 11	8	V-->T
1TSFA	7 - 11	9	E-->A
1TSFA	7 - 11	9	E-->D
1TSFA	7 - 11	9	E-->F
1TSFA	7 - 11	9	E-->H
1TSFA	7 - 11	9	E-->I
1TSFA	7 - 11	9	E-->K
1TSFA	7 - 11	9	E-->L
1TSFA	7 - 11	9	E-->M
1TSFA	7 - 11	9	E-->P
1TSFA	7 - 11	9	E-->Q
1TSFA	7 - 11	9	E-->R
1TSFA	7 - 11	9	E-->T
1TSFA	7 - 11	9	E-->V
1TSFA	7 - 11	9	E-->W
1TSFA	7 - 11	10	L-->A
1TSFA	7 - 11	10	L-->C
1TSFA	7 - 11	10	L-->D
1TSFA	7 - 11	10	L-->E
1TSFA	7 - 11	10	L-->F
1TSFA	7 - 11	10	L-->G
1TSFA	7 - 11	10	L-->H
1TSFA	7 - 11	10	L-->I
1TSFA	7 - 11	10	L-->K
1TSFA	7 - 11	10	L-->N
1TSFA	7 - 11	10	L-->P

1TSFA	7 - 11	10	L-->Q
1TSFA	7 - 11	10	L-->R
1TSFA	7 - 11	10	L-->S
1TSFA	7 - 11	10	L-->V
1TSFA	7 - 11	11	I-->A
1TSFA	7 - 11	11	I-->D
1TSFA	7 - 11	11	I-->E
1TSFA	7 - 11	11	I-->F
1TSFA	7 - 11	11	I-->K
1TSFA	7 - 11	11	I-->L
1TSFA	7 - 11	11	I-->N
1TSFA	7 - 11	11	I-->P
1TSFA	7 - 11	11	I-->R
1TSFA	7 - 11	11	I-->T
1TSFA	7 - 11	11	I-->V
1TSFA	7 - 11	11	I-->W
1TSFA	7 - 11	11	I-->Y
1QSTA	166 - 171	171	K-->N
1QSTA	166 - 171	171	K-->S
1JUVA	53 - 57	53	A-->D
1JUVA	53 - 57	53	A-->G
1JUVA	53 - 57	53	A-->P
1JUVA	53 - 57	54	K-->A
1JUVA	53 - 57	54	K-->I
1JUVA	53 - 57	54	K-->N
1JUVA	53 - 57	54	K-->T
1JUVA	53 - 57	55	T-->V

1JUVA	53	57	56	F-->C
1JUVA	53	57	56	F-->E
1JUVA	53	57	56	F-->G
1JUVA	53	57	56	F-->R
1JUVA	53	57	57	Q-->E
1JUVA	53	57	57	Q-->L
1JUVA	53	57	57	Q-->P
1JUVA	53	57	57	Q-->T
3CG6A	98	104	99	Q-->I
2F37B	212	218	217	A-->L
2F37B	248	253	249	L-->R
2F37B	248	253	252	L-->N
2F37B	248	253	253	V-->E
2F37B	297	303	299	K-->T
2O99A	66	72	70	A-->Y
2O99A	77	81	77	E-->A
2O99A	77	81	77	E-->N
2O99A	77	81	77	E-->P
2O99A	77	81	77	E-->Q
2O99A	77	81	77	E-->V
2O99A	77	81	78	E-->C
2O99A	77	81	78	E-->F
2O99A	77	81	78	E-->H
2O99A	77	81	78	E-->I
2O99A	77	81	78	E-->K
2O99A	77	81	78	E-->M
2O99A	77	81	78	E-->P

2O99A	77	81	78	E-->Q
2O99A	77	81	78	E-->V
2O99A	77	81	79	Q-->A
2O99A	77	81	79	Q-->D
2O99A	77	81	79	Q-->E
2O99A	77	81	79	Q-->G
2O99A	77	81	79	Q-->I
2O99A	77	81	79	Q-->K
2O99A	77	81	79	Q-->N
2O99A	77	81	79	Q-->P
2O99A	77	81	79	Q-->T
2O99A	77	81	79	Q-->V
2O99A	77	81	80	V-->E
2O99A	77	81	80	V-->K
2O99A	77	81	80	V-->L
2O99A	77	81	80	V-->Q
2O99A	77	81	81	T-->N
2O99A	77	81	81	T-->P
2JGBA	217	222	217	H-->P
2VLGD	34	38	34	S-->D
2VLGD	34	38	34	S-->F
2VLGD	34	38	34	S-->G
2VLGD	34	38	34	S-->K
2VLGD	34	38	34	S-->M
2VLGD	34	38	34	S-->N
2VLGD	34	38	34	S-->T
2VLGD	34	38	35	K-->A

2VLGD	34 - 38	35	K-->E
2VLGD	34 - 38	35	K-->H
2VLGD	34 - 38	35	K-->L
2VLGD	34 - 38	35	K-->N
2VLGD	34 - 38	35	K-->T
2VLGD	34 - 38	35	K-->V
2VLGD	34 - 38	36	L-->F
2VLGD	34 - 38	36	L-->G
2VLGD	34 - 38	37	H-->A
2VLGD	34 - 38	37	H-->E
2VLGD	34 - 38	37	H-->G
2VLGD	34 - 38	37	H-->I
2VLGD	34 - 38	37	H-->N
2VLGD	34 - 38	37	H-->P
2VLGD	34 - 38	37	H-->Q
2VLGD	34 - 38	37	H-->R
2VLGD	34 - 38	37	H-->S
2VLGD	34 - 38	37	H-->T
2VLGD	34 - 38	37	H-->V
2VLGD	34 - 38	38	L-->P
2VLGD	34 - 38	38	L-->S
1S5UG	126 - 131	126	K-->N
1S5UG	126 - 131	127	S-->V
1S5UG	126 - 131	129	V-->N
1S5UG	126 - 131	129	V-->Q
1S5UG	126 - 131	131	E-->G
1N0WB	1536 - 1541	1537	E-->D

1N0WB	1536	1541	1537	E-->K
1N0WB	1536	1541	1537	E-->Q
1PFVA	276	281	279	E-->F
1U5DA	172	176	172	S-->A
1U5DA	172	176	172	S-->C
1U5DA	172	176	172	S-->E
1U5DA	172	176	172	S-->I
1U5DA	172	176	173	K-->A
1U5DA	172	176	173	K-->F
1U5DA	172	176	173	K-->G
1U5DA	172	176	173	K-->H
1U5DA	172	176	173	K-->L
1U5DA	172	176	173	K-->P
1U5DA	172	176	173	K-->Q
1U5DA	172	176	173	K-->R
1U5DA	172	176	173	K-->S
1U5DA	172	176	173	K-->V
1U5DA	172	176	174	K-->A
1U5DA	172	176	174	K-->D
1U5DA	172	176	174	K-->L
1U5DA	172	176	175	E-->F
1U5DA	172	176	175	E-->G
1U5DA	172	176	175	E-->I
1U5DA	172	176	175	E-->K
1U5DA	172	176	175	E-->L
1U5DA	172	176	175	E-->M
1U5DA	172	176	175	E-->N

1U5DA	172 - 176	175	E-->P	
1U5DA	172 - 176	175	E-->R	
1U5DA	172 - 176	175	E-->T	
1U5DA	172 - 176	175	E-->V	
1U5DA	172 - 176	175	E-->Y	
1U5DA	172 - 176	176	S-->F	
1U5DA	172 - 176	176	S-->H	
1U5DA	172 - 176	176	S-->I	
1U5DA	172 - 176	176	S-->Q	
1U5DA	172 - 176	176	S-->V	
2I5IA	49 - 54	54	S-->Q	
2I5IA	219 - 224	220	I-->G	
2I5IA	219 - 224	220	I-->N	
2I5IA	219 - 224	220	I-->V	
2I5IA	219 - 224	221	L-->T	
2QIYB	73 - 77	73	D-->E	
2QIYB	73 - 77	73	D-->G	
2QIYB	73 - 77	73	D-->L	
2QIYB	73 - 77	73	D-->P	
2QIYB	73 - 77	73	D-->S	
2QIYB	73 - 77	73	D-->T	
2QIYB	73 - 77	73	D-->V	
2QIYB	73 - 77	74	A-->D	
2QIYB	73 - 77	74	A-->F	
2QIYB	73 - 77	74	A-->G	
2QIYB	73 - 77	74	A-->H	
2QIYB	73 - 77	74	A-->K	

2QIYB	73	77	74	A-->L
2QIYB	73	77	74	A-->P
2QIYB	73	77	74	A-->Q
2QIYB	73	77	74	A-->S
2QIYB	73	77	74	A-->V
2QIYB	73	77	74	A-->Y
2QIYB	73	77	75	K-->F
2QIYB	73	77	75	K-->G
2QIYB	73	77	75	K-->I
2QIYB	73	77	75	K-->L
2QIYB	73	77	75	K-->S
2QIYB	73	77	75	K-->T
2QIYB	73	77	75	K-->Y
2QIYB	73	77	76	V-->A
2QIYB	73	77	76	V-->G
2QIYB	73	77	76	V-->N
2QIYB	73	77	76	V-->T
2QIYB	73	77	76	V-->Y
2QIYB	73	77	77	R-->D
2QIYB	73	77	77	R-->E
2QIYB	73	77	77	R-->I
2QIYB	73	77	77	R-->M
2QIYB	73	77	77	R-->P
2QIYB	73	77	77	R-->S
2QIYB	73	77	77	R-->T
2QIYB	73	77	77	R-->V
2A15A	25	30	26	E-->D

2A15A	25 - 30	29	L-->R	
2A15A	25 - 30	30	A-->L	
2AJ7B	77 - 82	77	E-->A	
2AJ7B	77 - 82	82	Q-->I	
2ISBA	13 - 18	13	K-->T	
2ISBA	13 - 18	16	I-->P	
2ISBA	111 - 116	111	E-->A	
2ISBA	111 - 116	112	E-->V	
2ISBA	111 - 116	114	V-->E	
2ISBA	111 - 116	114	V-->L	
2ISBA	111 - 116	115	E-->H	
2OITA	125 - 129	125	V-->D	
2OITA	125 - 129	125	V-->E	
2OITA	125 - 129	125	V-->G	
2OITA	125 - 129	125	V-->H	
2OITA	125 - 129	125	V-->I	
2OITA	125 - 129	125	V-->N	
2OITA	125 - 129	125	V-->R	
2OITA	125 - 129	126	R-->A	
2OITA	125 - 129	126	R-->E	
2OITA	125 - 129	126	R-->G	
2OITA	125 - 129	126	R-->M	
2OITA	125 - 129	126	R-->N	
2OITA	125 - 129	126	R-->P	
2OITA	125 - 129	126	R-->Q	
2OITA	125 - 129	126	R-->S	
2OITA	125 - 129	126	R-->Y	

2OITA	125 - 129	127	T-->C
2OITA	125 - 129	127	T-->E
2OITA	125 - 129	127	T-->G
2OITA	125 - 129	127	T-->K
2OITA	125 - 129	127	T-->N
2OITA	125 - 129	128	F-->A
2OITA	125 - 129	128	F-->D
2OITA	125 - 129	128	F-->E
2OITA	125 - 129	128	F-->H
2OITA	125 - 129	128	F-->I
2OITA	125 - 129	128	F-->K
2OITA	125 - 129	128	F-->L
2OITA	125 - 129	128	F-->V
2OITA	125 - 129	128	F-->Y
2OITA	125 - 129	129	S-->A
2OITA	125 - 129	129	S-->P
2OITA	125 - 129	129	S-->Q
1CXYA	58 - 63	58	T-->G
3BJNA	142 - 147	143	A-->G
3BJNA	142 - 147	144	S-->D
3BJNA	142 - 147	144	S-->P
3BJNA	142 - 147	145	S-->V
3BJNA	142 - 147	145	S-->Y
3BJNA	142 - 147	146	K-->R
1SBXA	123 - 127	123	L-->A
1SBXA	123 - 127	123	L-->D
1SBXA	123 - 127	123	L-->H

1SBXA	123 - 127	123	L-->N
1SBXA	123 - 127	123	L-->Q
1SBXA	123 - 127	123	L-->T
1SBXA	123 - 127	123	L-->Y
1SBXA	123 - 127	124	P-->S
1SBXA	123 - 127	125	Q-->C
1SBXA	123 - 127	125	Q-->D
1SBXA	123 - 127	125	Q-->E
1SBXA	123 - 127	125	Q-->I
1SBXA	123 - 127	125	Q-->L
1SBXA	123 - 127	125	Q-->M
1SBXA	123 - 127	125	Q-->N
1SBXA	123 - 127	125	Q-->P
1SBXA	123 - 127	125	Q-->R
1SBXA	123 - 127	125	Q-->S
1SBXA	123 - 127	125	Q-->T
1SBXA	123 - 127	125	Q-->V
1SBXA	123 - 127	125	Q-->Y
1SBXA	123 - 127	126	I-->F
1SBXA	123 - 127	126	I-->G
1SBXA	123 - 127	126	I-->H
1SBXA	123 - 127	126	I-->K
1SBXA	123 - 127	126	I-->L
1SBXA	123 - 127	126	I-->M
1SBXA	123 - 127	126	I-->N
1SBXA	123 - 127	126	I-->P
1SBXA	123 - 127	126	I-->S

1SBXA	123	127	126	I-->V
1SBXA	123	127	127	L-->E
1SBXA	123	127	127	L-->P
1SBXA	123	127	127	L-->S
1SBXA	123	127	127	L-->V
2QRUA	103	107	103	A-->D
2QRUA	103	107	103	A-->E
2QRUA	103	107	103	A-->G
2QRUA	103	107	103	A-->H
2QRUA	103	107	103	A-->I
2QRUA	103	107	103	A-->K
2QRUA	103	107	103	A-->L
2QRUA	103	107	103	A-->N
2QRUA	103	107	103	A-->R
2QRUA	103	107	103	A-->S
2QRUA	103	107	103	A-->T
2QRUA	103	107	103	A-->V
2QRUA	103	107	104	G-->D
2QRUA	103	107	104	G-->E
2QRUA	103	107	104	G-->K
2QRUA	103	107	104	G-->L
2QRUA	103	107	104	G-->N
2QRUA	103	107	104	G-->Q
2QRUA	103	107	104	G-->R
2QRUA	103	107	104	G-->S
2QRUA	103	107	104	G-->V
2QRUA	103	107	105	G-->D

2QRUA	103	107	105	G-->E
2QRUA	103	107	105	G-->F
2QRUA	103	107	105	G-->K
2QRUA	103	107	105	G-->L
2QRUA	103	107	105	G-->M
2QRUA	103	107	105	G-->P
2QRUA	103	107	105	G-->Q
2QRUA	103	107	105	G-->R
2QRUA	103	107	105	G-->S
2QRUA	103	107	105	G-->T
2QRUA	103	107	105	G-->V
2QRUA	103	107	105	G-->Y
2QRUA	103	107	106	Y-->A
2QRUA	103	107	106	Y-->D
2QRUA	103	107	106	Y-->E
2QRUA	103	107	106	Y-->G
2QRUA	103	107	106	Y-->H
2QRUA	103	107	106	Y-->K
2QRUA	103	107	106	Y-->L
2QRUA	103	107	106	Y-->M
2QRUA	103	107	106	Y-->N
2QRUA	103	107	106	Y-->P
2QRUA	103	107	106	Y-->Q
2QRUA	103	107	106	Y-->R
2QRUA	103	107	106	Y-->S
2QRUA	103	107	106	Y-->T
2QRUA	103	107	106	Y-->V

2QRUA	103 - 107	106	Y-->W
2QRUA	103 - 107	107	L-->A
2QRUA	103 - 107	107	L-->C
2QRUA	103 - 107	107	L-->D
2QRUA	103 - 107	107	L-->E
2QRUA	103 - 107	107	L-->F
2QRUA	103 - 107	107	L-->G
2QRUA	103 - 107	107	L-->I
2QRUA	103 - 107	107	L-->K
2QRUA	103 - 107	107	L-->P
2QRUA	103 - 107	107	L-->R
2QRUA	103 - 107	107	L-->S
2QRUA	103 - 107	107	L-->T
2QRUA	103 - 107	107	L-->V
2QRUA	103 - 107	107	L-->Y
2QRUA	183 - 187	184	P-->A
2QRUA	183 - 187	185	H-->E
2QRUA	183 - 187	185	H-->F
2QRUA	183 - 187	185	H-->G
2QRUA	183 - 187	185	H-->I
2QRUA	183 - 187	185	H-->L
2QRUA	183 - 187	185	H-->Q
2QRUA	183 - 187	185	H-->V
2QRUA	183 - 187	186	F-->E
2QRUA	183 - 187	186	F-->R
2QRUA	183 - 187	186	F-->S
2QRUA	183 - 187	187	Y-->N

2QRUA	183 - 187	187	Y-->P
2QRUA	183 - 187	187	Y-->S
2QRUA	183 - 187	187	Y-->T
2QRUA	202 - 206	202	D-->A
2QRUA	202 - 206	202	D-->F
2QRUA	202 - 206	202	D-->I
2QRUA	202 - 206	202	D-->K
2QRUA	202 - 206	202	D-->P
2QRUA	202 - 206	202	D-->S
2QRUA	202 - 206	202	D-->T
2QRUA	202 - 206	202	D-->V
2QRUA	202 - 206	203	E-->C
2QRUA	202 - 206	203	E-->D
2QRUA	202 - 206	203	E-->F
2QRUA	202 - 206	203	E-->G
2QRUA	202 - 206	203	E-->H
2QRUA	202 - 206	203	E-->I
2QRUA	202 - 206	203	E-->K
2QRUA	202 - 206	203	E-->P
2QRUA	202 - 206	203	E-->Q
2QRUA	202 - 206	203	E-->V
2QRUA	202 - 206	203	E-->Y
2QRUA	202 - 206	204	T-->C
2QRUA	202 - 206	204	T-->E
2QRUA	202 - 206	204	T-->K
2QRUA	202 - 206	204	T-->N
2QRUA	202 - 206	204	T-->Q

2QRUA	202	- 206	204	T-->S
2QRUA	202	- 206	204	T-->V
2QRUA	202	- 206	205	L-->A
2QRUA	202	- 206	205	L-->G
2QRUA	202	- 206	205	L-->I
2QRUA	202	- 206	205	L-->K
2QRUA	202	- 206	205	L-->S
2QRUA	202	- 206	205	L-->T
2QRUA	202	- 206	206	K-->A
2QRUA	202	- 206	206	K-->D
2QRUA	202	- 206	206	K-->F
2QRUA	202	- 206	206	K-->G
2QRUA	202	- 206	206	K-->I
2QRUA	202	- 206	206	K-->L
2QRUA	202	- 206	206	K-->M
2QRUA	202	- 206	206	K-->T
2QRUA	202	- 206	206	K-->V
1X3KA	40	- 45	45	K-->S
2AJ6A	44	- 49	44	I-->G
2I6VA	247	- 252	250	F-->A
2I6VA	247	- 252	250	F-->P
2I6VA	247	- 252	251	E-->I
1XWWA	79	- 84	79	K-->P
1XWWA	79	- 84	79	K-->Q
1XWWA	79	- 84	81	D-->K
1XWWA	79	- 84	84	T-->F
1HTRP	23	- 27	23	L-->A

1HTRP	23 - 27	23	L-->D
1HTRP	23 - 27	23	L-->E
1HTRP	23 - 27	23	L-->K
1HTRP	23 - 27	23	L-->M
1HTRP	23 - 27	23	L-->N
1HTRP	23 - 27	23	L-->P
1HTRP	23 - 27	23	L-->Q
1HTRP	23 - 27	23	L-->R
1HTRP	23 - 27	23	L-->S
1HTRP	23 - 27	23	L-->T
1HTRP	23 - 27	23	L-->W
1HTRP	23 - 27	24	G-->D
1HTRP	23 - 27	24	G-->P
1HTRP	23 - 27	24	G-->Q
1HTRP	23 - 27	24	G-->S
1HTRP	23 - 27	25	E-->A
1HTRP	23 - 27	25	E-->F
1HTRP	23 - 27	25	E-->G
1HTRP	23 - 27	25	E-->I
1HTRP	23 - 27	25	E-->P
1HTRP	23 - 27	25	E-->R
1HTRP	23 - 27	26	F-->D
1HTRP	23 - 27	26	F-->E
1HTRP	23 - 27	26	F-->G
1HTRP	23 - 27	26	F-->I
1HTRP	23 - 27	26	F-->K
1HTRP	23 - 27	26	F-->P

1HTRP	23 - 27	26	F-->Q	
1HTRP	23 - 27	26	F-->R	
1HTRP	23 - 27	26	F-->S	
1HTRP	23 - 27	26	F-->T	
1HTRP	23 - 27	26	F-->V	
1HTRP	23 - 27	27	L-->A	
1HTRP	23 - 27	27	L-->D	
1HTRP	23 - 27	27	L-->E	
1HTRP	23 - 27	27	L-->G	
1HTRP	23 - 27	27	L-->H	
1HTRP	23 - 27	27	L-->I	
1HTRP	23 - 27	27	L-->K	
1HTRP	23 - 27	27	L-->N	
1HTRP	23 - 27	27	L-->Q	
1HTRP	23 - 27	27	L-->S	
1HTRP	23 - 27	27	L-->T	
1HTRP	34 - 38	34	P-->A	
1HTRP	34 - 38	34	P-->R	
1HTRP	34 - 38	35	A-->I	
1HTRP	34 - 38	36	W-->D	
1HTRP	34 - 38	36	W-->I	
1HTRP	34 - 38	36	W-->K	
1HTRP	34 - 38	36	W-->T	
1HTRP	34 - 38	37	K-->E	
1HTRP	34 - 38	37	K-->G	
1HTRP	34 - 38	37	K-->S	
1HTRP	34 - 38	38	Y-->G	

1HTRP	34 - 38	38	Y-->P
1HTRP	34 - 38	38	Y-->Q
1VJFA	72 - 76	72	L-->A
1VJFA	72 - 76	72	L-->H
1VJFA	72 - 76	72	L-->I
1VJFA	72 - 76	72	L-->M
1VJFA	72 - 76	72	L-->P
1VJFA	72 - 76	73	H-->G
1VJFA	72 - 76	73	H-->K
1VJFA	72 - 76	73	H-->L
1VJFA	72 - 76	73	H-->N
1VJFA	72 - 76	73	H-->P
1VJFA	72 - 76	74	H-->I
1VJFA	72 - 76	74	H-->K
1VJFA	72 - 76	74	H-->P
1VJFA	72 - 76	74	H-->T
1VJFA	72 - 76	74	H-->V
1VJFA	72 - 76	75	V-->D
1VJFA	72 - 76	76	I-->A
1VJFA	72 - 76	76	I-->D
1VJFA	72 - 76	76	I-->E
1VJFA	72 - 76	76	I-->H
1VJFA	72 - 76	76	I-->P
1VJFA	72 - 76	76	I-->S
1VJFA	102 - 107	102	A-->E
1VJFA	102 - 107	102	A-->Q
1VJFA	102 - 107	103	F-->E

1DJ7A	63	69	65	A-->V
1DJ7A	77	82	79	M-->A
1RO2A	72	76	72	W-->E
1RO2A	72	76	72	W-->K
1RO2A	72	76	72	W-->P
1RO2A	72	76	72	W-->T
1RO2A	72	76	73	Q-->K
1RO2A	72	76	73	Q-->S
1RO2A	72	76	75	Y-->C
1RO2A	72	76	75	Y-->I
1IUJB	69	74	70	A-->F
1IUJB	69	74	70	A-->K
1IUJB	69	74	70	A-->T
1IUJB	69	74	71	F-->L
1IUJB	69	74	72	K-->V
1IUJB	69	74	73	E-->P
3CMBB	42	46	42	R-->A
3CMBB	42	46	42	R-->D
3CMBB	42	46	42	R-->E
3CMBB	42	46	42	R-->G
3CMBB	42	46	42	R-->P
3CMBB	42	46	42	R-->S
3CMBB	42	46	42	R-->T
3CMBB	42	46	42	R-->V
3CMBB	42	46	43	D-->A
3CMBB	42	46	43	D-->F
3CMBB	42	46	43	D-->I

3CMBB	42	46	43	D-->K
3CMBB	42	46	43	D-->L
3CMBB	42	46	43	D-->N
3CMBB	42	46	43	D-->S
3CMBB	42	46	43	D-->T
3CMBB	42	46	43	D-->V
3CMBB	42	46	44	L-->A
3CMBB	42	46	44	L-->D
3CMBB	42	46	44	L-->E
3CMBB	42	46	44	L-->F
3CMBB	42	46	44	L-->N
3CMBB	42	46	44	L-->P
3CMBB	42	46	44	L-->R
3CMBB	42	46	44	L-->S
3CMBB	42	46	44	L-->T
3CMBB	42	46	45	L-->I
3CMBB	42	46	45	L-->K
3CMBB	42	46	45	L-->P
3CMBB	42	46	45	L-->S
3CMBB	42	46	46	E-->A
3CMBB	42	46	46	E-->C
3CMBB	42	46	46	E-->D
3CMBB	42	46	46	E-->F
3CMBB	42	46	46	E-->N
3CMBB	42	46	46	E-->P
3CMBB	42	46	46	E-->T
3CMBB	42	46	46	E-->V

1TVGA	27 - 31	27	P-->A
1TVGA	27 - 31	27	P-->E
1TVGA	27 - 31	27	P-->H
1TVGA	27 - 31	27	P-->M
1TVGA	27 - 31	27	P-->R
1TVGA	27 - 31	27	P-->T
1TVGA	27 - 31	28	E-->A
1TVGA	27 - 31	28	E-->D
1TVGA	27 - 31	28	E-->F
1TVGA	27 - 31	28	E-->I
1TVGA	27 - 31	28	E-->K
1TVGA	27 - 31	28	E-->N
1TVGA	27 - 31	28	E-->Q
1TVGA	27 - 31	28	E-->S
1TVGA	27 - 31	28	E-->T
1TVGA	27 - 31	28	E-->V
1TVGA	27 - 31	29	N-->E
1TVGA	27 - 31	29	N-->F
1TVGA	27 - 31	29	N-->G
1TVGA	27 - 31	29	N-->I
1TVGA	27 - 31	29	N-->L
1TVGA	27 - 31	29	N-->R
1TVGA	27 - 31	29	N-->S
1TVGA	27 - 31	30	I-->A
1TVGA	27 - 31	30	I-->C
1TVGA	27 - 31	30	I-->G
1TVGA	27 - 31	30	I-->H

1TVGA	27	-	31	30	I-->K
1TVGA	27	-	31	30	I-->L
1TVGA	27	-	31	30	I-->P
1TVGA	27	-	31	30	I-->Q
1TVGA	27	-	31	30	I-->R
1TVGA	27	-	31	30	I-->S
1TVGA	27	-	31	31	I-->D
1TVGA	27	-	31	31	I-->K
1TVGA	27	-	31	31	I-->L
1TVGA	27	-	31	31	I-->M
1TVGA	27	-	31	31	I-->P
1TVGA	27	-	31	31	I-->T
1TVGA	27	-	31	31	I-->V
1IT2A	46	-	51	46	P-->E
1IT2A	46	-	51	47	Q-->V
1IT2A	46	-	51	50	A-->G
2D3DA	485	-	490	485	D-->A
2D3DA	485	-	490	486	E-->K
2D3DA	485	-	490	486	E-->V
2D3DA	485	-	490	487	T-->L
2D3DA	485	-	490	487	T-->S
1O26A	55	-	59	55	T-->I
1O26A	55	-	59	55	T-->Q
1O26A	55	-	59	55	T-->S
1O26A	55	-	59	55	T-->W
1O26A	55	-	59	55	T-->Y
1O26A	55	-	59	56	P-->D

1O26A	55 - 59	56	P-->E
1O26A	55 - 59	56	P-->Q
1O26A	55 - 59	56	P-->T
1O26A	55 - 59	57	F-->E
1O26A	55 - 59	57	F-->G
1O26A	55 - 59	57	F-->I
1O26A	55 - 59	57	F-->K
1O26A	55 - 59	57	F-->L
1O26A	55 - 59	57	F-->V
1O26A	55 - 59	58	E-->G
1O26A	55 - 59	58	E-->K
1O26A	55 - 59	58	E-->P
1O26A	55 - 59	59	H-->D
1O26A	55 - 59	59	H-->K
1O26A	55 - 59	59	H-->L
1O26A	55 - 59	59	H-->Q
1O26A	55 - 59	59	H-->R
1O26A	143 - 149	144	E-->I
2YWIA	119 - 124	119	E-->V
2YWIA	119 - 124	122	K-->W
2YWIA	119 - 124	123	A-->Y
2CIUA	120 - 125	120	K-->N
2CIUA	120 - 125	121	D-->A
2CIUA	120 - 125	121	D-->T
2CIUA	120 - 125	122	I-->P
2CIUA	120 - 125	124	S-->N
2CIUA	120 - 125	125	L-->Y

1G8QA	181 - 185	181	I-->D
1G8QA	181 - 185	181	I-->H
1G8QA	181 - 185	181	I-->K
1G8QA	181 - 185	181	I-->M
1G8QA	181 - 185	181	I-->N
1G8QA	181 - 185	181	I-->P
1G8QA	181 - 185	181	I-->Q
1G8QA	181 - 185	181	I-->S
1G8QA	181 - 185	181	I-->T
1G8QA	181 - 185	181	I-->V
1G8QA	181 - 185	182	I-->A
1G8QA	181 - 185	182	I-->E
1G8QA	181 - 185	182	I-->G
1G8QA	181 - 185	182	I-->L
1G8QA	181 - 185	182	I-->N
1G8QA	181 - 185	182	I-->P
1G8QA	181 - 185	182	I-->T
1G8QA	181 - 185	182	I-->V
1G8QA	181 - 185	183	S-->D
1G8QA	181 - 185	183	S-->F
1G8QA	181 - 185	183	S-->I
1G8QA	181 - 185	183	S-->K
1G8QA	181 - 185	183	S-->L
1G8QA	181 - 185	183	S-->N
1G8QA	181 - 185	183	S-->Q
1G8QA	181 - 185	183	S-->T
1G8QA	181 - 185	184	N-->D

1G8QA	181 - 185	184	N-->E
1G8QA	181 - 185	184	N-->G
1G8QA	181 - 185	184	N-->L
1G8QA	181 - 185	184	N-->S
1G8QA	181 - 185	184	N-->T
1G8QA	181 - 185	185	L-->A
1G8QA	181 - 185	185	L-->D
1G8QA	181 - 185	185	L-->E
1G8QA	181 - 185	185	L-->I
1G8QA	181 - 185	185	L-->K
1G8QA	181 - 185	185	L-->M
1G8QA	181 - 185	185	L-->P
1G8QA	181 - 185	185	L-->R
1G8QA	181 - 185	185	L-->T
1NC5A	338 - 342	338	Y-->D
1NC5A	338 - 342	338	Y-->G
1NC5A	338 - 342	338	Y-->L
1NC5A	338 - 342	338	Y-->N
1NC5A	338 - 342	338	Y-->S
1NC5A	338 - 342	339	D-->E
1NC5A	338 - 342	340	Y-->K
1NC5A	338 - 342	340	Y-->L
1NC5A	338 - 342	340	Y-->N
1NC5A	338 - 342	340	Y-->R
1NC5A	338 - 342	341	Y-->A
1NC5A	338 - 342	341	Y-->D
1NC5A	338 - 342	341	Y-->K

1NC5A	338	342	341	Y-->L
1NC5A	338	342	342	V-->A
1NC5A	338	342	342	V-->C
1NC5A	338	342	342	V-->E
1NC5A	338	342	342	V-->G
1NC5A	338	342	342	V-->I
1NC5A	338	342	342	V-->Q
2CXAA	35	39	35	P-->A
2CXAA	35	39	35	P-->D
2CXAA	35	39	35	P-->E
2CXAA	35	39	35	P-->G
2CXAA	35	39	35	P-->K
2CXAA	35	39	35	P-->N
2CXAA	35	39	35	P-->Q
2CXAA	35	39	35	P-->R
2CXAA	35	39	35	P-->S
2CXAA	35	39	35	P-->T
2CXAA	35	39	35	P-->V
2CXAA	35	39	36	A-->E
2CXAA	35	39	36	A-->H
2CXAA	35	39	36	A-->K
2CXAA	35	39	36	A-->L
2CXAA	35	39	36	A-->M
2CXAA	35	39	36	A-->N
2CXAA	35	39	36	A-->P
2CXAA	35	39	36	A-->S
2CXAA	35	39	36	A-->T

2CXAA	35 - 39	36	A-->V
2CXAA	35 - 39	37	R-->A
2CXAA	35 - 39	37	R-->D
2CXAA	35 - 39	37	R-->E
2CXAA	35 - 39	37	R-->G
2CXAA	35 - 39	37	R-->H
2CXAA	35 - 39	37	R-->K
2CXAA	35 - 39	37	R-->L
2CXAA	35 - 39	37	R-->N
2CXAA	35 - 39	37	R-->P
2CXAA	35 - 39	37	R-->T
2CXAA	35 - 39	37	R-->V
2CXAA	35 - 39	38	L-->A
2CXAA	35 - 39	38	L-->E
2CXAA	35 - 39	38	L-->G
2CXAA	35 - 39	38	L-->M
2CXAA	35 - 39	38	L-->N
2CXAA	35 - 39	38	L-->R
2CXAA	35 - 39	38	L-->S
2CXAA	35 - 39	38	L-->V
2CXAA	35 - 39	39	L-->A
2CXAA	35 - 39	39	L-->D
2CXAA	35 - 39	39	L-->E
2CXAA	35 - 39	39	L-->I
2CXAA	35 - 39	39	L-->K
2CXAA	35 - 39	39	L-->R
2CXAA	35 - 39	39	L-->S

2CXAA	35 - 39	39	L-->V
2CXAA	35 - 39	39	L-->Y
2CXAA	79 - 83	80	R-->I
2CXAA	79 - 83	80	R-->N
2CXAA	79 - 83	80	R-->Q
2CXAA	79 - 83	81	F-->R
2CXAA	79 - 83	81	F-->S
2CXAA	79 - 83	82	H-->A
2CXAA	79 - 83	82	H-->F
2CXAA	79 - 83	82	H-->I
2CXAA	79 - 83	82	H-->L
2CXAA	79 - 83	82	H-->V
2CXAA	79 - 83	83	K-->H
2CXAA	79 - 83	83	K-->Y
2CXAA	192 - 196	192	D-->A
2CXAA	192 - 196	192	D-->F
2CXAA	192 - 196	192	D-->K
2CXAA	192 - 196	192	D-->V
2CXAA	192 - 196	193	H-->D
2CXAA	192 - 196	193	H-->E
2CXAA	192 - 196	193	H-->F
2CXAA	192 - 196	193	H-->G
2CXAA	192 - 196	193	H-->I
2CXAA	192 - 196	193	H-->L
2CXAA	192 - 196	193	H-->P
2CXAA	192 - 196	193	H-->R
2CXAA	192 - 196	193	H-->W

2CXAA	192	196	193	H-->Y
2CXAA	192	196	194	T-->E
2CXAA	192	196	194	T-->F
2CXAA	192	196	194	T-->L
2CXAA	192	196	194	T-->R
2CXAA	192	196	194	T-->V
2CXAA	192	196	194	T-->Y
2CXAA	192	196	195	A-->D
2CXAA	192	196	195	A-->E
2CXAA	192	196	195	A-->I
2CXAA	192	196	196	S-->Y
2ASFA	12	17	12	D-->P
2ASFA	12	17	12	D-->V
1KQFC	41	45	41	Q-->R
1KQFC	41	45	42	W-->A
1KQFC	41	45	42	W-->G
1KQFC	41	45	42	W-->S
1KQFC	41	45	42	W-->V
1KQFC	41	45	43	L-->R
1KQFC	41	45	44	T-->R
1KQFC	41	45	45	Q-->D
1KQFC	92	96	92	I-->A
1KQFC	92	96	92	I-->E
1KQFC	92	96	92	I-->G
1KQFC	92	96	92	I-->K
1KQFC	92	96	92	I-->L
1KQFC	92	96	92	I-->N

1KQFC	92 - 96	92	I-->P
1KQFC	92 - 96	92	I-->Q
1KQFC	92 - 96	92	I-->R
1KQFC	92 - 96	92	I-->S
1KQFC	92 - 96	92	I-->T
1KQFC	92 - 96	92	I-->V
1KQFC	92 - 96	92	I-->Y
1KQFC	92 - 96	93	V-->D
1KQFC	92 - 96	93	V-->G
1KQFC	92 - 96	93	V-->H
1KQFC	92 - 96	93	V-->L
1KQFC	92 - 96	93	V-->P
1KQFC	92 - 96	93	V-->R
1KQFC	92 - 96	94	E-->A
1KQFC	92 - 96	94	E-->D
1KQFC	92 - 96	94	E-->F
1KQFC	92 - 96	94	E-->G
1KQFC	92 - 96	94	E-->I
1KQFC	92 - 96	94	E-->K
1KQFC	92 - 96	94	E-->S
1KQFC	92 - 96	94	E-->T
1KQFC	92 - 96	95	V-->D
1KQFC	92 - 96	95	V-->G
1KQFC	92 - 96	95	V-->I
1KQFC	92 - 96	95	V-->L
1KQFC	92 - 96	95	V-->N
1KQFC	92 - 96	95	V-->P

1KQFC	92 - 96	95	V-->T
1KQFC	92 - 96	96	L-->A
1KQFC	92 - 96	96	L-->D
1KQFC	92 - 96	96	L-->H
1KQFC	92 - 96	96	L-->K
1KQFC	92 - 96	96	L-->N
1KQFC	92 - 96	96	L-->P
1KQFC	92 - 96	96	L-->Q
1KQFC	92 - 96	96	L-->S
1KQFC	92 - 96	96	L-->T
1KQFC	92 - 96	96	L-->V
1KQFC	92 - 96	96	L-->W
1KQFC	92 - 96	96	L-->Y
1KQFC	179 - 184	179	S-->R
1KQFC	179 - 184	179	S-->T
1KQFC	179 - 184	182	G-->D
1KQFC	179 - 184	183	M-->T
2DQAA	43 - 48	44	P-->G
2DQAA	56 - 60	56	W-->A
2DQAA	56 - 60	56	W-->E
2DQAA	56 - 60	56	W-->N
2DQAA	56 - 60	56	W-->P
2DQAA	56 - 60	56	W-->R
2DQAA	56 - 60	56	W-->S
2DQAA	56 - 60	56	W-->V
2DQAA	56 - 60	56	W-->Y
2DQAA	56 - 60	57	K-->S

2DQAA	56 - 60	59	C-->A
2DQAA	56 - 60	59	C-->G
2DQAA	56 - 60	59	C-->R
2DQAA	56 - 60	59	C-->S
2R4IA	3 - 7	3	Q-->A
2R4IA	3 - 7	3	Q-->C
2R4IA	3 - 7	3	Q-->D
2R4IA	3 - 7	3	Q-->G
2R4IA	3 - 7	3	Q-->I
2R4IA	3 - 7	3	Q-->P
2R4IA	3 - 7	3	Q-->R
2R4IA	3 - 7	3	Q-->T
2R4IA	3 - 7	3	Q-->V
2R4IA	3 - 7	3	Q-->W
2R4IA	3 - 7	3	Q-->Y
2R4IA	3 - 7	4	R-->A
2R4IA	3 - 7	4	R-->F
2R4IA	3 - 7	4	R-->G
2R4IA	3 - 7	4	R-->L
2R4IA	3 - 7	4	R-->P
2R4IA	3 - 7	4	R-->S
2R4IA	3 - 7	4	R-->V
2R4IA	3 - 7	4	R-->Y
2R4IA	3 - 7	5	D-->A
2R4IA	3 - 7	5	D-->F
2R4IA	3 - 7	5	D-->G
2R4IA	3 - 7	5	D-->I

2R4IA	3 - 7	5	D-->L
2R4IA	3 - 7	5	D-->T
2R4IA	3 - 7	5	D-->V
2R4IA	3 - 7	6	V-->S
2R4IA	3 - 7	6	V-->Y
2R4IA	3 - 7	7	I-->A
2R4IA	3 - 7	7	I-->N
2R4IA	3 - 7	7	I-->T
2R4IA	3 - 7	7	I-->Y
1P5ZB	34 - 38	34	K-->A
1P5ZB	34 - 38	34	K-->D
1P5ZB	34 - 38	34	K-->E
1P5ZB	34 - 38	34	K-->N
1P5ZB	34 - 38	34	K-->P
1P5ZB	34 - 38	34	K-->R
1P5ZB	34 - 38	34	K-->S
1P5ZB	34 - 38	34	K-->T
1P5ZB	34 - 38	35	S-->A
1P5ZB	34 - 38	35	S-->F
1P5ZB	34 - 38	35	S-->G
1P5ZB	34 - 38	35	S-->I
1P5ZB	34 - 38	35	S-->L
1P5ZB	34 - 38	35	S-->N
1P5ZB	34 - 38	35	S-->Q
1P5ZB	34 - 38	35	S-->V
1P5ZB	34 - 38	36	T-->A
1P5ZB	34 - 38	36	T-->E

1P5ZB	34 - 38	36	T-->G
1P5ZB	34 - 38	36	T-->I
1P5ZB	34 - 38	36	T-->L
1P5ZB	34 - 38	36	T-->P
1P5ZB	34 - 38	36	T-->S
1P5ZB	34 - 38	36	T-->W
1P5ZB	34 - 38	36	T-->Y
1P5ZB	34 - 38	37	F-->G
1P5ZB	34 - 38	37	F-->H
1P5ZB	34 - 38	37	F-->L
1P5ZB	34 - 38	37	F-->P
1P5ZB	34 - 38	37	F-->R
1P5ZB	34 - 38	37	F-->T
1P5ZB	34 - 38	37	F-->V
1P5ZB	34 - 38	38	V-->D
1P5ZB	34 - 38	38	V-->F
1P5ZB	34 - 38	38	V-->G
1P5ZB	34 - 38	38	V-->P
1P5ZB	34 - 38	38	V-->R
1P5ZB	226 - 230	226	D-->R
1P5ZB	226 - 230	226	D-->V
1P5ZB	226 - 230	227	Y-->A
1P5ZB	226 - 230	227	Y-->E
1P5ZB	226 - 230	227	Y-->K
1P5ZB	226 - 230	227	Y-->L
1P5ZB	226 - 230	227	Y-->T
1P5ZB	226 - 230	227	Y-->V

1P5ZB	226 - 230	228	L-->I
1P5ZB	226 - 230	228	L-->S
1P5ZB	226 - 230	228	L-->V
1P5ZB	226 - 230	229	Q-->A
1P5ZB	226 - 230	229	Q-->D
1P5ZB	226 - 230	229	Q-->G
1P5ZB	226 - 230	229	Q-->I
1P5ZB	226 - 230	229	Q-->N
1P5ZB	226 - 230	229	Q-->T
1P5ZB	226 - 230	229	Q-->Y
1P5ZB	226 - 230	230	E-->G
1P5ZB	226 - 230	230	E-->L
1P5ZB	226 - 230	230	E-->P
1P5ZB	226 - 230	230	E-->R
1TQJC	188 - 193	188	W-->R
1TQJC	188 - 193	188	W-->T
1TQJC	188 - 193	189	Q-->L
1TQJC	188 - 193	191	L-->E
1TQJC	188 - 193	192	E-->D
1TQJC	188 - 193	192	E-->L
2A7MA	196 - 200	196	K-->E
2A7MA	196 - 200	196	K-->G
2A7MA	196 - 200	196	K-->P
2A7MA	196 - 200	196	K-->Q
2A7MA	196 - 200	196	K-->S
2A7MA	196 - 200	197	E-->I
2A7MA	196 - 200	197	E-->K

2A7MA	196 - 200	198	N-->I
2A7MA	196 - 200	198	N-->K
2A7MA	196 - 200	198	N-->L
2A7MA	196 - 200	198	N-->P
2A7MA	196 - 200	199	F-->A
2A7MA	196 - 200	199	F-->D
2A7MA	196 - 200	199	F-->G
2A7MA	196 - 200	199	F-->L
2A7MA	196 - 200	199	F-->Y
2A7MA	196 - 200	200	E-->D
2A7MA	196 - 200	200	E-->Q
2A7MA	196 - 200	200	E-->S
2A7MA	196 - 200	200	E-->T
2QLTA	103 - 107	103	I-->F
2QLTA	103 - 107	103	I-->G
2QLTA	103 - 107	103	I-->V
2QLTA	103 - 107	104	P-->E
2QLTA	103 - 107	104	P-->H
2QLTA	103 - 107	104	P-->K
2QLTA	103 - 107	104	P-->R
2QLTA	103 - 107	104	P-->S
2QLTA	103 - 107	104	P-->Y
2QLTA	103 - 107	105	E-->L
2QLTA	103 - 107	105	E-->M
2QLTA	103 - 107	106	K-->E
2QLTA	103 - 107	106	K-->F
2QLTA	103 - 107	106	K-->G

2QLTA	103	-	107	106	K-->L
2QLTA	103	-	107	106	K-->M
2QLTA	103	-	107	106	K-->P
2QLTA	103	-	107	106	K-->Q
2QLTA	103	-	107	106	K-->R
2QLTA	103	-	107	106	K-->S
2QLTA	103	-	107	107	Y-->A
2QLTA	103	-	107	107	Y-->D
2QLTA	103	-	107	107	Y-->E
2QLTA	103	-	107	107	Y-->G
2QLTA	103	-	107	107	Y-->H
2QLTA	103	-	107	107	Y-->I
2QLTA	103	-	107	107	Y-->M
2QLTA	103	-	107	107	Y-->N
2QLTA	103	-	107	107	Y-->Q
2QLTA	103	-	107	107	Y-->R
2QLTA	103	-	107	107	Y-->S
2QLTA	103	-	107	107	Y-->T
2QLTA	103	-	107	107	Y-->V
2C6UA	180	-	185	180	E-->Q
2RAFA	112	-	117	113	A-->C
2RAFA	112	-	117	113	A-->S
2RAFA	112	-	117	116	Q-->A
2RAFA	112	-	117	117	S-->V
2OKFA	101	-	105	101	L-->D
2OKFA	101	-	105	101	L-->G
2OKFA	101	-	105	101	L-->K

2OKFA	101 - 105	101	L-->S
2OKFA	101 - 105	101	L-->V
2OKFA	101 - 105	102	T-->E
2OKFA	101 - 105	102	T-->K
2OKFA	101 - 105	102	T-->N
2OKFA	101 - 105	103	T-->A
2OKFA	101 - 105	103	T-->D
2OKFA	101 - 105	103	T-->E
2OKFA	101 - 105	103	T-->F
2OKFA	101 - 105	103	T-->K
2OKFA	101 - 105	103	T-->N
2OKFA	101 - 105	103	T-->V
2OKFA	101 - 105	103	T-->Y
2OKFA	101 - 105	104	Y-->E
2OKFA	101 - 105	104	Y-->G
2OKFA	101 - 105	104	Y-->I
2OKFA	101 - 105	104	Y-->K
2OKFA	101 - 105	104	Y-->L
2OKFA	101 - 105	104	Y-->P
2OKFA	101 - 105	104	Y-->R
2OKFA	101 - 105	104	Y-->S
2OKFA	101 - 105	104	Y-->T
2OKFA	101 - 105	105	K-->D
2OKFA	101 - 105	105	K-->G
2OKFA	101 - 105	105	K-->I
2OKFA	101 - 105	105	K-->L
2OKFA	101 - 105	105	K-->P

2OKFA	101 - 105	105	K-->R
2OKFA	101 - 105	105	K-->S
2OKFA	101 - 105	105	K-->T
2OKFA	101 - 105	105	K-->V
2OKFA	101 - 105	105	K-->Y
2IA1B	56 - 61	56	D-->G
2IA1B	56 - 61	56	D-->V
2IA1B	56 - 61	57	I-->R
2IA1B	56 - 61	58	D-->Q
1W0HA	184 - 190	185	F-->V
1W0HA	194 - 198	194	Q-->D
1W0HA	194 - 198	194	Q-->E
1W0HA	194 - 198	194	Q-->G
1W0HA	194 - 198	194	Q-->L
1W0HA	194 - 198	194	Q-->P
1W0HA	194 - 198	195	D-->A
1W0HA	194 - 198	195	D-->L
1W0HA	194 - 198	195	D-->N
1W0HA	194 - 198	195	D-->S
1W0HA	194 - 198	195	D-->T
1W0HA	194 - 198	196	Q-->F
1W0HA	194 - 198	196	Q-->G
1W0HA	194 - 198	196	Q-->V
1W0HA	194 - 198	197	V-->A
1W0HA	194 - 198	197	V-->P
1W0HA	194 - 198	197	V-->R
1W0HA	194 - 198	198	D-->R

1W0HA	194	- 198	198	D-->T
1W0HA	194	- 198	198	D-->V
1XS0A	6	- 11	7	S-->P
1XS0A	6	- 11	10	A-->T
1XS0A	30	- 34	31	W-->E
1XS0A	30	- 34	31	W-->F
1XS0A	30	- 34	31	W-->K
1XS0A	30	- 34	31	W-->L
1XS0A	30	- 34	33	M-->F
1XS0A	30	- 34	33	M-->G
1XS0A	30	- 34	33	M-->L
1XS0A	30	- 34	33	M-->N
2A0BA	658	- 662	658	K-->D
2A0BA	658	- 662	658	K-->I
2A0BA	658	- 662	658	K-->L
2A0BA	658	- 662	658	K-->S
2A0BA	658	- 662	658	K-->V
2A0BA	658	- 662	659	S-->A
2A0BA	658	- 662	659	S-->E
2A0BA	658	- 662	659	S-->F
2A0BA	658	- 662	659	S-->G
2A0BA	658	- 662	659	S-->H
2A0BA	658	- 662	659	S-->L
2A0BA	658	- 662	659	S-->P
2A0BA	658	- 662	659	S-->R
2A0BA	658	- 662	659	S-->V
2A0BA	658	- 662	659	S-->W

2A0BA	658 - 662	660	E-->A
2A0BA	658 - 662	660	E-->D
2A0BA	658 - 662	660	E-->I
2A0BA	658 - 662	660	E-->Q
2A0BA	658 - 662	660	E-->S
2A0BA	658 - 662	660	E-->V
2A0BA	658 - 662	660	E-->W
2A0BA	658 - 662	661	A-->E
2A0BA	658 - 662	661	A-->G
2A0BA	658 - 662	661	A-->L
2A0BA	658 - 662	661	A-->P
2A0BA	658 - 662	661	A-->Q
2A0BA	658 - 662	661	A-->S
2A0BA	658 - 662	661	A-->V
2A0BA	658 - 662	662	L-->E
2A0BA	658 - 662	662	L-->I
2HVFA	23 - 28	26	A-->K
2HVFA	23 - 28	27	N-->G
2OOAA	933 - 939	935	K-->D
2OOAA	933 - 939	938	K-->L
2AFWA	175 - 179	175	D-->C
2AFWA	175 - 179	175	D-->F
2AFWA	175 - 179	175	D-->G
2AFWA	175 - 179	175	D-->I
2AFWA	175 - 179	175	D-->K
2AFWA	175 - 179	175	D-->M
2AFWA	175 - 179	175	D-->N

2AFWA	175 - 179	175	D-->P	
2AFWA	175 - 179	175	D-->Q	
2AFWA	175 - 179	175	D-->R	
2AFWA	175 - 179	175	D-->S	
2AFWA	175 - 179	176	K-->A	
2AFWA	175 - 179	176	K-->E	
2AFWA	175 - 179	176	K-->F	
2AFWA	175 - 179	176	K-->G	
2AFWA	175 - 179	176	K-->I	
2AFWA	175 - 179	176	K-->L	
2AFWA	175 - 179	176	K-->N	
2AFWA	175 - 179	176	K-->P	
2AFWA	175 - 179	176	K-->R	
2AFWA	175 - 179	176	K-->T	
2AFWA	175 - 179	176	K-->V	
2AFWA	175 - 179	177	K-->D	
2AFWA	175 - 179	177	K-->E	
2AFWA	175 - 179	177	K-->H	
2AFWA	175 - 179	177	K-->I	
2AFWA	175 - 179	177	K-->L	
2AFWA	175 - 179	177	K-->N	
2AFWA	175 - 179	177	K-->P	
2AFWA	175 - 179	177	K-->R	
2AFWA	175 - 179	177	K-->T	
2AFWA	175 - 179	177	K-->V	
2AFWA	175 - 179	178	L-->A	
2AFWA	175 - 179	178	L-->H	

2AFWA	175 - 179	178	L-->I
2AFWA	175 - 179	178	L-->P
2AFWA	175 - 179	178	L-->Q
2AFWA	175 - 179	178	L-->T
2AFWA	175 - 179	178	L-->V
2AFWA	175 - 179	179	L-->A
2AFWA	175 - 179	179	L-->E
2AFWA	175 - 179	179	L-->G
2AFWA	175 - 179	179	L-->H
2AFWA	175 - 179	179	L-->K
2AFWA	175 - 179	179	L-->N
2AFWA	175 - 179	179	L-->P
2AFWA	175 - 179	179	L-->S
2AFWA	175 - 179	179	L-->T
2AFWA	175 - 179	179	L-->V
1SMBA	144 - 148	144	F-->H
1SMBA	144 - 148	144	F-->K
1SMBA	144 - 148	144	F-->L
1SMBA	144 - 148	144	F-->P
1SMBA	144 - 148	144	F-->Q
1SMBA	144 - 148	144	F-->S
1SMBA	144 - 148	144	F-->T
1SMBA	144 - 148	144	F-->V
1SMBA	144 - 148	145	F-->A
1SMBA	144 - 148	145	F-->E
1SMBA	144 - 148	145	F-->P
1SMBA	144 - 148	145	F-->S

1SMBA	144	148	146	E-->A
1SMBA	144	148	146	E-->F
1SMBA	144	148	146	E-->K
1SMBA	144	148	146	E-->P
1SMBA	144	148	146	E-->R
1SMBA	144	148	146	E-->T
1SMBA	144	148	147	E-->I
1SMBA	144	148	147	E-->K
1SMBA	144	148	147	E-->M
1SMBA	144	148	147	E-->R
1SMBA	144	148	147	E-->Y
1SMBA	144	148	148	N-->T
2Q2IB	7	11	7	Y-->G
2Q2IB	7	11	7	Y-->R
2Q2IB	7	11	7	Y-->V
2Q2IB	7	11	8	A-->E
2Q2IB	7	11	8	A-->I
2Q2IB	7	11	8	A-->W
2Q2IB	7	11	9	D-->G
2Q2IB	7	11	9	D-->L
2Q2IB	7	11	9	D-->P
2Q2IB	7	11	9	D-->S
2Q2IB	7	11	10	F-->C
2Q2IB	7	11	10	F-->D
2Q2IB	7	11	10	F-->E
2Q2IB	7	11	10	F-->G
2Q2IB	7	11	10	F-->K

2Q2IB	7 - 11	10	F-->L
2Q2IB	7 - 11	10	F-->P
2Q2IB	7 - 11	10	F-->R
2Q2IB	7 - 11	10	F-->T
2Q2IB	7 - 11	11	E-->F
2Q2IB	7 - 11	11	E-->G
2Q2IB	7 - 11	11	E-->I
2Q2IB	7 - 11	11	E-->Q
2Q2IB	7 - 11	11	E-->S
2Q2IB	7 - 11	11	E-->V
2O8QA	32 - 36	32	V-->A
2O8QA	32 - 36	32	V-->N
2O8QA	32 - 36	32	V-->S
2O8QA	32 - 36	33	N-->A
2O8QA	32 - 36	33	N-->C
2O8QA	32 - 36	33	N-->D
2O8QA	32 - 36	33	N-->E
2O8QA	32 - 36	33	N-->F
2O8QA	32 - 36	33	N-->H
2O8QA	32 - 36	33	N-->I
2O8QA	32 - 36	33	N-->L
2O8QA	32 - 36	33	N-->P
2O8QA	32 - 36	33	N-->Q
2O8QA	32 - 36	33	N-->S
2O8QA	32 - 36	33	N-->T
2O8QA	32 - 36	33	N-->V
2O8QA	32 - 36	33	N-->W

2O8QA	32 - 36	34	E-->A
2O8QA	32 - 36	34	E-->C
2O8QA	32 - 36	34	E-->F
2O8QA	32 - 36	34	E-->G
2O8QA	32 - 36	34	E-->I
2O8QA	32 - 36	34	E-->K
2O8QA	32 - 36	34	E-->L
2O8QA	32 - 36	34	E-->N
2O8QA	32 - 36	34	E-->P
2O8QA	32 - 36	34	E-->T
2O8QA	32 - 36	35	A-->G
2O8QA	32 - 36	35	A-->I
2O8QA	32 - 36	35	A-->K
2O8QA	32 - 36	35	A-->N
2O8QA	32 - 36	36	T-->A
2O8QA	32 - 36	36	T-->E
2O8QA	32 - 36	36	T-->F
2O8QA	32 - 36	36	T-->I
2O8QA	32 - 36	36	T-->K
2O8QA	32 - 36	36	T-->L
2O8QA	32 - 36	36	T-->S
2O8QA	32 - 36	36	T-->V
3BA3A	53 - 57	53	A-->E
3BA3A	53 - 57	53	A-->G
3BA3A	53 - 57	53	A-->H
3BA3A	53 - 57	53	A-->L
3BA3A	53 - 57	53	A-->N

3BA3A	53 - 57	53	A-->R
3BA3A	53 - 57	54	L-->A
3BA3A	53 - 57	54	L-->E
3BA3A	53 - 57	54	L-->G
3BA3A	53 - 57	54	L-->H
3BA3A	53 - 57	54	L-->K
3BA3A	53 - 57	54	L-->M
3BA3A	53 - 57	54	L-->S
3BA3A	53 - 57	55	K-->G
3BA3A	53 - 57	55	K-->I
3BA3A	53 - 57	55	K-->L
3BA3A	53 - 57	55	K-->M
3BA3A	53 - 57	55	K-->P
3BA3A	53 - 57	55	K-->T
3BA3A	53 - 57	55	K-->V
3BA3A	53 - 57	56	V-->E
3BA3A	53 - 57	56	V-->F
3BA3A	53 - 57	56	V-->I
3BA3A	53 - 57	56	V-->L
3BA3A	53 - 57	56	V-->S
3BA3A	53 - 57	56	V-->Y
3BA3A	53 - 57	57	Y-->A
3BA3A	53 - 57	57	Y-->C
3BA3A	53 - 57	57	Y-->D
3BA3A	53 - 57	57	Y-->E
3BA3A	53 - 57	57	Y-->G
3BA3A	53 - 57	57	Y-->I

3BA3A	53	57	57	Y-->L
3BA3A	53	57	57	Y-->S
3BA3A	53	57	57	Y-->T
3BA3A	53	57	57	Y-->V
2BEMA	50	54	50	G-->L
2BEMA	50	54	50	G-->W
2BEMA	50	54	51	S-->A
2BEMA	50	54	51	S-->D
2BEMA	50	54	51	S-->F
2BEMA	50	54	51	S-->I
2BEMA	50	54	51	S-->P
2BEMA	50	54	52	V-->A
2BEMA	50	54	52	V-->I
2BEMA	50	54	52	V-->K
2BEMA	50	54	52	V-->N
2BEMA	50	54	52	V-->T
2BEMA	50	54	52	V-->W
2BEMA	50	54	52	V-->Y
2BEMA	50	54	53	Q-->E
2BEMA	50	54	53	Q-->F
2BEMA	50	54	53	Q-->G
2BEMA	50	54	53	Q-->K
2BEMA	50	54	53	Q-->N
2BEMA	50	54	53	Q-->P
2BEMA	50	54	53	Q-->T
2BEMA	50	54	54	Y-->A
2BEMA	50	54	54	Y-->D

2BEMA	50 - 54	54	Y-->G
2BEMA	50 - 54	54	Y-->I
2BEMA	50 - 54	54	Y-->L
2BEMA	50 - 54	54	Y-->R
2BEMA	50 - 54	54	Y-->S
2BEMA	50 - 54	54	Y-->T
2BEMA	50 - 54	54	Y-->V
1VHWA	116 - 120	116	V-->R
1VHWA	116 - 120	116	V-->S
1VHWA	116 - 120	116	V-->T
1VHWA	116 - 120	117	N-->A
1VHWA	116 - 120	117	N-->D
1VHWA	116 - 120	117	N-->F
1VHWA	116 - 120	117	N-->G
1VHWA	116 - 120	117	N-->I
1VHWA	116 - 120	117	N-->K
1VHWA	116 - 120	117	N-->L
1VHWA	116 - 120	117	N-->R
1VHWA	116 - 120	117	N-->V
1VHWA	116 - 120	117	N-->Y
1VHWA	116 - 120	118	R-->A
1VHWA	116 - 120	118	R-->G
1VHWA	116 - 120	118	R-->K
1VHWA	116 - 120	118	R-->L
1VHWA	116 - 120	118	R-->N
1VHWA	116 - 120	118	R-->S
1VHWA	116 - 120	118	R-->T

1VHWA	116 - 120	118	R-->Y
1VHWA	116 - 120	119	I-->H
1VHWA	116 - 120	119	I-->R
1VHWA	116 - 120	120	R-->A
1VHWA	116 - 120	120	R-->D
1VHWA	116 - 120	120	R-->E
1VHWA	116 - 120	120	R-->G
1VHWA	116 - 120	120	R-->H
1VHWA	116 - 120	120	R-->I
1VHWA	116 - 120	120	R-->M
1VHWA	116 - 120	120	R-->N
1VHWA	116 - 120	120	R-->P
1VHWA	116 - 120	120	R-->T
1VHWA	116 - 120	120	R-->V
2RBDB	127 - 131	127	E-->A
2RBDB	127 - 131	127	E-->D
2RBDB	127 - 131	127	E-->F
2RBDB	127 - 131	127	E-->G
2RBDB	127 - 131	127	E-->H
2RBDB	127 - 131	127	E-->I
2RBDB	127 - 131	127	E-->L
2RBDB	127 - 131	127	E-->N
2RBDB	127 - 131	127	E-->P
2RBDB	127 - 131	127	E-->Q
2RBDB	127 - 131	127	E-->R
2RBDB	127 - 131	127	E-->S
2RBDB	127 - 131	127	E-->T

2RBDB	127 - 131	127	E-->V
2RBDB	127 - 131	127	E-->Y
2RBDB	127 - 131	128	D-->A
2RBDB	127 - 131	128	D-->C
2RBDB	127 - 131	128	D-->E
2RBDB	127 - 131	128	D-->I
2RBDB	127 - 131	128	D-->L
2RBDB	127 - 131	128	D-->N
2RBDB	127 - 131	128	D-->P
2RBDB	127 - 131	128	D-->Q
2RBDB	127 - 131	128	D-->R
2RBDB	127 - 131	128	D-->T
2RBDB	127 - 131	128	D-->V
2RBDB	127 - 131	128	D-->W
2RBDB	127 - 131	129	V-->D
2RBDB	127 - 131	129	V-->E
2RBDB	127 - 131	129	V-->F
2RBDB	127 - 131	129	V-->G
2RBDB	127 - 131	129	V-->I
2RBDB	127 - 131	129	V-->L
2RBDB	127 - 131	129	V-->N
2RBDB	127 - 131	129	V-->S
2RBDB	127 - 131	129	V-->T
2RBDB	127 - 131	129	V-->Y
2RBDB	127 - 131	130	A-->E
2RBDB	127 - 131	130	A-->F
2RBDB	127 - 131	130	A-->H

2RBDB	127 - 131	130	A-->K
2RBDB	127 - 131	130	A-->L
2RBDB	127 - 131	130	A-->N
2RBDB	127 - 131	130	A-->P
2RBDB	127 - 131	130	A-->R
2RBDB	127 - 131	130	A-->S
2RBDB	127 - 131	130	A-->T
2RBDB	127 - 131	130	A-->V
2RBDB	127 - 131	131	L-->E
2RBDB	127 - 131	131	L-->F
2RBDB	127 - 131	131	L-->I
2RBDB	127 - 131	131	L-->K
2RBDB	127 - 131	131	L-->Q
2RBDB	127 - 131	131	L-->T
2RBDB	127 - 131	131	L-->V
2RBDB	133 - 137	133	F-->A
2RBDB	133 - 137	133	F-->R
2RBDB	133 - 137	133	F-->V
2RBDB	133 - 137	134	S-->K
2RBDB	133 - 137	134	S-->N
2RBDB	133 - 137	135	Q-->C
2RBDB	133 - 137	135	Q-->D
2RBDB	133 - 137	135	Q-->E
2RBDB	133 - 137	136	F-->D
2RBDB	133 - 137	136	F-->L
2RBDB	133 - 137	137	H-->K
2RBDB	133 - 137	137	H-->P

2RBDB	133 - 137	137	H-->S	
2RBDB	133 - 137	137	H-->V	
2RBDB	148 - 153	149	K-->Q	
2RBDB	148 - 153	151	N-->P	
2RBDB	148 - 153	152	K-->Q	
2RBDB	148 - 153	153	N-->D	
2RBDB	148 - 153	153	N-->H	
2RBDB	148 - 153	153	N-->K	
2A2KA	429 - 433	429	P-->S	
2A2KA	429 - 433	429	P-->V	
2A2KA	429 - 433	430	Y-->A	
2A2KA	429 - 433	430	Y-->E	
2A2KA	429 - 433	430	Y-->G	
2A2KA	429 - 433	430	Y-->H	
2A2KA	429 - 433	430	Y-->K	
2A2KA	429 - 433	430	Y-->R	
2A2KA	429 - 433	430	Y-->V	
2A2KA	429 - 433	431	E-->H	
2A2KA	429 - 433	431	E-->N	
2A2KA	429 - 433	431	E-->P	
2A2KA	429 - 433	432	Y-->F	
2A2KA	429 - 433	432	Y-->G	
2A2KA	429 - 433	432	Y-->I	
2A2KA	429 - 433	432	Y-->K	
2A2KA	429 - 433	432	Y-->L	
2A2KA	429 - 433	432	Y-->Q	
2A2KA	429 - 433	432	Y-->S	

2A2KA	429 - 433	432	Y-->T
2A2KA	429 - 433	432	Y-->V
2A2KA	429 - 433	433	E-->L
2A2KA	511 - 515	511	G-->A
2A2KA	511 - 515	511	G-->I
2A2KA	511 - 515	511	G-->S
2A2KA	511 - 515	511	G-->V
2A2KA	511 - 515	512	Y-->E
2A2KA	511 - 515	512	Y-->K
2A2KA	511 - 515	512	Y-->L
2A2KA	511 - 515	512	Y-->N
2A2KA	511 - 515	512	Y-->P
2A2KA	511 - 515	512	Y-->Q
2A2KA	511 - 515	512	Y-->S
2A2KA	511 - 515	512	Y-->T
2A2KA	511 - 515	513	K-->D
2A2KA	511 - 515	513	K-->E
2A2KA	511 - 515	513	K-->F
2A2KA	511 - 515	513	K-->L
2A2KA	511 - 515	514	E-->D
2A2KA	511 - 515	514	E-->L
2A2KA	511 - 515	514	E-->P
2A2KA	511 - 515	514	E-->T
2A2KA	511 - 515	514	E-->V
2A2KA	511 - 515	515	F-->D
2A2KA	511 - 515	515	F-->E
2A2KA	511 - 515	515	F-->G

2A2KA	511	- 515	515	F-->I
2A2KA	511	- 515	515	F-->N
2A2KA	511	- 515	515	F-->P
2A2KA	511	- 515	515	F-->R
2A2KA	511	- 515	515	F-->S
2A2KA	511	- 515	515	F-->W
2O1RA	16	- 21	16	L-->G
2O1RA	16	- 21	16	L-->R
2O1RA	16	- 21	17	R-->N
2O1RA	16	- 21	18	D-->L
2O1RA	16	- 21	19	L-->Q
2O1RA	16	- 21	21	K-->A
2O1RA	16	- 21	21	K-->Q
1HQ1A	37	- 41	37	M-->A
1HQ1A	37	- 41	37	M-->E
1HQ1A	37	- 41	37	M-->G
1HQ1A	37	- 41	37	M-->H
1HQ1A	37	- 41	37	M-->K
1HQ1A	37	- 41	37	M-->N
1HQ1A	37	- 41	37	M-->T
1HQ1A	37	- 41	39	E-->A
1HQ1A	37	- 41	39	E-->P
1HQ1A	37	- 41	39	E-->R
1HQ1A	37	- 41	40	R-->K
1WRMA	23	- 28	28	K-->G
2GRCA	1481	- 1485	1481	L-->A
2GRCA	1481	- 1485	1481	L-->D

2GRCA	1481 - 1485	1481	L-->G
2GRCA	1481 - 1485	1481	L-->H
2GRCA	1481 - 1485	1481	L-->K
2GRCA	1481 - 1485	1481	L-->P
2GRCA	1481 - 1485	1481	L-->R
2GRCA	1481 - 1485	1481	L-->T
2GRCA	1481 - 1485	1481	L-->Y
2GRCA	1481 - 1485	1482	S-->D
2GRCA	1481 - 1485	1482	S-->F
2GRCA	1481 - 1485	1482	S-->G
2GRCA	1481 - 1485	1482	S-->L
2GRCA	1481 - 1485	1482	S-->Q
2GRCA	1481 - 1485	1482	S-->T
2GRCA	1481 - 1485	1483	E-->D
2GRCA	1481 - 1485	1483	E-->G
2GRCA	1481 - 1485	1483	E-->K
2GRCA	1481 - 1485	1483	E-->R
2GRCA	1481 - 1485	1484	V-->A
2GRCA	1481 - 1485	1484	V-->E
2GRCA	1481 - 1485	1484	V-->G
2GRCA	1481 - 1485	1484	V-->K
2GRCA	1481 - 1485	1484	V-->M
2GRCA	1481 - 1485	1484	V-->Q
2GRCA	1481 - 1485	1484	V-->R
2GRCA	1481 - 1485	1485	F-->A
2GRCA	1481 - 1485	1485	F-->D
2GRCA	1481 - 1485	1485	F-->E

2GRCA	1481 - 1485	1485	F-->G
2GRCA	1481 - 1485	1485	F-->H
2GRCA	1481 - 1485	1485	F-->L
2GRCA	1481 - 1485	1485	F-->P
2GRCA	1481 - 1485	1485	F-->V
2GRCA	1481 - 1485	1485	F-->Y
2GRCA	1495 - 1500	1497	Y-->G
2GRCA	1495 - 1500	1497	Y-->M
2GRCA	1495 - 1500	1498	Y-->D
2GRCA	1495 - 1500	1500	L-->P
1Y5HB	51 - 56	52	R-->A
1Y5HB	51 - 56	52	R-->S
1Y5HB	51 - 56	52	R-->Y
1Y5HB	51 - 56	54	I-->V
1Y5HB	70 - 74	70	A-->D
1Y5HB	70 - 74	70	A-->E
1Y5HB	70 - 74	70	A-->G
1Y5HB	70 - 74	70	A-->I
1Y5HB	70 - 74	70	A-->K
1Y5HB	70 - 74	70	A-->L
1Y5HB	70 - 74	70	A-->M
1Y5HB	70 - 74	70	A-->N
1Y5HB	70 - 74	70	A-->P
1Y5HB	70 - 74	70	A-->R
1Y5HB	70 - 74	70	A-->S
1Y5HB	70 - 74	70	A-->T
1Y5HB	70 - 74	70	A-->V

1Y5HB	70 - 74	70	A-->Y
1Y5HB	70 - 74	71	G-->D
1Y5HB	70 - 74	71	G-->E
1Y5HB	70 - 74	71	G-->I
1Y5HB	70 - 74	71	G-->K
1Y5HB	70 - 74	71	G-->L
1Y5HB	70 - 74	71	G-->R
1Y5HB	70 - 74	71	G-->S
1Y5HB	70 - 74	71	G-->V
1Y5HB	70 - 74	72	E-->A
1Y5HB	70 - 74	72	E-->D
1Y5HB	70 - 74	72	E-->F
1Y5HB	70 - 74	72	E-->G
1Y5HB	70 - 74	72	E-->H
1Y5HB	70 - 74	72	E-->I
1Y5HB	70 - 74	72	E-->K
1Y5HB	70 - 74	72	E-->L
1Y5HB	70 - 74	72	E-->M
1Y5HB	70 - 74	72	E-->N
1Y5HB	70 - 74	72	E-->P
1Y5HB	70 - 74	72	E-->Q
1Y5HB	70 - 74	72	E-->R
1Y5HB	70 - 74	72	E-->S
1Y5HB	70 - 74	72	E-->T
1Y5HB	70 - 74	72	E-->V
1Y5HB	70 - 74	72	E-->Y
1Y5HB	70 - 74	73	L-->A

1Y5HB	70 - 74	73	L-->C
1Y5HB	70 - 74	73	L-->D
1Y5HB	70 - 74	73	L-->E
1Y5HB	70 - 74	73	L-->F
1Y5HB	70 - 74	73	L-->G
1Y5HB	70 - 74	73	L-->H
1Y5HB	70 - 74	73	L-->K
1Y5HB	70 - 74	73	L-->N
1Y5HB	70 - 74	73	L-->Q
1Y5HB	70 - 74	73	L-->R
1Y5HB	70 - 74	73	L-->S
1Y5HB	70 - 74	73	L-->V
1Y5HB	70 - 74	73	L-->Y
1Y5HB	70 - 74	74	A-->C
1Y5HB	70 - 74	74	A-->D
1Y5HB	70 - 74	74	A-->E
1Y5HB	70 - 74	74	A-->G
1Y5HB	70 - 74	74	A-->K
1Y5HB	70 - 74	74	A-->L
1Y5HB	70 - 74	74	A-->N
1Y5HB	70 - 74	74	A-->P
1Y5HB	70 - 74	74	A-->Q
1Y5HB	70 - 74	74	A-->R
1Y5HB	70 - 74	74	A-->S
1Y5HB	70 - 74	74	A-->V
1Y5HB	70 - 74	74	A-->Y
1Y5HB	116 - 121	117	A-->D

1Y5HB	116	121	121	R-->N
1QW2A	63	67	63	E-->D
1QW2A	63	67	63	E-->G
1QW2A	63	67	63	E-->I
1QW2A	63	67	63	E-->V
1QW2A	63	67	64	T-->E
1QW2A	63	67	64	T-->G
1QW2A	63	67	64	T-->I
1QW2A	63	67	65	S-->G
1QW2A	63	67	65	S-->K
1QW2A	63	67	65	S-->Q
1QW2A	63	67	65	S-->V
1QW2A	63	67	66	N-->A
1QW2A	63	67	66	N-->C
1QW2A	63	67	66	N-->D
1QW2A	63	67	66	N-->I
1QW2A	63	67	66	N-->P
1QW2A	63	67	66	N-->S
1QW2A	63	67	66	N-->V
1QW2A	63	67	67	K-->F
1QW2A	63	67	67	K-->N
1QW2A	63	67	67	K-->S
1QW2A	63	67	67	K-->W
1QW2A	63	67	67	K-->Y
1QW2A	94	98	94	Q-->F
1QW2A	94	98	94	Q-->T
1QW2A	94	98	94	Q-->V

1QW2A	94 - 98	95	E-->A	
1QW2A	94 - 98	95	E-->D	
1QW2A	94 - 98	95	E-->K	
1QW2A	94 - 98	95	E-->P	
1QW2A	94 - 98	95	E-->S	
1QW2A	94 - 98	95	E-->T	
1QW2A	94 - 98	96	A-->L	
1QW2A	94 - 98	96	A-->S	
1QW2A	94 - 98	96	A-->Y	
1QW2A	94 - 98	97	Q-->E	
1QW2A	94 - 98	97	Q-->P	
1QW2A	94 - 98	98	K-->E	
1QW2A	94 - 98	98	K-->Q	
1QW2A	94 - 98	98	K-->R	
1QW2A	94 - 98	98	K-->T	
1H32A	40 - 45	41	D-->R	
1H32A	40 - 45	42	T-->D	
1H32A	40 - 45	43	Q-->L	
1H32A	40 - 45	44	A-->C	
1H32A	76 - 80	77	A-->L	
1H32A	76 - 80	78	D-->A	
1H32A	76 - 80	78	D-->G	
1H32A	76 - 80	78	D-->Q	
1H32A	76 - 80	78	D-->S	
1H32A	76 - 80	78	D-->T	
1H32A	76 - 80	80	H-->E	
1H32A	76 - 80	80	H-->S	

1H32A	177	- 181	177	C-->A
1H32A	177	- 181	177	C-->Y
1H32A	177	- 181	178	A-->D
1H32A	177	- 181	178	A-->E
1H32A	177	- 181	178	A-->F
1H32A	177	- 181	178	A-->Q
1H32A	177	- 181	178	A-->S
1H32A	177	- 181	178	A-->T
1H32A	177	- 181	179	S-->A
1H32A	177	- 181	179	S-->G
1H32A	177	- 181	179	S-->Q
1H32A	177	- 181	179	S-->T
1H32A	177	- 181	181	H-->E
1H32A	177	- 181	181	H-->I
1Z3EA	62	- 67	63	V-->I
1Z3EA	109	- 113	109	E-->D
1Z3EA	109	- 113	109	E-->G
1Z3EA	109	- 113	109	E-->S
1Z3EA	109	- 113	110	I-->D
1Z3EA	109	- 113	110	I-->F
1Z3EA	109	- 113	110	I-->H
1Z3EA	109	- 113	110	I-->K
1Z3EA	109	- 113	110	I-->N
1Z3EA	109	- 113	110	I-->V
1Z3EA	109	- 113	110	I-->Y
1Z3EA	109	- 113	111	R-->A
1Z3EA	109	- 113	111	R-->E

1Z3EA	109 - 113	111	R-->G
1Z3EA	109 - 113	111	R-->I
1Z3EA	109 - 113	111	R-->K
1Z3EA	109 - 113	111	R-->L
1Z3EA	109 - 113	111	R-->S
1Z3EA	109 - 113	111	R-->V
1Z3EA	109 - 113	112	R-->G
1Z3EA	109 - 113	112	R-->P
1Z3EA	109 - 113	112	R-->Q
1Z3EA	109 - 113	112	R-->T
1Z3EA	109 - 113	112	R-->V
1Z3EA	109 - 113	113	F-->A
1Z3EA	109 - 113	113	F-->C
1Z3EA	109 - 113	113	F-->G
1Z3EA	109 - 113	113	F-->I
1Z3EA	109 - 113	113	F-->L
1Z3EA	109 - 113	113	F-->M
1Z3EA	109 - 113	113	F-->W
1Z3EA	109 - 113	113	F-->Y
1Z3EB	274 - 278	274	V-->G
1Z3EB	274 - 278	274	V-->R
1Z3EB	274 - 278	275	Q-->A
1Z3EB	274 - 278	275	Q-->C
1Z3EB	274 - 278	275	Q-->E
1Z3EB	274 - 278	275	Q-->F
1Z3EB	274 - 278	275	Q-->G
1Z3EB	274 - 278	275	Q-->K

1Z3EB	274 - 278	275	Q-->N
1Z3EB	274 - 278	275	Q-->P
1Z3EB	274 - 278	275	Q-->R
1Z3EB	274 - 278	275	Q-->T
1Z3EB	274 - 278	276	E-->C
1Z3EB	274 - 278	276	E-->G
1Z3EB	274 - 278	276	E-->L
1Z3EB	274 - 278	276	E-->R
1Z3EB	274 - 278	276	E-->V
1Z3EB	274 - 278	276	E-->W
1Z3EB	274 - 278	277	L-->D
1Z3EB	274 - 278	277	L-->E
1Z3EB	274 - 278	277	L-->G
1Z3EB	274 - 278	277	L-->H
1Z3EB	274 - 278	277	L-->N
1Z3EB	274 - 278	277	L-->S
1Z3EB	274 - 278	277	L-->V
1Z3EB	274 - 278	278	A-->D
1Z3EB	274 - 278	278	A-->E
1Z3EB	274 - 278	278	A-->I
1Z3EB	274 - 278	278	A-->L
1Z3EB	274 - 278	278	A-->N
1Z3EB	274 - 278	278	A-->S
1Z3EB	282 - 286	282	E-->G
1Z3EB	282 - 286	283	E-->D
1Z3EB	282 - 286	283	E-->P
1Z3EB	282 - 286	285	M-->P

1Z3EB	282	286	285	M-->S
1Z3EB	282	286	285	M-->V
1Z3EB	282	286	286	M-->C
1Z3EB	282	286	286	M-->D
1Z3EB	282	286	286	M-->G
1Z3EB	282	286	286	M-->I
1Z3EB	282	286	286	M-->K
1SJ1A	16	20	16	I-->A
1SJ1A	16	20	16	I-->H
1SJ1A	16	20	16	I-->L
1SJ1A	16	20	16	I-->P
1SJ1A	16	20	16	I-->Y
1SJ1A	16	20	17	C-->A
1SJ1A	16	20	17	C-->D
1SJ1A	16	20	17	C-->E
1SJ1A	16	20	17	C-->G
1SJ1A	16	20	17	C-->K
1SJ1A	16	20	17	C-->N
1SJ1A	16	20	17	C-->Q
1SJ1A	16	20	17	C-->S
1SJ1A	16	20	17	C-->W
1SJ1A	16	20	18	A-->F
1SJ1A	16	20	18	A-->G
1SJ1A	16	20	18	A-->I
1SJ1A	16	20	18	A-->S
1SJ1A	16	20	18	A-->W
1SJ1A	16	20	19	S-->G

1SJ1A	16 - 20	19	S-->I
1SJ1A	16 - 20	19	S-->L
1SJ1A	16 - 20	19	S-->P
1SJ1A	16 - 20	19	S-->R
1SJ1A	16 - 20	20	L-->M
2O6PA	48 - 52	48	I-->C
2O6PA	48 - 52	48	I-->D
2O6PA	48 - 52	48	I-->E
2O6PA	48 - 52	48	I-->G
2O6PA	48 - 52	48	I-->T
2O6PA	48 - 52	49	A-->P
2O6PA	48 - 52	49	A-->R
2O6PA	48 - 52	50	N-->H
2O6PA	48 - 52	50	N-->I
2O6PA	48 - 52	50	N-->K
2O6PA	48 - 52	50	N-->M
2O6PA	48 - 52	51	D-->A
2O6PA	48 - 52	51	D-->G
2O6PA	48 - 52	51	D-->N
2O6PA	48 - 52	51	D-->Y
2O6PA	48 - 52	52	Y-->I
2O6PA	48 - 52	52	Y-->P
2O6PA	48 - 52	52	Y-->Q
2O6PA	48 - 52	52	Y-->T
1DFMB	74 - 79	74	K-->L
1DFMB	74 - 79	75	R-->Y
2H6FB	528 - 533	530	H-->L

2H6FB	528 - 533	531	A-->R
2IW1A	223 - 227	223	E-->D
2IW1A	223 - 227	223	E-->G
2IW1A	223 - 227	223	E-->H
2IW1A	223 - 227	224	S-->E
2IW1A	223 - 227	224	S-->P
2IW1A	223 - 227	224	S-->V
2IW1A	223 - 227	225	L-->A
2IW1A	223 - 227	226	R-->G
2IW1A	223 - 227	226	R-->K
2IW1A	223 - 227	226	R-->P
2IW1A	223 - 227	227	H-->A
2IW1A	223 - 227	227	H-->D
2IW1A	223 - 227	227	H-->E
2IW1A	223 - 227	227	H-->F
2IW1A	223 - 227	227	H-->G
2IW1A	223 - 227	227	H-->I
2IW1A	223 - 227	227	H-->L
2IW1A	223 - 227	227	H-->P
2DGED	105 - 110	105	T-->S
2DGED	105 - 110	106	K-->H
2DGED	105 - 110	107	D-->I
2DGED	105 - 110	108	L-->D
1QCZA	17 - 22	19	D-->E
1TP6A	28 - 33	29	L-->F
1TP6A	28 - 33	29	L-->T
1TP6A	28 - 33	33	M-->Q

1F7LA	25 - 29	25	F-->A
1F7LA	25 - 29	25	F-->E
1F7LA	25 - 29	25	F-->G
1F7LA	25 - 29	25	F-->K
1F7LA	25 - 29	25	F-->L
1F7LA	25 - 29	25	F-->R
1F7LA	25 - 29	25	F-->S
1F7LA	25 - 29	26	A-->D
1F7LA	25 - 29	26	A-->G
1F7LA	25 - 29	26	A-->K
1F7LA	25 - 29	26	A-->T
1F7LA	25 - 29	27	E-->G
1F7LA	25 - 29	27	E-->M
1F7LA	25 - 29	28	R-->D
1F7LA	25 - 29	28	R-->E
1F7LA	25 - 29	28	R-->K
1F7LA	25 - 29	28	R-->V
1F7LA	25 - 29	29	I-->E
1F7LA	25 - 29	29	I-->L
1F7LA	25 - 29	29	I-->P
1F7LA	25 - 29	29	I-->S
1OPDA	47 - 52	48	F-->V
1OPDA	47 - 52	50	L-->T
1Z3XA	20 - 26	26	A-->P
1Z3XA	127 - 132	129	A-->L
1Z3XA	127 - 132	132	R-->C
1Z3XA	127 - 132	132	R-->D

1Z3XA	127 - 132	132	R-->T	
1Z3XA	137 - 141	137	F-->G	
1Z3XA	137 - 141	137	F-->I	
1Z3XA	137 - 141	137	F-->L	
1Z3XA	137 - 141	137	F-->N	
1Z3XA	137 - 141	137	F-->T	
1Z3XA	137 - 141	137	F-->V	
1Z3XA	137 - 141	138	T-->A	
1Z3XA	137 - 141	138	T-->D	
1Z3XA	137 - 141	138	T-->E	
1Z3XA	137 - 141	138	T-->F	
1Z3XA	137 - 141	138	T-->G	
1Z3XA	137 - 141	138	T-->I	
1Z3XA	137 - 141	138	T-->K	
1Z3XA	137 - 141	138	T-->L	
1Z3XA	137 - 141	138	T-->M	
1Z3XA	137 - 141	138	T-->P	
1Z3XA	137 - 141	138	T-->R	
1Z3XA	137 - 141	138	T-->S	
1Z3XA	137 - 141	138	T-->V	
1Z3XA	137 - 141	138	T-->Y	
1Z3XA	137 - 141	139	E-->C	
1Z3XA	137 - 141	139	E-->F	
1Z3XA	137 - 141	139	E-->K	
1Z3XA	137 - 141	139	E-->W	
1Z3XA	137 - 141	139	E-->Y	
1Z3XA	137 - 141	140	V-->F	

1Z3XA	137 - 141	140	V-->G	
1Z3XA	137 - 141	140	V-->N	
1Z3XA	137 - 141	141	E-->A	
1Z3XA	137 - 141	141	E-->H	
1Z3XA	137 - 141	141	E-->N	
1Z3XA	137 - 141	141	E-->P	
1Z3XA	137 - 141	141	E-->S	
1Z3XA	137 - 141	141	E-->T	
1Z3XA	137 - 141	141	E-->V	
2P14A	2 - 7	2	Q-->T	
2P14A	2 - 7	5	L-->I	
2P14A	2 - 7	5	L-->T	
2FOMA	63 - 68	64	Q-->D	
2FOMA	63 - 68	64	Q-->V	
2FOMA	63 - 68	65	A-->C	
2FOMA	63 - 68	66	E-->V	
2FOMA	63 - 68	67	I-->F	
2FOMA	63 - 68	68	S-->N	
1VYIA	247 - 252	252	H-->G	
1VYIA	247 - 252	252	H-->P	
1VYIA	272 - 277	274	F-->G	
1VYIA	272 - 277	275	Q-->S	
1ISUA	4 - 9	6	M-->P	
1ISUA	4 - 9	7	R-->V	
1ISUA	4 - 9	8	K-->P	
1UV4A	60 - 64	60	S-->N	
1UV4A	60 - 64	61	W-->I	

1UV4A	60 - 64	61	W-->P
1UV4A	60 - 64	62	W-->T
1UV4A	60 - 64	63	S-->G
1UV4A	60 - 64	63	S-->V
1UV4A	60 - 64	64	N-->D
1UV4A	60 - 64	64	N-->H
2IMJA	60 - 64	60	P-->A
2IMJA	60 - 64	60	P-->D
2IMJA	60 - 64	60	P-->E
2IMJA	60 - 64	60	P-->G
2IMJA	60 - 64	60	P-->K
2IMJA	60 - 64	60	P-->L
2IMJA	60 - 64	60	P-->M
2IMJA	60 - 64	60	P-->Q
2IMJA	60 - 64	60	P-->R
2IMJA	60 - 64	60	P-->S
2IMJA	60 - 64	60	P-->V
2IMJA	60 - 64	61	E-->A
2IMJA	60 - 64	61	E-->D
2IMJA	60 - 64	61	E-->F
2IMJA	60 - 64	61	E-->G
2IMJA	60 - 64	61	E-->K
2IMJA	60 - 64	61	E-->L
2IMJA	60 - 64	61	E-->R
2IMJA	60 - 64	61	E-->V
2IMJA	60 - 64	62	R-->D
2IMJA	60 - 64	62	R-->E

2IMJA	60 - 64	62	R-->F
2IMJA	60 - 64	62	R-->G
2IMJA	60 - 64	62	R-->K
2IMJA	60 - 64	62	R-->L
2IMJA	60 - 64	62	R-->N
2IMJA	60 - 64	62	R-->P
2IMJA	60 - 64	62	R-->S
2IMJA	60 - 64	62	R-->T
2IMJA	60 - 64	62	R-->V
2IMJA	60 - 64	62	R-->W
2IMJA	60 - 64	62	R-->Y
2IMJA	60 - 64	63	V-->F
2IMJA	60 - 64	63	V-->I
2IMJA	60 - 64	63	V-->K
2IMJA	60 - 64	63	V-->L
2IMJA	60 - 64	64	S-->A
2IMJA	60 - 64	64	S-->E
2IMJA	60 - 64	64	S-->F
2IMJA	60 - 64	64	S-->G
2IMJA	60 - 64	64	S-->I
2IMJA	60 - 64	64	S-->K
2IMJA	60 - 64	64	S-->M
2IMJA	60 - 64	64	S-->N
2IMJA	60 - 64	64	S-->R
2IMJA	60 - 64	64	S-->T
2IMJA	60 - 64	64	S-->V
2IMJA	60 - 64	64	S-->Y

2BKMA	63 - 68	64	Y-->F	
2BKMA	63 - 68	66	A-->V	
2BKMA	63 - 68	67	E-->L	
2BKMA	73 - 77	73	L-->G	
2BKMA	73 - 77	73	L-->T	
2BKMA	73 - 77	73	L-->V	
2BKMA	73 - 77	74	R-->G	
2BKMA	73 - 77	74	R-->K	
2BKMA	73 - 77	74	R-->L	
2BKMA	73 - 77	74	R-->Q	
2BKMA	73 - 77	74	R-->S	
2BKMA	73 - 77	74	R-->T	
2BKMA	73 - 77	75	A-->F	
2BKMA	73 - 77	75	A-->H	
2BKMA	73 - 77	75	A-->I	
2BKMA	73 - 77	75	A-->L	
2BKMA	73 - 77	75	A-->P	
2BKMA	73 - 77	75	A-->V	
2BKMA	73 - 77	76	R-->E	
2BKMA	73 - 77	76	R-->G	
2BKMA	73 - 77	76	R-->I	
2BKMA	73 - 77	76	R-->P	
2BKMA	73 - 77	76	R-->S	
2BKMA	73 - 77	77	H-->F	
2BKMA	73 - 77	77	H-->G	
2BKMA	73 - 77	77	H-->K	
2BKMA	73 - 77	77	H-->P	

2BKMA	73	77	77	H-->S
2BKMA	73	77	77	H-->T
2BKMA	73	77	77	H-->V
1YN9C	71	75	71	V-->C
1YN9C	71	75	71	V-->G
1YN9C	71	75	71	V-->S
1YN9C	71	75	72	H-->A
1YN9C	71	75	72	H-->F
1YN9C	71	75	72	H-->G
1YN9C	71	75	72	H-->Q
1YN9C	71	75	72	H-->R
1YN9C	71	75	72	H-->S
1YN9C	71	75	72	H-->T
1YN9C	71	75	72	H-->V
1YN9C	71	75	73	F-->G
1YN9C	71	75	73	F-->L
1YN9C	71	75	73	F-->T
1YN9C	71	75	73	F-->V
1YN9C	71	75	74	L-->E
1YN9C	71	75	74	L-->H
1YN9C	71	75	74	L-->Q
1YN9C	71	75	75	R-->G
1YN9C	71	75	75	R-->K
1YN9C	71	75	75	R-->P
2PU3A	84	88	84	A-->L
2PU3A	84	88	84	A-->Y
2PU3A	84	88	85	W-->F

2PU3A	84	88	85	W-->G
2PU3A	84	88	85	W-->L
2PU3A	84	88	85	W-->P
2PU3A	84	88	85	W-->R
2PU3A	84	88	85	W-->T
2PU3A	84	88	86	Q-->F
2PU3A	84	88	86	Q-->L
2PU3A	84	88	86	Q-->S
2PU3A	84	88	87	F-->C
2PU3A	84	88	88	G-->H
2PU3A	84	88	88	G-->I
2PU3A	84	88	88	G-->Q
2PU3A	124	130	127	N-->I
1DQZA	124	128	124	G-->A
1DQZA	124	128	124	G-->K
1DQZA	124	128	124	G-->L
1DQZA	124	128	124	G-->P
1DQZA	124	128	124	G-->R
1DQZA	124	128	124	G-->S
1DQZA	124	128	125	W-->A
1DQZA	124	128	125	W-->C
1DQZA	124	128	125	W-->D
1DQZA	124	128	125	W-->F
1DQZA	124	128	125	W-->G
1DQZA	124	128	125	W-->I
1DQZA	124	128	125	W-->L
1DQZA	124	128	125	W-->N

1DQZA	124	128	125	W-->Q
1DQZA	124	128	125	W-->R
1DQZA	124	128	125	W-->S
1DQZA	124	128	125	W-->T
1DQZA	124	128	125	W-->V
1DQZA	124	128	125	W-->Y
1DQZA	124	128	126	D-->I
1DQZA	124	128	126	D-->K
1DQZA	124	128	126	D-->Y
1DQZA	124	128	127	I-->A
1DQZA	124	128	127	I-->N
1DQZA	124	128	127	I-->W
1DQZA	124	128	128	N-->E
1DQZA	124	128	128	N-->S
1DQZA	55	59	55	A-->C
1DQZA	55	59	55	A-->D
1DQZA	55	59	55	A-->G
1DQZA	55	59	55	A-->V
1DQZA	55	59	56	F-->G
1DQZA	55	59	56	F-->I
1DQZA	55	59	56	F-->T
1DQZA	55	59	57	E-->I
1DQZA	55	59	57	E-->S
1DQZA	55	59	58	E-->K
1DQZA	55	59	58	E-->P
1DQZA	55	59	58	E-->V
1DQZA	55	59	59	Y-->A

1DQZA	55	- 59	59	Y-->D
1DQZA	55	- 59	59	Y-->E
1DQZA	55	- 59	59	Y-->G
1DQZA	55	- 59	59	Y-->H
1DQZA	95	- 100	95	W-->K
1DQZA	174	- 178	174	A-->E
1DQZA	174	- 178	176	S-->H
1DQZA	174	- 178	176	S-->N
1DQZA	174	- 178	177	M-->I
1DQZA	174	- 178	177	M-->Y
1DQZA	174	- 178	178	W-->V
1DQZA	184	- 188	184	P-->K
1DQZA	184	- 188	185	A-->F
1DQZA	184	- 188	185	A-->R
1DQZA	184	- 188	186	W-->D
1DQZA	184	- 188	186	W-->G
1DQZA	184	- 188	186	W-->K
1DQZA	184	- 188	186	W-->L
1DQZA	184	- 188	186	W-->N
1DQZA	184	- 188	186	W-->P
1DQZA	184	- 188	186	W-->T
1DQZA	184	- 188	186	W-->V
1DQZA	184	- 188	187	K-->G
1DQZA	184	- 188	188	R-->G
1DQZA	184	- 188	188	R-->P
1DQZA	184	- 188	188	R-->Q
1X82A	161	- 166	165	E-->L

1X82A	161 - 166	166	K-->L
1X82A	161 - 166	166	K-->N
3C9QA	135 - 141	138	Y-->S
1QWOA	317 - 321	317	S-->A
1QWOA	317 - 321	317	S-->D
1QWOA	317 - 321	317	S-->E
1QWOA	317 - 321	317	S-->F
1QWOA	317 - 321	317	S-->G
1QWOA	317 - 321	317	S-->I
1QWOA	317 - 321	317	S-->K
1QWOA	317 - 321	317	S-->L
1QWOA	317 - 321	317	S-->P
1QWOA	317 - 321	317	S-->Q
1QWOA	317 - 321	317	S-->V
1QWOA	317 - 321	318	T-->A
1QWOA	317 - 321	318	T-->D
1QWOA	317 - 321	318	T-->E
1QWOA	317 - 321	318	T-->G
1QWOA	317 - 321	318	T-->H
1QWOA	317 - 321	318	T-->I
1QWOA	317 - 321	318	T-->K
1QWOA	317 - 321	318	T-->L
1QWOA	317 - 321	318	T-->P
1QWOA	317 - 321	318	T-->R
1QWOA	317 - 321	318	T-->S
1QWOA	317 - 321	318	T-->V
1QWOA	317 - 321	319	L-->A

1QWOA	317 - 321	319	L-->D
1QWOA	317 - 321	319	L-->H
1QWOA	317 - 321	319	L-->P
1QWOA	317 - 321	319	L-->R
1QWOA	317 - 321	319	L-->S
1QWOA	317 - 321	319	L-->T
1QWOA	317 - 321	319	L-->V
1QWOA	317 - 321	320	V-->A
1QWOA	317 - 321	320	V-->D
1QWOA	317 - 321	320	V-->E
1QWOA	317 - 321	320	V-->G
1QWOA	317 - 321	320	V-->H
1QWOA	317 - 321	320	V-->K
1QWOA	317 - 321	320	V-->L
1QWOA	317 - 321	320	V-->N
1QWOA	317 - 321	320	V-->Q
1QWOA	317 - 321	320	V-->R
1QWOA	317 - 321	320	V-->T
1QWOA	317 - 321	321	S-->A
1QWOA	317 - 321	321	S-->D
1QWOA	317 - 321	321	S-->E
1QWOA	317 - 321	321	S-->F
1QWOA	317 - 321	321	S-->G
1QWOA	317 - 321	321	S-->I
1QWOA	317 - 321	321	S-->L
1QWOA	317 - 321	321	S-->N
1QWOA	317 - 321	321	S-->R

1QWOA	431 - 435	432	W-->C
1QWOA	431 - 435	432	W-->E
1QWOA	431 - 435	432	W-->F
1QWOA	431 - 435	432	W-->G
1QWOA	431 - 435	432	W-->H
1QWOA	431 - 435	432	W-->P
1QWOA	431 - 435	432	W-->R
1QWOA	431 - 435	433	A-->E
1QWOA	431 - 435	433	A-->H
1QWOA	431 - 435	434	R-->T
1QWOA	431 - 435	435	S-->G
1QWOA	431 - 435	435	S-->L
1NF9A	90 - 94	90	L-->D
1NF9A	90 - 94	90	L-->E
1NF9A	90 - 94	90	L-->N
1NF9A	90 - 94	91	K-->F
1NF9A	90 - 94	91	K-->L
1NF9A	90 - 94	91	K-->R
1NF9A	90 - 94	91	K-->V
1NF9A	90 - 94	92	D-->E
1NF9A	90 - 94	93	F-->D
1NF9A	90 - 94	93	F-->P
1NF9A	90 - 94	94	W-->A
1NF9A	90 - 94	94	W-->D
1NF9A	90 - 94	94	W-->E
1NF9A	90 - 94	94	W-->G
1NF9A	90 - 94	94	W-->H

1NF9A	90 - 94	94	W-->K
1NF9A	90 - 94	94	W-->L
1NF9A	90 - 94	94	W-->N
1NF9A	90 - 94	94	W-->P
1NF9A	90 - 94	94	W-->S
1NF9A	90 - 94	94	W-->T
1NF9A	202 - 206	202	T-->A
1NF9A	202 - 206	202	T-->D
1NF9A	202 - 206	202	T-->E
1NF9A	202 - 206	202	T-->G
1NF9A	202 - 206	202	T-->H
1NF9A	202 - 206	202	T-->I
1NF9A	202 - 206	202	T-->K
1NF9A	202 - 206	202	T-->N
1NF9A	202 - 206	202	T-->P
1NF9A	202 - 206	202	T-->V
1NF9A	202 - 206	202	T-->Y
1NF9A	202 - 206	203	D-->A
1NF9A	202 - 206	203	D-->C
1NF9A	202 - 206	203	D-->E
1NF9A	202 - 206	203	D-->F
1NF9A	202 - 206	203	D-->G
1NF9A	202 - 206	203	D-->H
1NF9A	202 - 206	203	D-->L
1NF9A	202 - 206	203	D-->P
1NF9A	202 - 206	203	D-->Q
1NF9A	202 - 206	203	D-->R

1NF9A	202	206	203	D-->V
1NF9A	202	206	204	E-->F
1NF9A	202	206	204	E-->G
1NF9A	202	206	204	E-->I
1NF9A	202	206	204	E-->L
1NF9A	202	206	204	E-->P
1NF9A	202	206	204	E-->R
1NF9A	202	206	204	E-->S
1NF9A	202	206	204	E-->V
1NF9A	202	206	204	E-->Y
1NF9A	202	206	205	V-->D
1NF9A	202	206	205	V-->E
1NF9A	202	206	205	V-->H
1NF9A	202	206	205	V-->K
1NF9A	202	206	205	V-->P
1NF9A	202	206	205	V-->Q
1NF9A	202	206	205	V-->S
1NF9A	202	206	205	V-->Y
1NF9A	202	206	206	L-->A
1NF9A	202	206	206	L-->H
1NF9A	202	206	206	L-->K
1NF9A	202	206	206	L-->P
1NF9A	202	206	206	L-->R
1NF9A	202	206	206	L-->T
1NF9A	202	206	206	L-->V
1NF9A	202	206	206	L-->Y
2G1UA	40	45	45	R-->G

2G1UA	62 - 66	62	F-->A
2G1UA	62 - 66	62	F-->D
2G1UA	62 - 66	62	F-->I
2G1UA	62 - 66	62	F-->K
2G1UA	62 - 66	62	F-->P
2G1UA	62 - 66	62	F-->S
2G1UA	62 - 66	62	F-->T
2G1UA	62 - 66	62	F-->V
2G1UA	62 - 66	63	E-->A
2G1UA	62 - 66	63	E-->D
2G1UA	62 - 66	63	E-->F
2G1UA	62 - 66	63	E-->I
2G1UA	62 - 66	63	E-->K
2G1UA	62 - 66	63	E-->L
2G1UA	62 - 66	63	E-->P
2G1UA	62 - 66	63	E-->Q
2G1UA	62 - 66	63	E-->R
2G1UA	62 - 66	63	E-->T
2G1UA	62 - 66	64	T-->C
2G1UA	62 - 66	64	T-->E
2G1UA	62 - 66	64	T-->G
2G1UA	62 - 66	64	T-->K
2G1UA	62 - 66	64	T-->L
2G1UA	62 - 66	65	L-->A
2G1UA	62 - 66	65	L-->K
2G1UA	62 - 66	65	L-->N
2G1UA	62 - 66	65	L-->P

2G1UA	62	66	65	L-->R
2G1UA	62	66	66	K-->A
2G1UA	62	66	66	K-->L
2G1UA	62	66	66	K-->P
2G1UA	62	66	66	K-->S
2G1UA	62	66	66	K-->V
2IWRA	130	135	130	A-->E
2IWRA	130	135	131	K-->P
2IWRA	130	135	135	W-->F
2IWRA	130	135	135	W-->N
1VQSA	74	79	76	W-->A
1VQSA	74	79	76	W-->K
1VQSA	74	79	79	F-->V
1ZKPB	74	78	74	V-->A
1ZKPB	74	78	74	V-->C
1ZKPB	74	78	74	V-->D
1ZKPB	74	78	74	V-->G
1ZKPB	74	78	74	V-->H
1ZKPB	74	78	74	V-->R
1ZKPB	74	78	74	V-->S
1ZKPB	74	78	75	L-->D
1ZKPB	74	78	75	L-->G
1ZKPB	74	78	75	L-->H
1ZKPB	74	78	75	L-->K
1ZKPB	74	78	75	L-->N
1ZKPB	74	78	75	L-->P
1ZKPB	74	78	75	L-->R

1ZKPB	74 - 78	75	L-->T
1ZKPB	74 - 78	75	L-->V
1ZKPB	74 - 78	76	A-->D
1ZKPB	74 - 78	76	A-->G
1ZKPB	74 - 78	76	A-->L
1ZKPB	74 - 78	76	A-->N
1ZKPB	74 - 78	76	A-->Q
1ZKPB	74 - 78	77	Q-->A
1ZKPB	74 - 78	77	Q-->F
1ZKPB	74 - 78	77	Q-->L
1ZKPB	74 - 78	77	Q-->P
1ZKPB	74 - 78	77	Q-->S
1ZKPB	74 - 78	77	Q-->T
1ZKPB	74 - 78	77	Q-->V
1ZKPB	74 - 78	77	Q-->Y
1ZKPB	74 - 78	78	L-->A
1ZKPB	74 - 78	78	L-->D
1ZKPB	74 - 78	78	L-->F
1ZKPB	74 - 78	78	L-->G
1ZKPB	74 - 78	78	L-->N
1ZKPB	74 - 78	78	L-->R
1ZKPB	74 - 78	78	L-->S
1ZKPB	74 - 78	78	L-->T
1ZKPB	74 - 78	78	L-->V
1ZKPB	74 - 78	78	L-->Y
1ZKPB	162 - 166	162	F-->C
1ZKPB	162 - 166	162	F-->D

1ZKPB	162 - 166	162	F-->E
1ZKPB	162 - 166	162	F-->G
1ZKPB	162 - 166	162	F-->H
1ZKPB	162 - 166	162	F-->I
1ZKPB	162 - 166	162	F-->L
1ZKPB	162 - 166	162	F-->S
1ZKPB	162 - 166	162	F-->T
1ZKPB	162 - 166	162	F-->Y
1ZKPB	162 - 166	163	I-->H
1ZKPB	162 - 166	163	I-->K
1ZKPB	162 - 166	163	I-->L
1ZKPB	162 - 166	163	I-->P
1ZKPB	162 - 166	163	I-->S
1ZKPB	162 - 166	163	I-->T
1ZKPB	162 - 166	164	P-->D
1ZKPB	162 - 166	164	P-->K
1ZKPB	162 - 166	164	P-->N
1ZKPB	162 - 166	165	F-->A
1ZKPB	162 - 166	165	F-->G
1ZKPB	162 - 166	165	F-->L
1ZKPB	162 - 166	165	F-->N
1ZKPB	162 - 166	165	F-->P
1ZKPB	162 - 166	165	F-->R
1ZKPB	162 - 166	165	F-->S
1ZKPB	162 - 166	165	F-->V
1ZKPB	162 - 166	166	T-->A
1ZKPB	162 - 166	166	T-->N

1ZKPB	162 - 166	166	T-->Q
1ZKPB	162 - 166	166	T-->R
1ZKPB	162 - 166	166	T-->S
2QL8A	121 - 126	121	P-->L
2QL8A	121 - 126	122	V-->A
2QL8A	121 - 126	123	S-->D
2QL8A	121 - 126	123	S-->E
2QL8A	121 - 126	123	S-->P
2QL8A	121 - 126	124	K-->A
2QL8A	121 - 126	124	K-->P
2QL8A	121 - 126	125	L-->E
2QL8A	121 - 126	125	L-->K
8ABPA	294 - 300	296	E-->S
1Z1SA	48 - 53	51	I-->V
1WPUA	26 - 32	29	E-->V
2IMSA	167 - 173	170	W-->A
2OLMA	130 - 134	130	P-->A
2OLMA	130 - 134	130	P-->D
2OLMA	130 - 134	130	P-->T
2OLMA	130 - 134	130	P-->W
2OLMA	130 - 134	131	E-->K
2OLMA	130 - 134	131	E-->Q
2OLMA	130 - 134	131	E-->S
2OLMA	130 - 134	131	E-->W
2OLMA	130 - 134	131	E-->Y
2OLMA	130 - 134	132	Q-->E
2OLMA	130 - 134	132	Q-->G

2OLMA	130 - 134	132	Q-->H
2OLMA	130 - 134	132	Q-->K
2OLMA	130 - 134	132	Q-->M
2OLMA	130 - 134	132	Q-->P
2OLMA	130 - 134	133	A-->D
2OLMA	130 - 134	133	A-->E
2OLMA	130 - 134	133	A-->F
2OLMA	130 - 134	133	A-->K
2OLMA	130 - 134	133	A-->L
2OLMA	130 - 134	133	A-->M
2OLMA	130 - 134	134	K-->F
2OLMA	130 - 134	134	K-->V
1RKUA	157 - 162	159	M-->G
1RKUA	157 - 162	159	M-->P
1RKUA	173 - 178	178	E-->G
1RKUA	173 - 178	178	E-->Q
1O82A	64 - 69	64	K-->H
1O82A	64 - 69	64	K-->I
1O82A	64 - 69	64	K-->Q
1O82A	64 - 69	65	R-->I
1O82A	64 - 69	65	R-->Q
1O82A	64 - 69	66	A-->V
1O82A	64 - 69	67	V-->F
1O82A	64 - 69	69	A-->L
1O82A	64 - 69	69	A-->T
2FB6A	16 - 21	17	D-->I
2FB6A	16 - 21	18	T-->R

2FB6A	84 - 89	84	A-->I
2FB6A	84 - 89	85	S-->K
2FB6A	84 - 89	86	I-->T
2FB6A	84 - 89	87	I-->F
1X2IA	34 - 39	34	V-->R
1X2IA	34 - 39	35	E-->G
1X2IA	34 - 39	36	R-->D
1X2IA	34 - 39	38	F-->E
1X2IA	34 - 39	39	T-->I
2PQ8A	199 - 203	199	E-->F
2PQ8A	199 - 203	199	E-->H
2PQ8A	199 - 203	200	D-->F
2PQ8A	199 - 203	200	D-->K
2PQ8A	199 - 203	200	D-->L
2PQ8A	199 - 203	200	D-->Q
2PQ8A	199 - 203	200	D-->T
2PQ8A	199 - 203	200	D-->V
2PQ8A	199 - 203	200	D-->W
2PQ8A	199 - 203	201	Y-->A
2PQ8A	199 - 203	201	Y-->D
2PQ8A	199 - 203	201	Y-->E
2PQ8A	199 - 203	201	Y-->F
2PQ8A	199 - 203	201	Y-->K
2PQ8A	199 - 203	201	Y-->L
2PQ8A	199 - 203	201	Y-->P
2PQ8A	199 - 203	201	Y-->T
2PQ8A	199 - 203	201	Y-->V

2PQ8A	199 - 203	202	G-->A
2PQ8A	199 - 203	202	G-->D
2PQ8A	199 - 203	202	G-->F
2PQ8A	199 - 203	202	G-->K
2PQ8A	199 - 203	202	G-->P
2PQ8A	199 - 203	202	G-->Q
2PQ8A	199 - 203	202	G-->R
2PQ8A	199 - 203	202	G-->V
2PQ8A	199 - 203	202	G-->Y
2PQ8A	199 - 203	203	K-->D
2PQ8A	199 - 203	203	K-->G
2PQ8A	199 - 203	203	K-->Q
2PQ8A	199 - 203	203	K-->R
2PQ8A	199 - 203	203	K-->S
1IDPA	35 - 39	35	W-->F
1IDPA	35 - 39	35	W-->G
1IDPA	35 - 39	35	W-->I
1IDPA	35 - 39	35	W-->K
1IDPA	35 - 39	35	W-->L
1IDPA	35 - 39	35	W-->P
1IDPA	35 - 39	35	W-->Y
1IDPA	35 - 39	36	D-->L
1IDPA	35 - 39	37	R-->E
1IDPA	35 - 39	37	R-->S
1IDPA	35 - 39	38	L-->E
1IDPA	35 - 39	38	L-->R
1IDPA	35 - 39	39	R-->K

1BGFA	28 - 33	32	H-->G
1BGFA	35 - 40	37	W-->G
1BGFA	35 - 40	37	W-->K
1HLQC	10 - 15	11	N-->Q
1HLQC	10 - 15	13	K-->E
1HLQC	10 - 15	14	S-->A
1HLQC	10 - 15	14	S-->V
1HLQC	10 - 15	15	L-->A
1GVDA	115 - 119	115	W-->A
1GVDA	115 - 119	115	W-->D
1GVDA	115 - 119	115	W-->F
1GVDA	115 - 119	115	W-->G
1GVDA	115 - 119	115	W-->I
1GVDA	115 - 119	115	W-->K
1GVDA	115 - 119	115	W-->L
1GVDA	115 - 119	115	W-->M
1GVDA	115 - 119	115	W-->N
1GVDA	115 - 119	115	W-->P
1GVDA	115 - 119	115	W-->Q
1GVDA	115 - 119	115	W-->V
1GVDA	115 - 119	115	W-->Y
1GVDA	115 - 119	116	S-->K
1GVDA	115 - 119	117	V-->A
1GVDA	115 - 119	117	V-->E
1GVDA	115 - 119	117	V-->H
1GVDA	115 - 119	117	V-->K
1GVDA	115 - 119	118	I-->H

1GVDA	115 - 119	118	I-->P
1GVDA	115 - 119	119	A-->D
1GVDA	115 - 119	119	A-->I
2ODIA	189 - 193	189	T-->E
2ODIA	189 - 193	189	T-->F
2ODIA	189 - 193	189	T-->G
2ODIA	189 - 193	189	T-->N
2ODIA	189 - 193	189	T-->V
2ODIA	189 - 193	190	S-->A
2ODIA	189 - 193	190	S-->D
2ODIA	189 - 193	190	S-->F
2ODIA	189 - 193	190	S-->G
2ODIA	189 - 193	190	S-->I
2ODIA	189 - 193	190	S-->L
2ODIA	189 - 193	190	S-->N
2ODIA	189 - 193	190	S-->Q
2ODIA	189 - 193	190	S-->R
2ODIA	189 - 193	190	S-->T
2ODIA	189 - 193	190	S-->V
2ODIA	189 - 193	191	L-->C
2ODIA	189 - 193	191	L-->F
2ODIA	189 - 193	191	L-->G
2ODIA	189 - 193	191	L-->K
2ODIA	189 - 193	191	L-->P
2ODIA	189 - 193	191	L-->V
2ODIA	189 - 193	192	I-->G
2ODIA	189 - 193	192	I-->K

2ODIA	189 - 193	192	I-->M
2ODIA	189 - 193	192	I-->P
2ODIA	189 - 193	192	I-->Q
2ODIA	189 - 193	192	I-->T
2ODIA	189 - 193	192	I-->V
2ODIA	189 - 193	193	N-->A
2ODIA	189 - 193	193	N-->E
2ODIA	189 - 193	193	N-->F
2ODIA	189 - 193	193	N-->L
2ODIA	189 - 193	193	N-->P
2ODIA	189 - 193	193	N-->Q
2ODIA	189 - 193	193	N-->S
2ODIA	189 - 193	193	N-->T
2ODIA	189 - 193	193	N-->Y
2ODIA	224 - 230	225	S-->D
1ZKKA	341 - 346	341	K-->S
1ZKKA	341 - 346	343	S-->R
1ZKKA	341 - 346	345	E-->L
1ZKKA	341 - 346	345	E-->T
1ZKKA	341 - 346	346	A-->I
1Z67A	118 - 122	118	L-->A
1Z67A	118 - 122	118	L-->D
1Z67A	118 - 122	118	L-->E
1Z67A	118 - 122	118	L-->F
1Z67A	118 - 122	118	L-->G
1Z67A	118 - 122	118	L-->H
1Z67A	118 - 122	118	L-->I

1Z67A	118	122	118	L-->M
1Z67A	118	122	118	L-->N
1Z67A	118	122	118	L-->P
1Z67A	118	122	118	L-->Q
1Z67A	118	122	118	L-->R
1Z67A	118	122	118	L-->S
1Z67A	118	122	118	L-->T
1Z67A	118	122	118	L-->V
1Z67A	118	122	118	L-->W
1Z67A	118	122	118	L-->Y
1Z67A	118	122	119	L-->A
1Z67A	118	122	119	L-->C
1Z67A	118	122	119	L-->D
1Z67A	118	122	119	L-->E
1Z67A	118	122	119	L-->F
1Z67A	118	122	119	L-->G
1Z67A	118	122	119	L-->H
1Z67A	118	122	119	L-->I
1Z67A	118	122	119	L-->K
1Z67A	118	122	119	L-->P
1Z67A	118	122	119	L-->S
1Z67A	118	122	119	L-->T
1Z67A	118	122	119	L-->V
1Z67A	118	122	119	L-->Y
1Z67A	118	122	120	S-->A
1Z67A	118	122	120	S-->C
1Z67A	118	122	120	S-->D

1Z67A	118	122	120	S-->E
1Z67A	118	122	120	S-->F
1Z67A	118	122	120	S-->G
1Z67A	118	122	120	S-->H
1Z67A	118	122	120	S-->I
1Z67A	118	122	120	S-->K
1Z67A	118	122	120	S-->L
1Z67A	118	122	120	S-->P
1Z67A	118	122	120	S-->Q
1Z67A	118	122	120	S-->T
1Z67A	118	122	120	S-->V
1Z67A	118	122	121	A-->C
1Z67A	118	122	121	A-->D
1Z67A	118	122	121	A-->E
1Z67A	118	122	121	A-->G
1Z67A	118	122	121	A-->H
1Z67A	118	122	121	A-->I
1Z67A	118	122	121	A-->L
1Z67A	118	122	121	A-->P
1Z67A	118	122	121	A-->Q
1Z67A	118	122	121	A-->R
1Z67A	118	122	121	A-->S
1Z67A	118	122	121	A-->T
1Z67A	118	122	121	A-->V
1Z67A	118	122	122	G-->A
1Z67A	118	122	122	G-->D
1Z67A	118	122	122	G-->E

1Z67A	118	- 122	122	G-->I
1Z67A	118	- 122	122	G-->L
1Z67A	118	- 122	122	G-->N
1Z67A	118	- 122	122	G-->R
1Z67A	118	- 122	122	G-->S
1Z67A	118	- 122	122	G-->T
1Z67A	118	- 122	122	G-->V
1Z67A	124	- 130	129	K-->S
1WL8A	36	- 41	36	L-->G
1WL8A	36	- 41	37	E-->L
1WL8A	36	- 41	38	E-->P
1WL8A	36	- 41	38	E-->V
1WL8A	36	- 41	39	I-->G
1WL8A	36	- 41	41	A-->E
1WL8A	36	- 41	41	A-->T
1WL8A	61	- 66	63	K-->T
2NN5A	-8	- -1	-8	E-->A
2NN5A	-8	- -1	-3	Q-->S
2QJLA	49	- 55	50	N-->K
2DPLB	192	- 196	192	G-->A
2DPLB	192	- 196	192	G-->D
2DPLB	192	- 196	192	G-->I
2DPLB	192	- 196	192	G-->K
2DPLB	192	- 196	192	G-->P
2DPLB	192	- 196	192	G-->R
2DPLB	192	- 196	192	G-->S
2DPLB	192	- 196	192	G-->V

2DPLB	192 - 196	193	L-->A
2DPLB	192 - 196	193	L-->D
2DPLB	192 - 196	193	L-->E
2DPLB	192 - 196	193	L-->G
2DPLB	192 - 196	193	L-->I
2DPLB	192 - 196	193	L-->K
2DPLB	192 - 196	193	L-->P
2DPLB	192 - 196	193	L-->Q
2DPLB	192 - 196	193	L-->R
2DPLB	192 - 196	193	L-->S
2DPLB	192 - 196	193	L-->T
2DPLB	192 - 196	193	L-->V
2DPLB	192 - 196	193	L-->Y
2DPLB	192 - 196	194	A-->C
2DPLB	192 - 196	194	A-->D
2DPLB	192 - 196	194	A-->E
2DPLB	192 - 196	194	A-->F
2DPLB	192 - 196	194	A-->G
2DPLB	192 - 196	194	A-->H
2DPLB	192 - 196	194	A-->I
2DPLB	192 - 196	194	A-->K
2DPLB	192 - 196	194	A-->L
2DPLB	192 - 196	194	A-->N
2DPLB	192 - 196	194	A-->P
2DPLB	192 - 196	194	A-->R
2DPLB	192 - 196	194	A-->S
2DPLB	192 - 196	194	A-->T

2DPLB	192 - 196	194	A-->V
2DPLB	192 - 196	195	V-->A
2DPLB	192 - 196	195	V-->C
2DPLB	192 - 196	195	V-->D
2DPLB	192 - 196	195	V-->E
2DPLB	192 - 196	195	V-->F
2DPLB	192 - 196	195	V-->K
2DPLB	192 - 196	195	V-->M
2DPLB	192 - 196	195	V-->T
2DPLB	192 - 196	195	V-->Y
2DPLB	192 - 196	196	R-->D
2DPLB	192 - 196	196	R-->E
2DPLB	192 - 196	196	R-->F
2DPLB	192 - 196	196	R-->G
2DPLB	192 - 196	196	R-->I
2DPLB	192 - 196	196	R-->K
2DPLB	192 - 196	196	R-->L
2DPLB	192 - 196	196	R-->M
2DPLB	192 - 196	196	R-->N
2DPLB	192 - 196	196	R-->P
2DPLB	192 - 196	196	R-->S
2DPLB	192 - 196	196	R-->T
2DPLB	192 - 196	196	R-->V
2DPLB	192 - 196	196	R-->Y
2PQXA	93 - 98	95	R-->I
2PQXA	93 - 98	96	M-->N
2PQXA	187 - 192	188	E-->L

2PQXA	187 - 192	191	K-->V
1NOFA	240 - 244	240	P-->D
1NOFA	240 - 244	240	P-->G
1NOFA	240 - 244	240	P-->H
1NOFA	240 - 244	240	P-->Q
1NOFA	240 - 244	240	P-->V
1NOFA	240 - 244	241	L-->E
1NOFA	240 - 244	241	L-->G
1NOFA	240 - 244	241	L-->K
1NOFA	240 - 244	241	L-->S
1NOFA	240 - 244	242	A-->N
1NOFA	240 - 244	242	A-->P
1NOFA	240 - 244	242	A-->Q
1NOFA	240 - 244	242	A-->S
1NOFA	240 - 244	242	A-->V
1NOFA	240 - 244	243	Q-->D
1NOFA	240 - 244	243	Q-->E
1NOFA	240 - 244	243	Q-->G
1NOFA	240 - 244	243	Q-->K
1NOFA	240 - 244	243	Q-->L
1NOFA	240 - 244	243	Q-->N
1NOFA	240 - 244	243	Q-->P
1NOFA	240 - 244	243	Q-->S
1NOFA	240 - 244	243	Q-->T
1NOFA	240 - 244	243	Q-->V
1NOFA	240 - 244	243	Q-->Y
1NOFA	240 - 244	244	N-->C

1NOFA	240 - 244	244	N-->F
1NOFA	240 - 244	244	N-->G
1NOFA	240 - 244	244	N-->I
1NOFA	240 - 244	244	N-->K
1NOFA	240 - 244	244	N-->P
1NOFA	240 - 244	244	N-->Q
2BWQA	805 - 810	805	R-->F
2BWQA	805 - 810	806	R-->I
2BWQA	805 - 810	806	R-->W
2BWQA	805 - 810	808	F-->A
2POFA	184 - 189	189	Q-->N
1V30A	184 - 188	184	E-->A
1V30A	184 - 188	184	E-->C
1V30A	184 - 188	184	E-->D
1V30A	184 - 188	184	E-->F
1V30A	184 - 188	184	E-->G
1V30A	184 - 188	184	E-->I
1V30A	184 - 188	184	E-->K
1V30A	184 - 188	184	E-->L
1V30A	184 - 188	184	E-->N
1V30A	184 - 188	184	E-->P
1V30A	184 - 188	184	E-->S
1V30A	184 - 188	184	E-->T
1V30A	184 - 188	184	E-->V
1V30A	184 - 188	184	E-->Y
1V30A	184 - 188	185	I-->D
1V30A	184 - 188	185	I-->E

1V30A	184	188	185	I-->F
1V30A	184	188	185	I-->G
1V30A	184	188	185	I-->L
1V30A	184	188	185	I-->N
1V30A	184	188	185	I-->P
1V30A	184	188	185	I-->R
1V30A	184	188	185	I-->S
1V30A	184	188	185	I-->T
1V30A	184	188	185	I-->V
1V30A	184	188	186	G-->A
1V30A	184	188	186	G-->E
1V30A	184	188	186	G-->F
1V30A	184	188	186	G-->I
1V30A	184	188	186	G-->K
1V30A	184	188	186	G-->L
1V30A	184	188	186	G-->N
1V30A	184	188	186	G-->Q
1V30A	184	188	186	G-->R
1V30A	184	188	186	G-->Y
1V30A	184	188	187	T-->A
1V30A	184	188	187	T-->C
1V30A	184	188	187	T-->D
1V30A	184	188	187	T-->E
1V30A	184	188	187	T-->F
1V30A	184	188	187	T-->G
1V30A	184	188	187	T-->H
1V30A	184	188	187	T-->I

1V30A	184 - 188	187	T-->L	
1V30A	184 - 188	187	T-->N	
1V30A	184 - 188	187	T-->P	
1V30A	184 - 188	187	T-->Q	
1V30A	184 - 188	187	T-->R	
1V30A	184 - 188	187	T-->S	
1V30A	184 - 188	187	T-->V	
1V30A	184 - 188	188	G-->D	
1V30A	184 - 188	188	G-->F	
1V30A	184 - 188	188	G-->H	
1V30A	184 - 188	188	G-->I	
1V30A	184 - 188	188	G-->K	
1V30A	184 - 188	188	G-->L	
1V30A	184 - 188	188	G-->S	
1V30A	184 - 188	188	G-->V	
1IE9A	217 - 223	223	Q-->G	
1IE9A	297 - 301	297	V-->A	
1IE9A	297 - 301	297	V-->D	
1IE9A	297 - 301	297	V-->F	
1IE9A	297 - 301	297	V-->G	
1IE9A	297 - 301	297	V-->H	
1IE9A	297 - 301	297	V-->I	
1IE9A	297 - 301	297	V-->L	
1IE9A	297 - 301	297	V-->N	
1IE9A	297 - 301	297	V-->P	
1IE9A	297 - 301	297	V-->T	
1IE9A	297 - 301	297	V-->Y	

1IE9A	297 - 301	298	S-->A
1IE9A	297 - 301	298	S-->E
1IE9A	297 - 301	298	S-->F
1IE9A	297 - 301	298	S-->G
1IE9A	297 - 301	298	S-->H
1IE9A	297 - 301	298	S-->I
1IE9A	297 - 301	298	S-->K
1IE9A	297 - 301	298	S-->L
1IE9A	297 - 301	298	S-->M
1IE9A	297 - 301	298	S-->P
1IE9A	297 - 301	298	S-->Q
1IE9A	297 - 301	298	S-->V
1IE9A	297 - 301	298	S-->W
1IE9A	297 - 301	298	S-->Y
1IE9A	297 - 301	299	D-->A
1IE9A	297 - 301	299	D-->C
1IE9A	297 - 301	299	D-->E
1IE9A	297 - 301	299	D-->G
1IE9A	297 - 301	299	D-->K
1IE9A	297 - 301	299	D-->L
1IE9A	297 - 301	299	D-->M
1IE9A	297 - 301	299	D-->N
1IE9A	297 - 301	299	D-->P
1IE9A	297 - 301	299	D-->R
1IE9A	297 - 301	299	D-->S
1IE9A	297 - 301	299	D-->T
1IE9A	297 - 301	299	D-->V

1IE9A	297 - 301	300	V-->A
1IE9A	297 - 301	300	V-->E
1IE9A	297 - 301	300	V-->F
1IE9A	297 - 301	300	V-->G
1IE9A	297 - 301	300	V-->K
1IE9A	297 - 301	300	V-->N
1IE9A	297 - 301	300	V-->P
1IE9A	297 - 301	300	V-->Q
1IE9A	297 - 301	300	V-->R
1IE9A	297 - 301	300	V-->S
1IE9A	297 - 301	300	V-->T
1IE9A	297 - 301	300	V-->W
1IE9A	297 - 301	301	T-->A
1IE9A	297 - 301	301	T-->C
1IE9A	297 - 301	301	T-->D
1IE9A	297 - 301	301	T-->E
1IE9A	297 - 301	301	T-->G
1IE9A	297 - 301	301	T-->H
1IE9A	297 - 301	301	T-->I
1IE9A	297 - 301	301	T-->N
1IE9A	297 - 301	301	T-->P
1IE9A	297 - 301	301	T-->S
1IE9A	297 - 301	301	T-->W
1IE9A	297 - 301	301	T-->Y
1IE9A	416 - 422	421	V-->E
2FQ3A	321 - 326	323	E-->F
2OHWA	123 - 128	127	R-->Y

2OHWA	123 - 128	128	S-->L
2EW0A	107 - 113	108	I-->H
1PFBA	67 - 73	72	Y-->G
1T7RA	67 - 71	67	V-->A
1T7RA	67 - 71	67	V-->C
1T7RA	67 - 71	67	V-->D
1T7RA	67 - 71	67	V-->E
1T7RA	67 - 71	67	V-->G
1T7RA	67 - 71	67	V-->I
1T7RA	67 - 71	67	V-->K
1T7RA	67 - 71	67	V-->M
1T7RA	67 - 71	67	V-->N
1T7RA	67 - 71	67	V-->S
1T7RA	67 - 71	68	P-->A
1T7RA	67 - 71	68	P-->E
1T7RA	67 - 71	68	P-->R
1T7RA	67 - 71	68	P-->T
1T7RA	67 - 71	68	P-->V
1T7RA	67 - 71	69	K-->A
1T7RA	67 - 71	69	K-->C
1T7RA	67 - 71	69	K-->D
1T7RA	67 - 71	69	K-->E
1T7RA	67 - 71	69	K-->F
1T7RA	67 - 71	69	K-->G
1T7RA	67 - 71	69	K-->I
1T7RA	67 - 71	69	K-->L
1T7RA	67 - 71	69	K-->M

1T7RA	67 - 71	69	K-->P
1T7RA	67 - 71	69	K-->R
1T7RA	67 - 71	69	K-->V
1T7RA	67 - 71	70	I-->D
1T7RA	67 - 71	70	I-->E
1T7RA	67 - 71	70	I-->G
1T7RA	67 - 71	70	I-->K
1T7RA	67 - 71	70	I-->L
1T7RA	67 - 71	70	I-->Q
1T7RA	67 - 71	70	I-->R
1T7RA	67 - 71	70	I-->S
1T7RA	67 - 71	70	I-->T
1T7RA	67 - 71	70	I-->V
1T7RA	67 - 71	71	L-->A
1T7RA	67 - 71	71	L-->D
1T7RA	67 - 71	71	L-->E
1T7RA	67 - 71	71	L-->T
1T7RA	67 - 71	71	L-->V
1K7JA	83 - 88	85	A-->K
1K7JA	83 - 88	85	A-->V
1K7JA	83 - 88	88	L-->I
1F8EA	105 - 109	105	A-->C
1F8EA	105 - 109	105	A-->D
1F8EA	105 - 109	105	A-->E
1F8EA	105 - 109	105	A-->G
1F8EA	105 - 109	105	A-->H
1F8EA	105 - 109	105	A-->I

1F8EA	105 - 109	105	A-->K
1F8EA	105 - 109	105	A-->L
1F8EA	105 - 109	105	A-->Q
1F8EA	105 - 109	105	A-->S
1F8EA	105 - 109	105	A-->T
1F8EA	105 - 109	105	A-->V
1F8EA	105 - 109	105	A-->W
1F8EA	105 - 109	106	V-->A
1F8EA	105 - 109	106	V-->D
1F8EA	105 - 109	106	V-->E
1F8EA	105 - 109	106	V-->F
1F8EA	105 - 109	106	V-->G
1F8EA	105 - 109	106	V-->K
1F8EA	105 - 109	106	V-->P
1F8EA	105 - 109	106	V-->R
1F8EA	105 - 109	106	V-->S
1F8EA	105 - 109	107	R-->A
1F8EA	105 - 109	107	R-->C
1F8EA	105 - 109	107	R-->D
1F8EA	105 - 109	107	R-->E
1F8EA	105 - 109	107	R-->F
1F8EA	105 - 109	107	R-->H
1F8EA	105 - 109	107	R-->I
1F8EA	105 - 109	107	R-->K
1F8EA	105 - 109	107	R-->L
1F8EA	105 - 109	107	R-->P
1F8EA	105 - 109	107	R-->S

1F8EA	105	109	107	R-->T
1F8EA	105	109	107	R-->V
1F8EA	105	109	107	R-->Y
1F8EA	105	109	108	I-->A
1F8EA	105	109	108	I-->D
1F8EA	105	109	108	I-->E
1F8EA	105	109	108	I-->F
1F8EA	105	109	108	I-->K
1F8EA	105	109	108	I-->P
1F8EA	105	109	108	I-->R
1F8EA	105	109	108	I-->T
1F8EA	105	109	108	I-->Y
1F8EA	105	109	109	G-->A
1F8EA	105	109	109	G-->D
1F8EA	105	109	109	G-->H
1F8EA	105	109	109	G-->K
1F8EA	105	109	109	G-->P
1F8EA	105	109	109	G-->R
1F8EA	105	109	109	G-->T
1F8EA	105	109	109	G-->V
1UWWB	130	135	133	L-->V
1UWWB	130	135	134	E-->L
2QFAA	15	20	16	D-->L
2QFAA	15	20	16	D-->P
2QFAA	15	20	17	H-->G
2QFAA	15	20	20	S-->N
2QFAA	35	40	36	E-->L

2QFAA	35 - 40	38	M-->T
1TZPB	132 - 136	132	S-->D
1TZPB	132 - 136	132	S-->E
1TZPB	132 - 136	132	S-->G
1TZPB	132 - 136	132	S-->H
1TZPB	132 - 136	132	S-->I
1TZPB	132 - 136	132	S-->K
1TZPB	132 - 136	132	S-->N
1TZPB	132 - 136	132	S-->T
1TZPB	132 - 136	132	S-->V
1TZPB	132 - 136	133	A-->C
1TZPB	132 - 136	133	A-->E
1TZPB	132 - 136	133	A-->G
1TZPB	132 - 136	133	A-->K
1TZPB	132 - 136	133	A-->L
1TZPB	132 - 136	133	A-->N
1TZPB	132 - 136	133	A-->P
1TZPB	132 - 136	133	A-->S
1TZPB	132 - 136	133	A-->T
1TZPB	132 - 136	133	A-->V
1TZPB	132 - 136	134	Q-->A
1TZPB	132 - 136	134	Q-->C
1TZPB	132 - 136	134	Q-->D
1TZPB	132 - 136	134	Q-->E
1TZPB	132 - 136	134	Q-->L
1TZPB	132 - 136	134	Q-->M
1TZPB	132 - 136	134	Q-->N

1TZPB	132 - 136	134	Q-->R
1TZPB	132 - 136	134	Q-->V
1TZPB	132 - 136	135	L-->A
1TZPB	132 - 136	135	L-->D
1TZPB	132 - 136	135	L-->F
1TZPB	132 - 136	135	L-->G
1TZPB	132 - 136	135	L-->I
1TZPB	132 - 136	135	L-->N
1TZPB	132 - 136	135	L-->S
1TZPB	132 - 136	135	L-->T
1TZPB	132 - 136	135	L-->V
1TZPB	132 - 136	136	L-->G
1TZPB	132 - 136	136	L-->I
1TZPB	132 - 136	136	L-->N
1TZPB	132 - 136	136	L-->R
1TZPB	132 - 136	136	L-->S
1TZPB	132 - 136	136	L-->W
1TZPB	195 - 199	196	W-->E
1TZPB	195 - 199	196	W-->G
1TZPB	195 - 199	196	W-->H
1TZPB	195 - 199	196	W-->L
1TZPB	195 - 199	196	W-->R
1TZPB	195 - 199	197	L-->I
1TZPB	195 - 199	198	R-->I
1TZPB	195 - 199	199	K-->I
1TZPB	195 - 199	199	K-->Y
1TZPB	237 - 241	237	A-->C

1TZPB	237	- 241	237	A-->F
1TZPB	237	- 241	237	A-->G
1TZPB	237	- 241	237	A-->S
1TZPB	237	- 241	237	A-->V
1TZPB	237	- 241	237	A-->Y
1TZPB	237	- 241	238	E-->G
1TZPB	237	- 241	238	E-->I
1TZPB	237	- 241	238	E-->K
1TZPB	237	- 241	238	E-->L
1TZPB	237	- 241	238	E-->N
1TZPB	237	- 241	238	E-->P
1TZPB	237	- 241	238	E-->S
1TZPB	237	- 241	238	E-->V
1TZPB	237	- 241	239	L-->A
1TZPB	237	- 241	239	L-->G
1TZPB	237	- 241	239	L-->H
1TZPB	237	- 241	239	L-->I
1TZPB	237	- 241	239	L-->R
1TZPB	237	- 241	240	Q-->A
1TZPB	237	- 241	240	Q-->E
1TZPB	237	- 241	240	Q-->G
1TZPB	237	- 241	240	Q-->I
1TZPB	237	- 241	240	Q-->K
1TZPB	237	- 241	240	Q-->P
1TZPB	237	- 241	240	Q-->T
1TZPB	237	- 241	240	Q-->V
1TZPB	237	- 241	241	S-->A

1TZPB	237	241	241	S-->D
1TZPB	237	241	241	S-->F
1TZPB	237	241	241	S-->G
1TZPB	237	241	241	S-->H
1TZPB	237	241	241	S-->I
1TZPB	237	241	241	S-->L
1TZPB	237	241	241	S-->Q
1TZPB	237	241	241	S-->R
1SVFC	176	180	176	V-->A
1SVFC	176	180	176	V-->D
1SVFC	176	180	176	V-->F
1SVFC	176	180	176	V-->G
1SVFC	176	180	176	V-->K
1SVFC	176	180	176	V-->L
1SVFC	176	180	176	V-->Q
1SVFC	176	180	176	V-->T
1SVFC	176	180	177	S-->A
1SVFC	176	180	177	S-->F
1SVFC	176	180	177	S-->K
1SVFC	176	180	177	S-->N
1SVFC	176	180	177	S-->P
1SVFC	176	180	177	S-->T
1SVFC	176	180	177	S-->V
1SVFC	176	180	178	P-->D
1SVFC	176	180	178	P-->G
1SVFC	176	180	178	P-->L
1SVFC	176	180	178	P-->Q

1SVFC	176 - 180	178	P-->S
1SVFC	176 - 180	178	P-->V
1SVFC	176 - 180	178	P-->Y
1SVFC	176 - 180	179	A-->D
1SVFC	176 - 180	179	A-->K
1SVFC	176 - 180	179	A-->L
1SVFC	176 - 180	179	A-->M
1SVFC	176 - 180	179	A-->P
1SVFC	176 - 180	179	A-->R
1SVFC	176 - 180	179	A-->T
1SVFC	176 - 180	179	A-->V
1SVFC	176 - 180	180	I-->A
1SVFC	176 - 180	180	I-->D
1SVFC	176 - 180	180	I-->E
1SVFC	176 - 180	180	I-->F
1SVFC	176 - 180	180	I-->G
1SVFC	176 - 180	180	I-->M
1SVFC	176 - 180	180	I-->N
1SVFC	176 - 180	180	I-->P
1SVFC	176 - 180	180	I-->S
1SVFC	176 - 180	180	I-->V
1SVFC	176 - 180	180	I-->W
3B9GB	86 - 91	91	D-->G
2BU3A	54 - 58	54	F-->A
2BU3A	54 - 58	54	F-->D
2BU3A	54 - 58	54	F-->G
2BU3A	54 - 58	54	F-->H

2BU3A	54 - 58	54	F-->K
2BU3A	54 - 58	54	F-->P
2BU3A	54 - 58	54	F-->Q
2BU3A	54 - 58	54	F-->T
2BU3A	54 - 58	54	F-->V
2BU3A	54 - 58	55	F-->A
2BU3A	54 - 58	55	F-->D
2BU3A	54 - 58	55	F-->E
2BU3A	54 - 58	55	F-->H
2BU3A	54 - 58	55	F-->K
2BU3A	54 - 58	55	F-->M
2BU3A	54 - 58	55	F-->N
2BU3A	54 - 58	55	F-->Q
2BU3A	54 - 58	55	F-->S
2BU3A	54 - 58	55	F-->T
2BU3A	54 - 58	56	P-->A
2BU3A	54 - 58	56	P-->G
2BU3A	54 - 58	56	P-->N
2BU3A	54 - 58	56	P-->T
2BU3A	54 - 58	56	P-->V
2BU3A	54 - 58	57	L-->A
2BU3A	54 - 58	57	L-->I
2BU3A	54 - 58	57	L-->M
2BU3A	54 - 58	57	L-->S
2BU3A	54 - 58	57	L-->T
2BU3A	54 - 58	57	L-->Y
2BU3A	54 - 58	58	S-->D

2BU3A	54 - 58	58	S-->E	
2BU3A	54 - 58	58	S-->K	
2BU3A	54 - 58	58	S-->P	
2BU3A	54 - 58	58	S-->T	
2BU3A	214 - 220	218	W-->A	
2QNTA	79 - 83	79	V-->A	
2QNTA	79 - 83	79	V-->F	
2QNTA	79 - 83	79	V-->I	
2QNTA	79 - 83	79	V-->K	
2QNTA	79 - 83	79	V-->L	
2QNTA	79 - 83	79	V-->P	
2QNTA	79 - 83	80	D-->C	
2QNTA	79 - 83	80	D-->G	
2QNTA	79 - 83	80	D-->H	
2QNTA	79 - 83	80	D-->L	
2QNTA	79 - 83	80	D-->N	
2QNTA	79 - 83	80	D-->P	
2QNTA	79 - 83	80	D-->S	
2QNTA	79 - 83	80	D-->V	
2QNTA	79 - 83	81	A-->F	
2QNTA	79 - 83	81	A-->G	
2QNTA	79 - 83	81	A-->N	
2QNTA	79 - 83	81	A-->P	
2QNTA	79 - 83	81	A-->S	
2QNTA	79 - 83	81	A-->T	
2QNTA	79 - 83	81	A-->V	
2QNTA	79 - 83	82	A-->D	

2QNTA	79 - 83	82	A-->E	
2QNTA	79 - 83	82	A-->G	
2QNTA	79 - 83	82	A-->I	
2QNTA	79 - 83	82	A-->L	
2QNTA	79 - 83	82	A-->Q	
2QNTA	79 - 83	82	A-->T	
2QNTA	79 - 83	82	A-->V	
2QNTA	79 - 83	83	F-->A	
2QNTA	79 - 83	83	F-->D	
2QNTA	79 - 83	83	F-->E	
2QNTA	79 - 83	83	F-->I	
2QNTA	79 - 83	83	F-->K	
2QNTA	79 - 83	83	F-->L	
2QNTA	79 - 83	83	F-->S	
2QNTA	79 - 83	83	F-->T	
2QNTA	79 - 83	83	F-->Y	
2V0UA	432 - 438	434	F-->Q	
2FSQA	68 - 72	68	P-->F	
2FSQA	68 - 72	68	P-->R	
2FSQA	68 - 72	68	P-->Y	
2FSQA	68 - 72	69	E-->A	
2FSQA	68 - 72	69	E-->G	
2FSQA	68 - 72	69	E-->K	
2FSQA	68 - 72	69	E-->L	
2FSQA	68 - 72	69	E-->N	
2FSQA	68 - 72	69	E-->P	
2FSQA	68 - 72	70	A-->G	

2FSQA	68	-	72	70	A-->K
2FSQA	68	-	72	70	A-->P
2FSQA	68	-	72	70	A-->S
2FSQA	68	-	72	71	S-->D
2FSQA	68	-	72	71	S-->G
2FSQA	68	-	72	71	S-->K
2FSQA	68	-	72	71	S-->L
2FSQA	68	-	72	71	S-->R
2FSQA	68	-	72	71	S-->T
2FSQA	68	-	72	72	Q-->A
2FSQA	68	-	72	72	Q-->D
2FSQA	68	-	72	72	Q-->G
2FSQA	68	-	72	72	Q-->P
2FSQA	68	-	72	72	Q-->R
2FSQA	68	-	72	72	Q-->S
2FSQA	68	-	72	72	Q-->V
2VPTA	163	-	169	166	K-->D
1VJUB	270	-	276	276	S-->P
1QOPB	136	-	141	141	R-->G
2AXIA	81	-	86	82	L-->H
2AXIA	81	-	86	84	D-->N
2AXIA	81	-	86	86	F-->I
2AXIA	81	-	86	86	F-->K
2AXIA	81	-	86	86	F-->V
2RFFA	88	-	93	92	D-->R
1LLFA	85	-	90	88	Q-->E
1LLFA	159	-	163	159	A-->E

1LLFA	159 - 163	159	A-->T
1LLFA	159 - 163	160	S-->G
1LLFA	159 - 163	160	S-->I
1LLFA	159 - 163	161	W-->A
1LLFA	159 - 163	161	W-->D
1LLFA	159 - 163	161	W-->G
1LLFA	159 - 163	161	W-->K
1LLFA	159 - 163	161	W-->P
1LLFA	159 - 163	161	W-->T
1LLFA	159 - 163	161	W-->V
1LLFA	159 - 163	161	W-->Y
1LLFA	159 - 163	162	G-->Q
1LLFA	159 - 163	163	F-->C
1LLFA	159 - 163	163	F-->G
1LLFA	159 - 163	163	F-->H
1LLFA	159 - 163	163	F-->L
1LLFA	159 - 163	163	F-->P
1LLFA	159 - 163	163	F-->Q
1LLFA	159 - 163	163	F-->R
1LLFA	159 - 163	163	F-->Y
1LLFA	167 - 172	169	I-->L
1LLFA	167 - 172	170	K-->H
1LLFA	167 - 172	170	K-->Q
1LLFA	167 - 172	171	A-->W
1LLFA	319 - 324	319	M-->T
1LLFA	319 - 324	323	V-->R
1K0MB	53 - 58	53	E-->V

1K0MB	53 - 58	54	T-->E
1K0MB	53 - 58	54	T-->P
1K0MB	53 - 58	56	Q-->R
1K0MB	53 - 58	58	L-->I
2ODKA	6 - 11	6	V-->A
2ODKA	6 - 11	7	Q-->F
2JEPB	231 - 235	231	I-->D
2JEPB	231 - 235	231	I-->G
2JEPB	231 - 235	231	I-->K
2JEPB	231 - 235	231	I-->L
2JEPB	231 - 235	231	I-->P
2JEPB	231 - 235	231	I-->Q
2JEPB	231 - 235	231	I-->R
2JEPB	231 - 235	231	I-->S
2JEPB	231 - 235	231	I-->T
2JEPB	231 - 235	231	I-->V
2JEPB	231 - 235	232	D-->A
2JEPB	231 - 235	232	D-->E
2JEPB	231 - 235	232	D-->F
2JEPB	231 - 235	232	D-->H
2JEPB	231 - 235	232	D-->I
2JEPB	231 - 235	232	D-->S
2JEPB	231 - 235	232	D-->T
2JEPB	231 - 235	232	D-->V
2JEPB	231 - 235	233	Y-->D
2JEPB	231 - 235	233	Y-->E
2JEPB	231 - 235	233	Y-->G

2JEPB	231 - 235	233	Y-->N
2JEPB	231 - 235	233	Y-->S
2JEPB	231 - 235	233	Y-->V
2JEPB	231 - 235	234	T-->A
2JEPB	231 - 235	234	T-->E
2JEPB	231 - 235	234	T-->F
2JEPB	231 - 235	234	T-->G
2JEPB	231 - 235	234	T-->I
2JEPB	231 - 235	234	T-->L
2JEPB	231 - 235	234	T-->N
2JEPB	231 - 235	234	T-->S
2JEPB	231 - 235	234	T-->V
2JEPB	231 - 235	235	V-->T
2JEPB	231 - 235	235	V-->Y
1WB4A	938 - 942	938	P-->A
1WB4A	938 - 942	938	P-->C
1WB4A	938 - 942	938	P-->E
1WB4A	938 - 942	938	P-->G
1WB4A	938 - 942	938	P-->I
1WB4A	938 - 942	938	P-->L
1WB4A	938 - 942	938	P-->N
1WB4A	938 - 942	938	P-->R
1WB4A	938 - 942	938	P-->V
1WB4A	938 - 942	939	Q-->A
1WB4A	938 - 942	939	Q-->D
1WB4A	938 - 942	939	Q-->E
1WB4A	938 - 942	939	Q-->G

1WB4A	938 - 942	939	Q-->K	
1WB4A	938 - 942	939	Q-->L	
1WB4A	938 - 942	939	Q-->N	
1WB4A	938 - 942	939	Q-->P	
1WB4A	938 - 942	939	Q-->R	
1WB4A	938 - 942	939	Q-->S	
1WB4A	938 - 942	939	Q-->T	
1WB4A	938 - 942	939	Q-->V	
1WB4A	938 - 942	939	Q-->W	
1WB4A	938 - 942	939	Q-->Y	
1WB4A	938 - 942	940	G-->A	
1WB4A	938 - 942	940	G-->L	
1WB4A	938 - 942	940	G-->P	
1WB4A	938 - 942	940	G-->R	
1WB4A	938 - 942	941	I-->A	
1WB4A	938 - 942	941	I-->D	
1WB4A	938 - 942	941	I-->E	
1WB4A	938 - 942	941	I-->K	
1WB4A	938 - 942	941	I-->L	
1WB4A	938 - 942	941	I-->N	
1WB4A	938 - 942	941	I-->P	
1WB4A	938 - 942	941	I-->Q	
1WB4A	938 - 942	941	I-->T	
1WB4A	938 - 942	941	I-->V	
1WB4A	938 - 942	941	I-->Y	
1WB4A	938 - 942	942	A-->I	
1WB4A	938 - 942	942	A-->P	

1WB4A	938 - 942	942	A-->S
1WB4A	938 - 942	942	A-->T
1H2WA	511 - 516	511	G-->I
1H2WA	511 - 516	516	K-->D
1H2WA	511 - 516	516	K-->M
1N8VA	13 - 17	13	L-->D
1N8VA	13 - 17	13	L-->E
1N8VA	13 - 17	13	L-->G
1N8VA	13 - 17	13	L-->K
1N8VA	13 - 17	13	L-->Q
1N8VA	13 - 17	13	L-->Y
1N8VA	13 - 17	14	D-->E
1N8VA	13 - 17	14	D-->G
1N8VA	13 - 17	14	D-->K
1N8VA	13 - 17	14	D-->P
1N8VA	13 - 17	14	D-->Q
1N8VA	13 - 17	14	D-->R
1N8VA	13 - 17	14	D-->T
1N8VA	13 - 17	14	D-->V
1N8VA	13 - 17	15	E-->A
1N8VA	13 - 17	15	E-->G
1N8VA	13 - 17	15	E-->I
1N8VA	13 - 17	15	E-->L
1N8VA	13 - 17	15	E-->R
1N8VA	13 - 17	15	E-->S
1N8VA	13 - 17	15	E-->V
1N8VA	13 - 17	16	I-->D

1N8VA	13 - 17	16	I-->E
1N8VA	13 - 17	16	I-->F
1N8VA	13 - 17	16	I-->K
1N8VA	13 - 17	16	I-->N
1N8VA	13 - 17	16	I-->P
1N8VA	13 - 17	16	I-->Q
1N8VA	13 - 17	16	I-->R
1N8VA	13 - 17	16	I-->S
1N8VA	13 - 17	16	I-->T
1N8VA	13 - 17	17	L-->A
1N8VA	13 - 17	17	L-->D
1N8VA	13 - 17	17	L-->F
1N8VA	13 - 17	17	L-->G
1N8VA	13 - 17	17	L-->H
1N8VA	13 - 17	17	L-->P
1N8VA	13 - 17	17	L-->V
1N8VA	13 - 17	17	L-->W
1K55C	56 - 60	56	L-->A
1K55C	56 - 60	56	L-->C
1K55C	56 - 60	56	L-->F
1K55C	56 - 60	56	L-->G
1K55C	56 - 60	56	L-->H
1K55C	56 - 60	56	L-->K
1K55C	56 - 60	56	L-->Q
1K55C	56 - 60	56	L-->S
1K55C	56 - 60	56	L-->T
1K55C	56 - 60	57	A-->E

1K55C	56 - 60	57	A-->F
1K55C	56 - 60	57	A-->G
1K55C	56 - 60	57	A-->N
1K55C	56 - 60	57	A-->T
1K55C	56 - 60	57	A-->V
1K55C	56 - 60	58	R-->A
1K55C	56 - 60	58	R-->C
1K55C	56 - 60	58	R-->D
1K55C	56 - 60	58	R-->F
1K55C	56 - 60	58	R-->K
1K55C	56 - 60	58	R-->L
1K55C	56 - 60	58	R-->N
1K55C	56 - 60	58	R-->Q
1K55C	56 - 60	58	R-->S
1K55C	56 - 60	58	R-->T
1K55C	56 - 60	58	R-->W
1K55C	56 - 60	58	R-->Y
1K55C	56 - 60	59	A-->C
1K55C	56 - 60	59	A-->F
1K55C	56 - 60	59	A-->G
1K55C	56 - 60	59	A-->I
1K55C	56 - 60	59	A-->P
1K55C	56 - 60	59	A-->T
1K55C	56 - 60	59	A-->V
1K55C	56 - 60	59	A-->W
1K55C	56 - 60	59	A-->Y
1K55C	56 - 60	60	S-->E

1K55C	56 - 60	60	S-->F
1K55C	56 - 60	60	S-->I
1K55C	56 - 60	60	S-->K
1K55C	56 - 60	60	S-->P
1K55C	56 - 60	60	S-->Q
1K55C	56 - 60	60	S-->V
1K55C	108 - 113	110	G-->F
1K55C	108 - 113	113	Q-->E
1K55C	108 - 113	113	Q-->I
1RYQA	43 - 48	43	E-->I
1RYQA	43 - 48	45	A-->P
1RYQA	43 - 48	46	K-->G
1RYQA	43 - 48	46	K-->I
1RYQA	43 - 48	48	I-->D
1S9UA	88 - 92	88	G-->Y
1S9UA	88 - 92	89	S-->A
1S9UA	88 - 92	89	S-->D
1S9UA	88 - 92	89	S-->E
1S9UA	88 - 92	89	S-->K
1S9UA	88 - 92	89	S-->L
1S9UA	88 - 92	89	S-->W
1S9UA	88 - 92	90	V-->D
1S9UA	88 - 92	90	V-->G
1S9UA	88 - 92	90	V-->L
1S9UA	88 - 92	90	V-->Q
1S9UA	88 - 92	90	V-->R
1S9UA	88 - 92	91	W-->A

1S9UA	88	92	91	W-->D
1S9UA	88	92	91	W-->E
1S9UA	88	92	91	W-->F
1S9UA	88	92	91	W-->G
1S9UA	88	92	91	W-->I
1S9UA	88	92	91	W-->K
1S9UA	88	92	91	W-->L
1S9UA	88	92	91	W-->M
1S9UA	88	92	91	W-->N
1S9UA	88	92	91	W-->P
1S9UA	88	92	91	W-->Q
1S9UA	88	92	91	W-->R
1S9UA	88	92	91	W-->S
1S9UA	88	92	91	W-->T
1S9UA	88	92	91	W-->V
1S9UA	88	92	91	W-->Y
1S9UA	88	92	92	L-->A
1S9UA	88	92	92	L-->E
1S9UA	88	92	92	L-->G
1S9UA	88	92	92	L-->I
1S9UA	88	92	92	L-->N
1S9UA	88	92	92	L-->S
1S9UA	88	92	92	L-->T
1S9UA	88	92	92	L-->V
1HX0A	58	62	59	W-->T
1HX0A	58	62	59	W-->V
1HX0A	58	62	62	Y-->D

1HX0A	154	159	155	Y-->S
1HX0A	154	159	156	Q-->G
1HX0A	274	279	279	N-->S
3B79A	22	26	22	P-->D
3B79A	22	26	22	P-->E
3B79A	22	26	22	P-->G
3B79A	22	26	22	P-->I
3B79A	22	26	22	P-->L
3B79A	22	26	22	P-->Q
3B79A	22	26	22	P-->V
3B79A	22	26	23	E-->A
3B79A	22	26	23	E-->D
3B79A	22	26	23	E-->G
3B79A	22	26	23	E-->H
3B79A	22	26	23	E-->I
3B79A	22	26	23	E-->K
3B79A	22	26	23	E-->L
3B79A	22	26	23	E-->N
3B79A	22	26	23	E-->S
3B79A	22	26	23	E-->T
3B79A	22	26	23	E-->V
3B79A	22	26	23	E-->W
3B79A	22	26	23	E-->Y
3B79A	22	26	24	A-->D
3B79A	22	26	24	A-->E
3B79A	22	26	24	A-->G
3B79A	22	26	24	A-->H

3B79A	22 - 26	24	A-->L
3B79A	22 - 26	24	A-->N
3B79A	22 - 26	24	A-->R
3B79A	22 - 26	24	A-->S
3B79A	22 - 26	24	A-->T
3B79A	22 - 26	24	A-->Y
3B79A	22 - 26	25	L-->D
3B79A	22 - 26	25	L-->F
3B79A	22 - 26	25	L-->G
3B79A	22 - 26	25	L-->H
3B79A	22 - 26	25	L-->I
3B79A	22 - 26	25	L-->N
3B79A	22 - 26	25	L-->P
3B79A	22 - 26	25	L-->Q
3B79A	22 - 26	25	L-->S
3B79A	22 - 26	25	L-->V
3B79A	22 - 26	25	L-->Y
3B79A	22 - 26	26	V-->E
3B79A	22 - 26	26	V-->F
3B79A	22 - 26	26	V-->I
3B79A	22 - 26	26	V-->M
3B79A	22 - 26	26	V-->P
3B79A	22 - 26	26	V-->Q
3B79A	22 - 26	26	V-->R
3B79A	22 - 26	26	V-->S
1WBEA	31 - 35	31	P-->E
1WBEA	31 - 35	31	P-->F

1WBEA	31 - 35	31	P-->I
1WBEA	31 - 35	31	P-->M
1WBEA	31 - 35	31	P-->N
1WBEA	31 - 35	31	P-->Q
1WBEA	31 - 35	31	P-->V
1WBEA	31 - 35	32	P-->H
1WBEA	31 - 35	32	P-->R
1WBEA	31 - 35	32	P-->V
1WBEA	31 - 35	33	F-->E
1WBEA	31 - 35	33	F-->G
1WBEA	31 - 35	33	F-->H
1WBEA	31 - 35	33	F-->K
1WBEA	31 - 35	33	F-->Y
1WBEA	31 - 35	34	F-->D
1WBEA	31 - 35	34	F-->G
1WBEA	31 - 35	34	F-->I
1WBEA	31 - 35	34	F-->P
1WBEA	31 - 35	34	F-->S
1WBEA	31 - 35	34	F-->Y
1WBEA	31 - 35	35	D-->A
1WBEA	31 - 35	35	D-->E
1WBEA	31 - 35	35	D-->G
1WBEA	31 - 35	35	D-->N
1WBEA	31 - 35	35	D-->P
1WBEA	31 - 35	35	D-->S
2P8GA	89 - 93	90	W-->L
2P8GA	89 - 93	90	W-->Q

2P8GA	89 - 93	90	W-->R
2P8GA	89 - 93	90	W-->V
2P8GA	89 - 93	91	V-->I
2P8GA	89 - 93	92	H-->I
2P8GA	110 - 115	112	S-->G
2P8GA	110 - 115	112	S-->L
2P8GA	153 - 158	155	D-->V
2P8GA	153 - 158	157	R-->G
2HD9A	105 - 110	106	P-->G
2HD9A	105 - 110	107	L-->P
2HD9A	105 - 110	108	I-->H
1UZKA	1559 - 1563	1560	A-->C
1UZKA	1559 - 1563	1560	A-->G
1UZKA	1559 - 1563	1560	A-->K
1UZKA	1559 - 1563	1560	A-->L
1UZKA	1559 - 1563	1560	A-->Q
1UZKA	1559 - 1563	1562	C-->F
1UZKA	1559 - 1563	1562	C-->G
1UZKA	1559 - 1563	1562	C-->N
1UZKA	1559 - 1563	1562	C-->P
1UZKA	1559 - 1563	1563	C-->K
1UZKA	1559 - 1563	1563	C-->L
1UZKA	1559 - 1563	1563	C-->V
1UZKA	1583 - 1588	1584	E-->T
1UZKA	1583 - 1588	1585	Y-->H
1UZKA	1583 - 1588	1585	Y-->I
1UZKA	1583 - 1588	1585	Y-->L

1UZKA	1583	- 1588	1586	K-->T
1UZKA	1583	- 1588	1587	I-->M
1UZKA	1583	- 1588	1588	L-->G
2EIYB	27	- 31	27	H-->Y
2EIYB	27	- 31	28	A-->L
2EIYB	27	- 31	28	A-->S
2EIYB	27	- 31	29	L-->I
2EIYB	27	- 31	29	L-->T
2EIYB	27	- 31	30	H-->C
2EIYB	27	- 31	30	H-->D
2EIYB	27	- 31	30	H-->M
2EIYB	27	- 31	30	H-->R
2EIYB	27	- 31	31	Y-->I
2EIYB	27	- 31	31	Y-->M
2EIYB	27	- 31	31	Y-->R
2EIYB	132	- 137	134	A-->P
2EIYB	132	- 137	135	V-->R
2EIYB	132	- 137	136	R-->S
2EIYB	242	- 246	242	R-->I
2EIYB	242	- 246	242	R-->L
2EIYB	242	- 246	243	D-->I
2EIYB	242	- 246	243	D-->Q
2EIYB	242	- 246	243	D-->R
2EIYB	242	- 246	243	D-->V
2EIYB	242	- 246	244	Q-->A
2EIYB	242	- 246	244	Q-->D
2EIYB	242	- 246	244	Q-->G

2EIYB	242	-	246	244	Q-->I
2EIYB	242	-	246	244	Q-->K
2EIYB	242	-	246	244	Q-->N
2EIYB	242	-	246	244	Q-->S
2EIYB	242	-	246	244	Q-->T
2EIYB	242	-	246	244	Q-->W
2EIYB	242	-	246	245	L-->N
2EIYB	242	-	246	245	L-->S
2EIYB	242	-	246	246	Y-->I
2EIYB	242	-	246	246	Y-->K
2EIYB	242	-	246	246	Y-->P
2EIYB	242	-	246	246	Y-->V
2EIYB	242	-	246	246	Y-->W
2EIYB	296	-	301	297	E-->G
1KUFA	18	-	23	20	M-->L
1VKKA	54	-	60	55	E-->I
2OSSA	70	-	75	70	V-->R
2OSSA	70	-	75	74	L-->M
2OSSA	70	-	75	75	W-->S
1SJWA	34	-	39	38	E-->K
1SJWA	34	-	39	39	H-->T
1SJWA	34	-	39	39	H-->V
2JDAB	26	-	30	26	P-->D
2JDAB	26	-	30	26	P-->E
2JDAB	26	-	30	26	P-->F
2JDAB	26	-	30	26	P-->G
2JDAB	26	-	30	26	P-->I

2JDAB	26 - 30	26	P-->K
2JDAB	26 - 30	26	P-->L
2JDAB	26 - 30	26	P-->R
2JDAB	26 - 30	26	P-->S
2JDAB	26 - 30	26	P-->Y
2JDAB	26 - 30	27	A-->D
2JDAB	26 - 30	27	A-->R
2JDAB	26 - 30	27	A-->V
2JDAB	26 - 30	28	N-->A
2JDAB	26 - 30	28	N-->E
2JDAB	26 - 30	28	N-->G
2JDAB	26 - 30	28	N-->I
2JDAB	26 - 30	28	N-->K
2JDAB	26 - 30	28	N-->M
2JDAB	26 - 30	28	N-->R
2JDAB	26 - 30	28	N-->S
2JDAB	26 - 30	28	N-->T
2JDAB	26 - 30	28	N-->V
2JDAB	26 - 30	29	I-->A
2JDAB	26 - 30	29	I-->D
2JDAB	26 - 30	29	I-->G
2JDAB	26 - 30	29	I-->L
2JDAB	26 - 30	29	I-->P
2JDAB	26 - 30	29	I-->Q
2JDAB	26 - 30	29	I-->R
2JDAB	26 - 30	29	I-->S
2JDAB	26 - 30	29	I-->T

2JDAB	26 - 30	29	I-->V
2JDAB	26 - 30	30	A-->G
2JDAB	26 - 30	30	A-->I
2JDAB	26 - 30	30	A-->K
2JDAB	26 - 30	30	A-->L
2JDAB	26 - 30	30	A-->M
2JDAB	26 - 30	30	A-->P
2JDAB	26 - 30	30	A-->Q
2JDAB	26 - 30	30	A-->S
2JDAB	26 - 30	30	A-->T
2JDAB	26 - 30	30	A-->V
2IYVA	173 - 178	173	E-->L
2IYVA	173 - 178	174	A-->K
2IYVA	173 - 178	176	T-->E
2IYVA	173 - 178	178	E-->I
2QIKA	116 - 120	116	W-->F
2QIKA	116 - 120	116	W-->N
2QIKA	116 - 120	116	W-->P
2QIKA	116 - 120	117	K-->S
2QIKA	116 - 120	120	Q-->N
2QIKA	141 - 146	142	R-->D
2QIKA	141 - 146	142	R-->P
2QE8A	233 - 237	233	D-->E
2QE8A	233 - 237	233	D-->G
2QE8A	233 - 237	233	D-->H
2QE8A	233 - 237	233	D-->I
2QE8A	233 - 237	233	D-->K

2QE8A	233 - 237	233	D-->L
2QE8A	233 - 237	233	D-->M
2QE8A	233 - 237	233	D-->N
2QE8A	233 - 237	233	D-->S
2QE8A	233 - 237	233	D-->T
2QE8A	233 - 237	233	D-->V
2QE8A	233 - 237	233	D-->Y
2QE8A	233 - 237	234	A-->C
2QE8A	233 - 237	234	A-->E
2QE8A	233 - 237	234	A-->F
2QE8A	233 - 237	234	A-->G
2QE8A	233 - 237	234	A-->I
2QE8A	233 - 237	234	A-->K
2QE8A	233 - 237	234	A-->L
2QE8A	233 - 237	234	A-->M
2QE8A	233 - 237	234	A-->N
2QE8A	233 - 237	234	A-->P
2QE8A	233 - 237	234	A-->S
2QE8A	233 - 237	234	A-->V
2QE8A	233 - 237	235	E-->A
2QE8A	233 - 237	235	E-->C
2QE8A	233 - 237	235	E-->G
2QE8A	233 - 237	235	E-->H
2QE8A	233 - 237	235	E-->K
2QE8A	233 - 237	235	E-->L
2QE8A	233 - 237	235	E-->M
2QE8A	233 - 237	235	E-->Q

2QE8A	233 - 237	235	E-->S
2QE8A	233 - 237	235	E-->T
2QE8A	233 - 237	235	E-->V
2QE8A	233 - 237	235	E-->W
2QE8A	233 - 237	236	L-->A
2QE8A	233 - 237	236	L-->D
2QE8A	233 - 237	236	L-->E
2QE8A	233 - 237	236	L-->I
2QE8A	233 - 237	236	L-->N
2QE8A	233 - 237	236	L-->Q
2QE8A	233 - 237	236	L-->S
2QE8A	233 - 237	236	L-->T
2QE8A	233 - 237	236	L-->V
2QE8A	233 - 237	236	L-->Y
2QE8A	233 - 237	237	G-->A
2QE8A	233 - 237	237	G-->C
2QE8A	233 - 237	237	G-->D
2QE8A	233 - 237	237	G-->E
2QE8A	233 - 237	237	G-->F
2QE8A	233 - 237	237	G-->L
2QE8A	233 - 237	237	G-->N
2QE8A	233 - 237	237	G-->Q
2QE8A	233 - 237	237	G-->R
2QE8A	233 - 237	237	G-->V
1VHUA	94 - 98	94	N-->D
1VHUA	94 - 98	94	N-->E
1VHUA	94 - 98	94	N-->G

1VHUA	94 - 98	94	N-->I
1VHUA	94 - 98	94	N-->K
1VHUA	94 - 98	94	N-->M
1VHUA	94 - 98	94	N-->P
1VHUA	94 - 98	94	N-->Q
1VHUA	94 - 98	94	N-->T
1VHUA	94 - 98	94	N-->V
1VHUA	94 - 98	95	L-->G
1VHUA	94 - 98	95	L-->K
1VHUA	94 - 98	95	L-->M
1VHUA	94 - 98	95	L-->P
1VHUA	94 - 98	95	L-->S
1VHUA	94 - 98	95	L-->V
1VHUA	94 - 98	96	E-->F
1VHUA	94 - 98	96	E-->K
1VHUA	94 - 98	96	E-->P
1VHUA	94 - 98	96	E-->Q
1VHUA	94 - 98	96	E-->T
1VHUA	94 - 98	96	E-->V
1VHUA	94 - 98	97	E-->A
1VHUA	94 - 98	97	E-->G
1VHUA	94 - 98	97	E-->K
1VHUA	94 - 98	97	E-->L
1VHUA	94 - 98	97	E-->P
1VHUA	94 - 98	97	E-->Q
1VHUA	94 - 98	97	E-->T
1VHUA	94 - 98	98	R-->A

1VHUA	94	98	98	R-->D
1VHUA	94	98	98	R-->F
1VHUA	94	98	98	R-->H
1VHUA	94	98	98	R-->K
1VHUA	94	98	98	R-->N
1VHUA	94	98	98	R-->P
1VHUA	94	98	98	R-->S
1VHUA	94	98	98	R-->V
1VHUA	94	98	98	R-->Y
2UV4A	262	267	262	V-->S
2UV4A	262	267	263	T-->E
2UV4A	262	267	264	K-->H
2UV4A	262	267	264	K-->L
2UV4A	262	267	267	Q-->P
1GPIA	64	70	65	K-->Y
1GPIA	78	82	78	Y-->A
1GPIA	78	82	78	Y-->D
1GPIA	78	82	78	Y-->E
1GPIA	78	82	78	Y-->G
1GPIA	78	82	78	Y-->I
1GPIA	78	82	78	Y-->L
1GPIA	78	82	78	Y-->P
1GPIA	78	82	78	Y-->Q
1GPIA	78	82	78	Y-->R
1GPIA	78	82	78	Y-->S
1GPIA	78	82	78	Y-->T
1GPIA	78	82	78	Y-->V

1GPIA	78 - 82	78	Y-->W
1GPIA	78 - 82	79	S-->D
1GPIA	78 - 82	79	S-->E
1GPIA	78 - 82	79	S-->G
1GPIA	78 - 82	79	S-->K
1GPIA	78 - 82	79	S-->Q
1GPIA	78 - 82	79	S-->T
1GPIA	78 - 82	79	S-->Y
1GPIA	78 - 82	80	G-->A
1GPIA	78 - 82	80	G-->L
1GPIA	78 - 82	80	G-->N
1GPIA	78 - 82	80	G-->P
1GPIA	78 - 82	80	G-->Q
1GPIA	78 - 82	80	G-->R
1GPIA	78 - 82	80	G-->T
1GPIA	78 - 82	80	G-->V
1GPIA	78 - 82	81	T-->D
1GPIA	78 - 82	81	T-->E
1GPIA	78 - 82	81	T-->G
1GPIA	78 - 82	81	T-->N
1GPIA	78 - 82	81	T-->P
1GPIA	78 - 82	81	T-->R
1GPIA	78 - 82	81	T-->V
1GPIA	78 - 82	82	Y-->A
1GPIA	78 - 82	82	Y-->C
1GPIA	78 - 82	82	Y-->G
1GPIA	78 - 82	82	Y-->I

1GPIA	78 - 82	82	Y-->K
1GPIA	78 - 82	82	Y-->L
1GPIA	78 - 82	82	Y-->P
1GPIA	78 - 82	82	Y-->R
1GPIA	78 - 82	82	Y-->S
1GPIA	78 - 82	82	Y-->T
1V9YA	22 - 27	23	F-->V
1V9YA	22 - 27	27	E-->Q
2Q4NA	21 - 26	22	P-->T
2Q4NA	21 - 26	26	L-->S
2RDQA	87 - 91	89	A-->R
2RDQA	87 - 91	89	A-->T
2RDQA	87 - 91	91	W-->L
2RDQA	87 - 91	91	W-->S
2RDQA	87 - 91	91	W-->V
2RDQA	87 - 91	91	W-->Y
3BLNA	109 - 114	111	V-->P
3BLNA	109 - 114	113	N-->I
3BLNA	109 - 114	113	N-->V
2PV1A	212 - 219	214	K-->L
2PV1A	241 - 246	243	F-->I
2PV1A	241 - 246	246	A-->K
1UCDA	11 - 16	12	P-->C
1UCDA	11 - 16	12	P-->V
1UCDA	11 - 16	13	A-->L
1UCDA	11 - 16	15	C-->G
1ZCEA	19 - 25	19	Q-->E

1ZCEA	100 - 105	103	V-->A
1ZCEA	100 - 105	103	V-->P
1ZCEA	100 - 105	104	K-->I
1ZCEA	100 - 105	105	A-->G
2D5WA	199 - 203	199	P-->A
2D5WA	199 - 203	199	P-->R
2D5WA	199 - 203	199	P-->T
2D5WA	199 - 203	199	P-->W
2D5WA	199 - 203	200	Q-->A
2D5WA	199 - 203	200	Q-->D
2D5WA	199 - 203	200	Q-->G
2D5WA	199 - 203	200	Q-->I
2D5WA	199 - 203	200	Q-->K
2D5WA	199 - 203	200	Q-->L
2D5WA	199 - 203	200	Q-->R
2D5WA	199 - 203	200	Q-->V
2D5WA	199 - 203	201	A-->C
2D5WA	199 - 203	201	A-->L
2D5WA	199 - 203	201	A-->P
2D5WA	199 - 203	201	A-->Q
2D5WA	199 - 203	201	A-->T
2D5WA	199 - 203	201	A-->V
2D5WA	199 - 203	202	L-->A
2D5WA	199 - 203	202	L-->M
2D5WA	199 - 203	202	L-->R
2D5WA	199 - 203	203	N-->A
2D5WA	199 - 203	203	N-->D

2D5WA	199 - 203	203	N-->E
2D5WA	199 - 203	203	N-->G
2D5WA	199 - 203	203	N-->H
2D5WA	199 - 203	203	N-->M
2D5WA	199 - 203	203	N-->P
2D5WA	199 - 203	203	N-->Q
2D5WA	199 - 203	203	N-->S
2D5WA	199 - 203	203	N-->T
2D5WA	315 - 320	315	Q-->A
2D5WA	315 - 320	315	Q-->S
2D5WA	315 - 320	315	Q-->V
2D5WA	315 - 320	317	V-->L
2D5WA	315 - 320	318	K-->Y
2D5WA	315 - 320	319	D-->L
2HXMA	193 - 197	193	S-->F
2HXMA	193 - 197	193	S-->H
2HXMA	193 - 197	193	S-->R
2HXMA	193 - 197	194	G-->A
2HXMA	193 - 197	195	W-->A
2HXMA	193 - 197	195	W-->D
2HXMA	193 - 197	195	W-->E
2HXMA	193 - 197	195	W-->F
2HXMA	193 - 197	195	W-->G
2HXMA	193 - 197	195	W-->H
2HXMA	193 - 197	195	W-->I
2HXMA	193 - 197	195	W-->K
2HXMA	193 - 197	195	W-->N

2HXMA	193	197	195	W-->P
2HXMA	193	197	195	W-->Q
2HXMA	193	197	195	W-->R
2HXMA	193	197	195	W-->T
2HXMA	193	197	195	W-->V
2HXMA	193	197	196	A-->G
2HXMA	193	197	196	A-->L
2HXMA	193	197	196	A-->Q
2HXMA	193	197	196	A-->V
2HXMA	193	197	196	A-->W
2HXMA	193	197	196	A-->Y
2HXMA	193	197	197	K-->A
2HXMA	193	197	197	K-->L
2HXMA	193	197	197	K-->S
2HXMA	193	197	197	K-->T
2HXMA	193	197	197	K-->V
2HXMA	193	197	197	K-->W
1YG9A	249	254	251	K-->S
1HYOA	59	63	59	Q-->E
1HYOA	59	63	59	Q-->I
1HYOA	59	63	59	Q-->V
1HYOA	59	63	60	H-->M
1HYOA	59	63	60	H-->N
1HYOA	59	63	60	H-->S
1HYOA	59	63	60	H-->V
1HYOA	59	63	62	F-->A
1HYOA	59	63	62	F-->K

1HYOA	59 - 63	63	D-->K
1HYOA	59 - 63	63	D-->L
1HYOA	59 - 63	63	D-->Q
1HYOA	68 - 73	72	G-->A
2IMQX	192 - 196	192	I-->A
2IMQX	192 - 196	192	I-->D
2IMQX	192 - 196	192	I-->E
2IMQX	192 - 196	192	I-->M
2IMQX	192 - 196	192	I-->Q
2IMQX	192 - 196	192	I-->V
2IMQX	192 - 196	193	D-->C
2IMQX	192 - 196	193	D-->E
2IMQX	192 - 196	193	D-->H
2IMQX	192 - 196	193	D-->L
2IMQX	192 - 196	193	D-->S
2IMQX	192 - 196	193	D-->T
2IMQX	192 - 196	194	G-->D
2IMQX	192 - 196	194	G-->H
2IMQX	192 - 196	195	L-->E
2IMQX	192 - 196	195	L-->F
2IMQX	192 - 196	195	L-->G
2IMQX	192 - 196	195	L-->K
2IMQX	192 - 196	195	L-->M
2IMQX	192 - 196	196	M-->A
2IMQX	192 - 196	196	M-->D
2IMQX	192 - 196	196	M-->E
2IMQX	192 - 196	196	M-->F

2IMQX	192 - 196	196	M-->I
2IMQX	192 - 196	196	M-->K
2IMQX	192 - 196	196	M-->L
2IMQX	192 - 196	196	M-->N
2IMQX	192 - 196	196	M-->P
2IMQX	192 - 196	196	M-->R
2IMQX	192 - 196	196	M-->S
2IMQX	192 - 196	196	M-->T
2IMQX	192 - 196	196	M-->V
2IMQX	192 - 196	196	M-->Y
2IMQX	201 - 206	203	K-->D
2IMQX	201 - 206	205	K-->G
1O8XA	127 - 131	127	A-->D
1O8XA	127 - 131	127	A-->E
1O8XA	127 - 131	127	A-->G
1O8XA	127 - 131	127	A-->H
1O8XA	127 - 131	127	A-->L
1O8XA	127 - 131	127	A-->P
1O8XA	127 - 131	127	A-->R
1O8XA	127 - 131	127	A-->S
1O8XA	127 - 131	127	A-->V
1O8XA	127 - 131	127	A-->W
1O8XA	127 - 131	127	A-->Y
1O8XA	127 - 131	128	R-->A
1O8XA	127 - 131	128	R-->C
1O8XA	127 - 131	128	R-->D
1O8XA	127 - 131	128	R-->E

1O8XA	127 - 131	128	R-->F
1O8XA	127 - 131	128	R-->G
1O8XA	127 - 131	128	R-->I
1O8XA	127 - 131	128	R-->K
1O8XA	127 - 131	128	R-->M
1O8XA	127 - 131	128	R-->N
1O8XA	127 - 131	128	R-->P
1O8XA	127 - 131	128	R-->S
1O8XA	127 - 131	128	R-->T
1O8XA	127 - 131	128	R-->V
1O8XA	127 - 131	128	R-->Y
1O8XA	127 - 131	129	A-->E
1O8XA	127 - 131	129	A-->F
1O8XA	127 - 131	129	A-->G
1O8XA	127 - 131	129	A-->H
1O8XA	127 - 131	129	A-->I
1O8XA	127 - 131	129	A-->K
1O8XA	127 - 131	129	A-->L
1O8XA	127 - 131	129	A-->N
1O8XA	127 - 131	129	A-->V
1O8XA	127 - 131	129	A-->Y
1O8XA	127 - 131	130	T-->A
1O8XA	127 - 131	130	T-->E
1O8XA	127 - 131	130	T-->K
1O8XA	127 - 131	130	T-->M
1O8XA	127 - 131	130	T-->R
1O8XA	127 - 131	130	T-->S

1O8XA	127	131	130	T-->Y
1O8XA	127	131	131	L-->A
1O8XA	127	131	131	L-->D
1O8XA	127	131	131	L-->E
1O8XA	127	131	131	L-->I
1O8XA	127	131	131	L-->N
1O8XA	127	131	131	L-->P
1O8XA	127	131	131	L-->V
2NLVB	79	84	80	A-->E
2NLVB	79	84	81	E-->F
2NLVB	79	84	81	E-->S
2NLVB	79	84	83	L-->K
2NLVB	79	84	84	V-->P
2NLVB	100	105	104	Q-->Y
2GSOA	58	62	58	A-->F
2GSOA	58	62	59	D-->R
2GSOA	58	62	59	D-->S
2GSOA	58	62	59	D-->T
2GSOA	58	62	60	M-->E
2GSOA	58	62	60	M-->G
2GSOA	58	62	60	M-->I
2GSOA	58	62	60	M-->L
2GSOA	58	62	60	M-->P
2GSOA	58	62	60	M-->R
2GSOA	58	62	60	M-->S
2GSOA	58	62	60	M-->V
2GSOA	58	62	61	L-->R

2GSOA	58 - 62	61	L-->T
2GSOA	58 - 62	62	D-->E
2GSOA	58 - 62	62	D-->F
2GSOA	58 - 62	62	D-->G
2GSOA	58 - 62	62	D-->M
2GSOA	58 - 62	62	D-->S
2GSOA	348 - 353	349	D-->I
2GSOA	348 - 353	352	A-->I
2GSOA	348 - 353	352	A-->K
2RE2B	53 - 58	54	K-->L
2RE2B	53 - 58	57	L-->F
2RE2B	53 - 58	57	L-->G
2RE2B	53 - 58	57	L-->P
2J6BA	93 - 99	95	E-->N
2VEBA	139 - 144	142	L-->P
2VEBA	146 - 151	151	A-->P
2GZQA	7 - 12	8	E-->S
2GZQA	7 - 12	11	K-->P
2G8MA	70 - 74	70	A-->M
2G8MA	70 - 74	70	A-->V
2G8MA	70 - 74	71	Y-->L
2G8MA	70 - 74	71	Y-->Q
2G8MA	70 - 74	71	Y-->R
2G8MA	70 - 74	73	H-->L
2G8MA	70 - 74	73	H-->P
2G8MA	70 - 74	73	H-->V
2G8MA	70 - 74	73	H-->Y

2G8MA	70	74	74	E-->F
2G8MA	70	74	74	E-->G
2G8MA	70	74	74	E-->I
2G8MA	70	74	74	E-->L
2G8MA	70	74	74	E-->M
2G8MA	70	74	74	E-->S
2G8MA	70	74	74	E-->V
2G8MA	135	140	137	E-->G
2G8MA	135	140	137	E-->L
2G8MA	135	140	139	D-->W
2G8MA	135	140	140	K-->I
2G8MA	135	140	140	K-->V
2F69A	252	256	252	H-->I
2F69A	252	256	252	H-->L
2F69A	252	256	252	H-->M
2F69A	252	256	252	H-->S
2F69A	252	256	252	H-->Y
2F69A	252	256	253	Q-->A
2F69A	252	256	253	Q-->N
2F69A	252	256	253	Q-->R
2F69A	252	256	253	Q-->Y
2F69A	252	256	254	E-->D
2F69A	252	256	254	E-->Y
2F69A	252	256	255	V-->S
2F69A	252	256	255	V-->W
2F69A	252	256	256	D-->L
2F69A	252	256	256	D-->N

2F69A	252	256	256	D-->P
2F69A	252	256	256	D-->S
2F69A	252	256	256	D-->W
1GK9A	117	122	118	Q-->R
2E3BA	63	67	63	P-->A
2E3BA	63	67	63	P-->D
2E3BA	63	67	63	P-->G
2E3BA	63	67	63	P-->H
2E3BA	63	67	63	P-->I
2E3BA	63	67	63	P-->L
2E3BA	63	67	63	P-->M
2E3BA	63	67	63	P-->Q
2E3BA	63	67	63	P-->R
2E3BA	63	67	63	P-->S
2E3BA	63	67	63	P-->V
2E3BA	63	67	63	P-->Y
2E3BA	63	67	64	A-->D
2E3BA	63	67	64	A-->E
2E3BA	63	67	64	A-->F
2E3BA	63	67	64	A-->G
2E3BA	63	67	64	A-->H
2E3BA	63	67	64	A-->I
2E3BA	63	67	64	A-->K
2E3BA	63	67	64	A-->L
2E3BA	63	67	64	A-->N
2E3BA	63	67	64	A-->R
2E3BA	63	67	64	A-->S

2E3BA	63 - 67	64	A-->V
2E3BA	63 - 67	64	A-->Y
2E3BA	63 - 67	65	L-->A
2E3BA	63 - 67	65	L-->C
2E3BA	63 - 67	65	L-->F
2E3BA	63 - 67	65	L-->G
2E3BA	63 - 67	65	L-->H
2E3BA	63 - 67	65	L-->I
2E3BA	63 - 67	65	L-->N
2E3BA	63 - 67	65	L-->Q
2E3BA	63 - 67	65	L-->T
2E3BA	63 - 67	65	L-->V
2E3BA	63 - 67	66	T-->A
2E3BA	63 - 67	66	T-->D
2E3BA	63 - 67	66	T-->G
2E3BA	63 - 67	66	T-->H
2E3BA	63 - 67	66	T-->K
2E3BA	63 - 67	66	T-->N
2E3BA	63 - 67	66	T-->P
2E3BA	63 - 67	66	T-->Q
2E3BA	63 - 67	66	T-->V
2E3BA	63 - 67	67	A-->D
2E3BA	63 - 67	67	A-->G
2E3BA	63 - 67	67	A-->L
2E3BA	63 - 67	67	A-->N
2E3BA	63 - 67	67	A-->P
2E3BA	63 - 67	67	A-->R

2E3BA	63 - 67	67	A-->S	
2E3BA	63 - 67	67	A-->T	
2E3BA	63 - 67	67	A-->V	
2E3BA	63 - 67	67	A-->Y	
2E3BA	211 - 216	215	E-->K	
1GK9B	367 - 371	367	F-->A	
1GK9B	367 - 371	369	K-->P	
1GK9B	367 - 371	370	W-->D	
1GK9B	367 - 371	370	W-->G	
1GK9B	367 - 371	370	W-->L	
1GK9B	367 - 371	370	W-->P	
1GK9B	367 - 371	370	W-->R	
1GK9B	367 - 371	370	W-->T	
1GK9B	367 - 371	370	W-->V	
1GK9B	367 - 371	371	Y-->D	
1GK9B	525 - 529	525	L-->G	
1GK9B	525 - 529	525	L-->N	
1GK9B	525 - 529	526	K-->R	
1GK9B	525 - 529	527	M-->G	
1GK9B	525 - 529	527	M-->K	
1GK9B	525 - 529	527	M-->P	
1GK9B	525 - 529	527	M-->R	
1GK9B	525 - 529	527	M-->S	
1GK9B	525 - 529	527	M-->V	
1GK9B	525 - 529	528	Y-->C	
1GK9B	525 - 529	528	Y-->D	
1GK9B	525 - 529	528	Y-->E	

1GK9B	525 - 529	528	Y-->F
1GK9B	525 - 529	528	Y-->H
1GK9B	525 - 529	528	Y-->K
1GK9B	525 - 529	528	Y-->L
1GK9B	525 - 529	528	Y-->N
1GK9B	525 - 529	528	Y-->R
1GK9B	525 - 529	529	E-->P
1GK9B	540 - 545	541	Q-->L
1GK9B	540 - 545	542	D-->A
1GK9B	540 - 545	543	V-->G
1GK9B	540 - 545	544	E-->A
1GK9B	540 - 545	544	E-->R
1WVFA	161 - 166	166	M-->A
1WVFA	161 - 166	166	M-->E
1NXMA	148 - 153	148	L-->S
1NXMA	148 - 153	148	L-->V
1NXMA	148 - 153	150	L-->E
1NXMA	148 - 153	151	K-->P
1NXMA	148 - 153	152	P-->L
1NXMA	148 - 153	152	P-->Y
1LQ9A	38 - 42	39	W-->E
1LQ9A	38 - 42	39	W-->G
1LQ9A	38 - 42	39	W-->M
1LQ9A	38 - 42	39	W-->R
1LQ9A	38 - 42	40	I-->V
1LQ9A	38 - 42	41	R-->G
1LQ9A	38 - 42	41	R-->P

1LQ9A	38 - 42	42	E-->Q
1LQ9A	69 - 75	74	V-->I
1W2LA	78 - 82	78	A-->D
1W2LA	78 - 82	78	A-->F
1W2LA	78 - 82	78	A-->G
1W2LA	78 - 82	78	A-->N
1W2LA	78 - 82	78	A-->T
1W2LA	78 - 82	78	A-->V
1W2LA	78 - 82	79	S-->A
1W2LA	78 - 82	79	S-->F
1W2LA	78 - 82	79	S-->K
1W2LA	78 - 82	79	S-->L
1W2LA	78 - 82	79	S-->Q
1W2LA	78 - 82	79	S-->R
1W2LA	78 - 82	79	S-->T
1W2LA	78 - 82	80	Y-->A
1W2LA	78 - 82	80	Y-->E
1W2LA	78 - 82	80	Y-->G
1W2LA	78 - 82	80	Y-->K
1W2LA	78 - 82	80	Y-->L
1W2LA	78 - 82	80	Y-->M
1W2LA	78 - 82	80	Y-->P
1W2LA	78 - 82	80	Y-->Q
1W2LA	78 - 82	80	Y-->R
1W2LA	78 - 82	80	Y-->S
1W2LA	78 - 82	80	Y-->T
1W2LA	78 - 82	81	A-->D

1W2LA	78 - 82	81	A-->F
1W2LA	78 - 82	81	A-->I
1W2LA	78 - 82	81	A-->P
1W2LA	78 - 82	81	A-->Q
1W2LA	78 - 82	81	A-->R
1W2LA	78 - 82	81	A-->S
1W2LA	78 - 82	81	A-->T
1W2LA	78 - 82	81	A-->W
1W2LA	78 - 82	82	S-->G
1W2LA	78 - 82	82	S-->K
1W2LA	78 - 82	82	S-->L
1W2LA	78 - 82	82	S-->N
1W2LA	78 - 82	82	S-->P
1W2LA	78 - 82	82	S-->T
1W2LA	78 - 82	82	S-->Y
2OKTA	83 - 88	83	Y-->G
2OKTA	83 - 88	83	Y-->N
2OKTA	83 - 88	83	Y-->P
2OKTA	202 - 207	204	M-->D
2OKTA	202 - 207	207	E-->K
1KA1A	147 - 151	147	T-->D
1KA1A	147 - 151	147	T-->E
1KA1A	147 - 151	147	T-->F
1KA1A	147 - 151	147	T-->K
1KA1A	147 - 151	147	T-->N
1KA1A	147 - 151	147	T-->Q
1KA1A	147 - 151	147	T-->S

1KA1A	147 - 151	147	T-->V	
1KA1A	147 - 151	148	K-->D	
1KA1A	147 - 151	148	K-->E	
1KA1A	147 - 151	148	K-->G	
1KA1A	147 - 151	148	K-->H	
1KA1A	147 - 151	148	K-->P	
1KA1A	147 - 151	148	K-->S	
1KA1A	147 - 151	148	K-->V	
1KA1A	147 - 151	149	G-->I	
1KA1A	147 - 151	149	G-->L	
1KA1A	147 - 151	149	G-->Y	
1KA1A	147 - 151	150	F-->D	
1KA1A	147 - 151	150	F-->G	
1KA1A	147 - 151	150	F-->I	
1KA1A	147 - 151	150	F-->K	
1KA1A	147 - 151	150	F-->R	
1KA1A	147 - 151	150	F-->T	
1KA1A	147 - 151	150	F-->V	
1KA1A	147 - 151	150	F-->Y	
1KA1A	147 - 151	151	L-->D	
1KA1A	147 - 151	151	L-->F	
1KA1A	147 - 151	151	L-->K	
1KA1A	147 - 151	151	L-->N	
1KA1A	147 - 151	151	L-->P	
1KA1A	147 - 151	151	L-->S	
1KA1A	147 - 151	151	L-->T	
1KA1A	147 - 151	151	L-->V	

1KA1A	147 - 151	151	L-->Y
1DCSA	10 - 14	10	L-->A
1DCSA	10 - 14	10	L-->D
1DCSA	10 - 14	10	L-->E
1DCSA	10 - 14	10	L-->F
1DCSA	10 - 14	10	L-->N
1DCSA	10 - 14	10	L-->S
1DCSA	10 - 14	10	L-->T
1DCSA	10 - 14	10	L-->V
1DCSA	10 - 14	11	A-->D
1DCSA	10 - 14	11	A-->E
1DCSA	10 - 14	11	A-->G
1DCSA	10 - 14	11	A-->I
1DCSA	10 - 14	11	A-->L
1DCSA	10 - 14	11	A-->N
1DCSA	10 - 14	11	A-->P
1DCSA	10 - 14	11	A-->R
1DCSA	10 - 14	11	A-->V
1DCSA	10 - 14	12	E-->A
1DCSA	10 - 14	12	E-->D
1DCSA	10 - 14	12	E-->G
1DCSA	10 - 14	12	E-->I
1DCSA	10 - 14	12	E-->K
1DCSA	10 - 14	12	E-->P
1DCSA	10 - 14	12	E-->S
1DCSA	10 - 14	12	E-->T
1DCSA	10 - 14	12	E-->V

1DCSA	10 - 14	12	E-->Y	
1DCSA	10 - 14	13	L-->E	
1DCSA	10 - 14	13	L-->G	
1DCSA	10 - 14	13	L-->H	
1DCSA	10 - 14	13	L-->I	
1DCSA	10 - 14	13	L-->N	
1DCSA	10 - 14	13	L-->P	
1DCSA	10 - 14	13	L-->S	
1DCSA	10 - 14	13	L-->T	
1DCSA	10 - 14	13	L-->Y	
1DCSA	10 - 14	14	Q-->D	
1DCSA	10 - 14	14	Q-->G	
1DCSA	10 - 14	14	Q-->L	
1DCSA	10 - 14	14	Q-->N	
1DCSA	10 - 14	14	Q-->P	
1DCSA	10 - 14	14	Q-->R	
1DCSA	10 - 14	14	Q-->S	
1DCSA	10 - 14	14	Q-->T	
1DCSA	60 - 65	62	E-->P	
1DCSA	149 - 153	149	V-->D	
1DCSA	149 - 153	149	V-->G	
1DCSA	149 - 153	149	V-->I	
1DCSA	149 - 153	149	V-->K	
1DCSA	149 - 153	149	V-->L	
1DCSA	149 - 153	149	V-->N	
1DCSA	149 - 153	149	V-->S	
1DCSA	149 - 153	150	E-->A	

1DCSA	149 - 153	150	E-->G
1DCSA	149 - 153	150	E-->L
1DCSA	149 - 153	150	E-->N
1DCSA	149 - 153	150	E-->P
1DCSA	149 - 153	150	E-->S
1DCSA	149 - 153	150	E-->T
1DCSA	149 - 153	150	E-->Y
1DCSA	149 - 153	151	A-->E
1DCSA	149 - 153	151	A-->F
1DCSA	149 - 153	151	A-->G
1DCSA	149 - 153	151	A-->N
1DCSA	149 - 153	152	F-->A
1DCSA	149 - 153	152	F-->E
1DCSA	149 - 153	152	F-->G
1DCSA	149 - 153	152	F-->I
1DCSA	149 - 153	152	F-->L
1DCSA	149 - 153	152	F-->N
1DCSA	149 - 153	152	F-->P
1DCSA	149 - 153	152	F-->R
1DCSA	149 - 153	152	F-->V
1DCSA	149 - 153	153	L-->C
1DCSA	149 - 153	153	L-->I
1DCSA	149 - 153	153	L-->P
1DCSA	149 - 153	153	L-->S
1DCSA	149 - 153	153	L-->V
1DCSA	149 - 153	153	L-->W
1DCSA	228 - 233	228	A-->R

1DCSA	228	233	229	I-->F
1DCSA	228	233	229	I-->K
1DCSA	228	233	229	I-->N
1DCSA	228	233	229	I-->T
1DCSA	228	233	230	A-->L
1DCSA	228	233	233	V-->S
1DCSA	228	233	233	V-->Y
1DCSA	275	280	276	P-->V
1DCSA	275	280	276	P-->Y
1DCSA	275	280	278	A-->V
1DCSA	294	298	294	F-->W
1DCSA	294	298	295	Q-->E
1DCSA	294	298	295	Q-->W
1DCSA	294	298	296	D-->N
1DCSA	294	298	297	W-->K
1DCSA	294	298	297	W-->V
1DCSA	294	298	298	I-->A
1PQHB	73	77	73	G-->I
1PQHB	73	77	73	G-->N
1PQHB	73	77	73	G-->V
1PQHB	73	77	74	A-->G
1PQHB	73	77	74	A-->I
1PQHB	73	77	74	A-->K
1PQHB	73	77	74	A-->S
1PQHB	73	77	74	A-->V
1PQHB	73	77	74	A-->Y
1PQHB	73	77	75	A-->D

1PQHB	73 - 77	75	A-->E
1PQHB	73 - 77	75	A-->G
1PQHB	73 - 77	75	A-->K
1PQHB	73 - 77	75	A-->L
1PQHB	73 - 77	75	A-->N
1PQHB	73 - 77	75	A-->T
1PQHB	73 - 77	75	A-->V
1PQHB	73 - 77	76	A-->C
1PQHB	73 - 77	76	A-->D
1PQHB	73 - 77	76	A-->F
1PQHB	73 - 77	76	A-->G
1PQHB	73 - 77	76	A-->N
1PQHB	73 - 77	76	A-->P
1PQHB	73 - 77	76	A-->V
1PQHB	73 - 77	77	H-->A
1PQHB	73 - 77	77	H-->C
1PQHB	73 - 77	77	H-->D
1PQHB	73 - 77	77	H-->E
1PQHB	73 - 77	77	H-->G
1PQHB	73 - 77	77	H-->I
1PQHB	73 - 77	77	H-->L
1PQHB	73 - 77	77	H-->N
1PQHB	73 - 77	77	H-->P
1PQHB	73 - 77	77	H-->Q
1PQHB	73 - 77	77	H-->R
1PQHB	73 - 77	77	H-->S
1PQHB	73 - 77	77	H-->T

1PQHB	73 - 77	77	H-->V
2DXUA	143 - 147	143	M-->D
2DXUA	143 - 147	143	M-->E
2DXUA	143 - 147	143	M-->F
2DXUA	143 - 147	143	M-->G
2DXUA	143 - 147	143	M-->H
2DXUA	143 - 147	143	M-->I
2DXUA	143 - 147	143	M-->K
2DXUA	143 - 147	143	M-->L
2DXUA	143 - 147	143	M-->Q
2DXUA	143 - 147	143	M-->S
2DXUA	143 - 147	143	M-->T
2DXUA	143 - 147	143	M-->V
2DXUA	143 - 147	144	K-->G
2DXUA	143 - 147	144	K-->H
2DXUA	143 - 147	144	K-->L
2DXUA	143 - 147	145	L-->G
2DXUA	143 - 147	145	L-->I
2DXUA	143 - 147	145	L-->R
2DXUA	143 - 147	146	E-->H
2DXUA	143 - 147	146	E-->R
2DXUA	143 - 147	147	L-->A
2DXUA	143 - 147	147	L-->D
2DXUA	143 - 147	147	L-->G
2DXUA	143 - 147	147	L-->I
2DXUA	143 - 147	147	L-->R
2DXUA	143 - 147	147	L-->S

2DXUA	143	147	147	L-->V
2DXUA	143	147	147	L-->W
1QKSA	492	496	492	I-->D
1QKSA	492	496	492	I-->E
1QKSA	492	496	492	I-->H
1QKSA	492	496	492	I-->P
1QKSA	492	496	492	I-->R
1QKSA	492	496	493	A-->G
1QKSA	492	496	494	E-->N
1QKSA	492	496	495	W-->A
1QKSA	492	496	495	W-->D
1QKSA	492	496	495	W-->G
1QKSA	492	496	495	W-->H
1QKSA	492	496	495	W-->K
1QKSA	492	496	495	W-->N
1QKSA	492	496	495	W-->P
1QKSA	492	496	496	A-->K
1QKSA	492	496	496	A-->Y
1QKSA	559	563	560	Y-->V
1QKSA	559	563	561	N-->G
1QKSA	559	563	561	N-->K
1QKSA	559	563	562	T-->R
1QKSA	559	563	562	T-->Y
1QKSA	559	563	563	M-->E
1QKSA	559	563	563	M-->G
1QKSA	559	563	563	M-->K
1QKSA	559	563	563	M-->T

2PC1A	75 - 79	75	E-->D
2PC1A	75 - 79	75	E-->F
2PC1A	75 - 79	75	E-->G
2PC1A	75 - 79	75	E-->H
2PC1A	75 - 79	75	E-->I
2PC1A	75 - 79	75	E-->P
2PC1A	75 - 79	75	E-->R
2PC1A	75 - 79	75	E-->S
2PC1A	75 - 79	75	E-->V
2PC1A	75 - 79	76	V-->A
2PC1A	75 - 79	76	V-->P
2PC1A	75 - 79	76	V-->S
2PC1A	75 - 79	77	Y-->D
2PC1A	75 - 79	77	Y-->G
2PC1A	75 - 79	77	Y-->I
2PC1A	75 - 79	77	Y-->S
2PC1A	75 - 79	77	Y-->T
2PC1A	75 - 79	77	Y-->V
2PC1A	75 - 79	77	Y-->W
2PC1A	75 - 79	78	D-->A
2PC1A	75 - 79	78	D-->F
2PC1A	75 - 79	78	D-->G
2PC1A	75 - 79	78	D-->H
2PC1A	75 - 79	78	D-->I
2PC1A	75 - 79	78	D-->V
2PC1A	75 - 79	78	D-->W
2PC1A	75 - 79	79	A-->E

2PC1A	75 - 79	79	A-->G
2PC1A	75 - 79	79	A-->L
2PC1A	75 - 79	79	A-->M
2PC1A	75 - 79	79	A-->R
2PC1A	75 - 79	79	A-->S
2PC1A	75 - 79	79	A-->T
2PC1A	75 - 79	79	A-->V
2PC1A	75 - 79	79	A-->W
2PC1A	138 - 143	139	H-->C
3CBWA	278 - 282	278	Y-->A
3CBWA	278 - 282	278	Y-->D
3CBWA	278 - 282	278	Y-->E
3CBWA	278 - 282	278	Y-->G
3CBWA	278 - 282	278	Y-->I
3CBWA	278 - 282	278	Y-->K
3CBWA	278 - 282	278	Y-->L
3CBWA	278 - 282	278	Y-->N
3CBWA	278 - 282	278	Y-->P
3CBWA	278 - 282	278	Y-->Q
3CBWA	278 - 282	278	Y-->T
3CBWA	278 - 282	278	Y-->V
3CBWA	278 - 282	279	D-->H
3CBWA	278 - 282	279	D-->L
3CBWA	278 - 282	279	D-->M
3CBWA	278 - 282	279	D-->N
3CBWA	278 - 282	279	D-->P
3CBWA	278 - 282	279	D-->T

3CBWA	278 - 282	279	D-->V
3CBWA	278 - 282	280	Q-->F
3CBWA	278 - 282	280	Q-->G
3CBWA	278 - 282	280	Q-->K
3CBWA	278 - 282	280	Q-->P
3CBWA	278 - 282	280	Q-->R
3CBWA	278 - 282	280	Q-->T
3CBWA	278 - 282	281	L-->A
3CBWA	278 - 282	281	L-->R
3CBWA	278 - 282	281	L-->S
3CBWA	278 - 282	282	T-->E
3CBWA	278 - 282	282	T-->G
3CBWA	278 - 282	282	T-->I
3CBWA	278 - 282	282	T-->S
3CBWA	336 - 341	340	Y-->C
3CBWA	336 - 341	341	H-->T
2E6FA	272 - 277	273	A-->L
2E6FA	272 - 277	274	L-->G
2FCWB	122 - 126	122	C-->A
2FCWB	122 - 126	122	C-->D
2FCWB	122 - 126	122	C-->G
2FCWB	122 - 126	122	C-->I
2FCWB	122 - 126	122	C-->L
2FCWB	122 - 126	122	C-->M
2FCWB	122 - 126	122	C-->N
2FCWB	122 - 126	122	C-->Q
2FCWB	122 - 126	122	C-->S

2FCWB	122 - 126	122	C-->T
2FCWB	122 - 126	122	C-->V
2FCWB	122 - 126	122	C-->W
2FCWB	122 - 126	122	C-->Y
2FCWB	122 - 126	123	P-->A
2FCWB	122 - 126	123	P-->G
2FCWB	122 - 126	123	P-->R
2FCWB	122 - 126	123	P-->T
2FCWB	122 - 126	123	P-->W
2FCWB	122 - 126	124	V-->D
2FCWB	122 - 126	124	V-->H
2FCWB	122 - 126	125	L-->D
2FCWB	122 - 126	125	L-->E
2FCWB	122 - 126	125	L-->S
2FCWB	122 - 126	126	T-->A
2FCWB	122 - 126	126	T-->D
2FCWB	122 - 126	126	T-->I
2FCWB	122 - 126	126	T-->L
2FCWB	122 - 126	126	T-->Y
2DY0A	32 - 36	32	T-->A
2DY0A	32 - 36	32	T-->E
2DY0A	32 - 36	32	T-->I
2DY0A	32 - 36	32	T-->K
2DY0A	32 - 36	32	T-->N
2DY0A	32 - 36	32	T-->P
2DY0A	32 - 36	32	T-->R
2DY0A	32 - 36	32	T-->S

2DY0A	32	36	32	T-->V
2DY0A	32	36	32	T-->W
2DY0A	32	36	32	T-->Y
2DY0A	32	36	33	S-->A
2DY0A	32	36	33	S-->D
2DY0A	32	36	33	S-->I
2DY0A	32	36	33	S-->L
2DY0A	32	36	33	S-->T
2DY0A	32	36	33	S-->V
2DY0A	32	36	33	S-->W
2DY0A	32	36	33	S-->Y
2DY0A	32	36	34	L-->A
2DY0A	32	36	34	L-->D
2DY0A	32	36	34	L-->G
2DY0A	32	36	34	L-->K
2DY0A	32	36	34	L-->N
2DY0A	32	36	34	L-->P
2DY0A	32	36	34	L-->S
2DY0A	32	36	34	L-->T
2DY0A	32	36	35	L-->A
2DY0A	32	36	35	L-->F
2DY0A	32	36	35	L-->G
2DY0A	32	36	35	L-->I
2DY0A	32	36	35	L-->P
2DY0A	32	36	35	L-->T
2DY0A	32	36	35	L-->W
2DY0A	32	36	36	E-->A

2DY0A	32 - 36	36	E-->D
2DY0A	32 - 36	36	E-->G
2DY0A	32 - 36	36	E-->I
2DY0A	32 - 36	36	E-->L
2DY0A	32 - 36	36	E-->M
2DY0A	32 - 36	36	E-->P
2DY0A	32 - 36	36	E-->S
2DY0A	32 - 36	36	E-->T
2DY0A	32 - 36	36	E-->V
2DY0A	32 - 36	36	E-->Y
2DY0A	163 - 168	163	G-->E
2DY0A	163 - 168	163	G-->P
2DY0A	163 - 168	164	E-->T
2DY0A	163 - 168	166	R-->G
2DY0A	163 - 168	167	L-->G
2G3RA	1590 - 1594	1590	L-->D
2G3RA	1590 - 1594	1590	L-->P
2G3RA	1590 - 1594	1590	L-->T
2G3RA	1590 - 1594	1590	L-->V
2G3RA	1590 - 1594	1591	E-->A
2G3RA	1590 - 1594	1591	E-->F
2G3RA	1590 - 1594	1591	E-->K
2G3RA	1590 - 1594	1591	E-->L
2G3RA	1590 - 1594	1591	E-->N
2G3RA	1590 - 1594	1591	E-->R
2G3RA	1590 - 1594	1591	E-->T
2G3RA	1590 - 1594	1592	Q-->A

2G3RA	1590	- 1594	1592	Q-->C
2G3RA	1590	- 1594	1592	Q-->E
2G3RA	1590	- 1594	1592	Q-->F
2G3RA	1590	- 1594	1592	Q-->G
2G3RA	1590	- 1594	1592	Q-->H
2G3RA	1590	- 1594	1592	Q-->L
2G3RA	1590	- 1594	1592	Q-->S
2G3RA	1590	- 1594	1592	Q-->T
2G3RA	1590	- 1594	1592	Q-->V
2G3RA	1590	- 1594	1592	Q-->Y
2G3RA	1590	- 1594	1593	G-->E
2G3RA	1590	- 1594	1593	G-->L
2G3RA	1590	- 1594	1593	G-->P
2G3RA	1590	- 1594	1594	N-->A
2G3RA	1590	- 1594	1594	N-->E
2G3RA	1590	- 1594	1594	N-->I
2G3RA	1590	- 1594	1594	N-->K
2G3RA	1590	- 1594	1594	N-->M
2G3RA	1590	- 1594	1594	N-->P
2G3RA	1590	- 1594	1594	N-->Q
2G3RA	1590	- 1594	1594	N-->S
2G3RA	1590	- 1594	1594	N-->T
2G3RA	1590	- 1594	1594	N-->V
2G3RA	1590	- 1594	1594	N-->W
1Y6XA	86	- 90	86	L-->A
1Y6XA	86	- 90	86	L-->G
1Y6XA	86	- 90	86	L-->H

1Y6XA	86 - 90	86	L-->P
1Y6XA	86 - 90	86	L-->Q
1Y6XA	86 - 90	87	D-->A
1Y6XA	86 - 90	87	D-->E
1Y6XA	86 - 90	87	D-->G
1Y6XA	86 - 90	87	D-->I
1Y6XA	86 - 90	87	D-->L
1Y6XA	86 - 90	87	D-->P
1Y6XA	86 - 90	87	D-->Q
1Y6XA	86 - 90	87	D-->R
1Y6XA	86 - 90	87	D-->T
1Y6XA	86 - 90	87	D-->V
1Y6XA	86 - 90	88	D-->G
1Y6XA	86 - 90	88	D-->I
1Y6XA	86 - 90	88	D-->K
1Y6XA	86 - 90	88	D-->P
1Y6XA	86 - 90	88	D-->R
1Y6XA	86 - 90	88	D-->S
1Y6XA	86 - 90	88	D-->T
1Y6XA	86 - 90	88	D-->V
1Y6XA	86 - 90	88	D-->W
1Y6XA	86 - 90	89	V-->A
1Y6XA	86 - 90	89	V-->E
1Y6XA	86 - 90	89	V-->H
1Y6XA	86 - 90	89	V-->I
1Y6XA	86 - 90	89	V-->L
1Y6XA	86 - 90	89	V-->N

1Y6XA	86 - 90	89	V-->Q	
1Y6XA	86 - 90	89	V-->S	
1Y6XA	86 - 90	89	V-->Y	
1Y6XA	86 - 90	90	Y-->A	
1Y6XA	86 - 90	90	Y-->D	
1Y6XA	86 - 90	90	Y-->E	
1Y6XA	86 - 90	90	Y-->F	
1Y6XA	86 - 90	90	Y-->G	
1Y6XA	86 - 90	90	Y-->K	
1Y6XA	86 - 90	90	Y-->Q	
1Y6XA	86 - 90	90	Y-->T	
2TPSA	195 - 199	195	A-->E	
2TPSA	195 - 199	195	A-->G	
2TPSA	195 - 199	195	A-->H	
2TPSA	195 - 199	195	A-->I	
2TPSA	195 - 199	195	A-->R	
2TPSA	195 - 199	195	A-->V	
2TPSA	195 - 199	195	A-->Y	
2TPSA	195 - 199	196	P-->A	
2TPSA	195 - 199	196	P-->E	
2TPSA	195 - 199	196	P-->G	
2TPSA	195 - 199	196	P-->L	
2TPSA	195 - 199	196	P-->T	
2TPSA	195 - 199	196	P-->V	
2TPSA	195 - 199	197	V-->A	
2TPSA	195 - 199	197	V-->E	
2TPSA	195 - 199	197	V-->H	

2TPSA	195 - 199	197	V-->L
2TPSA	195 - 199	197	V-->N
2TPSA	195 - 199	197	V-->R
2TPSA	195 - 199	198	I-->A
2TPSA	195 - 199	198	I-->D
2TPSA	195 - 199	198	I-->L
2TPSA	195 - 199	198	I-->N
2TPSA	195 - 199	198	I-->P
2TPSA	195 - 199	198	I-->R
2TPSA	195 - 199	198	I-->T
2TPSA	195 - 199	199	Q-->A
2TPSA	195 - 199	199	Q-->D
2TPSA	195 - 199	199	Q-->F
2TPSA	195 - 199	199	Q-->G
2TPSA	195 - 199	199	Q-->H
2TPSA	195 - 199	199	Q-->K
2TPSA	195 - 199	199	Q-->L
2TPSA	195 - 199	199	Q-->N
2TPSA	195 - 199	199	Q-->P
2TPSA	195 - 199	199	Q-->R
2TPSA	195 - 199	199	Q-->S
2TPSA	195 - 199	199	Q-->T
2TPSA	195 - 199	199	Q-->V
2TPSA	195 - 199	199	Q-->W
2FVVA	108 - 113	110	S-->G
2FVVA	134 - 139	134	P-->K
2FVVA	134 - 139	134	P-->W

2FVVA	134 - 139	138	S-->V
2FVVA	134 - 139	139	Y-->N
2B82A	12 - 16	12	V-->F
2B82A	12 - 16	12	V-->H
2B82A	12 - 16	12	V-->I
2B82A	12 - 16	12	V-->L
2B82A	12 - 16	12	V-->N
2B82A	12 - 16	12	V-->P
2B82A	12 - 16	12	V-->T
2B82A	12 - 16	12	V-->Y
2B82A	12 - 16	13	A-->I
2B82A	12 - 16	13	A-->P
2B82A	12 - 16	13	A-->Q
2B82A	12 - 16	13	A-->T
2B82A	12 - 16	13	A-->V
2B82A	12 - 16	14	R-->A
2B82A	12 - 16	14	R-->E
2B82A	12 - 16	14	R-->F
2B82A	12 - 16	14	R-->G
2B82A	12 - 16	14	R-->H
2B82A	12 - 16	14	R-->I
2B82A	12 - 16	14	R-->K
2B82A	12 - 16	14	R-->L
2B82A	12 - 16	14	R-->N
2B82A	12 - 16	14	R-->P
2B82A	12 - 16	14	R-->T
2B82A	12 - 16	14	R-->V

2B82A	12 - 16	14	R-->Y
2B82A	12 - 16	15	L-->E
2B82A	12 - 16	15	L-->I
2B82A	12 - 16	15	L-->N
2B82A	12 - 16	15	L-->V
2B82A	12 - 16	16	A-->K
2B82A	12 - 16	16	A-->L
2B82A	12 - 16	16	A-->M
2B82A	12 - 16	16	A-->N
2B82A	12 - 16	16	A-->P
2B82A	12 - 16	16	A-->Q
2B82A	12 - 16	16	A-->S
2B82A	12 - 16	16	A-->T
2B82A	12 - 16	16	A-->V
2B82A	153 - 158	153	S-->L
2B82A	153 - 158	155	W-->F
2Q6MA	484 - 488	484	E-->A
2Q6MA	484 - 488	484	E-->F
2Q6MA	484 - 488	484	E-->G
2Q6MA	484 - 488	484	E-->P
2Q6MA	484 - 488	484	E-->T
2Q6MA	484 - 488	484	E-->V
2Q6MA	484 - 488	484	E-->Y
2Q6MA	484 - 488	485	N-->F
2Q6MA	484 - 488	485	N-->I
2Q6MA	484 - 488	485	N-->K
2Q6MA	484 - 488	485	N-->L

2Q6MA	484	488	485	N-->Q
2Q6MA	484	488	485	N-->R
2Q6MA	484	488	485	N-->T
2Q6MA	484	488	485	N-->V
2Q6MA	484	488	486	E-->G
2Q6MA	484	488	486	E-->K
2Q6MA	484	488	486	E-->L
2Q6MA	484	488	486	E-->N
2Q6MA	484	488	486	E-->P
2Q6MA	484	488	486	E-->Q
2Q6MA	484	488	486	E-->V
2Q6MA	484	488	487	E-->D
2Q6MA	484	488	487	E-->I
2Q6MA	484	488	487	E-->K
2Q6MA	484	488	487	E-->M
2Q6MA	484	488	487	E-->N
2Q6MA	484	488	487	E-->R
2Q6MA	484	488	488	K-->A
2Q6MA	484	488	488	K-->D
2Q6MA	484	488	488	K-->E
2Q6MA	484	488	488	K-->F
2Q6MA	484	488	488	K-->G
2Q6MA	484	488	488	K-->S
2Q6MA	484	488	488	K-->T
2Q6MA	484	488	488	K-->V
2Q6MA	498	502	498	A-->E
2Q6MA	498	502	498	A-->I

2Q6MA	498 - 502	498	A-->S
2Q6MA	498 - 502	499	E-->F
2Q6MA	498 - 502	499	E-->G
2Q6MA	498 - 502	499	E-->H
2Q6MA	498 - 502	499	E-->L
2Q6MA	498 - 502	499	E-->N
2Q6MA	498 - 502	499	E-->P
2Q6MA	498 - 502	499	E-->S
2Q6MA	498 - 502	500	V-->A
2Q6MA	498 - 502	500	V-->E
2Q6MA	498 - 502	501	A-->D
2Q6MA	498 - 502	501	A-->E
2Q6MA	498 - 502	501	A-->G
2Q6MA	498 - 502	501	A-->I
2Q6MA	498 - 502	501	A-->L
2Q6MA	498 - 502	501	A-->N
2Q6MA	498 - 502	501	A-->P
2Q6MA	498 - 502	501	A-->R
2Q6MA	498 - 502	501	A-->V
2Q6MA	498 - 502	502	H-->A
2Q6MA	498 - 502	502	H-->D
2Q6MA	498 - 502	502	H-->E
2Q6MA	498 - 502	502	H-->F
2Q6MA	498 - 502	502	H-->I
2Q6MA	498 - 502	502	H-->K
2Q6MA	498 - 502	502	H-->L
2Q6MA	498 - 502	502	H-->N

2Q6MA	498 - 502	502	H-->T
2Q6MA	498 - 502	502	H-->V
2Q6MA	519 - 523	519	R-->D
2Q6MA	519 - 523	519	R-->E
2Q6MA	519 - 523	519	R-->K
2Q6MA	519 - 523	519	R-->L
2Q6MA	519 - 523	519	R-->P
2Q6MA	519 - 523	519	R-->T
2Q6MA	519 - 523	519	R-->V
2Q6MA	519 - 523	519	R-->Y
2Q6MA	519 - 523	520	A-->E
2Q6MA	519 - 523	520	A-->G
2Q6MA	519 - 523	520	A-->I
2Q6MA	519 - 523	520	A-->K
2Q6MA	519 - 523	520	A-->L
2Q6MA	519 - 523	520	A-->S
2Q6MA	519 - 523	521	E-->G
2Q6MA	519 - 523	521	E-->L
2Q6MA	519 - 523	521	E-->Q
2Q6MA	519 - 523	521	E-->S
2Q6MA	519 - 523	521	E-->T
2Q6MA	519 - 523	521	E-->V
2Q6MA	519 - 523	521	E-->W
2Q6MA	519 - 523	521	E-->Y
2Q6MA	519 - 523	522	R-->F
2Q6MA	519 - 523	522	R-->G
2Q6MA	519 - 523	522	R-->H

2Q6MA	519 - 523	522	R-->L
2Q6MA	519 - 523	522	R-->V
2Q6MA	519 - 523	523	D-->A
2Q6MA	519 - 523	523	D-->E
2Q6MA	519 - 523	523	D-->I
2Q6MA	519 - 523	523	D-->L
2Q6MA	519 - 523	523	D-->N
2Q6MA	519 - 523	523	D-->P
2Q6MA	519 - 523	523	D-->S
2Q6MA	519 - 523	523	D-->V
2Q6MA	535 - 540	538	L-->Q
1K3XA	35 - 39	35	Y-->T
1K3XA	35 - 39	36	Q-->E
1K3XA	35 - 39	36	Q-->F
1K3XA	35 - 39	36	Q-->R
1K3XA	35 - 39	37	S-->D
1K3XA	35 - 39	37	S-->G
1K3XA	35 - 39	37	S-->P
1K3XA	35 - 39	38	Q-->A
1K3XA	35 - 39	38	Q-->D
1K3XA	35 - 39	38	Q-->G
1K3XA	35 - 39	38	Q-->L
1K3XA	35 - 39	38	Q-->N
1K3XA	35 - 39	38	Q-->V
1K3XA	35 - 39	38	Q-->Y
1K3XA	35 - 39	39	L-->I
1K3XA	35 - 39	39	L-->P

1K3XA	120	124	120	P-->F
1K3XA	120	124	120	P-->V
1K3XA	120	124	120	P-->W
1K3XA	120	124	121	F-->Q
1K3XA	120	124	121	F-->Y
1K3XA	120	124	122	L-->A
1K3XA	120	124	122	L-->E
1K3XA	120	124	122	L-->Y
1K3XA	120	124	123	Q-->A
1K3XA	120	124	123	Q-->D
1K3XA	120	124	123	Q-->G
1K3XA	120	124	123	Q-->H
1K3XA	120	124	123	Q-->I
1K3XA	120	124	123	Q-->K
1K3XA	120	124	123	Q-->P
1K3XA	120	124	123	Q-->V
1K3XA	120	124	124	R-->C
1EAQB	52	57	52	V-->L
1EAQB	52	57	53	E-->A
1EAQB	52	57	53	E-->T
1EAQB	52	57	53	E-->Y
1EAQB	52	57	54	V-->F
1EAQB	52	57	56	A-->G
1EAQB	52	57	56	A-->L
1EAQB	52	57	57	D-->T
1S3CA	64	68	64	E-->H
1S3CA	64	68	64	E-->N

1S3CA	64 - 68	64	E-->V
1S3CA	64 - 68	65	P-->D
1S3CA	64 - 68	65	P-->I
1S3CA	64 - 68	65	P-->L
1S3CA	64 - 68	65	P-->T
1S3CA	64 - 68	65	P-->V
1S3CA	64 - 68	66	Y-->D
1S3CA	64 - 68	66	Y-->T
1S3CA	64 - 68	67	E-->A
1S3CA	64 - 68	67	E-->F
1S3CA	64 - 68	67	E-->L
1S3CA	64 - 68	68	Q-->A
1S3CA	64 - 68	68	Q-->G
1S3CA	64 - 68	68	Q-->K
1S3CA	64 - 68	68	Q-->L
1S3CA	64 - 68	68	Q-->N
1S3CA	64 - 68	68	Q-->P
1S3CA	64 - 68	68	Q-->V
1S3CA	109 - 114	109	S-->E
1S3CA	109 - 114	109	S-->F
1S3CA	109 - 114	114	D-->L
2O37A	6 - 11	6	K-->D
2O37A	6 - 11	6	K-->V
2O37A	6 - 11	9	D-->S
2O37A	69 - 73	69	L-->A
2O37A	69 - 73	69	L-->D
2O37A	69 - 73	69	L-->F

2O37A	69 - 73	69	L-->G
2O37A	69 - 73	69	L-->M
2O37A	69 - 73	69	L-->R
2O37A	69 - 73	69	L-->V
2O37A	69 - 73	70	E-->A
2O37A	69 - 73	70	E-->G
2O37A	69 - 73	70	E-->K
2O37A	69 - 73	70	E-->L
2O37A	69 - 73	70	E-->N
2O37A	69 - 73	70	E-->V
2O37A	69 - 73	71	A-->D
2O37A	69 - 73	71	A-->E
2O37A	69 - 73	71	A-->F
2O37A	69 - 73	71	A-->G
2O37A	69 - 73	71	A-->I
2O37A	69 - 73	71	A-->L
2O37A	69 - 73	71	A-->P
2O37A	69 - 73	71	A-->T
2O37A	69 - 73	71	A-->Y
2O37A	69 - 73	72	A-->D
2O37A	69 - 73	72	A-->E
2O37A	69 - 73	72	A-->F
2O37A	69 - 73	72	A-->L
2O37A	69 - 73	72	A-->Q
2O37A	69 - 73	72	A-->Y
2O37A	69 - 73	73	R-->F
2O37A	69 - 73	73	R-->G

2O37A	69 - 73	73	R-->P
2O37A	69 - 73	73	R-->Q
2O37A	69 - 73	73	R-->S
2O37A	69 - 73	73	R-->V
2O37A	69 - 73	73	R-->Y
3CHBD	5 - 9	5	I-->F
3CHBD	5 - 9	5	I-->K
3CHBD	5 - 9	5	I-->S
3CHBD	5 - 9	6	T-->L
3CHBD	5 - 9	6	T-->M
3CHBD	5 - 9	7	D-->F
3CHBD	5 - 9	7	D-->T
3CHBD	5 - 9	8	L-->A
3CHBD	5 - 9	8	L-->D
3CHBD	5 - 9	8	L-->E
3CHBD	5 - 9	8	L-->F
3CHBD	5 - 9	8	L-->I
3CHBD	5 - 9	8	L-->R
3CHBD	5 - 9	9	C-->D
3CHBD	5 - 9	9	C-->I
3CHBD	5 - 9	9	C-->K
3CHBD	5 - 9	9	C-->L
3CHBD	5 - 9	9	C-->R
3CHBD	5 - 9	9	C-->S
3CHBD	5 - 9	9	C-->T
2B3NB	70 - 74	70	E-->I
2B3NB	70 - 74	70	E-->L

2B3NB	70 - 74	70	E-->T
2B3NB	70 - 74	71	D-->C
2B3NB	70 - 74	71	D-->G
2B3NB	70 - 74	71	D-->K
2B3NB	70 - 74	71	D-->N
2B3NB	70 - 74	71	D-->P
2B3NB	70 - 74	71	D-->S
2B3NB	70 - 74	71	D-->T
2B3NB	70 - 74	71	D-->W
2B3NB	70 - 74	72	F-->A
2B3NB	70 - 74	72	F-->D
2B3NB	70 - 74	72	F-->H
2B3NB	70 - 74	72	F-->P
2B3NB	70 - 74	72	F-->R
2B3NB	70 - 74	72	F-->Y
2B3NB	70 - 74	73	A-->F
2B3NB	70 - 74	73	A-->P
2B3NB	70 - 74	73	A-->R
2B3NB	70 - 74	73	A-->V
2B3NB	70 - 74	74	S-->E
2B3NB	70 - 74	74	S-->F
2B3NB	70 - 74	74	S-->I
2B3NB	70 - 74	74	S-->L
2B3NB	70 - 74	74	S-->M
2B3NB	70 - 74	74	S-->R
2B3NB	70 - 74	74	S-->T
2B3NB	70 - 74	74	S-->V

2B3NB	70	- 74	74	S-->Y
2VBKA	152	- 156	153	H-->V
2VBKA	152	- 156	154	D-->T
2VBKA	152	- 156	155	K-->D
2VBKA	152	- 156	155	K-->I
2VBKA	152	- 156	155	K-->L
2VBKA	152	- 156	155	K-->S
2VBKA	152	- 156	155	K-->T
2VBKA	152	- 156	156	N-->D
2VBKA	152	- 156	156	N-->G
2VBKA	152	- 156	156	N-->I
2VBKA	152	- 156	156	N-->L
2VBKA	152	- 156	156	N-->T
2VBKA	152	- 156	156	N-->V
1MF7A	195	- 199	195	P-->A
1MF7A	195	- 199	195	P-->D
1MF7A	195	- 199	195	P-->F
1MF7A	195	- 199	195	P-->G
1MF7A	195	- 199	195	P-->H
1MF7A	195	- 199	195	P-->L
1MF7A	195	- 199	195	P-->R
1MF7A	195	- 199	196	R-->A
1MF7A	195	- 199	196	R-->E
1MF7A	195	- 199	196	R-->G
1MF7A	195	- 199	196	R-->H
1MF7A	195	- 199	196	R-->I
1MF7A	195	- 199	196	R-->K

1MF7A	195 - 199	196	R-->L
1MF7A	195 - 199	196	R-->P
1MF7A	195 - 199	196	R-->V
1MF7A	195 - 199	196	R-->W
1MF7A	195 - 199	196	R-->Y
1MF7A	195 - 199	197	S-->D
1MF7A	195 - 199	197	S-->E
1MF7A	195 - 199	197	S-->F
1MF7A	195 - 199	197	S-->G
1MF7A	195 - 199	197	S-->I
1MF7A	195 - 199	197	S-->K
1MF7A	195 - 199	197	S-->L
1MF7A	195 - 199	197	S-->P
1MF7A	195 - 199	197	S-->R
1MF7A	195 - 199	197	S-->T
1MF7A	195 - 199	197	S-->V
1MF7A	195 - 199	198	L-->D
1MF7A	195 - 199	198	L-->K
1MF7A	195 - 199	198	L-->N
1MF7A	195 - 199	198	L-->R
1MF7A	195 - 199	198	L-->S
1MF7A	195 - 199	198	L-->T
1MF7A	195 - 199	198	L-->V
1MF7A	195 - 199	198	L-->Y
1MF7A	195 - 199	199	V-->A
1MF7A	195 - 199	199	V-->D
1MF7A	195 - 199	199	V-->E

1MF7A	195 - 199	199	V-->I
1MF7A	195 - 199	199	V-->K
1MF7A	195 - 199	199	V-->Y
1MF7A	256 - 261	256	I-->P
1MF7A	256 - 261	257	P-->T
1MF7A	256 - 261	260	D-->P
1MF7A	256 - 261	261	R-->N
1MF7A	256 - 261	261	R-->V
1MF7A	256 - 261	261	R-->Y
3BHYA	266 - 271	266	I-->S
3C8LA	41 - 45	41	V-->E
3C8LA	41 - 45	41	V-->G
3C8LA	41 - 45	41	V-->K
3C8LA	41 - 45	41	V-->L
3C8LA	41 - 45	41	V-->R
3C8LA	41 - 45	41	V-->W
3C8LA	41 - 45	42	W-->A
3C8LA	41 - 45	42	W-->E
3C8LA	41 - 45	42	W-->G
3C8LA	41 - 45	42	W-->K
3C8LA	41 - 45	42	W-->L
3C8LA	41 - 45	42	W-->M
3C8LA	41 - 45	42	W-->P
3C8LA	41 - 45	42	W-->Q
3C8LA	41 - 45	42	W-->R
3C8LA	41 - 45	42	W-->S
3C8LA	41 - 45	42	W-->Y

3C8LA	41	45	43	E-->A
3C8LA	41	45	43	E-->D
3C8LA	41	45	43	E-->G
3C8LA	41	45	43	E-->P
3C8LA	41	45	43	E-->T
3C8LA	41	45	44	V-->H
3C8LA	41	45	45	A-->H
3C8LA	41	45	45	A-->K
3C8LA	41	45	45	A-->P
3C8LA	41	45	45	A-->W
3C8LA	69	73	69	E-->D
3C8LA	69	73	69	E-->G
3C8LA	69	73	69	E-->H
3C8LA	69	73	69	E-->K
3C8LA	69	73	69	E-->L
3C8LA	69	73	69	E-->N
3C8LA	69	73	69	E-->P
3C8LA	69	73	69	E-->Q
3C8LA	69	73	69	E-->R
3C8LA	69	73	69	E-->T
3C8LA	69	73	69	E-->Y
3C8LA	69	73	70	E-->A
3C8LA	69	73	70	E-->C
3C8LA	69	73	70	E-->D
3C8LA	69	73	70	E-->F
3C8LA	69	73	70	E-->G
3C8LA	69	73	70	E-->I

3C8LA	69 - 73	70	E-->L
3C8LA	69 - 73	70	E-->P
3C8LA	69 - 73	70	E-->R
3C8LA	69 - 73	70	E-->T
3C8LA	69 - 73	70	E-->V
3C8LA	69 - 73	70	E-->Y
3C8LA	69 - 73	71	E-->A
3C8LA	69 - 73	71	E-->G
3C8LA	69 - 73	71	E-->H
3C8LA	69 - 73	71	E-->I
3C8LA	69 - 73	71	E-->L
3C8LA	69 - 73	71	E-->M
3C8LA	69 - 73	71	E-->P
3C8LA	69 - 73	71	E-->R
3C8LA	69 - 73	71	E-->S
3C8LA	69 - 73	71	E-->T
3C8LA	69 - 73	71	E-->V
3C8LA	69 - 73	72	V-->A
3C8LA	69 - 73	72	V-->E
3C8LA	69 - 73	72	V-->F
3C8LA	69 - 73	72	V-->G
3C8LA	69 - 73	72	V-->H
3C8LA	69 - 73	72	V-->K
3C8LA	69 - 73	72	V-->L
3C8LA	69 - 73	72	V-->P
3C8LA	69 - 73	72	V-->Q
3C8LA	69 - 73	72	V-->R

3C8LA	69 - 73	72	V-->S
3C8LA	69 - 73	72	V-->T
3C8LA	69 - 73	73	L-->E
3C8LA	69 - 73	73	L-->F
3C8LA	69 - 73	73	L-->I
3C8LA	69 - 73	73	L-->T
3C8LA	69 - 73	73	L-->V
3C8LA	69 - 73	73	L-->W
1KYFA	760 - 765	760	D-->R
1KYFA	760 - 765	761	D-->G
1MJUL	183 - 187	183	K-->A
1MJUL	183 - 187	183	K-->D
1MJUL	183 - 187	183	K-->E
1MJUL	183 - 187	183	K-->F
1MJUL	183 - 187	183	K-->G
1MJUL	183 - 187	183	K-->R
1MJUL	183 - 187	183	K-->V
1MJUL	183 - 187	184	D-->A
1MJUL	183 - 187	184	D-->E
1MJUL	183 - 187	184	D-->F
1MJUL	183 - 187	184	D-->G
1MJUL	183 - 187	184	D-->K
1MJUL	183 - 187	184	D-->M
1MJUL	183 - 187	184	D-->R
1MJUL	183 - 187	184	D-->W
1MJUL	183 - 187	185	E-->H
1MJUL	183 - 187	185	E-->L

1MJUL	183 - 187	185	E-->T	
1MJUL	183 - 187	186	Y-->G	
1MJUL	183 - 187	186	Y-->I	
1MJUL	183 - 187	186	Y-->L	
1MJUL	183 - 187	186	Y-->P	
1MJUL	183 - 187	187	E-->A	
1MJUL	183 - 187	187	E-->D	
1MJUL	183 - 187	187	E-->Y	
2QCFA	435 - 440	435	P-->N	
2QCFA	435 - 440	435	P-->T	
2QCFA	435 - 440	436	Q-->H	
1D4OA	31 - 35	31	Y-->A	
1D4OA	31 - 35	31	Y-->C	
1D4OA	31 - 35	31	Y-->L	
1D4OA	31 - 35	31	Y-->V	
1D4OA	31 - 35	32	G-->K	
1D4OA	31 - 35	32	G-->N	
1D4OA	31 - 35	32	G-->Q	
1D4OA	31 - 35	33	L-->G	
1D4OA	31 - 35	33	L-->N	
1D4OA	31 - 35	33	L-->R	
1D4OA	31 - 35	34	C-->A	
1D4OA	31 - 35	34	C-->D	
1D4OA	31 - 35	34	C-->E	
1D4OA	31 - 35	34	C-->G	
1D4OA	31 - 35	34	C-->H	
1D4OA	31 - 35	34	C-->I	

1D4OA	31 - 35	34	C-->K
1D4OA	31 - 35	34	C-->N
1D4OA	31 - 35	34	C-->P
1D4OA	31 - 35	34	C-->Q
1D4OA	31 - 35	34	C-->R
1D4OA	31 - 35	34	C-->S
1D4OA	31 - 35	34	C-->V
1D4OA	31 - 35	35	A-->E
1D4OA	31 - 35	35	A-->I
1E29A	56 - 60	56	L-->A
1E29A	56 - 60	56	L-->D
1E29A	56 - 60	56	L-->E
1E29A	56 - 60	56	L-->G
1E29A	56 - 60	56	L-->K
1E29A	56 - 60	56	L-->N
1E29A	56 - 60	56	L-->Q
1E29A	56 - 60	56	L-->R
1E29A	56 - 60	56	L-->T
1E29A	56 - 60	57	A-->D
1E29A	56 - 60	57	A-->E
1E29A	56 - 60	57	A-->F
1E29A	56 - 60	57	A-->G
1E29A	56 - 60	57	A-->H
1E29A	56 - 60	57	A-->I
1E29A	56 - 60	57	A-->K
1E29A	56 - 60	57	A-->L
1E29A	56 - 60	57	A-->N

1E29A	56 - 60	57	A-->P	
1E29A	56 - 60	57	A-->R	
1E29A	56 - 60	57	A-->T	
1E29A	56 - 60	57	A-->V	
1E29A	56 - 60	57	A-->W	
1E29A	56 - 60	57	A-->Y	
1E29A	56 - 60	58	D-->A	
1E29A	56 - 60	58	D-->F	
1E29A	56 - 60	58	D-->G	
1E29A	56 - 60	58	D-->L	
1E29A	56 - 60	58	D-->P	
1E29A	56 - 60	58	D-->R	
1E29A	56 - 60	58	D-->S	
1E29A	56 - 60	58	D-->T	
1E29A	56 - 60	58	D-->V	
1E29A	56 - 60	59	L-->A	
1E29A	56 - 60	59	L-->G	
1E29A	56 - 60	59	L-->I	
1E29A	56 - 60	59	L-->K	
1E29A	56 - 60	59	L-->P	
1E29A	56 - 60	59	L-->R	
1E29A	56 - 60	59	L-->T	
1E29A	56 - 60	59	L-->V	
1E29A	56 - 60	60	A-->D	
1E29A	56 - 60	60	A-->F	
1E29A	56 - 60	60	A-->G	
1E29A	56 - 60	60	A-->L	

1E29A	56 - 60	60	A-->N
1E29A	56 - 60	60	A-->P
1E29A	56 - 60	60	A-->Q
1E29A	56 - 60	60	A-->R
1E29A	56 - 60	60	A-->S
1E29A	56 - 60	60	A-->T
1E29A	56 - 60	60	A-->V
1E29A	56 - 60	60	A-->Y
1RYOA	209 - 213	209	T-->D
1RYOA	209 - 213	209	T-->E
1RYOA	209 - 213	209	T-->F
1RYOA	209 - 213	209	T-->I
1RYOA	209 - 213	209	T-->R
1RYOA	209 - 213	209	T-->S
1RYOA	209 - 213	209	T-->V
1RYOA	209 - 213	209	T-->Y
1RYOA	209 - 213	210	I-->D
1RYOA	209 - 213	210	I-->E
1RYOA	209 - 213	210	I-->L
1RYOA	209 - 213	210	I-->N
1RYOA	209 - 213	210	I-->Q
1RYOA	209 - 213	210	I-->V
1RYOA	209 - 213	210	I-->Y
1RYOA	209 - 213	211	F-->A
1RYOA	209 - 213	211	F-->D
1RYOA	209 - 213	211	F-->H
1RYOA	209 - 213	211	F-->I

1RYOA	209	213	211	F-->K
1RYOA	209	213	211	F-->N
1RYOA	209	213	211	F-->T
1RYOA	209	213	211	F-->V
1RYOA	209	213	211	F-->W
1RYOA	209	213	211	F-->Y
1RYOA	209	213	212	E-->D
1RYOA	209	213	212	E-->F
1RYOA	209	213	212	E-->K
1RYOA	209	213	212	E-->P
1RYOA	209	213	212	E-->T
1RYOA	209	213	213	N-->E
1RYOA	209	213	213	N-->K
1RYOA	235	240	236	D-->G
1RYOA	235	240	237	E-->S
1RYOA	311	315	311	A-->I
1RYOA	311	315	311	A-->M
1RYOA	311	315	312	K-->D
1RYOA	311	315	312	K-->F
1RYOA	311	315	312	K-->G
1RYOA	311	315	313	M-->A
1RYOA	311	315	313	M-->D
1RYOA	311	315	313	M-->G
1RYOA	311	315	313	M-->H
1RYOA	311	315	313	M-->I
1RYOA	311	315	313	M-->Q
1RYOA	311	315	314	Y-->G

1RYOA	311	- 315	314	Y-->S
1RYOA	311	- 315	314	Y-->V
1RYOA	311	- 315	315	L-->Y
2HHGA	13	- 17	13	L-->D
2HHGA	13	- 17	13	L-->F
2HHGA	13	- 17	13	L-->I
2HHGA	13	- 17	13	L-->P
2HHGA	13	- 17	13	L-->R
2HHGA	13	- 17	13	L-->S
2HHGA	13	- 17	13	L-->V
2HHGA	13	- 17	14	D-->F
2HHGA	13	- 17	14	D-->K
2HHGA	13	- 17	14	D-->L
2HHGA	13	- 17	14	D-->N
2HHGA	13	- 17	14	D-->P
2HHGA	13	- 17	14	D-->S
2HHGA	13	- 17	14	D-->V
2HHGA	13	- 17	14	D-->Y
2HHGA	13	- 17	15	E-->D
2HHGA	13	- 17	15	E-->L
2HHGA	13	- 17	15	E-->P
2HHGA	13	- 17	15	E-->R
2HHGA	13	- 17	15	E-->T
2HHGA	13	- 17	15	E-->V
2HHGA	13	- 17	16	A-->D
2HHGA	13	- 17	16	A-->E
2HHGA	13	- 17	16	A-->G

2HHGA	13 - 17	16	A-->H
2HHGA	13 - 17	16	A-->K
2HHGA	13 - 17	16	A-->L
2HHGA	13 - 17	16	A-->M
2HHGA	13 - 17	16	A-->N
2HHGA	13 - 17	16	A-->Q
2HHGA	13 - 17	16	A-->R
2HHGA	13 - 17	16	A-->V
2HHGA	13 - 17	17	N-->D
2HHGA	13 - 17	17	N-->F
2HHGA	13 - 17	17	N-->G
2HHGA	13 - 17	17	N-->H
2HHGA	13 - 17	17	N-->I
2HHGA	13 - 17	17	N-->K
2HHGA	13 - 17	17	N-->P
2HHGA	13 - 17	17	N-->Q
2HHGA	13 - 17	17	N-->R
2HHGA	13 - 17	17	N-->S
2HHGA	13 - 17	17	N-->T
2HHGA	13 - 17	17	N-->Y
2HHGA	67 - 71	67	L-->A
2HHGA	67 - 71	67	L-->K
2HHGA	67 - 71	69	F-->H
2HHGA	67 - 71	69	F-->K
2HHGA	67 - 71	69	F-->P
2HHGA	67 - 71	70	W-->A
2HHGA	67 - 71	70	W-->D

2HHGA	67 - 71	70	W-->H
2HHGA	67 - 71	70	W-->L
2HHGA	67 - 71	70	W-->S
2HHGA	67 - 71	70	W-->T
2HHGA	67 - 71	71	I-->D
2HHGA	67 - 71	71	I-->G
1OBOA	1041 - 1046	1041	V-->A
1OBOA	1041 - 1046	1042	T-->I
1OBOA	1041 - 1046	1043	D-->A
1OBOA	1041 - 1046	1043	D-->F
1OBOA	1041 - 1046	1044	L-->N
1OBOA	1041 - 1046	1044	L-->Q
1OBOA	1041 - 1046	1045	N-->A
1OBOA	1041 - 1046	1045	N-->D
1Z2NX	15 - 21	21	L-->K
1Z2NX	213 - 218	216	I-->E
1Z2NX	213 - 218	216	I-->Y
1Z2NX	213 - 218	218	S-->V
2C1VA	183 - 188	184	F-->R
2C1VA	183 - 188	187	F-->E
1R5YA	56 - 61	56	P-->S
1R5YA	56 - 61	58	T-->R
1R5YA	56 - 61	61	A-->E
1R5YA	80 - 85	81	E-->V
1R5YA	80 - 85	85	K-->D
1R5YA	80 - 85	85	K-->F
1R5YA	89 - 93	89	L-->N

1R5YA	89	93	90	H-->A
1R5YA	89	93	90	H-->D
1R5YA	89	93	90	H-->G
1R5YA	89	93	92	F-->Q
1R5YA	89	93	92	F-->R
1R5YA	89	93	93	M-->A
1R5YA	89	93	93	M-->Q
1R5YA	89	93	93	M-->T
1R5YA	89	93	93	M-->V
1R5YA	190	195	195	N-->S
1R5YA	283	289	286	R-->Y
1R5YA	321	325	321	A-->F
1R5YA	321	325	321	A-->L
1R5YA	321	325	321	A-->Q
1R5YA	321	325	323	C-->F
1R5YA	321	325	323	C-->M
1R5YA	321	325	323	C-->P
1R5YA	321	325	323	C-->R
1R5YA	321	325	324	Q-->D
1R5YA	321	325	324	Q-->F
1R5YA	321	325	324	Q-->P
1R5YA	321	325	325	K-->D
1R5YA	321	325	325	K-->M
1R5YA	321	325	325	K-->N
1R5YA	321	325	325	K-->V
1TU9A	33	38	38	K-->P
3BUUA	131	135	131	N-->A

3BUUA	131 - 135	131	N-->D
3BUUA	131 - 135	131	N-->E
3BUUA	131 - 135	131	N-->F
3BUUA	131 - 135	131	N-->G
3BUUA	131 - 135	131	N-->I
3BUUA	131 - 135	131	N-->K
3BUUA	131 - 135	131	N-->P
3BUUA	131 - 135	131	N-->R
3BUUA	131 - 135	131	N-->S
3BUUA	131 - 135	131	N-->T
3BUUA	131 - 135	131	N-->V
3BUUA	131 - 135	132	G-->E
3BUUA	131 - 135	132	G-->H
3BUUA	131 - 135	132	G-->K
3BUUA	131 - 135	132	G-->L
3BUUA	131 - 135	132	G-->N
3BUUA	131 - 135	132	G-->P
3BUUA	131 - 135	132	G-->V
3BUUA	131 - 135	133	D-->A
3BUUA	131 - 135	133	D-->E
3BUUA	131 - 135	133	D-->G
3BUUA	131 - 135	133	D-->I
3BUUA	131 - 135	133	D-->P
3BUUA	131 - 135	133	D-->Q
3BUUA	131 - 135	133	D-->R
3BUUA	131 - 135	133	D-->T
3BUUA	131 - 135	133	D-->V

3BUUA	131	-	135	133	D-->W
3BUUA	131	-	135	133	D-->Y
3BUUA	131	-	135	134	I-->F
3BUUA	131	-	135	134	I-->K
3BUUA	131	-	135	134	I-->S
3BUUA	131	-	135	134	I-->T
3BUUA	131	-	135	134	I-->V
3BUUA	131	-	135	134	I-->W
3BUUA	131	-	135	135	A-->C
3BUUA	131	-	135	135	A-->I
3BUUA	131	-	135	135	A-->K
3BUUA	131	-	135	135	A-->L
3BUUA	131	-	135	135	A-->P
3BUUA	131	-	135	135	A-->R
3BUUA	131	-	135	135	A-->S
3BUUA	131	-	135	135	A-->T
3BUUA	131	-	135	135	A-->V
2BMOB	50	-	56	50	Y-->G
2BMOB	50	-	56	53	W-->D
1NWWB	40	-	46	46	Y-->V
1UCRA	68	-	73	68	A-->D
1UCRA	68	-	73	71	Q-->A
1UCRA	68	-	73	71	Q-->T
1UCRA	68	-	73	72	A-->L
1UCRA	68	-	73	73	A-->I
1Z0WA	568	-	573	573	D-->P
3B4UA	218	-	225	224	M-->V

1M15A	26 - 30	26	L-->A
1M15A	26 - 30	26	L-->D
1M15A	26 - 30	26	L-->E
1M15A	26 - 30	26	L-->R
1M15A	26 - 30	26	L-->S
1M15A	26 - 30	26	L-->T
1M15A	26 - 30	26	L-->Y
1M15A	26 - 30	27	L-->E
1M15A	26 - 30	27	L-->K
1M15A	26 - 30	27	L-->P
1M15A	26 - 30	27	L-->R
1M15A	26 - 30	27	L-->S
1M15A	26 - 30	28	K-->C
1M15A	26 - 30	28	K-->F
1M15A	26 - 30	28	K-->N
1M15A	26 - 30	28	K-->R
1M15A	26 - 30	28	K-->V
1M15A	26 - 30	29	K-->A
1M15A	26 - 30	29	K-->C
1M15A	26 - 30	29	K-->I
1M15A	26 - 30	29	K-->T
1M15A	26 - 30	30	H-->C
1M15A	26 - 30	30	H-->D
1M15A	26 - 30	30	H-->K
1M15A	26 - 30	30	H-->P
1M15A	26 - 30	30	H-->S
1M15A	26 - 30	30	H-->Y

1M15A	193 - 197	193	R-->G
1M15A	193 - 197	193	R-->H
1M15A	193 - 197	193	R-->N
1M15A	193 - 197	193	R-->S
1M15A	193 - 197	193	R-->V
1M15A	193 - 197	193	R-->Y
1M15A	193 - 197	194	F-->D
1M15A	193 - 197	194	F-->G
1M15A	193 - 197	194	F-->I
1M15A	193 - 197	194	F-->K
1M15A	193 - 197	194	F-->L
1M15A	193 - 197	194	F-->N
1M15A	193 - 197	194	F-->S
1M15A	193 - 197	194	F-->V
1M15A	193 - 197	194	F-->W
1M15A	193 - 197	195	L-->A
1M15A	193 - 197	195	L-->E
1M15A	193 - 197	195	L-->K
1M15A	193 - 197	195	L-->N
1M15A	193 - 197	195	L-->V
1M15A	193 - 197	196	Q-->A
1M15A	193 - 197	196	Q-->C
1M15A	193 - 197	196	Q-->F
1M15A	193 - 197	196	Q-->G
1M15A	193 - 197	196	Q-->I
1M15A	193 - 197	196	Q-->R
1M15A	193 - 197	196	Q-->T

1M15A	193 - 197	197	T-->I
1M15A	193 - 197	197	T-->L
1M15A	193 - 197	197	T-->P
1M15A	193 - 197	197	T-->Q
1M15A	288 - 292	288	P-->F
1M15A	288 - 292	288	P-->G
1M15A	288 - 292	288	P-->I
1M15A	288 - 292	288	P-->Q
1M15A	288 - 292	288	P-->R
1M15A	288 - 292	289	K-->A
1M15A	288 - 292	289	K-->G
1M15A	288 - 292	289	K-->I
1M15A	288 - 292	289	K-->L
1M15A	288 - 292	289	K-->R
1M15A	288 - 292	289	K-->S
1M15A	288 - 292	289	K-->V
1M15A	288 - 292	289	K-->W
1M15A	288 - 292	290	L-->A
1M15A	288 - 292	290	L-->F
1M15A	288 - 292	290	L-->G
1M15A	288 - 292	290	L-->Q
1M15A	288 - 292	291	A-->D
1M15A	288 - 292	291	A-->E
1M15A	288 - 292	291	A-->R
1M15A	288 - 292	291	A-->S
1M15A	288 - 292	291	A-->V
1M15A	288 - 292	292	K-->A

1M15A	288 - 292	292	K-->D
1M15A	288 - 292	292	K-->G
1M15A	288 - 292	292	K-->I
1M15A	288 - 292	292	K-->L
1M15A	288 - 292	292	K-->N
1M15A	288 - 292	292	K-->S
1M15A	288 - 292	292	K-->V
1VR7A	23 - 29	26	I-->D
2V3GA	76 - 80	76	T-->A
2V3GA	76 - 80	76	T-->C
2V3GA	76 - 80	76	T-->D
2V3GA	76 - 80	76	T-->E
2V3GA	76 - 80	76	T-->F
2V3GA	76 - 80	76	T-->I
2V3GA	76 - 80	76	T-->K
2V3GA	76 - 80	76	T-->P
2V3GA	76 - 80	76	T-->Q
2V3GA	76 - 80	76	T-->R
2V3GA	76 - 80	76	T-->S
2V3GA	76 - 80	76	T-->V
2V3GA	76 - 80	77	V-->C
2V3GA	76 - 80	77	V-->G
2V3GA	76 - 80	77	V-->P
2V3GA	76 - 80	77	V-->S
2V3GA	76 - 80	77	V-->Y
2V3GA	76 - 80	78	D-->A
2V3GA	76 - 80	78	D-->E

2V3GA	76 - 80	78	D-->F
2V3GA	76 - 80	78	D-->I
2V3GA	76 - 80	78	D-->K
2V3GA	76 - 80	78	D-->L
2V3GA	76 - 80	78	D-->N
2V3GA	76 - 80	78	D-->R
2V3GA	76 - 80	78	D-->S
2V3GA	76 - 80	78	D-->T
2V3GA	76 - 80	78	D-->V
2V3GA	76 - 80	79	I-->A
2V3GA	76 - 80	79	I-->D
2V3GA	76 - 80	79	I-->E
2V3GA	76 - 80	79	I-->G
2V3GA	76 - 80	79	I-->K
2V3GA	76 - 80	79	I-->N
2V3GA	76 - 80	79	I-->P
2V3GA	76 - 80	79	I-->Q
2V3GA	76 - 80	79	I-->R
2V3GA	76 - 80	79	I-->S
2V3GA	76 - 80	79	I-->T
2V3GA	76 - 80	79	I-->V
2V3GA	76 - 80	79	I-->W
2V3GA	76 - 80	80	K-->D
2V3GA	76 - 80	80	K-->E
2V3GA	76 - 80	80	K-->F
2V3GA	76 - 80	80	K-->G
2V3GA	76 - 80	80	K-->L

2V3GA	76	80	80	K-->N
2V3GA	76	80	80	K-->Q
2V3GA	76	80	80	K-->S
2V3GA	76	80	80	K-->Y
2BK9A	36	41	37	S-->P
2BK9A	36	41	38	N-->K
2BK9A	36	41	41	K-->G
2BK9A	49	54	49	L-->P
2BK9A	49	54	50	E-->D
2BK9A	49	54	50	E-->S
2BK9A	49	54	51	E-->F
2BK9A	49	54	51	E-->N
2BK9A	49	54	51	E-->S
2BK9A	49	54	53	S-->P
2BK9A	49	54	54	G-->E
2BK9A	49	54	54	G-->L
1UWKA	327	333	330	M-->G
1H97A	40	45	45	H-->V
2RFRA	36	40	36	A-->E
2RFRA	36	40	36	A-->G
2RFRA	36	40	36	A-->H
2RFRA	36	40	36	A-->I
2RFRA	36	40	36	A-->L
2RFRA	36	40	36	A-->P
2RFRA	36	40	36	A-->R
2RFRA	36	40	36	A-->V
2RFRA	36	40	36	A-->W

2RFRA	36 - 40	36	A-->Y
2RFRA	36 - 40	37	E-->A
2RFRA	36 - 40	37	E-->C
2RFRA	36 - 40	37	E-->D
2RFRA	36 - 40	37	E-->F
2RFRA	36 - 40	37	E-->H
2RFRA	36 - 40	37	E-->I
2RFRA	36 - 40	37	E-->K
2RFRA	36 - 40	37	E-->L
2RFRA	36 - 40	37	E-->M
2RFRA	36 - 40	37	E-->P
2RFRA	36 - 40	37	E-->R
2RFRA	36 - 40	37	E-->T
2RFRA	36 - 40	37	E-->V
2RFRA	36 - 40	37	E-->Y
2RFRA	36 - 40	38	A-->C
2RFRA	36 - 40	38	A-->D
2RFRA	36 - 40	38	A-->E
2RFRA	36 - 40	38	A-->G
2RFRA	36 - 40	38	A-->I
2RFRA	36 - 40	38	A-->K
2RFRA	36 - 40	38	A-->M
2RFRA	36 - 40	38	A-->N
2RFRA	36 - 40	38	A-->P
2RFRA	36 - 40	38	A-->Q
2RFRA	36 - 40	38	A-->R
2RFRA	36 - 40	38	A-->T

2RFRA	36	40	38	A-->V
2RFRA	36	40	38	A-->W
2RFRA	36	40	39	L-->F
2RFRA	36	40	39	L-->H
2RFRA	36	40	39	L-->K
2RFRA	36	40	39	L-->P
2RFRA	36	40	39	L-->Q
2RFRA	36	40	39	L-->S
2RFRA	36	40	39	L-->T
2RFRA	36	40	39	L-->V
2RFRA	36	40	39	L-->Y
2RFRA	36	40	40	S-->D
2RFRA	36	40	40	S-->E
2RFRA	36	40	40	S-->G
2RFRA	36	40	40	S-->H
2RFRA	36	40	40	S-->K
2RFRA	36	40	40	S-->L
2RFRA	36	40	40	S-->M
2RFRA	36	40	40	S-->P
2RFRA	145	151	149	A-->V
1M1NA	63	67	63	A-->D
1M1NA	63	67	63	A-->E
1M1NA	63	67	63	A-->G
1M1NA	63	67	63	A-->I
1M1NA	63	67	63	A-->K
1M1NA	63	67	63	A-->M
1M1NA	63	67	63	A-->S

1M1NA	63 - 67	63	A-->V
1M1NA	63 - 67	64	Y-->A
1M1NA	63 - 67	64	Y-->C
1M1NA	63 - 67	64	Y-->F
1M1NA	63 - 67	64	Y-->G
1M1NA	63 - 67	64	Y-->H
1M1NA	63 - 67	64	Y-->I
1M1NA	63 - 67	64	Y-->L
1M1NA	63 - 67	64	Y-->N
1M1NA	63 - 67	64	Y-->P
1M1NA	63 - 67	64	Y-->R
1M1NA	63 - 67	64	Y-->S
1M1NA	63 - 67	64	Y-->T
1M1NA	63 - 67	64	Y-->V
1M1NA	63 - 67	65	A-->D
1M1NA	63 - 67	65	A-->E
1M1NA	63 - 67	65	A-->G
1M1NA	63 - 67	65	A-->K
1M1NA	63 - 67	65	A-->L
1M1NA	63 - 67	65	A-->Q
1M1NA	63 - 67	65	A-->R
1M1NA	63 - 67	65	A-->S
1M1NA	63 - 67	65	A-->T
1M1NA	63 - 67	65	A-->V
1M1NA	63 - 67	66	G-->D
1M1NA	63 - 67	66	G-->K
1M1NA	63 - 67	66	G-->L

1M1NA	63 - 67	66	G-->N
1M1NA	63 - 67	66	G-->P
1M1NA	63 - 67	66	G-->Q
1M1NA	63 - 67	66	G-->W
1M1NA	63 - 67	67	S-->D
1M1NA	63 - 67	67	S-->F
1M1NA	63 - 67	67	S-->K
1M1NA	63 - 67	67	S-->L
1M1NA	63 - 67	67	S-->Q
1M1NA	63 - 67	67	S-->Y
1M1NA	87 - 91	87	G-->L
1M1NA	87 - 91	88	C-->A
1M1NA	87 - 91	88	C-->D
1M1NA	87 - 91	88	C-->E
1M1NA	87 - 91	88	C-->F
1M1NA	87 - 91	88	C-->G
1M1NA	87 - 91	88	C-->K
1M1NA	87 - 91	88	C-->N
1M1NA	87 - 91	88	C-->P
1M1NA	87 - 91	88	C-->R
1M1NA	87 - 91	89	G-->L
1M1NA	87 - 91	90	Q-->G
1M1NA	87 - 91	90	Q-->L
1M1NA	87 - 91	90	Q-->V
1M1NA	87 - 91	91	Y-->K
1M1NA	87 - 91	91	Y-->R
1M1NA	120 - 125	120	E-->G

1M1NA	120 - 125	121	K-->Q
1M1NA	120 - 125	121	K-->S
1M1NA	120 - 125	124	V-->E
1M1NA	120 - 125	125	F-->A
1M1NA	120 - 125	125	F-->G
1M1NA	120 - 125	125	F-->L
1M1NA	120 - 125	125	F-->T
1M1NA	155 - 159	155	P-->E
1M1NA	155 - 159	155	P-->G
1M1NA	155 - 159	155	P-->H
1M1NA	155 - 159	155	P-->K
1M1NA	155 - 159	155	P-->L
1M1NA	155 - 159	155	P-->N
1M1NA	155 - 159	155	P-->S
1M1NA	155 - 159	155	P-->V
1M1NA	155 - 159	155	P-->W
1M1NA	155 - 159	156	I-->A
1M1NA	155 - 159	156	I-->D
1M1NA	155 - 159	156	I-->G
1M1NA	155 - 159	156	I-->H
1M1NA	155 - 159	156	I-->N
1M1NA	155 - 159	156	I-->S
1M1NA	155 - 159	156	I-->T
1M1NA	155 - 159	156	I-->V
1M1NA	155 - 159	157	G-->D
1M1NA	155 - 159	157	G-->I
1M1NA	155 - 159	157	G-->K

1M1NA	155 - 159	157	G-->L
1M1NA	155 - 159	157	G-->N
1M1NA	155 - 159	157	G-->R
1M1NA	155 - 159	157	G-->T
1M1NA	155 - 159	157	G-->V
1M1NA	155 - 159	157	G-->Y
1M1NA	155 - 159	158	L-->A
1M1NA	155 - 159	158	L-->E
1M1NA	155 - 159	158	L-->G
1M1NA	155 - 159	158	L-->I
1M1NA	155 - 159	158	L-->P
1M1NA	155 - 159	158	L-->R
1M1NA	155 - 159	158	L-->T
1M1NA	155 - 159	158	L-->Y
1M1NA	155 - 159	159	I-->A
1M1NA	155 - 159	159	I-->F
1M1NA	155 - 159	159	I-->G
1M1NA	155 - 159	159	I-->L
1M1NA	155 - 159	159	I-->N
1M1NA	155 - 159	159	I-->P
1M1NA	155 - 159	159	I-->R
1M1NA	155 - 159	159	I-->S
1M1NA	155 - 159	159	I-->Y
1M1NA	364 - 368	364	I-->E
1M1NA	364 - 368	364	I-->Q
1M1NA	364 - 368	364	I-->S
1M1NA	364 - 368	365	G-->I

1M1NA	364	368	365	G-->L
1M1NA	364	368	365	G-->M
1M1NA	364	368	365	G-->P
1M1NA	364	368	365	G-->T
1M1NA	364	368	366	A-->G
1M1NA	364	368	366	A-->L
1M1NA	364	368	366	A-->N
1M1NA	364	368	366	A-->P
1M1NA	364	368	366	A-->T
1M1NA	364	368	367	Y-->A
1M1NA	364	368	367	Y-->F
1M1NA	364	368	367	Y-->G
1M1NA	364	368	367	Y-->I
1M1NA	364	368	367	Y-->L
1M1NA	364	368	367	Y-->M
1M1NA	364	368	367	Y-->P
1M1NA	364	368	367	Y-->R
1M1NA	364	368	367	Y-->S
1M1NA	364	368	367	Y-->T
1M1NA	364	368	367	Y-->V
1M1NA	364	368	368	E-->C
1M1NA	364	368	368	E-->G
1M1NA	364	368	368	E-->L
1M1NA	364	368	368	E-->Q
1M1NA	364	368	368	E-->R
1M1NA	364	368	368	E-->S
1M1NA	364	368	368	E-->V

1M1NA	384 - 388	384	N-->A
1M1NA	384 - 388	384	N-->F
1M1NA	384 - 388	384	N-->G
1M1NA	384 - 388	384	N-->L
1M1NA	384 - 388	384	N-->W
1M1NA	384 - 388	384	N-->Y
1M1NA	384 - 388	385	D-->G
1M1NA	384 - 388	385	D-->I
1M1NA	384 - 388	385	D-->K
1M1NA	384 - 388	385	D-->Q
1M1NA	384 - 388	386	D-->A
1M1NA	384 - 388	386	D-->H
1M1NA	384 - 388	386	D-->L
1M1NA	384 - 388	386	D-->P
1M1NA	384 - 388	386	D-->R
1M1NA	384 - 388	386	D-->Y
1M1NA	384 - 388	387	Y-->D
1M1NA	384 - 388	387	Y-->N
1M1NA	384 - 388	387	Y-->R
1M1NA	384 - 388	388	D-->A
1M1NA	384 - 388	388	D-->F
1M1NA	384 - 388	388	D-->I
1M1NA	384 - 388	388	D-->L
1M1NA	384 - 388	388	D-->P
1M1NA	384 - 388	388	D-->R
1M1NA	384 - 388	388	D-->T
1M1NA	384 - 388	388	D-->V

1M1NB	120 - 125	120	E-->I
1M1NB	120 - 125	120	E-->L
1M1NB	120 - 125	120	E-->S
1M1NB	120 - 125	122	A-->T
1M1NB	120 - 125	122	A-->V
1M1NB	120 - 125	125	F-->Y
2P86A	142 - 146	142	T-->A
2P86A	142 - 146	142	T-->G
2P86A	142 - 146	142	T-->L
2P86A	142 - 146	142	T-->R
2P86A	142 - 146	142	T-->Y
2P86A	142 - 146	143	S-->G
2P86A	142 - 146	143	S-->V
2P86A	142 - 146	144	F-->E
2P86A	142 - 146	144	F-->H
2P86A	142 - 146	144	F-->T
2P86A	142 - 146	144	F-->W
2P86A	142 - 146	145	M-->A
2P86A	142 - 146	145	M-->D
2P86A	142 - 146	145	M-->E
2P86A	142 - 146	145	M-->F
2P86A	142 - 146	145	M-->R
2P86A	142 - 146	145	M-->S
2P86A	142 - 146	145	M-->T
2P86A	142 - 146	145	M-->V
2P86A	142 - 146	146	D-->H
2P86A	142 - 146	146	D-->T

2FHZA	15 - 20	18	F-->G	
2AWKA	4 - 8	4	G-->E	
2AWKA	4 - 8	4	G-->M	
2AWKA	4 - 8	4	G-->N	
2AWKA	4 - 8	4	G-->R	
2AWKA	4 - 8	5	E-->D	
2AWKA	4 - 8	5	E-->F	
2AWKA	4 - 8	5	E-->G	
2AWKA	4 - 8	5	E-->H	
2AWKA	4 - 8	5	E-->I	
2AWKA	4 - 8	5	E-->K	
2AWKA	4 - 8	5	E-->L	
2AWKA	4 - 8	5	E-->N	
2AWKA	4 - 8	5	E-->P	
2AWKA	4 - 8	5	E-->T	
2AWKA	4 - 8	5	E-->V	
2AWKA	4 - 8	5	E-->W	
2AWKA	4 - 8	6	E-->A	
2AWKA	4 - 8	6	E-->D	
2AWKA	4 - 8	6	E-->G	
2AWKA	4 - 8	6	E-->K	
2AWKA	4 - 8	6	E-->L	
2AWKA	4 - 8	6	E-->M	
2AWKA	4 - 8	6	E-->N	
2AWKA	4 - 8	6	E-->P	
2AWKA	4 - 8	6	E-->Q	
2AWKA	4 - 8	6	E-->R	

2AWKA	4 -	8	7	L-->F
2AWKA	4 -	8	7	L-->I
2AWKA	4 -	8	7	L-->K
2AWKA	4 -	8	7	L-->N
2AWKA	4 -	8	7	L-->R
2AWKA	4 -	8	7	L-->S
2AWKA	4 -	8	7	L-->T
2AWKA	4 -	8	7	L-->V
2AWKA	4 -	8	8	F-->C
2AWKA	4 -	8	8	F-->E
2AWKA	4 -	8	8	F-->G
2AWKA	4 -	8	8	F-->H
2AWKA	4 -	8	8	F-->I
2AWKA	4 -	8	8	F-->K
2AWKA	4 -	8	8	F-->L
2AWKA	4 -	8	8	F-->N
2AWKA	4 -	8	8	F-->P
2AWKA	4 -	8	8	F-->Q
2AWKA	4 -	8	8	F-->R
2AWKA	4 -	8	8	F-->S
2AWKA	4 -	8	8	F-->T
2AWKA	4 -	8	8	F-->V
2AWKA	4 -	8	8	F-->Y
1K8UA	51 -	56	52	E-->G
1K8UA	51 -	56	52	E-->L
1K8UA	51 -	56	52	E-->V
1K8UA	51 -	56	53	I-->N

1K8UA	51	56	55	R-->A
1K8UA	51	56	55	R-->P
1K8UA	58	62	58	E-->G
1K8UA	58	62	58	E-->I
1K8UA	58	62	58	E-->K
1K8UA	58	62	58	E-->R
1K8UA	58	62	58	E-->V
1K8UA	58	62	59	D-->G
1K8UA	58	62	59	D-->I
1K8UA	58	62	59	D-->K
1K8UA	58	62	59	D-->N
1K8UA	58	62	59	D-->P
1K8UA	58	62	59	D-->Q
1K8UA	58	62	60	L-->A
1K8UA	58	62	60	L-->D
1K8UA	58	62	60	L-->E
1K8UA	58	62	60	L-->G
1K8UA	58	62	60	L-->T
1K8UA	58	62	60	L-->Y
1K8UA	58	62	61	D-->E
1K8UA	58	62	61	D-->G
1K8UA	58	62	61	D-->K
1K8UA	58	62	61	D-->M
1K8UA	58	62	61	D-->P
1K8UA	58	62	61	D-->R
1K8UA	58	62	61	D-->S
1K8UA	58	62	61	D-->V

1K8UA	58 - 62	62	R-->A
1K8UA	58 - 62	62	R-->D
1K8UA	58 - 62	62	R-->I
1K8UA	58 - 62	62	R-->L
1K8UA	58 - 62	62	R-->M
1K8UA	58 - 62	62	R-->V
1XMTA	77 - 81	77	S-->A
1XMTA	77 - 81	77	S-->D
1XMTA	77 - 81	77	S-->E
1XMTA	77 - 81	77	S-->F
1XMTA	77 - 81	77	S-->I
1XMTA	77 - 81	77	S-->L
1XMTA	77 - 81	77	S-->V
1XMTA	77 - 81	77	S-->Y
1XMTA	77 - 81	78	Y-->C
1XMTA	77 - 81	78	Y-->E
1XMTA	77 - 81	78	Y-->G
1XMTA	77 - 81	78	Y-->I
1XMTA	77 - 81	78	Y-->K
1XMTA	77 - 81	78	Y-->L
1XMTA	77 - 81	78	Y-->M
1XMTA	77 - 81	78	Y-->P
1XMTA	77 - 81	78	Y-->Q
1XMTA	77 - 81	78	Y-->R
1XMTA	77 - 81	78	Y-->T
1XMTA	77 - 81	79	V-->E
1XMTA	77 - 81	79	V-->F

1XMTA	77 - 81	79	V-->L
1XMTA	77 - 81	79	V-->P
1XMTA	77 - 81	79	V-->Q
1XMTA	77 - 81	79	V-->R
1XMTA	77 - 81	79	V-->S
1XMTA	77 - 81	80	S-->A
1XMTA	77 - 81	80	S-->D
1XMTA	77 - 81	80	S-->G
1XMTA	77 - 81	80	S-->I
1XMTA	77 - 81	80	S-->L
1XMTA	77 - 81	80	S-->P
1XMTA	77 - 81	80	S-->R
1XMTA	77 - 81	81	D-->F
1XMTA	77 - 81	81	D-->K
1XMTA	77 - 81	81	D-->L
1XMTA	77 - 81	81	D-->T
1XMTA	77 - 81	81	D-->V
1XMTA	88 - 93	89	S-->R
1XMTA	88 - 93	90	W-->L
1ODMA	255 - 260	255	S-->P
1ODMA	255 - 260	258	A-->P
1Z70X	1148 - 1152	1148	E-->G
1Z70X	1148 - 1152	1148	E-->K
1Z70X	1148 - 1152	1148	E-->N
1Z70X	1148 - 1152	1148	E-->S
1Z70X	1148 - 1152	1148	E-->V
1Z70X	1148 - 1152	1148	E-->Y

1Z70X	1148 - 1152	1149	A-->F
1Z70X	1148 - 1152	1149	A-->K
1Z70X	1148 - 1152	1149	A-->P
1Z70X	1148 - 1152	1149	A-->Q
1Z70X	1148 - 1152	1149	A-->Y
1Z70X	1148 - 1152	1150	E-->I
1Z70X	1148 - 1152	1151	K-->C
1Z70X	1148 - 1152	1151	K-->E
1Z70X	1148 - 1152	1151	K-->F
1Z70X	1148 - 1152	1151	K-->G
1Z70X	1148 - 1152	1151	K-->R
1Z70X	1148 - 1152	1152	F-->D
1Z70X	1148 - 1152	1152	F-->G
1Z70X	1148 - 1152	1152	F-->L
1Z70X	1148 - 1152	1152	F-->N
1Z70X	1148 - 1152	1152	F-->Q
1Z70X	1148 - 1152	1152	F-->T
2NSZA	441 - 445	441	K-->C
2NSZA	441 - 445	441	K-->H
2NSZA	441 - 445	441	K-->S
2NSZA	441 - 445	441	K-->Y
2NSZA	441 - 445	442	Q-->A
2NSZA	441 - 445	442	Q-->S
2NSZA	441 - 445	442	Q-->V
2NSZA	441 - 445	442	Q-->W
2NSZA	441 - 445	443	L-->N
2NSZA	441 - 445	443	L-->T

2NSZA	441	445	443	L-->V
2NSZA	441	445	444	R-->A
2NSZA	441	445	444	R-->E
2NSZA	441	445	444	R-->N
2NSZA	441	445	444	R-->P
2NSZA	441	445	444	R-->V
2NSZA	441	445	445	D-->A
2NSZA	441	445	445	D-->F
2NSZA	441	445	445	D-->I
2NSZA	441	445	445	D-->K
2NSZA	441	445	445	D-->L
2UUYB	66	70	66	M-->A
2UUYB	66	70	66	M-->E
2UUYB	66	70	66	M-->F
2UUYB	66	70	66	M-->H
2UUYB	66	70	66	M-->K
2UUYB	66	70	66	M-->N
2UUYB	66	70	66	M-->R
2UUYB	66	70	68	E-->N
2UUYB	66	70	69	C-->G
2UUYB	66	70	69	C-->L
2UUYB	66	70	69	C-->S
2UUYB	66	70	69	C-->T
2UUYB	66	70	69	C-->W
2UUYB	66	70	70	A-->Y
3CLMA	21	25	21	R-->A
3CLMA	21	25	21	R-->G

3CLMA	21 - 25	21	R-->V
3CLMA	21 - 25	22	S-->A
3CLMA	21 - 25	22	S-->F
3CLMA	21 - 25	22	S-->K
3CLMA	21 - 25	22	S-->L
3CLMA	21 - 25	22	S-->N
3CLMA	21 - 25	22	S-->P
3CLMA	21 - 25	22	S-->T
3CLMA	21 - 25	22	S-->V
3CLMA	21 - 25	22	S-->Y
3CLMA	21 - 25	23	L-->D
3CLMA	21 - 25	23	L-->G
3CLMA	21 - 25	23	L-->N
3CLMA	21 - 25	23	L-->P
3CLMA	21 - 25	23	L-->Q
3CLMA	21 - 25	24	V-->A
3CLMA	21 - 25	24	V-->G
3CLMA	21 - 25	24	V-->K
3CLMA	21 - 25	24	V-->R
3CLMA	21 - 25	24	V-->W
3CLMA	21 - 25	25	Q-->E
3CLMA	21 - 25	25	Q-->I
3CLMA	21 - 25	25	Q-->L
3CLMA	21 - 25	25	Q-->N
3CLMA	21 - 25	25	Q-->R
3CLMA	21 - 25	25	Q-->S
3CLMA	21 - 25	25	Q-->V

3CLMA	271 - 275	271	L-->A
3CLMA	271 - 275	271	L-->D
3CLMA	271 - 275	271	L-->E
3CLMA	271 - 275	271	L-->G
3CLMA	271 - 275	271	L-->H
3CLMA	271 - 275	271	L-->I
3CLMA	271 - 275	271	L-->P
3CLMA	271 - 275	271	L-->Q
3CLMA	271 - 275	271	L-->S
3CLMA	271 - 275	271	L-->Y
3CLMA	271 - 275	272	Y-->D
3CLMA	271 - 275	272	Y-->G
3CLMA	271 - 275	272	Y-->H
3CLMA	271 - 275	272	Y-->I
3CLMA	271 - 275	272	Y-->L
3CLMA	271 - 275	272	Y-->N
3CLMA	271 - 275	272	Y-->P
3CLMA	271 - 275	272	Y-->Q
3CLMA	271 - 275	272	Y-->S
3CLMA	271 - 275	272	Y-->T
3CLMA	271 - 275	272	Y-->V
3CLMA	271 - 275	272	Y-->W
3CLMA	271 - 275	273	V-->A
3CLMA	271 - 275	273	V-->F
3CLMA	271 - 275	273	V-->H
3CLMA	271 - 275	273	V-->I
3CLMA	271 - 275	273	V-->Q

3CLMA	271 - 275	273	V-->T
3CLMA	271 - 275	273	V-->Y
3CLMA	271 - 275	274	D-->A
3CLMA	271 - 275	274	D-->E
3CLMA	271 - 275	274	D-->F
3CLMA	271 - 275	274	D-->G
3CLMA	271 - 275	274	D-->H
3CLMA	271 - 275	274	D-->I
3CLMA	271 - 275	274	D-->M
3CLMA	271 - 275	274	D-->N
3CLMA	271 - 275	274	D-->P
3CLMA	271 - 275	274	D-->S
3CLMA	271 - 275	274	D-->T
3CLMA	271 - 275	274	D-->V
3CLMA	271 - 275	275	S-->A
3CLMA	271 - 275	275	S-->D
3CLMA	271 - 275	275	S-->E
3CLMA	271 - 275	275	S-->F
3CLMA	271 - 275	275	S-->G
3CLMA	271 - 275	275	S-->H
3CLMA	271 - 275	275	S-->I
3CLMA	271 - 275	275	S-->L
3CLMA	271 - 275	275	S-->P
3CLMA	271 - 275	275	S-->R
3CLMA	271 - 275	275	S-->T
1U07B	165 - 170	166	R-->G
1U07B	165 - 170	166	R-->V

1U07B	165	- 170	167	A-->E
1U07B	165	- 170	167	A-->G
1U07B	165	- 170	168	Q-->A
1U07B	165	- 170	170	L-->R
1U07B	165	- 170	170	L-->S
1JF8A	69	- 74	69	N-->E
1JF8A	83	- 88	83	S-->V
1JF8A	83	- 88	86	D-->Y
2EABA	423	- 427	423	N-->C
2EABA	423	- 427	423	N-->F
2EABA	423	- 427	423	N-->I
2EABA	423	- 427	423	N-->Y
2EABA	423	- 427	424	L-->F
2EABA	423	- 427	425	Q-->S
2EABA	423	- 427	426	M-->G
2EABA	423	- 427	426	M-->I
2EABA	423	- 427	426	M-->L
2EABA	423	- 427	426	M-->T
2EABA	423	- 427	427	N-->D
2EABA	423	- 427	427	N-->S
1RA0A	248	- 252	248	T-->G
1RA0A	248	- 252	250	M-->D
1RA0A	248	- 252	250	M-->P
1RA0A	248	- 252	250	M-->R
1RA0A	248	- 252	250	M-->W
1RA0A	248	- 252	251	H-->R
1RA0A	248	- 252	252	S-->H

1RA0A	298 - 303	298	V-->G
1RA0A	298 - 303	301	M-->L
1RA0A	298 - 303	301	M-->R
1RA0A	384 - 390	387	A-->I
1LU4A	1090 - 1094	1090	V-->K
1LU4A	1090 - 1094	1090	V-->R
1LU4A	1090 - 1094	1091	I-->K
1LU4A	1090 - 1094	1091	I-->R
1LU4A	1090 - 1094	1091	I-->V
1LU4A	1090 - 1094	1092	W-->D
1LU4A	1090 - 1094	1092	W-->E
1LU4A	1090 - 1094	1092	W-->F
1LU4A	1090 - 1094	1092	W-->K
1LU4A	1090 - 1094	1092	W-->L
1LU4A	1090 - 1094	1092	W-->M
1LU4A	1090 - 1094	1092	W-->N
1LU4A	1090 - 1094	1092	W-->P
1LU4A	1090 - 1094	1092	W-->S
1LU4A	1090 - 1094	1092	W-->V
1LU4A	1090 - 1094	1093	A-->L
1LU4A	1090 - 1094	1093	A-->M
1LU4A	1090 - 1094	1094	R-->A
1LU4A	1090 - 1094	1094	R-->E
1LU4A	1090 - 1094	1094	R-->G
1LU4A	1090 - 1094	1094	R-->I
1LU4A	1090 - 1094	1094	R-->N
1LU4A	1090 - 1094	1094	R-->S

2GZVA	57 - 61	57	P-->D
2GZVA	57 - 61	57	P-->E
2GZVA	57 - 61	57	P-->F
2GZVA	57 - 61	57	P-->G
2GZVA	57 - 61	57	P-->I
2GZVA	57 - 61	57	P-->R
2GZVA	57 - 61	57	P-->T
2GZVA	57 - 61	57	P-->V
2GZVA	57 - 61	58	A-->C
2GZVA	57 - 61	58	A-->D
2GZVA	57 - 61	58	A-->G
2GZVA	57 - 61	58	A-->K
2GZVA	57 - 61	58	A-->L
2GZVA	57 - 61	58	A-->M
2GZVA	57 - 61	58	A-->N
2GZVA	57 - 61	58	A-->Q
2GZVA	57 - 61	58	A-->V
2GZVA	57 - 61	58	A-->Y
2GZVA	57 - 61	59	A-->G
2GZVA	57 - 61	59	A-->L
2GZVA	57 - 61	59	A-->P
2GZVA	57 - 61	59	A-->Q
2GZVA	57 - 61	59	A-->R
2GZVA	57 - 61	59	A-->T
2GZVA	57 - 61	59	A-->V
2GZVA	57 - 61	60	L-->A
2GZVA	57 - 61	60	L-->D

2GZVA	57 - 61	60	L-->E
2GZVA	57 - 61	60	L-->G
2GZVA	57 - 61	60	L-->H
2GZVA	57 - 61	60	L-->P
2GZVA	57 - 61	60	L-->R
2GZVA	57 - 61	60	L-->T
2GZVA	57 - 61	60	L-->V
2GZVA	57 - 61	60	L-->Y
2GZVA	57 - 61	61	D-->A
2GZVA	57 - 61	61	D-->E
2GZVA	57 - 61	61	D-->G
2GZVA	57 - 61	61	D-->I
2GZVA	57 - 61	61	D-->K
2GZVA	57 - 61	61	D-->L
2GZVA	57 - 61	61	D-->N
2GZVA	57 - 61	61	D-->P
2GZVA	57 - 61	61	D-->S
2GZVA	57 - 61	61	D-->V
1K7CA	45 - 50	47	S-->L
1K7CA	45 - 50	49	T-->N
1K7CA	182 - 187	182	N-->T
1K7CA	182 - 187	185	V-->F
1K7CA	182 - 187	185	V-->T
1K7CA	182 - 187	187	S-->K
1K7CA	182 - 187	187	S-->Y
1K7CA	218 - 222	218	S-->A
1K7CA	218 - 222	218	S-->D

1K7CA	218 - 222	218	S-->K
1K7CA	218 - 222	218	S-->N
1K7CA	218 - 222	218	S-->P
1K7CA	218 - 222	218	S-->Q
1K7CA	218 - 222	218	S-->V
1K7CA	218 - 222	219	L-->D
1K7CA	218 - 222	219	L-->E
1K7CA	218 - 222	219	L-->G
1K7CA	218 - 222	219	L-->I
1K7CA	218 - 222	219	L-->K
1K7CA	218 - 222	219	L-->T
1K7CA	218 - 222	219	L-->Y
1K7CA	218 - 222	220	K-->A
1K7CA	218 - 222	220	K-->C
1K7CA	218 - 222	220	K-->F
1K7CA	218 - 222	220	K-->G
1K7CA	218 - 222	220	K-->L
1K7CA	218 - 222	220	K-->M
1K7CA	218 - 222	220	K-->P
1K7CA	218 - 222	220	K-->S
1K7CA	218 - 222	220	K-->V
1K7CA	218 - 222	220	K-->Y
1K7CA	218 - 222	221	S-->A
1K7CA	218 - 222	221	S-->D
1K7CA	218 - 222	221	S-->G
1K7CA	218 - 222	221	S-->I
1K7CA	218 - 222	221	S-->N

1K7CA	218	222	221	S-->T
1K7CA	218	222	222	V-->A
1K7CA	218	222	222	V-->E
1K7CA	218	222	222	V-->G
1K7CA	218	222	222	V-->L
1K7CA	218	222	222	V-->P
1K7CA	218	222	222	V-->Q
1K7CA	218	222	222	V-->R
1K7CA	218	222	222	V-->S
1K7CA	218	222	222	V-->T
3BF7B	33	39	37	R-->E
3BF7B	175	180	176	P-->R
3BF7B	175	180	177	V-->E
3BF7B	175	180	178	L-->H
3BF7B	175	180	179	W-->R
3BF7B	175	180	179	W-->V
3BF7B	182	186	182	Y-->A
3BF7B	182	186	182	Y-->C
3BF7B	182	186	182	Y-->H
3BF7B	182	186	182	Y-->L
3BF7B	182	186	182	Y-->Q
3BF7B	182	186	184	H-->A
3BF7B	182	186	184	H-->C
3BF7B	182	186	184	H-->E
3BF7B	182	186	184	H-->K
3BF7B	182	186	184	H-->N
3BF7B	182	186	184	H-->V

3BF7B	182 - 186	185	I-->E
3BF7B	182 - 186	185	I-->F
3BF7B	182 - 186	185	I-->H
3BF7B	182 - 186	185	I-->R
3BF7B	182 - 186	186	V-->G
3BF7B	182 - 186	186	V-->N
3BF7B	182 - 186	186	V-->P
2II2A	266 - 270	266	A-->G
2II2A	266 - 270	267	W-->E
2II2A	266 - 270	267	W-->G
2II2A	266 - 270	267	W-->I
2II2A	266 - 270	267	W-->K
2II2A	266 - 270	267	W-->S
2II2A	266 - 270	269	H-->N
2II2A	266 - 270	270	Y-->D
1SU8A	40 - 44	40	G-->F
1SU8A	40 - 44	40	G-->I
1SU8A	40 - 44	40	G-->P
1SU8A	40 - 44	40	G-->V
1SU8A	40 - 44	40	G-->Y
1SU8A	40 - 44	41	F-->A
1SU8A	40 - 44	41	F-->D
1SU8A	40 - 44	41	F-->E
1SU8A	40 - 44	41	F-->G
1SU8A	40 - 44	41	F-->I
1SU8A	40 - 44	41	F-->L
1SU8A	40 - 44	41	F-->Q

1SU8A	40 - 44	41	F-->R
1SU8A	40 - 44	41	F-->S
1SU8A	40 - 44	41	F-->T
1SU8A	40 - 44	41	F-->V
1SU8A	40 - 44	41	F-->W
1SU8A	40 - 44	42	G-->A
1SU8A	40 - 44	42	G-->D
1SU8A	40 - 44	42	G-->E
1SU8A	40 - 44	42	G-->I
1SU8A	40 - 44	42	G-->L
1SU8A	40 - 44	42	G-->N
1SU8A	40 - 44	42	G-->Q
1SU8A	40 - 44	42	G-->R
1SU8A	40 - 44	42	G-->V
1SU8A	40 - 44	42	G-->Y
1SU8A	40 - 44	43	E-->A
1SU8A	40 - 44	43	E-->F
1SU8A	40 - 44	43	E-->G
1SU8A	40 - 44	43	E-->K
1SU8A	40 - 44	43	E-->P
1SU8A	40 - 44	43	E-->R
1SU8A	40 - 44	43	E-->S
1SU8A	40 - 44	43	E-->T
1SU8A	40 - 44	44	T-->A
1SU8A	40 - 44	44	T-->D
1SU8A	40 - 44	44	T-->E
1SU8A	40 - 44	44	T-->F

1SU8A	40 - 44	44	T-->L
1SU8A	40 - 44	44	T-->P
1SU8A	40 - 44	44	T-->S
1SU8A	40 - 44	44	T-->V
1SU8A	40 - 44	44	T-->Y
1SU8A	277 - 282	278	N-->G
1SU8A	316 - 320	316	E-->S
1SU8A	316 - 320	317	M-->F
1SU8A	316 - 320	317	M-->N
1SU8A	316 - 320	317	M-->P
1SU8A	316 - 320	317	M-->R
1SU8A	316 - 320	318	A-->P
1SU8A	316 - 320	319	M-->F
1SU8A	316 - 320	319	M-->I
1SU8A	316 - 320	320	I-->R
1SU8A	489 - 495	495	L-->Y
1T2DA	50 - 54	50	T-->L
1T2DA	50 - 54	50	T-->Y
1T2DA	50 - 54	51	N-->E
1T2DA	50 - 54	51	N-->L
1T2DA	50 - 54	52	V-->E
1T2DA	50 - 54	52	V-->L
1T2DA	50 - 54	53	M-->C
1T2DA	50 - 54	53	M-->D
1T2DA	50 - 54	53	M-->F
1T2DA	50 - 54	53	M-->K
1T2DA	50 - 54	53	M-->L

1T2DA	50 - 54	53	M-->N	
1T2DA	50 - 54	53	M-->P	
1T2DA	50 - 54	53	M-->Q	
1T2DA	50 - 54	53	M-->S	
1T2DA	50 - 54	53	M-->T	
1T2DA	50 - 54	54	A-->K	
1T2DA	50 - 54	54	A-->S	
1T2DA	50 - 54	54	A-->V	
1T2DA	199 - 204	200	Q-->D	
1T2DA	199 - 204	202	F-->G	
1T2DA	199 - 204	203	I-->L	
2ABSA	31 - 36	31	S-->F	
2ABSA	31 - 36	32	S-->T	
2ABSA	31 - 36	34	L-->S	
2ABSA	31 - 36	36	E-->K	
2ABSA	143 - 147	143	G-->E	
2ABSA	143 - 147	143	G-->L	
2ABSA	143 - 147	143	G-->S	
2ABSA	143 - 147	143	G-->T	
2ABSA	143 - 147	144	A-->G	
2ABSA	143 - 147	144	A-->I	
2ABSA	143 - 147	144	A-->K	
2ABSA	143 - 147	144	A-->M	
2ABSA	143 - 147	144	A-->Q	
2ABSA	143 - 147	144	A-->S	
2ABSA	143 - 147	144	A-->T	
2ABSA	143 - 147	144	A-->V	

2ABSA	143	-	147	144	A-->W
2ABSA	143	-	147	144	A-->Y
2ABSA	143	-	147	145	C-->A
2ABSA	143	-	147	145	C-->D
2ABSA	143	-	147	145	C-->E
2ABSA	143	-	147	145	C-->F
2ABSA	143	-	147	145	C-->G
2ABSA	143	-	147	145	C-->I
2ABSA	143	-	147	145	C-->L
2ABSA	143	-	147	145	C-->M
2ABSA	143	-	147	145	C-->N
2ABSA	143	-	147	145	C-->P
2ABSA	143	-	147	145	C-->R
2ABSA	143	-	147	145	C-->S
2ABSA	143	-	147	145	C-->T
2ABSA	143	-	147	145	C-->V
2ABSA	143	-	147	145	C-->Y
2ABSA	143	-	147	146	G-->A
2ABSA	143	-	147	146	G-->C
2ABSA	143	-	147	146	G-->S
2ABSA	143	-	147	146	G-->T
2ABSA	143	-	147	146	G-->Y
2ABSA	143	-	147	147	S-->G
2ABSA	143	-	147	147	S-->V
2ABSA	143	-	147	147	S-->Y
2ABSA	200	-	205	200	P-->E
2ABSA	200	-	205	202	C-->R

2BHUA	143 - 148	143	Y-->A	
2BHUA	150 - 156	154	K-->D	
2BHUA	150 - 156	155	E-->V	
2BHUA	344 - 348	344	G-->F	
2BHUA	344 - 348	344	G-->P	
2BHUA	344 - 348	344	G-->V	
2BHUA	344 - 348	344	G-->Y	
2BHUA	344 - 348	345	Y-->A	
2BHUA	344 - 348	345	Y-->E	
2BHUA	344 - 348	345	Y-->G	
2BHUA	344 - 348	345	Y-->I	
2BHUA	344 - 348	345	Y-->K	
2BHUA	344 - 348	345	Y-->L	
2BHUA	344 - 348	345	Y-->N	
2BHUA	344 - 348	345	Y-->P	
2BHUA	344 - 348	345	Y-->T	
2BHUA	344 - 348	345	Y-->V	
2BHUA	344 - 348	346	Y-->A	
2BHUA	344 - 348	346	Y-->D	
2BHUA	344 - 348	346	Y-->E	
2BHUA	344 - 348	346	Y-->F	
2BHUA	344 - 348	346	Y-->G	
2BHUA	344 - 348	346	Y-->I	
2BHUA	344 - 348	346	Y-->K	
2BHUA	344 - 348	346	Y-->L	
2BHUA	344 - 348	346	Y-->N	
2BHUA	344 - 348	346	Y-->Q	

2BHUA	344 - 348	346	Y-->R
2BHUA	344 - 348	346	Y-->S
2BHUA	344 - 348	346	Y-->T
2BHUA	344 - 348	346	Y-->V
2BHUA	344 - 348	347	A-->C
2BHUA	344 - 348	347	A-->D
2BHUA	344 - 348	347	A-->H
2BHUA	344 - 348	347	A-->N
2BHUA	344 - 348	347	A-->R
2BHUA	344 - 348	347	A-->T
2BHUA	344 - 348	347	A-->V
2BHUA	344 - 348	347	A-->W
2BHUA	344 - 348	348	G-->A
2BHUA	344 - 348	348	G-->D
2BHUA	344 - 348	348	G-->M
2BHUA	344 - 348	348	G-->N
2BHUA	344 - 348	348	G-->P
2BHUA	344 - 348	348	G-->Y
2BHUA	399 - 403	399	H-->F
2BHUA	399 - 403	399	H-->G
2BHUA	399 - 403	399	H-->K
2BHUA	399 - 403	399	H-->L
2BHUA	399 - 403	399	H-->M
2BHUA	399 - 403	399	H-->N
2BHUA	399 - 403	399	H-->Y
2BHUA	399 - 403	400	D-->I
2BHUA	399 - 403	401	Q-->A

2BHUA	399 - 403	401	Q-->C
2BHUA	399 - 403	401	Q-->D
2BHUA	399 - 403	401	Q-->E
2BHUA	399 - 403	401	Q-->G
2BHUA	399 - 403	401	Q-->I
2BHUA	399 - 403	401	Q-->L
2BHUA	399 - 403	401	Q-->S
2BHUA	399 - 403	401	Q-->T
2BHUA	399 - 403	401	Q-->V
2BHUA	399 - 403	402	I-->E
2BHUA	399 - 403	402	I-->K
2BHUA	399 - 403	402	I-->N
2BHUA	399 - 403	402	I-->P
2BHUA	399 - 403	402	I-->V
2BHUA	489 - 493	489	E-->C
2BHUA	489 - 493	489	E-->P
2BHUA	489 - 493	489	E-->T
2BHUA	489 - 493	489	E-->W
2BHUA	489 - 493	490	Q-->D
2BHUA	489 - 493	490	Q-->I
2BHUA	489 - 493	490	Q-->L
2BHUA	489 - 493	490	Q-->N
2BHUA	489 - 493	490	Q-->R
2BHUA	489 - 493	490	Q-->V
2BHUA	489 - 493	491	T-->C
2BHUA	489 - 493	491	T-->E
2BHUA	489 - 493	491	T-->Q

2BHUA	489 - 493	491	T-->S
2BHUA	489 - 493	491	T-->V
2BHUA	489 - 493	492	F-->D
2BHUA	489 - 493	492	F-->G
2BHUA	489 - 493	492	F-->H
2BHUA	489 - 493	492	F-->L
2BHUA	489 - 493	492	F-->P
2BHUA	489 - 493	492	F-->Q
2BHUA	489 - 493	493	L-->F
2BHUA	489 - 493	493	L-->G
2BHUA	489 - 493	493	L-->I
2BHUA	489 - 493	493	L-->K
2BHUA	489 - 493	493	L-->M
1QL0A	79 - 83	79	A-->D
1QL0A	79 - 83	79	A-->E
1QL0A	79 - 83	79	A-->G
1QL0A	79 - 83	79	A-->H
1QL0A	79 - 83	79	A-->I
1QL0A	79 - 83	79	A-->N
1QL0A	79 - 83	79	A-->R
1QL0A	79 - 83	80	N-->A
1QL0A	79 - 83	80	N-->C
1QL0A	79 - 83	80	N-->D
1QL0A	79 - 83	80	N-->E
1QL0A	79 - 83	80	N-->G
1QL0A	79 - 83	80	N-->I
1QL0A	79 - 83	80	N-->K

1QL0A	79 - 83	80	N-->L
1QL0A	79 - 83	80	N-->P
1QL0A	79 - 83	80	N-->Q
1QL0A	79 - 83	80	N-->R
1QL0A	79 - 83	80	N-->S
1QL0A	79 - 83	80	N-->T
1QL0A	79 - 83	80	N-->V
1QL0A	79 - 83	81	A-->G
1QL0A	79 - 83	81	A-->H
1QL0A	79 - 83	81	A-->I
1QL0A	79 - 83	81	A-->K
1QL0A	79 - 83	81	A-->N
1QL0A	79 - 83	81	A-->P
1QL0A	79 - 83	81	A-->W
1QL0A	79 - 83	82	A-->C
1QL0A	79 - 83	82	A-->D
1QL0A	79 - 83	82	A-->F
1QL0A	79 - 83	82	A-->G
1QL0A	79 - 83	82	A-->H
1QL0A	79 - 83	82	A-->K
1QL0A	79 - 83	82	A-->L
1QL0A	79 - 83	82	A-->P
1QL0A	79 - 83	82	A-->R
1QL0A	79 - 83	82	A-->S
1QL0A	79 - 83	82	A-->T
1QL0A	79 - 83	83	L-->G
1QL0A	79 - 83	83	L-->H

1QL0A	79 - 83	83	L-->I
1QL0A	79 - 83	83	L-->K
1QL0A	79 - 83	83	L-->N
1QL0A	79 - 83	83	L-->P
1QL0A	79 - 83	83	L-->R
1QL0A	79 - 83	83	L-->S
1QL0A	79 - 83	83	L-->V
1QL0A	102 - 106	102	W-->A
1QL0A	102 - 106	102	W-->C
1QL0A	102 - 106	102	W-->D
1QL0A	102 - 106	102	W-->E
1QL0A	102 - 106	102	W-->F
1QL0A	102 - 106	102	W-->G
1QL0A	102 - 106	102	W-->H
1QL0A	102 - 106	102	W-->I
1QL0A	102 - 106	102	W-->K
1QL0A	102 - 106	102	W-->Q
1QL0A	102 - 106	102	W-->T
1QL0A	102 - 106	103	E-->I
1QL0A	102 - 106	103	E-->S
1QL0A	102 - 106	103	E-->Y
1QL0A	102 - 106	104	S-->F
1QL0A	102 - 106	104	S-->G
1QL0A	102 - 106	104	S-->M
1QL0A	102 - 106	105	L-->E
1QL0A	102 - 106	106	N-->F
1QL0A	102 - 106	106	N-->Q

1QL0A	102 - 106	106	N-->R
1QL0A	237 - 241	237	L-->V
1QL0A	237 - 241	238	P-->D
1QL0A	237 - 241	238	P-->G
1QL0A	237 - 241	238	P-->L
1QL0A	237 - 241	238	P-->T
1QL0A	237 - 241	238	P-->V
1QL0A	237 - 241	239	E-->G
1QL0A	237 - 241	239	E-->S
1QL0A	237 - 241	239	E-->V
1QL0A	237 - 241	240	L-->A
1QL0A	237 - 241	240	L-->G
1QL0A	237 - 241	240	L-->K
1QL0A	237 - 241	240	L-->Y
1QL0A	237 - 241	241	M-->A
1QL0A	237 - 241	241	M-->D
1QL0A	237 - 241	241	M-->E
1QL0A	237 - 241	241	M-->F
1QL0A	237 - 241	241	M-->I
1QL0A	237 - 241	241	M-->K
1QL0A	237 - 241	241	M-->L
1QL0A	237 - 241	241	M-->N
1QL0A	237 - 241	241	M-->P
1QL0A	237 - 241	241	M-->Q
1QL0A	237 - 241	241	M-->R
1QL0A	237 - 241	241	M-->S
1QL0A	237 - 241	241	M-->T

2OSXA	308 - 312	308	L-->A
2OSXA	308 - 312	308	L-->D
2OSXA	308 - 312	308	L-->F
2OSXA	308 - 312	308	L-->G
2OSXA	308 - 312	308	L-->I
2OSXA	308 - 312	308	L-->N
2OSXA	308 - 312	308	L-->Q
2OSXA	308 - 312	308	L-->R
2OSXA	308 - 312	308	L-->S
2OSXA	308 - 312	308	L-->W
2OSXA	308 - 312	308	L-->Y
2OSXA	308 - 312	309	P-->A
2OSXA	308 - 312	309	P-->C
2OSXA	308 - 312	309	P-->D
2OSXA	308 - 312	309	P-->E
2OSXA	308 - 312	309	P-->G
2OSXA	308 - 312	309	P-->I
2OSXA	308 - 312	309	P-->K
2OSXA	308 - 312	309	P-->L
2OSXA	308 - 312	309	P-->N
2OSXA	308 - 312	309	P-->Q
2OSXA	308 - 312	309	P-->S
2OSXA	308 - 312	309	P-->T
2OSXA	308 - 312	309	P-->V
2OSXA	308 - 312	309	P-->W
2OSXA	308 - 312	309	P-->Y
2OSXA	308 - 312	310	L-->A

2OSXA	308 - 312	310	L-->D
2OSXA	308 - 312	310	L-->E
2OSXA	308 - 312	310	L-->F
2OSXA	308 - 312	310	L-->G
2OSXA	308 - 312	310	L-->I
2OSXA	308 - 312	310	L-->K
2OSXA	308 - 312	310	L-->N
2OSXA	308 - 312	310	L-->P
2OSXA	308 - 312	310	L-->Q
2OSXA	308 - 312	310	L-->S
2OSXA	308 - 312	310	L-->V
2OSXA	308 - 312	310	L-->W
2OSXA	308 - 312	310	L-->Y
2OSXA	308 - 312	311	D-->E
2OSXA	308 - 312	311	D-->G
2OSXA	308 - 312	311	D-->I
2OSXA	308 - 312	311	D-->N
2OSXA	308 - 312	311	D-->P
2OSXA	308 - 312	311	D-->Q
2OSXA	308 - 312	311	D-->R
2OSXA	308 - 312	311	D-->S
2OSXA	308 - 312	311	D-->T
2OSXA	308 - 312	311	D-->V
2OSXA	308 - 312	311	D-->Y
2OSXA	308 - 312	312	I-->A
2OSXA	308 - 312	312	I-->E
2OSXA	308 - 312	312	I-->F

2OSXA	308 - 312	312	I-->K
2OSXA	308 - 312	312	I-->L
2OSXA	308 - 312	312	I-->M
2OSXA	308 - 312	312	I-->N
2OSXA	308 - 312	312	I-->P
2OSXA	308 - 312	312	I-->Q
2OSXA	308 - 312	312	I-->R
2OSXA	308 - 312	312	I-->S
2OSXA	308 - 312	312	I-->V
2OSXA	400 - 405	400	L-->H
2OSXA	400 - 405	400	L-->K
2OSXA	400 - 405	401	L-->G
2OSXA	400 - 405	401	L-->K
2OSXA	400 - 405	401	L-->V
2OSXA	400 - 405	402	V-->D
2OSXA	400 - 405	403	D-->G
2OSXA	400 - 405	403	D-->R
2OSXA	400 - 405	405	L-->G
2OSXA	400 - 405	405	L-->P
2OSXA	400 - 405	405	L-->V
1ZL0B	100 - 105	102	R-->P
1ZL0B	100 - 105	105	A-->T
1ZL0B	137 - 141	137	A-->C
1ZL0B	137 - 141	137	A-->D
1ZL0B	137 - 141	137	A-->E
1ZL0B	137 - 141	137	A-->F
1ZL0B	137 - 141	137	A-->G

1ZL0B	137 - 141	137	A-->H
1ZL0B	137 - 141	137	A-->I
1ZL0B	137 - 141	137	A-->K
1ZL0B	137 - 141	137	A-->L
1ZL0B	137 - 141	137	A-->P
1ZL0B	137 - 141	137	A-->R
1ZL0B	137 - 141	137	A-->S
1ZL0B	137 - 141	137	A-->V
1ZL0B	137 - 141	138	T-->A
1ZL0B	137 - 141	138	T-->D
1ZL0B	137 - 141	138	T-->E
1ZL0B	137 - 141	138	T-->F
1ZL0B	137 - 141	138	T-->G
1ZL0B	137 - 141	138	T-->I
1ZL0B	137 - 141	138	T-->L
1ZL0B	137 - 141	138	T-->P
1ZL0B	137 - 141	138	T-->S
1ZL0B	137 - 141	138	T-->V
1ZL0B	137 - 141	138	T-->Y
1ZL0B	137 - 141	139	G-->A
1ZL0B	137 - 141	139	G-->C
1ZL0B	137 - 141	139	G-->D
1ZL0B	137 - 141	139	G-->E
1ZL0B	137 - 141	139	G-->I
1ZL0B	137 - 141	139	G-->K
1ZL0B	137 - 141	139	G-->L
1ZL0B	137 - 141	139	G-->N

1ZL0B	137 - 141	139	G-->P
1ZL0B	137 - 141	139	G-->Q
1ZL0B	137 - 141	139	G-->R
1ZL0B	137 - 141	139	G-->S
1ZL0B	137 - 141	139	G-->T
1ZL0B	137 - 141	139	G-->V
1ZL0B	137 - 141	139	G-->W
1ZL0B	137 - 141	139	G-->Y
1ZL0B	137 - 141	140	L-->D
1ZL0B	137 - 141	140	L-->E
1ZL0B	137 - 141	140	L-->F
1ZL0B	137 - 141	140	L-->G
1ZL0B	137 - 141	140	L-->H
1ZL0B	137 - 141	140	L-->I
1ZL0B	137 - 141	140	L-->K
1ZL0B	137 - 141	140	L-->N
1ZL0B	137 - 141	140	L-->P
1ZL0B	137 - 141	140	L-->R
1ZL0B	137 - 141	140	L-->S
1ZL0B	137 - 141	140	L-->T
1ZL0B	137 - 141	140	L-->W
1ZL0B	137 - 141	140	L-->Y
1ZL0B	137 - 141	141	G-->C
1ZL0B	137 - 141	141	G-->D
1ZL0B	137 - 141	141	G-->F
1ZL0B	137 - 141	141	G-->H
1ZL0B	137 - 141	141	G-->K

1ZL0B	137 - 141	141	G-->R
1ZL0B	137 - 141	141	G-->S
1ZL0B	137 - 141	141	G-->T
1ZL0B	137 - 141	141	G-->W
1ZL0B	193 - 197	193	L-->A
1ZL0B	193 - 197	193	L-->D
1ZL0B	193 - 197	193	L-->F
1ZL0B	193 - 197	193	L-->H
1ZL0B	193 - 197	193	L-->K
1ZL0B	193 - 197	193	L-->N
1ZL0B	193 - 197	193	L-->P
1ZL0B	193 - 197	193	L-->R
1ZL0B	193 - 197	193	L-->S
1ZL0B	193 - 197	193	L-->T
1ZL0B	193 - 197	193	L-->V
1ZL0B	193 - 197	193	L-->Y
1ZL0B	193 - 197	194	T-->A
1ZL0B	193 - 197	194	T-->D
1ZL0B	193 - 197	194	T-->F
1ZL0B	193 - 197	194	T-->H
1ZL0B	193 - 197	194	T-->I
1ZL0B	193 - 197	194	T-->L
1ZL0B	193 - 197	194	T-->N
1ZL0B	193 - 197	194	T-->P
1ZL0B	193 - 197	194	T-->S
1ZL0B	193 - 197	194	T-->V
1ZL0B	193 - 197	195	A-->D

1ZL0B	193 - 197	195	A-->E
1ZL0B	193 - 197	195	A-->G
1ZL0B	193 - 197	195	A-->H
1ZL0B	193 - 197	195	A-->I
1ZL0B	193 - 197	195	A-->K
1ZL0B	193 - 197	195	A-->L
1ZL0B	193 - 197	195	A-->M
1ZL0B	193 - 197	195	A-->P
1ZL0B	193 - 197	195	A-->R
1ZL0B	193 - 197	195	A-->T
1ZL0B	193 - 197	196	L-->A
1ZL0B	193 - 197	196	L-->D
1ZL0B	193 - 197	196	L-->E
1ZL0B	193 - 197	196	L-->F
1ZL0B	193 - 197	196	L-->G
1ZL0B	193 - 197	196	L-->I
1ZL0B	193 - 197	196	L-->K
1ZL0B	193 - 197	196	L-->N
1ZL0B	193 - 197	196	L-->P
1ZL0B	193 - 197	196	L-->Q
1ZL0B	193 - 197	196	L-->R
1ZL0B	193 - 197	196	L-->S
1ZL0B	193 - 197	196	L-->T
1ZL0B	193 - 197	196	L-->V
1ZL0B	193 - 197	197	A-->G
1ZL0B	193 - 197	197	A-->K
1ZL0B	193 - 197	197	A-->L

1ZL0B	193 - 197	197	A-->N
1ZL0B	193 - 197	197	A-->P
1ZL0B	193 - 197	197	A-->S
1ZL0B	193 - 197	197	A-->T
1ZL0B	193 - 197	197	A-->Y
1C5EA	94 - 98	94	E-->H
1C5EA	94 - 98	94	E-->K
1C5EA	94 - 98	94	E-->R
1C5EA	94 - 98	95	T-->G
1C5EA	94 - 98	95	T-->K
1C5EA	94 - 98	95	T-->R
1C5EA	94 - 98	96	K-->A
1C5EA	94 - 98	96	K-->D
1C5EA	94 - 98	96	K-->E
1C5EA	94 - 98	96	K-->L
1C5EA	94 - 98	96	K-->R
1C5EA	94 - 98	96	K-->S
1C5EA	94 - 98	96	K-->T
1C5EA	94 - 98	97	K-->C
1C5EA	94 - 98	97	K-->D
1C5EA	94 - 98	97	K-->E
1C5EA	94 - 98	97	K-->F
1C5EA	94 - 98	97	K-->I
1C5EA	94 - 98	97	K-->R
1C5EA	94 - 98	97	K-->V
1C5EA	94 - 98	98	R-->A
1C5EA	94 - 98	98	R-->D

1C5EA	94 - 98	98	R-->E
1C5EA	94 - 98	98	R-->G
1C5EA	94 - 98	98	R-->K
1C5EA	94 - 98	98	R-->L
1C5EA	94 - 98	98	R-->V
1C5EA	94 - 98	98	R-->Y
1N62C	37 - 42	41	M-->E
1N62C	37 - 42	41	M-->G
1N62C	78 - 83	82	I-->T
1N62C	85 - 90	86	F-->L
1N62C	85 - 90	88	A-->I
1N62C	85 - 90	88	A-->N
1N62C	85 - 90	90	K-->I
1N62C	92 - 98	96	E-->V
1N62C	104 - 109	105	Q-->N
1N62C	104 - 109	109	M-->L
1N62C	112 - 117	113	G-->F
1N62C	112 - 117	113	G-->T
1N62C	112 - 117	113	G-->V
1N62C	112 - 117	115	N-->H
1N62C	112 - 117	115	N-->K
1N62C	112 - 117	115	N-->V
1N62C	112 - 117	116	A-->E
1N62C	227 - 233	227	E-->I
1QLWA	115 - 121	117	I-->T
1W23B	226 - 231	226	Y-->K
1W23B	226 - 231	228	T-->A

1W66A	168 - 172	168	A-->F
1W66A	168 - 172	168	A-->I
1W66A	168 - 172	168	A-->P
1W66A	168 - 172	168	A-->Q
1W66A	168 - 172	168	A-->V
1W66A	168 - 172	169	A-->G
1W66A	168 - 172	169	A-->L
1W66A	168 - 172	169	A-->N
1W66A	168 - 172	169	A-->T
1W66A	168 - 172	169	A-->W
1W66A	168 - 172	169	A-->Y
1W66A	168 - 172	170	F-->A
1W66A	168 - 172	170	F-->D
1W66A	168 - 172	170	F-->E
1W66A	168 - 172	170	F-->G
1W66A	168 - 172	170	F-->H
1W66A	168 - 172	170	F-->I
1W66A	168 - 172	170	F-->L
1W66A	168 - 172	170	F-->N
1W66A	168 - 172	170	F-->P
1W66A	168 - 172	170	F-->R
1W66A	168 - 172	170	F-->S
1W66A	168 - 172	170	F-->T
1W66A	168 - 172	170	F-->V
1W66A	168 - 172	171	T-->E
1W66A	168 - 172	171	T-->I
1W66A	168 - 172	171	T-->P

1W66A	168 - 172	171	T-->R	
1W66A	168 - 172	172	A-->C	
1W66A	168 - 172	172	A-->G	
1W66A	168 - 172	172	A-->I	
1W66A	168 - 172	172	A-->L	
1W66A	168 - 172	172	A-->M	
1W66A	168 - 172	172	A-->P	
1W66A	168 - 172	172	A-->S	
1W66A	168 - 172	172	A-->V	
1W66A	186 - 190	186	L-->A	
1W66A	186 - 190	186	L-->E	
1W66A	186 - 190	186	L-->F	
1W66A	186 - 190	186	L-->G	
1W66A	186 - 190	186	L-->H	
1W66A	186 - 190	186	L-->K	
1W66A	186 - 190	186	L-->P	
1W66A	186 - 190	186	L-->Q	
1W66A	186 - 190	186	L-->S	
1W66A	186 - 190	186	L-->T	
1W66A	186 - 190	186	L-->V	
1W66A	186 - 190	186	L-->Y	
1W66A	186 - 190	187	S-->E	
1W66A	186 - 190	187	S-->G	
1W66A	186 - 190	187	S-->H	
1W66A	186 - 190	187	S-->K	
1W66A	186 - 190	187	S-->M	
1W66A	186 - 190	187	S-->T	

1W66A	186 - 190	187	S-->V
1W66A	186 - 190	187	S-->Y
1W66A	186 - 190	188	A-->C
1W66A	186 - 190	188	A-->F
1W66A	186 - 190	188	A-->G
1W66A	186 - 190	188	A-->K
1W66A	186 - 190	188	A-->L
1W66A	186 - 190	188	A-->R
1W66A	186 - 190	188	A-->S
1W66A	186 - 190	188	A-->T
1W66A	186 - 190	188	A-->V
1W66A	186 - 190	189	E-->A
1W66A	186 - 190	189	E-->F
1W66A	186 - 190	189	E-->I
1W66A	186 - 190	189	E-->L
1W66A	186 - 190	189	E-->N
1W66A	186 - 190	189	E-->P
1W66A	186 - 190	189	E-->Q
1W66A	186 - 190	189	E-->S
1W66A	186 - 190	189	E-->T
1W66A	186 - 190	189	E-->V
1W66A	186 - 190	190	L-->A
1W66A	186 - 190	190	L-->D
1W66A	186 - 190	190	L-->E
1W66A	186 - 190	190	L-->F
1W66A	186 - 190	190	L-->G
1W66A	186 - 190	190	L-->I

1W66A	186 - 190	190	L-->K
1W66A	186 - 190	190	L-->N
1W66A	186 - 190	190	L-->R
1W66A	186 - 190	190	L-->S
1W66A	186 - 190	190	L-->V
1UWCA	234 - 238	234	C-->G
1UWCA	234 - 238	234	C-->L
1UWCA	234 - 238	234	C-->W
1UWCA	234 - 238	234	C-->Y
1DS1A	177 - 183	182	P-->K
2QOLA	793 - 798	796	I-->G
2QOLA	793 - 798	797	A-->D
2QOLA	793 - 798	798	Y-->G
2QOLA	793 - 797	793	P-->A
2QOLA	793 - 797	793	P-->D
2QOLA	793 - 797	793	P-->E
2QOLA	793 - 797	793	P-->G
2QOLA	793 - 797	793	P-->I
2QOLA	793 - 797	793	P-->K
2QOLA	793 - 797	793	P-->Q
2QOLA	793 - 797	793	P-->R
2QOLA	793 - 797	793	P-->V
2QOLA	793 - 797	793	P-->Y
2QOLA	793 - 797	794	G-->A
2QOLA	793 - 797	794	G-->D
2QOLA	793 - 797	794	G-->I
2QOLA	793 - 797	794	G-->L

2QOLA	793 - 797	794	G-->M
2QOLA	793 - 797	794	G-->Q
2QOLA	793 - 797	794	G-->R
2QOLA	793 - 797	794	G-->S
2QOLA	793 - 797	794	G-->T
2QOLA	793 - 797	794	G-->V
2QOLA	793 - 797	795	S-->E
2QOLA	793 - 797	795	S-->G
2QOLA	793 - 797	795	S-->H
2QOLA	793 - 797	795	S-->I
2QOLA	793 - 797	795	S-->K
2QOLA	793 - 797	795	S-->L
2QOLA	793 - 797	795	S-->Q
2QOLA	793 - 797	795	S-->R
2QOLA	793 - 797	795	S-->V
2QOLA	793 - 797	796	L-->C
2QOLA	793 - 797	796	L-->D
2QOLA	793 - 797	796	L-->E
2QOLA	793 - 797	796	L-->F
2QOLA	793 - 797	796	L-->G
2QOLA	793 - 797	796	L-->K
2QOLA	793 - 797	796	L-->N
2QOLA	793 - 797	796	L-->P
2QOLA	793 - 797	796	L-->Q
2QOLA	793 - 797	796	L-->V
2QOLA	793 - 797	797	K-->A
2QOLA	793 - 797	797	K-->C

2QOLA	793 - 797	797	K-->D
2QOLA	793 - 797	797	K-->E
2QOLA	793 - 797	797	K-->F
2QOLA	793 - 797	797	K-->G
2QOLA	793 - 797	797	K-->H
2QOLA	793 - 797	797	K-->I
2QOLA	793 - 797	797	K-->L
2QOLA	793 - 797	797	K-->M
2QOLA	793 - 797	797	K-->P
2QOLA	793 - 797	797	K-->R
2QOLA	793 - 797	797	K-->T
2QOLA	793 - 797	797	K-->V
2QOLA	793 - 797	797	K-->W
1XJUA	32 - 38	36	E-->Q
1XJUA	118 - 122	118	C-->G
1XJUA	118 - 122	118	C-->L
1XJUA	118 - 122	118	C-->M
1XJUA	118 - 122	118	C-->Q
1XJUA	118 - 122	118	C-->V
1XJUA	118 - 122	118	C-->W
1XJUA	118 - 122	118	C-->Y
1XJUA	118 - 122	119	N-->C
1XJUA	118 - 122	119	N-->F
1XJUA	118 - 122	119	N-->G
1XJUA	118 - 122	121	L-->A
1XJUA	118 - 122	121	L-->G
1XJUA	118 - 122	121	L-->I

1XJUA	118	122	121	L-->K
1XJUA	118	122	121	L-->N
1XJUA	118	122	121	L-->R
1XJUA	118	122	121	L-->V
1XJUA	118	122	121	L-->W
1XJUA	118	122	122	R-->C
1XJUA	118	122	122	R-->D
1XJUA	118	122	122	R-->L
1XJUA	118	122	122	R-->T
2FFYA	106	111	106	L-->I
2FFYA	106	111	108	H-->K
2FFYA	106	111	108	H-->V
2FFYA	106	111	109	L-->F
2FFYA	106	111	110	A-->D
2FFYA	106	111	110	A-->K
2FFYA	106	111	111	T-->P
2FFYA	106	111	111	T-->Q
2FFYA	193	198	193	P-->L
2FFYA	193	198	193	P-->R
2FFYA	193	198	194	A-->G
2FFYA	193	198	196	E-->F
2FFYA	193	198	198	N-->E
2FFYA	193	198	198	N-->V
2FFYA	280	285	280	P-->K
2FFYA	280	285	282	S-->H
2FFYA	280	285	282	S-->L
2FFYA	280	285	282	S-->V

2FFYA	280 - 285	285	N-->A
2FFYA	280 - 285	285	N-->L
2O90A	19 - 23	19	D-->E
2O90A	19 - 23	19	D-->R
2O90A	19 - 23	19	D-->S
2O90A	19 - 23	19	D-->V
2O90A	19 - 23	20	W-->E
2O90A	19 - 23	20	W-->K
2O90A	19 - 23	20	W-->V
2O90A	19 - 23	20	W-->Y
2O90A	19 - 23	22	Q-->A
2O90A	19 - 23	22	Q-->N
2O90A	19 - 23	22	Q-->R
2O90A	19 - 23	22	Q-->S
2O90A	19 - 23	22	Q-->V
2O90A	39 - 44	42	A-->N
2O90A	39 - 44	42	A-->P
2O90A	39 - 44	42	A-->Q
1SFSA	39 - 43	39	Q-->A
1SFSA	39 - 43	39	Q-->H
1SFSA	39 - 43	39	Q-->I
1SFSA	39 - 43	39	Q-->N
1SFSA	39 - 43	39	Q-->P
1SFSA	39 - 43	39	Q-->T
1SFSA	39 - 43	39	Q-->Y
1SFSA	39 - 43	40	V-->K
1SFSA	39 - 43	40	V-->L

1SFSA	39 - 43	40	V-->R
1SFSA	39 - 43	40	V-->T
1SFSA	39 - 43	40	V-->Y
1SFSA	39 - 43	41	A-->D
1SFSA	39 - 43	41	A-->E
1SFSA	39 - 43	41	A-->L
1SFSA	39 - 43	41	A-->T
1SFSA	39 - 43	41	A-->V
1SFSA	39 - 43	42	V-->L
1SFSA	39 - 43	43	Q-->C
1SFSA	39 - 43	43	Q-->E
1SFSA	39 - 43	43	Q-->I
1SFSA	39 - 43	43	Q-->K
1SFSA	39 - 43	43	Q-->L
1SFSA	39 - 43	43	Q-->M
1SFSA	39 - 43	43	Q-->P
1SFSA	39 - 43	43	Q-->V
1SFSA	39 - 43	43	Q-->Y
1PMHX	93 - 98	94	D-->I
1PMHX	93 - 98	95	E-->G
1PMHX	93 - 98	96	F-->A
2DKOA	136 - 141	137	K-->N
2DKOA	136 - 141	139	I-->Q
2DKOA	136 - 141	139	I-->S
2DKOA	136 - 141	141	N-->G
2Z8AA	1003 - 1008	1005	D-->L
2Z8AA	1003 - 1008	1008	A-->G

2Z8AA	1003 - 1008	1008	A-->T	
2Z8AA	1057 - 1062	1059	Q-->D	
2Z8AA	1057 - 1062	1060	G-->S	
2Z8AA	1057 - 1062	1062	A-->S	
2HINB	55 - 60	57	T-->P	
2HINB	55 - 60	59	Q-->E	
2HINB	55 - 60	60	R-->I	
2CZQA	103 - 107	103	A-->D	
2CZQA	103 - 107	103	A-->E	
2CZQA	103 - 107	103	A-->G	
2CZQA	103 - 107	103	A-->I	
2CZQA	103 - 107	103	A-->L	
2CZQA	103 - 107	103	A-->N	
2CZQA	103 - 107	103	A-->P	
2CZQA	103 - 107	103	A-->S	
2CZQA	103 - 107	104	A-->C	
2CZQA	103 - 107	104	A-->D	
2CZQA	103 - 107	104	A-->S	
2CZQA	103 - 107	104	A-->Y	
2CZQA	103 - 107	105	F-->A	
2CZQA	103 - 107	105	F-->D	
2CZQA	103 - 107	105	F-->G	
2CZQA	103 - 107	105	F-->L	
2CZQA	103 - 107	105	F-->Q	
2CZQA	103 - 107	105	F-->V	
2CZQA	103 - 107	105	F-->Y	
2CZQA	103 - 107	106	N-->E	

2CZQA	103 - 107	106	N-->I
2CZQA	103 - 107	106	N-->P
2CZQA	103 - 107	106	N-->R
2CZQA	103 - 107	107	A-->D
2CZQA	103 - 107	107	A-->I
2CZQA	103 - 107	107	A-->L
2CZQA	103 - 107	107	A-->N
2CZQA	103 - 107	107	A-->P
2CZQA	103 - 107	107	A-->R
2CZQA	103 - 107	107	A-->S
2CZQA	103 - 107	107	A-->V
2CZQA	103 - 107	107	A-->W
2CZQA	103 - 107	107	A-->Y
2CZQA	151 - 156	151	S-->I
2CZQA	151 - 156	155	S-->V
2D5MA	92 - 96	92	K-->D
2D5MA	92 - 96	92	K-->G
2D5MA	92 - 96	92	K-->N
2D5MA	92 - 96	92	K-->P
2D5MA	92 - 96	92	K-->S
2D5MA	92 - 96	92	K-->Y
2D5MA	92 - 96	93	F-->D
2D5MA	92 - 96	93	F-->E
2D5MA	92 - 96	93	F-->K
2D5MA	92 - 96	93	F-->L
2D5MA	92 - 96	93	F-->M
2D5MA	92 - 96	93	F-->N

2D5MA	92 - 96	93	F-->P
2D5MA	92 - 96	93	F-->V
2D5MA	92 - 96	94	A-->I
2D5MA	92 - 96	94	A-->K
2D5MA	92 - 96	94	A-->L
2D5MA	92 - 96	94	A-->R
2D5MA	92 - 96	94	A-->T
2D5MA	92 - 96	94	A-->V
2D5MA	92 - 96	95	S-->A
2D5MA	92 - 96	95	S-->D
2D5MA	92 - 96	95	S-->G
2D5MA	92 - 96	95	S-->H
2D5MA	92 - 96	95	S-->N
2D5MA	92 - 96	95	S-->Q
2D5MA	92 - 96	95	S-->T
2D5MA	92 - 96	95	S-->V
2D5MA	92 - 96	96	L-->A
2D5MA	92 - 96	96	L-->C
2D5MA	92 - 96	96	L-->K
2D5MA	92 - 96	96	L-->Q
2D5MA	92 - 96	96	L-->R
2D5MA	92 - 96	96	L-->S
2D5MA	92 - 96	96	L-->T
2D5MA	92 - 96	96	L-->Y
2RK3A	58 - 62	58	L-->D
2RK3A	58 - 62	58	L-->G
2RK3A	58 - 62	58	L-->R

2RK3A	58 - 62	59	E-->A
2RK3A	58 - 62	59	E-->G
2RK3A	58 - 62	59	E-->I
2RK3A	58 - 62	59	E-->K
2RK3A	58 - 62	59	E-->L
2RK3A	58 - 62	59	E-->N
2RK3A	58 - 62	59	E-->S
2RK3A	58 - 62	59	E-->T
2RK3A	58 - 62	59	E-->V
2RK3A	58 - 62	59	E-->Y
2RK3A	58 - 62	60	D-->G
2RK3A	58 - 62	60	D-->S
2RK3A	58 - 62	60	D-->V
2RK3A	58 - 62	60	D-->Y
2RK3A	58 - 62	61	A-->D
2RK3A	58 - 62	61	A-->E
2RK3A	58 - 62	61	A-->G
2RK3A	58 - 62	61	A-->I
2RK3A	58 - 62	61	A-->P
2RK3A	58 - 62	61	A-->V
2RK3A	58 - 62	61	A-->Y
2RK3A	58 - 62	62	K-->A
2RK3A	58 - 62	62	K-->D
2RK3A	58 - 62	62	K-->E
2RK3A	58 - 62	62	K-->F
2RK3A	58 - 62	62	K-->G
2RK3A	58 - 62	62	K-->L

2RK3A	58	62	62	K-->N
2RK3A	58	62	62	K-->P
2RK3A	58	62	62	K-->Q
2RK3A	58	62	62	K-->R
2RK3A	58	62	62	K-->S
2RK3A	58	62	62	K-->V
2RK3A	58	62	62	K-->Y
2RK3A	109	114	109	P-->F
2RK3A	109	114	109	P-->R
2RK3A	109	114	110	T-->V
2RK3A	109	114	111	A-->N
2RK3A	109	114	112	L-->A
2RK3A	109	114	112	L-->K
2RK3A	109	114	112	L-->R
2RK3A	109	114	112	L-->T
2RK3A	109	114	113	L-->R
1KMVA	54	59	58	F-->H
1KMVA	54	59	58	F-->P
1KMVA	54	59	59	S-->A
1RQWA	148	152	148	A-->E
1RQWA	148	152	148	A-->N
1RQWA	148	152	149	C-->A
1RQWA	148	152	149	C-->D
1RQWA	148	152	149	C-->E
1RQWA	148	152	149	C-->F
1RQWA	148	152	149	C-->G
1RQWA	148	152	149	C-->I

1RQWA	148	152	149	C-->K
1RQWA	148	152	149	C-->L
1RQWA	148	152	149	C-->N
1RQWA	148	152	149	C-->P
1RQWA	148	152	149	C-->Q
1RQWA	148	152	149	C-->R
1RQWA	148	152	149	C-->S
1RQWA	148	152	149	C-->V
1RQWA	148	152	149	C-->Y
1RQWA	148	152	150	T-->A
1RQWA	148	152	150	T-->V
1RQWA	148	152	151	V-->L
1RQWA	148	152	151	V-->N
1RQWA	148	152	152	F-->D
1RQWA	148	152	152	F-->E
1RQWA	148	152	152	F-->I
1RQWA	148	152	152	F-->L
1RQWA	148	152	152	F-->M
1RQWA	148	152	152	F-->P
1RQWA	148	152	152	F-->S
2HBWA	70	75	70	L-->D
2HBWA	70	75	70	L-->T
2HBWA	70	75	70	L-->Y
2HBWA	70	75	71	G-->D
2HBWA	70	75	71	G-->K
2HBWA	70	75	72	D-->L
2HBWA	70	75	73	L-->E

2HBWA	70 - 75	73	L-->S
2HBWA	70 - 75	74	G-->I
2HBWA	70 - 75	74	G-->R
2HBWA	70 - 75	74	G-->S
2HBWA	70 - 75	75	L-->H
2HBWA	70 - 75	75	L-->K
2HBWA	131 - 135	131	Q-->C
2HBWA	131 - 135	131	Q-->D
2HBWA	131 - 135	131	Q-->E
2HBWA	131 - 135	131	Q-->F
2HBWA	131 - 135	131	Q-->G
2HBWA	131 - 135	131	Q-->K
2HBWA	131 - 135	131	Q-->L
2HBWA	131 - 135	131	Q-->R
2HBWA	131 - 135	131	Q-->S
2HBWA	131 - 135	131	Q-->V
2HBWA	131 - 135	132	A-->E
2HBWA	131 - 135	132	A-->F
2HBWA	131 - 135	132	A-->H
2HBWA	131 - 135	132	A-->L
2HBWA	131 - 135	132	A-->P
2HBWA	131 - 135	133	A-->G
2HBWA	131 - 135	133	A-->L
2HBWA	131 - 135	133	A-->P
2HBWA	131 - 135	133	A-->S
2HBWA	131 - 135	133	A-->T
2HBWA	131 - 135	134	F-->E

2HBWA	131	- 135	134	F-->I
2HBWA	131	- 135	134	F-->K
2HBWA	131	- 135	134	F-->L
2HBWA	131	- 135	134	F-->N
2HBWA	131	- 135	134	F-->P
2HBWA	131	- 135	134	F-->R
2HBWA	131	- 135	134	F-->S
2HBWA	131	- 135	135	V-->A
2HBWA	131	- 135	135	V-->D
2HBWA	131	- 135	135	V-->H
2HBWA	131	- 135	135	V-->Q
2HBWA	131	- 135	135	V-->S
2HBWA	131	- 135	135	V-->T
2C71A	585	- 590	585	P-->A
2C71A	585	- 590	585	P-->L
2C71A	585	- 590	585	P-->V
2C71A	585	- 590	586	T-->L
2C71A	585	- 590	590	N-->T
2OIZD	149	- 154	150	Y-->L
2OIZD	149	- 154	150	Y-->R
2OIZD	149	- 154	154	L-->A
1YQSA	102	- 106	102	V-->A
1YQSA	102	- 106	102	V-->E
1YQSA	102	- 106	102	V-->Q
1YQSA	102	- 106	102	V-->S
1YQSA	102	- 106	102	V-->T
1YQSA	102	- 106	103	R-->D

1YQSA	102 - 106	104	Q-->G
1YQSA	102 - 106	104	Q-->H
1YQSA	102 - 106	104	Q-->L
1YQSA	102 - 106	104	Q-->R
1YQSA	102 - 106	104	Q-->W
1YQSA	102 - 106	105	V-->T
1YQSA	102 - 106	106	M-->A
1YQSA	102 - 106	106	M-->D
1YQSA	102 - 106	106	M-->E
1YQSA	102 - 106	106	M-->F
1YQSA	102 - 106	106	M-->N
1YQSA	102 - 106	106	M-->P
1YQSA	102 - 106	106	M-->Q
1PSRA	57 - 61	57	V-->E
1PSRA	57 - 61	57	V-->K
1PSRA	57 - 61	57	V-->L
1PSRA	57 - 61	57	V-->P
1PSRA	57 - 61	58	F-->G
1PSRA	57 - 61	58	F-->I
1PSRA	57 - 61	58	F-->K
1PSRA	57 - 61	58	F-->L
1PSRA	57 - 61	58	F-->P
1PSRA	57 - 61	58	F-->S
1PSRA	57 - 61	58	F-->Y
1PSRA	57 - 61	59	E-->F
1PSRA	57 - 61	59	E-->G
1PSRA	57 - 61	59	E-->K

1PSRA	57	61	59	E-->L
1PSRA	57	61	59	E-->N
1PSRA	57	61	59	E-->P
1PSRA	57	61	59	E-->S
1PSRA	57	61	60	K-->D
1PSRA	57	61	60	K-->F
1PSRA	57	61	60	K-->G
1PSRA	57	61	60	K-->N
1PSRA	57	61	60	K-->P
1PSRA	57	61	60	K-->Q
1PSRA	57	61	60	K-->Y
1PSRA	57	61	61	K-->C
1PSRA	57	61	61	K-->E
1PSRA	57	61	61	K-->G
1PSRA	57	61	61	K-->N
1PSRA	57	61	61	K-->P
1PSRA	57	61	61	K-->R
1PSRA	57	61	61	K-->V
1D5TA	57	61	57	E-->A
1D5TA	57	61	58	T-->R
1D5TA	57	61	58	T-->W
1D5TA	57	61	59	E-->D
1D5TA	57	61	60	A-->L
1D5TA	57	61	60	A-->S
1UOWA	349	354	349	F-->N
2BOGX	118	122	118	L-->I
2BOGX	118	122	118	L-->P

2BOGX	118 - 122	118	L-->R
2BOGX	118 - 122	119	I-->F
2BOGX	118 - 122	119	I-->L
2BOGX	118 - 122	119	I-->P
2BOGX	118 - 122	120	S-->I
2BOGX	118 - 122	120	S-->M
2BOGX	118 - 122	120	S-->V
2BOGX	118 - 122	120	S-->Y
2BOGX	118 - 122	121	L-->P
2BOGX	118 - 122	122	M-->A
2BOGX	118 - 122	122	M-->D
2BOGX	118 - 122	122	M-->E
2BOGX	118 - 122	122	M-->F
2BOGX	118 - 122	122	M-->K
2BOGX	118 - 122	122	M-->L
2BOGX	118 - 122	122	M-->N
2BOGX	118 - 122	122	M-->P
2BOGX	118 - 122	122	M-->Q
2BOGX	118 - 122	122	M-->R
2BOGX	118 - 122	122	M-->S
2BOGX	118 - 122	122	M-->T
2HEUB	100 - 104	100	D-->A
2HEUB	100 - 104	100	D-->H
2HEUB	100 - 104	100	D-->L
2HEUB	100 - 104	100	D-->S
2HEUB	100 - 104	100	D-->T
2HEUB	100 - 104	101	Y-->D

2HEUB	100 - 104	101	Y-->G
2HEUB	100 - 104	101	Y-->K
2HEUB	100 - 104	101	Y-->Q
2HEUB	100 - 104	101	Y-->R
2HEUB	100 - 104	101	Y-->T
2HEUB	100 - 104	101	Y-->V
2HEUB	100 - 104	102	L-->I
2HEUB	100 - 104	102	L-->P
2HEUB	100 - 104	103	K-->D
2HEUB	100 - 104	103	K-->H
2HEUB	100 - 104	103	K-->N
2HEUB	100 - 104	103	K-->Q
2HEUB	100 - 104	104	R-->I
2HEUB	100 - 104	104	R-->L
2HEUB	100 - 104	104	R-->N
2HEUB	100 - 104	104	R-->P
2HEUB	100 - 104	104	R-->S
2HEUB	100 - 104	104	R-->Y
2HEUB	135 - 141	136	D-->V
2ANXA	96 - 100	96	K-->C
2ANXA	96 - 100	96	K-->D
2ANXA	96 - 100	96	K-->F
2ANXA	96 - 100	96	K-->G
2ANXA	96 - 100	96	K-->I
2ANXA	96 - 100	96	K-->M
2ANXA	96 - 100	96	K-->Q
2ANXA	96 - 100	96	K-->S

2ANXA	96 - 100	97	S-->E	
2ANXA	96 - 100	97	S-->G	
2ANXA	96 - 100	97	S-->I	
2ANXA	96 - 100	97	S-->L	
2ANXA	96 - 100	97	S-->M	
2ANXA	96 - 100	97	S-->V	
2ANXA	96 - 100	98	A-->D	
2ANXA	96 - 100	98	A-->L	
2ANXA	96 - 100	98	A-->S	
2ANXA	96 - 100	99	F-->A	
2ANXA	96 - 100	99	F-->D	
2ANXA	96 - 100	99	F-->K	
2ANXA	96 - 100	99	F-->L	
2ANXA	96 - 100	99	F-->P	
2ANXA	96 - 100	99	F-->Q	
2ANXA	96 - 100	99	F-->R	
2ANXA	96 - 100	99	F-->S	
2ANXA	96 - 100	100	A-->D	
2ANXA	96 - 100	100	A-->K	
2ANXA	96 - 100	100	A-->V	
2ANXA	103 - 109	108	L-->T	
1WUIS	32 - 37	32	I-->K	
1WUIS	32 - 37	32	I-->V	
1WUIS	32 - 37	33	D-->G	
1WUIS	32 - 37	33	D-->P	
1WUIS	32 - 37	34	T-->C	
1WUIS	32 - 37	34	T-->P	

1WUIS	32 - 37	36	I-->L
1WUIS	32 - 37	36	I-->Q
1WUIS	32 - 37	37	L-->P
1WUIS	32 - 37	37	L-->V
1WUIS	113 - 117	113	T-->H
1WUIS	113 - 117	113	T-->Y
1WUIS	113 - 117	114	C-->A
1WUIS	113 - 117	114	C-->L
1WUIS	113 - 117	114	C-->M
1WUIS	113 - 117	114	C-->R
1WUIS	113 - 117	114	C-->S
1WUIS	113 - 117	114	C-->T
1WUIS	113 - 117	115	A-->P
1WUIS	113 - 117	115	A-->Q
1WUIS	113 - 117	115	A-->R
1WUIS	113 - 117	115	A-->V
1WUIS	113 - 117	116	T-->A
1WUIS	113 - 117	116	T-->M
1WUIS	113 - 117	116	T-->V
1WUIS	113 - 117	117	F-->A
1WUIS	113 - 117	117	F-->D
1WUIS	113 - 117	117	F-->P
1WUIS	113 - 117	117	F-->S
1WUIS	113 - 117	117	F-->T
1WUIS	194 - 198	194	L-->A
1WUIS	194 - 198	194	L-->E
1WUIS	194 - 198	194	L-->F

1WUIS	194 - 198	194	L-->P
1WUIS	194 - 198	195	P-->G
1WUIS	194 - 198	195	P-->H
1WUIS	194 - 198	195	P-->R
1WUIS	194 - 198	195	P-->S
1WUIS	194 - 198	195	P-->V
1WUIS	194 - 198	196	H-->A
1WUIS	194 - 198	196	H-->D
1WUIS	194 - 198	196	H-->E
1WUIS	194 - 198	196	H-->G
1WUIS	194 - 198	196	H-->I
1WUIS	194 - 198	196	H-->K
1WUIS	194 - 198	196	H-->L
1WUIS	194 - 198	196	H-->M
1WUIS	194 - 198	196	H-->Q
1WUIS	194 - 198	196	H-->R
1WUIS	194 - 198	196	H-->S
1WUIS	194 - 198	196	H-->V
1WUIS	194 - 198	197	F-->A
1WUIS	194 - 198	197	F-->D
1WUIS	194 - 198	197	F-->G
1WUIS	194 - 198	197	F-->K
1WUIS	194 - 198	197	F-->L
1WUIS	194 - 198	197	F-->P
1WUIS	194 - 198	197	F-->Q
1WUIS	194 - 198	197	F-->S
1WUIS	194 - 198	197	F-->V

1WUIS	194 - 198	198	D-->N	
1WUIS	194 - 198	198	D-->P	
1WUIS	194 - 198	198	D-->S	
1WUIS	194 - 198	198	D-->T	
1KQPA	220 - 224	220	D-->C	
1KQPA	220 - 224	220	D-->E	
1KQPA	220 - 224	220	D-->G	
1KQPA	220 - 224	220	D-->I	
1KQPA	220 - 224	220	D-->K	
1KQPA	220 - 224	220	D-->L	
1KQPA	220 - 224	220	D-->P	
1KQPA	220 - 224	220	D-->T	
1KQPA	220 - 224	220	D-->Y	
1KQPA	220 - 224	221	E-->G	
1KQPA	220 - 224	221	E-->L	
1KQPA	220 - 224	221	E-->N	
1KQPA	220 - 224	222	T-->A	
1KQPA	220 - 224	222	T-->C	
1KQPA	220 - 224	222	T-->E	
1KQPA	220 - 224	222	T-->G	
1KQPA	220 - 224	222	T-->L	
1KQPA	220 - 224	222	T-->P	
1KQPA	220 - 224	222	T-->R	
1KQPA	220 - 224	222	T-->V	
1KQPA	220 - 224	222	T-->Y	
1KQPA	220 - 224	223	E-->G	
1KQPA	220 - 224	223	E-->I	

1KQPA	220	- 224	223	E-->K
1KQPA	220	- 224	223	E-->L
1KQPA	220	- 224	223	E-->N
1KQPA	220	- 224	223	E-->P
1KQPA	220	- 224	223	E-->R
1KQPA	220	- 224	223	E-->T
1KQPA	220	- 224	223	E-->V
1KQPA	220	- 224	224	L-->D
1KQPA	220	- 224	224	L-->E
1KQPA	220	- 224	224	L-->R
1KQPA	220	- 224	224	L-->S
1KQPA	220	- 224	224	L-->V
1RW1A	76	- 82	81	E-->V
1I27A	470	- 475	470	T-->A
1I27A	470	- 475	470	T-->D
1I27A	470	- 475	470	T-->L
1I27A	470	- 475	470	T-->R
1I27A	470	- 475	475	K-->F
1I27A	470	- 475	475	K-->I
1I27A	470	- 475	475	K-->Y
1G2YB	13	- 18	14	A-->L
1G2YB	13	- 18	14	A-->V
1G2YB	13	- 18	15	A-->D
1G2YB	13	- 18	15	A-->E
1G2YB	13	- 18	16	L-->T
1ZK4A	201	- 205	201	A-->H
1ZK4A	201	- 205	201	A-->N

1ZK4A	201 - 205	201	A-->R
1ZK4A	201 - 205	201	A-->S
1ZK4A	201 - 205	202	E-->A
1ZK4A	201 - 205	202	E-->L
1ZK4A	201 - 205	202	E-->P
1ZK4A	201 - 205	202	E-->V
1ZK4A	201 - 205	203	E-->G
1ZK4A	201 - 205	203	E-->N
1ZK4A	201 - 205	203	E-->P
1ZK4A	201 - 205	204	A-->P
1ZK4A	201 - 205	204	A-->V
1ZK4A	201 - 205	205	M-->A
1ZK4A	201 - 205	205	M-->D
1ZK4A	201 - 205	205	M-->E
1ZK4A	201 - 205	205	M-->H
1ZK4A	201 - 205	205	M-->K
1ZK4A	201 - 205	205	M-->L
1ZK4A	201 - 205	205	M-->P
1ZK4A	201 - 205	205	M-->Q
1ZK4A	201 - 205	205	M-->R
1ZK4A	201 - 205	205	M-->V
2I4AA	201 - 205	201	E-->A
2I4AA	201 - 205	201	E-->C
2I4AA	201 - 205	201	E-->D
2I4AA	201 - 205	201	E-->F
2I4AA	201 - 205	201	E-->L
2I4AA	201 - 205	201	E-->N

2I4AA	201	205	201	E-->V
2I4AA	201	205	201	E-->Y
2I4AA	201	205	202	T-->A
2I4AA	201	205	202	T-->D
2I4AA	201	205	202	T-->F
2I4AA	201	205	202	T-->I
2I4AA	201	205	202	T-->M
2I4AA	201	205	202	T-->N
2I4AA	201	205	203	P-->G
2I4AA	201	205	203	P-->I
2I4AA	201	205	203	P-->K
2I4AA	201	205	203	P-->N
2I4AA	201	205	203	P-->R
2I4AA	201	205	204	N-->A
2I4AA	201	205	204	N-->E
2I4AA	201	205	204	N-->I
2I4AA	201	205	204	N-->P
2I4AA	201	205	204	N-->S
2I4AA	201	205	205	A-->P
2I4AA	201	205	205	A-->S
2I4AA	201	205	205	A-->T
2I4AA	201	205	205	A-->V
1MN8A	8	14	14	G-->S
2RBKA	54	58	54	S-->A
2RBKA	54	58	54	S-->C
2RBKA	54	58	54	S-->D
2RBKA	54	58	54	S-->E

2RBKA	54 - 58	54	S-->I
2RBKA	54 - 58	54	S-->L
2RBKA	54 - 58	54	S-->P
2RBKA	54 - 58	54	S-->V
2RBKA	54 - 58	55	E-->C
2RBKA	54 - 58	55	E-->D
2RBKA	54 - 58	55	E-->H
2RBKA	54 - 58	55	E-->I
2RBKA	54 - 58	55	E-->K
2RBKA	54 - 58	55	E-->L
2RBKA	54 - 58	55	E-->N
2RBKA	54 - 58	55	E-->R
2RBKA	54 - 58	55	E-->S
2RBKA	54 - 58	55	E-->W
2RBKA	54 - 58	56	L-->A
2RBKA	54 - 58	56	L-->E
2RBKA	54 - 58	56	L-->P
2RBKA	54 - 58	57	Q-->A
2RBKA	54 - 58	57	Q-->K
2RBKA	54 - 58	57	Q-->L
2RBKA	54 - 58	57	Q-->N
2RBKA	54 - 58	57	Q-->P
2RBKA	54 - 58	57	Q-->Y
2RBKA	54 - 58	58	D-->A
2RBKA	54 - 58	58	D-->E
2RBKA	54 - 58	58	D-->G
2RBKA	54 - 58	58	D-->N

2RBKA	54	58	58	D-->S
2RBKA	54	58	58	D-->V
2RBKA	119	124	120	M-->Q
2RBKA	119	124	122	K-->E
2RBKA	139	143	139	F-->C
2RBKA	139	143	139	F-->E
2RBKA	139	143	139	F-->G
2RBKA	139	143	139	F-->H
2RBKA	139	143	139	F-->K
2RBKA	139	143	139	F-->L
2RBKA	139	143	139	F-->N
2RBKA	139	143	139	F-->S
2RBKA	139	143	139	F-->T
2RBKA	139	143	140	E-->A
2RBKA	139	143	140	E-->F
2RBKA	139	143	140	E-->I
2RBKA	139	143	140	E-->R
2RBKA	139	143	140	E-->S
2RBKA	139	143	140	E-->T
2RBKA	139	143	140	E-->Y
2RBKA	139	143	141	E-->A
2RBKA	139	143	141	E-->G
2RBKA	139	143	141	E-->I
2RBKA	139	143	141	E-->P
2RBKA	139	143	141	E-->Q
2RBKA	139	143	141	E-->T
2RBKA	139	143	141	E-->Y

2RBKA	139	143	142	A-->G
2RBKA	139	143	142	A-->I
2RBKA	139	143	142	A-->N
2RBKA	139	143	142	A-->P
2RBKA	139	143	142	A-->S
2RBKA	139	143	142	A-->T
2RBKA	139	143	142	A-->V
2RBKA	139	143	143	S-->A
2RBKA	139	143	143	S-->G
2RBKA	139	143	143	S-->K
2RBKA	139	143	143	S-->L
2RBKA	139	143	143	S-->N
2RBKA	139	143	143	S-->W
2RBKA	233	238	233	E-->P
2RBKA	233	238	236	K-->V
1TT8A	35	39	35	T-->K
1TT8A	35	39	35	T-->R
1TT8A	35	39	36	K-->A
1TT8A	35	39	36	K-->D
1TT8A	35	39	36	K-->I
1TT8A	35	39	36	K-->V
1TT8A	35	39	36	K-->Y
1TT8A	35	39	37	R-->K
1TT8A	35	39	37	R-->P
1TT8A	35	39	37	R-->S
1TT8A	35	39	37	R-->V
1TT8A	35	39	38	F-->G

1TT8A	35 - 39	38	F-->Q
1TT8A	35 - 39	38	F-->W
1TT8A	35 - 39	39	E-->A
1TT8A	35 - 39	39	E-->G
1TT8A	35 - 39	39	E-->K
1TT8A	35 - 39	39	E-->T
1Q6ZA	71 - 77	71	S-->D
1Q6ZA	169 - 174	171	S-->Q
1Q6ZA	169 - 174	173	H-->V
1Q6ZA	169 - 174	173	H-->W
1Q6ZA	169 - 173	169	P-->A
1Q6ZA	169 - 173	169	P-->E
1Q6ZA	169 - 173	169	P-->F
1Q6ZA	169 - 173	169	P-->G
1Q6ZA	169 - 173	169	P-->I
1Q6ZA	169 - 173	169	P-->K
1Q6ZA	169 - 173	169	P-->L
1Q6ZA	169 - 173	169	P-->R
1Q6ZA	169 - 173	169	P-->S
1Q6ZA	169 - 173	169	P-->T
1Q6ZA	169 - 173	169	P-->V
1Q6ZA	169 - 173	169	P-->Y
1Q6ZA	169 - 173	170	D-->E
1Q6ZA	169 - 173	170	D-->F
1Q6ZA	169 - 173	170	D-->L
1Q6ZA	169 - 173	170	D-->N
1Q6ZA	169 - 173	170	D-->T

1Q6ZA	169 - 173	170	D-->V
1Q6ZA	169 - 173	171	V-->A
1Q6ZA	169 - 173	171	V-->D
1Q6ZA	169 - 173	171	V-->G
1Q6ZA	169 - 173	171	V-->I
1Q6ZA	169 - 173	171	V-->L
1Q6ZA	169 - 173	171	V-->N
1Q6ZA	169 - 173	171	V-->P
1Q6ZA	169 - 173	171	V-->Q
1Q6ZA	169 - 173	171	V-->S
1Q6ZA	169 - 173	171	V-->T
1Q6ZA	169 - 173	171	V-->Y
1Q6ZA	169 - 173	172	D-->A
1Q6ZA	169 - 173	172	D-->F
1Q6ZA	169 - 173	172	D-->H
1Q6ZA	169 - 173	172	D-->I
1Q6ZA	169 - 173	172	D-->K
1Q6ZA	169 - 173	172	D-->L
1Q6ZA	169 - 173	172	D-->M
1Q6ZA	169 - 173	172	D-->P
1Q6ZA	169 - 173	172	D-->Q
1Q6ZA	169 - 173	172	D-->R
1Q6ZA	169 - 173	172	D-->Y
1Q6ZA	169 - 173	173	A-->C
1Q6ZA	169 - 173	173	A-->D
1Q6ZA	169 - 173	173	A-->F
1Q6ZA	169 - 173	173	A-->G

1Q6ZA	169 - 173	173	A-->I
1Q6ZA	169 - 173	173	A-->L
1Q6ZA	169 - 173	173	A-->P
1Q6ZA	169 - 173	173	A-->Q
1Q6ZA	169 - 173	173	A-->R
1Q6ZA	169 - 173	173	A-->V
1Q6ZA	302 - 307	305	A-->I
1Q6ZA	302 - 307	306	A-->D
1Q6ZA	302 - 307	306	A-->F
1GKMA	171 - 177	177	V-->G
1GKMA	189 - 194	189	E-->P
1GKMA	189 - 194	189	E-->T
1GKMA	189 - 194	190	G-->V
1GKMA	189 - 194	191	L-->P
1GKMA	189 - 194	192	A-->K
1GKMA	189 - 194	192	A-->V
1GKMA	189 - 194	193	L-->V
1GKMA	189 - 194	194	L-->S
1GKMA	189 - 194	194	L-->T
1GKMA	189 - 194	194	L-->V
1GKMA	189 - 193	189	E-->A
1GKMA	189 - 193	189	E-->D
1GKMA	189 - 193	189	E-->G
1GKMA	189 - 193	189	E-->I
1GKMA	189 - 193	189	E-->L
1GKMA	189 - 193	189	E-->N
1GKMA	189 - 193	189	E-->P

1GKMA	189 - 193	189	E-->R	
1GKMA	189 - 193	189	E-->S	
1GKMA	189 - 193	189	E-->T	
1GKMA	189 - 193	189	E-->V	
1GKMA	189 - 193	189	E-->Y	
1GKMA	189 - 193	190	V-->D	
1GKMA	189 - 193	190	V-->F	
1GKMA	189 - 193	190	V-->G	
1GKMA	189 - 193	190	V-->I	
1GKMA	189 - 193	190	V-->K	
1GKMA	189 - 193	190	V-->L	
1GKMA	189 - 193	190	V-->P	
1GKMA	189 - 193	190	V-->S	
1GKMA	189 - 193	190	V-->T	
1GKMA	189 - 193	190	V-->Y	
1GKMA	189 - 193	191	S-->A	
1GKMA	189 - 193	191	S-->E	
1GKMA	189 - 193	191	S-->F	
1GKMA	189 - 193	191	S-->G	
1GKMA	189 - 193	191	S-->H	
1GKMA	189 - 193	191	S-->I	
1GKMA	189 - 193	191	S-->K	
1GKMA	189 - 193	191	S-->L	
1GKMA	189 - 193	191	S-->N	
1GKMA	189 - 193	191	S-->P	
1GKMA	189 - 193	191	S-->R	
1GKMA	189 - 193	191	S-->T	

1GKMA	189	193	191	S-->V
1GKMA	189	193	191	S-->Y
1GKMA	189	193	192	L-->C
1GKMA	189	193	192	L-->G
1GKMA	189	193	192	L-->I
1GKMA	189	193	192	L-->K
1GKMA	189	193	192	L-->P
1GKMA	189	193	192	L-->S
1GKMA	189	193	192	L-->V
1GKMA	189	193	193	S-->D
1GKMA	189	193	193	S-->E
1GKMA	189	193	193	S-->G
1GKMA	189	193	193	S-->I
1GKMA	189	193	193	S-->L
1GKMA	189	193	193	S-->N
1GKMA	189	193	193	S-->P
1GKMA	189	193	193	S-->Q
1GKMA	189	193	193	S-->T
1GKMA	189	193	193	S-->V
1GKMA	189	193	193	S-->W
1GKMA	189	193	193	S-->Y
1GA6A	138	143	138	E-->V
1GA6A	138	143	139	A-->T
1GA6A	138	143	142	N-->D
1GA6A	138	143	142	N-->V
1GA6A	138	143	143	A-->Y
1GA6A	245	249	245	S-->V

1GA6A	245 - 249	246	W-->F	
1GA6A	245 - 249	246	W-->R	
1GA6A	245 - 249	246	W-->S	
1GA6A	245 - 249	246	W-->T	
1GA6A	245 - 249	246	W-->V	
1GA6A	245 - 249	246	W-->Y	
1GA6A	245 - 249	247	Q-->L	
1GA6A	245 - 249	247	Q-->P	
1GA6A	245 - 249	247	Q-->V	
1GA6A	245 - 249	248	S-->C	
1GA6A	245 - 249	248	S-->P	
1GA6A	245 - 249	248	S-->T	
1GA6A	245 - 249	249	V-->I	
2CWSA	108 - 112	108	S-->A	
2CWSA	108 - 112	108	S-->E	
2CWSA	108 - 112	108	S-->F	
2CWSA	108 - 112	108	S-->G	
2CWSA	108 - 112	108	S-->I	
2CWSA	108 - 112	108	S-->K	
2CWSA	108 - 112	108	S-->M	
2CWSA	108 - 112	108	S-->P	
2CWSA	108 - 112	108	S-->T	
2CWSA	108 - 112	108	S-->W	
2CWSA	108 - 112	108	S-->Y	
2CWSA	108 - 112	109	A-->C	
2CWSA	108 - 112	109	A-->D	
2CWSA	108 - 112	109	A-->F	

2CWSA	108 - 112	109	A-->G	
2CWSA	108 - 112	109	A-->K	
2CWSA	108 - 112	109	A-->L	
2CWSA	108 - 112	109	A-->M	
2CWSA	108 - 112	109	A-->N	
2CWSA	108 - 112	109	A-->P	
2CWSA	108 - 112	109	A-->Q	
2CWSA	108 - 112	109	A-->T	
2CWSA	108 - 112	110	N-->A	
2CWSA	108 - 112	110	N-->C	
2CWSA	108 - 112	110	N-->D	
2CWSA	108 - 112	110	N-->E	
2CWSA	108 - 112	110	N-->F	
2CWSA	108 - 112	110	N-->G	
2CWSA	108 - 112	110	N-->I	
2CWSA	108 - 112	110	N-->K	
2CWSA	108 - 112	110	N-->L	
2CWSA	108 - 112	110	N-->M	
2CWSA	108 - 112	110	N-->Q	
2CWSA	108 - 112	110	N-->R	
2CWSA	108 - 112	110	N-->S	
2CWSA	108 - 112	110	N-->T	
2CWSA	108 - 112	110	N-->V	
2CWSA	108 - 112	110	N-->Y	
2CWSA	108 - 112	111	L-->F	
2CWSA	108 - 112	111	L-->I	
2CWSA	108 - 112	111	L-->P	

2CWSA	108 - 112	111	L-->Q
2CWSA	108 - 112	111	L-->R
2CWSA	108 - 112	111	L-->T
2CWSA	108 - 112	111	L-->V
2CWSA	108 - 112	112	G-->A
2CWSA	108 - 112	112	G-->H
2CWSA	108 - 112	112	G-->I
2CWSA	108 - 112	112	G-->L
2CWSA	108 - 112	112	G-->P
2CWSA	108 - 112	112	G-->S
2CWSA	108 - 112	112	G-->T
2CWSA	108 - 112	112	G-->V
2CWSA	108 - 112	112	G-->W
2CWSA	108 - 112	112	G-->Y
2CWSA	270 - 274	270	A-->I
2CWSA	270 - 274	271	G-->T
2CWSA	270 - 274	272	W-->A
2CWSA	270 - 274	272	W-->I
2CWSA	270 - 274	272	W-->L
2CWSA	270 - 274	272	W-->N
2CWSA	270 - 274	272	W-->P
2CWSA	270 - 274	272	W-->S
2CWSA	270 - 274	272	W-->V
2CWSA	270 - 274	272	W-->Y
2CWSA	270 - 274	273	K-->G
2CWSA	270 - 274	273	K-->S
2CWSA	270 - 274	274	N-->D

2CWSA	270 - 274	274	N-->F
2CWSA	270 - 274	274	N-->G
2CWSA	270 - 274	274	N-->K
2CWSA	270 - 274	274	N-->P
2CWSA	270 - 274	274	N-->Y
1O7JA	36 - 42	42	A-->H
1O7JA	44 - 48	44	P-->A
1O7JA	44 - 48	44	P-->D
1O7JA	44 - 48	44	P-->E
1O7JA	44 - 48	44	P-->F
1O7JA	44 - 48	44	P-->G
1O7JA	44 - 48	44	P-->K
1O7JA	44 - 48	44	P-->L
1O7JA	44 - 48	44	P-->N
1O7JA	44 - 48	44	P-->S
1O7JA	44 - 48	44	P-->V
1O7JA	44 - 48	44	P-->W
1O7JA	44 - 48	45	E-->F
1O7JA	44 - 48	45	E-->G
1O7JA	44 - 48	45	E-->L
1O7JA	44 - 48	45	E-->N
1O7JA	44 - 48	45	E-->P
1O7JA	44 - 48	45	E-->Q
1O7JA	44 - 48	45	E-->R
1O7JA	44 - 48	45	E-->S
1O7JA	44 - 48	45	E-->V
1O7JA	44 - 48	46	V-->A

1O7JA	44 - 48	46	V-->D
1O7JA	44 - 48	46	V-->E
1O7JA	44 - 48	46	V-->F
1O7JA	44 - 48	46	V-->G
1O7JA	44 - 48	46	V-->H
1O7JA	44 - 48	46	V-->I
1O7JA	44 - 48	46	V-->L
1O7JA	44 - 48	46	V-->M
1O7JA	44 - 48	46	V-->P
1O7JA	44 - 48	46	V-->Q
1O7JA	44 - 48	46	V-->R
1O7JA	44 - 48	46	V-->S
1O7JA	44 - 48	47	K-->C
1O7JA	44 - 48	47	K-->E
1O7JA	44 - 48	47	K-->G
1O7JA	44 - 48	47	K-->L
1O7JA	44 - 48	47	K-->N
1O7JA	44 - 48	47	K-->R
1O7JA	44 - 48	47	K-->S
1O7JA	44 - 48	47	K-->V
1O7JA	44 - 48	48	K-->A
1O7JA	44 - 48	48	K-->D
1O7JA	44 - 48	48	K-->F
1O7JA	44 - 48	48	K-->H
1O7JA	44 - 48	48	K-->I
1O7JA	44 - 48	48	K-->L
1O7JA	44 - 48	48	K-->P

1O7JA	44 - 48	48	K-->S
1O7JA	44 - 48	48	K-->T
1O7JA	44 - 48	48	K-->V
1O7JA	44 - 48	48	K-->Y
1EB6A	104 - 109	105	I-->E
1EB6A	104 - 109	106	Y-->S
1EB6A	104 - 109	108	S-->G
1EB6A	104 - 108	104	Y-->A
1EB6A	104 - 108	104	Y-->C
1EB6A	104 - 108	104	Y-->D
1EB6A	104 - 108	104	Y-->E
1EB6A	104 - 108	104	Y-->F
1EB6A	104 - 108	104	Y-->G
1EB6A	104 - 108	104	Y-->H
1EB6A	104 - 108	104	Y-->I
1EB6A	104 - 108	104	Y-->K
1EB6A	104 - 108	104	Y-->L
1EB6A	104 - 108	104	Y-->M
1EB6A	104 - 108	104	Y-->P
1EB6A	104 - 108	104	Y-->S
1EB6A	104 - 108	104	Y-->V
1EB6A	104 - 108	104	Y-->W
1EB6A	104 - 108	105	D-->A
1EB6A	104 - 108	105	D-->C
1EB6A	104 - 108	105	D-->G
1EB6A	104 - 108	105	D-->H
1EB6A	104 - 108	105	D-->K

1EB6A	104	108	105	D-->M
1EB6A	104	108	105	D-->N
1EB6A	104	108	105	D-->R
1EB6A	104	108	105	D-->T
1EB6A	104	108	105	D-->W
1EB6A	104	108	106	A-->C
1EB6A	104	108	106	A-->D
1EB6A	104	108	106	A-->H
1EB6A	104	108	106	A-->L
1EB6A	104	108	106	A-->P
1EB6A	104	108	106	A-->Q
1EB6A	104	108	106	A-->S
1EB6A	104	108	106	A-->T
1EB6A	104	108	106	A-->V
1EB6A	104	108	107	A-->D
1EB6A	104	108	107	A-->E
1EB6A	104	108	107	A-->M
1EB6A	104	108	107	A-->N
1EB6A	104	108	107	A-->P
1EB6A	104	108	107	A-->Q
1EB6A	104	108	107	A-->R
1EB6A	104	108	107	A-->S
1EB6A	104	108	107	A-->T
1EB6A	104	108	108	T-->E
1EB6A	104	108	108	T-->G
1EB6A	104	108	108	T-->N
1EB6A	104	108	108	T-->S

1EB6A	156 - 160	156	A-->H
1EB6A	156 - 160	156	A-->R
1EB6A	156 - 160	156	A-->V
1EB6A	156 - 160	157	Q-->A
1EB6A	156 - 160	157	Q-->C
1EB6A	156 - 160	157	Q-->E
1EB6A	156 - 160	157	Q-->F
1EB6A	156 - 160	157	Q-->G
1EB6A	156 - 160	157	Q-->H
1EB6A	156 - 160	157	Q-->I
1EB6A	156 - 160	157	Q-->K
1EB6A	156 - 160	157	Q-->L
1EB6A	156 - 160	157	Q-->P
1EB6A	156 - 160	157	Q-->S
1EB6A	156 - 160	157	Q-->T
1EB6A	156 - 160	157	Q-->V
1EB6A	156 - 160	157	Q-->Y
1EB6A	156 - 160	158	D-->A
1EB6A	156 - 160	158	D-->G
1EB6A	156 - 160	158	D-->I
1EB6A	156 - 160	158	D-->L
1EB6A	156 - 160	158	D-->M
1EB6A	156 - 160	158	D-->P
1EB6A	156 - 160	158	D-->R
1EB6A	156 - 160	158	D-->S
1EB6A	156 - 160	158	D-->T
1EB6A	156 - 160	158	D-->V

1EB6A	156	160	159	A-->E
1EB6A	156	160	159	A-->F
1EB6A	156	160	159	A-->I
1EB6A	156	160	159	A-->L
1EB6A	156	160	159	A-->N
1EB6A	156	160	159	A-->S
1EB6A	156	160	159	A-->T
1EB6A	156	160	160	L-->D
1EB6A	156	160	160	L-->G
1EB6A	156	160	160	L-->K
1JFBA	74	79	74	A-->V
1JFBA	74	79	76	G-->V
1JFBA	74	79	77	K-->A
1JFBA	74	79	77	K-->P
1JFBA	74	79	78	Q-->I
1JFBA	74	79	78	Q-->T
1P1XA	107	111	107	Y-->I
1P1XA	107	111	107	Y-->P
1P1XA	107	111	108	R-->K
1P1XA	107	111	108	R-->S
1P1XA	107	111	111	M-->E
1P1XA	107	111	111	M-->F
1P1XA	107	111	111	M-->I
1P1XA	107	111	111	M-->L
1P1XA	107	111	111	M-->N
1P1XA	107	111	111	M-->P
1P1XA	107	111	111	M-->S

1P1XA	107	111	111	M-->T
1P1XA	107	111	111	M-->V
1P1XA	107	111	111	M-->Y
1UG6A	32	36	32	I-->G
1UG6A	32	36	33	W-->A
1UG6A	32	36	33	W-->H
1UG6A	32	36	33	W-->I
1UG6A	32	36	33	W-->N
1UG6A	32	36	33	W-->P
1UG6A	32	36	33	W-->S
1UG6A	32	36	33	W-->V
1UG6A	32	36	34	D-->K
1UG6A	32	36	36	F-->I
1UG6A	32	36	36	F-->N
1UG6A	32	36	36	F-->R
1UG6A	268	272	268	D-->A
1UG6A	268	272	268	D-->F
1UG6A	268	272	268	D-->G
1UG6A	268	272	268	D-->H
1UG6A	268	272	268	D-->I
1UG6A	268	272	268	D-->K
1UG6A	268	272	268	D-->N
1UG6A	268	272	268	D-->P
1UG6A	268	272	268	D-->Q
1UG6A	268	272	268	D-->R
1UG6A	268	272	268	D-->S
1UG6A	268	272	268	D-->T

1UG6A	268	- 272	268	D-->V
1UG6A	268	- 272	269	L-->A
1UG6A	268	- 272	269	L-->D
1UG6A	268	- 272	269	L-->E
1UG6A	268	- 272	269	L-->F
1UG6A	268	- 272	269	L-->G
1UG6A	268	- 272	269	L-->I
1UG6A	268	- 272	269	L-->M
1UG6A	268	- 272	269	L-->N
1UG6A	268	- 272	269	L-->P
1UG6A	268	- 272	269	L-->Q
1UG6A	268	- 272	269	L-->R
1UG6A	268	- 272	269	L-->T
1UG6A	268	- 272	269	L-->V
1UG6A	268	- 272	270	E-->A
1UG6A	268	- 272	270	E-->D
1UG6A	268	- 272	270	E-->G
1UG6A	268	- 272	270	E-->I
1UG6A	268	- 272	270	E-->K
1UG6A	268	- 272	270	E-->L
1UG6A	268	- 272	270	E-->N
1UG6A	268	- 272	270	E-->P
1UG6A	268	- 272	270	E-->Q
1UG6A	268	- 272	270	E-->R
1UG6A	268	- 272	270	E-->T
1UG6A	268	- 272	270	E-->V
1UG6A	268	- 272	270	E-->Y

1UG6A	268 - 272	271	L-->A
1UG6A	268 - 272	271	L-->D
1UG6A	268 - 272	271	L-->E
1UG6A	268 - 272	271	L-->G
1UG6A	268 - 272	271	L-->I
1UG6A	268 - 272	271	L-->K
1UG6A	268 - 272	271	L-->N
1UG6A	268 - 272	271	L-->P
1UG6A	268 - 272	271	L-->Q
1UG6A	268 - 272	271	L-->R
1UG6A	268 - 272	271	L-->T
1UG6A	268 - 272	271	L-->V
1UG6A	268 - 272	271	L-->W
1UG6A	268 - 272	272	V-->E
1UG6A	268 - 272	272	V-->G
1UG6A	268 - 272	272	V-->H
1UG6A	268 - 272	272	V-->I
1UG6A	268 - 272	272	V-->K
1UG6A	268 - 272	272	V-->L
1UG6A	268 - 272	272	V-->P
1UG6A	268 - 272	272	V-->S
1UG6A	268 - 272	272	V-->T
1MWQA	14 - 19	14	L-->D
1K4IA	14 - 18	14	P-->A
1K4IA	14 - 18	17	T-->A
1IXHA	61 - 67	61	D-->S
1IXHA	114 - 119	115	A-->P

1IXHA	114 - 119	115	A-->Y	
1IXHA	114 - 119	116	I-->A	
1IXHA	114 - 119	118	K-->P	
1IXHA	114 - 119	119	L-->Y	
1IXHA	196 - 200	196	Y-->F	
1IXHA	196 - 200	196	Y-->M	
1IXHA	196 - 200	196	Y-->S	
1IXHA	196 - 200	196	Y-->T	
1IXHA	196 - 200	197	A-->I	
1IXHA	196 - 200	197	A-->K	
1IXHA	196 - 200	197	A-->V	
1IXHA	196 - 200	198	Y-->G	
1IXHA	196 - 200	198	Y-->I	
1IXHA	196 - 200	198	Y-->M	
1IXHA	196 - 200	198	Y-->R	
1IXHA	196 - 200	198	Y-->T	
1IXHA	196 - 200	199	A-->D	
1IXHA	196 - 200	199	A-->E	
1IXHA	196 - 200	199	A-->G	
1IXHA	196 - 200	199	A-->R	
1IXHA	196 - 200	200	K-->G	
1IXHA	196 - 200	200	K-->N	
1IXHA	196 - 200	200	K-->P	
1IXHA	196 - 200	200	K-->S	
1IXHA	196 - 200	200	K-->T	
1IXHA	196 - 200	200	K-->V	
1IXHA	285 - 290	289	N-->G	

2V8TA	105	109	105	G-->I
2V8TA	105	109	105	G-->L
2V8TA	105	109	105	G-->Q
2V8TA	105	109	105	G-->S
2V8TA	105	109	105	G-->T
2V8TA	105	109	105	G-->V
2V8TA	105	109	106	F-->A
2V8TA	105	109	106	F-->C
2V8TA	105	109	106	F-->D
2V8TA	105	109	106	F-->E
2V8TA	105	109	106	F-->G
2V8TA	105	109	106	F-->H
2V8TA	105	109	106	F-->K
2V8TA	105	109	106	F-->L
2V8TA	105	109	106	F-->N
2V8TA	105	109	106	F-->R
2V8TA	105	109	106	F-->S
2V8TA	105	109	106	F-->V
2V8TA	105	109	106	F-->Y
2V8TA	105	109	107	A-->D
2V8TA	105	109	107	A-->E
2V8TA	105	109	107	A-->F
2V8TA	105	109	107	A-->G
2V8TA	105	109	107	A-->K
2V8TA	105	109	107	A-->L
2V8TA	105	109	107	A-->N
2V8TA	105	109	107	A-->S

2V8TA	105 - 109	107	A-->T
2V8TA	105 - 109	107	A-->W
2V8TA	105 - 109	107	A-->Y
2V8TA	105 - 109	108	K-->F
2V8TA	105 - 109	108	K-->G
2V8TA	105 - 109	108	K-->I
2V8TA	105 - 109	108	K-->L
2V8TA	105 - 109	108	K-->M
2V8TA	105 - 109	108	K-->T
2V8TA	105 - 109	108	K-->V
2V8TA	105 - 109	109	D-->A
2V8TA	105 - 109	109	D-->E
2V8TA	105 - 109	109	D-->N
2V8TA	105 - 109	109	D-->R
2V8TA	105 - 109	109	D-->S
2V8TA	105 - 109	109	D-->T
2V8TA	105 - 109	109	D-->V
2V8TA	105 - 109	109	D-->W
2V8TA	105 - 109	109	D-->Y
1M1QA	5 - 10	6	S-->A
1M1QA	5 - 10	9	H-->V
1M1QA	5 - 9	5	S-->A
1M1QA	5 - 9	5	S-->D
1M1QA	5 - 9	5	S-->G
1M1QA	5 - 9	5	S-->I
1M1QA	5 - 9	5	S-->M
1M1QA	5 - 9	5	S-->P

1M1QA	5 - 9	5	S-->R
1M1QA	5 - 9	5	S-->T
1M1QA	5 - 9	5	S-->V
1M1QA	5 - 9	6	A-->D
1M1QA	5 - 9	6	A-->E
1M1QA	5 - 9	6	A-->G
1M1QA	5 - 9	6	A-->I
1M1QA	5 - 9	6	A-->L
1M1QA	5 - 9	6	A-->M
1M1QA	5 - 9	6	A-->N
1M1QA	5 - 9	6	A-->P
1M1QA	5 - 9	6	A-->Q
1M1QA	5 - 9	6	A-->S
1M1QA	5 - 9	6	A-->T
1M1QA	5 - 9	6	A-->Y
1M1QA	5 - 9	7	S-->A
1M1QA	5 - 9	7	S-->D
1M1QA	5 - 9	7	S-->E
1M1QA	5 - 9	7	S-->G
1M1QA	5 - 9	7	S-->I
1M1QA	5 - 9	7	S-->K
1M1QA	5 - 9	7	S-->L
1M1QA	5 - 9	7	S-->M
1M1QA	5 - 9	7	S-->P
1M1QA	5 - 9	7	S-->Q
1M1QA	5 - 9	7	S-->R
1M1QA	5 - 9	7	S-->T

1M1QA	5 - 9	7	S-->V
1M1QA	5 - 9	8	V-->A
1M1QA	5 - 9	8	V-->D
1M1QA	5 - 9	8	V-->E
1M1QA	5 - 9	8	V-->F
1M1QA	5 - 9	8	V-->K
1M1QA	5 - 9	8	V-->P
1M1QA	5 - 9	8	V-->R
1M1QA	5 - 9	8	V-->S
1M1QA	5 - 9	8	V-->T
1M1QA	5 - 9	8	V-->Y
1M1QA	5 - 9	9	L-->A
1M1QA	5 - 9	9	L-->D
1M1QA	5 - 9	9	L-->G
1M1QA	5 - 9	9	L-->H
1M1QA	5 - 9	9	L-->I
1M1QA	5 - 9	9	L-->K
1M1QA	5 - 9	9	L-->N
1M1QA	5 - 9	9	L-->P
1M1QA	5 - 9	9	L-->Q
1M1QA	5 - 9	9	L-->R
1M1QA	5 - 9	9	L-->S
1M1QA	5 - 9	9	L-->T
1M1QA	5 - 9	9	L-->V
1XG0C	4 - 9	4	A-->P
1XG0C	103 - 108	103	S-->Q
1XG0C	103 - 108	103	S-->R

1XG0C	103 - 108	106	E-->W
1XG0C	103 - 108	107	D-->V
1XG0C	103 - 108	108	R-->L
2E4TA	71 - 75	71	Y-->D
2E4TA	71 - 75	71	Y-->E
2E4TA	71 - 75	71	Y-->F
2E4TA	71 - 75	71	Y-->G
2E4TA	71 - 75	71	Y-->L
2E4TA	71 - 75	71	Y-->N
2E4TA	71 - 75	71	Y-->P
2E4TA	71 - 75	71	Y-->R
2E4TA	71 - 75	71	Y-->S
2E4TA	71 - 75	71	Y-->T
2E4TA	71 - 75	71	Y-->V
2E4TA	71 - 75	72	L-->A
2E4TA	71 - 75	72	L-->C
2E4TA	71 - 75	72	L-->F
2E4TA	71 - 75	72	L-->I
2E4TA	71 - 75	72	L-->P
2E4TA	71 - 75	72	L-->Q
2E4TA	71 - 75	72	L-->T
2E4TA	71 - 75	72	L-->V
2E4TA	71 - 75	73	L-->D
2E4TA	71 - 75	73	L-->E
2E4TA	71 - 75	73	L-->I
2E4TA	71 - 75	73	L-->K
2E4TA	71 - 75	73	L-->N

2E4TA	71	-	75	73	L-->P
2E4TA	71	-	75	73	L-->V
2E4TA	71	-	75	74	E-->C
2E4TA	71	-	75	74	E-->D
2E4TA	71	-	75	74	E-->F
2E4TA	71	-	75	74	E-->K
2E4TA	71	-	75	74	E-->P
2E4TA	71	-	75	74	E-->Q
2E4TA	71	-	75	74	E-->R
2E4TA	71	-	75	74	E-->T
2E4TA	71	-	75	74	E-->V
2E4TA	71	-	75	75	D-->A
2E4TA	71	-	75	75	D-->E
2E4TA	71	-	75	75	D-->F
2E4TA	71	-	75	75	D-->G
2E4TA	71	-	75	75	D-->K
2E4TA	71	-	75	75	D-->P
2E4TA	71	-	75	75	D-->Q
2E4TA	71	-	75	75	D-->S
2E4TA	187	-	193	190	W-->S
2E4TA	235	-	240	236	A-->P
2E4TA	235	-	240	237	A-->T
2E4TA	327	-	331	331	W-->I
2E4TA	327	-	331	331	W-->Q
1UFYA	109	-	114	113	R-->I
1UFYA	109	-	114	113	R-->M
1WY3A	55	-	60	56	S-->I

1WY3A	55 - 60	59	A-->S
1MJ5A	159 - 163	159	G-->D
1MJ5A	159 - 163	159	G-->E
1MJ5A	159 - 163	159	G-->F
1MJ5A	159 - 163	159	G-->H
1MJ5A	159 - 163	159	G-->M
1MJ5A	159 - 163	159	G-->P
1MJ5A	159 - 163	159	G-->T
1MJ5A	159 - 163	159	G-->Y
1MJ5A	159 - 163	160	E-->A
1MJ5A	159 - 163	160	E-->C
1MJ5A	159 - 163	160	E-->D
1MJ5A	159 - 163	160	E-->F
1MJ5A	159 - 163	160	E-->G
1MJ5A	159 - 163	160	E-->H
1MJ5A	159 - 163	160	E-->I
1MJ5A	159 - 163	160	E-->K
1MJ5A	159 - 163	160	E-->L
1MJ5A	159 - 163	160	E-->P
1MJ5A	159 - 163	160	E-->R
1MJ5A	159 - 163	160	E-->S
1MJ5A	159 - 163	160	E-->T
1MJ5A	159 - 163	160	E-->V
1MJ5A	159 - 163	160	E-->Y
1MJ5A	159 - 163	161	E-->A
1MJ5A	159 - 163	161	E-->D
1MJ5A	159 - 163	161	E-->F

1MJ5A	159 - 163	161	E-->K
1MJ5A	159 - 163	161	E-->L
1MJ5A	159 - 163	161	E-->N
1MJ5A	159 - 163	161	E-->P
1MJ5A	159 - 163	161	E-->Q
1MJ5A	159 - 163	161	E-->S
1MJ5A	159 - 163	161	E-->T
1MJ5A	159 - 163	161	E-->V
1MJ5A	159 - 163	161	E-->W
1MJ5A	159 - 163	161	E-->Y
1MJ5A	159 - 163	162	L-->A
1MJ5A	159 - 163	162	L-->E
1MJ5A	159 - 163	162	L-->F
1MJ5A	159 - 163	162	L-->I
1MJ5A	159 - 163	162	L-->K
1MJ5A	159 - 163	162	L-->M
1MJ5A	159 - 163	162	L-->N
1MJ5A	159 - 163	162	L-->R
1MJ5A	159 - 163	162	L-->T
1MJ5A	159 - 163	162	L-->V
1MJ5A	159 - 163	162	L-->Y
1MJ5A	159 - 163	163	V-->C
1MJ5A	159 - 163	163	V-->E
1MJ5A	159 - 163	163	V-->F
1MJ5A	159 - 163	163	V-->G
1MJ5A	159 - 163	163	V-->H
1MJ5A	159 - 163	163	V-->I

1MJ5A	159 -	163	163	V-->K
1MJ5A	159 -	163	163	V-->L
1MJ5A	159 -	163	163	V-->N
1MJ5A	159 -	163	163	V-->P
1MJ5A	159 -	163	163	V-->Q
1MJ5A	159 -	163	163	V-->R
1MJ5A	159 -	163	163	V-->S
1MJ5A	159 -	163	163	V-->T
1MJ5A	159 -	163	163	V-->Y
7A3HA	5 -	9	5	V-->D
7A3HA	5 -	9	5	V-->E
7A3HA	5 -	9	5	V-->K
7A3HA	5 -	9	6	V-->K
7A3HA	5 -	9	6	V-->L
7A3HA	5 -	9	6	V-->T
7A3HA	5 -	9	7	E-->A
7A3HA	5 -	9	7	E-->C
7A3HA	5 -	9	7	E-->D
7A3HA	5 -	9	7	E-->F
7A3HA	5 -	9	7	E-->G
7A3HA	5 -	9	7	E-->L
7A3HA	5 -	9	7	E-->N
7A3HA	5 -	9	7	E-->Q
7A3HA	5 -	9	7	E-->T
7A3HA	5 -	9	7	E-->V
7A3HA	5 -	9	7	E-->W
7A3HA	5 -	9	8	E-->A

7A3HA	5 - 9	8	E-->G
7A3HA	5 - 9	8	E-->I
7A3HA	5 - 9	8	E-->K
7A3HA	5 - 9	8	E-->L
7A3HA	5 - 9	8	E-->N
7A3HA	5 - 9	8	E-->R
7A3HA	5 - 9	8	E-->S
7A3HA	5 - 9	8	E-->T
7A3HA	5 - 9	8	E-->V
7A3HA	5 - 9	8	E-->Y
7A3HA	5 - 9	9	H-->A
7A3HA	5 - 9	9	H-->D
7A3HA	5 - 9	9	H-->E
7A3HA	5 - 9	9	H-->F
7A3HA	5 - 9	9	H-->G
7A3HA	5 - 9	9	H-->I
7A3HA	5 - 9	9	H-->N
7A3HA	5 - 9	9	H-->P
7A3HA	5 - 9	9	H-->R
7A3HA	5 - 9	9	H-->V
7A3HA	5 - 9	9	H-->Y
7A3HA	182 - 186	183	H-->E
7A3HA	182 - 186	183	H-->I
7A3HA	182 - 186	183	H-->M
7A3HA	182 - 186	184	H-->D
7A3HA	182 - 186	184	H-->E
7A3HA	182 - 186	184	H-->F

7A3HA	182 - 186	184	H-->G
7A3HA	182 - 186	184	H-->N
7A3HA	182 - 186	184	H-->P
7A3HA	182 - 186	184	H-->S
7A3HA	182 - 186	184	H-->Y
7A3HA	182 - 186	185	A-->G
7A3HA	182 - 186	185	A-->S
7A3HA	182 - 186	186	A-->N
7A3HA	182 - 186	186	A-->V
2FVYA	96 - 100	96	R-->A
2FVYA	96 - 100	96	R-->D
2FVYA	96 - 100	96	R-->E
2FVYA	96 - 100	96	R-->F
2FVYA	96 - 100	96	R-->I
2FVYA	96 - 100	96	R-->K
2FVYA	96 - 100	96	R-->L
2FVYA	96 - 100	96	R-->N
2FVYA	96 - 100	96	R-->P
2FVYA	96 - 100	96	R-->S
2FVYA	96 - 100	96	R-->V
2FVYA	96 - 100	97	K-->A
2FVYA	96 - 100	97	K-->C
2FVYA	96 - 100	97	K-->G
2FVYA	96 - 100	97	K-->I
2FVYA	96 - 100	97	K-->L
2FVYA	96 - 100	97	K-->P
2FVYA	96 - 100	97	K-->R

2FVYA	96	100	97	K-->T
2FVYA	96	100	97	K-->W
2FVYA	96	100	98	A-->D
2FVYA	96	100	98	A-->E
2FVYA	96	100	98	A-->F
2FVYA	96	100	98	A-->G
2FVYA	96	100	98	A-->L
2FVYA	96	100	98	A-->N
2FVYA	96	100	98	A-->Y
2FVYA	96	100	99	L-->A
2FVYA	96	100	99	L-->D
2FVYA	96	100	99	L-->G
2FVYA	96	100	99	L-->M
2FVYA	96	100	99	L-->N
2FVYA	96	100	100	D-->A
2FVYA	96	100	100	D-->F
2FVYA	96	100	100	D-->G
2FVYA	96	100	100	D-->I
2FVYA	96	100	100	D-->K
2FVYA	96	100	100	D-->N
2FVYA	96	100	100	D-->R
2FVYA	96	100	100	D-->S
2FVYA	96	100	100	D-->T
1N4WA	140	145	143	M-->F
1N4WA	140	145	145	D-->V
1N4WA	162	167	164	W-->S
1N4WA	232	238	235	A-->W

1IQZA	16	20	16	A-->G
1IQZA	16	20	16	A-->L
1IQZA	16	20	16	A-->T
1IQZA	16	20	17	C-->A
1IQZA	16	20	17	C-->D
1IQZA	16	20	17	C-->E
1IQZA	16	20	17	C-->G
1IQZA	16	20	17	C-->H
1IQZA	16	20	17	C-->I
1IQZA	16	20	17	C-->K
1IQZA	16	20	17	C-->L
1IQZA	16	20	17	C-->M
1IQZA	16	20	17	C-->N
1IQZA	16	20	17	C-->P
1IQZA	16	20	17	C-->R
1IQZA	16	20	17	C-->S
1IQZA	16	20	17	C-->T
1IQZA	16	20	17	C-->V
1IQZA	16	20	17	C-->Y
1IQZA	16	20	18	G-->D
1IQZA	16	20	18	G-->E
1IQZA	16	20	18	G-->P
1IQZA	16	20	18	G-->S
1IQZA	16	20	19	A-->I
1IQZA	16	20	19	A-->K
1IQZA	16	20	19	A-->L
1IQZA	16	20	19	A-->M

1IQZA	16 - 20	19	A-->P
1IQZA	16 - 20	20	A-->C
1IQZA	16 - 20	20	A-->D
1IQZA	16 - 20	20	A-->H
1IQZA	16 - 20	20	A-->N
1IQZA	16 - 20	20	A-->S
1IQZA	16 - 20	20	A-->T
1IQZA	16 - 20	20	A-->V
2NRLA	3 - 8	3	D-->L
2NRLA	3 - 8	4	F-->S
2NRLA	3 - 8	6	A-->G
2NRLA	3 - 8	6	A-->L
2NRLA	3 - 8	7	V-->R
2NRLA	3 - 8	8	L-->S
2NRLA	3 - 8	8	L-->V
2PVBA	60 - 64	60	E-->A
2PVBA	60 - 64	60	E-->G
2PVBA	60 - 64	60	E-->H
2PVBA	60 - 64	60	E-->I
2PVBA	60 - 64	60	E-->L
2PVBA	60 - 64	60	E-->P
2PVBA	60 - 64	60	E-->R
2PVBA	60 - 64	60	E-->S
2PVBA	60 - 64	61	D-->F
2PVBA	60 - 64	61	D-->G
2PVBA	60 - 64	61	D-->I
2PVBA	60 - 64	61	D-->K

2PVBA	60 - 64	61	D-->L
2PVBA	60 - 64	61	D-->P
2PVBA	60 - 64	61	D-->Q
2PVBA	60 - 64	61	D-->R
2PVBA	60 - 64	61	D-->T
2PVBA	60 - 64	61	D-->V
2PVBA	60 - 64	61	D-->Y
2PVBA	60 - 64	62	E-->A
2PVBA	60 - 64	62	E-->C
2PVBA	60 - 64	62	E-->F
2PVBA	60 - 64	62	E-->I
2PVBA	60 - 64	62	E-->L
2PVBA	60 - 64	62	E-->S
2PVBA	60 - 64	62	E-->T
2PVBA	60 - 64	62	E-->V
2PVBA	60 - 64	63	L-->A
2PVBA	60 - 64	63	L-->D
2PVBA	60 - 64	63	L-->F
2PVBA	60 - 64	63	L-->G
2PVBA	60 - 64	63	L-->I
2PVBA	60 - 64	63	L-->R
2PVBA	60 - 64	63	L-->T
2PVBA	60 - 64	64	K-->D
2PVBA	60 - 64	64	K-->G
2PVBA	60 - 64	64	K-->I
2PVBA	60 - 64	64	K-->L
2PVBA	60 - 64	64	K-->M

2PVBA	60 - 64	64	K-->P
2PVBA	60 - 64	64	K-->Q
2PVBA	60 - 64	64	K-->R
2PVBA	60 - 64	64	K-->S
2PVBA	60 - 64	64	K-->T
2PVBA	60 - 64	64	K-->V
1OEWA	113 - 117	113	S-->A
1OEWA	113 - 117	113	S-->D
1OEWA	113 - 117	113	S-->F
1OEWA	113 - 117	113	S-->I
1OEWA	113 - 117	113	S-->L
1OEWA	113 - 117	113	S-->M
1OEWA	113 - 117	113	S-->N
1OEWA	113 - 117	113	S-->T
1OEWA	113 - 117	113	S-->V
1OEWA	113 - 117	114	S-->D
1OEWA	113 - 117	114	S-->E
1OEWA	113 - 117	114	S-->F
1OEWA	113 - 117	114	S-->H
1OEWA	113 - 117	114	S-->T
1OEWA	113 - 117	115	F-->C
1OEWA	113 - 117	115	F-->I
1OEWA	113 - 117	115	F-->K
1OEWA	113 - 117	115	F-->N
1OEWA	113 - 117	115	F-->R
1OEWA	113 - 117	115	F-->T
1OEWA	113 - 117	115	F-->V

1OEWA	113 - 117	116	T-->D
1OEWA	113 - 117	116	T-->I
1OEWA	113 - 117	116	T-->L
1OEWA	113 - 117	116	T-->R
1OEWA	113 - 117	117	E-->A
1OEWA	113 - 117	117	E-->F
1OEWA	113 - 117	117	E-->I
1OEWA	113 - 117	117	E-->K
1OEWA	113 - 117	117	E-->L
1OEWA	113 - 117	117	E-->M
1OEWA	113 - 117	117	E-->N
1OEWA	113 - 117	117	E-->P
1OEWA	113 - 117	117	E-->S
1OEWA	113 - 117	117	E-->V
1OEWA	113 - 117	117	E-->Y
1OEWA	143 - 147	143	F-->D
1OEWA	143 - 147	143	F-->E
1OEWA	143 - 147	143	F-->L
1OEWA	143 - 147	143	F-->N
1OEWA	143 - 147	143	F-->V
1OEWA	143 - 147	144	F-->A
1OEWA	143 - 147	144	F-->E
1OEWA	143 - 147	144	F-->G
1OEWA	143 - 147	144	F-->L
1OEWA	143 - 147	144	F-->T
1OEWA	143 - 147	145	D-->G
1OEWA	143 - 147	145	D-->N

1OEWA	143 - 147	145	D-->S
1OEWA	143 - 147	146	N-->G
1OEWA	143 - 147	146	N-->I
1OEWA	143 - 147	146	N-->P
1OEWA	143 - 147	146	N-->S
1OEWA	143 - 147	147	A-->D
1OEWA	143 - 147	147	A-->G
1OEWA	143 - 147	147	A-->P
2BW4A	105 - 111	110	A-->S
2BW4A	142 - 147	144	W-->K
2BW4A	142 - 147	145	H-->I
2BW4A	142 - 147	147	T-->L
2BW4A	308 - 312	308	L-->C
2BW4A	308 - 312	308	L-->E
2BW4A	308 - 312	308	L-->I
2BW4A	308 - 312	309	I-->D
2BW4A	308 - 312	309	I-->G
2BW4A	308 - 312	309	I-->K
2BW4A	308 - 312	309	I-->L
2BW4A	308 - 312	309	I-->N
2BW4A	308 - 312	309	I-->P
2BW4A	308 - 312	309	I-->Q
2BW4A	308 - 312	309	I-->S
2BW4A	308 - 312	309	I-->T
2BW4A	308 - 312	310	E-->L
2BW4A	308 - 312	310	E-->Y
2BW4A	308 - 312	311	A-->C

2BW4A	308 - 312	311	A-->D
2BW4A	308 - 312	311	A-->I
2BW4A	308 - 312	311	A-->Y
2BW4A	308 - 312	312	F-->A
2BW4A	308 - 312	312	F-->D
2BW4A	308 - 312	312	F-->E
2BW4A	308 - 312	312	F-->G
2BW4A	308 - 312	312	F-->K
2BW4A	308 - 312	312	F-->M
2BW4A	308 - 312	312	F-->Q
2BW4A	308 - 312	312	F-->R
2BW4A	308 - 312	312	F-->S
2BW4A	308 - 312	312	F-->T
2BW4A	308 - 312	312	F-->Y
1VYRA	187 - 192	189	H-->P
1VYRA	187 - 192	190	Q-->P
1VYRA	187 - 192	191	F-->P
1VYRA	324 - 328	324	R-->C
1VYRA	324 - 328	324	R-->D
1VYRA	324 - 328	324	R-->F
1VYRA	324 - 328	324	R-->I
1VYRA	324 - 328	324	R-->K
1VYRA	324 - 328	324	R-->M
1VYRA	324 - 328	324	R-->P
1VYRA	324 - 328	324	R-->Q
1VYRA	324 - 328	324	R-->S
1VYRA	324 - 328	324	R-->V

1VYRA	324 - 328	324	R-->Y
1VYRA	324 - 328	325	D-->E
1VYRA	324 - 328	325	D-->P
1VYRA	324 - 328	325	D-->Y
1VYRA	324 - 328	326	Y-->D
1VYRA	324 - 328	326	Y-->F
1VYRA	324 - 328	326	Y-->I
1VYRA	324 - 328	326	Y-->N
1VYRA	324 - 328	326	Y-->Q
1VYRA	324 - 328	326	Y-->V
1VYRA	324 - 328	327	I-->K
1VYRA	324 - 328	327	I-->N
1VYRA	324 - 328	327	I-->P
1VYRA	324 - 328	327	I-->R
1VYRA	324 - 328	327	I-->Y
1VYRA	324 - 328	328	A-->D
1VYRA	324 - 328	328	A-->F
1VYRA	324 - 328	328	A-->H
1VYRA	324 - 328	328	A-->K
1VYRA	324 - 328	328	A-->L
1G66A	119 - 124	123	N-->C
1G66A	119 - 124	124	M-->A
1I1WA	6 - 11	8	Q-->T
1I1WA	143 - 147	143	V-->A
1I1WA	143 - 147	143	V-->E
1I1WA	143 - 147	143	V-->F
1I1WA	143 - 147	143	V-->I

1I1WA	143 - 147	143	V-->K
1I1WA	143 - 147	143	V-->L
1I1WA	143 - 147	143	V-->P
1I1WA	143 - 147	143	V-->R
1I1WA	143 - 147	143	V-->T
1I1WA	143 - 147	144	F-->A
1I1WA	143 - 147	144	F-->D
1I1WA	143 - 147	144	F-->E
1I1WA	143 - 147	144	F-->G
1I1WA	143 - 147	144	F-->I
1I1WA	143 - 147	144	F-->K
1I1WA	143 - 147	144	F-->L
1I1WA	143 - 147	144	F-->Q
1I1WA	143 - 147	144	F-->R
1I1WA	143 - 147	144	F-->S
1I1WA	143 - 147	144	F-->T
1I1WA	143 - 147	144	F-->V
1I1WA	143 - 147	145	L-->D
1I1WA	143 - 147	145	L-->G
1I1WA	143 - 147	145	L-->N
1I1WA	143 - 147	145	L-->Q
1I1WA	143 - 147	145	L-->S
1I1WA	143 - 147	145	L-->V
1I1WA	143 - 147	146	N-->D
1I1WA	143 - 147	146	N-->E
1I1WA	143 - 147	146	N-->F
1I1WA	143 - 147	146	N-->G

1I1WA	143 - 147	146	N-->I
1I1WA	143 - 147	146	N-->K
1I1WA	143 - 147	146	N-->P
1I1WA	143 - 147	146	N-->R
1I1WA	143 - 147	146	N-->S
1I1WA	143 - 147	147	V-->D
1I1WA	143 - 147	147	V-->F
1I1WA	143 - 147	147	V-->K
1I1WA	143 - 147	147	V-->M
1I1WA	143 - 147	147	V-->N
1I1WA	143 - 147	147	V-->P
1I1WA	143 - 147	147	V-->Q
1I1WA	143 - 147	147	V-->T
1I1WA	143 - 147	147	V-->W
2DDXA	83 - 87	83	V-->A
2DDXA	83 - 87	83	V-->D
2DDXA	83 - 87	83	V-->E
2DDXA	83 - 87	83	V-->F
2DDXA	83 - 87	83	V-->I
2DDXA	83 - 87	83	V-->L
2DDXA	83 - 87	83	V-->M
2DDXA	83 - 87	83	V-->N
2DDXA	83 - 87	83	V-->P
2DDXA	83 - 87	84	D-->A
2DDXA	83 - 87	84	D-->E
2DDXA	83 - 87	84	D-->G
2DDXA	83 - 87	84	D-->I

2DDXA	83 - 87	84	D-->K
2DDXA	83 - 87	84	D-->L
2DDXA	83 - 87	84	D-->N
2DDXA	83 - 87	84	D-->P
2DDXA	83 - 87	84	D-->Q
2DDXA	83 - 87	84	D-->R
2DDXA	83 - 87	84	D-->T
2DDXA	83 - 87	84	D-->V
2DDXA	83 - 87	84	D-->W
2DDXA	83 - 87	84	D-->Y
2DDXA	83 - 87	85	A-->D
2DDXA	83 - 87	85	A-->F
2DDXA	83 - 87	85	A-->I
2DDXA	83 - 87	85	A-->K
2DDXA	83 - 87	85	A-->L
2DDXA	83 - 87	85	A-->N
2DDXA	83 - 87	85	A-->P
2DDXA	83 - 87	85	A-->S
2DDXA	83 - 87	85	A-->T
2DDXA	83 - 87	85	A-->V
2DDXA	83 - 87	85	A-->Y
2DDXA	83 - 87	86	V-->A
2DDXA	83 - 87	86	V-->D
2DDXA	83 - 87	86	V-->E
2DDXA	83 - 87	86	V-->F
2DDXA	83 - 87	86	V-->H
2DDXA	83 - 87	86	V-->I

2DDXA	83 - 87	86	V-->L	
2DDXA	83 - 87	86	V-->N	
2DDXA	83 - 87	86	V-->P	
2DDXA	83 - 87	86	V-->Q	
2DDXA	83 - 87	86	V-->R	
2DDXA	83 - 87	86	V-->S	
2DDXA	83 - 87	86	V-->T	
2DDXA	83 - 87	87	A-->D	
2DDXA	83 - 87	87	A-->E	
2DDXA	83 - 87	87	A-->F	
2DDXA	83 - 87	87	A-->G	
2DDXA	83 - 87	87	A-->H	
2DDXA	83 - 87	87	A-->I	
2DDXA	83 - 87	87	A-->N	
2DDXA	83 - 87	87	A-->P	
2DDXA	83 - 87	87	A-->Q	
2DDXA	83 - 87	87	A-->V	
2DDXA	83 - 87	87	A-->W	
2DDXA	83 - 87	87	A-->Y	
1MUWA	217 - 221	217	V-->A	
1MUWA	217 - 221	217	V-->Q	
1MUWA	217 - 221	218	G-->F	
1MUWA	217 - 221	218	G-->H	
1MUWA	217 - 221	218	G-->I	
1MUWA	217 - 221	218	G-->K	
1MUWA	217 - 221	218	G-->L	
1MUWA	217 - 221	218	G-->N	

1MUWA	217 - 221	218	G-->S
1MUWA	217 - 221	219	H-->C
1MUWA	217 - 221	219	H-->L
1MUWA	217 - 221	219	H-->Q
1MUWA	217 - 221	219	H-->R
1MUWA	217 - 221	219	H-->S
1MUWA	217 - 221	219	H-->T
1MUWA	217 - 221	219	H-->V
1MUWA	217 - 221	219	H-->Y
1MUWA	217 - 221	220	E-->P
1MUWA	217 - 221	220	E-->R
1MUWA	217 - 221	220	E-->S
1MUWA	217 - 221	221	Q-->A
1MUWA	217 - 221	221	Q-->F
1MUWA	217 - 221	221	Q-->I
1MUWA	217 - 221	221	Q-->L
1MUWA	346 - 351	347	Q-->V
1MUWA	346 - 351	351	A-->L
1MUWA	361 - 366	361	V-->K
1MUWA	361 - 366	362	D-->A
1MUWA	361 - 366	362	D-->N
1MUWA	361 - 366	362	D-->P
1MUWA	361 - 366	362	D-->Q
1MUWA	361 - 366	362	D-->T
1MUWA	361 - 366	362	D-->V
1MUWA	361 - 366	363	A-->I
1MUWA	361 - 366	364	A-->I

1MUWA	361	- 366	365	A-->K
1MUWA	361	- 366	365	A-->S
1MUWA	361	- 366	366	A-->D
1MUWA	361	- 366	366	A-->S
1X8QA	175	- 181	176	D-->H
2O7AA	85	- 90	88	Y-->G
2O7AA	85	- 90	88	Y-->L
2O7AA	85	- 90	89	D-->W
2O7AA	108	- 112	108	E-->A
2O7AA	108	- 112	108	E-->D
2O7AA	108	- 112	108	E-->G
2O7AA	108	- 112	108	E-->I
2O7AA	108	- 112	108	E-->K
2O7AA	108	- 112	108	E-->P
2O7AA	108	- 112	108	E-->R
2O7AA	108	- 112	108	E-->S
2O7AA	108	- 112	108	E-->T
2O7AA	108	- 112	108	E-->V
2O7AA	108	- 112	109	T-->A
2O7AA	108	- 112	109	T-->C
2O7AA	108	- 112	109	T-->D
2O7AA	108	- 112	109	T-->F
2O7AA	108	- 112	109	T-->G
2O7AA	108	- 112	109	T-->I
2O7AA	108	- 112	109	T-->N
2O7AA	108	- 112	109	T-->P
2O7AA	108	- 112	109	T-->R

2O7AA	108 - 112	109	T-->S
2O7AA	108 - 112	109	T-->V
2O7AA	108 - 112	110	G-->E
2O7AA	108 - 112	110	G-->I
2O7AA	108 - 112	110	G-->Q
2O7AA	108 - 112	110	G-->R
2O7AA	108 - 112	110	G-->T
2O7AA	108 - 112	110	G-->V
2O7AA	108 - 112	110	G-->W
2O7AA	108 - 112	110	G-->Y
2O7AA	108 - 112	111	V-->D
2O7AA	108 - 112	111	V-->E
2O7AA	108 - 112	111	V-->G
2O7AA	108 - 112	111	V-->H
2O7AA	108 - 112	111	V-->I
2O7AA	108 - 112	111	V-->K
2O7AA	108 - 112	111	V-->L
2O7AA	108 - 112	111	V-->M
2O7AA	108 - 112	111	V-->N
2O7AA	108 - 112	111	V-->R
2O7AA	108 - 112	111	V-->S
2O7AA	108 - 112	111	V-->T
2O7AA	108 - 112	112	A-->D
2O7AA	108 - 112	112	A-->E
2O7AA	108 - 112	112	A-->G
2O7AA	108 - 112	112	A-->I
2O7AA	108 - 112	112	A-->K

2O7AA	108 - 112	112	A-->L
2O7AA	108 - 112	112	A-->P
2O7AA	108 - 112	112	A-->S
2O7AA	108 - 112	112	A-->T
2O7AA	108 - 112	112	A-->V
2O7AA	137 - 141	137	R-->L
2O7AA	137 - 141	137	R-->T
2O7AA	137 - 141	138	W-->G
2O7AA	137 - 141	140	N-->L
2O7AA	137 - 141	141	Q-->G
2O7AA	137 - 141	141	Q-->K
2JFRA	131 - 137	131	V-->S
1N55A	96 - 101	100	T-->S
1N55A	131 - 135	131	L-->K
1N55A	131 - 135	132	Q-->E
1N55A	131 - 135	132	Q-->G
1N55A	131 - 135	132	Q-->P
1N55A	131 - 135	132	Q-->R
1N55A	131 - 135	132	Q-->Y
1N55A	131 - 135	133	Q-->F
1N55A	131 - 135	133	Q-->K
1N55A	131 - 135	133	Q-->R
1N55A	131 - 135	133	Q-->S
1N55A	131 - 135	133	Q-->T
1N55A	131 - 135	134	R-->D
1N55A	131 - 135	134	R-->G
1N55A	131 - 135	135	E-->G

1N55A	131 - 135	135	E-->P
1N55A	131 - 135	135	E-->R
1N55A	131 - 135	135	E-->T
1N55A	200 - 205	202	V-->K
1N55A	200 - 205	203	A-->I
1N55A	200 - 205	203	A-->L
1N55A	200 - 205	205	K-->W
1N55A	219 - 223	219	A-->D
1N55A	219 - 223	219	A-->E
1N55A	219 - 223	219	A-->F
1N55A	219 - 223	219	A-->G
1N55A	219 - 223	219	A-->I
1N55A	219 - 223	219	A-->L
1N55A	219 - 223	219	A-->R
1N55A	219 - 223	219	A-->V
1N55A	219 - 223	219	A-->Y
1N55A	219 - 223	220	A-->D
1N55A	219 - 223	220	A-->E
1N55A	219 - 223	220	A-->F
1N55A	219 - 223	220	A-->G
1N55A	219 - 223	220	A-->H
1N55A	219 - 223	220	A-->K
1N55A	219 - 223	220	A-->L
1N55A	219 - 223	220	A-->T
1N55A	219 - 223	221	T-->A
1N55A	219 - 223	221	T-->D
1N55A	219 - 223	221	T-->G

1N55A	219 - 223	221	T-->M
1N55A	219 - 223	221	T-->P
1N55A	219 - 223	221	T-->R
1N55A	219 - 223	221	T-->V
1N55A	219 - 223	222	L-->E
1N55A	219 - 223	222	L-->G
1N55A	219 - 223	222	L-->H
1N55A	219 - 223	222	L-->I
1N55A	219 - 223	222	L-->K
1N55A	219 - 223	222	L-->R
1N55A	219 - 223	222	L-->V
1N55A	219 - 223	222	L-->Y
1N55A	219 - 223	223	Y-->A
1N55A	219 - 223	223	Y-->D
1N55A	219 - 223	223	Y-->E
1N55A	219 - 223	223	Y-->F
1N55A	219 - 223	223	Y-->G
1N55A	219 - 223	223	Y-->H
1N55A	219 - 223	223	Y-->I
1N55A	219 - 223	223	Y-->L
1N55A	219 - 223	223	Y-->P
1N55A	219 - 223	223	Y-->Q
1N55A	219 - 223	223	Y-->R
1N55A	219 - 223	223	Y-->S
1N55A	219 - 223	223	Y-->T
1N55A	219 - 223	223	Y-->V
1NWZA	44 - 50	46	E-->Q

2H5CA	231 - 238	234	I-->P
1IUAA	12 - 16	12	P-->A
1IUAA	12 - 16	12	P-->D
1IUAA	12 - 16	12	P-->G
1IUAA	12 - 16	12	P-->I
1IUAA	12 - 16	12	P-->L
1IUAA	12 - 16	12	P-->N
1IUAA	12 - 16	12	P-->T
1IUAA	12 - 16	12	P-->Y
1IUAA	12 - 16	13	T-->A
1IUAA	12 - 16	13	T-->D
1IUAA	12 - 16	13	T-->E
1IUAA	12 - 16	13	T-->F
1IUAA	12 - 16	13	T-->G
1IUAA	12 - 16	13	T-->H
1IUAA	12 - 16	13	T-->I
1IUAA	12 - 16	13	T-->L
1IUAA	12 - 16	13	T-->N
1IUAA	12 - 16	13	T-->Q
1IUAA	12 - 16	13	T-->R
1IUAA	12 - 16	13	T-->V
1IUAA	12 - 16	13	T-->Y
1IUAA	12 - 16	14	A-->G
1IUAA	12 - 16	14	A-->H
1IUAA	12 - 16	14	A-->I
1IUAA	12 - 16	14	A-->R
1IUAA	12 - 16	14	A-->S

1IUAA	12	16	14	A-->T
1IUAA	12	16	15	I-->D
1IUAA	12	16	15	I-->G
1IUAA	12	16	15	I-->L
1IUAA	12	16	15	I-->P
1IUAA	12	16	15	I-->Q
1IUAA	12	16	15	I-->S
1IUAA	12	16	15	I-->T
1IUAA	12	16	15	I-->V
1IUAA	12	16	16	A-->E
1IUAA	12	16	16	A-->F
1IUAA	12	16	16	A-->M
1IUAA	12	16	16	A-->R
1IUAA	12	16	16	A-->T
1IUAA	12	16	16	A-->V
1IUAA	12	16	16	A-->Y
1GCIA	6	10	6	W-->A
1GCIA	6	10	6	W-->D
1GCIA	6	10	6	W-->E
1GCIA	6	10	6	W-->F
1GCIA	6	10	6	W-->H
1GCIA	6	10	6	W-->N
1GCIA	6	10	6	W-->P
1GCIA	6	10	6	W-->Q
1GCIA	6	10	6	W-->V
1GCIA	6	10	6	W-->Y
1GCIA	6	10	7	G-->D

1GCIA	6	10	7	G-->H
1GCIA	6	10	7	G-->L
1GCIA	6	10	7	G-->N
1GCIA	6	10	7	G-->R
1GCIA	6	10	7	G-->V
1GCIA	6	10	8	I-->S
1GCIA	6	10	8	I-->T
1GCIA	6	10	10	R-->D
1GCIA	6	10	10	R-->W
1GCIA	13	18	13	A-->L
1R6JA	226	230	226	S-->G
1R6JA	226	230	226	S-->I
1R6JA	226	230	226	S-->K
1R6JA	226	230	226	S-->L
1R6JA	226	230	226	S-->R
1R6JA	226	230	226	S-->T
1R6JA	226	230	227	A-->I
1R6JA	226	230	227	A-->M
1R6JA	226	230	227	A-->P
1R6JA	226	230	227	A-->R
1R6JA	226	230	227	A-->T
1R6JA	226	230	227	A-->Y
1R6JA	226	230	228	A-->C
1R6JA	226	230	228	A-->E
1R6JA	226	230	228	A-->G
1R6JA	226	230	228	A-->K
1R6JA	226	230	228	A-->T

1R6JA	226 - 230	228	A-->V
1R6JA	226 - 230	228	A-->Y
1R6JA	226 - 230	229	R-->A
1R6JA	226 - 230	229	R-->F
1R6JA	226 - 230	229	R-->G
1R6JA	226 - 230	229	R-->P
1R6JA	226 - 230	230	N-->A
1R6JA	226 - 230	230	N-->F
1R6JA	226 - 230	230	N-->G
1R6JA	226 - 230	230	N-->L
1R6JA	226 - 230	230	N-->T
2VB1A	80 - 84	80	C-->A
2VB1A	80 - 84	80	C-->F
2VB1A	80 - 84	80	C-->G
2VB1A	80 - 84	80	C-->K
2VB1A	80 - 84	80	C-->L
2VB1A	80 - 84	80	C-->M
2VB1A	80 - 84	80	C-->N
2VB1A	80 - 84	80	C-->Q
2VB1A	80 - 84	80	C-->V
2VB1A	80 - 84	80	C-->Y
2VB1A	80 - 84	82	A-->F
2VB1A	80 - 84	82	A-->G
2VB1A	80 - 84	82	A-->H
2VB1A	80 - 84	82	A-->P
2VB1A	80 - 84	82	A-->R
2VB1A	80 - 84	82	A-->T

2VB1A	80 - 84	83	L-->G
2VB1A	80 - 84	83	L-->I
2VB1A	80 - 84	83	L-->T
2VB1A	80 - 84	84	L-->C
2VB1A	80 - 84	84	L-->G
2VB1A	80 - 84	84	L-->K
2VB1A	80 - 84	84	L-->N
2VB1A	80 - 84	84	L-->Q
2VB1A	80 - 84	84	L-->S
2VB1A	80 - 84	84	L-->T
1ZOFB	108 - 112	108	S-->D
1ZOFB	108 - 112	108	S-->I
1ZOFB	108 - 112	108	S-->K
1ZOFB	108 - 112	108	S-->L
1ZOFB	108 - 112	108	S-->N
1ZOFB	108 - 112	108	S-->T
1ZOFB	108 - 112	108	S-->V
1ZOFB	108 - 112	109	I-->D
1ZOFB	108 - 112	109	I-->S
1ZOFB	108 - 112	109	I-->W
1ZOFB	108 - 112	110	S-->D
1ZOFB	108 - 112	110	S-->F
1ZOFB	108 - 112	110	S-->N
1ZOFB	108 - 112	110	S-->P
1ZOFB	108 - 112	110	S-->Q
1ZOFB	108 - 112	110	S-->T
1ZOFB	108 - 112	111	R-->A

1ZOFB	108	112	111	R-->C
1ZOFB	108	112	111	R-->F
1ZOFB	108	112	111	R-->G
1ZOFB	108	112	111	R-->I
1ZOFB	108	112	111	R-->K
1ZOFB	108	112	111	R-->L
1ZOFB	108	112	111	R-->N
1ZOFB	108	112	111	R-->P
1ZOFB	108	112	111	R-->S
1ZOFB	108	112	111	R-->W
1ZOFB	108	112	112	D-->F
1ZOFB	108	112	112	D-->H
1ZOFB	108	112	112	D-->M
1ZOFB	108	112	112	D-->N
1ZOFB	108	112	112	D-->P
1ZOFB	108	112	112	D-->S
1ZOFB	108	112	112	D-->T
1ZOFB	108	112	112	D-->V
1ZOFB	108	112	112	D-->W
2NUAE	220	225	220	G-->N
2NUAE	220	225	221	N-->Q
2NUAE	220	225	222	A-->L
2NUAE	220	225	224	F-->P
2NUAE	220	225	224	F-->W
2NUAE	220	225	225	R-->T
2NUAE	227	231	227	P-->A
2NUAE	227	231	227	P-->D

2NUAE	227 - 231	227	P-->K
2NUAE	227 - 231	227	P-->S
2NUAE	227 - 231	227	P-->V
2NUAE	227 - 231	227	P-->W
2NUAE	227 - 231	228	D-->A
2NUAE	227 - 231	228	D-->E
2NUAE	227 - 231	228	D-->H
2NUAE	227 - 231	228	D-->L
2NUAE	227 - 231	228	D-->N
2NUAE	227 - 231	228	D-->P
2NUAE	227 - 231	228	D-->T
2NUAE	227 - 231	228	D-->V
2NUAE	227 - 231	228	D-->Y
2NUAE	227 - 231	229	L-->E
2NUAE	227 - 231	229	L-->G
2NUAE	227 - 231	229	L-->R
2NUAE	227 - 231	229	L-->S
2NUAE	227 - 231	229	L-->Y
2NUAE	227 - 231	230	R-->A
2NUAE	227 - 231	230	R-->D
2NUAE	227 - 231	230	R-->E
2NUAE	227 - 231	230	R-->F
2NUAE	227 - 231	230	R-->I
2NUAE	227 - 231	230	R-->K
2NUAE	227 - 231	230	R-->L
2NUAE	227 - 231	230	R-->P
2NUAE	227 - 231	230	R-->S

2NUAE	227 - 231	230	R-->T
2NUAE	227 - 231	231	E-->A
2NUAE	227 - 231	231	E-->F
2NUAE	227 - 231	231	E-->G
2NUAE	227 - 231	231	E-->K
2NUAE	227 - 231	231	E-->L
2NUAE	227 - 231	231	E-->N
2NUAE	227 - 231	231	E-->P
2NUAE	227 - 231	231	E-->R
2NUAE	227 - 231	231	E-->V
2OCFD	78 - 83	81	M-->D
2OCFD	78 - 83	81	M-->G
2OCFD	78 - 83	81	M-->P
1XHUB	93 - 97	93	K-->A
1XHUB	93 - 97	93	K-->C
1XHUB	93 - 97	93	K-->D
1XHUB	93 - 97	93	K-->E
1XHUB	93 - 97	93	K-->F
1XHUB	93 - 97	93	K-->G
1XHUB	93 - 97	93	K-->M
1XHUB	93 - 97	93	K-->N
1XHUB	93 - 97	93	K-->P
1XHUB	93 - 97	93	K-->S
1XHUB	93 - 97	93	K-->T
1XHUB	93 - 97	93	K-->V
1XHUB	93 - 97	93	K-->Y
1XHUB	93 - 97	94	A-->D

1XHUB	93 - 97	94	A-->G	
1XHUB	93 - 97	94	A-->I	
1XHUB	93 - 97	94	A-->K	
1XHUB	93 - 97	95	A-->G	
1XHUB	93 - 97	95	A-->I	
1XHUB	93 - 97	95	A-->K	
1XHUB	93 - 97	95	A-->L	
1XHUB	93 - 97	95	A-->W	
1XHUB	93 - 97	95	A-->Y	
1XHUB	93 - 97	96	T-->C	
1XHUB	93 - 97	96	T-->E	
1XHUB	93 - 97	96	T-->F	
1XHUB	93 - 97	96	T-->G	
1XHUB	93 - 97	96	T-->H	
1XHUB	93 - 97	96	T-->P	
1XHUB	93 - 97	96	T-->S	
1XHUB	93 - 97	96	T-->V	
1XHUB	93 - 97	96	T-->Y	
1XHUB	93 - 97	97	E-->D	
1XHUB	93 - 97	97	E-->F	
1XHUB	93 - 97	97	E-->G	
1XHUB	93 - 97	97	E-->H	
1XHUB	93 - 97	97	E-->K	
1XHUB	93 - 97	97	E-->L	
1XHUB	93 - 97	97	E-->N	
1XHUB	93 - 97	97	E-->P	
1XHUB	93 - 97	97	E-->S	

1XHUB	93 - 97	97	E-->V
1GLEG	152 - 156	152	R-->E
1GLEG	152 - 156	152	R-->G
1GLEG	152 - 156	152	R-->P
1GLEG	152 - 156	152	R-->Q
1GLEG	152 - 156	152	R-->S
1GLEG	152 - 156	152	R-->Y
1GLEG	152 - 156	153	E-->A
1GLEG	152 - 156	153	E-->I
1GLEG	152 - 156	153	E-->M
1GLEG	152 - 156	153	E-->R
1GLEG	152 - 156	153	E-->T
1GLEG	152 - 156	153	E-->W
1GLEG	152 - 156	154	R-->A
1GLEG	152 - 156	154	R-->D
1GLEG	152 - 156	154	R-->E
1GLEG	152 - 156	154	R-->L
1GLEG	152 - 156	154	R-->S
1GLEG	152 - 156	154	R-->Y
1GLEG	152 - 156	155	A-->L
1GLEG	152 - 156	155	A-->N
1GLEG	152 - 156	155	A-->T
1GLEG	152 - 156	155	A-->V
1GLEG	152 - 156	156	R-->A
1GLEG	152 - 156	156	R-->G
1GLEG	152 - 156	156	R-->L
1GLEG	152 - 156	156	R-->Q

1GLEG	183 - 187	183	Y-->G
1GLEG	183 - 187	183	Y-->I
1GLEG	183 - 187	183	Y-->K
1GLEG	183 - 187	183	Y-->L
1GLEG	183 - 187	183	Y-->P
1GLEG	183 - 187	184	T-->A
1GLEG	183 - 187	184	T-->G
1GLEG	183 - 187	184	T-->I
1GLEG	183 - 187	184	T-->K
1GLEG	183 - 187	184	T-->L
1GLEG	183 - 187	184	T-->V
1GLEG	183 - 187	184	T-->W
1GLEG	183 - 187	185	N-->A
1GLEG	183 - 187	185	N-->D
1GLEG	183 - 187	185	N-->F
1GLEG	183 - 187	185	N-->G
1GLEG	183 - 187	185	N-->L
1GLEG	183 - 187	185	N-->P
1GLEG	183 - 187	185	N-->S
1GLEG	183 - 187	186	A-->C
1GLEG	183 - 187	186	A-->K
1GLEG	183 - 187	186	A-->L
1GLEG	183 - 187	186	A-->N
1GLEG	183 - 187	186	A-->R
1GLEG	183 - 187	187	S-->N
1GLEG	183 - 187	187	S-->V
1GLEG	183 - 187	187	S-->Y

1GLEG	201 - 206	201	D-->L
1GLEG	201 - 206	201	D-->Y
1GLEG	201 - 206	203	M-->F
1GLEG	201 - 206	203	M-->K
1GLEG	329 - 333	329	S-->G
1GLEG	329 - 333	329	S-->P
1GLEG	329 - 333	330	E-->F
1GLEG	329 - 333	330	E-->M
1GLEG	329 - 333	330	E-->Q
1GLEG	329 - 333	330	E-->V
1GLEG	329 - 333	331	Y-->A
1GLEG	329 - 333	331	Y-->D
1GLEG	329 - 333	331	Y-->E
1GLEG	329 - 333	331	Y-->I
1GLEG	329 - 333	331	Y-->K
1GLEG	329 - 333	331	Y-->N
1GLEG	329 - 333	331	Y-->R
1GLEG	329 - 333	331	Y-->T
1GLEG	329 - 333	331	Y-->V
1GLEG	329 - 333	332	F-->A
1GLEG	329 - 333	332	F-->G
1GLEG	329 - 333	332	F-->I
1GLEG	329 - 333	332	F-->L
1GLEG	329 - 333	332	F-->P
1GLEG	329 - 333	333	A-->D
1GLEG	329 - 333	333	A-->H
1GLEG	329 - 333	333	A-->S

1GLEG	329	333	333	A-->V
1ZR5B	202	207	204	N-->A
1ZR5B	202	207	204	N-->P
1ZR5B	202	207	205	L-->S
1ZR5B	202	207	205	L-->T
1ZR5B	202	207	206	A-->E
1ZR5B	202	207	206	A-->L
2PK9C	89	93	89	L-->E
2PK9C	89	93	89	L-->K
2PK9C	89	93	89	L-->N
2PK9C	89	93	90	K-->I
2PK9C	89	93	91	K-->A
2PK9C	89	93	91	K-->F
2PK9C	89	93	91	K-->G
2PK9C	89	93	91	K-->N
2PK9C	89	93	92	Y-->K
2PK9C	89	93	92	Y-->V
2PK9C	89	93	93	M-->D
2PK9C	89	93	93	M-->E
2PK9C	89	93	93	M-->F
2PK9C	89	93	93	M-->N
2PK9C	89	93	93	M-->R
2PK9C	89	93	93	M-->S
2PK9C	89	93	93	M-->V
2PK9C	89	93	93	M-->Y
2PK9C	234	238	234	P-->A
2PK9C	234	238	234	P-->E

2PK9C	234 - 238	234	P-->I
2PK9C	234 - 238	234	P-->L
2PK9C	234 - 238	234	P-->M
2PK9C	234 - 238	234	P-->R
2PK9C	234 - 238	234	P-->V
2PK9C	234 - 238	235	S-->I
2PK9C	234 - 238	235	S-->K
2PK9C	234 - 238	235	S-->L
2PK9C	234 - 238	235	S-->N
2PK9C	234 - 238	236	V-->F
2PK9C	234 - 238	236	V-->G
2PK9C	234 - 238	236	V-->K
2PK9C	234 - 238	236	V-->M
2PK9C	234 - 238	236	V-->N
2PK9C	234 - 238	236	V-->Q
2PK9C	234 - 238	236	V-->S
2PK9C	234 - 238	236	V-->T
2PK9C	234 - 238	236	V-->W
2PK9C	234 - 238	236	V-->Y
2PK9C	234 - 238	237	T-->A
2PK9C	234 - 238	237	T-->F
2PK9C	234 - 238	237	T-->G
2PK9C	234 - 238	237	T-->H
2PK9C	234 - 238	237	T-->I
2PK9C	234 - 238	237	T-->K
2PK9C	234 - 238	237	T-->L
2PK9C	234 - 238	237	T-->P

2PK9C	234 - 238	237	T-->Y
2PK9C	234 - 238	238	K-->A
2PK9C	234 - 238	238	K-->C
2PK9C	234 - 238	238	K-->D
2PK9C	234 - 238	238	K-->E
2PK9C	234 - 238	238	K-->H
2PK9C	234 - 238	238	K-->I
2PK9C	234 - 238	238	K-->L
2PK9C	234 - 238	238	K-->P
2PK9C	234 - 238	238	K-->S
2PK9C	234 - 238	238	K-->T
2PK9C	234 - 238	238	K-->V
2O42A	53 - 57	53	E-->G
2O42A	53 - 57	53	E-->H
2O42A	53 - 57	53	E-->I
2O42A	53 - 57	53	E-->K
2O42A	53 - 57	53	E-->N
2O42A	53 - 57	53	E-->R
2O42A	53 - 57	53	E-->W
2O42A	53 - 57	53	E-->Y
2O42A	53 - 57	54	R-->A
2O42A	53 - 57	54	R-->D
2O42A	53 - 57	54	R-->F
2O42A	53 - 57	54	R-->G
2O42A	53 - 57	54	R-->H
2O42A	53 - 57	54	R-->I
2O42A	53 - 57	54	R-->K

2042A	53 - 57	54	R-->L
2042A	53 - 57	54	R-->M
2042A	53 - 57	54	R-->S
2042A	53 - 57	54	R-->T
2042A	53 - 57	54	R-->V
2042A	53 - 57	55	D-->A
2042A	53 - 57	55	D-->F
2042A	53 - 57	55	D-->G
2042A	53 - 57	55	D-->H
2042A	53 - 57	55	D-->I
2042A	53 - 57	55	D-->K
2042A	53 - 57	55	D-->N
2042A	53 - 57	55	D-->P
2042A	53 - 57	55	D-->R
2042A	53 - 57	55	D-->V
2042A	53 - 57	56	I-->A
2042A	53 - 57	56	I-->D
2042A	53 - 57	56	I-->F
2042A	53 - 57	56	I-->G
2042A	53 - 57	56	I-->L
2042A	53 - 57	56	I-->N
2042A	53 - 57	56	I-->P
2042A	53 - 57	56	I-->R
2042A	53 - 57	56	I-->T
2042A	53 - 57	56	I-->V
2042A	53 - 57	57	L-->E
2042A	53 - 57	57	L-->I

2O42A	53 - 57	57	L-->M
2O42A	53 - 57	57	L-->P
2O42A	53 - 57	57	L-->R
2GLUA	-2 - 3	-2	H-->I
2GLUA	-2 - 3	1	L-->S
2GLUA	-2 - 3	3	L-->E
2GLUA	145 - 150	147	E-->P
2GLUA	197 - 202	197	D-->I
2GLUA	197 - 202	200	R-->V
2IO2C	434 - 439	435	S-->T
2IO2C	443 - 447	443	P-->A
2IO2C	443 - 447	443	P-->D
2IO2C	443 - 447	443	P-->E
2IO2C	443 - 447	443	P-->G
2IO2C	443 - 447	443	P-->H
2IO2C	443 - 447	443	P-->I
2IO2C	443 - 447	443	P-->K
2IO2C	443 - 447	443	P-->L
2IO2C	443 - 447	443	P-->Q
2IO2C	443 - 447	443	P-->R
2IO2C	443 - 447	443	P-->S
2IO2C	443 - 447	443	P-->T
2IO2C	443 - 447	443	P-->V
2IO2C	443 - 447	444	E-->A
2IO2C	443 - 447	444	E-->D
2IO2C	443 - 447	444	E-->G
2IO2C	443 - 447	444	E-->H

2IO2C	443 - 447	444	E-->K
2IO2C	443 - 447	444	E-->L
2IO2C	443 - 447	444	E-->N
2IO2C	443 - 447	444	E-->P
2IO2C	443 - 447	444	E-->R
2IO2C	443 - 447	444	E-->S
2IO2C	443 - 447	444	E-->V
2IO2C	443 - 447	445	K-->D
2IO2C	443 - 447	445	K-->E
2IO2C	443 - 447	445	K-->G
2IO2C	443 - 447	445	K-->H
2IO2C	443 - 447	445	K-->I
2IO2C	443 - 447	445	K-->P
2IO2C	443 - 447	445	K-->R
2IO2C	443 - 447	445	K-->T
2IO2C	443 - 447	445	K-->V
2IO2C	443 - 447	446	L-->C
2IO2C	443 - 447	446	L-->E
2IO2C	443 - 447	446	L-->F
2IO2C	443 - 447	446	L-->G
2IO2C	443 - 447	446	L-->I
2IO2C	443 - 447	446	L-->N
2IO2C	443 - 447	446	L-->P
2IO2C	443 - 447	446	L-->Q
2IO2C	443 - 447	446	L-->R
2IO2C	443 - 447	446	L-->S
2IO2C	443 - 447	446	L-->T

2IO2C	443	-	447	447	L-->D
2IO2C	443	-	447	447	L-->E
2IO2C	443	-	447	447	L-->G
2IO2C	443	-	447	447	L-->N
2IO2C	443	-	447	447	L-->P
2IO2C	443	-	447	447	L-->Q
2IO2C	443	-	447	447	L-->R
2IO2C	443	-	447	447	L-->S
2IO2C	443	-	447	447	L-->Y
1QQEA	21	-	25	21	F-->Q
1QQEA	21	-	25	22	M-->G
1QQEA	21	-	25	22	M-->R
1QQEA	21	-	25	22	M-->S
1QQEA	21	-	25	22	M-->V
1FYVA	655	-	660	655	L-->I
1FYVA	655	-	660	656	L-->E
1FYVA	655	-	660	659	L-->E
1FYVA	699	-	704	703	Q-->D
1FYVA	699	-	704	703	Q-->M
1FYVA	744	-	749	744	H-->D
1FYVA	744	-	749	744	H-->G
1FYVA	744	-	749	744	H-->K
1FYVA	744	-	749	745	K-->S
1FYVA	744	-	749	749	L-->G
2B9RA	14	-	19	16	Q-->K
2B9RA	14	-	19	16	Q-->L
2B9RA	235	-	239	235	T-->A

2B9RA	235 - 239	235	T-->D
2B9RA	235 - 239	235	T-->E
2B9RA	235 - 239	235	T-->I
2B9RA	235 - 239	235	T-->K
2B9RA	235 - 239	235	T-->L
2B9RA	235 - 239	235	T-->N
2B9RA	235 - 239	235	T-->R
2B9RA	235 - 239	235	T-->S
2B9RA	235 - 239	235	T-->V
2B9RA	235 - 239	235	T-->Y
2B9RA	235 - 239	236	V-->D
2B9RA	235 - 239	236	V-->G
2B9RA	235 - 239	236	V-->L
2B9RA	235 - 239	236	V-->S
2B9RA	235 - 239	236	V-->T
2B9RA	235 - 239	237	K-->D
2B9RA	235 - 239	237	K-->G
2B9RA	235 - 239	237	K-->H
2B9RA	235 - 239	237	K-->I
2B9RA	235 - 239	237	K-->N
2B9RA	235 - 239	237	K-->P
2B9RA	235 - 239	237	K-->R
2B9RA	235 - 239	237	K-->S
2B9RA	235 - 239	237	K-->T
2B9RA	235 - 239	237	K-->W
2B9RA	235 - 239	238	N-->A
2B9RA	235 - 239	238	N-->D

2B9RA	235	239	238	N-->E
2B9RA	235	239	238	N-->F
2B9RA	235	239	238	N-->G
2B9RA	235	239	238	N-->I
2B9RA	235	239	238	N-->L
2B9RA	235	239	238	N-->M
2B9RA	235	239	238	N-->P
2B9RA	235	239	238	N-->T
2B9RA	235	239	238	N-->V
2B9RA	235	239	238	N-->Y
2B9RA	235	239	239	K-->A
2B9RA	235	239	239	K-->C
2B9RA	235	239	239	K-->D
2B9RA	235	239	239	K-->F
2B9RA	235	239	239	K-->G
2B9RA	235	239	239	K-->I
2B9RA	235	239	239	K-->L
2B9RA	235	239	239	K-->N
2B9RA	235	239	239	K-->Q
2B9RA	235	239	239	K-->S
2B9RA	235	239	239	K-->T
2B9RA	235	239	239	K-->V
2B9RA	235	239	239	K-->W
1OFTA	89	94	91	W-->D
2OZKD	69	74	69	I-->D
2OZKD	69	74	70	K-->L
2OZKD	69	74	71	I-->V

2OZKD	69 - 74	72	L-->G
2OZKD	69 - 74	73	N-->G
2OZKD	69 - 74	74	N-->I
2OZKD	220 - 224	220	E-->S
2OZKD	220 - 224	221	F-->D
2OZKD	220 - 224	221	F-->I
2OZKD	220 - 224	221	F-->N
2OZKD	220 - 224	221	F-->S
2OZKD	220 - 224	221	F-->V
2OZKD	220 - 224	223	Q-->C
2OZKD	220 - 224	223	Q-->I
2OZKD	220 - 224	223	Q-->N
2OZKD	220 - 224	223	Q-->Y
2OZKD	220 - 224	224	R-->D
2OZKD	220 - 224	224	R-->P
2OZKD	233 - 237	233	E-->A
2OZKD	233 - 237	233	E-->Q
2OZKD	233 - 237	233	E-->V
2OZKD	233 - 237	234	H-->A
2OZKD	233 - 237	234	H-->F
2OZKD	233 - 237	234	H-->G
2OZKD	233 - 237	234	H-->N
2OZKD	233 - 237	234	H-->R
2OZKD	233 - 237	234	H-->Y
2OZKD	233 - 237	235	I-->E
2OZKD	233 - 237	235	I-->P
2OZKD	233 - 237	235	I-->S

2OZKD	233 - 237	235	I-->V
2OZKD	233 - 237	235	I-->Y
2OZKD	233 - 237	237	Y-->A
2OZKD	233 - 237	237	Y-->G
2OZKD	233 - 237	237	Y-->L
2OZKD	233 - 237	237	Y-->M
2OZKD	233 - 237	237	Y-->Q
2OZKD	233 - 237	237	Y-->S
2OZKD	233 - 237	237	Y-->V
1MKDA	203 - 209	204	F-->P
1MKDA	306 - 311	306	N-->S
1MKDA	306 - 311	307	Q-->S
1MKDA	306 - 311	308	F-->Q
1MKDA	315 - 319	315	E-->A
1MKDA	315 - 319	315	E-->D
1MKDA	315 - 319	315	E-->G
1MKDA	315 - 319	315	E-->H
1MKDA	315 - 319	315	E-->L
1MKDA	315 - 319	315	E-->V
1MKDA	315 - 319	316	L-->E
1MKDA	315 - 319	316	L-->K
1MKDA	315 - 319	316	L-->T
1MKDA	315 - 319	317	A-->E
1MKDA	315 - 319	317	A-->F
1MKDA	315 - 319	317	A-->G
1MKDA	315 - 319	317	A-->P
1MKDA	315 - 319	318	L-->E

1MKDA	315 - 319	318	L-->H
1MKDA	315 - 319	318	L-->V
1MKDA	315 - 319	319	M-->A
1MKDA	315 - 319	319	M-->C
1MKDA	315 - 319	319	M-->D
1MKDA	315 - 319	319	M-->G
1MKDA	315 - 319	319	M-->I
1MKDA	315 - 319	319	M-->K
1MKDA	315 - 319	319	M-->P
1MKDA	315 - 319	319	M-->Q
1MKDA	315 - 319	319	M-->S
1MKDA	315 - 319	319	M-->T
1MKDA	315 - 319	319	M-->V
1MKDA	315 - 319	319	M-->W
1W1WE	504 - 509	504	F-->D
1W1WE	504 - 509	504	F-->E
1W1WE	504 - 509	504	F-->Y
1W1WE	504 - 509	505	T-->D
1W1WE	504 - 509	507	V-->K
1W1WE	504 - 509	507	V-->Q
2PJRB	636 - 640	636	R-->E
2PJRB	636 - 640	636	R-->K
2PJRB	636 - 640	637	F-->A
2PJRB	636 - 640	637	F-->E
2PJRB	636 - 640	637	F-->H
2PJRB	636 - 640	637	F-->K
2PJRB	636 - 640	637	F-->R

2PJRB	636 - 640	637	F-->V
2PJRB	636 - 640	637	F-->Y
2PJRB	636 - 640	638	L-->D
2PJRB	636 - 640	638	L-->M
2PJRB	636 - 640	638	L-->V
2PJRB	636 - 640	638	L-->W
2PJRB	636 - 640	639	N-->A
2PJRB	636 - 640	639	N-->F
2PJRB	636 - 640	639	N-->I
2PJRB	636 - 640	639	N-->L
2PJRB	636 - 640	639	N-->S
2PJRB	636 - 640	639	N-->T
2PJRB	636 - 640	640	E-->N
2PJRB	636 - 640	640	E-->P
2PJRB	636 - 640	640	E-->V
1R0BG	26 - 32	27	F-->D
1XQSA	145 - 150	147	H-->P
1HWOA	162 - 166	162	P-->A
1HWOA	162 - 166	162	P-->D
1HWOA	162 - 166	162	P-->F
1HWOA	162 - 166	162	P-->G
1HWOA	162 - 166	162	P-->M
1HWOA	162 - 166	162	P-->R
1HWOA	162 - 166	162	P-->V
1HWOA	162 - 166	163	E-->G
1HWOA	162 - 166	163	E-->H
1HWOA	162 - 166	163	E-->K

1HWOA	162 - 166	163	E-->L
1HWOA	162 - 166	163	E-->Q
1HWOA	162 - 166	163	E-->R
1HWOA	162 - 166	163	E-->V
1HWOA	162 - 166	164	A-->G
1HWOA	162 - 166	164	A-->K
1HWOA	162 - 166	164	A-->L
1HWOA	162 - 166	164	A-->R
1HWOA	162 - 166	164	A-->T
1HWOA	162 - 166	165	A-->D
1HWOA	162 - 166	165	A-->F
1HWOA	162 - 166	165	A-->L
1HWOA	162 - 166	165	A-->P
1HWOA	162 - 166	165	A-->R
1HWOA	162 - 166	165	A-->S
1HWOA	162 - 166	165	A-->V
1HWOA	162 - 166	165	A-->Y
1HWOA	162 - 166	166	R-->A
1HWOA	162 - 166	166	R-->D
1HWOA	162 - 166	166	R-->L
1HWOA	162 - 166	166	R-->S
1HWOA	162 - 166	166	R-->T
1HWOA	162 - 166	166	R-->Y
1XVLA	118 - 123	120	Q-->E
1XVLA	118 - 123	120	Q-->G
1XVLA	224 - 229	224	S-->V
1XVLA	224 - 229	227	A-->L

1SRQD	177 - 183	179	R-->Y
1SRQD	177 - 183	182	V-->L
2IUUA	378 - 383	378	V-->Y
2IUUA	378 - 383	380	R-->T
2IUUA	378 - 383	381	I-->A
2IUUA	378 - 383	383	N-->G
2IUUA	423 - 427	423	F-->D
2IUUA	423 - 427	423	F-->G
2IUUA	423 - 427	423	F-->K
2IUUA	423 - 427	423	F-->L
2IUUA	423 - 427	423	F-->N
2IUUA	423 - 427	423	F-->P
2IUUA	423 - 427	423	F-->Q
2IUUA	423 - 427	423	F-->R
2IUUA	423 - 427	423	F-->S
2IUUA	423 - 427	423	F-->V
2IUUA	423 - 427	424	S-->G
2IUUA	423 - 427	424	S-->L
2IUUA	423 - 427	424	S-->Y
2IUUA	423 - 427	425	E-->F
2IUUA	423 - 427	425	E-->I
2IUUA	423 - 427	425	E-->K
2IUUA	423 - 427	425	E-->L
2IUUA	423 - 427	425	E-->N
2IUUA	423 - 427	425	E-->P
2IUUA	423 - 427	425	E-->R
2IUUA	423 - 427	425	E-->T

2IUUA	423	- 427	425	E-->Y
2IUUA	423	- 427	426	V-->G
2IUUA	423	- 427	426	V-->I
2IUUA	423	- 427	426	V-->K
2IUUA	423	- 427	426	V-->M
2IUUA	423	- 427	426	V-->N
2IUUA	423	- 427	426	V-->P
2IUUA	423	- 427	426	V-->T
2IUUA	423	- 427	427	L-->E
2IUUA	423	- 427	427	L-->I
2IUUA	423	- 427	427	L-->K
2IUUA	423	- 427	427	L-->Q
2IUUA	423	- 427	427	L-->R
2IUUA	423	- 427	427	L-->S
2IUUA	423	- 427	427	L-->T
2IUUA	503	- 507	503	E-->H
2IUUA	503	- 507	503	E-->L
2IUUA	503	- 507	503	E-->P
2IUUA	503	- 507	503	E-->R
2IUUA	503	- 507	503	E-->Y
2IUUA	503	- 507	504	L-->D
2IUUA	503	- 507	504	L-->F
2IUUA	503	- 507	504	L-->G
2IUUA	503	- 507	504	L-->H
2IUUA	503	- 507	504	L-->R
2IUUA	503	- 507	504	L-->S
2IUUA	503	- 507	504	L-->V

2IUUA	503 - 507	505	S-->D	
2IUUA	503 - 507	505	S-->G	
2IUUA	503 - 507	505	S-->K	
2IUUA	503 - 507	505	S-->N	
2IUUA	503 - 507	505	S-->P	
2IUUA	503 - 507	505	S-->Q	
2IUUA	503 - 507	505	S-->R	
2IUUA	503 - 507	506	I-->D	
2IUUA	503 - 507	506	I-->G	
2IUUA	503 - 507	506	I-->L	
2IUUA	503 - 507	506	I-->N	
2IUUA	503 - 507	506	I-->P	
2IUUA	503 - 507	506	I-->Q	
2IUUA	503 - 507	506	I-->S	
2IUUA	503 - 507	506	I-->V	
2IUUA	503 - 507	507	Y-->D	
2IUUA	503 - 507	507	Y-->E	
2IUUA	503 - 507	507	Y-->F	
2IUUA	503 - 507	507	Y-->H	
2IUUA	503 - 507	507	Y-->I	
2IUUA	503 - 507	507	Y-->K	
2IUUA	503 - 507	507	Y-->Q	
2IUUA	503 - 507	507	Y-->S	
2IUUA	503 - 507	507	Y-->T	
2IUUA	503 - 507	507	Y-->V	
2IUUA	640 - 644	640	G-->D	
2IUUA	640 - 644	640	G-->H	

2IUUA	640 - 644	640	G-->I
2IUUA	640 - 644	640	G-->K
2IUUA	640 - 644	640	G-->L
2IUUA	640 - 644	640	G-->Q
2IUUA	640 - 644	640	G-->R
2IUUA	640 - 644	640	G-->T
2IUUA	640 - 644	640	G-->V
2IUUA	640 - 644	641	L-->A
2IUUA	640 - 644	641	L-->E
2IUUA	640 - 644	641	L-->F
2IUUA	640 - 644	641	L-->G
2IUUA	640 - 644	641	L-->I
2IUUA	640 - 644	641	L-->N
2IUUA	640 - 644	641	L-->P
2IUUA	640 - 644	641	L-->Q
2IUUA	640 - 644	641	L-->R
2IUUA	640 - 644	641	L-->S
2IUUA	640 - 644	642	I-->A
2IUUA	640 - 644	642	I-->D
2IUUA	640 - 644	642	I-->E
2IUUA	640 - 644	642	I-->F
2IUUA	640 - 644	642	I-->G
2IUUA	640 - 644	642	I-->L
2IUUA	640 - 644	642	I-->P
2IUUA	640 - 644	642	I-->Q
2IUUA	640 - 644	642	I-->R
2IUUA	640 - 644	642	I-->S

2IUUA	640 - 644	642	I-->V	
2IUUA	640 - 644	643	K-->A	
2IUUA	640 - 644	643	K-->C	
2IUUA	640 - 644	643	K-->D	
2IUUA	640 - 644	643	K-->F	
2IUUA	640 - 644	643	K-->G	
2IUUA	640 - 644	643	K-->L	
2IUUA	640 - 644	643	K-->P	
2IUUA	640 - 644	643	K-->Q	
2IUUA	640 - 644	643	K-->S	
2IUUA	640 - 644	643	K-->V	
2IUUA	640 - 644	643	K-->W	
2IUUA	640 - 644	643	K-->Y	
2IUUA	640 - 644	644	A-->C	
2IUUA	640 - 644	644	A-->D	
2IUUA	640 - 644	644	A-->E	
2IUUA	640 - 644	644	A-->F	
2IUUA	640 - 644	644	A-->G	
2IUUA	640 - 644	644	A-->H	
2IUUA	640 - 644	644	A-->L	
2IUUA	640 - 644	644	A-->N	
2IUUA	640 - 644	644	A-->Q	
2IUUA	640 - 644	644	A-->R	
2IUUA	640 - 644	644	A-->S	
2IUUA	640 - 644	644	A-->T	
2IUUA	640 - 644	644	A-->V	
1L0AA	476 - 480	476	Q-->A	

1L0AA	476 - 480	476	Q-->C
1L0AA	476 - 480	476	Q-->D
1L0AA	476 - 480	476	Q-->E
1L0AA	476 - 480	476	Q-->G
1L0AA	476 - 480	476	Q-->L
1L0AA	476 - 480	476	Q-->N
1L0AA	476 - 480	476	Q-->V
1L0AA	476 - 480	476	Q-->Y
1L0AA	476 - 480	477	T-->A
1L0AA	476 - 480	477	T-->F
1L0AA	476 - 480	477	T-->I
1L0AA	476 - 480	477	T-->K
1L0AA	476 - 480	477	T-->R
1L0AA	476 - 480	477	T-->W
1L0AA	476 - 480	478	V-->L
1L0AA	476 - 480	478	V-->T
1L0AA	476 - 480	479	L-->C
1L0AA	476 - 480	479	L-->F
1L0AA	476 - 480	479	L-->I
1L0AA	476 - 480	479	L-->P
1L0AA	476 - 480	479	L-->Q
1L0AA	476 - 480	479	L-->V
1L0AA	476 - 480	479	L-->Y
1L0AA	476 - 480	480	E-->A
1L0AA	476 - 480	480	E-->C
1L0AA	476 - 480	480	E-->F
1L0AA	476 - 480	480	E-->G

1L0AA	476	480	480	E-->L
1L0AA	476	480	480	E-->P
1L0AA	476	480	480	E-->S
1L0AA	476	480	480	E-->T
1L0AA	476	480	480	E-->V
2HJMC	33	37	33	I-->D
2HJMC	33	37	33	I-->K
2HJMC	33	37	33	I-->N
2HJMC	33	37	33	I-->S
2HJMC	33	37	34	E-->G
2HJMC	33	37	34	E-->L
2HJMC	33	37	34	E-->V
2HJMC	33	37	35	K-->F
2HJMC	33	37	35	K-->G
2HJMC	33	37	35	K-->T
2HJMC	33	37	35	K-->Y
2HJMC	33	37	36	T-->E
2HJMC	33	37	36	T-->G
2HJMC	33	37	36	T-->K
2HJMC	33	37	36	T-->L
2HJMC	33	37	36	T-->N
2HJMC	33	37	36	T-->P
2HJMC	33	37	36	T-->Y
2HJMC	33	37	37	R-->I
2HJMC	33	37	37	R-->K
2HJMC	33	37	37	R-->N
2HJMC	33	37	37	R-->P

2HJMC	33 - 37	37	R-->T
2HJMC	33 - 37	37	R-->V
2BP7B	46 - 50	46	L-->G
2BP7B	46 - 50	46	L-->R
2BP7B	46 - 50	46	L-->T
2BP7B	46 - 50	47	Q-->G
2BP7B	46 - 50	47	Q-->I
2BP7B	46 - 50	47	Q-->L
2BP7B	46 - 50	47	Q-->P
2BP7B	46 - 50	47	Q-->R
2BP7B	46 - 50	47	Q-->S
2BP7B	46 - 50	47	Q-->T
2BP7B	46 - 50	47	Q-->Y
2BP7B	46 - 50	48	T-->F
2BP7B	46 - 50	48	T-->L
2BP7B	46 - 50	48	T-->V
2BP7B	46 - 50	49	K-->D
2BP7B	46 - 50	49	K-->S
2BP7B	46 - 50	50	Y-->I
2BP7B	46 - 50	50	Y-->K
2BP7B	46 - 50	50	Y-->L
2BP7B	46 - 50	50	Y-->N
2BP7B	46 - 50	50	Y-->P
2BP7B	46 - 50	50	Y-->R
2BP7B	46 - 50	50	Y-->T
2BP7B	46 - 50	50	Y-->V
1C9IA	334 - 339	334	I-->F

1C9IA	334	- 339	335	I-->E
1C9IA	334	- 339	339	T-->E
1N8BA	150	- 155	153	C-->N
1N8BA	196	- 201	196	P-->Y
1N8BA	196	- 201	199	S-->I
1N8BA	213	- 218	215	E-->Y
1N8BA	213	- 218	216	L-->A
1N8BA	213	- 218	216	L-->P
1N8BA	213	- 218	217	K-->R
1N8BA	213	- 218	218	E-->V
1TL1B	78	- 82	78	R-->F
1TL1B	78	- 82	78	R-->G
1TL1B	78	- 82	78	R-->H
1TL1B	78	- 82	78	R-->K
1TL1B	78	- 82	78	R-->P
1TL1B	78	- 82	78	R-->Q
1TL1B	78	- 82	78	R-->T
1TL1B	78	- 82	78	R-->V
1TL1B	78	- 82	79	E-->D
1TL1B	78	- 82	79	E-->I
1TL1B	78	- 82	79	E-->L
1TL1B	78	- 82	79	E-->P
1TL1B	78	- 82	79	E-->T
1TL1B	78	- 82	80	L-->E
1TL1B	78	- 82	80	L-->H
1TL1B	78	- 82	80	L-->N
1TL1B	78	- 82	80	L-->T

1TL1B	78 - 82	81	N-->A
1TL1B	78 - 82	81	N-->D
1TL1B	78 - 82	81	N-->I
1TL1B	78 - 82	81	N-->K
1TL1B	78 - 82	81	N-->T
1TL1B	78 - 82	82	K-->D
1TL1B	78 - 82	82	K-->E
1TL1B	78 - 82	82	K-->F
1TL1B	78 - 82	82	K-->G
1TL1B	78 - 82	82	K-->P
1TL1B	78 - 82	82	K-->V
1TL1B	78 - 82	82	K-->Y
1TL1B	155 - 159	155	G-->A
1TL1B	155 - 159	155	G-->D
1TL1B	155 - 159	155	G-->F
1TL1B	155 - 159	155	G-->K
1TL1B	155 - 159	155	G-->L
1TL1B	155 - 159	155	G-->Q
1TL1B	155 - 159	155	G-->T
1TL1B	155 - 159	155	G-->V
1TL1B	155 - 159	156	S-->A
1TL1B	155 - 159	156	S-->D
1TL1B	155 - 159	156	S-->G
1TL1B	155 - 159	156	S-->I
1TL1B	155 - 159	156	S-->K
1TL1B	155 - 159	156	S-->L
1TL1B	155 - 159	156	S-->N

1TL1B	155	159	156	S-->P
1TL1B	155	159	156	S-->Q
1TL1B	155	159	156	S-->R
1TL1B	155	159	156	S-->T
1TL1B	155	159	156	S-->V
1TL1B	155	159	157	P-->A
1TL1B	155	159	157	P-->C
1TL1B	155	159	157	P-->D
1TL1B	155	159	157	P-->F
1TL1B	155	159	157	P-->G
1TL1B	155	159	157	P-->I
1TL1B	155	159	157	P-->K
1TL1B	155	159	157	P-->L
1TL1B	155	159	157	P-->Q
1TL1B	155	159	157	P-->R
1TL1B	155	159	157	P-->S
1TL1B	155	159	157	P-->T
1TL1B	155	159	157	P-->V
1TL1B	155	159	158	A-->C
1TL1B	155	159	158	A-->D
1TL1B	155	159	158	A-->G
1TL1B	155	159	158	A-->I
1TL1B	155	159	158	A-->S
1TL1B	155	159	158	A-->T
1TL1B	155	159	158	A-->V
1TL1B	155	159	159	I-->A
1TL1B	155	159	159	I-->D

1TL1B	155 - 159	159	I-->G
1TL1B	155 - 159	159	I-->H
1TL1B	155 - 159	159	I-->K
1TL1B	155 - 159	159	I-->L
1TL1B	155 - 159	159	I-->N
1TL1B	155 - 159	159	I-->P
1TL1B	155 - 159	159	I-->R
1TL1B	155 - 159	159	I-->T
1TL1B	155 - 159	159	I-->V
1TL1B	421 - 425	421	P-->A
1TL1B	421 - 425	421	P-->C
1TL1B	421 - 425	421	P-->D
1TL1B	421 - 425	421	P-->F
1TL1B	421 - 425	421	P-->G
1TL1B	421 - 425	421	P-->H
1TL1B	421 - 425	421	P-->K
1TL1B	421 - 425	421	P-->L
1TL1B	421 - 425	421	P-->M
1TL1B	421 - 425	421	P-->N
1TL1B	421 - 425	421	P-->Q
1TL1B	421 - 425	421	P-->R
1TL1B	421 - 425	421	P-->S
1TL1B	421 - 425	421	P-->T
1TL1B	421 - 425	421	P-->V
1TL1B	421 - 425	421	P-->Y
1TL1B	421 - 425	422	L-->A
1TL1B	421 - 425	422	L-->C

1TL1B	421 - 425	422	L-->D
1TL1B	421 - 425	422	L-->E
1TL1B	421 - 425	422	L-->F
1TL1B	421 - 425	422	L-->G
1TL1B	421 - 425	422	L-->H
1TL1B	421 - 425	422	L-->K
1TL1B	421 - 425	422	L-->N
1TL1B	421 - 425	422	L-->R
1TL1B	421 - 425	422	L-->S
1TL1B	421 - 425	422	L-->T
1TL1B	421 - 425	422	L-->V
1TL1B	421 - 425	422	L-->Y
1TL1B	421 - 425	423	V-->D
1TL1B	421 - 425	423	V-->E
1TL1B	421 - 425	423	V-->F
1TL1B	421 - 425	423	V-->G
1TL1B	421 - 425	423	V-->K
1TL1B	421 - 425	423	V-->L
1TL1B	421 - 425	423	V-->M
1TL1B	421 - 425	423	V-->N
1TL1B	421 - 425	423	V-->P
1TL1B	421 - 425	423	V-->R
1TL1B	421 - 425	423	V-->T
1TL1B	421 - 425	424	K-->A
1TL1B	421 - 425	424	K-->C
1TL1B	421 - 425	424	K-->D
1TL1B	421 - 425	424	K-->F

1TL1B	421 - 425	424	K-->H
1TL1B	421 - 425	424	K-->I
1TL1B	421 - 425	424	K-->L
1TL1B	421 - 425	424	K-->M
1TL1B	421 - 425	424	K-->N
1TL1B	421 - 425	424	K-->P
1TL1B	421 - 425	424	K-->Q
1TL1B	421 - 425	424	K-->R
1TL1B	421 - 425	424	K-->S
1TL1B	421 - 425	424	K-->V
1TL1B	421 - 425	424	K-->Y
1TL1B	421 - 425	425	L-->A
1TL1B	421 - 425	425	L-->E
1TL1B	421 - 425	425	L-->F
1TL1B	421 - 425	425	L-->G
1TL1B	421 - 425	425	L-->K
1TL1B	421 - 425	425	L-->N
1TL1B	421 - 425	425	L-->P
1TL1B	421 - 425	425	L-->S
1TL1B	421 - 425	425	L-->V
1TL1B	421 - 425	425	L-->W
1TL1B	421 - 425	425	L-->Y
1L0OA	89 - 93	89	I-->A
1L0OA	89 - 93	89	I-->D
1L0OA	89 - 93	89	I-->L
1L0OA	89 - 93	89	I-->R
1L0OA	89 - 93	89	I-->S

1L0OA	89 - 93	89	I-->T
1L0OA	89 - 93	90	E-->D
1L0OA	89 - 93	90	E-->I
1L0OA	89 - 93	90	E-->K
1L0OA	89 - 93	90	E-->L
1L0OA	89 - 93	90	E-->P
1L0OA	89 - 93	90	E-->V
1L0OA	89 - 93	91	E-->F
1L0OA	89 - 93	91	E-->G
1L0OA	89 - 93	91	E-->L
1L0OA	89 - 93	91	E-->N
1L0OA	89 - 93	91	E-->T
1L0OA	89 - 93	92	A-->C
1L0OA	89 - 93	92	A-->G
1L0OA	89 - 93	92	A-->H
1L0OA	89 - 93	92	A-->K
1L0OA	89 - 93	92	A-->M
1L0OA	89 - 93	92	A-->Q
1L0OA	89 - 93	92	A-->R
1L0OA	89 - 93	92	A-->S
1L0OA	89 - 93	93	R-->F
1L0OA	89 - 93	93	R-->H
1L0OA	89 - 93	93	R-->N
1L0OA	89 - 93	93	R-->P
1L0OA	89 - 93	93	R-->T
1L0OA	89 - 93	93	R-->V
1V14A	75 - 80	75	N-->E

1V14A	75 - 80	76	K-->V
1V14A	75 - 80	78	S-->P
1V14A	75 - 80	79	V-->F
2Q7OE	36 - 41	36	G-->D
2Q7OE	36 - 41	39	T-->A
2Q7OE	36 - 41	39	T-->G
2Q7OE	36 - 41	39	T-->L
2Q7OE	36 - 41	40	D-->P
2Q7OE	36 - 41	41	K-->P
2Q7OE	36 - 41	41	K-->W
2Q7OE	168 - 172	168	R-->L
2Q7OE	168 - 172	169	T-->D
2Q7OE	168 - 172	170	M-->H
2Q7OE	168 - 172	170	M-->I
2Q7OE	168 - 172	170	M-->T
2Q7OE	168 - 172	171	R-->L
2Q7OE	175 - 179	175	L-->F
2Q7OE	175 - 179	175	L-->T
2Q7OE	175 - 179	176	S-->A
2Q7OE	175 - 179	176	S-->G
2Q7OE	175 - 179	176	S-->R
2Q7OE	175 - 179	177	T-->G
2Q7OE	175 - 179	177	T-->V
2Q7OE	175 - 179	178	W-->A
2Q7OE	175 - 179	178	W-->F
2Q7OE	175 - 179	178	W-->G
2Q7OE	175 - 179	178	W-->I

2Q7OE	175	-	179	178	W-->L
2Q7OE	175	-	179	178	W-->P
2Q7OE	175	-	179	178	W-->S
2Q7OE	175	-	179	179	K-->E
2Q7OE	175	-	179	179	K-->I
2Q7OE	175	-	179	179	K-->N
2Q7OE	257	-	265	262	A-->E
1T72B	78	-	82	78	A-->E
1T72B	78	-	82	78	A-->K
1T72B	78	-	82	78	A-->L
1T72B	78	-	82	78	A-->P
1T72B	78	-	82	78	A-->Q
1T72B	78	-	82	78	A-->R
1T72B	78	-	82	78	A-->S
1T72B	78	-	82	78	A-->V
1T72B	78	-	82	79	G-->C
1T72B	78	-	82	79	G-->E
1T72B	78	-	82	79	G-->F
1T72B	78	-	82	79	G-->H
1T72B	78	-	82	79	G-->L
1T72B	78	-	82	79	G-->N
1T72B	78	-	82	79	G-->R
1T72B	78	-	82	79	G-->V
1T72B	78	-	82	79	G-->Y
1T72B	78	-	82	80	D-->A
1T72B	78	-	82	80	D-->E
1T72B	78	-	82	80	D-->F

1T72B	78 - 82	80	D-->G
1T72B	78 - 82	80	D-->H
1T72B	78 - 82	80	D-->I
1T72B	78 - 82	80	D-->K
1T72B	78 - 82	80	D-->M
1T72B	78 - 82	80	D-->N
1T72B	78 - 82	80	D-->S
1T72B	78 - 82	80	D-->V
1T72B	78 - 82	81	L-->A
1T72B	78 - 82	81	L-->F
1T72B	78 - 82	81	L-->G
1T72B	78 - 82	81	L-->H
1T72B	78 - 82	81	L-->I
1T72B	78 - 82	81	L-->K
1T72B	78 - 82	81	L-->P
1T72B	78 - 82	81	L-->Q
1T72B	78 - 82	81	L-->R
1T72B	78 - 82	81	L-->S
1T72B	78 - 82	81	L-->T
1T72B	78 - 82	81	L-->W
1T72B	78 - 82	82	R-->A
1T72B	78 - 82	82	R-->D
1T72B	78 - 82	82	R-->E
1T72B	78 - 82	82	R-->F
1T72B	78 - 82	82	R-->G
1T72B	78 - 82	82	R-->I
1T72B	78 - 82	82	R-->K

1T72B	78 - 82	82	R-->P
1T72B	78 - 82	82	R-->S
1T72B	78 - 82	82	R-->T
1T72B	78 - 82	82	R-->V
1T72B	78 - 82	82	R-->Y
1T72B	121 - 125	121	V-->A
1T72B	121 - 125	121	V-->H
1T72B	121 - 125	121	V-->K
1T72B	121 - 125	121	V-->L
1T72B	121 - 125	121	V-->Q
1T72B	121 - 125	122	N-->A
1T72B	121 - 125	122	N-->C
1T72B	121 - 125	122	N-->E
1T72B	121 - 125	122	N-->G
1T72B	121 - 125	122	N-->I
1T72B	121 - 125	122	N-->P
1T72B	121 - 125	122	N-->Q
1T72B	121 - 125	122	N-->V
1T72B	121 - 125	123	I-->G
1T72B	121 - 125	123	I-->S
1T72B	121 - 125	123	I-->V
1T72B	121 - 125	123	I-->Y
1T72B	121 - 125	124	N-->A
1T72B	121 - 125	124	N-->D
1T72B	121 - 125	124	N-->E
1T72B	121 - 125	124	N-->I
1T72B	121 - 125	124	N-->L

1T72B	121 - 125	124	N-->Q
1T72B	121 - 125	124	N-->T
1T72B	121 - 125	124	N-->V
1T72B	121 - 125	125	F-->G
1T72B	121 - 125	125	F-->I
1T72B	121 - 125	125	F-->N
1T72B	121 - 125	125	F-->V
1T72B	127 - 132	131	K-->H
1T72B	127 - 132	132	E-->F
1T72B	172 - 176	172	T-->A
1T72B	172 - 176	172	T-->C
1T72B	172 - 176	172	T-->D
1T72B	172 - 176	172	T-->E
1T72B	172 - 176	172	T-->G
1T72B	172 - 176	172	T-->L
1T72B	172 - 176	172	T-->V
1T72B	172 - 176	173	Y-->C
1T72B	172 - 176	173	Y-->D
1T72B	172 - 176	173	Y-->E
1T72B	172 - 176	173	Y-->H
1T72B	172 - 176	173	Y-->K
1T72B	172 - 176	173	Y-->L
1T72B	172 - 176	173	Y-->P
1T72B	172 - 176	173	Y-->Q
1T72B	172 - 176	173	Y-->R
1T72B	172 - 176	173	Y-->V
1T72B	172 - 176	173	Y-->W

1T72B	172 - 176	174	V-->A
1T72B	172 - 176	174	V-->G
1T72B	172 - 176	174	V-->K
1T72B	172 - 176	174	V-->L
1T72B	172 - 176	174	V-->N
1T72B	172 - 176	174	V-->T
1T72B	172 - 176	175	L-->A
1T72B	172 - 176	175	L-->E
1T72B	172 - 176	175	L-->I
1T72B	172 - 176	175	L-->P
1T72B	172 - 176	175	L-->T
1T72B	172 - 176	175	L-->V
1T72B	172 - 176	176	E-->A
1T72B	172 - 176	176	E-->F
1T72B	172 - 176	176	E-->G
1T72B	172 - 176	176	E-->K
1T72B	172 - 176	176	E-->L
1T72B	172 - 176	176	E-->S
1T72B	172 - 176	176	E-->T
1T72B	172 - 176	176	E-->V
1T72B	172 - 176	176	E-->Y
1TGHA	266 - 272	267	E-->I
1TGHA	266 - 272	270	V-->A
2H0JA	71 - 76	71	A-->V
2H0JA	71 - 76	75	F-->Y
2HPAA	1220 - 1225	1224	S-->V
2HPAA	1336 - 1340	1336	W-->A

2HPAA	1336	- 1340	1337	S-->T
2HPAA	1336	- 1340	1337	S-->V
2HPAA	1336	- 1340	1338	T-->A
2HPAA	1336	- 1340	1338	T-->V
2HPAA	1336	- 1340	1340	C-->D
2P1DA	22	- 28	27	I-->V
2P1DA	22	- 28	28	Y-->T
2P1DA	86	- 91	87	W-->L
1JWAD	40	- 45	41	R-->D
1JWAD	40	- 45	42	W-->A
1JWAD	40	- 45	42	W-->P
1JWAD	40	- 45	45	A-->Y
2ZKIG	65	- 70	68	M-->G
2ZKIG	65	- 70	68	M-->S
2ZKIG	97	- 101	98	I-->Y
2ZKIG	97	- 101	99	L-->A
2ZKIG	97	- 101	99	L-->K
2ZKIG	97	- 101	99	L-->S
2ZKIG	97	- 101	99	L-->V
2ZKIG	97	- 101	100	W-->A
2ZKIG	97	- 101	100	W-->E
2ZKIG	97	- 101	100	W-->K
2ZKIG	97	- 101	100	W-->P
2ZKIG	97	- 101	100	W-->S
2ZKIG	97	- 101	101	K-->G
2ZKIG	97	- 101	101	K-->Y
2ZKIG	125	- 130	125	T-->K

2ZKIG	132 - 137	136	H-->L
2UWSL	134 - 139	139	M-->V
2C8NB	72 - 76	72	N-->A
2C8NB	72 - 76	72	N-->C
2C8NB	72 - 76	72	N-->F
2C8NB	72 - 76	72	N-->L
2C8NB	72 - 76	72	N-->R
2C8NB	72 - 76	72	N-->S
2C8NB	72 - 76	72	N-->T
2C8NB	72 - 76	72	N-->V
2C8NB	72 - 76	72	N-->W
2C8NB	72 - 76	73	F-->D
2C8NB	72 - 76	73	F-->K
2C8NB	72 - 76	73	F-->M
2C8NB	72 - 76	73	F-->P
2C8NB	72 - 76	73	F-->V
2C8NB	72 - 76	74	V-->E
2C8NB	72 - 76	74	V-->L
2C8NB	72 - 76	74	V-->P
2C8NB	72 - 76	74	V-->S
2C8NB	72 - 76	74	V-->T
2C8NB	72 - 76	75	S-->A
2C8NB	72 - 76	75	S-->G
2C8NB	72 - 76	75	S-->I
2C8NB	72 - 76	75	S-->N
2C8NB	72 - 76	75	S-->P
2C8NB	72 - 76	75	S-->T

2C8NB	72	76	76	N-->I
2C8NB	72	76	76	N-->P
2C8NB	72	76	76	N-->S
1NRXA	141	145	141	L-->H
1NRXA	141	145	141	L-->Q
1NRXA	141	145	141	L-->S
1NRXA	141	145	141	L-->V
1NRXA	141	145	142	L-->E
1NRXA	141	145	142	L-->S
1NRXA	141	145	142	L-->V
1NRXA	141	145	142	L-->Y
1NRXA	141	145	143	A-->D
1NRXA	141	145	143	A-->E
1NRXA	141	145	143	A-->F
1NRXA	141	145	143	A-->L
1NRXA	141	145	143	A-->N
1NRXA	141	145	143	A-->P
1NRXA	141	145	143	A-->V
1NRXA	141	145	144	M-->A
1NRXA	141	145	144	M-->D
1NRXA	141	145	144	M-->G
1NRXA	141	145	144	M-->K
1NRXA	141	145	144	M-->L
1NRXA	141	145	144	M-->N
1NRXA	141	145	144	M-->P
1NRXA	141	145	144	M-->S
1NRXA	141	145	144	M-->V

1NRXA	141 - 145	144	M-->Y
1NRXA	141 - 145	145	V-->A
1NRXA	141 - 145	145	V-->D
1NRXA	141 - 145	145	V-->E
1NRXA	141 - 145	145	V-->P
1NRXA	384 - 390	384	N-->P
1NRXA	384 - 390	385	E-->Y
1NRXA	384 - 390	388	R-->T
1NRXA	384 - 390	389	V-->P
1NRXA	384 - 390	390	V-->H
1OCZM	37 - 41	37	L-->V
1OCZM	37 - 41	38	D-->G
1OCZM	37 - 41	38	D-->I
1OCZM	37 - 41	38	D-->L
1OCZM	37 - 41	38	D-->P
1OCZM	37 - 41	38	D-->Q
1OCZM	37 - 41	38	D-->T
1OCZM	37 - 41	38	D-->V
1OCZM	37 - 41	39	N-->A
1OCZM	37 - 41	39	N-->I
1OCZM	37 - 41	39	N-->L
1OCZM	37 - 41	39	N-->P
1OCZM	37 - 41	39	N-->R
1OCZM	37 - 41	40	Y-->D
1OCZM	37 - 41	40	Y-->I
1OCZM	37 - 41	40	Y-->K
1OCZM	37 - 41	40	Y-->L

1OCZM	37 - 41	40	Y-->Q
1OCZM	37 - 41	40	Y-->R
1OCZM	37 - 41	40	Y-->S
1OCZM	37 - 41	40	Y-->T
1OCZM	37 - 41	41	K-->C
1OCZM	37 - 41	41	K-->D
1OCZM	37 - 41	41	K-->I
1OCZM	37 - 41	41	K-->N
1OCZM	37 - 41	41	K-->R
1OCZM	37 - 41	41	K-->Y
1ES7D	783 - 788	786	Q-->T
1TKOA	96 - 102	102	E-->V
4CTSA	38 - 42	38	V-->G
4CTSA	38 - 42	38	V-->R
4CTSA	38 - 42	38	V-->W
4CTSA	38 - 42	39	D-->K
4CTSA	38 - 42	39	D-->Q
4CTSA	38 - 42	40	M-->Q
4CTSA	38 - 42	41	M-->D
4CTSA	38 - 42	41	M-->Q
4CTSA	38 - 42	41	M-->S
4CTSA	38 - 42	41	M-->V
2JDXA	327 - 332	327	I-->F
2JDXA	327 - 332	327	I-->S
2JDXA	327 - 332	330	F-->D
2JDXA	327 - 332	330	F-->K
2JDXA	327 - 332	330	F-->R

2JDXA	327 - 332	332	K-->H
2JDXA	327 - 332	332	K-->P
2JDXA	356 - 360	356	K-->G
2JDXA	356 - 360	356	K-->Q
2JDXA	356 - 360	357	W-->E
2JDXA	356 - 360	357	W-->I
2JDXA	356 - 360	357	W-->K
2JDXA	356 - 360	357	W-->N
2JDXA	356 - 360	357	W-->V
2JDXA	356 - 360	358	L-->I
2JDXA	356 - 360	360	M-->G
2JDXA	356 - 360	360	M-->N
2JDXA	356 - 360	360	M-->S
1RQFB	27 - 31	27	E-->L
1RQFB	27 - 31	27	E-->S
1RQFB	27 - 31	27	E-->T
1RQFB	27 - 31	28	D-->F
1RQFB	27 - 31	28	D-->I
1RQFB	27 - 31	29	Y-->A
1RQFB	27 - 31	29	Y-->G
1RQFB	27 - 31	29	Y-->S
1RQFB	27 - 31	30	I-->E
1RQFB	27 - 31	30	I-->G
1RQFB	27 - 31	31	Q-->T
1RQFB	27 - 31	31	Q-->V
1RQFB	27 - 31	31	Q-->Y
2O3OL	76 - 80	76	K-->A

2O3OL	76 - 80	76	K-->E
2O3OL	76 - 80	76	K-->I
2O3OL	76 - 80	76	K-->L
2O3OL	76 - 80	76	K-->P
2O3OL	76 - 80	76	K-->S
2O3OL	76 - 80	76	K-->T
2O3OL	76 - 80	76	K-->Y
2O3OL	76 - 80	77	E-->D
2O3OL	76 - 80	77	E-->F
2O3OL	76 - 80	77	E-->H
2O3OL	76 - 80	77	E-->I
2O3OL	76 - 80	77	E-->L
2O3OL	76 - 80	77	E-->P
2O3OL	76 - 80	77	E-->R
2O3OL	76 - 80	77	E-->T
2O3OL	76 - 80	77	E-->V
2O3OL	76 - 80	77	E-->Y
2O3OL	76 - 80	78	E-->D
2O3OL	76 - 80	78	E-->F
2O3OL	76 - 80	78	E-->G
2O3OL	76 - 80	78	E-->K
2O3OL	76 - 80	78	E-->L
2O3OL	76 - 80	78	E-->P
2O3OL	76 - 80	78	E-->R
2O3OL	76 - 80	78	E-->T
2O3OL	76 - 80	79	I-->D
2O3OL	76 - 80	79	I-->G

2O3OL	76 - 80	79	I-->K
2O3OL	76 - 80	79	I-->L
2O3OL	76 - 80	79	I-->M
2O3OL	76 - 80	79	I-->N
2O3OL	76 - 80	79	I-->S
2O3OL	76 - 80	79	I-->Y
2O3OL	76 - 80	80	E-->A
2O3OL	76 - 80	80	E-->F
2O3OL	76 - 80	80	E-->I
2O3OL	76 - 80	80	E-->L
2O3OL	76 - 80	80	E-->P
2O3OL	76 - 80	80	E-->R
2O3OL	76 - 80	80	E-->S
2OOYA	504 - 508	504	P-->Y
2OOYA	504 - 508	505	H-->E
2OOYA	504 - 508	505	H-->M
2OOYA	504 - 508	505	H-->Q
2OOYA	504 - 508	506	C-->L
2OOYA	504 - 508	506	C-->V
2OOYA	504 - 508	507	K-->W
2OOYA	504 - 508	508	R-->P
2OOYA	504 - 508	508	R-->S
2RFKA	246 - 250	246	M-->A
2RFKA	246 - 250	246	M-->E
2RFKA	246 - 250	246	M-->G
2RFKA	246 - 250	246	M-->H
2RFKA	246 - 250	246	M-->I

2RFKA	246	250	246	M-->P
2RFKA	246	250	246	M-->Q
2RFKA	246	250	246	M-->T
2RFKA	246	250	246	M-->V
2RFKA	246	250	247	E-->D
2RFKA	246	250	247	E-->F
2RFKA	246	250	247	E-->I
2RFKA	246	250	247	E-->K
2RFKA	246	250	247	E-->Q
2RFKA	246	250	247	E-->T
2RFKA	246	250	248	K-->M
2RFKA	246	250	248	K-->N
2RFKA	246	250	249	A-->D
2RFKA	246	250	249	A-->K
2RFKA	246	250	249	A-->M
2RFKA	246	250	249	A-->N
2RFKA	246	250	250	V-->D
2RFKA	246	250	250	V-->G
2RFKA	246	250	250	V-->L
2RFKA	246	250	250	V-->N
1Y56B	119	124	122	A-->P
1Y56B	119	124	123	K-->E
1Y56B	119	124	123	K-->L
1Y56B	119	124	124	E-->N
1Y56B	119	124	124	E-->V
1Y56B	293	297	293	P-->G
1Y56B	293	297	293	P-->I

1Y56B	293 - 297	293	P-->L
1Y56B	293 - 297	293	P-->R
1Y56B	293 - 297	293	P-->S
1Y56B	293 - 297	293	P-->Y
1Y56B	293 - 297	294	A-->D
1Y56B	293 - 297	294	A-->E
1Y56B	293 - 297	294	A-->K
1Y56B	293 - 297	294	A-->L
1Y56B	293 - 297	294	A-->M
1Y56B	293 - 297	294	A-->P
1Y56B	293 - 297	294	A-->R
1Y56B	293 - 297	294	A-->T
1Y56B	293 - 297	294	A-->V
1Y56B	293 - 297	294	A-->W
1Y56B	293 - 297	295	L-->G
1Y56B	293 - 297	295	L-->P
1Y56B	293 - 297	295	L-->S
1Y56B	293 - 297	296	K-->C
1Y56B	293 - 297	296	K-->D
1Y56B	293 - 297	296	K-->F
1Y56B	293 - 297	296	K-->G
1Y56B	293 - 297	296	K-->L
1Y56B	293 - 297	296	K-->N
1Y56B	293 - 297	296	K-->P
1Y56B	293 - 297	296	K-->Q
1Y56B	293 - 297	296	K-->S
1Y56B	293 - 297	296	K-->T

1Y56B	293 - 297	296	K-->V	
1Y56B	293 - 297	297	N-->A	
1Y56B	293 - 297	297	N-->D	
1Y56B	293 - 297	297	N-->E	
1Y56B	293 - 297	297	N-->F	
1Y56B	293 - 297	297	N-->G	
1Y56B	293 - 297	297	N-->H	
1Y56B	293 - 297	297	N-->I	
1Y56B	293 - 297	297	N-->P	
1Y56B	293 - 297	297	N-->Q	
1Y56B	293 - 297	297	N-->R	
1Y56B	293 - 297	297	N-->S	
1Y56B	293 - 297	297	N-->V	
1H1CA	185 - 189	185	V-->A	
1H1CA	185 - 189	185	V-->E	
1H1CA	185 - 189	185	V-->G	
1H1CA	185 - 189	185	V-->L	
1H1CA	185 - 189	185	V-->S	
1H1CA	185 - 189	186	D-->H	
1H1CA	185 - 189	186	D-->I	
1H1CA	185 - 189	186	D-->K	
1H1CA	185 - 189	186	D-->L	
1H1CA	185 - 189	186	D-->N	
1H1CA	185 - 189	186	D-->T	
1H1CA	185 - 189	186	D-->V	
1H1CA	185 - 189	187	F-->D	
1H1CA	185 - 189	187	F-->G	

1H1CA	185	189	187	F-->L
1H1CA	185	189	187	F-->M
1H1CA	185	189	187	F-->N
1H1CA	185	189	187	F-->P
1H1CA	185	189	187	F-->T
1H1CA	185	189	187	F-->V
1H1CA	185	189	187	F-->Y
1H1CA	185	189	188	L-->A
1H1CA	185	189	188	L-->C
1H1CA	185	189	188	L-->G
1H1CA	185	189	188	L-->K
1H1CA	185	189	188	L-->Q
1H1CA	185	189	188	L-->S
1H1CA	185	189	188	L-->V
1H1CA	185	189	189	K-->A
1H1CA	185	189	189	K-->E
1H1CA	185	189	189	K-->F
1H1CA	185	189	189	K-->G
1H1CA	185	189	189	K-->I
1H1CA	185	189	189	K-->L
1H1CA	185	189	189	K-->P
1H1CA	185	189	189	K-->Q
1H1CA	185	189	189	K-->S
1H1CA	185	189	189	K-->V
1H1CA	266	271	269	L-->V
1H1CA	266	271	271	E-->P
1H1CA	266	271	271	E-->V

1H1CA	321 - 325	321	R-->C
1H1CA	321 - 325	321	R-->D
1H1CA	321 - 325	321	R-->Y
1H1CA	321 - 325	322	E-->G
1H1CA	321 - 325	322	E-->S
1H1CA	321 - 325	322	E-->V
1H1CA	321 - 325	323	E-->G
1H1CA	321 - 325	323	E-->I
1H1CA	321 - 325	323	E-->L
1H1CA	321 - 325	323	E-->V
1H1CA	321 - 325	324	N-->A
1H1CA	321 - 325	324	N-->D
1H1CA	321 - 325	324	N-->G
1H1CA	321 - 325	324	N-->R
1H1CA	321 - 325	325	D-->G
1H1CA	321 - 325	325	D-->K
1H1CA	321 - 325	325	D-->L
1H1CA	321 - 325	325	D-->P
1H1CA	321 - 325	325	D-->V
1H1CA	327 - 332	328	L-->R
1H1CA	327 - 332	329	R-->G
1H1CA	327 - 332	329	R-->K
1H1CA	327 - 332	329	R-->L
1H1CA	327 - 332	332	E-->K
1SHZC	95 - 100	97	L-->C
1SHZC	116 - 120	116	P-->A
1SHZC	116 - 120	116	P-->G

1SHZC	116 - 120	116	P-->H
1SHZC	116 - 120	116	P-->K
1SHZC	116 - 120	116	P-->L
1SHZC	116 - 120	117	N-->D
1SHZC	116 - 120	117	N-->E
1SHZC	116 - 120	117	N-->H
1SHZC	116 - 120	117	N-->I
1SHZC	116 - 120	117	N-->K
1SHZC	116 - 120	117	N-->Q
1SHZC	116 - 120	117	N-->S
1SHZC	116 - 120	117	N-->V
1SHZC	116 - 120	117	N-->W
1SHZC	116 - 120	117	N-->Y
1SHZC	116 - 120	118	V-->F
1SHZC	116 - 120	118	V-->G
1SHZC	116 - 120	118	V-->Y
1SHZC	116 - 120	119	A-->D
1SHZC	116 - 120	119	A-->K
1SHZC	116 - 120	119	A-->R
1SHZC	116 - 120	119	A-->S
1SHZC	116 - 120	119	A-->V
1SHZC	116 - 120	119	A-->Y
1SHZC	116 - 120	120	F-->A
1SHZC	116 - 120	120	F-->E
1SHZC	116 - 120	120	F-->G
1SHZC	116 - 120	120	F-->I
1SHZC	116 - 120	120	F-->K

1SHZC	116 - 120	120	F-->L
1SHZC	116 - 120	120	F-->M
1SHZC	116 - 120	120	F-->P
1SHZC	116 - 120	120	F-->R
1SHZC	116 - 120	120	F-->Y
1SHZC	169 - 174	171	E-->M
1OGKD	141 - 145	141	E-->A
1OGKD	141 - 145	141	E-->D
1OGKD	141 - 145	141	E-->F
1OGKD	141 - 145	141	E-->G
1OGKD	141 - 145	141	E-->H
1OGKD	141 - 145	141	E-->I
1OGKD	141 - 145	141	E-->L
1OGKD	141 - 145	141	E-->M
1OGKD	141 - 145	141	E-->N
1OGKD	141 - 145	141	E-->P
1OGKD	141 - 145	141	E-->R
1OGKD	141 - 145	141	E-->S
1OGKD	141 - 145	141	E-->T
1OGKD	141 - 145	142	L-->A
1OGKD	141 - 145	142	L-->D
1OGKD	141 - 145	142	L-->E
1OGKD	141 - 145	142	L-->F
1OGKD	141 - 145	142	L-->G
1OGKD	141 - 145	142	L-->K
1OGKD	141 - 145	142	L-->P
1OGKD	141 - 145	142	L-->Q

1OGKD	141 - 145	142	L-->S
1OGKD	141 - 145	142	L-->T
1OGKD	141 - 145	142	L-->V
1OGKD	141 - 145	143	L-->A
1OGKD	141 - 145	143	L-->C
1OGKD	141 - 145	143	L-->D
1OGKD	141 - 145	143	L-->E
1OGKD	141 - 145	143	L-->I
1OGKD	141 - 145	143	L-->P
1OGKD	141 - 145	143	L-->Q
1OGKD	141 - 145	143	L-->R
1OGKD	141 - 145	143	L-->S
1OGKD	141 - 145	143	L-->T
1OGKD	141 - 145	143	L-->V
1OGKD	141 - 145	144	E-->A
1OGKD	141 - 145	144	E-->C
1OGKD	141 - 145	144	E-->D
1OGKD	141 - 145	144	E-->G
1OGKD	141 - 145	144	E-->H
1OGKD	141 - 145	144	E-->K
1OGKD	141 - 145	144	E-->L
1OGKD	141 - 145	144	E-->M
1OGKD	141 - 145	144	E-->N
1OGKD	141 - 145	144	E-->P
1OGKD	141 - 145	144	E-->S
1OGKD	141 - 145	144	E-->T
1OGKD	141 - 145	144	E-->V

1OGKD	141	- 145	144	E-->Y
1OGKD	141	- 145	145	L-->A
1OGKD	141	- 145	145	L-->D
1OGKD	141	- 145	145	L-->G
1OGKD	141	- 145	145	L-->K
1OGKD	141	- 145	145	L-->N
1OGKD	141	- 145	145	L-->P
1OGKD	141	- 145	145	L-->T
1OGKD	141	- 145	145	L-->V
1OGKD	141	- 145	145	L-->W
1MWJA	21	- 26	21	L-->V
1MWJA	21	- 26	22	S-->H
1MWJA	21	- 26	22	S-->Y
1MWJA	21	- 26	23	S-->Y
1MWJA	21	- 26	24	A-->D
1MWJA	21	- 26	24	A-->G
1MWJA	21	- 26	25	G-->K
1MWJA	21	- 26	25	G-->P
1MWJA	21	- 26	26	T-->R
2AF7A	3	- 7	3	R-->D
2AF7A	3	- 7	3	R-->E
2AF7A	3	- 7	3	R-->F
2AF7A	3	- 7	3	R-->G
2AF7A	3	- 7	3	R-->N
2AF7A	3	- 7	4	Y-->A
2AF7A	3	- 7	4	Y-->D
2AF7A	3	- 7	4	Y-->E

2AF7A	3 - 7	4	Y-->F
2AF7A	3 - 7	4	Y-->G
2AF7A	3 - 7	4	Y-->I
2AF7A	3 - 7	4	Y-->L
2AF7A	3 - 7	4	Y-->Q
2AF7A	3 - 7	4	Y-->T
2AF7A	3 - 7	4	Y-->V
2AF7A	3 - 7	5	R-->F
2AF7A	3 - 7	5	R-->T
2AF7A	3 - 7	5	R-->V
2AF7A	3 - 7	6	R-->D
2AF7A	3 - 7	6	R-->I
2AF7A	3 - 7	6	R-->L
2AF7A	3 - 7	6	R-->P
2AF7A	3 - 7	6	R-->S
2AF7A	3 - 7	6	R-->V
2AF7A	3 - 7	7	G-->R
2AF7A	3 - 7	7	G-->V
2AF7A	9 - 13	9	E-->A
2AF7A	9 - 13	9	E-->F
2AF7A	9 - 13	9	E-->G
2AF7A	9 - 13	9	E-->I
2AF7A	9 - 13	9	E-->L
2AF7A	9 - 13	9	E-->N
2AF7A	9 - 13	9	E-->S
2AF7A	9 - 13	9	E-->T
2AF7A	9 - 13	10	I-->G

2AF7A	9 - 13	10	I-->K
2AF7A	9 - 13	10	I-->Q
2AF7A	9 - 13	10	I-->S
2AF7A	9 - 13	10	I-->T
2AF7A	9 - 13	10	I-->V
2AF7A	9 - 13	11	L-->F
2AF7A	9 - 13	11	L-->S
2AF7A	9 - 13	11	L-->V
2AF7A	9 - 13	11	L-->W
2AF7A	9 - 13	12	N-->D
2AF7A	9 - 13	12	N-->E
2AF7A	9 - 13	12	N-->G
2AF7A	9 - 13	12	N-->T
2AF7A	9 - 13	12	N-->V
2AF7A	9 - 13	12	N-->Y
2AF7A	9 - 13	13	R-->M
2AF7A	9 - 13	13	R-->P
2AF7A	9 - 13	13	R-->T
2AF7A	9 - 13	13	R-->V
1Q6WC	53 - 57	53	V-->D
1Q6WC	53 - 57	54	E-->D
1Q6WC	53 - 57	54	E-->G
1Q6WC	53 - 57	54	E-->I
1Q6WC	53 - 57	54	E-->K
1Q6WC	53 - 57	54	E-->P
1Q6WC	53 - 57	54	E-->Q
1Q6WC	53 - 57	54	E-->R

1Q6WC	53 - 57	54	E-->S	
1Q6WC	53 - 57	54	E-->V	
1Q6WC	53 - 57	54	E-->W	
1Q6WC	53 - 57	55	F-->A	
1Q6WC	53 - 57	55	F-->G	
1Q6WC	53 - 57	55	F-->I	
1Q6WC	53 - 57	55	F-->L	
1Q6WC	53 - 57	55	F-->N	
1Q6WC	53 - 57	55	F-->P	
1Q6WC	53 - 57	55	F-->R	
1Q6WC	53 - 57	55	F-->S	
1Q6WC	53 - 57	55	F-->V	
1Q6WC	53 - 57	55	F-->Y	
1Q6WC	53 - 57	56	A-->C	
1Q6WC	53 - 57	56	A-->D	
1Q6WC	53 - 57	56	A-->E	
1Q6WC	53 - 57	56	A-->R	
1Q6WC	53 - 57	56	A-->S	
1Q6WC	53 - 57	57	K-->A	
1Q6WC	53 - 57	57	K-->I	
1Q6WC	53 - 57	57	K-->L	
1Q6WC	53 - 57	57	K-->P	
1Q6WC	53 - 57	57	K-->Q	
1Q6WC	53 - 57	57	K-->S	
1Q6WC	53 - 57	57	K-->T	
1Q6WC	53 - 57	57	K-->V	
1Q6WC	79 - 84	80	D-->L	

1Q6WC	79 - 84	80	D-->W	
1Q6WC	79 - 84	81	Q-->M	
1Q6WC	79 - 84	82	V-->S	
1Q6WC	79 - 84	83	I-->F	
1Q6WC	79 - 84	84	L-->I	
1Q6WC	79 - 84	84	L-->M	
1T6IB	65 - 70	68	F-->L	
1T6IB	65 - 70	69	E-->D	
1ZGLB	67 - 72	71	R-->A	
1ZGLB	67 - 72	71	R-->S	
1ZGLB	67 - 72	71	R-->Y	
1YEWA	40 - 44	40	A-->D	
1YEWA	40 - 44	42	M-->K	
1YEWA	40 - 44	44	M-->A	
1YEWA	369 - 373	369	A-->D	
1YEWA	369 - 373	369	A-->G	
1YEWA	369 - 373	369	A-->T	
1YEWA	369 - 373	370	A-->T	
1YEWA	369 - 373	370	A-->V	
1YEWA	369 - 373	370	A-->Y	
1YEWA	369 - 373	371	W-->A	
1YEWA	369 - 373	371	W-->E	
1YEWA	369 - 373	371	W-->F	
1YEWA	369 - 373	371	W-->G	
1YEWA	369 - 373	371	W-->H	
1YEWA	369 - 373	371	W-->I	
1YEWA	369 - 373	371	W-->L	

1YEWA	369	- 373	371	W-->N
1YEWA	369	- 373	371	W-->P
1YEWA	369	- 373	371	W-->Q
1YEWA	369	- 373	371	W-->R
1YEWA	369	- 373	371	W-->T
1YEWA	369	- 373	371	W-->V
1YEWA	369	- 373	371	W-->Y
1YEWA	369	- 373	372	E-->D
1YEWA	369	- 373	372	E-->K
1YEWA	369	- 373	372	E-->Q
1YEWA	369	- 373	372	E-->S
1YEWA	369	- 373	373	V-->N
1YEWA	369	- 373	373	V-->S
1YEWA	376	- 381	376	L-->V
1YEWA	376	- 381	379	I-->E
1YEWA	376	- 381	380	I-->A
1YEWA	376	- 381	381	Y-->D
1YEWC	83	- 88	83	P-->F
1YEWC	83	- 88	84	E-->G
1YEWC	83	- 88	86	E-->L
2J01N	111	- 117	115	R-->I
2J017	18	- 23	22	M-->L
2C37A	215	- 220	219	S-->E
2C37A	215	- 220	220	I-->N
2GK9A	73	- 77	73	P-->A
2GK9A	73	- 77	73	P-->E
2GK9A	73	- 77	73	P-->G

2GK9A	73 - 77	73	P-->I
2GK9A	73 - 77	73	P-->V
2GK9A	73 - 77	74	D-->E
2GK9A	73 - 77	74	D-->G
2GK9A	73 - 77	74	D-->I
2GK9A	73 - 77	74	D-->L
2GK9A	73 - 77	74	D-->M
2GK9A	73 - 77	74	D-->N
2GK9A	73 - 77	74	D-->S
2GK9A	73 - 77	74	D-->T
2GK9A	73 - 77	74	D-->V
2GK9A	73 - 77	75	D-->A
2GK9A	73 - 77	75	D-->H
2GK9A	73 - 77	75	D-->S
2GK9A	73 - 77	76	F-->A
2GK9A	73 - 77	76	F-->D
2GK9A	73 - 77	76	F-->G
2GK9A	73 - 77	76	F-->I
2GK9A	73 - 77	76	F-->K
2GK9A	73 - 77	76	F-->N
2GK9A	73 - 77	76	F-->S
2GK9A	73 - 77	77	K-->A
2GK9A	73 - 77	77	K-->E
2GK9A	73 - 77	77	K-->H
2GK9A	73 - 77	77	K-->I
2GK9A	73 - 77	77	K-->L
2GK9A	73 - 77	77	K-->N

2GK9A	73	77	77	K-->P
2GK9A	73	77	77	K-->Q
2GK9A	73	77	77	K-->T
2A7WA	73	77	73	V-->F
2A7WA	73	77	73	V-->K
2A7WA	73	77	73	V-->P
2A7WA	73	77	73	V-->S
2A7WA	73	77	73	V-->Y
2A7WA	73	77	74	L-->A
2A7WA	73	77	74	L-->D
2A7WA	73	77	74	L-->K
2A7WA	73	77	74	L-->N
2A7WA	73	77	74	L-->S
2A7WA	73	77	74	L-->T
2A7WA	73	77	74	L-->V
2A7WA	73	77	74	L-->Y
2A7WA	73	77	75	L-->A
2A7WA	73	77	75	L-->D
2A7WA	73	77	75	L-->E
2A7WA	73	77	75	L-->F
2A7WA	73	77	75	L-->G
2A7WA	73	77	75	L-->I
2A7WA	73	77	75	L-->K
2A7WA	73	77	75	L-->M
2A7WA	73	77	75	L-->N
2A7WA	73	77	75	L-->P
2A7WA	73	77	75	L-->Q

2A7WA	73 - 77	75	L-->S
2A7WA	73 - 77	75	L-->V
2A7WA	73 - 77	76	T-->A
2A7WA	73 - 77	76	T-->D
2A7WA	73 - 77	76	T-->E
2A7WA	73 - 77	76	T-->G
2A7WA	73 - 77	76	T-->I
2A7WA	73 - 77	76	T-->K
2A7WA	73 - 77	76	T-->P
2A7WA	73 - 77	76	T-->R
2A7WA	73 - 77	76	T-->S
2A7WA	73 - 77	76	T-->W
2A7WA	73 - 77	77	Y-->C
2A7WA	73 - 77	77	Y-->D
2A7WA	73 - 77	77	Y-->E
2A7WA	73 - 77	77	Y-->G
2A7WA	73 - 77	77	Y-->H
2A7WA	73 - 77	77	Y-->K
2A7WA	73 - 77	77	Y-->L
2A7WA	73 - 77	77	Y-->N
2A7WA	73 - 77	77	Y-->P
2A7WA	73 - 77	77	Y-->Q
2A7WA	73 - 77	77	Y-->R
2A7WA	73 - 77	77	Y-->T
2A7WA	73 - 77	77	Y-->V
2A7WA	82 - 86	82	P-->A
2A7WA	82 - 86	82	P-->E

2A7WA	82 - 86	82	P-->G
2A7WA	82 - 86	82	P-->I
2A7WA	82 - 86	82	P-->R
2A7WA	82 - 86	82	P-->S
2A7WA	82 - 86	82	P-->V
2A7WA	82 - 86	83	E-->A
2A7WA	82 - 86	83	E-->D
2A7WA	82 - 86	83	E-->F
2A7WA	82 - 86	83	E-->G
2A7WA	82 - 86	83	E-->H
2A7WA	82 - 86	83	E-->I
2A7WA	82 - 86	83	E-->L
2A7WA	82 - 86	83	E-->R
2A7WA	82 - 86	83	E-->S
2A7WA	82 - 86	83	E-->T
2A7WA	82 - 86	83	E-->V
2A7WA	82 - 86	83	E-->Y
2A7WA	82 - 86	84	D-->A
2A7WA	82 - 86	84	D-->E
2A7WA	82 - 86	84	D-->G
2A7WA	82 - 86	84	D-->H
2A7WA	82 - 86	84	D-->I
2A7WA	82 - 86	84	D-->K
2A7WA	82 - 86	84	D-->P
2A7WA	82 - 86	84	D-->R
2A7WA	82 - 86	84	D-->V
2A7WA	82 - 86	84	D-->W

2A7WA	82	86	84	D-->Y
2A7WA	82	86	85	V-->A
2A7WA	82	86	85	V-->C
2A7WA	82	86	85	V-->E
2A7WA	82	86	85	V-->F
2A7WA	82	86	85	V-->G
2A7WA	82	86	85	V-->I
2A7WA	82	86	85	V-->K
2A7WA	82	86	85	V-->L
2A7WA	82	86	85	V-->M
2A7WA	82	86	85	V-->N
2A7WA	82	86	85	V-->R
2A7WA	82	86	85	V-->T
2A7WA	82	86	85	V-->Y
2A7WA	82	86	86	V-->A
2A7WA	82	86	86	V-->D
2A7WA	82	86	86	V-->E
2A7WA	82	86	86	V-->G
2A7WA	82	86	86	V-->H
2A7WA	82	86	86	V-->K
2A7WA	82	86	86	V-->P
2A7WA	82	86	86	V-->Q
2A7WA	82	86	86	V-->R
2A7WA	82	86	86	V-->S
2A7WA	82	86	86	V-->Y
2A7WA	88	92	88	E-->D
2A7WA	88	92	88	E-->L

2A7WA	88 - 92	89	L-->A
2A7WA	88 - 92	89	L-->N
2A7WA	88 - 92	89	L-->P
2A7WA	88 - 92	89	L-->T
2A7WA	88 - 92	90	H-->K
2A7WA	88 - 92	90	H-->M
2A7WA	88 - 92	90	H-->R
2A7WA	88 - 92	90	H-->T
2A7WA	88 - 92	90	H-->V
2A7WA	88 - 92	90	H-->Y
2A7WA	88 - 92	91	R-->D
2A7WA	88 - 92	91	R-->G
2A7WA	88 - 92	91	R-->V
2A7WA	88 - 92	92	R-->E
2A7WA	88 - 92	92	R-->H
2A7WA	88 - 92	92	R-->S
2A7WA	88 - 92	92	R-->T
1NUBA	119 - 123	119	K-->D
1NUBA	119 - 123	119	K-->F
1NUBA	119 - 123	119	K-->I
1NUBA	119 - 123	119	K-->V
1NUBA	119 - 123	120	K-->S
1NUBA	119 - 123	121	G-->I
1NUBA	119 - 123	121	G-->K
1NUBA	119 - 123	121	G-->L
1NUBA	119 - 123	122	H-->A
1NUBA	119 - 123	122	H-->C

1NUBA	119 - 123	122	H-->D	
1NUBA	119 - 123	122	H-->E	
1NUBA	119 - 123	122	H-->F	
1NUBA	119 - 123	122	H-->G	
1NUBA	119 - 123	122	H-->I	
1NUBA	119 - 123	122	H-->K	
1NUBA	119 - 123	122	H-->L	
1NUBA	119 - 123	122	H-->Q	
1NUBA	119 - 123	122	H-->S	
1NUBA	119 - 123	122	H-->V	
1NUBA	119 - 123	123	K-->A	
1NUBA	119 - 123	123	K-->H	
1NUBA	119 - 123	123	K-->I	
1NUBA	119 - 123	123	K-->M	
1NUBA	119 - 123	123	K-->Q	
1NUBA	119 - 123	123	K-->T	
1NUBA	119 - 123	123	K-->W	
1NUBA	266 - 272	268	E-->H	
1AVOB	234 - 238	234	F-->D	
1AVOB	234 - 238	234	F-->H	
1AVOB	234 - 238	234	F-->I	
1AVOB	234 - 238	234	F-->K	
1AVOB	234 - 238	234	F-->L	
1AVOB	234 - 238	234	F-->N	
1AVOB	234 - 238	234	F-->S	
1AVOB	234 - 238	234	F-->V	
1AVOB	234 - 238	234	F-->Y	

1AVOB	234 - 238	235	E-->D
1AVOB	234 - 238	235	E-->H
1AVOB	234 - 238	235	E-->K
1AVOB	234 - 238	235	E-->L
1AVOB	234 - 238	235	E-->P
1AVOB	234 - 238	235	E-->S
1AVOB	234 - 238	235	E-->T
1AVOB	234 - 238	235	E-->V
1AVOB	234 - 238	236	K-->C
1AVOB	234 - 238	236	K-->E
1AVOB	234 - 238	236	K-->G
1AVOB	234 - 238	236	K-->L
1AVOB	234 - 238	236	K-->T
1AVOB	234 - 238	237	L-->A
1AVOB	234 - 238	237	L-->D
1AVOB	234 - 238	237	L-->E
1AVOB	234 - 238	237	L-->F
1AVOB	234 - 238	237	L-->P
1AVOB	234 - 238	237	L-->Q
1AVOB	234 - 238	237	L-->R
1AVOB	234 - 238	237	L-->S
1AVOB	234 - 238	237	L-->V
1AVOB	234 - 238	237	L-->Y
1AVOB	234 - 238	238	K-->D
1AVOB	234 - 238	238	K-->E
1AVOB	234 - 238	238	K-->G
1AVOB	234 - 238	238	K-->I

1AVOB	234 - 238	238	K-->M
1AVOB	234 - 238	238	K-->Q
1AVOB	234 - 238	238	K-->T
1AVOB	234 - 238	238	K-->V
1NSKL	13 - 17	13	P-->A
1NSKL	13 - 17	13	P-->G
1NSKL	13 - 17	13	P-->I
1NSKL	13 - 17	13	P-->L
1NSKL	13 - 17	13	P-->V
1NSKL	13 - 17	14	D-->A
1NSKL	13 - 17	14	D-->C
1NSKL	13 - 17	14	D-->I
1NSKL	13 - 17	14	D-->K
1NSKL	13 - 17	14	D-->L
1NSKL	13 - 17	14	D-->Q
1NSKL	13 - 17	14	D-->S
1NSKL	13 - 17	14	D-->V
1NSKL	13 - 17	15	G-->D
1NSKL	13 - 17	15	G-->S
1NSKL	13 - 17	15	G-->T
1NSKL	13 - 17	15	G-->Y
1NSKL	13 - 17	16	V-->G
1NSKL	13 - 17	16	V-->H
1NSKL	13 - 17	16	V-->K
1NSKL	13 - 17	16	V-->L
1NSKL	13 - 17	16	V-->M
1NSKL	13 - 17	16	V-->N

1NSKL	13 - 17	16	V-->Q
1NSKL	13 - 17	16	V-->S
1NSKL	13 - 17	17	Q-->A
1NSKL	13 - 17	17	Q-->D
1NSKL	13 - 17	17	Q-->F
1NSKL	13 - 17	17	Q-->G
1NSKL	13 - 17	17	Q-->H
1NSKL	13 - 17	17	Q-->I
1NSKL	13 - 17	17	Q-->K
1NSKL	13 - 17	17	Q-->L
1NSKL	13 - 17	17	Q-->M
1NSKL	13 - 17	17	Q-->N
1NSKL	13 - 17	17	Q-->P
1NSKL	13 - 17	17	Q-->R
1NSKL	13 - 17	17	Q-->T
1NSKL	13 - 17	17	Q-->V
1NSKL	13 - 17	17	Q-->W
1NSKL	104 - 108	104	I-->A
1NSKL	104 - 108	104	I-->E
1NSKL	104 - 108	104	I-->G
1NSKL	104 - 108	104	I-->H
1NSKL	104 - 108	104	I-->L
1NSKL	104 - 108	104	I-->M
1NSKL	104 - 108	104	I-->R
1NSKL	104 - 108	104	I-->V
1NSKL	104 - 108	104	I-->W
1NSKL	104 - 108	105	R-->D

1NSKL	104 - 108	105	R-->E
1NSKL	104 - 108	105	R-->G
1NSKL	104 - 108	105	R-->H
1NSKL	104 - 108	105	R-->I
1NSKL	104 - 108	105	R-->K
1NSKL	104 - 108	105	R-->M
1NSKL	104 - 108	105	R-->P
1NSKL	104 - 108	105	R-->S
1NSKL	104 - 108	105	R-->T
1NSKL	104 - 108	106	G-->A
1NSKL	104 - 108	106	G-->D
1NSKL	104 - 108	106	G-->E
1NSKL	104 - 108	106	G-->N
1NSKL	104 - 108	106	G-->S
1NSKL	104 - 108	106	G-->T
1NSKL	104 - 108	106	G-->V
1NSKL	104 - 108	106	G-->Y
1NSKL	104 - 108	107	D-->G
1NSKL	104 - 108	107	D-->P
1NSKL	104 - 108	107	D-->S
1NSKL	104 - 108	108	F-->A
1NSKL	104 - 108	108	F-->E
1NSKL	104 - 108	108	F-->G
1NSKL	104 - 108	108	F-->K
1NSKL	104 - 108	108	F-->L
1NSKL	104 - 108	108	F-->N
1NSKL	104 - 108	108	F-->S

1NSKL	104 - 108	108	F-->T
1NSKL	104 - 108	108	F-->V
2D5HA	360 - 365	363	R-->F
2D5HA	360 - 365	365	F-->D
2D5HA	455 - 459	455	I-->A
2D5HA	455 - 459	455	I-->D
2D5HA	455 - 459	455	I-->G
2D5HA	455 - 459	455	I-->Q
2D5HA	455 - 459	455	I-->S
2D5HA	455 - 459	455	I-->Y
2D5HA	455 - 459	456	K-->A
2D5HA	455 - 459	456	K-->D
2D5HA	455 - 459	456	K-->E
2D5HA	455 - 459	456	K-->G
2D5HA	455 - 459	456	K-->L
2D5HA	455 - 459	456	K-->M
2D5HA	455 - 459	457	D-->E
2D5HA	455 - 459	457	D-->G
2D5HA	455 - 459	457	D-->K
2D5HA	455 - 459	457	D-->L
2D5HA	455 - 459	457	D-->S
2D5HA	455 - 459	457	D-->T
2D5HA	455 - 459	457	D-->V
2D5HA	455 - 459	457	D-->Y
2D5HA	455 - 459	458	V-->G
2D5HA	455 - 459	458	V-->K
2D5HA	455 - 459	458	V-->S

2D5HA	455 - 459	458	V-->W
2D5HA	455 - 459	459	F-->D
2D5HA	455 - 459	459	F-->G
2D5HA	455 - 459	459	F-->I
2D5HA	455 - 459	459	F-->K
2D5HA	455 - 459	459	F-->L
2D5HA	455 - 459	459	F-->N
2D5HA	455 - 459	459	F-->P
2D5HA	455 - 459	459	F-->Q
2D5HA	455 - 459	459	F-->R
2D5HA	455 - 459	459	F-->T
2D5HA	455 - 459	459	F-->V
2D5HA	455 - 459	459	F-->W
2D5HA	455 - 459	459	F-->Y
2E1MC	593 - 597	593	R-->D
2E1MC	593 - 597	593	R-->E
2E1MC	593 - 597	593	R-->I
2E1MC	593 - 597	593	R-->K
2E1MC	593 - 597	593	R-->L
2E1MC	593 - 597	593	R-->N
2E1MC	593 - 597	593	R-->Q
2E1MC	593 - 597	593	R-->S
2E1MC	593 - 597	593	R-->V
2E1MC	593 - 597	593	R-->W
2E1MC	593 - 597	594	R-->A
2E1MC	593 - 597	594	R-->D
2E1MC	593 - 597	594	R-->E

2E1MC	593 - 597	594	R-->F
2E1MC	593 - 597	594	R-->G
2E1MC	593 - 597	594	R-->I
2E1MC	593 - 597	594	R-->K
2E1MC	593 - 597	594	R-->L
2E1MC	593 - 597	594	R-->M
2E1MC	593 - 597	594	R-->N
2E1MC	593 - 597	594	R-->P
2E1MC	593 - 597	594	R-->Q
2E1MC	593 - 597	594	R-->S
2E1MC	593 - 597	594	R-->V
2E1MC	593 - 597	595	I-->A
2E1MC	593 - 597	595	I-->G
2E1MC	593 - 597	595	I-->H
2E1MC	593 - 597	595	I-->K
2E1MC	593 - 597	595	I-->L
2E1MC	593 - 597	595	I-->N
2E1MC	593 - 597	595	I-->P
2E1MC	593 - 597	595	I-->S
2E1MC	593 - 597	595	I-->T
2E1MC	593 - 597	595	I-->Y
2E1MC	593 - 597	596	E-->A
2E1MC	593 - 597	596	E-->D
2E1MC	593 - 597	596	E-->G
2E1MC	593 - 597	596	E-->I
2E1MC	593 - 597	596	E-->L
2E1MC	593 - 597	596	E-->N

2E1MC	593	597	596	E-->S
2E1MC	593	597	596	E-->T
2E1MC	593	597	597	V-->C
2E1MC	593	597	597	V-->N
2E1MC	593	597	597	V-->R
2P7NA	178	183	181	L-->S
2P7NA	178	183	183	D-->S
2P7NA	318	322	318	T-->A
2P7NA	318	322	318	T-->P
2P7NA	318	322	318	T-->S
2P7NA	318	322	318	T-->V
2P7NA	318	322	319	P-->A
2P7NA	318	322	319	P-->C
2P7NA	318	322	319	P-->D
2P7NA	318	322	319	P-->E
2P7NA	318	322	319	P-->L
2P7NA	318	322	319	P-->N
2P7NA	318	322	319	P-->S
2P7NA	318	322	319	P-->T
2P7NA	318	322	320	I-->E
2P7NA	318	322	320	I-->F
2P7NA	318	322	320	I-->G
2P7NA	318	322	320	I-->K
2P7NA	318	322	320	I-->L
2P7NA	318	322	320	I-->N
2P7NA	318	322	321	D-->A
2P7NA	318	322	321	D-->G

2P7NA	318	322	321	D-->I
2P7NA	318	322	321	D-->K
2P7NA	318	322	321	D-->M
2P7NA	318	322	321	D-->T
2P7NA	318	322	322	T-->A
2P7NA	318	322	322	T-->E
2P7NA	318	322	322	T-->M
2P7NA	318	322	322	T-->R
2P7NA	318	322	322	T-->S
2P7NA	318	322	322	T-->V
2P7NA	318	322	322	T-->W
2P7NA	318	322	322	T-->Y
2P7NA	324	329	324	I-->T
2P7NA	324	329	325	R-->C
2P7NA	324	329	325	R-->F
2P7NA	324	329	325	R-->H
2P7NA	324	329	327	V-->Q
2P7NA	324	329	329	A-->K
1N25A	490	494	490	G-->A
1N25A	490	494	490	G-->C
1N25A	490	494	490	G-->E
1N25A	490	494	490	G-->I
1N25A	490	494	490	G-->L
1N25A	490	494	490	G-->M
1N25A	490	494	490	G-->Q
1N25A	490	494	490	G-->T
1N25A	490	494	490	G-->V

1N25A	490	494	490	G-->Y
1N25A	490	494	491	I-->C
1N25A	490	494	491	I-->F
1N25A	490	494	491	I-->G
1N25A	490	494	491	I-->K
1N25A	490	494	491	I-->L
1N25A	490	494	491	I-->R
1N25A	490	494	491	I-->S
1N25A	490	494	491	I-->T
1N25A	490	494	491	I-->V
1N25A	490	494	491	I-->Y
1N25A	490	494	492	N-->A
1N25A	490	494	492	N-->D
1N25A	490	494	492	N-->F
1N25A	490	494	492	N-->G
1N25A	490	494	492	N-->H
1N25A	490	494	492	N-->K
1N25A	490	494	492	N-->L
1N25A	490	494	492	N-->M
1N25A	490	494	492	N-->P
1N25A	490	494	492	N-->T
1N25A	490	494	492	N-->V
1N25A	490	494	492	N-->Y
1N25A	490	494	493	N-->A
1N25A	490	494	493	N-->G
1N25A	490	494	493	N-->H
1N25A	490	494	493	N-->I

1N25A	490	- 494	493	N-->K
1N25A	490	- 494	493	N-->L
1N25A	490	- 494	493	N-->Q
1N25A	490	- 494	493	N-->R
1N25A	490	- 494	493	N-->S
1N25A	490	- 494	493	N-->T
1N25A	490	- 494	493	N-->V
1N25A	490	- 494	494	L-->G
1N25A	490	- 494	494	L-->K
1N25A	490	- 494	494	L-->R
1N25A	490	- 494	494	L-->S
1N25A	498	- 502	498	R-->C
1N25A	498	- 502	498	R-->D
1N25A	498	- 502	498	R-->E
1N25A	498	- 502	498	R-->F
1N25A	498	- 502	498	R-->G
1N25A	498	- 502	498	R-->H
1N25A	498	- 502	498	R-->I
1N25A	498	- 502	498	R-->L
1N25A	498	- 502	498	R-->N
1N25A	498	- 502	498	R-->P
1N25A	498	- 502	498	R-->T
1N25A	498	- 502	499	D-->E
1N25A	498	- 502	499	D-->G
1N25A	498	- 502	499	D-->I
1N25A	498	- 502	499	D-->K
1N25A	498	- 502	499	D-->L

1N25A	498	502	499	D-->R
1N25A	498	502	499	D-->T
1N25A	498	502	499	D-->V
1N25A	498	502	500	Y-->E
1N25A	498	502	500	Y-->F
1N25A	498	502	500	Y-->H
1N25A	498	502	500	Y-->L
1N25A	498	502	500	Y-->R
1N25A	498	502	500	Y-->S
1N25A	498	502	500	Y-->T
1N25A	498	502	501	L-->A
1N25A	498	502	501	L-->C
1N25A	498	502	501	L-->F
1N25A	498	502	501	L-->I
1N25A	498	502	501	L-->P
1N25A	498	502	501	L-->R
1N25A	498	502	501	L-->S
1N25A	498	502	501	L-->T
1N25A	498	502	501	L-->Y
1N25A	498	502	502	D-->P
1N25A	498	502	502	D-->R
1N25A	498	502	502	D-->T
1N25A	498	502	502	D-->V
1N25A	498	502	502	D-->W
1OF5B	155	159	155	D-->E
1OF5B	155	159	155	D-->F
1OF5B	155	159	155	D-->V

1OF5B	155 - 159	155	D-->Y
1OF5B	155 - 159	156	R-->G
1OF5B	155 - 159	156	R-->L
1OF5B	155 - 159	156	R-->P
1OF5B	155 - 159	156	R-->S
1OF5B	155 - 159	156	R-->V
1OF5B	155 - 159	157	I-->E
1OF5B	155 - 159	157	I-->G
1OF5B	155 - 159	157	I-->K
1OF5B	155 - 159	157	I-->R
1OF5B	155 - 159	157	I-->W
1OF5B	155 - 159	158	F-->A
1OF5B	155 - 159	158	F-->E
1OF5B	155 - 159	158	F-->G
1OF5B	155 - 159	158	F-->I
1OF5B	155 - 159	158	F-->L
1OF5B	155 - 159	158	F-->N
1OF5B	155 - 159	158	F-->P
1OF5B	155 - 159	158	F-->V
1OF5B	155 - 159	159	R-->C
1OF5B	155 - 159	159	R-->F
1OF5B	155 - 159	159	R-->T
1OF5B	155 - 159	159	R-->V
1OF5B	155 - 159	159	R-->W
1F45B	66 - 70	66	L-->A
1F45B	66 - 70	66	L-->C
1F45B	66 - 70	66	L-->E

1F45B	66 - 70	66	L-->I
1F45B	66 - 70	66	L-->P
1F45B	66 - 70	66	L-->R
1F45B	66 - 70	66	L-->V
1F45B	66 - 70	66	L-->W
1F45B	66 - 70	67	E-->A
1F45B	66 - 70	67	E-->C
1F45B	66 - 70	67	E-->D
1F45B	66 - 70	67	E-->F
1F45B	66 - 70	67	E-->G
1F45B	66 - 70	67	E-->I
1F45B	66 - 70	67	E-->K
1F45B	66 - 70	67	E-->N
1F45B	66 - 70	67	E-->P
1F45B	66 - 70	67	E-->Q
1F45B	66 - 70	67	E-->R
1F45B	66 - 70	67	E-->S
1F45B	66 - 70	67	E-->T
1F45B	66 - 70	67	E-->V
1F45B	66 - 70	67	E-->W
1F45B	66 - 70	68	L-->I
1F45B	66 - 70	68	L-->M
1F45B	66 - 70	68	L-->S
1F45B	66 - 70	68	L-->T
1F45B	66 - 70	68	L-->Y
1F45B	66 - 70	69	T-->D
1F45B	66 - 70	69	T-->E

1F45B	66 - 70	69	T-->K
1F45B	66 - 70	69	T-->P
1F45B	66 - 70	70	K-->A
1F45B	66 - 70	70	K-->C
1F45B	66 - 70	70	K-->D
1F45B	66 - 70	70	K-->F
1F45B	66 - 70	70	K-->G
1F45B	66 - 70	70	K-->H
1F45B	66 - 70	70	K-->I
1F45B	66 - 70	70	K-->L
1F45B	66 - 70	70	K-->Q
1F45B	66 - 70	70	K-->R
1F45B	66 - 70	70	K-->T
1F45B	66 - 70	70	K-->Y
1W2EA	51 - 56	51	A-->G
1W2EA	51 - 56	52	D-->E
1W2EA	51 - 56	52	D-->K
1W2EA	51 - 56	52	D-->N
1W2EA	51 - 56	53	R-->N
1W2EA	51 - 56	54	V-->Y
1W2EA	51 - 56	55	A-->F
1W2EA	51 - 56	55	A-->G
1W2EA	51 - 56	55	A-->I
1W2EA	51 - 56	55	A-->L
1W2EA	51 - 56	55	A-->Q
1W2EA	51 - 56	55	A-->T
1W2EA	51 - 56	56	V-->F

2AWOD	228 - 233	229	F-->T
2AWOD	228 - 233	231	A-->H
2AWOD	228 - 233	231	A-->I
2R9BA	50 - 54	50	A-->G
2R9BA	50 - 54	50	A-->H
2R9BA	50 - 54	50	A-->K
2R9BA	50 - 54	50	A-->L
2R9BA	50 - 54	50	A-->Q
2R9BA	50 - 54	50	A-->V
2R9BA	50 - 54	51	G-->E
2R9BA	50 - 54	51	G-->Q
2R9BA	50 - 54	51	G-->R
2R9BA	50 - 54	51	G-->S
2R9BA	50 - 54	52	C-->A
2R9BA	50 - 54	52	C-->D
2R9BA	50 - 54	52	C-->G
2R9BA	50 - 54	52	C-->H
2R9BA	50 - 54	52	C-->I
2R9BA	50 - 54	52	C-->K
2R9BA	50 - 54	52	C-->L
2R9BA	50 - 54	52	C-->N
2R9BA	50 - 54	52	C-->P
2R9BA	50 - 54	52	C-->S
2R9BA	50 - 54	52	C-->T
2R9BA	50 - 54	52	C-->V
2R9BA	50 - 54	52	C-->Y
2R9BA	50 - 54	53	L-->K

2R9BA	50	54	53	L-->Q
2R9BA	50	54	53	L-->T
2R9BA	50	54	53	L-->V
2R9BA	50	54	54	T-->D
2R9BA	50	54	54	T-->H
2R9BA	50	54	54	T-->L
2R9BA	50	54	54	T-->N
2R9BA	50	54	54	T-->Q
2R9BA	50	54	54	T-->V
1SZIA	212	216	212	E-->D
1SZIA	212	216	212	E-->G
1SZIA	212	216	212	E-->N
1SZIA	212	216	212	E-->S
1SZIA	212	216	212	E-->T
1SZIA	212	216	212	E-->V
1SZIA	212	216	212	E-->Y
1SZIA	212	216	213	A-->E
1SZIA	212	216	213	A-->G
1SZIA	212	216	213	A-->I
1SZIA	212	216	213	A-->P
1SZIA	212	216	213	A-->S
1SZIA	212	216	213	A-->T
1SZIA	212	216	213	A-->V
1SZIA	212	216	214	E-->A
1SZIA	212	216	214	E-->D
1SZIA	212	216	214	E-->G
1SZIA	212	216	214	E-->K

1SZIA	212 - 216	214	E-->L
1SZIA	212 - 216	214	E-->P
1SZIA	212 - 216	214	E-->T
1SZIA	212 - 216	214	E-->V
1SZIA	212 - 216	215	L-->A
1SZIA	212 - 216	215	L-->D
1SZIA	212 - 216	215	L-->E
1SZIA	212 - 216	215	L-->F
1SZIA	212 - 216	215	L-->G
1SZIA	212 - 216	215	L-->I
1SZIA	212 - 216	215	L-->N
1SZIA	212 - 216	215	L-->W
1SZIA	212 - 216	216	A-->C
1SZIA	212 - 216	216	A-->D
1SZIA	212 - 216	216	A-->E
1SZIA	212 - 216	216	A-->G
1SZIA	212 - 216	216	A-->H
1SZIA	212 - 216	216	A-->L
1SZIA	212 - 216	216	A-->N
1SZIA	212 - 216	216	A-->P
1SZIA	212 - 216	216	A-->Q
1SZIA	212 - 216	216	A-->S
1SZIA	212 - 216	216	A-->T
1SZIA	212 - 216	216	A-->V
2ZIHB	116 - 120	116	S-->A
2ZIHB	116 - 120	116	S-->L
2ZIHB	116 - 120	116	S-->R

2ZIHB	116 - 120	117	A-->F
2ZIHB	116 - 120	117	A-->K
2ZIHB	116 - 120	117	A-->L
2ZIHB	116 - 120	118	R-->C
2ZIHB	116 - 120	118	R-->E
2ZIHB	116 - 120	118	R-->G
2ZIHB	116 - 120	118	R-->I
2ZIHB	116 - 120	118	R-->P
2ZIHB	116 - 120	119	K-->G
2ZIHB	116 - 120	119	K-->I
2ZIHB	116 - 120	119	K-->L
2ZIHB	116 - 120	119	K-->N
2ZIHB	116 - 120	119	K-->R
2ZIHB	116 - 120	119	K-->V
2ZIHB	116 - 120	120	R-->S
2ZIHB	116 - 120	120	R-->V
2JEUA	99 - 103	99	W-->D
2JEUA	99 - 103	102	Y-->A
2JEUA	99 - 103	102	Y-->D
2JEUA	99 - 103	102	Y-->P
2JEUA	99 - 103	103	M-->A
1JB1A	283 - 288	287	K-->I
2VHFB	936 - 940	936	R-->A
2VHFB	936 - 940	936	R-->F
2VHFB	936 - 940	936	R-->W
2VHFB	936 - 940	937	R-->D
2VHFB	936 - 940	937	R-->F

2VHFB	936 - 940	937	R-->G
2VHFB	936 - 940	937	R-->I
2VHFB	936 - 940	937	R-->L
2VHFB	936 - 940	937	R-->S
2VHFB	936 - 940	937	R-->Y
2VHFB	936 - 940	939	L-->F
2VHFB	936 - 940	939	L-->G
2VHFB	936 - 940	939	L-->R
2VHFB	936 - 940	939	L-->Y
2VHFB	936 - 940	940	C-->D
2VHFB	936 - 940	940	C-->K
2VHFB	936 - 940	940	C-->L
2VHFB	936 - 940	940	C-->P
2VHFB	936 - 940	940	C-->Q
2VHFB	936 - 940	940	C-->T
2VHFB	936 - 940	940	C-->V
2VHFB	936 - 940	940	C-->W
2J5XA	68 - 73	68	D-->M
2J5XA	68 - 73	72	P-->D
2CLYB	12 - 16	12	W-->A
2CLYB	12 - 16	12	W-->D
2CLYB	12 - 16	12	W-->F
2CLYB	12 - 16	12	W-->G
2CLYB	12 - 16	12	W-->H
2CLYB	12 - 16	12	W-->K
2CLYB	12 - 16	12	W-->L
2CLYB	12 - 16	12	W-->Q

2CLYB	12	16	12	W-->R
2CLYB	12	16	12	W-->S
2CLYB	12	16	12	W-->T
2CLYB	12	16	12	W-->V
2CLYB	12	16	13	V-->D
2CLYB	12	16	13	V-->L
2CLYB	12	16	13	V-->Q
2CLYB	12	16	13	V-->S
2CLYB	12	16	14	A-->R
2CLYB	12	16	14	A-->T
2CLYB	12	16	15	F-->G
2CLYB	12	16	15	F-->K
2CLYB	12	16	15	F-->L
2CLYB	12	16	15	F-->T
2CLYB	12	16	15	F-->Y
2CLYB	12	16	16	G-->E
2CLYB	12	16	16	G-->I
2CLYB	12	16	16	G-->P
2CLYB	53	58	57	K-->S
1THJA	176	180	176	Q-->A
1THJA	176	180	176	Q-->G
1THJA	176	180	176	Q-->I
1THJA	176	180	176	Q-->T
1THJA	176	180	177	A-->D
1THJA	176	180	177	A-->E
1THJA	176	180	177	A-->G
1THJA	176	180	177	A-->I

1THJA	176 - 180	177	A-->K
1THJA	176 - 180	177	A-->L
1THJA	176 - 180	177	A-->T
1THJA	176 - 180	178	E-->A
1THJA	176 - 180	178	E-->F
1THJA	176 - 180	178	E-->G
1THJA	176 - 180	178	E-->H
1THJA	176 - 180	178	E-->I
1THJA	176 - 180	178	E-->L
1THJA	176 - 180	178	E-->S
1THJA	176 - 180	179	A-->D
1THJA	176 - 180	179	A-->E
1THJA	176 - 180	179	A-->F
1THJA	176 - 180	179	A-->G
1THJA	176 - 180	179	A-->H
1THJA	176 - 180	179	A-->N
1THJA	176 - 180	179	A-->P
1THJA	176 - 180	179	A-->S
1THJA	176 - 180	179	A-->W
1THJA	176 - 180	180	D-->A
1THJA	176 - 180	180	D-->F
1THJA	176 - 180	180	D-->G
1THJA	176 - 180	180	D-->H
1THJA	176 - 180	180	D-->K
1THJA	176 - 180	180	D-->L
1THJA	176 - 180	180	D-->T
1THJA	176 - 180	180	D-->V

1THJA	176 - 180	180	D-->Y	
1M7UB	127 - 132	127	L-->E	
1M7UB	127 - 132	127	L-->M	
2PQJA	130 - 134	130	Y-->K	
2PQJA	130 - 134	130	Y-->N	
2PQJA	130 - 134	130	Y-->S	
2PQJA	130 - 134	131	Q-->F	
2PQJA	130 - 134	131	Q-->I	
2PQJA	130 - 134	131	Q-->K	
2PQJA	130 - 134	131	Q-->L	
2PQJA	130 - 134	131	Q-->P	
2PQJA	130 - 134	131	Q-->R	
2PQJA	130 - 134	132	D-->A	
2PQJA	130 - 134	132	D-->G	
2PQJA	130 - 134	132	D-->N	
2PQJA	130 - 134	132	D-->Y	
2PQJA	130 - 134	133	L-->S	
2PQJA	130 - 134	133	L-->T	
2PQJA	130 - 134	133	L-->V	
2PQJA	130 - 134	134	I-->N	
2PQJA	130 - 134	134	I-->S	
2PQJA	130 - 134	134	I-->T	
2PQJA	232 - 238	232	V-->D	
1T6YB	412 - 417	414	F-->R	
1T6YB	412 - 417	415	L-->I	
1T6YB	412 - 417	416	R-->L	
1T6YB	412 - 417	416	R-->Q	

1T6YB	452 - 456	452	I-->D
1T6YB	452 - 456	452	I-->E
1T6YB	452 - 456	452	I-->G
1T6YB	452 - 456	452	I-->R
1T6YB	452 - 456	452	I-->V
1T6YB	452 - 456	452	I-->W
1T6YB	452 - 456	452	I-->Y
1T6YB	452 - 456	453	P-->G
1T6YB	452 - 456	453	P-->K
1T6YB	452 - 456	453	P-->M
1T6YB	452 - 456	453	P-->T
1T6YB	452 - 456	454	A-->G
1T6YB	452 - 456	454	A-->K
1T6YB	452 - 456	454	A-->P
1T6YB	452 - 456	454	A-->S
1T6YB	452 - 456	454	A-->V
1T6YB	452 - 456	455	Y-->C
1T6YB	452 - 456	455	Y-->D
1T6YB	452 - 456	455	Y-->G
1T6YB	452 - 456	455	Y-->H
1T6YB	452 - 456	455	Y-->L
1T6YB	452 - 456	455	Y-->N
1T6YB	452 - 456	455	Y-->P
1T6YB	452 - 456	455	Y-->Q
1T6YB	452 - 456	455	Y-->R
1T6YB	452 - 456	455	Y-->T
1T6YB	452 - 456	455	Y-->V

1T6YB	452 - 456	456	L-->E
1T6YB	452 - 456	456	L-->I
1T6YB	452 - 456	456	L-->K
1T6YB	452 - 456	456	L-->T
1B04A	145 - 149	145	T-->A
1B04A	145 - 149	145	T-->D
1B04A	145 - 149	145	T-->G
1B04A	145 - 149	145	T-->I
1B04A	145 - 149	145	T-->K
1B04A	145 - 149	145	T-->N
1B04A	145 - 149	145	T-->P
1B04A	145 - 149	146	E-->C
1B04A	145 - 149	146	E-->D
1B04A	145 - 149	146	E-->G
1B04A	145 - 149	146	E-->L
1B04A	145 - 149	146	E-->T
1B04A	145 - 149	146	E-->V
1B04A	145 - 149	146	E-->Y
1B04A	145 - 149	147	N-->D
1B04A	145 - 149	147	N-->F
1B04A	145 - 149	147	N-->G
1B04A	145 - 149	147	N-->I
1B04A	145 - 149	147	N-->L
1B04A	145 - 149	147	N-->P
1B04A	145 - 149	147	N-->T
1B04A	145 - 149	147	N-->V
1B04A	145 - 149	148	L-->C

1B04A	145 - 149	148	L-->D
1B04A	145 - 149	148	L-->E
1B04A	145 - 149	148	L-->G
1B04A	145 - 149	148	L-->N
1B04A	145 - 149	148	L-->V
1B04A	145 - 149	149	K-->E
1B04A	145 - 149	149	K-->G
1B04A	145 - 149	149	K-->I
1B04A	145 - 149	149	K-->R
1B04A	145 - 149	149	K-->S
1B04A	145 - 149	149	K-->Y
1B04A	208 - 213	208	P-->V
1B04A	208 - 213	209	K-->F
1B04A	208 - 213	211	A-->G
1IAJB	1650 - 1654	1650	P-->A
1IAJB	1650 - 1654	1650	P-->C
1IAJB	1650 - 1654	1650	P-->G
1IAJB	1650 - 1654	1650	P-->H
1IAJB	1650 - 1654	1650	P-->K
1IAJB	1650 - 1654	1650	P-->L
1IAJB	1650 - 1654	1650	P-->N
1IAJB	1650 - 1654	1650	P-->Q
1IAJB	1650 - 1654	1650	P-->R
1IAJB	1650 - 1654	1650	P-->S
1IAJB	1650 - 1654	1650	P-->V
1IAJB	1650 - 1654	1651	E-->A
1IAJB	1650 - 1654	1651	E-->G

1IAJB	1650 - 1654	1651	E-->L
1IAJB	1650 - 1654	1651	E-->P
1IAJB	1650 - 1654	1651	E-->V
1IAJB	1650 - 1654	1651	E-->Y
1IAJB	1650 - 1654	1652	V-->D
1IAJB	1650 - 1654	1652	V-->G
1IAJB	1650 - 1654	1652	V-->I
1IAJB	1650 - 1654	1652	V-->R
1IAJB	1650 - 1654	1652	V-->T
1IAJB	1650 - 1654	1652	V-->W
1IAJB	1650 - 1654	1653	I-->A
1IAJB	1650 - 1654	1653	I-->F
1IAJB	1650 - 1654	1653	I-->G
1IAJB	1650 - 1654	1653	I-->N
1IAJB	1650 - 1654	1653	I-->R
1IAJB	1650 - 1654	1653	I-->S
1IAJB	1650 - 1654	1654	N-->P
1IAJB	1650 - 1654	1654	N-->S
1IAJB	1812 - 1816	1812	S-->L
1IAJB	1812 - 1816	1814	C-->F
1IAJB	1812 - 1816	1814	C-->I
1IAJB	1812 - 1816	1814	C-->Y
1IAJB	1812 - 1816	1815	R-->A
1IAJB	1812 - 1816	1816	K-->F
1IAJB	1812 - 1816	1816	K-->S
1JX7A	43 - 49	44	D-->A
1JX7A	65 - 70	69	T-->R

1JX7A	65	70	70	A-->G
1JX7A	79	84	79	K-->W
1JX7A	79	84	81	C-->A
1JX7A	79	84	81	C-->I
1NXHA	11	15	11	K-->A
1NXHA	11	15	11	K-->E
1NXHA	11	15	11	K-->G
1NXHA	11	15	11	K-->H
1NXHA	11	15	11	K-->L
1NXHA	11	15	11	K-->N
1NXHA	11	15	11	K-->Q
1NXHA	11	15	11	K-->R
1NXHA	11	15	11	K-->S
1NXHA	11	15	12	R-->F
1NXHA	11	15	12	R-->G
1NXHA	11	15	12	R-->H
1NXHA	11	15	12	R-->I
1NXHA	11	15	12	R-->L
1NXHA	11	15	12	R-->P
1NXHA	11	15	12	R-->S
1NXHA	11	15	12	R-->T
1NXHA	11	15	12	R-->V
1NXHA	11	15	12	R-->Y
1NXHA	11	15	13	R-->A
1NXHA	11	15	13	R-->C
1NXHA	11	15	13	R-->D
1NXHA	11	15	13	R-->G

1NXHA	11 - 15	13	R-->L
1NXHA	11 - 15	13	R-->P
1NXHA	11 - 15	13	R-->S
1NXHA	11 - 15	13	R-->T
1NXHA	11 - 15	13	R-->V
1NXHA	11 - 15	14	I-->P
1NXHA	11 - 15	14	I-->Q
1NXHA	11 - 15	14	I-->R
1NXHA	11 - 15	14	I-->T
1NXHA	11 - 15	14	I-->V
1NXHA	11 - 15	15	L-->G
1NXHA	11 - 15	15	L-->K
1NXHA	11 - 15	15	L-->M
1NXHA	11 - 15	15	L-->P
1NXHA	11 - 15	15	L-->R
1NXHA	11 - 15	15	L-->T
2VQBA	91 - 95	91	F-->E
2VQBA	91 - 95	91	F-->L
2VQBA	91 - 95	91	F-->P
2VQBA	91 - 95	91	F-->R
2VQBA	91 - 95	92	D-->R
2VQBA	91 - 95	93	E-->A
2VQBA	91 - 95	93	E-->C
2VQBA	91 - 95	93	E-->T
2VQBA	91 - 95	94	H-->E
2VQBA	91 - 95	94	H-->F
2VQBA	91 - 95	94	H-->K

2VQBA	91	95	94	H-->N
2VQBA	91	95	94	H-->S
2VQBA	91	95	94	H-->T
2VQBA	91	95	94	H-->Y
2VQBA	91	95	95	F-->A
2VQBA	91	95	95	F-->C
2VQBA	91	95	95	F-->G
2VQBA	91	95	95	F-->R
2VQBA	91	95	95	F-->T
2VQBA	91	95	95	F-->Y
2VQBA	445	449	445	L-->A
2VQBA	445	449	445	L-->D
2VQBA	445	449	445	L-->G
2VQBA	445	449	445	L-->N
2VQBA	445	449	445	L-->P
2VQBA	445	449	445	L-->Q
2VQBA	445	449	445	L-->R
2VQBA	445	449	445	L-->T
2VQBA	445	449	445	L-->Y
2VQBA	445	449	446	E-->G
2VQBA	445	449	446	E-->I
2VQBA	445	449	446	E-->K
2VQBA	445	449	446	E-->L
2VQBA	445	449	446	E-->N
2VQBA	445	449	446	E-->T
2VQBA	445	449	447	A-->D
2VQBA	445	449	447	A-->F

2VQBA	445 - 449	447	A-->G
2VQBA	445 - 449	447	A-->I
2VQBA	445 - 449	447	A-->L
2VQBA	445 - 449	447	A-->M
2VQBA	445 - 449	447	A-->N
2VQBA	445 - 449	447	A-->W
2VQBA	445 - 449	448	Y-->E
2VQBA	445 - 449	448	Y-->F
2VQBA	445 - 449	448	Y-->G
2VQBA	445 - 449	448	Y-->I
2VQBA	445 - 449	448	Y-->K
2VQBA	445 - 449	448	Y-->L
2VQBA	445 - 449	448	Y-->M
2VQBA	445 - 449	448	Y-->N
2VQBA	445 - 449	448	Y-->P
2VQBA	445 - 449	448	Y-->R
2VQBA	445 - 449	448	Y-->S
2VQBA	445 - 449	448	Y-->T
2VQBA	445 - 449	448	Y-->V
2VQBA	445 - 449	449	L-->E
2VQBA	445 - 449	449	L-->G
2VQBA	445 - 449	449	L-->H
2VQBA	445 - 449	449	L-->N
2VQBA	445 - 449	449	L-->R
2F7LA	360 - 365	361	A-->D
2F7LA	360 - 365	364	F-->D
2F7LA	360 - 365	365	D-->P

2F7LA	360 - 365	365	D-->Y
1T7SA	88 - 92	88	D-->A
1T7SA	88 - 92	88	D-->E
1T7SA	88 - 92	88	D-->F
1T7SA	88 - 92	88	D-->G
1T7SA	88 - 92	88	D-->H
1T7SA	88 - 92	88	D-->I
1T7SA	88 - 92	88	D-->N
1T7SA	88 - 92	88	D-->P
1T7SA	88 - 92	88	D-->Q
1T7SA	88 - 92	88	D-->R
1T7SA	88 - 92	89	A-->D
1T7SA	88 - 92	89	A-->F
1T7SA	88 - 92	89	A-->G
1T7SA	88 - 92	89	A-->I
1T7SA	88 - 92	89	A-->L
1T7SA	88 - 92	90	G-->I
1T7SA	88 - 92	91	F-->A
1T7SA	88 - 92	91	F-->G
1T7SA	88 - 92	91	F-->I
1T7SA	88 - 92	91	F-->P
1T7SA	88 - 92	91	F-->V
1T7SA	88 - 92	92	K-->A
1T7SA	88 - 92	92	K-->C
1T7SA	88 - 92	92	K-->D
1T7SA	88 - 92	92	K-->E
1T7SA	88 - 92	92	K-->F

1T7SA	88	92	92	K-->G
1T7SA	88	92	92	K-->N
1T7SA	88	92	92	K-->R
1T7SA	88	92	92	K-->T
1T7SA	88	92	92	K-->V
1T7SA	126	131	128	K-->L
1T7SA	126	131	129	Q-->P
2J14B	314	319	318	R-->K
2J14B	431	436	432	L-->K
2J14B	431	436	434	Q-->G
2J14B	431	436	434	Q-->V
1DKGA	88	94	91	F-->N
1XE8B	191	196	196	E-->L
2R7PA	55	60	59	K-->I
2R7PA	97	102	97	N-->H
2R7PA	97	102	97	N-->T
2R7PA	97	102	99	S-->F
2R7PA	97	102	100	N-->E
2R7PA	97	102	100	N-->G
1I1AC	247	251	247	T-->D
1I1AC	247	251	247	T-->E
1I1AC	247	251	247	T-->F
1I1AC	247	251	247	T-->G
1I1AC	247	251	247	T-->I
1I1AC	247	251	247	T-->L
1I1AC	247	251	247	T-->P
1I1AC	247	251	247	T-->R

1I1AC	247 - 251	248	K-->A
1I1AC	247 - 251	248	K-->F
1I1AC	247 - 251	248	K-->G
1I1AC	247 - 251	248	K-->H
1I1AC	247 - 251	248	K-->I
1I1AC	247 - 251	248	K-->L
1I1AC	247 - 251	248	K-->N
1I1AC	247 - 251	248	K-->P
1I1AC	247 - 251	248	K-->Q
1I1AC	247 - 251	248	K-->R
1I1AC	247 - 251	248	K-->T
1I1AC	247 - 251	248	K-->V
1I1AC	247 - 251	249	D-->A
1I1AC	247 - 251	249	D-->F
1I1AC	247 - 251	249	D-->G
1I1AC	247 - 251	249	D-->I
1I1AC	247 - 251	249	D-->K
1I1AC	247 - 251	249	D-->L
1I1AC	247 - 251	249	D-->M
1I1AC	247 - 251	249	D-->P
1I1AC	247 - 251	249	D-->R
1I1AC	247 - 251	249	D-->S
1I1AC	247 - 251	249	D-->V
1I1AC	247 - 251	249	D-->Y
1I1AC	247 - 251	250	V-->D
1I1AC	247 - 251	250	V-->I
1I1AC	247 - 251	250	V-->K

1I1AC	247 - 251	250	V-->L
1I1AC	247 - 251	250	V-->P
1I1AC	247 - 251	250	V-->Q
1I1AC	247 - 251	251	L-->D
1I1AC	247 - 251	251	L-->G
1I1AC	247 - 251	251	L-->I
1I1AC	247 - 251	251	L-->N
1I1AC	247 - 251	251	L-->P
1I1AC	247 - 251	251	L-->T
1I1AC	247 - 251	251	L-->Y
1I1AC	310 - 314	310	H-->D
1I1AC	310 - 314	310	H-->G
1I1AC	310 - 314	310	H-->I
1I1AC	310 - 314	310	H-->L
1I1AC	310 - 314	310	H-->S
1I1AC	310 - 314	312	D-->F
1I1AC	310 - 314	312	D-->G
1I1AC	310 - 314	312	D-->V
1I1AC	310 - 314	313	W-->A
1I1AC	310 - 314	313	W-->I
1I1AC	310 - 314	313	W-->P
1I1AC	310 - 314	314	L-->P
1I1AC	414 - 418	414	K-->G
1I1AC	414 - 418	414	K-->V
1I1AC	414 - 418	416	T-->V
1I1AC	414 - 418	416	T-->Y
1I1AC	414 - 418	417	W-->G

1I1AC	414 - 418	417	W-->K	
1I1AC	414 - 418	417	W-->L	
1I1AC	414 - 418	417	W-->V	
1I1AC	414 - 418	418	Q-->D	
1I1AC	414 - 418	418	Q-->E	
1I1AC	414 - 418	418	Q-->G	
1I1AC	414 - 418	418	Q-->H	
1I1AC	414 - 418	418	Q-->K	
1I1AC	414 - 418	418	Q-->T	
1I1AC	414 - 418	418	Q-->Y	
1IZYA	116 - 121	117	M-->P	
1IZYA	116 - 121	117	M-->V	
1IZYA	116 - 121	119	F-->G	
1IZYA	116 - 121	119	F-->I	
1IZYA	124 - 128	124	K-->D	
1IZYA	124 - 128	124	K-->E	
1IZYA	124 - 128	124	K-->G	
1IZYA	124 - 128	124	K-->P	
1IZYA	124 - 128	124	K-->S	
1IZYA	124 - 128	124	K-->T	
1IZYA	124 - 128	125	S-->A	
1IZYA	124 - 128	125	S-->D	
1IZYA	124 - 128	125	S-->E	
1IZYA	124 - 128	125	S-->F	
1IZYA	124 - 128	125	S-->G	
1IZYA	124 - 128	125	S-->N	
1IZYA	124 - 128	125	S-->P	

1IZYA	124	128	125	S-->T
1IZYA	124	128	125	S-->V
1IZYA	124	128	125	S-->Y
1IZYA	124	128	126	L-->D
1IZYA	124	128	126	L-->E
1IZYA	124	128	126	L-->Q
1IZYA	124	128	126	L-->T
1IZYA	124	128	126	L-->V
1IZYA	124	128	127	F-->D
1IZYA	124	128	127	F-->G
1IZYA	124	128	127	F-->K
1IZYA	124	128	127	F-->N
1IZYA	124	128	127	F-->Q
1IZYA	124	128	127	F-->S
1IZYA	124	128	127	F-->T
1IZYA	124	128	127	F-->Y
1IZYA	124	128	128	R-->A
1IZYA	124	128	128	R-->D
1IZYA	124	128	128	R-->G
1IZYA	124	128	128	R-->K
1IZYA	124	128	128	R-->S
1IZYA	124	128	128	R-->V
1IZYA	124	128	128	R-->W
1IZYA	133	138	136	V-->E
1IZYA	133	138	137	V-->N
1IZYA	133	138	138	A-->I
1IZYA	133	138	138	A-->L

1IZYA	133 - 138	138	A-->S
1F2NA	135 - 139	135	T-->D
1F2NA	135 - 139	135	T-->F
1F2NA	135 - 139	135	T-->I
1F2NA	135 - 139	135	T-->K
1F2NA	135 - 139	135	T-->Q
1F2NA	135 - 139	135	T-->R
1F2NA	135 - 139	136	G-->C
1F2NA	135 - 139	136	G-->K
1F2NA	135 - 139	136	G-->V
1F2NA	135 - 139	137	K-->D
1F2NA	135 - 139	137	K-->E
1F2NA	135 - 139	137	K-->F
1F2NA	135 - 139	137	K-->G
1F2NA	135 - 139	137	K-->Q
1F2NA	135 - 139	137	K-->S
1F2NA	135 - 139	137	K-->T
1F2NA	135 - 139	137	K-->Y
1F2NA	135 - 139	138	M-->A
1F2NA	135 - 139	138	M-->D
1F2NA	135 - 139	138	M-->E
1F2NA	135 - 139	138	M-->F
1F2NA	135 - 139	138	M-->G
1F2NA	135 - 139	138	M-->I
1F2NA	135 - 139	138	M-->K
1F2NA	135 - 139	138	M-->L
1F2NA	135 - 139	138	M-->P

1F2NA	135 - 139	138	M-->R
1F2NA	135 - 139	138	M-->S
1F2NA	135 - 139	138	M-->T
1F2NA	135 - 139	138	M-->V
1F2NA	135 - 139	139	S-->D
1F2NA	135 - 139	139	S-->F
1F2NA	135 - 139	139	S-->G
1F2NA	135 - 139	139	S-->I
1F2NA	135 - 139	139	S-->L
1F2NA	135 - 139	139	S-->N
1F2NA	135 - 139	139	S-->P
1F2NA	135 - 139	139	S-->Y
1F2NA	153 - 158	154	E-->C
1F2NA	153 - 158	155	G-->S
1F2NA	153 - 158	157	H-->E
1XPXA	1268 - 1273	1271	L-->P
1XPXA	1268 - 1273	1273	M-->D
